# Thianthrene/TfOH-catalyzed electrophilic halogenations using *N*-halosuccinimides as the halogen source†

**DOI:** 10.1039/d4sc04461d

**Published:** 2024-07-22

**Authors:** Haofeng Shi, Jingran Zhang, Xuemin Li, Jiaxin He, Yuli Sun, Jialiang Wu, Yunfei Du

**Affiliations:** a Tianjin Key Laboratory for Modern Drug Delivery & High-Efficiency, School of Pharmaceutical Science and Technology, Faculty of Medicine, Tianjin University Tianjin 300072 China duyunfeier@tju.edu.cn

## Abstract

Organohalides are vital organic building blocks with applications spanning various fields. However, direct halogenation of certain neutral or unreactive substrates by using solely the regular halogenating reagents has proven challenging. Although various halogenation approaches *via* activating halogenating reagents or substrates have emerged, a catalytic system enabling broad substrate applicability and diverse halogenation types remains relatively underexplored. Inspired by the halogenation of arenes *via* thianthrenation of arenes, here we report that thianthrene, in combined use with trifluoromethanesulfonic acid (TfOH), could work as an effective catalytic system to activate regular halogenating reagents (NXS). This new protocol could accomplish multiple types of halogenation of organic compounds including aromatics, olefins, alkynes and ketones. The mechanism study indicated that a highly reactive electrophilic halogen thianthrenium species, formed *in situ* from the reaction of NXS with thianthrene in the presence of TfOH, was crucial for the efficient halogenation process.

## Abstract 2

A thianthrene (TT)/TfOH-catalyzed electrophilic halogenation has been developed, enabling broad substrate applicability and diverse halogenation types *via* the generation of a TT-based halogenated thianthrenium salt.

## Graphical abstract

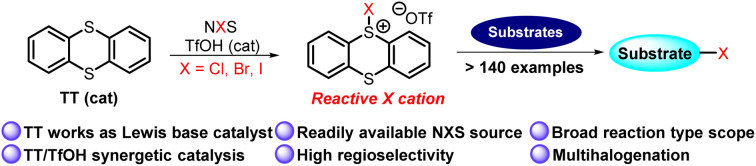

## Introduction

Organohalides undeniably hold a position of paramount importance within the realm of chemicals, finding extensive applications across a diverse spectrum of fields.^1–5^ In the pursuit of synthesizing halogenated aromatics, the employment of bromine (Br_2_) or chlorine (Cl_2_) was highly noticeable as the most direct and utilitarian approach.^6^ Nevertheless, owing to the inherent toxicity and potential risks in handling these particular reagents,^7,8^ various alternative halogenating reagents have been developed and applied, enabling efficient access to many aromatic halides.^9–15^ However, for some unactivated aromatics, the difficulty of realizing an efficient halogenation still exists. Generally, the conventional approaches to tackle the dilemma are to activate either the halogenating reagents or the non-reactive aromatics. Among the multitudinous halogenating reagents, the readily available and commonly used NXS (X = Cl, Br, I), with relatively lower reactivity and selectivity, are still the preferred choice.^16,17^ Accordingly, enhancing the reactivity and selectivity of NXS for halogenation reactions has attracted a great deal of interest from organic researchers and a series of efficient methods have been developed^18–22^ so far.

**Fig. 1:**
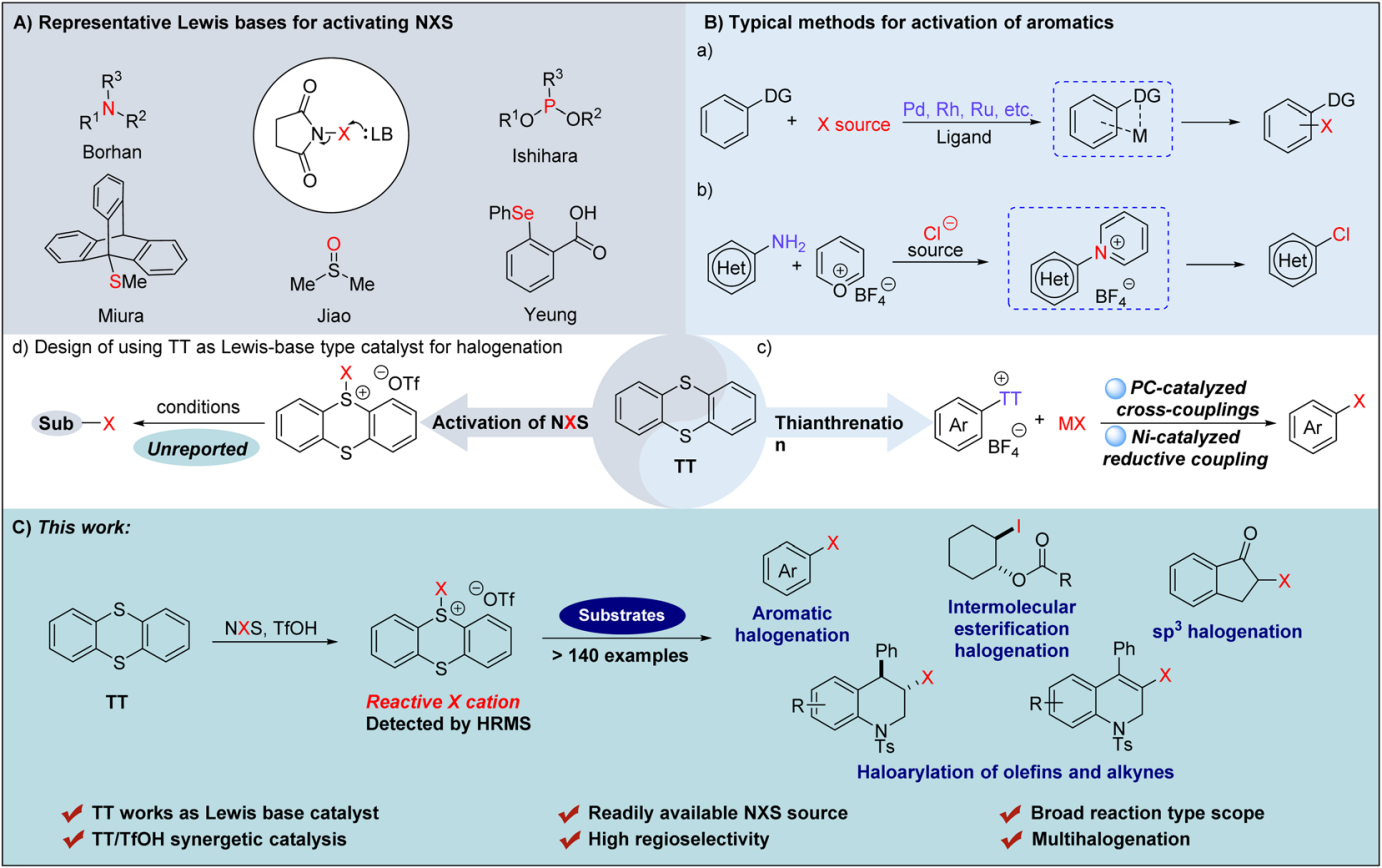
(A) Representative approaches of activating NXS by Lewis bases. (B) Typical methods for activating aromatics. (C) Application of the catalytic halogenation catalzyed by TT and TfOH (this work).

To our knowledge, the mechanisms driving the augmentation of NXS reactivity can be categorized into four catalytic modes that are respectively enabled by Brønsted acids,^23–25^ Lewis acids,^26–29^ halogen bond reagents^30,31^ and Lewis bases.^32–34^ Among them, the catalytic systems promoted by Lewis bases have gained significant attention and a series of reagents have been proved to be effective for activating NXS (Fig. 1A). Mechanistically, the nucleophilic nitrogen,^35–37^ phosphorus,^38^ oxygen,^39,40^ sulfur^41–47^ or selenium^48–50^ atoms present in Lewis bases interact with halogenating reagents to produce reactive halonium complexes, thus facilitating the catalysis of the halogenation reaction.^51,52^ For example, utilizing (DHQD)_2_PHAL as a chiral Lewis base, Borhan and co-workers^53,54^ developed an NXS-enabled asymmetric chlorolactonization of alkenoic acids. Ishihara^55^ reported NXS-mediated cascade halogenation of polyprenoids using phosphoramidites as the Lewis base. Miura^56^ disclosed triptycenyl sulfide-catalyzed halogenation of aromatics employing NXS as the halogenating reagent. Most strikingly, the readily available DMSO^39^ was employed by Jiao to realize chlorination of a series of aromatics. Furthermore, Yeung and coworkers^50^ prepared α-substituted phenyl selenide, and applied it to the catalytic bromination of multiple types of substrates *via* an exclusive tetrasubstituted neutral hypervalent Se–halogen species. Although multifarious Lewis base catalysts have been employed to realize the activation of NXS,^20^ it is still challenging to accomplish certain halofunctionalization reactions such as the halogenation of unreactive olefins and alkynes. Meanwhile, the Lewis base catalytic system that can concurrently accommodate chlorination, bromination, and iodination has been relatively less explored.^39^ In these regards, the development of alternative Lewis base catalytic systems that can realize efficient versatile halogenations and achieve multi-purpose halogenation, including halogenation of olefins and alkynes, should still be highly desirable.

In addition to activation of NXS, the utilization of reactive aromatic species *via* the activation of aromatics has also been proved to be a robust strategy to realize halogenation of aromatics.^57,58^ Among the existing methods, transition metal induced site-selective C–H halogenation of aromatics bearing directing groups has emerged as a versatile approach for C–X bond formation (Fig. 1B(a)).^59^ For example, utilizing the classical Sandmeyer reaction, Cornella and coworkers^60^ devised a chlorination strategy for converting aromatic sp^2^-C–NH_2_ groups into sp^2^-C–Cl *via* a reactive pyridinium intermediate, generated from the reaction of arenediazonium salts with Pyry-BF_4_ (Fig. 1B(b)). Most strikingly, Ritter and coworkers^61–63^ recently reported that the reaction of aromatic compounds with thianthrene (TT) underwent thianthrenation to give thianthrenium salt intermediates, which could *in situ* generate a reactive aryl radical that can achieve high positional selectivity in C–H halogenation *via* a photo-catalysis pathway (Fig. 1B(c)). This approach has been proved to be a ‘master key’ of LSF (Late-Stage Functionalization) and a series of halogenated aromatics were formed with no requirement of any particular directing groups or specific substitution pattern. In 2023, Cornella and Ritter further extended this concept to halogenation of aryl thianthrenium salts by using a Ni(i)-catalyzed reductive coupling process, which allowed for the synthesis of a triad of halogenated arenes in the same, single catalytic system (Fig. 1B(c)).^64^ Inspired by the pioneering works,^65,66^ we envisaged that in addition to activating aromatic compounds, thianthrene (TT) might also be used as a Lewis base to activate halogenating reagents forming a TT-based halogenated thianthrenium salt to realize catalytic halogenation as a reactive electrophilic halogen species in an efficient metal-free manner (Fig. 1B(d)). Herein, we reported that TT, in combined use with TfOH, could form an exclusive halogenation system to activate NXS, allowing for formation of a triad of halogenated arenes, but also direct C–H halogenation of (hetero)arenes, intermolecular halogenation and esterification, intramolecular haloarylation of olefines and alkynes, and synthesis of α-halogenated ketones (Fig. 1C).

## Results and discussion

Initially, 1-phenylpyrazole (1a) was employed as a model substrate to investigate the halogenation of unactivated aromatic compounds, which were difficult to be halogenated by solely using NXS. When substrate 1a was treated with NCS in the absence of any catalyst and additive, minimal chlorinated product formation was observed (Table 1, entry 1). To our delight, with the introduction of a catalytic amount of TT, the yield of 1a-Cl was prominently improved to 64% (Table 1, entry 2). The previous reports showed that the combination of Brønsted acids could promote the catalysis of thioether type Lewis bases.^56^ We further introduced a catalytic amount of TfOH to the reaction system. To our delight, the highly C-4 selective chlorinated product 1a-Cl was obtained in an excellent 94% yield (Table 1, entry 4). We also found that the chlorination of 1a with NCS in the presence of only catalytic TfOH resulted in the formation of product 1a-Cl in only 51% yield (Table 1, entry 3). This result obviously indicated that the application of both TT and TfOH played a synergistic role that can guarantee the best outcome of this chlorination reaction (Table 1, entry 4). Further screening of other additives showed that none of the additives including BF_3_·Et_2_O, TFA, TsOH and MsOH was superior to TfOH (Table 1, entries 4–8). Subsequently, the catalytic potential of diverse thioethers and sulfoxides was also explored. Among dibenzothiophene (DBT), dibenzothiophene sulfoxide (DBTSO), thianthrene-5-oxide (TTSO) and TT, TT demonstrated the highest catalytic efficiency (Table 1, entries 9–11). Other solvents including MeCN, DCE, and EtOAc were also evaluated, and the results reveal that DCE facilitated the highest yield of 98% (Table 1, entries 12–14). The same protocol could also be well applied to bromination and iodination of 1a utilizing NBS or NIS as halogenating reagents. Specifically, when 1a was subjected to the standard conditions by solely replacing NCS with NBS and NIS, the corresponding products 1a-Br and 1a-I were gained with yields of 97% and 93% respectively (Table 1, entries 17 and 18). It is worth noting that when 1a was solely treated with NBS or NIS, the conversion was incomplete and the corresponding halogenated products were obtained in much lower yield (Table 1, entries 15 and 16). Ultimately, the optimized reaction conditions were identified as follows: 1.0 equiv. of substrate, 1.2 equiv. of NXS, 5.0 mol% of TT and TfOH in DCE stirred at rt for 2 h.

**Table 1:** Optimization studies^a^

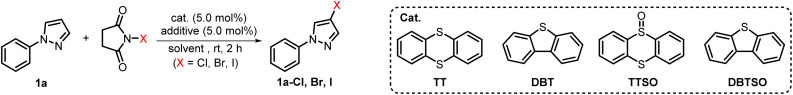											
Entry	Solvent	X source	Cat.	Additive	Yield^b^	Entry	Solvent	X source	Cat.	Additive	Yield^b^
1	DCM	NCS	—	—	Trace	10	DCM	NCS	DBTSO	TfOH	37%
2	DCM	NCS	TT	—	64%	11	DCM	NCS	TTSO	TfOH	64%
3	DCM	NCS	—	TfOH	51%	12	MeCN	NCS	TT	TfOH	90%
4	DCM	NCS	TT	TfOH	94%	13	DCE	NCS	TT	TfOH	98%
5	DCM	NCS	TT	BF_3_·Et_2_O	43%	14	EA	NCS	TT	TfOH	92%
6	DCM	NCS	TT	TFA	58%	15	DCE	NBS	—	—	29%
7	DCM	NCS	TT	TsOH	66%	16	DCE	NIS	—	—	23%
8	DCM	NCS	TT	MsOH	73%	17	DCE	NBS	TT	TfOH	97%
9	DCM	NCS	DBT	TfOH	61%	18	DCE	NIS	TT	TfOH	93%

a 1a (0.5 mmol), NXS (0.6 mmol), catalyst (5.0 mol%) and additive (5.0 mol%) in solvent (2.0 mL) for 2 h.

b Isolated yield.

**Scheme 1:**
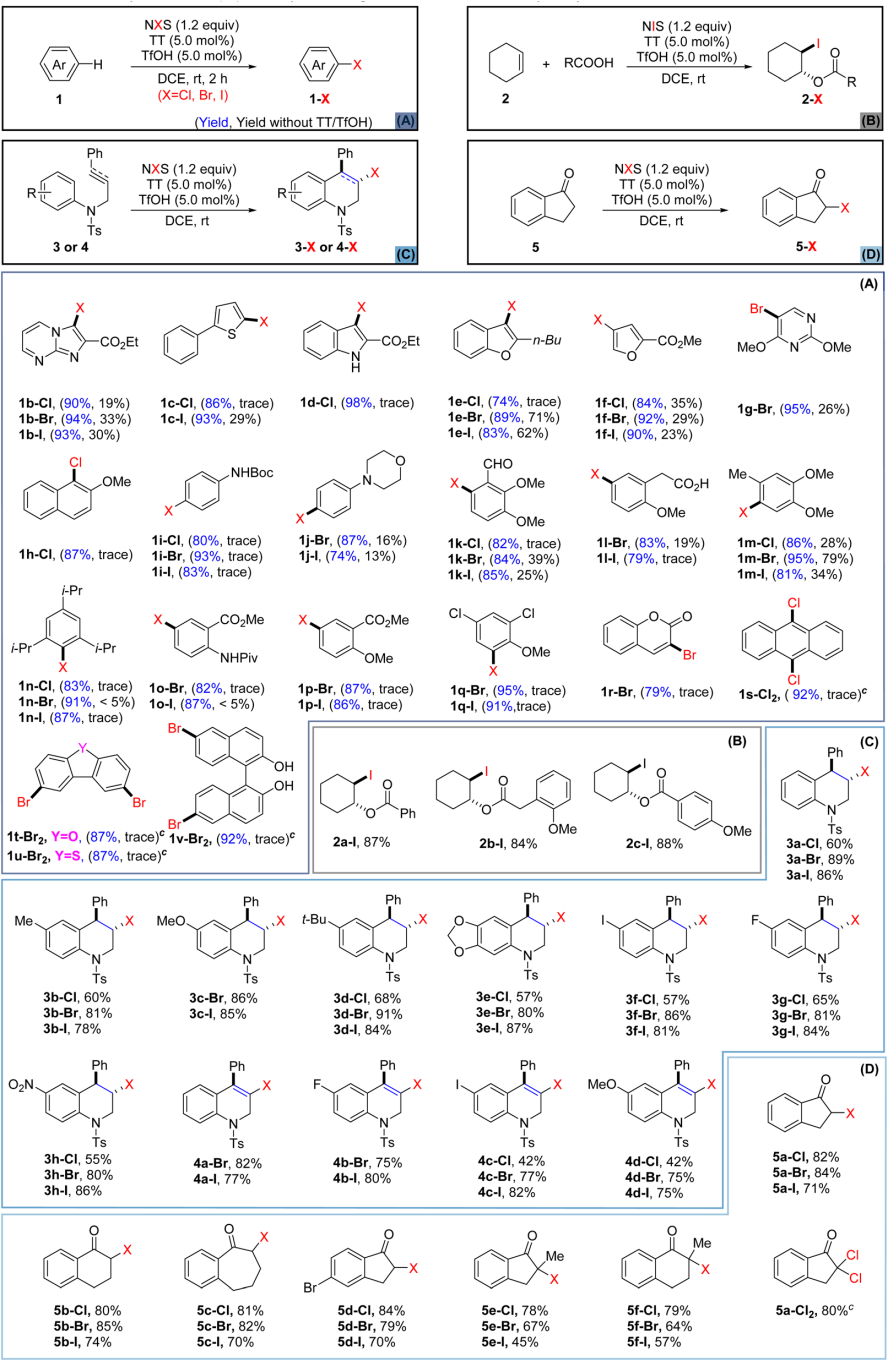
(A) Catalytic halogenation of heteroarenes and arenes;^a,b^ (B) catalytic bifunctionalization of cyclohexene;^b,d,e^ (C) catalytic haloarylation of olefins and alkynes;^a,b,e^ (D) catalytic halogenation of the inactive sp^3^-C position.^a,b,e a^Substrate (0.5 mmol), NXS (0.6 mmol), TT (5.0 mol%) and TfOH (5.0 mol%) in DCE (2.0 mL). ^b^Isolated yield. ^c^1.2 mmol of NXS was used. ^d^Cyclohexene (0.5 mmol), carboxylic acid (0.6 mmol), NIS (0.6 mmol), TT (5.0 mol%) and TfOH (5.0 mol%) in DCE (2.0 mL), rt, 6 h. ^e^No reaction without catalysts.

With the optimized conditions in hand, we next applied the newly established halogenating approach to various heteroarenes and arenes (Scheme 1A) that were not readily halogenated using the previously reported methods.^39^ For comparison purposes, we also conducted parallel tests without the use of TT and TfOH. Initially, heteroarenes including imidazole (1b), thiophene (1c), indole (1d), furan (1e–f), and pyrimidine (1g) were investigated. The results showed that thianthrene/TfOH-catalyzed electrophilic halogenation could notably enhance the reaction efficiency while the reaction carried out in the absence of TT/TfOH gave inferior outcomes in all cases. Next, the halogenation of electron-rich aromatics was studied and the results showed that all of them produced single regioselective products in higher yields and within shorter reaction time (1h–n). Anisole and aniline derivatives (1o–q) bearing electron-withdrawing groups also underwent bromination and iodination smoothly to give the corresponding halogenated products, albeit the chlorination was still hard to be achieved. Strikingly, the protocol was also eligible for the bromination of coumarin (1r), yielding α-position-substituted product 1r-Br in satisfactory yield. Furthermore, the TT/TfOH-catalyzed halogenation approach also demonstrated the capacity to accomplish site-selective multihalogenation of unactivated aromatic substrates. For example, when anthracene (1s) was subjected to the standard conditions by using 2.4 equiv. of NCS, 9,10-dichlorinated anthracene 1s-Cl_2_ could be obtained in a high yield. In addition, dibenzofuran (1t) and dibenzothiophene (1u) were also conveniently converted to the corresponding dibrominated products. Furthermore, dibromination at C-6 and C-6′ of BINOL could also be realized by the strategy, paving the path for accessing a library of binaphthyl-based functional molecules. Disappointingly, when this catalytic halogenation approach was applied to arenes bearing strong electron-withdrawing groups, such as F, CN, NO_2_, and CF_3_, the halogenation failed to be achieved (see the ESI† for details).

With the successful application of this newly established protocol to the halogenation of aromatics, we were encouraged to investigate the intermolecular haloesterification of cyclohexene. It is worth mentioning that treating cyclohexene solely with NIS and benzoic acid did not provide any iodinated product. However, with the introduction of TT and TfOH as catalysts, the reaction of cyclohexene with NIS led to the formation of 2a-I, 2b-I, and 2c-I with satisfactory yields (Scheme 1B). Although the replacement of NIS with the less reactive NBS or NCS proved to be ineffective for the corresponding haloesterification, the success of the above iodoesterification testified to the efficacy of this TT/TfOH-enabled catalytic system.

Intramolecular haloarylation of olefins and alkynes holds significance in constructing benzo heterocyclic rings and producing valuable synthetic intermediates, although this process poses challenges.^67^ While some effective strategies for the halogenation of olefins have been established by Barluenga,^68^ Yeung,^30^ and Song,^40^ an approach applicable for both unreactive olefins and alkynes has remained unexplored. The pursuit of alternative efficient strategies accommodating chlorination, bromination, and iodination should still be highly desirable. Consequently, we explored the application of our catalytic protocol to the intramolecular haloarylation of olefins and alkynes (Scheme 1C). Pleasingly, with the application of TT and TfOH as catalysts, the halogenation of olefins occurred smoothly to furnish the corresponding halogenated tetrahydroquinolines (3a–h, Cl, Br, I) with high chemo- and regioselectivities, albeit the chlorination displayed relatively lower efficacy. To our delight, the protocol could also be well applied to the intramolecular haloarylation of alkynes, as the reaction of alkynes (4) under the standard conditions delivered the halogenated 1,2-dihydroquinoline derivatives (4a–d, Cl, Br, I) in satisfactory yields. It is worth noting that for all the above versatile synthons, the solely use of NXS in the absence of catalysts did not exhibit sufficient activity to enable the transformation.^69–71^ Delightedly, the protocol was equally eligible for the α-halogenation of the ketones that were unreactive with the use of NXS alone. Under the standard conditions, ketones (5) were smoothly converted to the α-halogenated ketones (5a–f, Cl, Br, I). Moreover, α-dichlorination of 5a was also accomplished to give a product (5a-Cl_2_) in a good yield, when 2.0 equivalents of NCS were applied. Late-stage halogenation of natural products or drugs might significantly improve their pharmacological properties, providing an economic path for improving the parent drugs or promoting the development of new medicine.^72,73^ As many pharmaceutical agents bear versatile functional group(s), it might be challenging to achieve efficient halogenation of these bioactive molecules under mild conditions.^74^

**Scheme 2:**
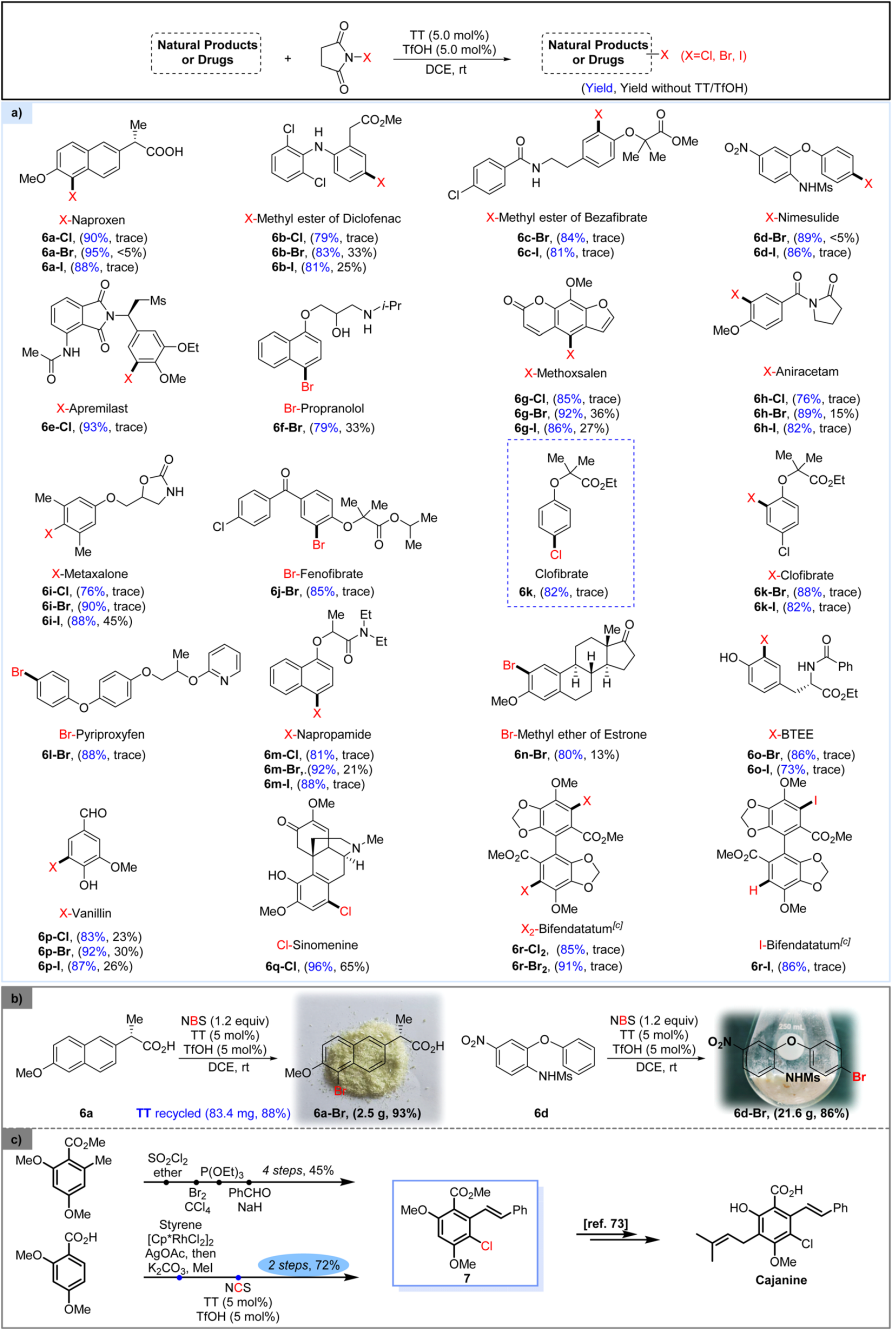
(a) TT/TfOH catalyzed direct halogenation of natural products and drugs;^a,b^ (b) gram-scale reaction and catalyst recovery. (c) Application of TT/TfOH catalyzed halogenation. ^a^Substrate (0.5 mmol), NXS (0.6 mmol), TT (5.0 mol%) and TfOH (5.0 mol%) in DCE (2.0 mL), rt, 6 h. ^b^Isolated yield. ^c^1.2 mmol of NXS was used.

With the thianthrene/TfOH-catalyzed halogenation protocol established, we investigated the direct halogenation of various pharmaceutically active compounds (Scheme 2). Naproxen (6a), featuring the presence of a carboxylic acid group in its structure, underwent smooth halogenation under the standard conditions to give the halogenated products in high yield. The methyl ester of diclofenac (6b), methyl ester of bezafibrate (6c), nimesulide (6d) and apremilast (6e), bearing an aniline or amide in their frameworks, were all well converted to the corresponding halogenated products by the method. Propranolol (6f), containing both an alcohol and an amine group, exhibited enhanced tolerance toward catalytic bromination by the conventional bromination approach. However, by using our protocol, the brominated propranolol 6f-Br could be achieved in a much higher yield. Furthermore, the heterocyclic ring-containing methoxsalen (6g), aniracetam (6h) and metaxalone (6i) could also be successfully halogenated, showcasing the tolerance of the approach to heterocyclic motifs. Bromination of fenofibrate (6j) was also proved to be successful, yielding the corresponding brominated product 6j-Br in good yield. The general chlorination of clofibrate, a drug commonly used for the treatment of transient high triglyceride,^75^ requires the use of chlorine or the more expensive 4-chlorophenol as the starting material.^76^ Here, by treating ethyl 2-methyl-2-phenoxypropanoate with NCS in the presence of TT and TfOH, we accomplished convenient synthesis of clofibrate (6k), which could also be further halogenated by the protocol to give the corresponding *ortho*-brominated or iodinated products (6k-Br, I). Pyriproxyfen (6l) and napropamide (6m), two kinds of pesticides, could also be converted to the corresponding halogenated derivatives in good yields. Notably, when the methyl ether of estrone (6n) was subjected to standard catalytic bromination conditions, the halogenation occurred smoothly to give product 6n-Br with good yield and excellent regioselectivity. Adopting the protocol, bromination and iodination of BTEE (*N*-benzoyl-l-tyrosine ethyl ester, 6o) occurred smoothly to give mono-halogenated products in satisfactory yield. The method was also applicable to the halogenation of naturally occurring vanillin (6p) and sinomenine (6q), as the corresponding halogenated derivatives could be achieved in satisfactory yields. Dihalogenation of bifendatatum (6r) could also be well accomplished by the method, resulting in a convenient synthesis of dichlorinated (6r-Cl_2_) and dibrominated products (6r-Br_2_). However, the reaction of bifendatatum (6r) with 2.4 equiv. of NIS under catalytic conditions did not give the analogous diiodinated product but afforded the mono-iodinated product 6r-I in good yield. The gram-scale transformation of the method was designed and executed successfully.

The large-scale bromination of both naproxen and nimesulide was successfully implemented to give the corresponding brominated products 6a-Br and 6b-Br in good yields, respectively. Strikingly, the TT catalyst could be recycled in 88% yield and applied for the next round of halogenation without affecting its efficacy (Scheme 2b). The synthetic utility of this catalytic halogenation protocol could be further demonstrated by its application in simplifying the synthetic route of cajanine,^77^ a pharmaceutical agent that has been studied as an anti-hepatitis C virus drug. In the originally reported synthetic pathway,^77^ cajanine was furnished in a total of 7 steps *via* the key intermediate 7, which was synthesized *via* a 4-step approach in 45% overall yield. In the simplified route, this pivotal intermediate 7 could be readily prepared from 2,4-dimethoxybenzoic acid *via* just 2 steps, with an overall 72% yield. Specifically, 2,4-dimethoxybenzoic acid could undergo a one-pot Rh-catalyzed coupling with styrene, followed by methylation to give stilbene carboxylic acid methyl ester.^78^ Subsequently, TT/TfOH catalyzed chlorination of the obtained stilbene carboxylic acid methyl ester conveniently delivered the chlorinated intermediate 7 (Scheme 2c).

**Fig. 2:**
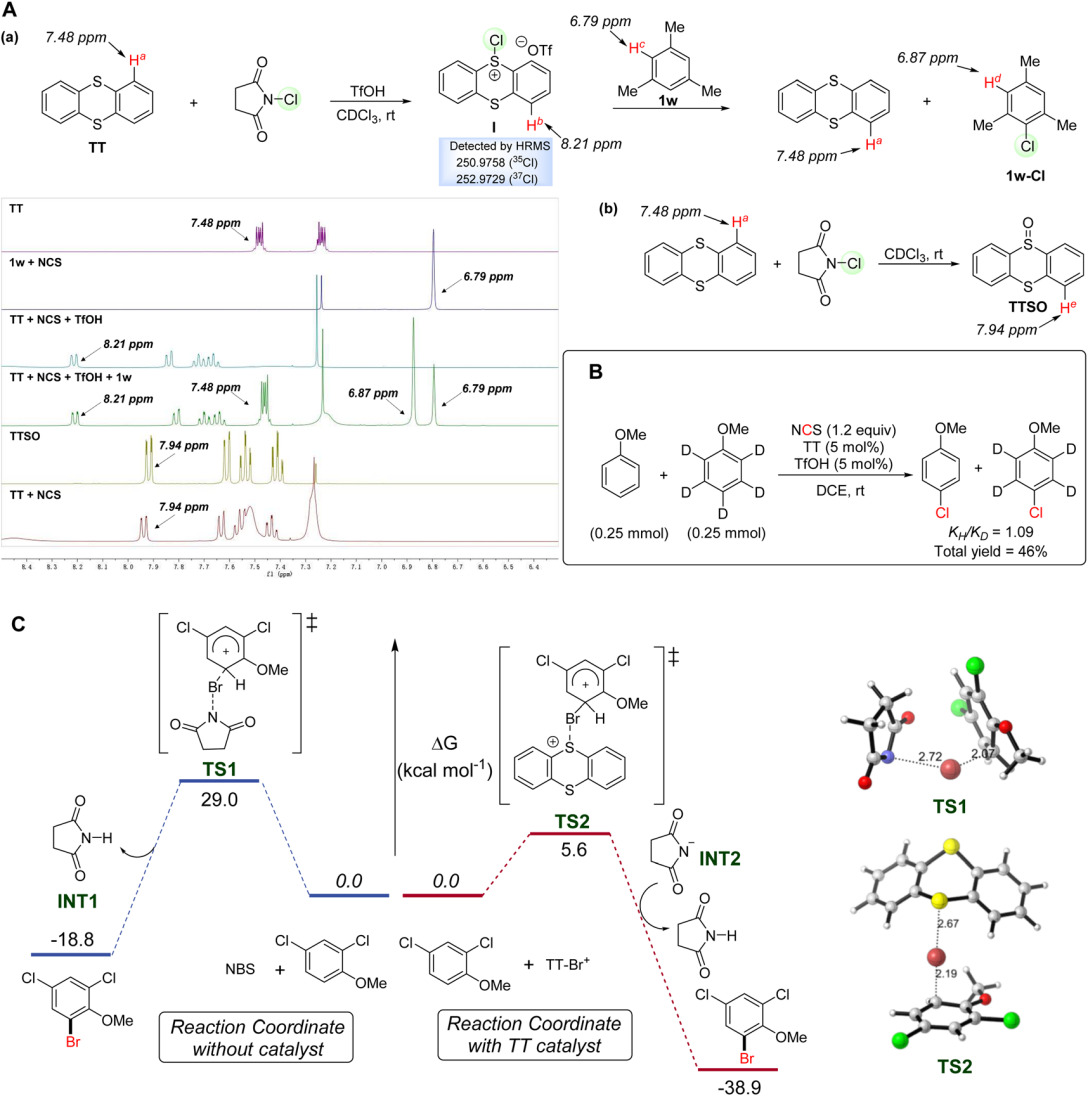
(A) NMR studies of reactive halogenated thianthrenium salts. (B) Kinetic isotope effect (KIE) studies. (C) Computational study of the reaction mechanism.

To investigate the mechanistic pathway of the reaction, several control experiments were carried out. Study on the relationship between yield and time was conducted and the results are shown in the ESI.† Furthermore, competitive reactions and a Hammett plot were established based on the outcomes of the reactions by subjecting anisole and *ortho*-substituted derivatives to the standard conditions. The observed linear relationship indicated that the electron-donating group has a better promoting effect on the chlorination step, which is consistent with the classical electrophilic chlorination process (see the ESI† for details). To clearly understand the mechanism of the catalytic halogenation process, the reaction progress was monitored by ^1^H-NMR (Fig. 2A). Each component was mixed in CDCl_3_ (1.0 mL) for 10 min before the analysis. When substrate 1w was solely treated with NCS, no chlorinated product was observed. However, upon mixing TT and TfOH with NCS in CDCl_3_, a new proton signal at 8.21 ppm appeared and the proton signal at 7.48 ppm (H^a^-TT) disappeared. We tentatively proposed that this result might be caused by the conversion of TT to the reactive intermediate I. Next, we investigated chemical shift changes after adding substrate 1w to the above mixture of TT, TfOH and NCS. The ^1^H-NMR spectrum evidenced the regeneration of TT (H^a^-TT) and formation of product 1w-Cl (H^d^-1w), which might suggest that it was the reactive intermediate I that realized the chlorination of 1w. One might also think that NCS could be used as an oxidant and TT might be oxidized by NCS in air to form TTSO. To rule out the possibility that the new chemical shift was that of TTSO, we treated TT with solely NCS in CDCl_3_. The result showed that the proton signal of TTSO at 7.94 ppm (H^e^-TTSO) could be detected and no chemical shift of 8.21 ppm was observed. In these regards, we tentatively concluded that the reaction of TT with NCS in the presence of TfOH gave the reactive intermediate I, rather than TTSO. Most strikingly, the results of the intermediate capture experiment conducted at −20 °C under an argon atmosphere showed that the reactive chlorinated thianthrenium salt intermediate could be detected by HRMS (see the ESI† for details). This outcome supported that the reaction process might involve the formation of halogenated thianthrenium salts, a sulfonium intermediate similar to those in previous reports.^43^ Kinetic isotope effect (KIE) studies were also carried out and the KIE value of 1.09 evidenced that the cleavage of a C–H bond was not involved in the rate-limiting step, which was also consistent with the conventional electrophilic chlorination mechanism (Fig. 2B).

In order to gain insight into the mode of action of this TT catalyst for halogenation, DFT calculations were performed in Fig. 2C. Based on previously reported works,^39,79^ we carried out the calculation by selecting the sum of the substrate and brominating reagent in different systems as the energetic reference of zero. The sum of the substrate and brominating reagent in different systems was selected as the energetic reference of zero. Without TT catalyst, the electrophilic bromination proceeded *via* transition state TS1 with an insurmountable energy barrier of 29.0 kcal mol^−1^, which is a kinetically uphill process to follow. The C–Br bond forming distance in the TS1 structure is 2.07 Å, indicating that it is a late-stage transition state with a high energy barrier. Later the deprotonation process easily provided the stable product 1q-Br. In the presence of a catalyst, *in situ* generated activated intermediates TT-Br^+^ facilitated the electrophilic bromination process through TS2 (kinetic barrier of 5.6) to afford the brominated product with a significant stabilization in energy. This low activation barrier is in line with the experimental results showing high catalytic activity observed at room temperature. In the structure of TS2, the C–Br bond forming distance is 2.19 Å, indicating that it is an early-state transition state with a low energy barrier. The following deprotonation process also generated product 1q-Br with ease, and the product formation is a highly thermodynamically downhill process. Anisole derivative 1q bearing an electron-withdrawing group gave the desired product 1q-Br with considerably high *ortho*-selectivity. In order to understand the selectivity of the bromination process, both *ortho* substitution and *meta* substitution pathways were evaluated by DFT calculation (see the ESI† for details). The computational results showed that the *meta* substitution pathway bears a higher barrier of electrophilic bromination (kinetic barrier of 11.2), which agrees well with the previously reported studies.^80^

**Scheme 3:**
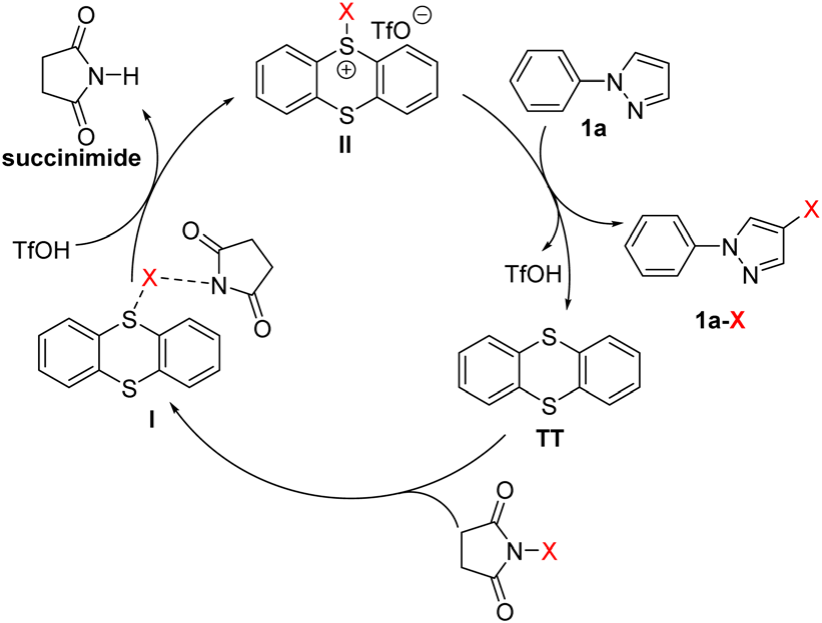
Proposed mechanism.

Based on the aforementioned control experiments and the previous reports,^81–83^ an electrophilic halogenation pathway was postulated to account for the reaction mechanism (Scheme 3). First, TT interacts with NXS to form an unreactive species I, a transition state resulting from the coordination of the succinimide anion with X *via* a halogen bond.^84^ With addition of TfOH, succinimide is then released from I and the reactive sulfonium salt II is simultaneously generated. Finally, the reaction of aromatic compounds, taking 1-phenylpyrazole (1a) as an example, with the electrophilic sulfonium II affords the halogenated aromatic compound 1a-X. TT and TfOH are regenerated in this final step and will enter the next cycle to play synergistic roles.

## Conclusions

In summary, we reported here an alternative NXS-mediated halogenation approach enabled by both TT and TfOH. Differing from the previous halogenation strategy of converting TT to aryl thianthrenium salts^85^ or using DMSO as an oxygen-centered Lewis base^36^ for activating NXS, this work highlights the application of thianthrene as an efficient sulfur-centered Lewis base to generate a TT-based sulfonium salt, a reactive electrophilic halogen species for the halogenation reaction to occur. Utilizing this newly established approach, various types of halogenation reactions including halogenation of aromatics, late-stage halogenation of bioactive molecules, haloarylation of olefins and alkynes, bifunctionalization of cyclohexene and halogenation of inactive sp^3^-C could all be accomplished. The application of this thianthrene/TfOH-catalyzed halogenation method in direct halogenation of various bioactive molecules underscores its efficiency and potential applicability in drug development and modification.

## Data availability

This is to certify that the data supporting this article have been included as part of the ESI,† and all relevant data are within the manuscript and its additional files.

## Author contributions

H. S., J. Z. and Y. D. conceived and designed the experiments. H. S. carried out most of the experiments. J. Z. ran the calculations. X. L., J. H., Y. S. and J. W. analyzed the data. H. S. and Y. D. wrote the paper. J. Z. and Y. D. directed the project.

## Conflicts of interest

There are no conflicts to declare.

## Supplementary Material

SC-015-D4SC04461D-s001
