# Supplementary material for: Thianthrene/TfOH-catalyzed electrophilic halogenations using N-halosuccinimides as the halogen source
Source: Chem Sci. 2024 Jul 22;15(32):13058–67. doi: 10.1039/d4sc04461d (PMC11323329; doi:10.1039/d4sc04461d)

Electronic Supplementary Material (ESI) for Chemical Science.
This journal is © The Royal Society of Chemistry 2024

Thianthrene/TfOH-Catalyzed Electrophilic Halogenations Using *N*-Halosuccinimides as Halogen Source

Haofeng Shi, Jingran Zhang, Xuemin Li, Jiaxin He, Yuli Sun, Jialiang Wu, Yunfei Du*

Tianjin Key Laboratory for Modern Drug Delivery & High-Efficiency, School of Pharmaceutical Science and Technology, Faculty of Medicine, Tianjin University, Tianjin 300072, China.

E-mail: duyunfeier@tju.edu.cn

Supporting Information

**I**	General Information	S1
**II**	Experimental Procedures and Spectroscopic Data	S2-S53
**III**	Mechanistic Studies	S53-S66
**IV**	References	S67
**V**	NMR Spectra of Starting Materials and Products	S68-S358

I. General Information

^1H, ^{13}C and ^{19}F NMR spectra were recorded on a 400 MHz, 500 MHz or 600 MHz spectrometer at 25 °C. Chemical shifts values are given in ppm and referred as the internal standard to TMS: 0.00 ppm. Chemical shifts were expressed in parts per million (*δ*) downfield from the internal standard tetramethylsilane, and were reported as s (singlet), d (doublet), t (triplet), q (quadruple), dd (doublet of doublet), m (multiplet), etc. The coupling constants *J*, are reported in Hertz (Hz). High resolution mass spectrometry (HRMS) data were collected by the Thermo Scientific Q Exactive HF. Melting points were determined with a Micromelting point apparatus. TLC plates were visualized by exposure to ultraviolet light.

Reagents and solvents were purchased as reagent grade and were used without further purification. All reactions were performed in standard glassware, heated at 70 °C for 3 h before used. Flash column chromatography was performed over silica gel (200-300 m) using a mixture of ethyl acetate (EtOAc) and petroleum ether (PE).

II. Experimental Procedures and Spectroscopic Data

Typical procedure for preparing of *N*-cinnamyl-4-methyl-*N*-phenylbenzenesulfonamides 3

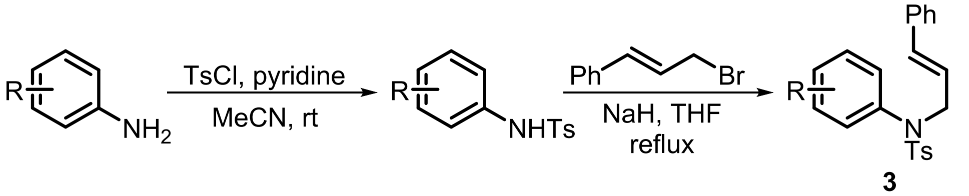

The starting materials **3** were prepared according to literature methods.[1] To a solution of aniline (10 mmol, 1.0 equiv) in MeCN (30 mL), was added pyridine (870.12 mg, 11 mmol, 1.1 equiv) . Then TsCl (2.10 g, 11 mmol, 1.1 equiv) was added slowly and the resulting mixture was stirred at rt overnight. When the reaction was completed, the reaction mixture was quenched with sat. aq. $NaHCO_3$ (50 mL) and extracted with DCM (3 x 20 mL). The combined organic extracts were dried over anhydrous Na_2SO_4, concentrated *in vacuo*. The residue was purified by silica gel flash column chromatography (EtOAc/PE = 1/10) to give the desired 4-methyl-*N*-phenylbenzenesulfonamides products.

To an anhydrous THF (20mL) solution of 4-methyl-*N*-phenylbenzenesulfonamide (5 mmol, 1.0 equiv), was added NaH (599.84 mg, 15 mmol, 3.0 equiv, 60% in mineral oil) portionwise in ice bath. After addition, the mixture was allowed to be stirred at the same temperature for 30 mins. Then an anhydr. THF (20mL) solution of cinnamyl bromide (1.97 g, 10 mmol, 2.0 equiv) was added dropwise to the mixture. After addition, the mixture was heated to reflux. When the reaction was completed, the reaction mixture was poured into ice water and extracted by DCM (3 x 20 mL). The organic phase was washed with sat. aq. NaCl (50 mL) and the combined organic extracts were dried over anhydrous Na_2SO_4, concentrated *in vacuo*. The residue was purified by silica gel flash column chromatography (EtOAc/PE = 1/10) to give the desired products **3**.

Typical procedure for preparing of 4-methyl-*N*-phenyl-*N*-(3-phenylprop-2-yn-1-yl)benzene-sulfonamide 4 [2-3]

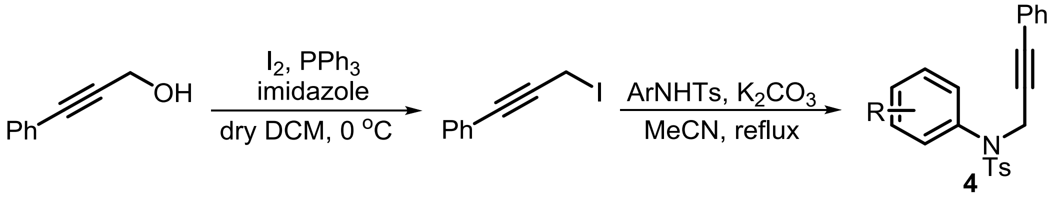

To a light-protected solution (flask wrapped in an aluminum foil) of phenyl propargyl alcohol (2 g, 15.13 mmol, 1.0 equiv) in dry DCM (50mL) under argon atmosphere at 0 °C, were added imidazole (1.55 g, 22.70 mmol, 1.5 equiv), triphenylphosphine (5.95 g, 22.70 mmol, 1.5 equiv) and iodine (5.76 g, 22.70 mmol, 1.5 equiv). The reaction mixture was stirred at 0 °C and the reaction progress was monitored by TLC. After completion of the reaction, the excess of iodine was removed by washing with sat. aq. $Na_2S_2O_3$ solution.

The organic layer was dried over anhydrous Na_2SO_4, concentrated *in vacuo* and the residue was purified by silica gel flashcolumn chromatography (EtOAc/PE = 1/10) to give (3-iodoprop-1-yn-1-yl) benzene.

To a solution of ArNHTs (4.54 mmol, 1.1 equiv) in MeCN (20 mL), were added K_2CO_3 (628.05 mg, 4.54 mmol, 1.1 equiv) and (3-iodoprop-1-yn-1-yl) benzene (1.0 g, 4.13 mmol, 1.0 equiv). The mixture was allowed to be heated to reflux and stirred until the reaction was completed. When the reaction was completed, the reaction mixture was diluted with water and extracted by DCM (3 x 20 mL). The organic layer was washed with sat. aq. NaCl (50 mL), dried over anhydrous Na_2SO_4, and concentrated *in vacuo*. The residue was purified by silica gel flash column chromatography (EtOAc/PE = 1/10) to give products **4**.

4-Methyl-*N*-phenyl-*N*-(3-phenylprop-2-yn-1-yl) benzenesulfonamide (4a)

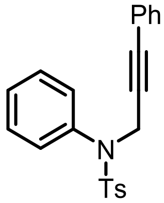

According to the above procedure, **4a** was obtained as a white solid (1.27 g, yield: 85%). mp: 91-93 °C. ^{1}H NMR (400 MHz, $CDCl_3$) δ 7.62 (d, *J* = 8.3 Hz, 2H), 7.36 (s, 5H), 7.32 – 7.27 (m, 3H), 7.23 – 7.17 (m, 4H), 4.69 (s, 2H), 2.39 (s, 3H). ^{13}C NMR (101 MHz, $CDCl_3$) δ 143.5, 139.8, 136.0, 131.5, 129.3, 129.1, 128.5, 128.5, 128.2, 128.2, 128.1, 122.4, 85.6, 83.7, 42.1, 21.6. HRMS (ESI) calcd for $C_{22}H_{20}NO_2S^+$ [M + H^+] 362.1209, found 362.1214.

***N*-(4-Fluorophenyl)-4-methyl-*N*-(3-phenylprop-2-yn-1-yl) benzenesulfonamide (4b)**

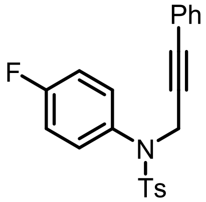

According to the above procedure, **4b** was obtained as colorless oil (1.21 g, yield: 77%). ^{1}H NMR (400 MHz, $CDCl_3$) δ 7.49 (d, *J* = 8.3 Hz, 2H), 7.22 – 7.13 (m, 5H), 7.12 – 7.05 (m, 4H), 6.94 – 6.86 (m, 2H), 4.54 (s, 2H), 2.26 (s, 3H). ^{19}F NMR (376 MHz, $CDCl_3$) δ -112.6.^{13}C NMR (101 MHz, $CDCl_3$) δ 162.2 (*J* = 248.3 Hz), 143.8, 135.7, 135.7 (*J* = 3.1 Hz), 131.5, 130.6 (*J* = 8.7 Hz), 129.4, 128.6, 128.2 (*J* = 16.8 Hz), 122.2, 116.0 (d, *J* = 22.6 Hz), 85.9, 83.4, 42.2, 21.5. HRMS (ESI) calcd for $C_{22}H_{19}FNO_2S^+$ [M + H^+] 380.1115, found 380.1119.

***N*-(4- Iodophenyl)-4-methyl-*N*-(3-phenylprop-2-yn-1-yl) benzenesulfonamide (4c)**

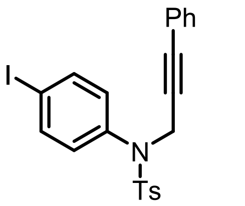

According to the above procedure, **4c** was gained as a yellow solid (1.21 g, yield: 77%). mp: 112-114 °C. ^{1}H NMR (400 MHz, $CDCl_3$) δ 7.68 (d, *J* = 8.6 Hz, 2H), 7.61 (d, *J* = 8.3 Hz, 2H), 7.34 – 7.28 (m, 3H), 7.25 – 7.16 (m, 4H), 7.11 (d, *J* = 8.6 Hz, 2H), 4.66 (s, 2H), 2.39 (s, 3H).^{13}C NMR (101 MHz, $CDCl_3$) δ 143.8, 139.7, 138.2, 135.6, 131.5, 130.2, 129.4, 128.6, 128.3, 128.1, 122.2, 93.6, 86.0, 83.2, 41.8, 21.5. HRMS (ESI) calcd for $C_{22}H_{19}INO_2S^+$ [M + H$^+$] 488.0176, found 488.0177.

***N*-(4- Methoxyphenyl)-4-methyl-*N*-(3-phenylprop-2-yn-1-yl) benzenesulfonamide (4d)**

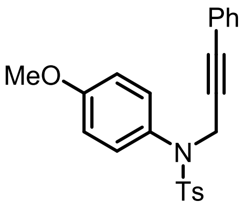

According to the above procedure, **4d** was obtained as a white solid (1.43 g, yield: 87%). mp: 88-90 °C. ^{1}H NMR (400 MHz, $CDCl_3$) δ 7.63 (d, *J* = 8.3 Hz, 2H), 7.35 – 7.28 (m, 3H), 7.26 – 7.18 (m, 6H), 6.90 – 6.82 (m, 2H), 4.65 (s, 2H), 3.82 (s, 3H), 2.40 (s, 3H). ^{13}C NMR (101 MHz, $CDCl_3$) δ 159.4, 143.4, 136.1, 132.3, 131.5, 130.2, 129.25, 128.4, 128.2, 128.2, 122.5, 114.2, 85.5, 83.8, 55.4, 42.3, 21.5. HRMS (ESI) calcd for $C_{23}H_{22}NO_3S^+$ [M + H$^+$] 392.1315, found 392.1320.

General procedure A for halogenation of aromatics: NXS (0.6 mmol, 1.20 equiv, X = Cl, Br, I) and thianthracene (5.41 mg, 0.025 mmol, 0.05 equiv) were dissolved in DCE (3.0 mL). With stirring of the mixture, trifluoromethanesulfonic acid (TfOH) (3.75 mg, 0.025 mmol, 0.05 equiv) was added. Then substrate **1** (0.5 mmol, 1.0 equiv) was added and the mixture was stirred at rt till the reaction was completed. The solvent of the reaction mixture was removed via rotavapor evaporation. The residue was purified by silica gel column chromatography to afford the corresponding halogenated products.

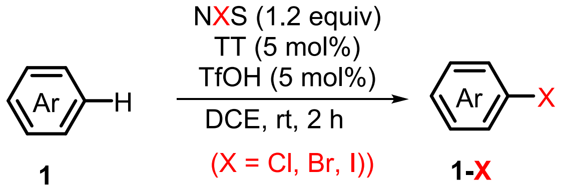

4-Chloro-1-phenyl-1*H*-pyrazole (1a-Cl)

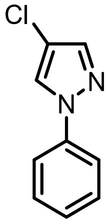

According to the procedure A, **1a-Cl** was gained as a white solid (86.5 mg, yield: 98%). mp: 63-65 °C. ^{1}H NMR (400 MHz, $CDCl_3$) δ 7.91 (s, 1H), 7.63 (d, *J* = 7.3 Hz, 3H), 7.46 (dd, *J* = 10.7, 5.2 Hz, 2H), 7.32 (t, *J* = 7.4 Hz, 1H). ^{13}C NMR (101 MHz, $CDCl_3$) δ 139.5, 129.6, 127.0, 124.8, 119.0, 112.4. HRMS (ESI) calcd for $C_9H_8{}^{35}ClN_2^+$ [M + H$^+$] 179.0371, found 179.0373.

4-Bromo-1-phenyl-1*H*-pyrazole (1a-Br)

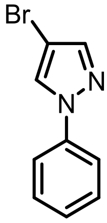

According to the procedure A, **1a-Br** was gained as a white solid (107.2 mg, yield: 97%). mp: 77-79 °C. ^{1}H NMR (400 MHz, $CDCl_3$) δ 7.93 (s, 1H), 7.76 – 7.58 (m, 3H), 7.45 (s, 2H), 7.32 (d, *J* = 6.6 Hz, 1H). ^{13}C NMR (101 MHz, $CDCl_3$) δ 141.6, 139.7, 129.6, 127.1, 119.0, 95.7. HRMS (ESI) calcd for $C_9H_8{}^{79}BrN_2^+$ [M + H$^+$] 222.9865, found 222.9866.

4-Iodo-1-phenyl-1*H*-pyrazole (1a-I)

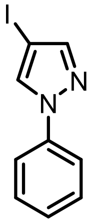

According to the procedure A, **1a-I** was gained as a white solid (124.6 mg, yield: 93%). mp: 69-71 °C. ^{1}H NMR (400 MHz, $CDCl_3$) δ 7.96 (s, 1H), 7.72 (s, 1H), 7.64 (d, *J* = 8.1 Hz, 2H), 7.46 (t, *J* = 7.9 Hz, 2H), 7.32 (t, *J* = 7.4 Hz, 1H). ^{13}C NMR (101 MHz, $CDCl_3$) δ 146.0, 139.5, 131.3, 129.6, 127.1, 119.1, 58.9. HRMS (ESI) calcd for $C_9H_8IN_2^+$ [M + H$^+$] 270.9727, found 270.9729.

Ethyl 3-chloroimidazo[1,2-*a*] pyrimidine-2-carboxylate (1b-Cl)

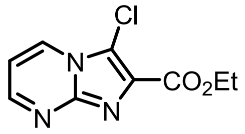

According to the procedure A, **1b-Cl** was obtained as a white solid (101.5 mg, yield: 90%). mp: 164-166 °C. ^{1}H NMR (400 MHz, CDCl$_3$) δ 8.72 (dd, *J* = 3.9, 1.9 Hz, 1H), 8.48 (dd, *J* = 6.9, 1.9 Hz, 1H), 7.12 (dd, *J* = 6.9, 4.0 Hz, 1H), 4.48 (q, *J* = 7.1 Hz, 2H), 1.44 (t, *J* = 7.1 Hz, 3H). ^{13}C NMR (101 MHz, CDCl$_3$) δ 161.7, 152.7, 145.6, 132.1, 131.3, 113.1, 110.6, 61.7, 14.3. HRMS (ESI) calcd for $C_9H_9{}^{35}Cl\ N_3O_2^+$ [M + H$^+$] 226.0378, found 226.0375.

Ethyl 3-bromoimidazo [1,2-*a*] pyrimidine-2-carboxylate (1b-Br)

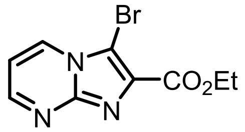

According to the procedure A, **1b-Br** was obtained as a white solid (126.9 mg, yield: 94%). mp: 200-202 °C. ^{1}H NMR (400 MHz, CDCl$_3$) δ 8.68 (dd, *J* = 4.0, 2.0 Hz, 1H), 8.51 (dd, *J* = 6.9, 2.0 Hz, 1H), 7.09 (dd, *J* = 6.9, 4.0 Hz, 1H), 4.46 (q, *J* = 7.1 Hz, 2H), 1.43 (t, *J* = 7.1 Hz, 3H). ^{13}C NMR (101 MHz, CDCl$_3$) δ 162.0, 152.7, 147.6, 135.0, 132.4, 110.74, 98.9, 61.7, 14.3. HRMS (ESI) calcd for $C_9H_9{}^{79}BrN_3O_2^+$ [M + H$^+$] 269.9873, found 269.9877.

Ethyl 3-iodoimidazo [1,2-*a*] pyrimidine-2-carboxylate (1b-I)

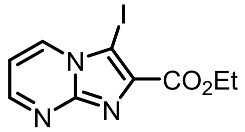

According to the procedure A, **1b-I** was obtained as a white solid (147.4 mg, yield: 93%). mp: 166-168 °C. ^{1}H NMR (400 MHz, CDCl$_3$) δ 8.65 (dd, *J* = 4.0, 2.0 Hz, 1H), 8.55 (dd, *J* = 6.9, 1.9 Hz, 1H), 7.08 (dd, *J* = 6.9, 4.0 Hz, 1H), 4.48 (q, *J* = 7.1 Hz, 2H), 1.45 (t, *J* = 7.1 Hz, 3H). ^{13}C NMR (101 MHz, CDCl$_3$) δ 162.3, 153.1, 150.5, 139.3, 134.9, 111.0, 67.0, 61.8, 14.3. HRMS (ESI) calcd for $C_9H_9IN_3O_2^+$ [M + H$^+$] 317.9734, found 317.9738.

2-Chloro-5-phenylthiophene (1c-Cl)

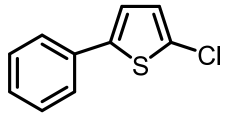

According to the procedure A, **1c-Cl** was gained as a white solid (83.7 mg, yield: 86%). mp: 80-82 °C. ^{1}H NMR (400 MHz, CDCl$_3$) δ 7.53 (d, *J* = 7.4 Hz, 2H), 7.39 (t, *J* = 7.3 Hz, 2H), 7.35 – 7.28 (m, 1H), 7.08

(d, J = 3.5 Hz, 1H), 6.90 (d, J = 3.5 Hz, 1H).^{13}C NMR (101 MHz, $CDCl_3$) δ 143.0, 133.7, 129.2, 129.0, 127.9, 127.1, 125.6, 122.3. HRMS (ESI) calcd for $C_{10}H_8{}^{35}ClS^+$ [M + H^+] 195.0030, found 195.0033.

2-Iodo-5-phenylthiophene (1c-I)

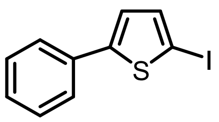

According to the procedure A, **1c-I** was obtained as a green solid (133.1 mg, yield: 93%). mp: 78-80 °C. ^{1}H NMR (400 MHz, $CDCl_3$) δ 7.45 – 7.38 (m, 2H), 7.30 – 7.24 (m, 2H), 7.22 – 7.16 (m, 1H), 7.12 (d, J = 3.8 Hz, 1H), 6.87 (d, J = 3.8 Hz, 1H). ^{13}C NMR (101 MHz, $CDCl_3$) δ 150.5, 137.9, 133.6, 129.0, 128.0, 125.8, 124.6, 72.5. HRMS (ESI) calcd for $C_{10}H_8IS^+$ [M + H^+] 286.9386, found 286.9382.

Ethyl 3-chloro-1*H*-indole-2-carboxylate (1d-Cl)

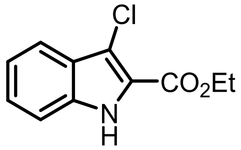

According to the procedure A, **1d-Cl** was obtained as a yellow solid (109.6 mg, yield: 98%). mp: 147-148 °C. ^{1}H NMR (400 MHz, $CDCl_3$) δ 9.02 (s, 1H), 7.72 (dd, J = 8.2, 0.8 Hz, 1H), 7.42 – 7.35 (m, 2H), 7.22 (ddd, J = 8.0, 5.8, 2.1 Hz, 1H), 4.47 (q, J = 7.1 Hz, 2H), 1.46 (t, J = 7.1 Hz, 3H). ^{13}C NMR (101 MHz, CDCl3) δ 161.0, 134.7, 126.6, 126.3, 122.4, 121.3, 120.3, 112.5, 112.1, 61.5, 14.4. HRMS (ESI) calcd for $C_{11}H_{11}{}^{35}ClNO_2{}^+$ [M + H^+] 224.0473, found 224.0476.

2-Butyl-3-chlorobenzofuran (1e-Cl)

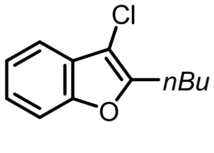

According to the procedure A, **1e-Cl** was obtained as colorless oil (77.2 mg, yield: 74%). ^{1}H NMR (400 MHz, $CDCl_3$) δ 7.58 – 7.52 (m, 1H), 7.49 – 7.42 (m, 1H), 7.35 – 7.28 (m, 2H), 2.87 (t, J = 7.5 Hz, 2H), 1.78 (ddd, J = 13.1, 8.5, 6.6 Hz, 2H), 1.45 (dq, J = 14.7, 7.4 Hz, 2H), 1.01 (t, J = 7.4 Hz, 3H). ^{13}C NMR (101 MHz, $CDCl_3$) δ 154.2, 153.0, 127.1, 124.3, 123.0, 118.3, 111.2, 107.8, 29.6, 25.6, 22.3, 13.8. HRMS (ESI) calcd for $C_{12}H_{14}{}^{35}ClO^+$ [M + H^+] 209.0728, found 209.0727.

3-Bromo-2-butylbenzofuran (1e-Br)

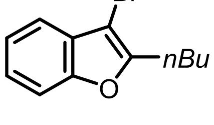

According to the procedure A, **1e-Br** was obtained as colorless oil (112.6 mg, yield: 89%). ^{1}H NMR (400

MHz, $CDCl_3$) δ 7.39 – 7.34 (m, 1H), 7.34 – 7.28 (m, 1H), 7.22 – 7.16 (m, 2H), 2.74 (t, *J* = 7.5 Hz, 2H), 1.73 – 1.59 (m, 2H), 1.39 – 1.26 (m, 2H), 0.87 (t, *J* = 7.4 Hz, 3H). ^{13}C NMR (101 MHz, $CDCl_3$) δ 155.9, 153.5, 128.4, 124.4, 123.1, 119.1, 111.1, 94.2, 29.7, 26.4, 22.2, 13.8. HRMS (ESI) calcd for $C_{12}H_{14}{}^{79}BrO^+$ [M + H^+] 253.0223, found 253.0226.

2-Butyl-3-iodobenzofuran (1e-I)

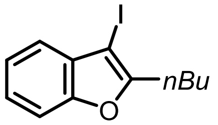

According to the procedure A, **1e-I** was gained as yellow oil (124.5 mg, yield: 83%). ^1H NMR (400 MHz, $CDCl_3$) δ 7.37 (m, 1H), 7.31 (m, 1H), 7.29 – 7.22 (m, 2H), 2.85 (t, *J* = 7.5 Hz, 2H), 1.78 – 1.66 (m, 2H), 1.48 – 1.35 (m, 2H), 0.95 (t, *J* = 7.4 Hz, 3H). ^{13}C NMR (101 MHz, $CDCl_3$) δ 159.2, 154.3, 131.1, 124.5, 123.1, 120.8, 111.0, 62.6, 30.0, 27.7, 22.2, 13.6. HRMS (ESI) calcd for $C_{12}H_{14}IO^+$ [M + H^+] 301.0084, found 301.0088.

Methyl 4-chlorofuran-2-carboxylate (1f-Cl)

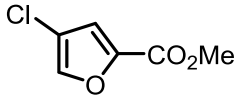

According to the procedure A, **1f-Cl**was gained as a white solid (67.4 mg, yield: 84%). mp: 37-39 °C. ^1H NMR (400 MHz, $CDCl_3$) δ 7.11 (d, *J* = 3.5 Hz, 1H), 6.27 (d, *J* = 3.5 Hz, 1H), 3.84 (s, 3H). ^{13}C NMR (101 MHz, $CDCl_3$) δ 158.0, 143.8, 141.1, 119.9, 108.88, 52.0. HRMS (ESI) calcd for $C_6H_6{}^{35}ClO_3^+$ [M + H^+] 161.0000, found161.0003.

Methyl 4-bromofuran-2-carboxylate (1f-Br)

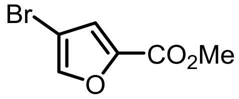

According to the procedure A, **1f- Br** was obtained as colorless oil (94.3 mg, yield: 92%). ^1H NMR (400 MHz, $CDCl_3$) δ 7.11 (d, *J* = 3.5 Hz, 1H), 6.44 (d, *J* = 3.5 Hz, 1H), 3.88 (s, 3H). ^{13}C NMR (101 MHz, $CDCl_3$) δ 158.0, 146.2, 127.5, 120.1, 113.9, 52.1. HRMS (ESI) calcd for $C_6H_6{}^{79}BrO_3^+$ [M + H^+] 204.9495, found 204.9497.

Methyl 4- iodofuran -2-carboxylate (1f-I)

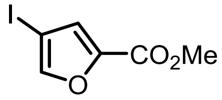

According to the procedure A, **1f- I** was obtained as colorless oil (113.4 mg, yield: 90%). ^{1}H NMR (400 MHz, $CDCl_3$) δ 7.05 (d, *J* = 3.5 Hz, 1H), 6.67 (d, *J* = 3.5 Hz, 1H), 3.89 (s, 3H). ^{13}C NMR (101 MHz, $CDCl_3$) δ 157.9, 149.7, 122.6, 120.1, 94.9, 52.1. HRMS (ESI) calcd for $C_6H_6IO_3^+$ [M + H$^+$] 252.9356, found 252.9357.

5-Bromo-2,4-dimethoxypyrimidine (1g-Br)

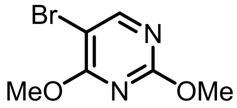

According to the procedure A, **1g-Br** was obtained as a white solid (104.0 mg, yield: 95%). mp: 62-64 °C. ^{1}H NMR (400 MHz, $CDCl_3$) δ 8.25 (s, 1H), 4.00 (s, 3H), 3.93 (s, 3H). ^{13}C NMR (101 MHz, $CDCl_3$) δ 166.8, 164.3, 159.1, 98.1, 55.3, 54.9. HRMS (ESI) calcd for $C_6H_8{}^{79}BrN_2O_2^+$ [M + H$^+$] 218.9764, found 218.9764.

1-Chloro-2-methoxynaphthalene (1h-Cl)

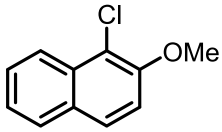

According to the procedure A, **1h-Cl** was obtained as a white solid (83.8 mg, yield: 87%). mp: 66-68 °C. ^{1}H NMR (400 MHz, $CDCl_3$) δ 8.24 (d, *J* = 8.6 Hz, 1H), 7.85 – 7.72 (m, 2H), 7.58 (t, *J* = 7.5 Hz, 1H), 7.41 (t, *J* = 7.4 Hz, 1H), 7.29 (d, *J* = 9.0 Hz, 1H), 4.03 (s, 3H). ^{13}C NMR (101 MHz, $CDCl_3$) δ 152.6, 131.9, 129.6, 128.0, 128.0, 127.5, 124.3, 123.5, 116.9, 113.7, 57.0. HRMS (ESI) calcd for $C_{11}H_{10}{}^{35}ClO^+$ [M + H$^+$] 193.0415, found 193.0416.

***tert*-Butyl (4-chlorophenyl) carbamate (1i-Cl)**

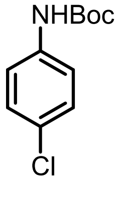

According to the procedure A, **1i-Cl** was gained as a white solid (91.6 mg, yield: 80%). mp: 98-100 °C. ^{1}H NMR (400 MHz, $CDCl_3$) δ 7.57 (d, *J* = 8.7 Hz, 2H), 7.14 (d, *J* = 8.4 Hz, 2H), 6.44 (s, 1H), 1.51 (s, 9H). ^{13}C NMR (101 MHz, $CDCl_3$) δ 152.6, 137.0, 129.0, 128.0, 119.7, 80.9, 28.3. HRMS (ESI) calcd for $C_{11}H_{15}{}^{35}ClNO_2^+$ [M + H$^+$] 228.0786, found 228.0790.

***tert*-Butyl (bromophenyl) carbamate (1i-Br)**

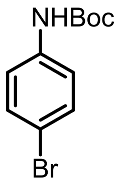

According to the procedure A, **1i-Br** was gained as a white solid (126.5 mg, yield: 93%). mp: 102-104 °C. ^{1}H NMR (400 MHz, $CDCl_3$) δ 7.42 – 7.34 (m, 2H), 7.30 – 7.21 (m, 2H), 6.50 (s, 1H), 1.51 (s, 9H). ^{13}C NMR (101 MHz, $CDCl_3$) δ 152.5, 137.5, 131.9, 120.1, 115.4, 80.9, 28.3. HRMS (ESI) calcd for $C_{11}H_{15}{}^{79}BrNO_2^+$ [M + H^+] 272.0281, found 272.0280.

***tert*-Butyl (4- iodophenyl) carbamate (1i-I)**

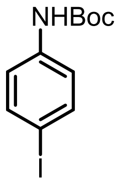

According to the procedure A, **1i-I** was gained as a white solid (132.4 mg, yield: 83%). mp: 142-144 °C. ^{1}H NMR (400 MHz, $CDCl_3$) δ 7.60 – 7.53 (m, 2H), 7.14 (d, J = 8.7 Hz, 2H), 6.56 (s, 1H), 1.50 (s, 9H). ^{13}C NMR (101 MHz, $CDCl_3$) δ 152.5, 138.2, 137.8, 120.5, 85.8, 80.9, 28.3. HRMS (ESI) calcd for $C_{11}H_{15}INO_2^+$ [M + H^+] 320.0142, found 320.0146.

4-(4-Bromophenyl) morpholine (1j-Br)

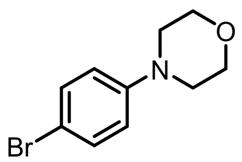

According to the procedure A, **1j-Br** was gained as a white solid (105.4 mg, yield: 87%). mp: 112-114 °C. ^{1}H NMR (400 MHz, $CDCl_3$) δ 7.35 (d, J = 8.8 Hz, 2H), 6.78 (d, J = 8.8 Hz, 1H), 3.95 – 3.79 (m, 2H), 3.22 – 3.06 (m, 2H). ^{13}C NMR (101 MHz, $CDCl_3$) δ 150.3, 132.0, 117.3, 112.2, 66.8, 49.2. HRMS (ESI) calcd for $C_{10}H_{13}{}^{79}BrNO^+$ [M + H^+] 242.0175, found 242.0177.

4-(4- Iodophenyl) morpholine (1j-I)

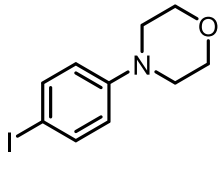

According to the procedure A, **1j-I** was gained as a white solid (107.0 mg, yield: 74%). mp: 143-145 °C. ^{1}H NMR (400 MHz, $CDCl_3$) δ 7.53 (s, 2H), 6.68 (s, 2H), 3.85 (s, 4H), 3.12 (s, 4H). ^{13}C NMR (101 MHz, $CDCl_3$) δ 150.9, 137.9, 117.8, 81.9, 66.7, 48.9. HRMS (ESI) calcd for $C_{10}H_{13}INO^+$ [M + H^+] 290.0036,

found 290.0038.

6-Chloro-2,3-dimethoxybenzaldehyde (1k-Cl)

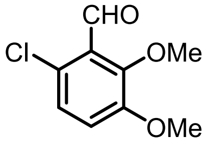

According to the procedure A, **1k-Cl** was obtained as a white solid (82.3 mg, yield: 82%). mp: 74-76 °C. ^{1}H NMR (400 MHz, $CDCl_3$) δ 10.43 (s, 1H), 7.13 (d, J = 8.8 Hz, 1H), 7.02 (d, J = 8.8 Hz, 1H), 3.93 (s, 3H), 3.88 (s, 3H). ^{13}C NMR (101 MHz, $CDCl_3$) δ 189.5, 152.2, 152.1, 127.5, 126.1, 125.7, 117.2, 62.3, 56.3. HRMS (ESI) calcd for $C_9H_{10}{}^{35}ClO_3^+$ [M + H$^+$] 201.0313, found 201.0315.

6- Bromo-2,3-dimethoxybenzaldehyde (1k-Br)

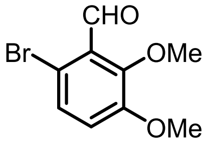

According to the procedure A, **1k-Br** was gained as a white solid (102.9 mg, yield: 84%). mp: 79-81 °C. ^{1}H NMR (400 MHz, $CDCl_3$) δ 10.34 (s, 1H), 7.35 (d, J = 5.7 Hz, 1H), 6.97 (d, J = 6.6 Hz, 1H), 3.93 (s, 3H), 3.89 (s, 3H). ^{13}C NMR (101 MHz, $CDCl_3$) δ 190.5, 152.8, 152.1, 129.4, 128.7, 117.5, 112.8, 62.4, 56.2. HRMS (ESI) calcd for $C_9H_{10}{}^{79}BrO_3^+$ [M + H$^+$] 244.9808, found 244.9810.

6- Iodo-2,3-dimethoxybenzaldehyde (1k-I)

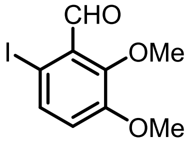

According to the procedure A, **1k-I** was obtained as a white solid (124.1 mg, yield: 85%). mp: 98-100 °C. ^{1}H NMR (400 MHz, $CDCl_3$) δ 10.27 (s, 1H), 7.68 (s, 1H), 7.36 (s, 1H), 3.95 (s, 3H), 3.88 (s, 3H). ^{13}C NMR (101 MHz, $CDCl_3$) δ 188.5, 153.8, 152.6, 130.9, 128.2, 126.7, 87.0, 62.4, 56.4. HRMS (ESI) calcd for $C_9H_{10}IO_3^+$ [M + H$^+$] 292.9669, found 292.9665.

2-(5-Bromo-2-methoxyphenyl) acetic acid (1l-Br)

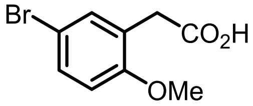

According to the procedure A, **1l-Br** was obtained as a white solid (101.7 mg, yield: 83%). mp: 131-133 °C. ^{1}H NMR (400 MHz, $CDCl_3$) δ 7.37 (dd, J = 8.7, 2.5 Hz, 1H), 7.30 (d, J = 2.4 Hz, 1H), 6.75 (d, J = 8.7

Hz, 1H), 3.80 (s, 3H), 3.63 (s, 2H). ^{13}C NMR (101 MHz, $CDCl_3$) δ 177.6, 156.7, 133.7, 131.5, 124.5, 112.6, 112.2, 55.8, 35.4. HRMS (ESI) calcd for $C_9H_{10}{}^{79}BrO_3^+$ [M + H$^+$] 244.9808, found 244.9812.

2-(5- Iodo-2-methoxyphenyl) acetic acid (1l-I)

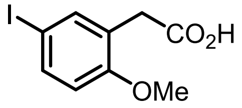

According to the procedure A, **1l-I** was gained as a white solid (115.4 mg, yield: 79%). mp: 158-160 °C. ^{1}H NMR (400 MHz, $CDCl_3$) δ 7.55 (dd, *J* = 8.6, 2.2 Hz, 1H), 7.47 (d, *J* = 2.2 Hz, 1H), 6.65 (d, *J* = 8.6 Hz, 1H), 3.80 (s, 3H), 3.60 (s, 2H). ^{13}C NMR (101 MHz, $CDCl_3$) δ 176.9, 157.5, 139.4, 137.6, 125.0, 112.8, 82.6, 55.7, 35.2. HRMS (ESI) calcd for $C_9H_{10}IO_3^+$ [M + H$^+$] 292.9669, found 292.9671.

1-Chloro-4,5-dimethoxy-2-methylbenzene (1m-Cl)

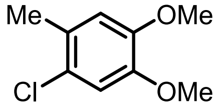

According to the procedure A, **1m-Cl** was obtained as colorless oil (80.3 mg, yield: 86%). ^{1}H NMR (400 MHz, $CDCl_3$) δ 6.82 (s, 1H), 6.68 (s, 1H), 3.82 (d, *J* = 3.5 Hz, 6H), 2.28 (s, 3H). ^{13}C NMR (101 MHz, $CDCl_3$) δ 147.59, 147.57, 127.6, 125.0, 113.6, 112.4, 56.12, 56.05, 19.5. HRMS (ESI) calcd for $C_9H_{12}{}^{35}ClO_2^+$ [M + H$^+$] 187.0520, found 187.0523.

1-Bromo-4,5-dimethoxy-2-methylbenzene (1m-Br)

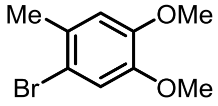

According to the procedure A, **1m-Br** was obtained as a white solid (109.8 mg, yield: 95%). mp: 38--40 °C. ^{1}H NMR (400 MHz, $CDCl_3$) δ 6.97 (s, 1H), 6.70 (s, 1H), 3.82 (d, *J* = 2.1 Hz, 6H), 2.30 (s, 3H). ^{13}C NMR (101 MHz, $CDCl_3$) δ 148.2, 147.7, 129.6, 115.3, 114.4, 113.6, 56.2, 56.0, 22.3. HRMS (ESI) calcd for $C_9H_{12}{}^{79}BrO_2^+$ [M + H$^+$] 231.0015, found 231.0019.

1-Iodo-4,5-dimethoxy-2-methylbenzene (1m-I)

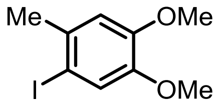

According to the procedure A, **1m-I** was obtained as a white solid (112.6 mg, yield: 81%). mp: 65-67 °C. ^{1}H NMR (400 MHz, $CDCl_3$) δ 7.19 (s, 1H), 6.74 (s, 1H), 3.82 (d, *J* = 2.9 Hz, 6H), 2.35 (s, 3H). ^{13}C NMR (101 MHz, $CDCl_3$) δ 149.2, 147.6, 133.6, 121.4, 112.7, 88.5, 56.2, 55.9, 27.5. HRMS (ESI) calcd for $C_9H_{12}IO_2^+$ [M + H$^+$] 278.9876, found 278.9874.

2-Chloro-1,3,5-triisopropylbenzene (1n-Cl)

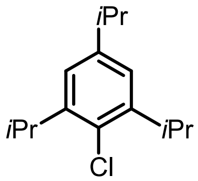

According to the procedure A, **1n-Cl** was obtained as yellow oil (99.1 mg, yield: 83%). ^{1}H NMR (400 MHz, $CDCl_3$) δ 7.04 (s, 2H), 3.60 – 3.44 (m, 2H), 2.98 – 2.85 (m, 1H), 1.29 (dd, J = 6.9, 1.6 Hz, 18H). ^{13}C NMR (101 MHz, $CDCl_3$) δ 147.1, 145.6, 130.1, 122.0, 34.2, 30.7, 24.1, 22.9. HRMS (ESI) calcd for $C_{15}H_{24}{}^{35}Cl^+$ [M + H$^+$] 239.1561, found 239.1565.

2-Bromo-1,3,5-triisopropylbenzene (1n-Br)

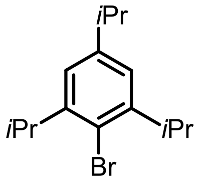

According to the procedure A, **1n-Br** was obtained as colorless oil (128.9 mg, yield: 91%). ^{1}H NMR (400 MHz, $CDCl_3$) δ 7.00 (s, 2H), 3.50 (hept, J = 6.8 Hz, 2H), 2.89 (hept, J = 6.9 Hz, 1H), 1.26 (d, J = 6.9 Hz, 18H). ^{13}C NMR (101 MHz, $CDCl_3$) δ 147.8, 147.4, 123.6, 122.3, 34.1, 33.6, 24.1, 23.1. HRMS (ESI) calcd for $C_{15}H_{24}{}^{79}Br^+$ [M + H$^+$] 283.1056, found 283.1054.

2-Iodo-1,3,5-triisopropylbenzene (1n-I)

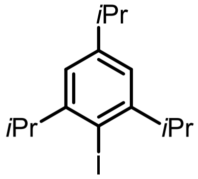

According to the procedure A, **1n-I** was gained as colorless oil (143.7 mg, yield: 87%). ^{1}H NMR (400 MHz, $CDCl_3$) δ 6.98 (s, 2H), 3.43 (m, 2H), 2.90 (m, 1H), 1.27 (dd, J = 6.9, 3.9 Hz, 18H). ^{13}C NMR (101 MHz, $CDCl_3$) δ 150.8, 148.9, 122.1, 105.8, 39.3, 33.9, 24.0, 23.5. HRMS (ESI) calcd for $C_{15}H_{24}I^+$ [M + H$^+$] 331.0917, found 331.0920.

Methyl 5-bromo-2-pivalamidobenzoate (1o-Br)

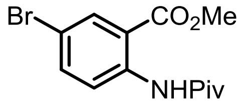

According to the procedure A, **1o-Br** was obtained as a white solid (128.8 mg, yield: 82%). mp: 77-79 °C. ^{1}H NMR (400 MHz, $CDCl_3$) δ 11.22 (s, 1H), 8.69 (d, J = 9.1 Hz, 1H), 8.12 (d, J = 2.5 Hz, 1H), 7.59 (dd,

J = 9.1, 2.5 Hz, 1H), 3.92 (s, 3H), 1.32 (s, 9H). ^{13}C NMR (101 MHz, $CDCl_3$) δ 177.9, 167.7, 141.1, 137.30 133.3, 122.0, 116.5, 114.5, 52.6, 40.4, 27.5. HRMS (ESI) calcd for $C_{13}H_{17}{}^{79}BrNO_3^+$ [M + H^+] 314.0386, found 314.0385.

Methyl 5-iodo-2-pivalamidobenzoate (1o-I)

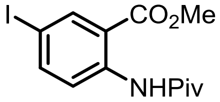

According to the procedure A, **1o-I** was obtained as a white solid (157.1 mg, yield: 87%). mp: 84-86 °C. ^1H NMR (400 MHz, $CDCl_3$) δ 11.22 (s, 1H), 8.56 (d, J = 9.0 Hz, 1H), 8.30 (d, J = 2.2 Hz, 1H), 7.76 (dd, J = 9.0, 2.2 Hz, 1H), 3.91 (s, 3H), 1.32 (s, 9H). ^{13}C NMR (101 MHz, $CDCl_3$) δ 177.9, 167.5, 143.1, 141.7, 139.3 122.3, 116.8, 84.5, 52.6, 40.5, 27.5. HRMS (ESI) calcd for $C_{13}H_{17}INO_3^+$ [M + H^+] 362.0248, found 362.0249.

Methyl 5-bromo-2-methoxybenzoate (1p-Br)

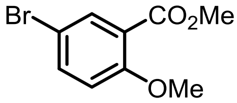

According to the procedure A, **1p-Br** was gained as colorless oil (106.5 mg, yield: 87%). ^1H NMR (400 MHz, $CDCl_3$) δ 7.90 (d, J = 2.6 Hz, 1H), 7.55 (dd, J = 8.9, 2.6 Hz, 1H), 6.86 (d, J = 8.9 Hz, 1H), 3.89 (d, J = 1.2 Hz, 6H). ^{13}C NMR (101 MHz, $CDCl_3$) δ 165.3, 158.3, 136.1, 134.2, 121.7, 113.9, 112.2, 56.3, 52.3. HRMS (ESI) calcd for $C_9H_{10}{}^{79}BrO_3^+$ [M + H^+] 244.9808, found 244.9806.

Methyl 5-iodo-2-methoxybenzoate (1p-I)

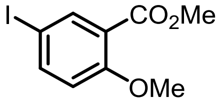

According to the procedure A, **1p-I** was gained as a white solid (127.1 mg, yield: 87%). mp: 54-56 °C. ^1H NMR (400 MHz, $CDCl_3$) δ 8.05 (s, 1H), 7.71 (d, J = 7.2 Hz, 1H), 6.74 (d, J = 7.9 Hz, 1H), 3.86 (s, 6H). ^{13}C NMR (101 MHz, $CDCl_3$) δ 165.2, 159.0, 142.0, 140.0, 122.2, 114.4, 81.7, 56.2, 52.3. HRMS (ESI) calcd for $C_9H_{10}IO_3^+$ [M + H^+] 292.9669, found 292.9672.

1-Bromo-3,5-dichloro-2-methoxybenzene (1q-Br)

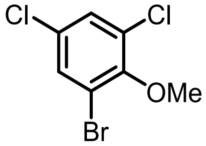

According to the procedure A, **1q-Br** was obtained as yellow oil (121.6 mg, yield: 95%). mp: 59-61 °C.

^1H NMR (400 MHz, $CDCl_3$) δ 7.45 (d, *J* = 2.5 Hz, 1H), 7.33 (d, *J* = 2.5 Hz, 1H), 3.87 (s, 3H). ^{13}C NMR (101 MHz, $CDCl_3$) δ 152.4, 131.6, 130.0, 129.7, 129.5, 118.8, 60.8. HRMS (ESI) calcd for $C_7H_6{}^{79}Br^{35}Cl_2O^+$ [M + H$^+$] 254.8974, found 254.8977.

1,5-Dichloro-3-iodo-2-methoxybenzene (1q-I)

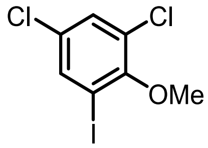

According to the procedure A, **1q-I** was obtained as a white solid (137.8 mg, yield: 91%). mp: 39-41 °C. ^1H NMR (400 MHz, $CDCl_3$) δ 7.34 (d, *J* = 2.5 Hz, 1H), 7.17 (dd, *J* = 8.8, 2.5 Hz, 1H), 6.82 (d, *J* = 8.8 Hz, 1H), 3.87 (s, 3H). ^{13}C NMR (101 MHz, $CDCl_3$) δ 153.9, 129.9, 127.6, 125.6, 123.2, 112.8, 56.3. HRMS (ESI) calcd for $C_7H_6{}^{35}Cl_2IO^+$ [M + H$^+$] 302.8835, found 302.8834.

3-Bromo-2*H*-chromen-2-one (1r-Br)

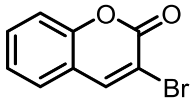

According to the procedure A, **1r-Br** was obtained as a white solid (88.9 mg, yield: 79%). mp: 108-110 °C. ^1H NMR (400 MHz, $CDCl_3$) δ 8.11 (s, 1H), 7.58 (ddd, *J* = 8.6, 7.3, 1.6 Hz, 1H), 7.47 (dd, *J* = 7.8, 1.5 Hz, 1H), 7.36 (d, *J* = 8.4 Hz, 1H), 7.32 (td, *J* = 7.7, 1.1 Hz, 1H). ^{13}C NMR (101 MHz, $CDCl_3$) δ 157.3, 153.4, 144.5, 132.2, 127.3, 125.1, 119.5, 117.0, 112.1. HRMS (ESI) calcd for $C_9H_6{}^{79}BrO_2^+$ [M + H$^+$] 224.9546, found 224.9546.

9,10-Dichloroanthracene (1s-Cl$_2$)

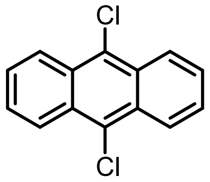

According to the procedure A, **1s-Cl$_2$** was gained as a light-yellow solid (113.7 mg, yield: 92%). mp: 202-204 °C. ^1H NMR (400 MHz, $CDCl_3$) δ 8.54 (dd, *J* = 6.7, 3.1 Hz, 4H), 7.64 (dd, *J* = 6.8, 3.1 Hz, 4H). ^{13}C NMR (101 MHz, $CDCl_3$) δ 129.2, 128.3, 127.1, 125.2. HRMS (ESI) calcd for $C_{14}H_9{}^{35}Cl_2^+$ [M + H$^+$] 247.0076, found 247.0079.

2,8-Dibromodibenzo[*b,d*]furan (1t-Br$_2$)

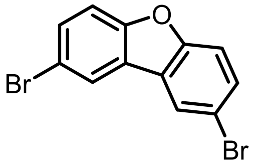

According to the procedure A, **1t-Br$_2$** was gained as a white solid (141.8 mg, yield: 87%). mp: 188-190 °C. ^{1}H NMR (400 MHz, CDCl$_3$) δ 8.02 (d, J = 1.9 Hz, 1H), 7.57 (dd, J = 8.7, 2.0 Hz, 2H), 7.44 (d, J = 8.7 Hz, 2H). ^{13}C NMR (101 MHz, CDCl$_3$) δ 155.3, 130.7, 125.2, 123.7, 115.9, 113.4. HRMS (ESI) calcd for $C_{12}H_7{}^{79}Br_2O^+$ [M + H$^+$] 324.8858, found 324.8854.

2,8-Dibromodibenzo[*b,d*]thiophene (1u-Br$_2$)

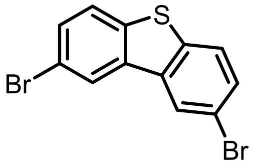

According to the procedure A, **1u-Br$_2$** was gained as a white solid (148.8 mg, yield: 87%). mp: 222-224 °C. ^{1}H NMR (400 MHz, CDCl$_3$) δ 8.22 (d, J = 1.8 Hz, 2H), 7.70 (d, J = 8.5 Hz, 2H), 7.57 (dd, J = 8.5, 1.8 Hz, 2H). ^{13}C NMR (101 MHz, CDCl$_3$) δ 138.6, 136.2, 130.3, 124.7, 124.2, 118.6. HRMS (ESI) calcd for $C_{12}H_7{}^{79}Br_2S^+$ [M + H$^+$] 340.8630, found 340.8631.

6,6'-Dibromo-[1,1'-binaphthalene]-2,2'-diol (1v-Br$_2$)

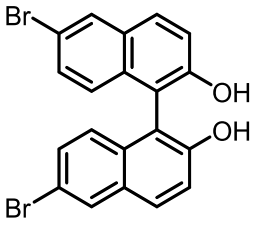

According to the procedure A, **1v-Br$_2$** was obtained as a white solid (204.3 mg, yield: 92%). mp: 204-206 °C. ^{1}H NMR (400 MHz, DMSO) δ 9.51 (s, 2H), 8.12 (s, 2H), 7.86 (d, J = 7.3 Hz, 2H), 7.34 (dd, J = 22.7, 7.5 Hz, 4H), 6.87 (d, J = 7.5 Hz, 2H). ^{13}C NMR (101 MHz, DMSO) δ 154.1, 133.1, 130.1, 129.8, 129.4, 128.7, 127.1, 120.2, 115.7, 115.5. HRMS (ESI) calcd for $C_{20}H_{13}{}^{79}Br_2O_2^+$ [M + H$^+$] 442.9277, found 442.9279.

Examples of arenes bearing strong EWGs failed to achieve halogenation

Although abundant examples, which have relatively low reactivity with solely NXS, could be halogenated via the catalytic system. Disappointingly, when this catalytic halogenation approach was applied to arenes bearing strong electron-withdrawing groups, such as F, CN, NO_2, CF_3, the halogenation was failed to achieve.

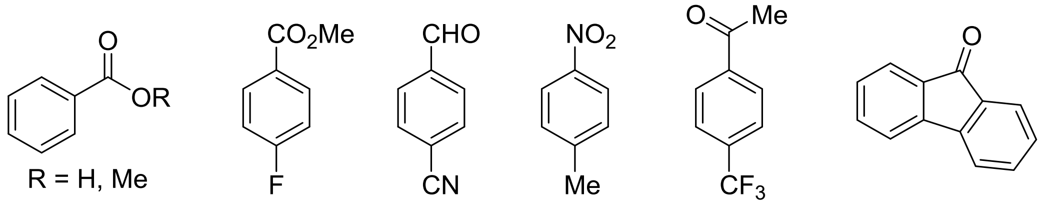

General procedure B for halogenation of aromatics: NIS (0.6 mmol, 1.20 equiv, 135.0 mg) and thianthracene (5.41 mg, 0.025 mmol, 0.05 equiv) were dissolved in DCE (3.0 mL). With stirring of the mixture, trifluoromethanesulfonic acid (TfOH) (3.75 mg, 0.025 mmol, 0.05 equiv) was added. Then cyclohexene (41.07 mg, 0.5 mmol, 1.0 equiv) and carboxylic acid (0.6 mmol, 1.2 equiv) was added sequentially and the mixture was stirred at rt until the reaction was completed. Then the solvent of reaction mixture was removed by rotary evaporation. The residue was purified by silica gel column chromatography to afford the corresponding halogenated products.

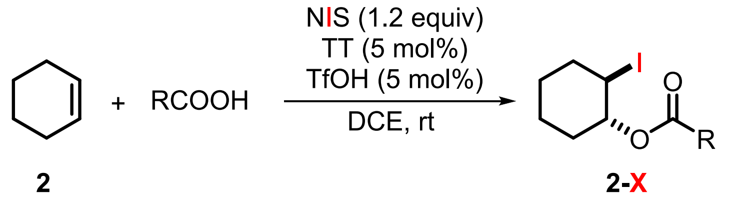

2-Iodocyclohexyl benzoate (2a-I)

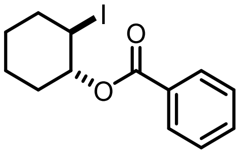

According to the procedure B, **2a-I** was gained as colorless oil (143.6 mg, yield: 87%). ^{1}H NMR (400 MHz, $CDCl_3$) δ 8.12 – 8.05 (m, 2H), 7.61 – 7.54 (m, 1H), 7.50 – 7.42 (m, 2H), 5.13 (m, 1H), 4.26 (m, 1H), 2.55 – 2.44 (m, 1H), 2.29 (m, 1H), 2.10 (m, 1H), 1.93 – 1.83 (m, 1H), 1.63 (m, 1H), 1.53 (m, 2H), 1.45 – 1.33 (m, 1H). ^{13}C NMR (101 MHz, $CDCl_3$) δ 165.5, 133.0, 130.3, 129.79, 128.4, 37.7, 31.5, 31.4, 26.9, 23.5. HRMS (ESI) calcd for $C_{13}H_{16}IO_2^+$ [M + H$^+$] 331.0189, found 331.0191.

2-Iodocyclohexyl 2-(2-methoxyphenyl) acetate (2b-I)

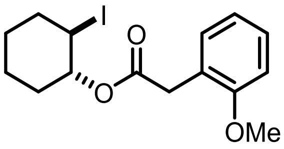

According to the procedure B, **2b-I** was gained as a white solid. (157.2 mg, yield: 84%). mp: 95-97 °C. ^{1}H NMR (400 MHz, $CDCl_3$) δ 7.33 – 7.20 (m, 2H), 7.02 – 6.84 (m, 2H), 4.95 (m, 1H), 4.10 (m, 1H), 3.84 (s, 3H), 3.68 (s, 2H), 2.47 – 2.32 (m, 1H), 2.25 – 2.10 (m, 1H), 2.08 – 1.95 (m, 1H), 1.78 (m, 1H), 1.60 (m,

1H), 1.51 – 1.37 (m, 2H), 1.32 (m, 1H). ^{13}C NMR (101 MHz, $CDCl_3$) δ 170.7, 157.6, 131.0, 128.5, 123.1, 120.5, 110.4, 55.4, 37.4, 36.3, 31.5, 31.0, 26.7, 23.3. HRMS (ESI) calcd for $C_{15}H_{20}IO_3^+$ [M + H$^+$] 375.0452, found375.0450.

2-Iodocyclohexyl benzoate (2c-I)

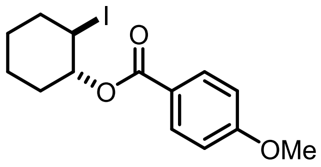

According to the procedure B, **2c-I** was gained as colorless oil (145.3 mg, yield: 88%). ^{1}H NMR (400 MHz, $CDCl_3$) δ 8.03 (d, *J* = 8.7 Hz, 2H), 6.93 (d, *J* = 8.7 Hz, 2H), 5.09 (m, 1H), 4.25 (m, 1H), 3.86 (s, 3H), 2.53 – 2.39 (m, 1H), 2.27 (d, *J* = 9.9 Hz, 1H), 2.15 – 2.01 (m, 1H), 1.86 (m, 1H), 1.67 – 1.59 (m, 1H), 1.57 – 1.45 (m, 2H), 1.43 – 1.32 (m, 1H). ^{13}C NMR (101 MHz, $CDCl_3$) δ 165.2, 163.5, 131.8, 122.7, 113.6, 55.5, 37.7, 31.7, 31.5, 26.9, 23.6. HRMS (ESI) calcd for $C_{14}H_{18}IO_3^+$ [M + H$^+$] 361.0295, found 361.0298.

General procedure C for halogenation of compounds 3 or 4: NXS (0.6 mmol, 1.20 equiv, X = Cl, Br, I) and thianthracene (5.4 mg, 0.025 mmol, 0.05 equiv) were dissolved in DCE (3.0 mL). With stirring of the mixture, trifluoromethanesulfonic acid (TfOH) (3.75 mg, 0.025 mmol, 0.05 equiv) was added. Then substrates **3 or 4** (0.5 mmol, 1.0 equiv) were added to the mixture and stirred at rt. After the reaction was completed, the solvent of reaction mixture was removed by rotary evaporation. The residue was purified by silica gel column chromatography on to afford the corresponding halogenated products.

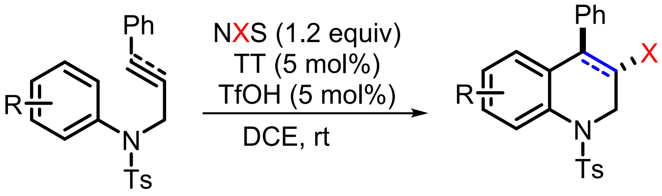

3 or 4 **3-X or 4-X**

3-Chloro-4-phenyl-1-tosyl-1,2,3,4-tetrahydroquinoline (3a-Cl)

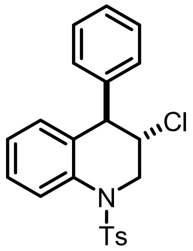

According to the procedure C, **3a-Cl** was obtained as a white solid (119.4 mg, yield: 60%). mp: 156-158 °C. ^{1}H NMR (400 MHz, $CDCl_3$) δ 7.83 (d, *J* = 8.4 Hz, 1H), 7.53 (d, *J* = 8.3 Hz, 2H), 7.23 – 7.11 (m, 4H),

7.07 (t, J = 7.3 Hz, 2H), 6.98 – 6.91 (m, 1H), 6.60 (d, J = 7.8 Hz, 1H), 6.51 – 6.45 (m, 2H), 4.58 (dd, J = 13.9, 3.9 Hz, 1H), 3.92 (d, J = 9.4 Hz, 1H), 3.75 (ddd, J = 11.3, 9.5, 3.9 Hz, 1H), 3.58 (dd, J = 13.9, 11.3 Hz, 1H), 2.37 (s, 3H). ^{13}C NMR (101 MHz, $CDCl_3$) δ 144.4, 141.5, 136.6, 135.8, 131.6, 130.5, 130.1, 128.9, 128.4, 127.5, 127.4, 126.0, 124.7, 57.2, 54.1, 52.1, 21.6. HRMS (ESI) calcd for $C_{22}H_{21}{}^{35}ClNO_2S^+$ [M + H$^+$] 398.0976, found 398.0974.

3-Bromo -4-phenyl-1-tosyl-1,2,3,4-tetrahydroquinoline (3a-Br)

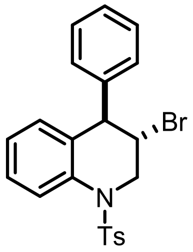

According to the procedure C, **3a-Br** was gained as a white solid (98.4 mg, yield: 89%). mp: 159-161 °C. ^{1}H NMR (400 MHz, $CDCl_3$) δ 7.92 (d, J = 7.3 Hz, 1H), 7.63 (d, J = 6.4 Hz, 2H), 7.48 – 7.11 (m, 6H), 7.05 (s, 1H), 6.69 (d, J = 6.3 Hz, 1H), 6.56 (d, J = 5.3 Hz, 2H), 4.79 (d, J = 12.7 Hz, 1H), 4.19 (d, J = 8.9 Hz, 1H), 3.94 (s, 1H), 3.80 (t, J = 12.5 Hz, 1H), 2.48 (s, 3H). ^{13}C NMR (101 MHz, $CDCl_3$) δ 144.4, 141.8, 136.7, 135.7, 131.9, 130.4, 130.1, 128.8, 128.4, 127.43, 127.37, 127.4, 126.0, 124.9, 54.4, 52.7, 49.3, 21.6. HRMS (ESI) calcd for $C_{22}H_{21}{}^{79}BrNO_2S^+$ [M + H$^+$] 442.0471, found 442.0473.

3-Iodo -4-phenyl-1-tosyl-1,2,3,4-tetrahydroquinoline (3a-I)

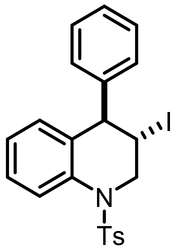

According to the procedure C, **3a-I** was gained as a white solid (102.7 mg, yield: 84%). mp: 176-178 °C. ^{1}H NMR (400 MHz, $CDCl_3$) δ 7.87 (dd, J = 8.3, 1.0 Hz, 1H), 7.60 (d, J = 8.3 Hz, 2H), 7.30 (d, J = 8.0 Hz, 2H), 7.25 – 7.18 (m, 2H), 7.14 (t, J = 7.4 Hz, 2H), 7.03 – 6.96 (m, 1H), 6.62 (d, J = 7.8 Hz, 1H), 6.54 – 6.46 (m, 2H), 4.85 (dd, J = 13.8, 3.4 Hz, 1H), 4.26 (d, J = 10.1 Hz, 1H), 4.05 – 3.96 (m, 1H), 3.89 (dd, J = 13.8, 12.3 Hz, 1H), 2.46 (s, 1H). ^{13}C NMR (101 MHz, $CDCl_3$) δ 144.4, 142.4, 136.8, 135.8, 131.8, 130.4, 130.1, 128.6, 128.4, 127.4, 127.3, 126.0, 125.1, 55.5, 54.9, 28.7, 21.6. HRMS (ESI) calcd for $C_{22}H_{21}INO_2S^+$ [M + H$^+$] 490.0332, found 490.0336.

3-Chloro-6-methyl-4-phenyl-1-tosyl-1,2,3,4-tetrahydroquinoline (3b-Cl)

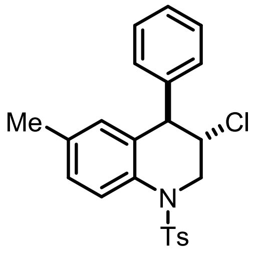

According to the procedure C, **3b-Cl** was gained as a white solid (123.4 mg, yield: 60%). mp: 149-151 °C. ^{1}H NMR (400 MHz, $CDCl_3$) δ 7.78 (d, *J* = 8.4 Hz, 1H), 7.60 (d, *J* = 8.1 Hz, 2H), 7.29 (d, *J* = 8.1 Hz, 2H), 7.21 (d, *J* = 7.2 Hz, 1H), 7.15 (t, *J* = 7.3 Hz, 2H), 7.04 (d, *J* = 8.1 Hz, 1H), 6.53 (d, *J* = 7.3 Hz, 2H), 6.46 (s, 1H), 4.63 (dd, *J* = 13.9, 3.7 Hz, 1H), 3.94 (d, *J* = 9.4 Hz, 1H), 3.83 – 3.73 (m, 1H), 3.69 – 3.55 (m, 1H), 2.45 (s, 3H), 2.15 (s, 3H). ^{13}C NMR (101 MHz, $CDCl_3$) δ 144.3, 141.9, 136.6, 135.9, 133.2, 131.7, 130.6, 130.1, 128.8, 128.4, 128.3, 127.5, 127.4, 124.9, 54.4, 52.8, 49.6, 21.6, 20.9. HRMS (ESI) calcd for $C_{23}H_{23}{}^{35}ClNO_2S^+$ [M + H$^+$] 412.1133, found 412.1137.

3-Bromo-6-methyl-4-phenyl-1-tosyl-1,2,3,4-tetrahydroquinoline (3b-Br)

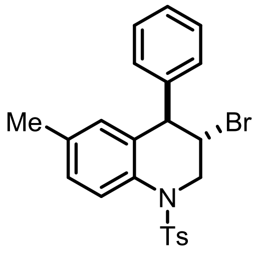

According to the procedure C, **3b-Br** was gained as a white solid (184.6 mg, yield: 81%). mp: 160-162 °C. ^{1}H NMR (400 MHz, $CDCl_3$) δ 7.76 (s, 1H), 7.60 (s, 2H), 7.30 (s, 2H), 7.21 (s, 1H), 7.14 (s, 2H), 7.04 (s, 1H), 6.51 (s, 2H), 6.44 (s, 1H), 4.73 (d, *J* = 10.6 Hz, 1H), 4.09 (s, 1H), 3.86 (s, 1H), 3.73 (s, 1H), 2.45 (s, 3H), 2.14 (s, 3H). ^{13}C NMR (101 MHz, $CDCl_3$) δ 144.3 141.6, 136.5, 135.9, 133.2, 131.5, 130.7, 130.6, 130.1, 130.0, 128.9, 128.9, 128.41, 128.36, 127.8, 127.5, 127.4, 127.3, 124.7, 57.4, 54.1, 52.2, 21.6, 20.9. HRMS (ESI) calcd for $C_{23}H_{23}{}^{79}BrNO_2S^+$ [M + H$^+$] 456.0627, found 456.0628.

3-Iodo-6-methyl-4-phenyl-1-tosyl-1,2,3,4-tetrahydroquinoline (3b-I)

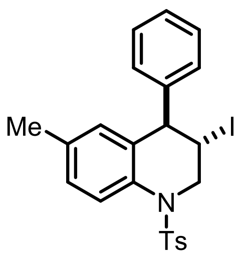

According to the procedure C, **3b-I** was gained as a white solid (98.2 mg, yield: 78%). mp: 186-188 °C. ^{1}H NMR (400 MHz, $CDCl_3$) δ 7.74 (d, *J* = 8.4 Hz, 1H), 7.60 (d, *J* = 8.2 Hz, 2H), 7.31 (d, *J* = 8.1 Hz, 2H), 7.22 (t, *J* = 7.3 Hz, 1H), 7.14 (t, *J* = 7.5 Hz, 2H), 7.03 (d, *J* = 8.2 Hz, 1H), 6.49 (d, *J* = 7.3 Hz, 2H), 6.41 (s,

1H), 4.82 (dd, J = 13.8, 3.4 Hz, 1H), 4.21 (d, J = 10.1 Hz, 1H), 4.02 – 3.92 (m, 1H), 3.91 – 3.80 (m, 1H), 2.47 (s, 3H), 2.14 (s, 3H). ^{13}C NMR (101 MHz, $CDCl_3$) δ 144.2, 142.5, 136.9, 135.8, 133.2, 131.6, 130.6, 130.1, 128.6, 128.4, 128.2, 127.4, 127.3, 125.1, 55.5, 54.9, 29.1, 21.6, 20.8. HRMS (ESI) calcd for $C_{23}H_{23}INO_2S^+$ [M + H^+] 504.0489, found 504.0486.

3-Chloro-6-methoxy-4-phenyl-1-tosyl-1,2,3,4-tetrahydroquinoline (3c-Br)

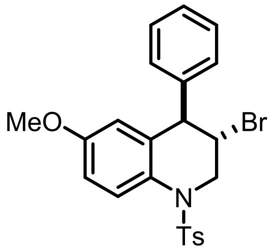

According to the procedure C, **3c-Br** was gained as a white solid (101.6 mg, yield: 86%). mp: 147-149 °C. ^1H NMR (400 MHz, $CDCl_3$) δ 7.81 (d, J = 9.1 Hz, 1H), 7.57 (d, J = 8.2 Hz, 2H), 7.30 (d, J = 8.1 Hz, 2H), 7.20 (t, J = 7.3 Hz, 1H), 7.13 (t, J = 7.4 Hz, 2H), 6.80 (dd, J = 9.1, 2.7 Hz, 1H), 6.47 (d, J = 7.3 Hz, 2H), 6.12 (d, J = 2.5 Hz, 1H), 4.76 (dd, J = 13.9, 3.7 Hz, 1H), 4.07 (d, J = 9.9 Hz, 1H), 3.90 – 3.80 (m, 1H), 3.78 – 3.69 (m, 1H), 3.62 (s, 3H), 2.46 (s, 3H). ^{13}C NMR (101 MHz, $CDCl_3$) δ 157.5, 144.3, 141.4, 136.6, 133.7, 130.1, 128.8, 128.7, 128.4, 127.5, 127.4, 126.7, 115.0, 113.2, 55.3, 54.6, 53.0, 49.2, 21.6. HRMS (ESI) calcd for $C_{23}H_{23}{}^{79}BrNO_3S^+$ [M + H^+] 472.0577, found472.0579.

3-Iodo-6-methoxy-4-phenyl-1-tosyl-1,2,3,4-tetrahydroquinoline (3c-I)

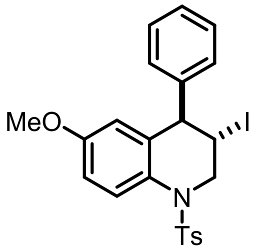

According to the procedure C, **3c-I** was obtained as a white solid (220.8 mg, yield: 85%). mp: 136-138 °C. ^1H NMR (400 MHz, $CDCl_3$) δ 7.79 (d, J = 9.1 Hz, 1H), 7.56 (d, J = 8.1 Hz, 2H), 7.31 (d, J = 8.1 Hz, 2H), 7.20 (t, J = 7.3 Hz, 1H), 7.11 (t, J = 7.5 Hz, 2H), 6.79 (dd, J = 9.1, 2.9 Hz, 1H), 6.43 (d, J = 7.7 Hz, 2H), 6.09 (d, J = 2.8 Hz, 1H), 4.85 (dd, J = 13.6, 3.0 Hz, 1H), 4.17 (d, J = 10.0 Hz, 1H), 3.98 – 3.89 (m, 1H), 3.89 – 3.81 (m, 1H), 3.61 (s, 3H), 2.47 (s, 3H). ^{13}C NMR (101 MHz, $CDCl_3$) δ 157.4, 144.3, 142.0, 136.7, 133.5, 130.2, 128.8, 128.6, 128.4, 127.5, 127.4, 126.9, 115.0, 113.1, 55.6, 55.3, 55.1, 28.8, 21.6. HRMS (ESI) calcd for $C_{23}H_{23}INO_3S^+$ [M + H^+] 520.0438, found 520.0441.

6-(*tert*-Butyl)-3-chloro-4-phenyl-1-tosyl-1,2,3,4-tetrahydroquinoline (3d-Cl)

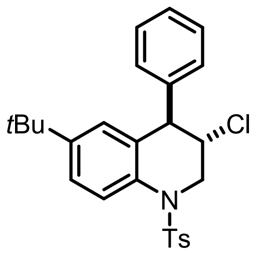

According to the procedure C, **3d-Cl** was obtained as yellow oil (154.4 mg, yield: 68%). ^{1}H NMR (400 MHz, $CDCl_3$) δ 7.82 (d, *J* = 8.7 Hz, 1H), 7.64 (d, *J* = 8.3 Hz, 2H), 7.31 (d, *J* = 8.3 Hz, 2H), 7.30 – 7.22 (m, 2H), 7.19 (dd, *J* = 11.3, 4.5 Hz, 2H), 6.69 (d, *J* = 1.6 Hz, 1H), 6.66 – 6.58 (m, 2H), 4.63 (dd, *J* = 13.9, 3.8 Hz, 1H), 4.04 (d, *J* = 9.3 Hz, 1H), 3.91 – 3.78 (m, 1H), 3.69 (dd, *J* = 13.8, 11.1 Hz, 1H), 2.47 (s, 3H), 1.16 (s, 9H). ^{13}C NMR (101 MHz, $CDCl_3$) δ 148.8, 144.3, 141.7, 136.6, 133.2, 130.8, 130.0, 128.9, 128.4, 127.4, 127.3, 127.3, 124.6, 124.0, 57.5, 54.2, 52.0, 34.3, 31.1, 21.6. HRMS (ESI) calcd for $C_{26}H_{29}{}^{35}ClNO_2S^+$ [M + H$^+$] 454.1602, found 454.1604.

6-(*tert*-Butyl)-3-bromo-4-phenyl-1-tosyl-1,2,3,4-tetrahydroquinoline (3d-Br)

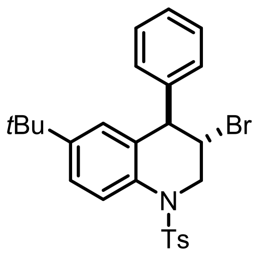

According to the procedure C, **3d-Br** was gained as a white solid. (111.0 mg, yield: 89%). mp: 72-74 ^{o}C. ^{1}H NMR (400 MHz, $CDCl_3$) δ 7.79 (d, *J* = 8.7 Hz, 1H), 7.64 (d, *J* = 8.2 Hz, 2H), 7.35 – 7.27 (m, 3H), 7.23 (d, *J* = 7.2 Hz, 1H), 7.18 (t, *J* = 7.2 Hz, 2H), 6.66 (d, *J* = 1.6 Hz, 1H), 6.61 (d, *J* = 7.1 Hz, 2H), 4.72 (dd, *J* = 13.9, 3.7 Hz, 1H), 4.19 (d, *J* = 9.5 Hz, 1H), 3.97 – 3.88 (m, 1H), 3.79 (dd, *J* = 13.8, 11.6 Hz, 1H), 2.48 (s, 3H), 1.15 (s, 9H). ^{13}C NMR (101 MHz, $CDCl_3$) δ 148.9, 144.2, 142.0, 136.8, 133.2, 131.1, 130.0, 128.8, 128.3, 127.4, 127.3, 127.2, 124.5, 124.2, 54.5, 52.6, 49.7, 34.3, 31.1, 21.6. HRMS (ESI) calcd for $C_{26}H_{29}{}^{79}BrNO_2S^+$ [M + H$^+$] 498.1097, found 498.1099.

6-(*tert*-Butyl)-3-Iodo-4-phenyl-1-tosyl-1,2,3,4-tetrahydroquinoline (3d-I)

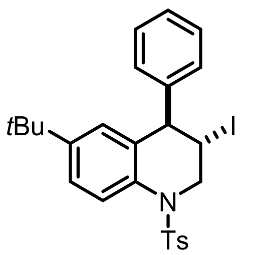

According to the procedure C, **3d-I** was gained as a white solid (114.6 mg, yield: 84%). mp: 105-107 °C.

^{1}H NMR (400 MHz, $CDCl_3$) δ 7.74 (d, *J* = 8.7 Hz, 1H), 7.61 (d, *J* = 8.2 Hz, 2H), 7.30 (d, *J* = 8.1 Hz, 2H), 7.26 – 7.18 (m, 2H), 7.14 (t, *J* = 7.3 Hz, 2H), 4.79 (dd, *J* = 13.8, 3.4 Hz, 1H), 4.27 (d, *J* = 10.0 Hz, 1H), 4.03 – 3.94 (m, 1H), 3.94 – 3.83 (m, 1H), 2.46 (s, 3H), 1.11 (s, 9H). ^{13}C NMR (101 MHz, $CDCl_3$) δ 148.8, 144.2, 142.6, 136.9, 133.2, 131.1, 130.1, 128.6, 128.3, 127.4, 127.3, 127.1, 124.5, 124.4, 55.6, 54.8, 34.3, 31.1, 29.3, 21.6. HRMS (ESI) calcd for $C_{26}H_{29}INO_2S^+$ [M + H^+] 546.0958, found 546.0955.

7-Chloro-8-phenyl-5-tosyl-5,6,7,8-tetrahydro-[1,3]dioxolo[4,5-*g*]quinoline (3e-Cl)

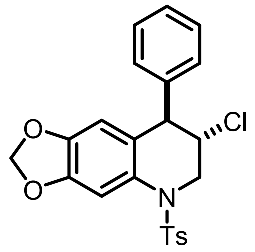

According to the procedure C, **3e-Cl** was gained as a white solid (63.0 mg, yield: 57%). mp: 212-214 °C. ^{1}H NMR (400 MHz, $CDCl_3$) δ 7.62 (d, *J* = 7.9 Hz, 2H), 7.42 (s, 1H), 7.32 (d, *J* = 7.8 Hz, 2H), 7.24 – 7.17 (m, 1H), 7.13 (t, *J* = 7.2 Hz, 2H), 6.49 (d, *J* = 7.1 Hz, 2H), 6.06 (s, 1H), 5.93 (d, *J* = 10.2 Hz, 2H), 4.65 (dd, *J* = 13.8, 3.4 Hz, 1H), 3.85 (d, *J* = 9.6 Hz, 1H), 3.79 – 3.67 (m, 1H), 3.64 – 3.51 (m, 1H), 2.47 (s, 3H). ^{13}C NMR (101 MHz, $CDCl_3$) δ 146.8, 146.1, 144.4, 141.3, 136.3, 130.1, 129.6, 128.8, 128.4, 127.6, 127.4, 125.7, 108.8, 106.0, 101.6, 57.1, 54.3, 52.5, 21.6. HRMS (ESI) calcd for $C_{23}H_{21}{}^{35}ClNO_4S^+$ [M + H^+] 442.0874, found 442.0872.

7-Bromo-8-phenyl-5-tosyl-5,6,7,8-tetrahydro- [1,3] dioxolo[4,5-*g*] quinoline (3e-Br)

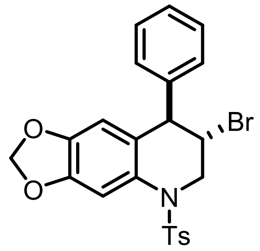

According to the procedure C, **3e-Br** was gained as a white solid (97.3 mg, yield: 80%). mp: 182-184 °C (dec). ^{1}H NMR (400 MHz, $CDCl_3$) δ 7.63 (d, *J* = 8.2 Hz, 2H), 7.40 (s, 1H), 7.33 (d, *J* = 8.0 Hz, 2H), 7.20 (t, *J* = 7.3 Hz, 1H), 7.12 (t, *J* = 7.4 Hz, 2H), 6.47 (d, *J* = 7.3 Hz, 2H), 6.03 (s, 1H), 5.92 (d, *J* = 8.8 Hz, 2H), 4.74 (dd, *J* = 13.8, 3.7 Hz, 1H), 4.01 (d, *J* = 9.8 Hz, 1H), 3.85 – 3.77 (m, 1H), 3.74 – 3.64 (m, 1H), 2.47 (s, 3H). ^{13}C NMR (101 MHz, $CDCl_3$) δ 146.7, 146.1, 144.4, 141.6, 136.4, 130.2, 129.5, 128.7, 128.4, 127.6, 127.4, 125.8, 108.8, 106.2, 101.6, 54.5, 53.1, 49.2, 21.6. HRMS (ESI) calcd for $C_{23}H_{21}{}^{79}BrNO_4S^+$ [M + H^+] 486.0369, found 486.0366.

7-Iodo-8-phenyl-5-tosyl-5,6,7,8-tetrahydro- [1,3] dioxolo[4,5-*g*] quinoline (3e-I)

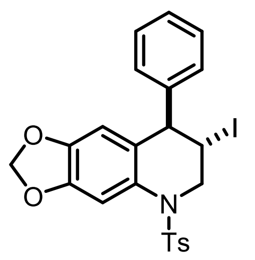

According to the procedure C, **3e-I** was gained as a white solid (116.1 mg, yield: 87%). mp: 162-164 °C (dec). ^{1}H NMR (400 MHz, $CDCl_3$) δ 7.62 (d, J = 8.2 Hz, 2H), 7.37 (s, 1H), 7.33 (d, J = 8.1 Hz, 2H), 7.20 (t, J = 7.3 Hz, 1H), 7.12 (t, J = 7.5 Hz, 2H), 6.46 (t, J = 14.1 Hz, 2H), 6.01 (s, 1H), 5.91 (d, J = 7.4 Hz, 2H), 4.83 (dd, J = 13.5, 3.0 Hz, 1H), 4.12 (d, J = 9.8 Hz, 1H), 3.95 – 3.87 (m, 1H), 3.86 – 3.78 (m, 1H), 2.48 (s, 3H). ^{13}C NMR (101 MHz, $CDCl_3$) δ 146.7, 146.0, 144.4, 142.2, 136.6, 130.2, 129.6, 128.5, 128.4, 127.5, 127.4, 125.6, 108.9, 106.3, 101.6, 55.6, 55.2, 28.7, 21.6. HRMS (ESI) calcd for $C_{23}H_{21}INO_4S^+$ [M + H^+] 534.0230, found 534.0233.

3-Chloro-6-iodo-4-phenyl-1-tosyl-1,2,3,4-tetrahydroquinoline (3f-Cl)

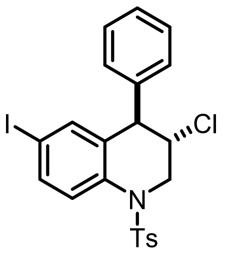

According to the procedure C, **3f-Cl** was obtained as a white solid (149.3 mg, yield: 57%). mp: 140-142 °C. ^{1}H NMR (400 MHz, $CDCl_3$) δ 7.64 (dd, J = 19.5, 8.1 Hz, 3H), 7.53 (d, J = 8.1 Hz, 1H), 7.32 (d, J = 7.4 Hz, 2H), 7.22 (s, 1H), 7.18 (d, J = 7.0 Hz, 2H), 7.00 (s, 1H), 6.54 (d, J = 6.8 Hz, 2H), 4.57 (d, J = 11.9 Hz, 1H), 3.95 (d, J = 8.7 Hz, 1H), 3.76 (d, J = 8.1 Hz, 1H), 3.70 – 3.56 (m, 1H), 2.46 (s, 3H). ^{13}C NMR (101 MHz, $CDCl_3$) δ 144.7, 140.7, 139.1, 136.5, 136.2, 135.8, 133.5, 130.2, 128.8, 128.7, 127.7, 127.4, 126.2, 90.3, 56.6, 53.6, 51.6, 21.6. HRMS (ESI) calcd for $C_{22}H_{20}{}^{35}ClINO_2S^+$ [M + H^+] 523.9942, found 523.9946.

3-Bromo-6-iodo-4-phenyl-1-tosyl-1,2,3,4-tetrahydroquinoline (3f- Br)

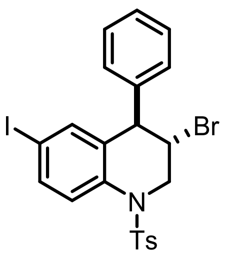

According to the procedure C, **3f-Br** was obtained as a white solid (244.4 mg, yield: 86%). mp: 151-153

°C. ^{1}H NMR (400 MHz, $CDCl_3$) δ 7.54 (t, *J* = 8.2 Hz, 3H), 7.45 (d, *J* = 8.8 Hz, 1H), 7.27 (d, *J* = 7.9 Hz, 2H), 7.15 (d, *J* = 7.2 Hz, 1H), 7.08 (t, *J* = 7.4 Hz, 2H), 6.86 (s, 1H), 6.40 (d, *J* = 7.4 Hz, 2H), 4.71 (d, *J* = 11.7 Hz, 1H), 4.12 (d, *J* = 9.2 Hz, 1H), 3.89 – 3.73 (m, 2H), 2.41 (s, 3H). ^{13}C NMR (101 MHz, $CDCl_3$) δ 144.7, 141.5, 139.0, 136.4, 135.8, 133.7, 130.3, 128.6, 128.5, 127.7, 127.4, 126.9, 100.0, 90.5, 55.0, 54.5, 27.5, 21.7. HRMS (ESI) calcd for $C_{22}H_{20}{}^{79}BrINO_2S^+$ [M + H^+] 567.9437, found 567.9439.

3,6-Diiodo-4-phenyl-1-tosyl-1,2,3,4-tetrahydroquinoline (3f-I)

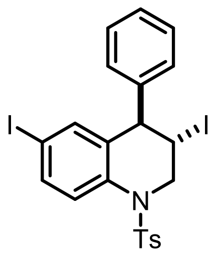

According to the procedure C, **3f-I** was gained as a white solid (115.1 mg, yield: 81%). mp: 138-140 °C. ^{1}H NMR (400 MHz, $CDCl_3$) δ 7.62 (dd, *J* = 8.3, 5.4 Hz, 3H), 7.52 (d, *J* = 8.7 Hz, 1H), 7.34 (d, *J* = 8.0 Hz, 2H), 7.24 (dd, *J* = 12.5, 5.1 Hz, 1H), 7.15 (t, *J* = 7.4 Hz, 2H), 6.95 (s, 1H), 6.49 (d, *J* = 7.3 Hz, 2H), 4.78 (dd, *J* = 13.3, 2.6 Hz, 1H), 4.20 (d, *J* = 9.5 Hz, 1H), 3.97 – 3.89 (m, 1H), 3.89 – 3.80 (m, 1H), 2.48 (s, 3H). ^{13}C NMR (101 MHz, $CDCl_3$) δ 144.7, 141.5, 139.0, 136.5, 136.4, 135.8, 133.7, 130.3, 128.6, 128.5, 127.7, 127.4, 126.8, 90.5, 55.0, 54.5, 27.5, 21.7. HRMS (ESI) calcd for $C_{22}H_{20}I_2NO_2S^+$ [M + H^+] 615.9299, found 615.9302.

3-Chloro-6-fluoro-4-phenyl-1-tosyl-1,2,3,4-tetrahydroquinoline (3g-Cl)

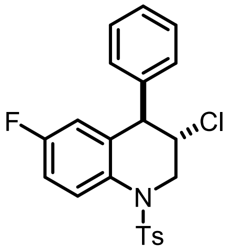

According to the procedure C, **3g-Cl** was gained as a white solid (67.5 mg, yield: 65%). mp: 178-180 °C. ^{1}H NMR (400 MHz, $CDCl_3$) δ 7.89 (dd, *J* = 9.1, 5.2 Hz, 1H), 7.58 (d, *J* = 8.1 Hz, 2H), 7.31 (d, *J* = 8.0 Hz, 2H), 7.22 (d, *J* = 7.5 Hz, 1H), 7.16 (t, *J* = 7.4 Hz, 2H), 6.96 (dd, *J* = 12.0, 4.9 Hz, 1H), 6.50 (d, *J* = 7.4 Hz, 2H), 6.41 – 6.32 (m, 1H), 4.67 (dd, *J* = 14.0, 3.8 Hz, 1H), 3.92 (d, *J* = 9.6 Hz, 1H), 3.81 – 3.72 (m, 1H), 3.68 – 3.57 (m, 1H), 2.46 (s, 3H). ^{19}F NMR (376 MHz, $CDCl_3$) δ -115.27. ^{13}C NMR (101 MHz, $CDCl_3$) δ 160.5 (d, *J* = 246.4 Hz), 144.6, 140.6, 136.3, 134.2 (d, *J* = 7.3 Hz), 131.8 (d, *J* = 2.5 Hz), 130.2, 128.8, 128.6, 127.7, 127.5, 126.9 (d, *J* = 8.3 Hz), 116.5 (d, *J* = 23.3 Hz), 114.8 (d, *J* = 22.6 Hz), 56.6, 54.2, 52.2, 21.6. HRMS (ESI) calcd for $C_{22}H_{20}{}^{35}ClFNO_2S^+$ [M + H^+] 416.0882, found 416.0885.

3-Bromo-6-fluoro-4-phenyl-1-tosyl-1,2,3,4-tetrahydroquinoline (3g-Br)

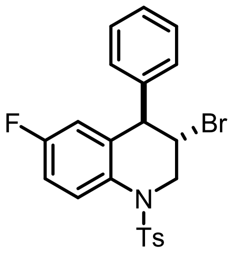

According to the procedure C, **3g-Br** was gained as a white solid (94.3 mg, yield: 82%). mp: 177-179 °C. ^{1}H NMR (400 MHz, $CDCl_3$) δ 7.87 (dd, J = 8.8, 5.2 Hz, 1H), 7.58 (d, J = 7.7 Hz, 2H), 7.32 (d, J = 7.6 Hz, 2H), 7.22 (d, J = 7.1 Hz, 1H), 7.15 (t, J = 7.0 Hz, 2H), 6.95 (t, J = 6.8 Hz, 1H), 6.48 (d, J = 7.1 Hz, 2H), 6.34 (d, J = 8.3 Hz, 1H), 4.88 – 4.68 (m, 1H), 4.08 (d, J = 9.6 Hz, 1H), 3.85 (t, J = 9.1 Hz, 1H), 3.79 – 3.65 (m, 1H). ^{19}F NMR (376 MHz, $CDCl_3$) δ -115.14. ^{13}C NMR (101 MHz, $CDCl_3$) δ 160.5 (d, J = 246.7 Hz), 144.6, 140.9, 136.4, 134.3 (d, J = 7.3 Hz), 131.7 (d, J = 2.5 Hz), 130.2, 128.7, 128.6, 127.7, 127.5, 127.1 (d, J = 8.3 Hz), 116.5 (d, J = 23.2 Hz), 114.8 (d, J = 22.7 Hz), 54.5, 52.9, 48.4, 21.6. HRMS (ESI) calcd for $C_{22}H_{20}{}^{79}BrFNO_2S^+$ [M + H$^+$] 460.0377, found 460.0378.

6-Fluoro-3-iodo-4-phenyl-1-tosyl-1,2,3,4-tetrahydroquinoline (3g-I)

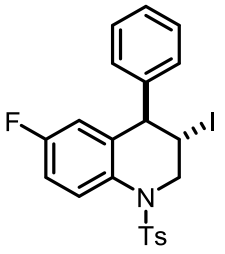

According to the procedure C, **3g-I** was obtained as a white solid (213.0 mg, yield: 84%). mp: 179-181 °C. ^{1}H NMR (400 MHz, $CDCl_3$) δ 7.85 (dd, J = 9.2, 5.3 Hz, 1H), 7.57 (d, J = 8.3 Hz, 2H), 7.33 (d, J = 8.0 Hz, 2H), 7.25 – 7.19 (m, 1H), 7.14 (t, J = 7.5 Hz, 2H), 6.44 (d, J = 7.1 Hz, 2H), 6.34 – 6.25 (m, 1H), 4.90 – 4.81 (m, 1H), 4.18 (d, J = 9.8 Hz, 1H), 3.98 – 3.90 (m, 1H), 3.86 (t, J = 12.6 Hz, 1H), 2.48 (s, 3H). ^{19}F NMR (376 MHz, $CDCl_3$) δ -115.08. ^{13}C NMR (101 MHz, $CDCl_3$) δ 160.4 (d, J = 246.5 Hz), 144.6, 141.5, 136.5, 134.1 (d, J = 7.3 Hz), 131.8 (d, J = 2.8 Hz), 130.3, 128.6, 128.5, 127.7, 127.44, 127.35, 116.4 (d, J = 23.2 Hz), 114.8 (d, J = 22.7 Hz), 55.6, 55.0, 27.6, 21.6. HRMS (ESI) calcd for $C_{22}H_{20}FINO_2S^+$ [M + H$^+$] 508.0238, found 508.0235.

3-Chloro-6-nitro-4-phenyl-1-tosyl-1,2,3,4-tetrahydroquinoline (3h-Cl)

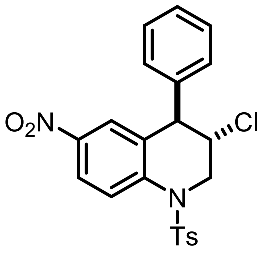

According to the procedure C, **3h-Cl** was obtained as yellow oil (121.7 mg, yield: 55%). mp: 141-143 °C. ^{1}H NMR (400 MHz, $CDCl_3$) δ 8.08 (s, 2H), 7.70 (d, *J* = 7.6 Hz, 3H), 7.34 (d, *J* = 7.1 Hz, 2H), 7.22 (s, 3H), 6.66 (d, *J* = 6.0 Hz, 2H), 4.46 (d, *J* = 13.3 Hz, 1H), 4.17 (d, *J* = 6.6 Hz, 1H), 4.01 (s, 1H), 3.95 – 3.81 (m, 1H), 2.46 (s, 3H). ^{13}C NMR (101 MHz, $CDCl_3$) δ 145.3, 144.3, 141.7, 140.2, 135.8, 130.3, 129.0, 128.6, 128.1, 127.4, 126.4, 123.0, 122.9, 56.1, 53.5, 50.7, 21.7. HRMS (ESI) calcd for $C_{22}H_{20}{}^{35}ClN_2O_4S^+$ [M + H^+] 443.0827, found 443.0827.

3-Bromo-6-nitro-4-phenyl-1-tosyl-1,2,3,4-tetrahydroquinoline (3h-Br)

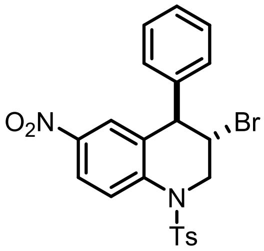

According to the procedure C, **3h-Br** was gained as a white solid (97.5 mg, yield: 80%). mp: 143-145 °C. ^{1}H NMR (400 MHz, $CDCl_3$) δ 8.08 (s, 2H), 7.70 (d, *J* = 8.0 Hz, 2H), 7.64 (s, 1H), 7.35 (d, *J* = 7.8 Hz, 2H), 7.27 (s, 1H), 7.21 (t, *J* = 7.3 Hz, 2H), 6.64 (d, *J* = 7.3 Hz, 2H), 4.59 (d, *J* = 11.2 Hz, 1H), 4.29 (d, *J* = 8.1 Hz, 1H), 4.04 (d, *J* = 7.3 Hz, 1H), 3.99 – 3.90 (m, 1H), 2.47 (s, 3H). ^{13}C NMR (101 MHz, $CDCl_3$) δ 145.3, 144.4, 141.6, 140.4, 135.9, 130.9, 130.4, 129.0, 128.6, 128.1, 127.4, 126.2, 123.4, 122.8, 53.9, 51.5, 47.3, 21.7. HRMS (ESI) calcd for $C_{22}H_{20}{}^{79}BrN_2O_4S^+$ [M + H^+] 487.0322, found 487.0320.

3-Iodo-6-nitro-4-phenyl-1-tosyl-1,2,3,4-tetrahydroquinoline (3h-I)

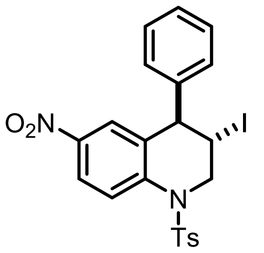

According to the procedure C, **3h-I** was gained as a yellow solid (114.9 mg, yield: 86%). mp: 139-141 °C. ^{1}H NMR (400 MHz, $CDCl_3$) δ 8.06 (s, 2H), 7.67 (d, *J* = 7.4 Hz, 2H), 7.58 (s, 1H), 7.36 (d, *J* = 7.3 Hz, 2H), 7.29 – 7.14 (m, 3H), 6.60 (d, *J* = 6.7 Hz, 2H), 4.69 (d, *J* = 13.1 Hz, 1H), 4.35 (d, *J* = 8.5 Hz, 1H), 4.07

(d, J = 8.6 Hz, 1H), 4.02 – 3.90 (m, 1H), 2.47 (s, 3H). ^{13}C NMR (101 MHz, CDCl$_3$) δ 145.3, 144.5, 141.6, 141.0, 136.1, 131.3, 130.4, 129.0, 128.4, 128.1, 127.3, 126.1, 124.0, 122.7, 55.2, 53.8, 25.6, 21.7. HRMS (ESI) calcd for $C_{22}H_{20}IN_2O_4S^+$ [M + H$^+$] 535.0186, found 535.0191.

3-Bromo-4-phenyl-1-tosyl-1,2-dihydroquinoline (4a-Br)

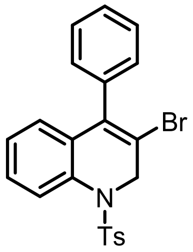

According to the procedure C, **4a-Br** was gained as a white solid (90.3 mg, yield: 82%). mp: 170-172 °C. ^{1}H NMR (400 MHz, CDCl$_3$) δ 7.79 (d, J = 7.9 Hz, 1H), 7.41 (d, J = 7.9 Hz, 2H), 7.37 – 7.26 (m, 4H), 7.17 (d, J = 7.8 Hz, 2H), 7.09 (t, J = 7.5 Hz, 1H), 6.54 (d, J = 7.7 Hz, 1H), 6.48 (d, J = 6.5 Hz, 2H), 4.83 (s, 2H), 2.40 (s, 3H). ^{13}C NMR (101 MHz, CDCl$_3$) δ 144.0, 137.1, 136.7, 135.5, 133.9, 131.5, 129.4, 129.4, 128.3, 128.1, 128.0, 127.5, 127.5, 127.1, 126.5, 115.1, 53.2, 21.5. HRMS (ESI) calcd for $C_{22}H_{19}{}^{79}BrNO_2S^+$ [M + H$^+$] 440.0314, found 440.0316.

3-Iodo-4-phenyl-1-tosyl-1,2-dihydroquinoline (4a-I)

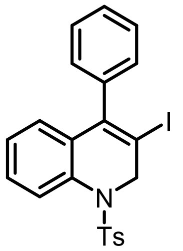

According to the procedure C, **4a-I** was obtained as a white solid (187.6 mg, yield: 77%). mp: 173-175 °C (dec). ^{1}H NMR (400 MHz, CDCl$_3$) δ 7.80 (dd, J = 8.0, 0.8 Hz, 1H), 7.45 (d, J = 8.3 Hz, 2H), 7.39 – 7.28 (m, 4H), 7.21 (d, J = 8.3 Hz, 2H), 7.10 (td, J = 7.7, 1.1 Hz, 1H), 6.56 (dd, J = 7.8, 1.3 Hz, 1H), 6.49 – 6.40 (m, 2H), 4.90 (s, 2H), 2.44 (s, 3H). ^{13}C NMR (101 MHz, CDCl$_3$) δ 143.9, 143.1, 140.1, 135.7, 134.2, 130.9, 129.5, 129.2, 128.5, 128.2, 128.1, 127.6, 127.4, 127.0, 126.7, 92.7, 56.9, 21.5. HRMS (ESI) calcd for $C_{22}H_{19}INO_2S^+$ [M + H$^+$] 488.0176, found 488.0177.

3-Bromo-6-fluoro-4-phenyl-1-tosyl-1,2-dihydroquinoline (4b-Br)

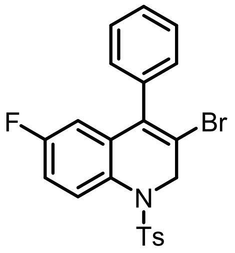

According to the procedure C, **4b-Br** was obtained as a white solid (171.9 mg, yield: 75%). mp: 145-147 °C. ^{1}H NMR (400 MHz, $CDCl_3$) δ 7.75 (dd, *J* = 8.1, 5.3 Hz, 1H), 7.41 (d, *J* = 7.7 Hz, 2H), 7.29 (d, *J* = 7.6 Hz, 3H), 7.19 (d, *J* = 7.6 Hz, 2H), 7.02 (t, *J* = 7.1 Hz, 1H), 6.46 (d, *J* = 6.4 Hz, 2H), 6.24 (d, *J* = 8.4 Hz, 1H), 4.82 (s, 2H), 2.41 (s, 3H). ^{19}F NMR (376 MHz, $CDCl_3$) δ -113.18. ^{13}C NMR (101 MHz, $CDCl_3$) δ 161.2 (d, *J* = 246.7 Hz), 144.2, 136.5, 136.0, 135.3, 133.2 (d, *J* = 8.5 Hz), 129.8 (d, *J* = 2.7 Hz), 129.5, 129.4, 129.3, 129.2, 128.3, 127.5, 116.8, 115.1 (d, *J* = 23.0 Hz), 113.1 (d, *J* = 24.8 Hz), 53.3, 21.5. HRMS (ESI) calcd for $C_{22}H_{18}{}^{79}BrFNO_2S^+$ [M + H$^+$] 458.0220, found 458.0224.

6-Fluoro-3-iodo-4-phenyl-1-tosyl-1,2-dihydroquinoline (4b-I)

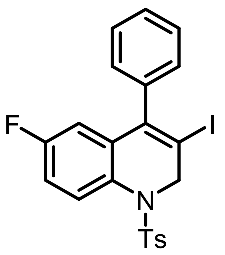

According to the procedure C, **4b-I** was obtained as brown oil (202.1 mg, yield: 80%). ^{1}H NMR (400 MHz, $CDCl_3$) δ 7.66 (dd, *J* = 8.9, 5.3 Hz, 1H), 7.35 (d, *J* = 8.3 Hz, 2H), 7.27 – 7.18 (m, 3H), 7.13 (d, *J* = 8.0 Hz, 2H), 6.94 (ddd, *J* = 8.8, 8.0, 2.9 Hz, 1H), 6.33 (dd, *J* = 7.8, 1.4 Hz, 2H), 6.16 (dd, *J* = 9.5, 2.9 Hz, 1H), 4.79 (s, 2H), 2.35 (s, 3H). ^{19}F NMR (376 MHz, $CDCl_3$) δ -113.21. ^{13}C NMR (101 MHz, $CDCl_3$) δ 161.0 (d, *J* = 246.7 Hz), 144.1, 142.4, 139.5, 135.5, 132.4 (d, *J* = 8.4 Hz), 130.1 (d, *J* = 2.8 Hz), 129.6, 129.2 (d, *J* = 8.6 Hz), 129.0, 128.4, 128.3, 127.6, 115.3 (d, *J* = 22.9 Hz), 113.3 (d, *J* = 24.7 Hz), 94.6, 57.0, 21.5. HRMS (ESI) calcd for $C_{22}H_{18}FINO_2S^+$ [M + H$^+$] 506.0081, found 506.0080.

3-Chloro-6-iodo-4-phenyl-1-tosyl-1,2-dihydroquinoline (4c-Cl)

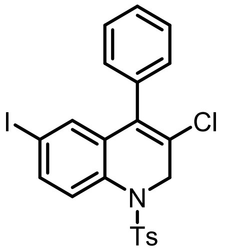

According to the procedure C, **4c-Cl** was obtained as a white solid (109.5 mg, yield: 42%). mp: 179-181

°C. ^{1}H NMR (400 MHz, $CDCl_3$) δ 7.62 (s, 1H), 7.52 (s, 1H), 7.41 (s, 2H), 7.31 (s, 3H), 7.20 (s, 2H), 6.85 (s, 1H), 6.49 (s, 2H), 4.69 (s, 2H), 2.40 (s, 3H). ^{13}C NMR (101 MHz, $CDCl_3$) δ 144.3, 137.1, 135.2, 134.8, 134.0, 133.6, 133.4, 133.0, 129.6, 129.4, 129.3, 128.3, 127.3, 125.0, 92.2, 51.2, 21.5. HRMS (ESI) calcd for $C_{22}H_{18}{}^{35}ClINO_2S^+$ [M + H^+] 521.9786, found 521.9789.

3-Bromo-6-iodo-4-phenyl-1-tosyl-1,2-dihydroquinoline (4c-Br)

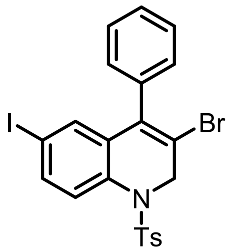

According to the procedure C, **4c-Br** was gained as a white solid (108.9 mg, yield: 77%). mp: 178-180 °C. ^{1}H NMR (400 MHz, $CDCl_3$) δ 7.64 (d, J = 7.6 Hz, 1H), 7.51 (d, J = 8.2 Hz, 1H), 7.43 (d, J = 7.6 Hz, 2H), 7.30 (d, J = 7.3 Hz, 3H), 7.20 (d, J = 7.5 Hz, 2H), 6.75 (d, J = 71.0 Hz, 1H), 6.46 (d, J = 6.1 Hz, 2H), 4.80 (s, 2H), 2.41 (s, 3H). ^{13}C NMR (101 MHz, $CDCl_3$) δ 144.2, 137.2, 136.0, 135.9, 135.4, 134.9, 133.7, 133.3, 129.6, 129.3, 129.2, 128.4, 127.4, 116.5, 92.2, 53.0, 21.5. HRMS (ESI) calcd for $C_{22}H_{18}{}^{79}BrINO_2S^+$ [M + H^+] 565.9281, found 565.9285.

3,6-Diiodo-4-phenyl-1-tosyl-1,2-dihydroquinoline (4c-I)

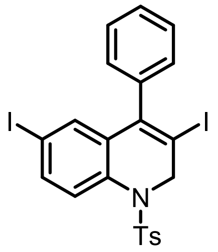

According to the procedure C, **4c-I** was obtained as brown oil (251.4 mg, yield: 82%). ^{1}H NMR (400 MHz, $CDCl_3$) δ 7.57 (dd, J = 8.5, 2.0 Hz, 1H), 7.42 (d, J = 8.5 Hz, 1H), 7.38 (d, J = 8.3 Hz, 2H), 7.29 – 7.18 (m, 3H), 7.15 (d, J = 8.1 Hz, 2H), 6.75 (d, J = 2.0 Hz, 1H), 6.36 – 6.29 (m, 2H), 4.78 (s, 2H), 2.36 (s, 3H). ^{13}C NMR (101 MHz, $CDCl_3$) δ 144.2, 141.9, 139.4, 137.4, 135.6, 135.1, 134.0, 132.5, 129.6, 129.2, 129.0, 128.5, 128.4, 127.6, 94.4, 92.0, 56.8, 21.5. HRMS (ESI) calcd for $C_{22}H_{18}I_2NO_2S^+$ [M + H^+] 613.9142, found 613.9140.

3-Chloro-6-methoxy-4-phenyl-1-tosyl-1,2-dihydroquinoline (4d-Cl)

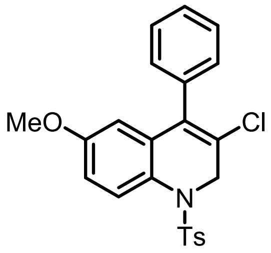

According to the procedure C, **4d-Cl** was gained as a white solid (45.3 mg, yield: 42%). mp: 117-119 °C. ^{1}H NMR (400 MHz, $CDCl_3$) δ 7.71 (d, *J* = 8.8 Hz, 1H), 7.39 (d, *J* = 8.2 Hz, 2H), 7.27 (q, *J* = 6.0 Hz, 3H), 7.16 (d, *J* = 8.0 Hz, 2H), 6.84 (dd, *J* = 8.8, 2.8 Hz, 1H), 6.50 (d, *J* = 6.2 Hz, 2H), 6.07 (d, *J* = 2.7 Hz, 1H), 4.69 (s, 2H), 3.65 (s, 3H), 2.39 (s, 3H). ^{13}C NMR (101 MHz, $CDCl_3$) δ 158.3, 143.9, 135.3, 134.6, 133.9, 132.6, 129.5, 129.4, 128.7, 128.1, 128.0, 127.5, 126.6, 124.4, 112.6, 112.3, 55.4, 51.5, 21.4. HRMS (ESI) calcd for $C_{23}H_{21}{}^{35}ClNO_3S^+$ [M + H$^+$] 426.0925, found 426.0923

3-Bromo-6-methoxy-4-phenyl-1-tosyl-1,2-dihydroquinoline (4d-Br)

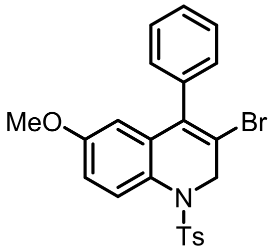

According to the procedure C, **4d-Br** was gained as a white solid (88.2 mg, yield: 75%). mp: 141-143 °C. ^{1}H NMR (400 MHz, $CDCl_3$) δ 7.71 (d, *J* = 8.8 Hz, 1H), 7.39 (d, *J* = 8.2 Hz, 2H), 7.27 (q, *J* = 6.0 Hz, 3H), 7.16 (d, *J* = 8.0 Hz, 2H), 6.84 (dd, *J* = 8.8, 2.8 Hz, 1H), 6.50 (d, *J* = 6.2 Hz, 2H), 6.07 (d, *J* = 2.7 Hz, 1H), 4.69 (s, 2H), 3.65 (s, 3H), 2.39 (s, 3H). ^{13}C NMR (101 MHz, $CDCl_3$) δ 158.3, 143.9, 137.0, 136.5, 135.4, 132.6, 129.4, 129.3, 128.7, 128.1, 128.1, 127.6, 126.8, 115.9, 112.8, 112.4, 55.4, 53.4, 21.5. HRMS (ESI) calcd for $C_{23}H_{21}{}^{79}BrNO_3S^+$ [M + H$^+$] 470.0420, found 470.0423.

3-Iodo-6-methoxy-4-phenyl-1-tosyl-1,2-dihydroquinoline (4d-I)

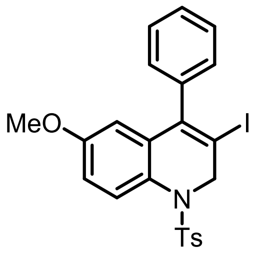

According to the procedure C, **4d-I** was gained as colorless oil (193.7 mg, yield: 75%). ^{1}H NMR (400 MHz, $CDCl_3$) δ 7.69 (d, *J* = 8.7 Hz, 1H), 7.43 (d, *J* = 7.7 Hz, 2H), 7.27 (d, *J* = 6.8 Hz, 3H), 7.19 (d, *J* = 7.6 Hz, 2H), 6.86 (d, *J* = 6.9 Hz, 1H), 6.40 (d, *J* = 6.3 Hz, 2H), 6.05 (s, 1H), 4.85 (s, 2H), 3.64 (s, 3H), 2.42 (s,

3H). ^{13}C NMR (101 MHz, $CDCl_3$) δ 158.1, 143.8, 143.0, 140.0, 135.7, 132.0, 129.5, 129.1, 128.6, 128.2, 128.1, 127.7, 127.4, 113.0, 112.6, 93.8, 57.1, 55.4, 21.5. HRMS (ESI) calcd for $C_{23}H_{21}INO_3S^+$ [M + H^+] 518.0281, found 518.0283.

General procedure D for halogenation of compounds 5: NXS (0.6 mmol, 1.20 equiv, X = Cl, Br, I) and thianthracene (5.41 mg, 0.025 mmol, 0.05 equiv) were dissolved in DCE (3.0 mL). With stirring of the mixture, trifluoromethanesulfonic acid (TfOH) (3.75 mg, 0.025 mmol, 0.05 equiv) was added. Then substrate **5** (0.5 mmol, 1.0 equiv) was added and the mixture was stirred at rt till the reaction was completed. The solvent of reaction mixture was removed by rotary evaporation. The residue was purified by silica gel column chromatography to afford the corresponding halogenated products.

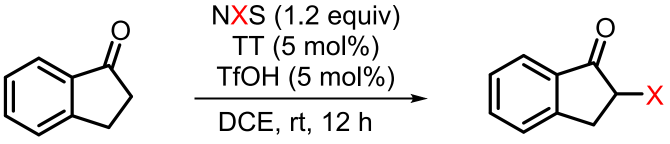

5 **5-X**

2-Chloro-2,3-dihydro-1*H*-inden-1-one (5a-Cl)

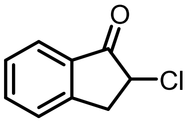

According to the procedure D, **5a-Cl** was obtained as colorless oil (68.3 mg, yield: 82%). ^1H NMR (400 MHz, $CDCl_3$) δ 7.81 (d, J = 7.7 Hz, 1H), 7.66 (t, J = 7.5 Hz, 1H), 7.43 (dd, J = 13.6, 7.1 Hz, 2H), 4.55 (dd, J = 7.8, 4.0 Hz, 1H), 3.77 (dd, J = 17.6, 7.8 Hz, 1H), 3.28 (dd, J = 17.6, 3.9 Hz, 1H). ^{13}C NMR (101 MHz, $CDCl_3$) δ 199.3, 150.8, 136.1, 133.8, 128.4, 126.4, 125.0, 55.8, 37.6. HRMS (ESI) calcd for $C_9H_8{}^{35}ClO^+$ [M + H^+] 167.0258, found 167.0259.

2-Bromo-2,3-dihydro-1*H*-inden-1-one (5a-Br)

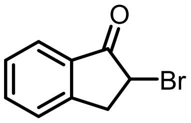

According to the procedure D, **5a-Br** was obtained as yellow oil (88.6 mg, yield: 84%). ^1H NMR (400 MHz, $CDCl_3$) δ 7.82 (d, J = 7.7 Hz, 1H), 7.66 (td, J = 7.7, 1.1 Hz, 1H), 7.49 – 7.39 (m, 2H), 4.64 (dd, J = 7.5, 3.2 Hz, 1H), 3.83 (dd, J = 18.1, 7.5 Hz, 1H), 3.41 (dd, J = 18.1, 3.1 Hz, 1H). ^{13}C NMR (101 MHz, $CDCl_3$) δ 199.6, 151.1, 136.0, 133.6, 128.3, 126.5, 125.1, 44.1, 38.0. HRMS (ESI) calcd for $C_9H_8{}^{79}BrO^+$ [M + H^+] 210.9753, found 210.9751.

2-Iodo-2,3-dihydro-1*H*-inden-1-one (5a-I)

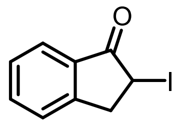

According to the procedure D, **5a-I** was obtained as brown oil (91.6 mg, yield: 71%). ^{1}H NMR (400 MHz, $CDCl_3$) δ 7.85 (d, *J* = 7.3 Hz, 1H), 7.66 (td, *J* = 7.5, 1.1 Hz, 1H), 7.42 (dd, *J* = 10.9, 4.0 Hz, 2H), 4.95 (dd, *J* = 7.4, 2.7 Hz, 1H), 3.88 (dd, *J* = 18.4, 7.4 Hz, 1H), 3.47 (dd, *J* = 18.4, 2.5 Hz, 1H). ^{13}C NMR (101 MHz, $CDCl_3$) δ 201.5, 151.3, 135.6, 132.9, 128.2, 126.5, 125.1, 39.6, 19.3. HRMS (ESI) calcd for $C_9H_8IO^+$ [M + H^+] 258.9614, found 258.9616.

2-Chloro-3,4-dihydronaphthalen-1(2*H*)-one (5b-Cl)

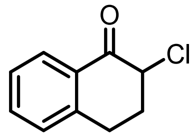

According to the procedure D, **5b-Cl** was obtained as colorless oil (72.3 mg, yield: 80%). ^{1}H NMR (400 MHz, $CDCl_3$) δ 8.06 (dd, *J* = 7.9, 1.0 Hz, 1H), 7.51 (td, *J* = 7.5, 1.4 Hz, 1H), 7.33 (t, *J* = 7.5 Hz, 1H), 7.26 (d, *J* = 7.5 Hz, 1H), 4.62 (dd, *J* = 7.8, 3.9 Hz, 1H), 3.27 (ddd, *J* = 17.1, 7.9, 4.6 Hz, 1H), 2.99 (ddd, *J* = 17.1, 6.8, 4.8 Hz, 1H), 2.65 – 2.52 (m, 1H), 2.44 (tdd, *J* = 12.2, 7.3, 4.6 Hz, 1H). ^{13}C NMR (101 MHz, $CDCl_3$) δ 190.8, 143.2, 134.2, 130.5, 128.8, 128.5, 127.1, 59.9, 32.5, 26.3. HRMS (ESI) calcd for $C_{10}H_{10}{}^{35}ClO^+$ [M + H^+] 181.0415, found 181.0416.

2-Bromo-3,4-dihydronaphthalen-1(2*H*)-one (5b-Br)

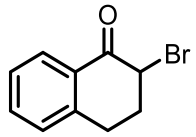

According to the procedure D, **5b-Br** was obtained as yellow oil (95.7 mg, yield: 85%). ^{1}H NMR (400 MHz, $CDCl_3$) δ 8.08 (dd, *J* = 7.8, 0.7 Hz, 1H), 7.52 (td, *J* = 7.5, 1.3 Hz, 1H), 7.34 (t, *J* = 7.6 Hz, 1H), 7.27 (d, *J* = 7.7 Hz, 1H), 4.80 – 4.66 (m, 1H), 3.39 – 3.23 (m, 1H), 2.91 (dt, *J* = 17.1, 4.4 Hz, 1H), 2.59 – 2.38 (m, 2H). ^{13}C NMR (101 MHz, $CDCl_3$) δ 190.6, 143.0, 134.2, 130.0, 128.8, 128.7, 127.1, 50.6, 32.0, 26.2. HRMS (ESI) calcd for $C_{10}H_{10}{}^{79}BrO^+$ [M + H^+] 224.9910, found 224.9915.

2-Iodo-3,4-dihydronaphthalen-1(2*H*)-one (5b-I)

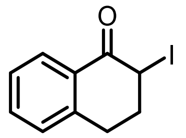

According to the procedure D, **5b-I** was obtained as a yellow solid (100.7 mg, yield: 74%). mp: 77-79 °C. ^{1}H NMR (400 MHz, $CDCl_3$) δ 8.09 (dd, *J* = 7.9, 1.0 Hz, 1H), 7.51 (td, *J* = 7.5, 1.4 Hz, 1H), 7.35 (t, *J* = 7.6 Hz, 1H), 7.32 – 7.23 (m, 1H), 5.02 (t, *J* = 3.5 Hz, 1H), 3.24 – 3.05 (m, 1H), 2.88 (dt, *J* = 17.2, 3.8 Hz, 1H), 2.28 (ddd, *J* = 14.9, 7.9, 3.9 Hz, 1H), 2.20 – 2.07 (m, 1H). ^{13}C NMR (101 MHz, $CDCl_3$) δ 191.9, 142.8, 134.0, 129.4, 128.8, 128.8, 127.1, 32.7, 30.7, 27.9. HRMS (ESI) calcd for $C_{10}H_{10}IO^+$ [M + H$^+$] 272.9771, found 272.9767.

6-Chloro-6,7,8,9-tetrahydro-5*H*-benzo[7]annulen-5-one (5c-Cl)

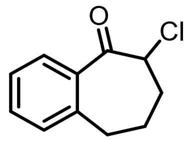

According to the procedure D, **5c-Cl** was obtained as yellow oil (78.8 mg, yield: 81%). ^{1}H NMR (400 MHz, $CDCl_3$) δ 7.64 (dd, *J* = 7.7, 1.3 Hz, 1H), 7.41 (td, *J* = 7.5, 1.4 Hz, 1H), 7.30 (t, *J* = 7.2 Hz, 1H), 7.21 (d, *J* = 7.6 Hz, 1H), 4.80 (dd, *J* = 8.7, 4.7 Hz, 1H), 3.02 (ddd, *J* = 15.8, 7.7, 3.3 Hz, 1H), 2.92 (ddd, *J* = 15.8, 9.6, 3.3 Hz, 1H), 2.39 (dddd, *J* = 14.3, 9.7, 6.1, 4.8 Hz, 1H), 2.20 (ddt, *J* = 14.1, 8.7, 5.3 Hz, 1H), 2.12 – 1.83 (m, 2H). ^{13}C NMR (101 MHz, $CDCl_3$) δ 199.5, 140.4, 137.5, 132.2, 129.9, 129.4, 126.8, 63.5, 34.0, 33.8, 23.7. HRMS (ESI) calcd for $C_{11}H_{12}{}^{35}ClO^+$ [M + H$^+$] 195.0571, found 195.0575.

6-Bromo-6,7,8,9-tetrahydro-5*H*-benzo[7]annulen-5-one (5c-Br)

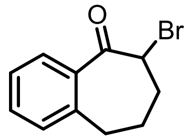

According to the procedure D, **5c-Br** was gained as yellow oil (100.4 mg, yield: 82%). ^{1}H NMR (400 MHz, $CDCl_3$) δ 7.52 (d, *J* = 7.6 Hz, 1H), 7.34 (t, *J* = 7.1 Hz, 1H), 7.21 (t, *J* = 7.5 Hz, 1H), 7.12 (d, *J* = 7.5 Hz, 1H), 4.78 (dd, *J* = 7.8, 4.1 Hz, 1H), 2.95 (m, 1H), 2.87 – 2.75 (m, 1H), 2.31 (m, 1H), 2.20 (m, 1H), 2.01 – 1.87 (m, 2H). ^{13}C NMR (101 MHz, $CDCl_3$) δ 200.0, 139.6, 137.7, 132.2, 129.7, 129.6, 126.7, 54.4, 33.8, 33.3, 24.0. HRMS (ESI) calcd for $C_{11}H_{12}{}^{79}BrO^+$ [M + H$^+$] 239.0066, found 239.0063.

6-Iodo-6,7,8,9-tetrahydro-5*H*-benzo[7]annulen-5-one (5c-I)

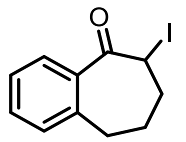

According to the procedure D, **5c-I** was gained as yellow oil (99.8 mg, yield: 70%). ^{1}H NMR (400 MHz, $CDCl_3$) δ 7.57 (d, *J* = 7.6 Hz, 1H), 7.41 (td, *J* = 7.5, 1.3 Hz, 1H), 7.28 (t, *J* = 7.6 Hz, 1H), 7.18 (d, *J* = 7.5

Hz, 1H), 5.16 – 5.05 (m, 1H), 3.06 – 2.94 (m, 1H), 2.93 – 2.83 (m, 1H), 2.24 – 2.11 (m, 2H), 2.02 – 1.88 (m, 2H). ^{13}C NMR (101 MHz, $CDCl_3$) δ 201.7, 138.9, 137.7, 132.2, 129.7, 129.5, 126.7, 33.4, 33.3, 33.3, 25.5. HRMS (ESI) calcd for $C_{11}H_{12}IO^+$ [M + H^+] 286.9927, found 286.9923.

5-Bromo-2-chloro-2,3-dihydro-1*H*-inden-1-one (5d-Cl)

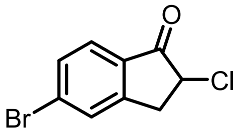

According to the procedure D, **5d-Cl** was obtained as a white solid (103.1 mg, yield: 84%). mp: 91-93 °C. ^1H NMR (400 MHz, $CDCl_3$) δ 7.69 (d, *J* = 8.2 Hz, 1H), 7.64 (s, 1H), 7.58 (d, *J* = 8.2 Hz, 1H), 4.55 (dd, *J* = 7.8, 4.0 Hz, 1H), 3.77 (dd, *J* = 17.8, 7.8 Hz, 1H), 3.29 (dd, *J* = 17.8, 3.9 Hz, 1H). ^{13}C NMR (101 MHz, $CDCl_3$) δ 198.0, 152.2, 132.7, 132.1, 131.7, 129.8, 126.2, 55.3, 37.02. HRMS (ESI) calcd for $C_9H_7{}^{79}Br$ $^{35}ClO^+$ [M + H^+] 244.9363, found 244.9362.

2,5-Dibromo-2,3-dihydro-1*H*-inden-1-one (5d-Br)

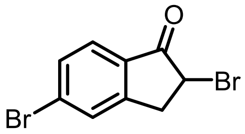

According to the procedure D, **5d-Br** was obtained as a white solid (114.5 mg, yield: 79%). mp: 98-100 °C. ^1H NMR (400 MHz, $CDCl_3$) δ 7.66 (d, *J* = 8.2 Hz, 1H), 7.61 (d, *J* = 0.8 Hz, 1H), 7.58 – 7.51 (m, 1H), 4.62 (dd, *J* = 7.5, 3.1 Hz, 1H), 3.81 (dd, *J* = 18.3, 7.5 Hz, 1H), 3.38 (dd, *J* = 18.3, 3.0 Hz, 1H). ^{13}C NMR (101 MHz, $CDCl_3$) δ 198.4, 152.6, 132.4, 132.0, 131.5, 129.8, 126.2, 43.5, 37.6. HRMS (ESI) calcd for $C_9H_7{}^{79}Br_2O^+$ [M + H^+] 288.8858, found 288.8856.

5-Bromo-2-iodo-2,3-dihydro-1*H*-inden-1-one (5d-I)

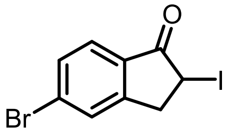

According to the procedure D, **5d-I** was obtained as brown oil (117.9 mg, yield: 70%). ^1H NMR (400 MHz, $CDCl_3$) δ 7.68 (d, *J* = 8.2 Hz, 1H), 7.60 (d, *J* = 0.7 Hz, 1H), 7.58 – 7.52 (m, 1H), 4.92 (dd, *J* = 7.4, 2.6 Hz, 1H), 3.86 (dd, *J* = 18.6, 7.4 Hz, 1H), 3.43 (dd, *J* = 18.6, 2.3 Hz, 1H). ^{13}C NMR (101 MHz, $CDCl_3$) δ 200.3, 152.7, 131.9, 131.7, 131.2, 129.8, 126.2, 39.3, 18.6. HRMS (ESI) calcd for $C_9H_7{}^{79}BrIO^+$ [M + H^+] 336.8719, found 336.8722.

2-Chloro-2-methyl-2,3-dihydro-1*H*-inden-1-one (5e-Cl)

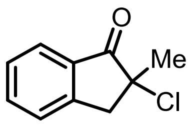

According to the procedure D, **5e-Cl** was obtained as yellow oil (70.4 mg, yield: 78%). ^{1}H NMR (400 MHz, $CDCl_3$) δ 7.85 – 7.78 (m, 1H), 7.64 (td, *J* = 7.6, 1.1 Hz, 1H), 7.47 – 7.37 (m, 2H), 3.63 (d, *J* = 17.8 Hz, 1H), 3.43 (d, *J* = 17.8 Hz, 1H), 1.78 (s, 3H). ^{13}C NMR (101 MHz, $CDCl_3$) δ 200.4, 149.6, 136.1, 132.9, 128.3, 126.5, 125.6, 66.8, 45.5, 26.2. HRMS (ESI) calcd for $C_{10}H_{10}{}^{35}ClO^+$ [M + H$^+$] 181.0415, found 181.0412.

2-Chloro-2-methyl-2,3-dihydro-1*H*-inden-1-one (5e-Br)

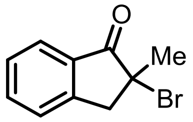

According to the procedure D, **5e-Br** was obtained as yellow oil (75.4 mg, yield: 67%). ^{1}H NMR (400 MHz, $CDCl_3$) δ 7.84 (d, *J* = 7.4 Hz, 1H), 7.64 (td, *J* = 7.6, 1.1 Hz, 1H), 7.42 (t, *J* = 7.5 Hz, 2H), 3.77 (d, *J* = 18.2 Hz, 1H), 3.47 (d, *J* = 18.2 Hz, 1H), 1.94 (s, 3H). ^{13}C NMR (101 MHz, $CDCl_3$) δ 200.3, 149.1, 135.9, 132.7, 128.3, 126.4, 125.6, 59.6, 46.4, 26.8. HRMS (ESI) calcd for $C_{10}H_{10}{}^{79}BrO^+$ [M + H$^+$] 224.9910, found 224.9914.

2-Iodo-2-methyl-2,3-dihydro-1*H*-inden-1-one (5e-I)

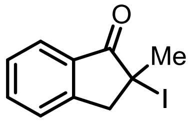

According to the procedure D, **5e-I** was obtained as yellow oil (61.2 mg, yield: 45%). mp: ^{1}H NMR (400 MHz, $CDCl_3$) δ 7.87 (d, *J* = 7.7 Hz, 1H), 7.65 (td, *J* = 7.5, 1.1 Hz, 1H), 7.48 – 7.36 (m, 2H), 3.85 (d, *J* = 18.5 Hz, 1H), 3.47 (d, *J* = 18.4 Hz, 1H), 2.09 (s, 3H). ^{13}C NMR (101 MHz, $CDCl_3$) δ 202.0, 148.3, 135.5, 131.9, 128.3, 126.3, 125.7, 49.0, 40.7, 29.6. HRMS (ESI) calcd for $C_{10}H_{10}IO^+$ [M + H$^+$] 272.9771, found 272.9772.

2-Chloro-2-methyl-3,4-dihydronaphthalen-1(2*H*)-one (5f-Cl)

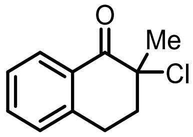

According to the procedure D, **5f-Cl** was obtained as yellow oil (76.9 mg, yield: 79%). ^{1}H NMR (400 MHz, $CDCl_3$) δ 8.10 (dd, *J* = 7.9, 1.0 Hz, 1H), 7.50 (td, *J* = 7.5, 1.4 Hz, 1H), 7.34 (t, *J* = 7.6 Hz, 1H), 7.25

(d, J = 7.3 Hz, 1H), 3.39 (ddd, J = 16.4, 11.3, 4.7 Hz, 1H), 2.93 – 2.82 (m, 1H), 2.50 (ddd, J = 14.5, 4.7, 3.1 Hz, 1H), 2.33 (ddd, J = 14.6, 11.3, 4.7 Hz, 1H), 1.83 (s, 3H). ^{13}C NMR (101 MHz, $CDCl_3$) δ 191.4, 143.1, 133.8, 129.8, 128.94, 128.7, 127.0, 67.6, 38.5, 26.7, 26.0. HRMS (ESI) calcd for $C_{11}H_{12}{}^{35}ClO^+$ [M + H^+] 195.0571, found 195.0577.

2-Bromo-2-methyl-3,4-dihydronaphthalen-1(2*H*)-one (5f-Br)

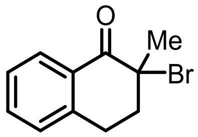

According to the procedure D, **5f-Br** was obtained as yellow oil (76.5 mg, yield: 64%). ^{1}H NMR (400 MHz, $CDCl_3$) δ 8.04 (d, J = 7.8 Hz, 1H), 7.42 (t, J = 7.3 Hz, 1H), 7.26 (t, J = 7.5 Hz, 1H), 7.17 (d, J = 7.3 Hz, 1H), 3.26 (ddd, J = 16.6, 11.9, 4.3 Hz, 1H), 2.82 (d, J = 15.9 Hz, 1H), 2.42 (dd, J = 14.8, 1.7 Hz, 1H), 2.20 – 2.05 (m, 1H), 1.95 (s, 3H). ^{13}C NMR (101 MHz, $CDCl_3$) δ 191.1, 142.8, 133.8, 129.5, 129.0, 128.7, 127.0, 63.5, 39.3, 28.0, 27.4. HRMS (ESI) calcd for $C_{11}H_{12}{}^{79}BrO^+$ [M + H^+] 239.0066, found 239.0062.

2-Iodo-2-methyl-3,4-dihydronaphthalen-1(2*H*)-one (5f-I)

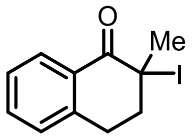

According to the procedure D, **5f-I** was obtained as yellow oil (81.5 mg, yield: 57%). ^{1}H NMR (400 MHz, $CDCl_3$) δ 8.06 (dd, J = 7.9, 1.0 Hz, 1H), 7.42 (td, J = 7.5, 1.4 Hz, 1H), 7.28 (t, J = 7.6 Hz, 1H), 7.22 – 7.17 (m, 1H), 3.04 (ddd, J = 16.5, 11.8, 4.5 Hz, 1H), 2.86 (ddd, J = 17.4, 4.6, 2.3 Hz, 1H), 2.28 (ddd, J = 15.1, 4.5, 2.4 Hz, 1H), 2.20 (s, 3H), 1.50 (ddd, J = 16.4, 12.0, 4.6 Hz, 2H). ^{13}C NMR (101 MHz, $CDCl_3$) δ 192.5, 142.4, 133.6, 129.0, 129.0, 128.7, 127.0, 49.7, 41.4, 31.1, 30.2. HRMS (ESI) calcd for $C_{11}H_{12}IO^+$ [M + H^+] 286.9927, found 286.9931.

2,2-Dichloro-2,3-dihydro-1*H*-inden-1-one (5a-Cl$_2$)

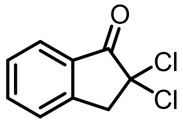

According to the procedure D, **5a-Cl$_2$** was obtained as colorless oil (80.4 mg, yield: 80%). mp: 64-66 °C. ^{1}H NMR (400 MHz, $CDCl_3$) δ 7.92 (d, J = 7.7 Hz, 1H), 7.74 (td, J = 7.6, 1.1 Hz, 1H), 7.55 – 7.47 (m, 1H), 7.43 (d, J = 7.7 Hz, 1H), 4.05 (s, 2H). ^{13}C NMR (101 MHz, $CDCl_3$) δ 191.8, 147.2, 137.1, 130.4, 129.1, 126.6, 126.2, 81.5, 50.2. HRMS (ESI) calcd for $C_9H_7{}^{35}Cl_2O^+$ [M + H^+] 200.9868, found 200.9872.

General procedure A for halogenation of natural products and drugs 6: NXS (0.6 mmol, 1.20 equiv, X = Cl, Br, I) and thianthracene (5.41 mg, 0.025 mmol, 0.05 equiv) were dissolved in DCE (3.0 mL). With stirring of the mixture, trifluoromethanesulfonic acid (TfOH) (3.75 mg, 0.025 mmol, 0.05 equiv) was added. Then substrate **6** (0.5 mmol, 1.0 equiv) was added and the mixture was stirred at rt till the reaction was completed. The solvent of reaction mixture was removed by rotary evaporation. The residue was purified by silica gel column chromatography to afford the corresponding halogenated products.

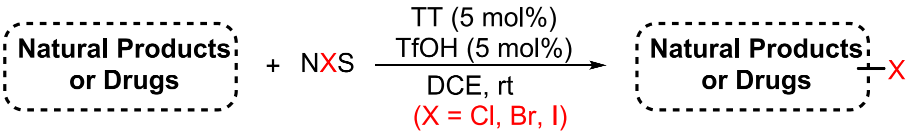

2-(5-Chloro-6-methoxynaphthalen-2-yl)propanoic acid (6a-Cl)

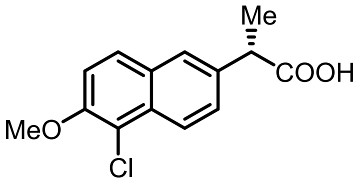

According to the procedure A, **6a-Cl** was obtained as a white solid (121.5 mg, yield: 90%). mp: 150-152 °C. ^{1}H NMR (400 MHz, DMSO) δ 12.41 (s, 1H), 8.05 (d, *J* = 8.8 Hz, 1H), 7.96 (d, *J* = 9.1 Hz, 1H), 7.84 (s, 1H), 7.58 (dd, *J* = 14.1, 5.2 Hz, 2H), 3.99 (s, 3H), 1.46 (d, *J* = 7.1 Hz, 3H). ^{13}C NMR (101 MHz, DMSO) δ 175.7, 152.7, 137.6, 130.4, 129.4, 128.7, 128.5, 126.7, 123.3, 115.2, 114.9, 57.2, 44.9, 18.8. HRMS (ESI) calcd for $C_{14}H_{14}{}^{35}ClO_3^+$ [M + H$^+$] 265.0626, found 265.0629.

2-(5-Bromo-6-methoxynaphthalen-2-yl)propanoic acid (6a-Br)

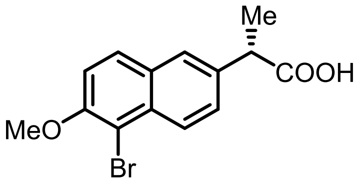

According to the procedure A, **6a-Br** was gained as a white solid (128.2 mg, yield: 95%). mp: 167-169 °C. ^{1}H NMR (400 MHz, DMSO) δ 12.42 (s, 1H), 8.04 (d, *J* = 8.8 Hz, 1H), 7.99 (d, *J* = 9.1 Hz, 1H), 7.83 (s, 1H), 7.58 (dd, *J* = 8.8, 1.6 Hz, 1H), 7.52 (d, *J* = 9.1 Hz, 1H), 3.99 (s, 3H), 3.86 (q, *J* = 7.1 Hz, 1H), 1.47 (d, *J* = 7.1 Hz, 3H). ^{13}C NMR (101 MHz, DMSO) δ 175.8, 153.9, 137.6, 131.8, 129.8, 129.6, 128.7, 126.8, 125.9, 114.9, 107.2, 57.3, 44.8, 18.8. HRMS (ESI) calcd for $C_{14}H_{14}{}^{79}BrO_3^+$ [M + H$^+$] 309.0121, found 309.0120.

2-(5-Iodo-6-methoxynaphthalen-2-yl)propanoic acid (6a-I)

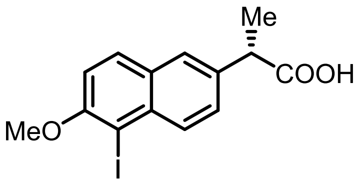

According to the procedure A, **6a-I** was obtained as a white solid (156.7 mg, yield: 88%). mp: 154-156 °C. ^{1}H NMR (400 MHz, DMSO) δ 12.39 (s, 1H), 7.98 (dd, *J* = 8.9, 2.8 Hz, 2H), 7.78 (d, *J* = 1.4 Hz, 1H), 7.53 (dd, *J* = 8.9, 1.8 Hz, 1H), 7.43 (d, *J* = 9.1 Hz, 1H), 3.97 (s, 3H), 1.46 (d, *J* = 7.1 Hz, 3H). ^{13}C NMR (101 MHz, DMSO) δ 175.8, 156.9, 137.5, 134.4, 131.0, 130.8, 129.8, 128.9, 126.8, 114.3, 87.1, 57.6, 44.7, 18.8. HRMS (ESI) calcd for $C_{14}H_{14}IO_3^+$ [M + H$^+$] 356.9982, found 356.9986.

Methyl 2-(5-chloro-2-((2,6-dichlorophenyl)amino)phenyl)acetate (6b-Cl)

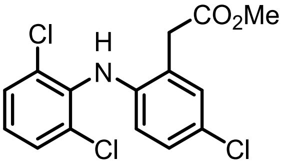

According to the procedure A, **6b-Cl** was obtained as a white solid (106.7 mg, yield: 79%). mp: 86-88 °C. ^{1}H NMR (400 MHz, $CDCl_3$) δ 7.26 (d, *J* = 8.1 Hz, 2H), 7.13 (d, *J* = 2.1 Hz, 1H), 7.00 (dd, *J* = 8.6, 2.2 Hz, 1H), 6.92 (t, *J* = 8.0 Hz, 1H), 6.83 (s, 1H), 6.38 (d, *J* = 8.6 Hz, 1H), 3.69 (s, 2H), 3.68 (s, 3H). ^{13}C NMR (101 MHz, $CDCl_3$) δ 172.1, 141.4, 137.4, 130.6, 129.6, 129.0, 127.9, 126.7, 125.6, 124.5, 119.3, 52.6, 38.3. HRMS (ESI) calcd for $C_{15}H_{13}{}^{35}Cl_3NO_2^+$ [M + H$^+$] 344.0006, found 344.0008.

Methyl 2-(5-bromo-2-((2,6-dichlorophenyl)amino)phenyl)acetate (6b-Br)

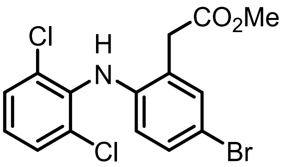

According to the procedure A, **6b-Br** was gained as a white solid (161.4 mg, yield: 83%). mp: 104-106 °C. ^{1}H NMR (400 MHz, $CDCl_3$) δ 7.34 (d, *J* = 7.6 Hz, 3H), 7.21 (d, *J* = 7.9 Hz, 1H), 7.00 (t, *J* = 7.8 Hz, 1H), 6.91 (s, 1H), 6.40 (d, *J* = 8.4 Hz, 1H), 3.76 (s, 5H). ^{13}C NMR (101 MHz, $CDCl_3$) δ 172.1, 142.0, 137.2, 133.5, 130.9, 129.7, 129.0, 125.9, 124.6, 119.6, 114.0, 52.6, 38.2. HRMS (ESI) calcd for $C_{15}H_{13}{}^{79}Br^{35}Cl_2NO_2^+$ [M + H$^+$] 387.9501, found 387.9504.

Methyl 2-(2-((2,6-dichlorophenyl)amino)-5-iodophenyl)acetate (6b-I)

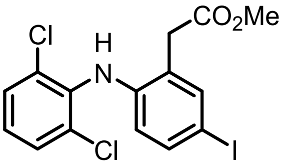

According to the procedure A, **6b-I** was gained as a white solid (176.6 mg, yield: 81%). mp: 118-120 °C. ^{1}H NMR (400 MHz, $CDCl_3$) δ 7.53 (s, 1H), 7.37 (dd, *J* = 15.4, 8.1 Hz, 3H), 7.10 – 6.99 (m, 1H), 6.94 (s, 1H), 6.28 (d, *J* = 8.0 Hz, 1H), 3.75 (d, *J* = 6.3 Hz, 5H).^{13}C NMR (101 MHz, $CDCl_3$) δ 172.1, 142.7, 139.3, 137.1, 136.8, 129.8, 129.0, 126.2, 124.7, 119.8, 84.0, 52.6, 38.0. HRMS (ESI) calcd for $C_{15}H_{13}{}^{35}Cl_2INO_2^+$ [M + H$^+$] 435.9363, found 435.9361.

Methyl 2-(2-bromo-4-(2-(4-chlorobenzamido)ethyl)phenoxy)-2-methylpropano-ate (6c-Br)

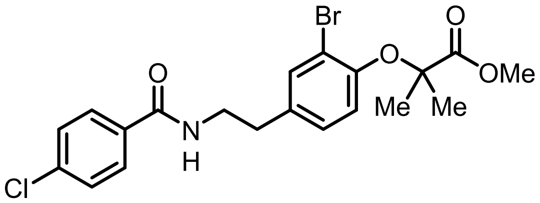

According to the procedure A, **6c-Br** was obtained as yellow oil (191.0 mg, yield: 84%). ^{1}H NMR (400 MHz, $CDCl_3$) δ 7.61 (d, *J* = 8.3 Hz, 2H), 7.43 – 7.29 (m, 3H), 6.98 (d, *J* = 8.2 Hz, 1H), 6.74 (d, *J* = 8.3 Hz, 1H), 6.47 (s, 1H), 3.76 (s, 3H), 3.58 (dd, *J* = 13.0, 6.6 Hz, 2H), 2.80 (t, *J* = 6.9 Hz, 2H), 1.59 (s, 6H). ^{13}C NMR (101 MHz, $CDCl_3$) δ 174.6, 166.6, 151.1, 137.7, 134.5, 133.7, 132.9, 128.8, 128.4, 119.6, 116.4, 80.8, 52.6, 41.1, 34.5, 25.2. HRMS (ESI) calcd for $C_{20}H_{22}{}^{79}Br^{35}ClNO_4^+$ [M + H$^+$] 454.0415, found 454.0417.

Methyl 2-(4-(2-(4-chlorobenzamido)ethyl)-2-iodophenoxy)-2-methylpropanoate (6c-I)

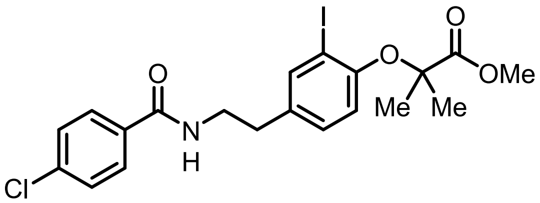

According to the procedure A, **6c-I** was gained as yellow oil (203.2 mg, yield: 81%). ^{1}H NMR (400 MHz, $CDCl_3$) δ 7.63 (d, *J* = 1.7 Hz, 2H), 7.61 (s, 1H), 7.36 (d, *J* = 8.4 Hz, 2H), 7.03 (dd, *J* = 8.3, 1.8 Hz, 1H), 6.65 (d, *J* = 8.3 Hz, 1H), 6.25 (s, 1H), 3.78 (s, 3H), 3.61 (dd, *J* = 13.0, 6.7 Hz, 2H), 2.80 (t, *J* = 6.9 Hz, 2H), 1.63 (s, 6H). ^{13}C NMR (101 MHz, $CDCl_3$) δ 174.6, 166.5, 153.7, 139.8, 137.7, 134.7, 132.9, 129.4, 128.9, 128.3, 117.7, 91.5, 80.8, 52.6, 41.1, 34.3, 25.3. HRMS (ESI) calcd for $C_{20}H_{20}{}^{35}ClINO_4^+$ [M + H$^+$] 502.0277, found 502.0281.

***N*-(2-(4-Bromophenoxy)-4-nitrophenyl)methanesulfonamide (6d-Br)**

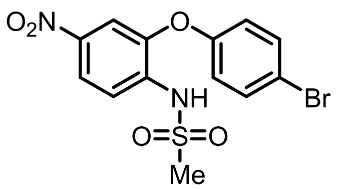

According to the procedure A, **6d-Br** was obtained as a white solid (172.3 mg, yield: 89%). mp: 117-119 °C. ^{1}H NMR (400 MHz, $CDCl_3$) δ 8.02 (d, *J* = 7.6 Hz, 1H), 7.77 (d, *J* = 9.0 Hz, 1H), 7.65 (s, 1H), 7.58 (d, *J* = 8.4 Hz, 2H), 7.37 (s, 1H), 6.97 (d, *J* = 8.4 Hz, 2H), 3.18 (s, 3H). ^{13}C NMR (101 MHz, $CDCl_3$) δ 153.3, 145.8, 143.6, 134.0, 133.8, 121.4, 119.8, 118.9, 117.4, 111.9, 40.7. HRMS (ESI) calcd for $C_{13}H_{12}{}^{79}BrN_2O_5S^+$ [M + H$^+$] 386.9645, found 386.9643.

***N*-(2-(4-Iodophenoxy)-4-nitrophenyl)methanesulfonamide (6d-I)**

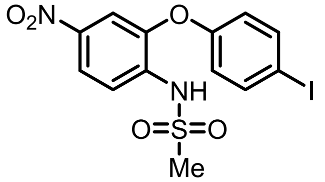

According to the procedure A, **6d-I** was gained as a white solid (186.7 mg, yield: 86%). mp: 184-186 °C. ^{1}H NMR (400 MHz, DMSO) δ 10.18 (s, 1H), 8.06 (dd, *J* = 9.0, 2.3 Hz, 1H), 7.77 (dd, *J* = 13.7, 8.9 Hz, 3H), 7.64 (d, *J* = 2.3 Hz, 1H), 6.98 (d, *J* = 8.6 Hz, 2H), 3.19 (s, 3H). ^{13}C NMR (101 MHz, DMSO) δ 155.9, 146.9, 143.6, 139.3, 136.5, 122.0, 121.6, 120.4, 114.0, 89.0, 41.4. HRMS (ESI) calcd for $C_{13}H_{12}IN_2O_5S^+$ [M + H$^+$] 434.9506, found 434.9509.

***N*-(2-(1-(3-Chloro-5-ethoxy-4-methoxyphenyl)-2-(methylsulfonyl)ethyl)-1,3-dioxoisoindolin-4-yl)acetamide (6e-Cl)**

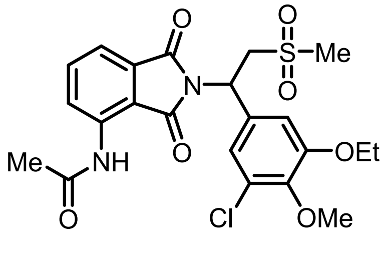

According to the procedure A, **6e-Cl** was gained as a white solid (230.1 mg, yield: 93%). mp: 100-102 °C. ^{1}H NMR (400 MHz, $CDCl_3$) δ 9.45 (s, 1H), 8.75 (d, *J* = 8.4 Hz, 1H), 7.70 – 7.61 (m, 1H), 7.49 (d, *J* = 7.3 Hz, 1H), 7.28 (s, 1H), 6.85 (s, 1H), 6.33 (dd, *J* = 11.7, 3.0 Hz, 1H), 4.50 (dd, *J* = 14.3, 12.0 Hz, 1H), 4.14 – 4.01 (m, 2H), 3.83 (s, 3H), 3.54 – 3.41 (m, 1H), 3.00 (s, 3H), 2.24 (s, 3H), 1.44 (t, *J* = 7.0 Hz, 3H). ^{13}C NMR (101 MHz, $CDCl_3$) δ 177.4, 169.6, 169.3, 168.0, 150.2, 147.6, 137.7, 136.2, 131.1, 126.0, 125.1, 124.1, 118.3, 115.1, 113.4, 112.7, 64.9, 56.2, 53.7, 45.8, 41.0, 29.6, 25.0. HRMS (ESI) calcd for $C_{22}H_{24}I^{35}ClN_2O_7S^+$ [M + H$^+$] 495.0987, found 495.0985.

1-((4-Bromonaphthalen-1-yl)oxy)-3-(isopropylamino)propan-2-ol (6f-Br)

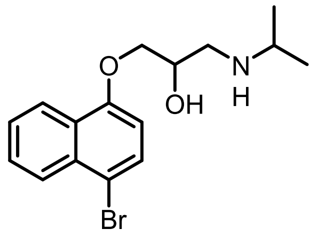

According to the procedure A, **6f-Br** was obtained as a white solid (139.6 mg, yield: 79%). mp: 107-109 °C. ^{1}H NMR (400 MHz, $CDCl_3$) δ 8.26 (d, *J* = 8.3 Hz, 1H), 8.16 (d, *J* = 8.4 Hz, 1H), 7.62 (dd, *J* = 16.1, 7.9 Hz, 2H), 7.52 (t, *J* = 7.5 Hz, 1H), 6.69 (d, *J* = 8.2 Hz, 1H), 4.26 – 4.08 (m, 3H), 3.03 (dd, *J* = 12.2, 3.6 Hz, 1H), 2.90 (ddd, *J* = 19.9, 12.4, 7.0 Hz, 2H), 2.70 (s, 2H), 1.22 – 1.07 (m, 6H). ^{13}C NMR (101 MHz, $CDCl_3$) δ 154.2, 132.5, 129.4, 127.8, 127.0, 126.8, 126.1, 122.3, 113.7, 105.7, 70.9, 68.2, 49.3, 49.2, 22.9, 22.7. HRMS (ESI) calcd for $C_{16}H_{21}{}^{79}BrNO_2^+$ [M + H$^+$] 338.0750, found 338.0751.

4-Chloro-9-methoxy-7*H*-furo[3,2-g]chromen-7-one (6g-Cl)

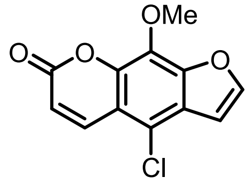

According to the procedure A, **6g-Cl** was obtained as a white solid (106.5 mg, yield: 85%). mp: 186-188 °C. ^{1}H NMR (400 MHz, $CDCl_3$) δ 8.13 (d, *J* = 9.9 Hz, 1H), 7.70 (d, *J* = 2.2 Hz, 1H), 6.91 (d, *J* = 2.2 Hz, 1H), 6.44 (d, *J* = 9.9 Hz, 1H), 4.27 (s, 3H). ^{13}C NMR (101 MHz, $CDCl_3$) δ 159.7, 147.1, 147.0, 143.6, 140.2, 131.9, 125.7, 116.2, 115.5, 114.2, 105.8, 61.5. HRMS (ESI) calcd for $C_{12}H_8{}^{35}ClO_4^+$ [M + H$^+$] 251.0106, found 251.0110.

4-Bromo-9-methoxy-7*H*-furo[3,2-g]chromen-7-one (6g-Br)

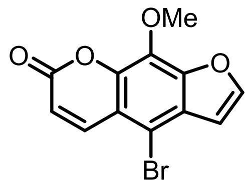

According to the procedure A, **6g-Br** was obtained as a white solid (135.7 mg, yield: 92%). mp: 183-185 °C. ^{1}H NMR (400 MHz, $CDCl_3$) δ 8.10 (d, *J* = 9.7 Hz, 1H), 7.72 (s, 1H), 6.86 (s, 1H), 6.43 (d, *J* = 9.6 Hz, 1H), 4.27 (s, 3H). ^{13}C NMR (101 MHz, $CDCl_3$) δ 159.7, 146.9, 146.6, 143.7, 142.6, 132.4, 128.0, 115.8, 115.7, 107.4, 105.5, 61.4. HRMS (ESI) calcd for $C_{12}H_8{}^{79}BrO_4^+$ [M + H$^+$] 294.9600, found 294.9603.

4-Iodo-9-methoxy-7*H*-furo[3,2-g]chromen-7-one (6g-I)

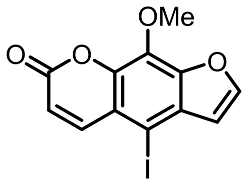

According to the procedure A, **6g-I** was obtained as a white solid (147.1 mg, yield: 86%). mp: 188-190 °C. ^{1}H NMR (400 MHz, $CDCl_3$) δ 7.99 (d, *J* = 9.9 Hz, 1H), 7.73 (d, *J* = 2.0 Hz, 1H), 6.77 (d, *J* = 2.0 Hz, 1H), 6.40 (d, *J* = 9.9 Hz, 1H), 4.28 (s, 3H). ^{13}C NMR (101 MHz, $CDCl_3$) δ 160.0, 147.1, 146.6, 145.5, 143.4, 133.2, 132.5, 118.4, 116.2, 110.7, 79.8, 61.4. HRMS (ESI) calcd for $C_{12}H_8IO_4^+$ [M + H^+] 342.9462, found 342.9463.

1-(3-Chloro-4-methoxybenzoyl)pyrrolidin-2-one (6h-Cl)

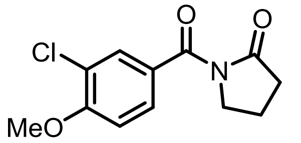

According to the procedure A, **6h-Cl** was gained as a white solid (96.3 mg, yield: 76%). mp: 128-130 °C. ^{1}H NMR (400 MHz, $CDCl_3$) δ 7.69 (s, 1H), 7.57 (s, 1H), 6.93 (s, 1H), 3.95 (s, 5H), 2.61 (s, 2H), 2.14 (s, 2H). ^{13}C NMR (101 MHz, $CDCl_3$) δ 174.7, 168.9, 158.1, 131.7, 129.9, 127.0, 121.9, 110.7, 56.3, 46.8, 33.4, 17.7. HRMS (ESI) calcd for $C_{12}H_{13}{}^{35}ClNO_3^+$ [M + H^+] 254.0578, found 254.0579.

1-(3-Bromo-4-methoxybenzoyl)pyrrolidin-2-one (6h-Br)

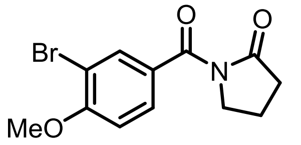

According to the procedure A, **6h-Br** was obtained as a white solid (132.7 mg, yield: 89%). mp: 140-142 °C. ^{1}H NMR (400 MHz, $CDCl_3$) δ 7.86 (d, *J* = 2.1 Hz, 1H), 7.60 (dd, *J* = 8.6, 2.1 Hz, 1H), 6.89 (d, *J* = 8.6 Hz, 1H), 3.94 (s, 3H), 3.91 (d, *J* = 7.1 Hz, 2H), 2.61 (t, *J* = 8.0 Hz, 2H), 2.20 – 2.08 (m, 2H). ^{13}C NMR (101 MHz, $CDCl_3$) δ 174.7, 168.8, 158.9, 134.8, 130.6, 127.4, 110.9, 110.5, 56.4, 46.8, 33.4, 17.7. HRMS (ESI) calcd for $C_{12}H_{13}{}^{79}BrNO_3^+$ [M + H^+] 298.0073, found 298.0078.

1-(3-Iodo-4-methoxybenzoyl)pyrrolidin-2-one (6h-I)

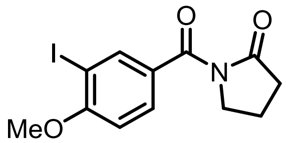

According to the procedure A, **6h-I** was gained as a white solid (141.5 mg, yield: 82%). mp: 151-153 °C. ^{1}H NMR (400 MHz, $CDCl_3$) δ 8.07 (d, *J* = 1.4 Hz, 1H), 7.70 – 7.58 (m, 1H), 6.80 (d, *J* = 8.5 Hz, 1H), 3.91

(d, J = 10.1 Hz, 5H), 2.60 (t, J = 7.9 Hz, 2H), 2.19 – 2.04 (m, 2H). ^{13}C NMR (101 MHz, $CDCl_3$) δ 174.6, 168.5, 161.1, 140.9, 131.6, 128.0, 109.4, 84.8, 56.6, 46.8, 33.4, 17.7. HRMS (ESI) calcd for $C_{12}H_{13}INO_3^+$ [M + H$^+$] 345.9935, found 345.9938.

5-((4-Chloro-3,5-dimethylphenoxy)methyl)oxazolidin-2-one (6i-Cl)

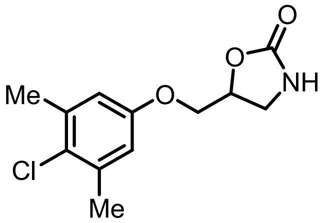

According to the procedure A, **6i-Cl** was obtained as a white solid (97.2 mg, yield: 76%). mp: 145-147 °C. ^1H NMR (400 MHz, DMSO) δ 7.60 (s, 1H), 6.83 (s, 2H), 4.89 (s, 1H), 4.26 – 3.97 (m, 2H), 3.61 (t, J = 8.5 Hz, 1H), 3.32 (d, J = 7.4 Hz, 1H), 2.29 (s, 6H). ^{13}C NMR (101 MHz, DMSO) δ 159.1, 156.7, 137.1, 125.9, 115.3, 73.9, 69.2, 41.9, 20.9. HRMS (ESI) calcd for $C_{12}H_{15}{}^{35}ClNO_3^+$ [M + H$^+$] 256.0735, found 256.0739.

5-((4-Bromo-3,5-dimethylphenoxy)methyl)oxazolidin-2-one (6i-Br)

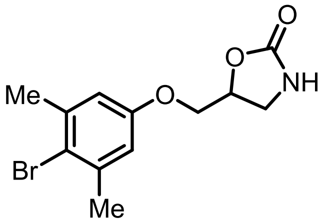

According to the procedure A, **6i-Br** was gained as a white solid (135.1 mg, yield: 90%). mp: 156-158 °C. ^1H NMR (400 MHz, DMSO) δ 7.58 (s, 1H), 6.84 (s, 2H), 4.88 (d, J = 2.7 Hz, 1H), 4.20 – 3.99 (m, 2H), 3.60 (t, J = 8.8 Hz, 1H), 3.29 (d, J = 7.9 Hz, 1H), 2.32 (s, 6H). ^{13}C NMR (101 MHz, DMSO) δ 159.0, 157.3, 139.1, 118.3, 115.3, 73.9, 69.2, 42.0, 23.9. HRMS (ESI) calcd for $C_{12}H_{15}{}^{79}BrNO_3^+$ [M + H$^+$] 300.0230, found 300.0235.

5-((4-Iodo-3,5-dimethylphenoxy)methyl)oxazolidin-2-one (6i-I)

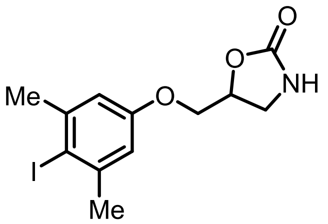

According to the procedure A, **6i-I** was obtained as a white solid (152.8 mg, yield: 88%). mp: 149-151 °C. ^1H NMR (400 MHz, DMSO) δ 7.58 (s, 1H), 6.84 (s, 2H), 4.88 (ddd, J = 9.5, 7.8, 4.8 Hz, 1H), 4.18 –

4.04 (m, 2H), 3.60 (t, J = 8.9 Hz, 1H), 3.29 (d, J = 8.3 Hz, 1H), 2.37 (s, 6H). ^{13}C NMR (101 MHz, DMSO) δ 159.0, 158.3, 142.8, 114.3, 97.9, 73.9, 69.1, 41.9, 29.5. HRMS (ESI) calcd for $C_{12}H_{15}INO_3^+$ [M + H$^+$] 348.0091, found 348.0093.

Isopropyl 2-(2-bromo-4-(4-chlorobenzoyl)phenoxy)-2-methylpropanoate (6j-Br)

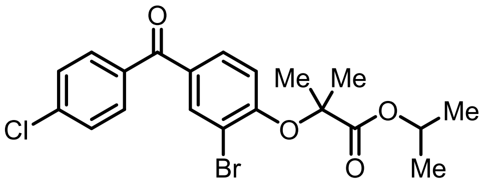

According to the procedure A, **6j-Br** was gained as a white solid (186.9 mg, yield: 85%). mp: 60-62 °C. ^{1}H NMR (400 MHz, $CDCl_3$) δ 8.03 (d, J = 2.1 Hz, 1H), 7.70 (d, J = 8.5 Hz, 2H), 7.63 (dd, J = 8.6, 2.1 Hz, 1H), 7.47 (d, J = 8.5 Hz, 2H), 6.82 (d, J = 8.6 Hz, 1H), 5.10 (dq, J = 12.5, 6.3 Hz, 1H), 1.69 (s, 6H), 1.22 (d, J = 6.3 Hz, 6H). ^{13}C NMR (101 MHz, $CDCl_3$) δ 193.0, 172.7, 156.4, 138.8, 135.8, 135.8, 131.6, 131.5, 130.2, 128.7, 116.6, 114.9, 81.2, 69.5, 25.3, 21.5. HRMS (ESI) calcd for $C_{20}H_{21}{}^{79}Br^{35}ClO_4^+$ [M + H$^+$] 439.0306, found 439.0310.

Ethyl 2-(4-chlorophenoxy)-2-methylpropanoate (6k)

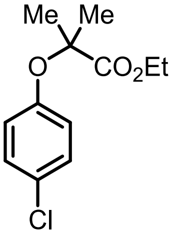

According to the procedure A, **6k** was gained as colorless oil (99.5 mg, yield: 82%). ^{1}H NMR (400 MHz, $CDCl_3$) δ 7.21 – 7.15 (m, 2H), 6.81 – 6.74 (m, 1H), 4.22 (q, J = 7.1 Hz, 1H), 1.57 (s, 3H), 1.24 (t, J = 7.1 Hz, 2H). ^{13}C NMR (101 MHz, $CDCl_3$) δ 174.0, 154.1, 129.1, 127.2, 120.5, 79.5, 61.5, 25.3, 14.1. HRMS (ESI) calcd for $C_{12}H_{16}{}^{35}ClO_3^+$ [M + H$^+$] 243.0782, found 243.0786.

Ethyl 2-(2-bromo-4-chlorophenoxy)-2-methylpropanoate (6k-Br)

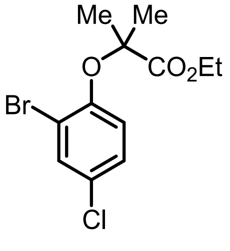

According to the procedure A, **6k-Br** was obtained as colorless oil (141.5 mg, yield: 88%). ^{1}H NMR (400 MHz, $CDCl_3$) δ 7.54 (d, J = 2.6 Hz, 1H), 7.15 (dd, J = 8.8, 2.6 Hz, 1H), 6.82 (d, J = 8.8 Hz, 1H), 4.25 (q, J = 7.1 Hz, 2H), 1.61 (s, 6H), 1.27 (t, J = 7.1 Hz, 3H). ^{13}C NMR (101 MHz, $CDCl_3$) δ 173.7, 151.5, 133.0,

127.9, 120.1, 116.8, 81.2, 61.7, 25.1, 14.1. HRMS (ESI) calcd for $C_{12}H_{15}{}^{79}Br^{35}ClO_3^+$ [M + H$^+$] 320.9888, found 320.9887.

Ethyl 2-(4-chloro-2-iodophenoxy)-2-methylpropanoate (6k-I)

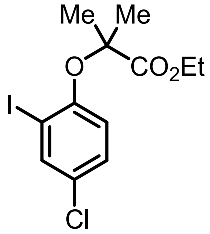

According to the procedure A, **6k-I** was gained as colorless oil (149.2 mg, yield: 81%). ^{1}H NMR (400 MHz, CDCl$_3$) δ 7.74 (d, *J* = 2.6 Hz, 1H), 7.17 (dd, *J* = 8.8, 2.6 Hz, 1H), 6.69 (d, *J* = 8.8 Hz, 1H), 4.24 (q, *J* = 7.1 Hz, 2H), 1.62 (s, 6H), 1.25 (t, *J* = 7.1 Hz, 3H). ^{13}C NMR (101 MHz, CDCl$_3$) δ 173.7, 154.0, 138.7, 128.8, 127.9, 118.1, 91.4, 81.3, 61.7, 25.3, 14.1. HRMS (ESI) calcd for $C_{12}H_{15}{}^{35}ClIO_3^+$ [M + H$^+$] 368.9749, found 368.9753.

2-((1-(4-(4-Bromophenoxy)phenoxy)propan-2-yl)oxy)pyridine (6l-Br)

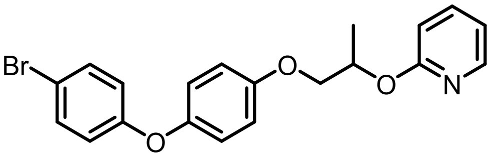

According to the procedure A, **6l-Br** was gained as a white solid (176.1 mg, yield: 88%). mp: 56-58 °C. ^{1}H NMR (400 MHz, CDCl$_3$) δ 8.15 (s, 1H), 7.58 (s, 1H), 7.37 (d, *J* = 6.7 Hz, 2H), 6.93 (s, 4H), 6.89 – 6.68 (m, 4H), 5.59 (s, 1H), 4.18 (s, 1H), 4.08 (s, 1H), 1.48 (d, *J* = 4.0 Hz, 3H). ^{13}C NMR (101 MHz, CDCl$_3$) δ 163.1, 157.8, 155.5, 149.8, 146.6, 138.9, 132.5, 120.9, 119.2, 116.8, 115.9, 114.7, 111.7, 71.1, 69.4, 17.0. HRMS (ESI) calcd for $C_{20}H_{19}{}^{79}BrNO_3^+$ [M + H$^+$] 400.0543, found 400.0547.

2-((4-Chloronaphthalen-1-yl)oxy)-*N*,*N*-diethylpropanamide (6m-Cl)

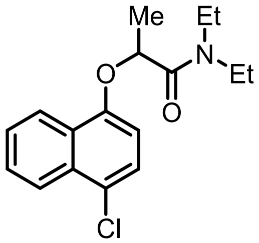

According to the procedure A, **6m-Cl** was obtained as a white solid (123.8 mg, yield: 81%). mp: 78-80 °C. ^{1}H NMR (400 MHz, CDCl$_3$) δ 8.33 (d, *J* = 8.3 Hz, 1H), 8.19 (d, *J* = 8.4 Hz, 1H), 7.60 (t, *J* = 7.2 Hz, 1H), 7.53 (t, *J* = 7.6 Hz, 1H), 7.40 (d, *J* = 8.3 Hz, 1H), 6.72 (d, *J* = 8.3 Hz, 1H), 5.08 (q, *J* = 6.7 Hz, 1H), 3.53 (dq, *J* = 14.2, 7.1 Hz, 1H), 3.46 – 3.28 (m, 3H), 1.72 (d, *J* = 6.7 Hz, 3H), 1.10 (t, *J* = 7.1 Hz, 3H), 1.00 (t, *J* = 7.1 Hz, 3H). ^{13}C NMR (101 MHz, CDCl$_3$) δ 169.9, 152.4, 131.5, 127.6, 126.8, 126.1, 125.7, 124.3, 124.0, 122.5,

105.8, 74.6, 41.1, 40.4, 18.0, 14.2, 12.6. HRMS (ESI) calcd for $C_{17}H_{21}{}^{35}ClNO_2^+$ [M + H$^+$] 306.1255, found 306.1257.

2-((4-Bromonaphthalen-1-yl)oxy)-*N*,*N*-diethylpropanamide (6m-Br)

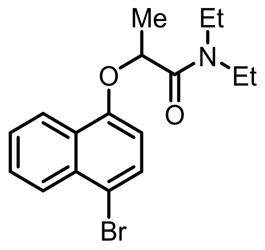

According to the procedure A, **6m-Br** was obtained as a white solid (161.1 mg, yield: 92%). mp: 79-81 °C. ^{1}H NMR (400 MHz, $CDCl_3$) δ 8.32 (d, *J* = 8.2 Hz, 1H), 8.16 (d, *J* = 8.4 Hz, 1H), 7.66 – 7.58 (m, 2H), 7.57 – 7.49 (m, 1H), 6.69 (d, *J* = 8.3 Hz, 1H), 5.09 (q, *J* = 6.7 Hz, 1H), 3.55 (dq, *J* = 14.2, 7.1 Hz, 1H), 3.47 – 3.32 (m, 3H), 1.73 (d, *J* = 6.7 Hz, 3H), 1.10 (t, *J* = 7.1 Hz, 3H), 1.00 (t, *J* = 7.1 Hz, 3H). ^{13}C NMR (101 MHz, $CDCl_3$) δ 169.9, 153.1, 132.6, 129.4, 127.9, 126.9, 126.2, 122.5, 114.1, 106.5, 74.6, 41.1, 40.4, 18.0, 14.2, 12.6. HRMS (ESI) calcd for $C_{17}H_{21}{}^{79}BrNO_2^+$ [M + H$^+$] 350.0750, found 350.0754.

***N*, *N*-Diethyl-2-((4-iodonaphthalen-1-yl)oxy)propanamide (6m-I)**

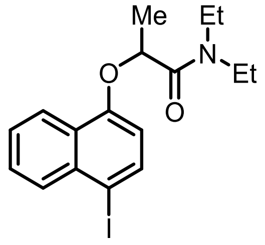

According to the procedure A, **6m-I** was gained as a white solid (174.8 mg, yield: 88%). mp: 80-82 °C. ^{1}H NMR (400 MHz, $CDCl_3$) δ 8.28 (d, *J* = 8.1 Hz, 1H), 8.01 (d, *J* = 8.3 Hz, 1H), 7.90 (d, *J* = 8.2 Hz, 1H), 7.62 – 7.55 (m, 1H), 7.52 (dd, *J* = 11.2, 3.9 Hz, 1H), 6.59 (d, *J* = 8.2 Hz, 1H), 5.08 (q, *J* = 6.7 Hz, 1H), 3.53 (m, 1H), 3.46 – 3.28 (m, 3H), 1.72 (d, *J* = 6.7 Hz, 3H), 1.10 (t, *J* = 7.1 Hz, 3H), 1.01 (t, *J* = 7.1 Hz, 3H). ^{13}C NMR (101 MHz, $CDCl_3$) δ 169.8, 154.1, 136.8, 135.0, 131.9, 128.3, 126.8, 126.2, 122.6, 107.4, 89.2, 74.5, 41.1, 40.4, 18.0, 14.2, 12.7. HRMS (ESI) calcd for $C_{17}H_{21}INO_2^+$ [M + H$^+$] 398.0611, found 398.0612.

(8*R*,9*S*,13*S*,14*S*)-2-Bromo-3-methoxy-13-methyl-6,7,8,9,11,12,13,14,15,16-decahydro-17*H*-cyclopenta[a]phenanthren-17-one (6n-Br)

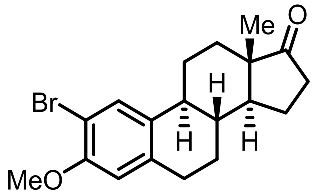

According to the procedure A, **6n-Br** was obtained as a white solid (145.3 mg, yield: 80%). mp: 194-196 °C. ^{1}H NMR (400 MHz, $CDCl_3$) δ 7.41 (s, 1H), 6.62 (s, 1H), 3.84 (d, *J* = 2.7 Hz, 3H), 2.98 – 2.74 (m, 2H), 2.56 – 2.43 (m, 1H), 2.38 – 2.29 (m, 1H), 2.25 – 2.10 (m, 2H), 2.09 – 1.98 (m, 2H), 1.97 – 1.88 (m, 1H), 1.67 – 1.58 (m, 1H), 1.54 (d, *J* = 11.1 Hz, 1H), 1.52 – 1.36 (m, 4H), 0.89 (d, *J* = 2.4 Hz, 3H). ^{13}C NMR (101 MHz, $CDCl_3$) δ 153.7, 137.0, 133.6, 130.2, 112.3, 108.7, 56.2, 50.3, 47.9, 43.7, 38.1, 35.9, 31.5, 29.5, 26.4, 25.9, 21.6, 13.9. HRMS (ESI) calcd for $C_{19}H_{24}{}^{79}BrO_2^+$ [M + H$^+$] 363.0954, found 363.0956.

Ethyl (*S*)-3-(3-bromo-4-hydroxyphenyl)-2-((2-oxo-2-phenylethyl)amino)propano-ate (6o-Br)

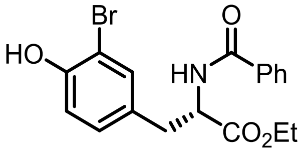

According to the procedure A, **6o-Br** was gained as a white solid (174.6 mg, yield: 86%). mp: 106-108 °C. ^{1}H NMR (400 MHz, $CDCl_3$) δ 7.75 (d, *J* = 7.3 Hz, 2H), 7.50 (t, *J* = 6.9 Hz, 1H), 7.42 (t, *J* = 7.2 Hz, 2H), 7.26 (s, 1H), 6.97 (d, *J* = 7.7 Hz, 1H), 6.88 (d, *J* = 8.1 Hz, 1H), 6.77 (d, *J* = 6.7 Hz, 1H), 6.36 (s, 1H), 5.01 (d, *J* = 6.0 Hz, 1H), 4.22 (d, *J* = 6.7 Hz, 2H), 3.16 (m, 2H), 1.29 (t, *J* = 7.0 Hz, 3H). ^{13}C NMR (101 MHz, $CDCl_3$) δ 171.6, 167.2, 151.8, 133.7, 133.1, 132.0, 130.0, 129.3, 128.7, 127.1, 116.3, 110.1, 61.9, 53.7, 36.8, 14.2. HRMS (ESI) calcd for $C_{19}H_{21}{}^{79}BrNO_4^+$ [M + H$^+$] 406.0648, found 406.0649.

Ethyl (*S*)-3-(4-hydroxy-3-iodophenyl)-2-((2-oxo-2-phenylethyl)amino)propano-ate (6o-I)

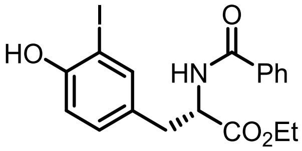

According to the procedure A, **6o-I** was gained as a white solid (165.4 mg, yield: 73%). mp: 60-62 °C. ^{1}H NMR (400 MHz, $CDCl_3$) δ 7.78 – 7.70 (m, 2H), 7.50 (t, *J* = 7.4 Hz, 1H), 7.46 (d, *J* = 2.0 Hz, 1H), 7.41 (t, *J* = 7.5 Hz, 2H), 6.98 (dd, *J* = 8.3, 2.0 Hz, 1H), 6.86 – 6.81 (m, 1H), 6.78 (d, *J* = 7.5 Hz, 1H), 6.65 (s, 1H), 5.05 – 4.93 (m, 1H), 4.31 – 4.14 (m, 2H), 3.13 (m, 2H), 1.34 – 1.24 (m, 3H). ^{13}C NMR (101 MHz, $CDCl_3$) δ 171.6, 167.3, 154.6, 139.4, 133.7, 132.0, 130.9, 129.6, 128.8, 127.1, 115.2, 85.1, 62.0, 53.8, 36.6, 14.3. HRMS (ESI) calcd for $C_{19}H_{21}INO_4^+$ [M + H$^+$] 454.0510, found 454.0515.

3-Chloro-4-hydroxy-5-methoxybenzaldehyde (6p-Cl)

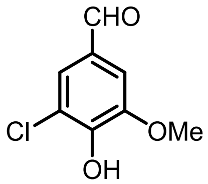

According to the procedure A, **6p-Cl** was obtained as a white solid (77.4 mg, yield: 83%). mp: 164-166 °C. ^{1}H NMR (400 MHz, $CDCl_3$) δ 9.79 (s, 1H), 7.50 (d, J = 1.5 Hz, 1H), 7.34 (d, J = 1.4 Hz, 1H), 6.43 (s, 1H), 3.99 (s, 3H). ^{13}C NMR (101 MHz, $CDCl_3$) δ 189.8, 147.9, 147.8, 129.4, 127.1, 119.9, 107.5, 56.7. HRMS (ESI) calcd for $C_8H_8{}^{35}ClO_3{}^+$ [M + H^+] 187.0156, found 187.0158.

3-Bromo-4-hydroxy-5-methoxybenzaldehyde (6p-Br)

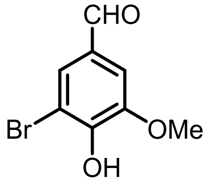

According to the procedure A, **6p-Br** was obtained as a white solid (106.3 mg, yield: 92%). mp: 167-169 °C. ^{1}H NMR (400 MHz, $CDCl_3$) δ 9.79 (s, 1H), 7.65 (d, J = 1.7 Hz, 1H), 7.37 (d, J = 1.6 Hz, 1H), 6.52 (s, 1H), 3.99 (s, 3H). ^{13}C NMR (101 MHz, $CDCl_3$) δ 189.7, 148.9, 147.7, 130.1, 130.0, 108.2, 108.0, 56.6. HRMS (ESI) calcd for $C_8H_8{}^{79}BrO_3{}^+$ [M + H^+] 230.9651, found 230.9655.

4-Hydroxy-3-iodo-5-methoxybenzaldehyde (6p-I)

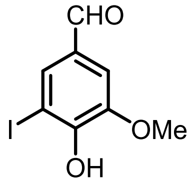

According to the procedure A, **6p-I** was obtained as a white solid (120.9 mg, yield: 87%). mp: 181-183 °C. ^{1}H NMR (400 MHz, $CDCl_3$) δ 9.77 (s, 1H), 7.82 (d, J = 1.6 Hz, 1H), 7.38 (d, J = 1.5 Hz, 1H), 6.68 (s, 1H), 3.97 (s, 3H). ^{13}C NMR (101 MHz, $CDCl_3$) δ 189.6, 151.4, 146.5, 136.2, 131.1, 108.6, 80.5, 56.6. HRMS (ESI) calcd for $C_8H_8IO_3{}^+$ [M + H^+] 278.9513, found 278.9516.

1-Chlorosinomenine (6q-Cl)

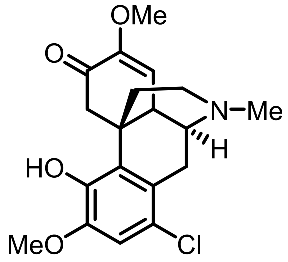

According to the procedure A, **6q-Cl** was obtained as a white solid (174.6 mg, yield: 96%). mp: 226-228

°C. ^{1}H NMR (400 MHz, $CDCl_3$) δ 6.74 (s, 1H), 6.23 (s, 1H), 5.42 (d, J = 1.9 Hz, 1H), 4.32 (d, J = 15.6 Hz, 1H), 3.79 (s, 3H), 3.48 (s, 3H), 3.35 (s, 1H), 3.15 (s, 1H), 3.05 (d, J = 19.0 Hz, 1H), 2.64 (d, J = 11.8 Hz, 1H), 2.59 – 2.43 (m, 5H), 2.16 – 2.04 (m, 1H), 2.02 – 1.90 (m, 2H). ^{13}C NMR (101 MHz, $CDCl_3$) δ 193.4, 152.5, 145.5, 143.6, 126.9, 123.8, 123.1, 114.0, 110.3, 56.4, 56.2, 54.9, 48.7, 47.1, 45.1, 42.5, 40.6, 35.3, 22.9. HRMS (ESI) calcd for $C_{19}H_{23}{}^{35}ClNO_4^+$ [M + H$^+$] 364.1310, found 364.1314.

Dimethyl 6,6'-dichloro-7,7'-dimethoxy-[4,4'-bibenzo[*d*][1,3]dioxole]-5,5'-dicarbo-xylate (6r-Cl$_2$)

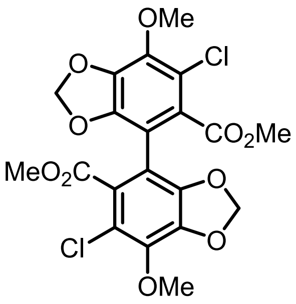

According to the procedure A, **6r-Cl$_2$** was obtained as a white solid (231.4 mg, yield: 85%). mp: 186-188 °C. ^{1}H NMR (400 MHz, $CDCl_3$) δ 6.00 (d, J = 7.7 Hz, 4H), 4.07 (s, 6H), 3.69 (s, 6H). ^{13}C NMR (101 MHz, $CDCl_3$) δ 165.7, 146.8, 139.9, 138.0, 127.6, 117.7, 107.9, 102.4, 60.3, 52.4. HRMS (ESI) calcd for $C_{20}H_{17}{}^{35}Cl_2O_{10}^+$ [M + H$^+$] 487.0193, found 487.0191.

Dimethyl 6,6'-dibhloro-7,7'-dimethoxy-[4,4'-bibenzo[*d*][1,3]dioxole]-5,5'-dicarbo-xylate (6r-Br$_2$)

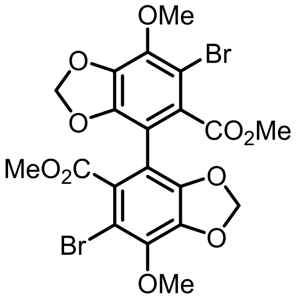

According to the procedure A, **6r-Br$_2$** was obtained as a white solid (262.1 mg, yield: 91%). mp: 228-230 °C. ^{1}H NMR (400 MHz, $CDCl_3$) δ 6.00 (dd, J = 9.2, 1.1 Hz, 4H), 4.07 (s, 6H), 3.69 (s, 6H). ^{13}C NMR (101 MHz, $CDCl_3$) δ 166.3, 147.7, 140.7, 137.6, 130.1, 108.3, 106.3, 102.4, 60.3, 52.4. HRMS (ESI) calcd for $C_{20}H_{17}{}^{79}Br_2O_{10}^+$ [M + H$^+$] 574.9183, found 574.9180.

Dimethyl 6-iodo-7,7'-dimethoxy-[4,4'-bibenzo[*d*][1,3]dioxole]-5,5'-dicarbo-xylate (6r-I)

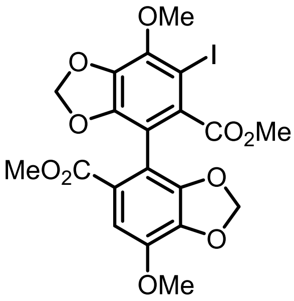

According to the procedure A, **6r-I** was obtained as a white solid (234.0 mg, yield: 86%). mp: 179-181 °C. ^{1}H NMR (400 MHz, $CDCl_3$) δ 7.30 (s, 1H), 6.03 (s, 1H), 5.99 (d, J = 3.8 Hz, 3H), 4.04 (s, 3H), 3.94 (s, 3H), 3.68 (s, 3H), 3.59 (s, 3H). ^{13}C NMR (101 MHz, $CDCl_3$) δ 167.9, 166.0, 148.1, 147.9, 143.0, 141.9, 138.1, 136.3, 134.2, 124.3, 111.2, 111.1, 109.4, 102.6, 102.1, 79.8, 60.2, 56.6, 52.4, 52.2. HRMS (ESI) calcd for $C_{20}H_{18}IO_{10}^{+}$ [M + H^{+}] 544.9939, found 544.9942.

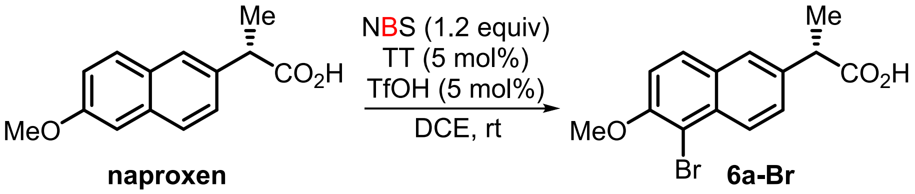

Gram-scale synthesis of product 6a-Br: NBS (18.55 g, 104.23 mmol, 1.20 equiv,) and thianthracene (939.43 mg, 4.34 mmol, 0.05 equiv) were dissolved in DCE (20 mL). With stirring of the mixture, trifluoromethanesulfonic acid (TfOH) (651.74 mg, 4.34 mmol, 0.05 equiv) was added. Then substrate naproxen (20.0 g, 86.86 mmol, 1.0 equiv) was added and the mixture was stirred at rt till the reaction was completed. The solvent of reaction mixture was removed by rotary evaporation. The residue was purified by silica gel column chromatography to afford product **6a-Br** as a white solid (25.0 g, yield: 93%).

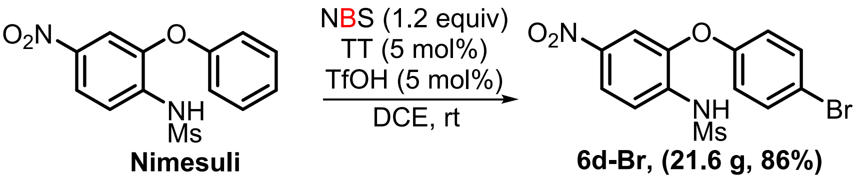

Gram-scale synthesis of product 6d-Br: NBS (1.39 g, 7.78 mmol, 1.20 equiv,) and thianthracene (70.2 mg, 0.324 mmol, 0.05 equiv) were dissolved in DCE (100 mL). With stirring of the mixture, trifluoromethanesulfonic acid (TfOH) (48.7 mg, 0.324 mmol, 0.05 equiv) was added. Then substrate nimesuli (2.0 g, 6.49 mmol, 1.0 equiv) was added and the mixture was stirred at rt till the reaction was completed. The solvent of reaction mixture was removed by rotary evaporation. The residue was purified by silica gel column chromatography to afford product **6d-Br** as a yellow solid (2.16 g, yield: 86%).

Procedure for synthesis of methyl (*E*)-3-chloro-4,6-dimethoxy-2-styrylbenzoate (7)

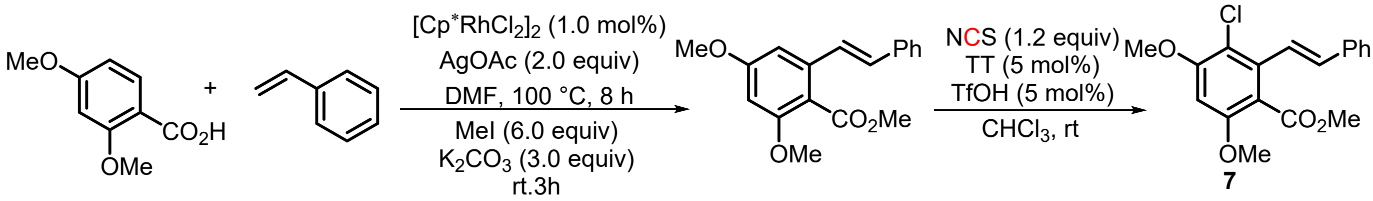

To a 25 mL two-necked flask with a reflux condenser, an argon balloon was added 2,4-dimethoxybenzoic acid (182.0 mg, 1 mmol), styrene (208.0 mg, 2.0 mmol), [(Cp*$RhCl_2$)] (6.0 mg, 0.01 mmol), AgOAc (332.4 mg, 2.0 mmol), and DMF (6 mL). Then, the resulting mixture was stirred under argon at 100 °C for 10 h. After cooling down to room temperature, MeI (846.0 mg, 6.0 mmol,) and K_2CO_3 (414.0 mg, 3 mmol) were added and the resulting mixture was stirred under air at room temperature for 3 h. The reaction was monitored by TLC and till the reaction was completed. The reaction mixture was then extracted with EtOAc (3 x 20 mL) and water (60 mL). The combined organic extracts were dried over anhydrous Na_2SO_4 and the solvent was removed by rotary evaporation. The residue was purified by silica gel column chromatography (EtOAc/PE = 10/90) to afford methyl (*E*)-2,4-dimethoxy-6-styrylbenzoate as a white solid (226.9 mg, 76%).[4]

NCS (80.1 mg, 0.6 mmol, 1.20 equiv,) and thianthracene (5.41 mg, 0.025 mmol, 0.05 equiv) were dissolved in DCE (3.0 mL). With stirring of the mixture, trifluoromethanesulfonic acid (TfOH) (3.75mg, 0.025mmol, 0.05 equiv) was added. Then substrate methyl (*E*)-2,4-dimethoxy-6-styrylbenzoate (149.17 mg, 0.5 mmol, 1.0 equiv) was added and the mixture was stirred at rt till the reaction was completed. The solvent of reaction mixture was removed by rotary evaporation. The residue was purified by silica gel column chromatography to afford product **7** as yellow oil (158.1 mg, yield: 95%).^{1}H NMR (400 MHz, $CDCl_3$) δ 7.48 (s, 2H), 7.35 (s, 2H), 7.29 (s, 1H), 7.22 (d, *J* = 17.6 Hz, 1H), 6.79 (d, *J* = 15.4 Hz, 1H), 6.48 (s, 1H), 3.94 (s, 3H), 3.87 (s, 3H), 3.78 (s, 3H). ^{13}C NMR (101 MHz, $CDCl_3$) δ 167.9, 156.6, 156.1, 136.8, 136.2, 134.9, 128.7, 128.3, 126.8, 124.2, 116.3, 114.0, 95.4, 56.5, 56.4, 52.5. HRMS (ESI) calcd for $C_{18}H_{18}{}^{35}ClO_4^+$ [M + H$^+$] 333.0888, found 333.0892.

III. Mechanism Study

The relationship between yield and time.

NBS (106.8 mg, 0.6 mmol, 1.20 equiv,) and thianthracene (5.41 mg, 0.025 mmol, 0.05 equiv) were dissolved in $CDCl_3$ (3.0 mL). With stirring of the mixture, trifluoromethanesulfonic acid (TfOH) (3.75 mg, 0.025 mmol, 0.05 equiv) was added. Then 1,3-dichloro-4-methoxybenzene (**1q)** (88.51 mg, 0.5 mmol) was added to the mixture and stirred at rt for 3.75 h. Tetrachloroethane (0.5 mmol) was added as an internal standard. We found that the signal of succinimide was recognized after 10 minutes later (2.70 ppm). The

increased signals (**1q-Cl**, at 3.80 ppm) and decreased signals (**1q**, at 6.78 ppm and 3.81 ppm) were detected during the 3.75 h reaction time.

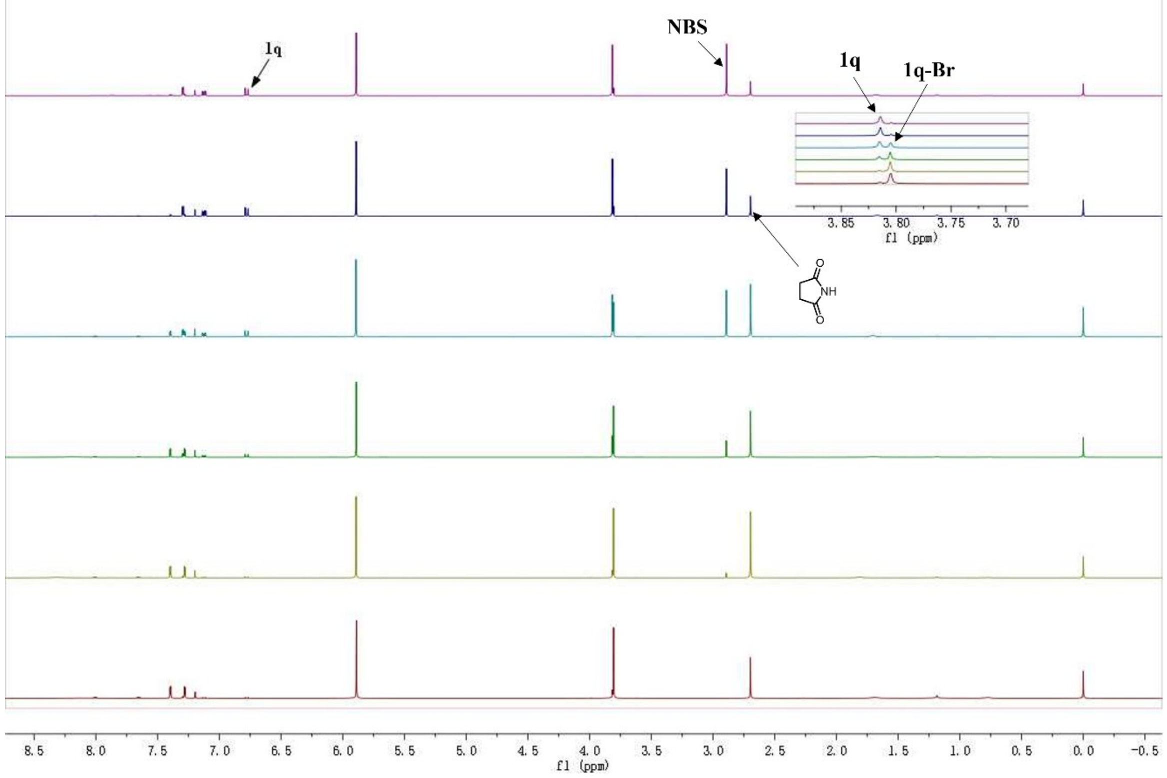

Figure S1. The NMR spectrum of relationship between yield and time

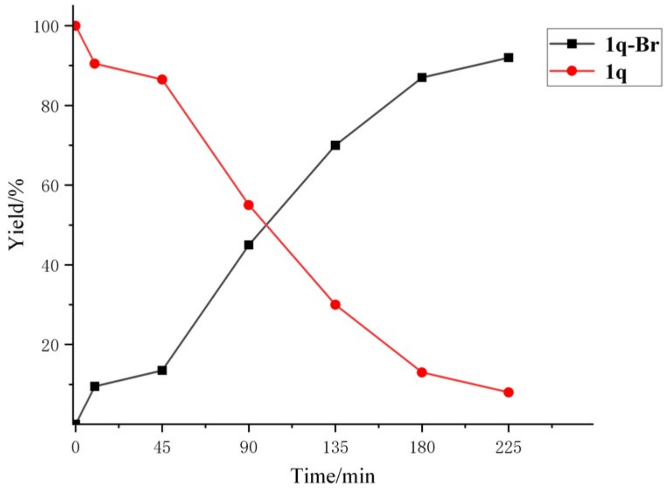

Figure S2. The relationship between yield and time

The Competitive reaction and Hammett plot

Competition experiments between anisole and *othro*-substituted anisoles: NCS (80.1 mg, 0.6 mmol, 1.20 equiv,) and thianthracene (5.41 mg, 0.025 mmol, 0.05 equiv) were dissolved in $CDCl_3$ (3.0 mL). With stirring of the mixture, trifluoromethanesulfonic acid (TfOH) (3.75 mg, 0.025 mmol, 0.05 equiv) was added. Then anisole (0.25 mmol) and *othro*-substituted anisole (0.25 mmol) were added to the mixture and stirred at rt. The yield of the chlorinated product was determined by ^{1}H NMR spectroscopy with 1,1,2,2-tetrachloroethane as an internal standard. The Hammett value is 0.12 for *m*-OMe, 0.35 for *m*-CHO, 0.37 for *m*-Br, 0.56 for *m*-CN, and 0.71 for *m*-NO_2. It is worth mentioning that the yield of *ortho*-dimethoxy should be halved because of its symmetrical structure.

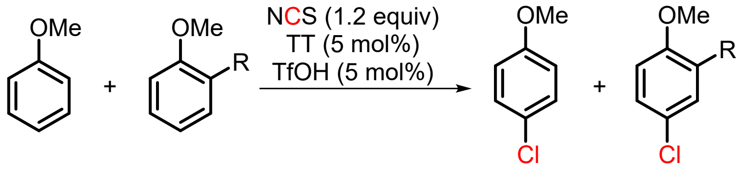

Table S1. Competition experiments

Entry	FG	time	Yield/ %		$\log(k_{FG}/k_H)$
			FG	H	
1	OMe	10min	62	33	0.274
2	CHO	15min	28.5	45.9	-0.207
3	Br	15min	27.5	51.7	-0.274
4	CN	15min	6.1	38.6	-0.801
5	NO_2	15min	2.4	30.9	-1.11

Table S2. Hammett Plot of catalytic chlorination of anisoles using the Hammett Constant σ_p

Entry	FG	σ_p	$\log(k_{FG}/k_H)$
1	-OMe	0.12	0.274
2	-CHO	0.35	-0.207
3	-Br	0.37	-0.274
4	-CN	0.56	-0.801
5	$-NO_2$	0.71	-1.11

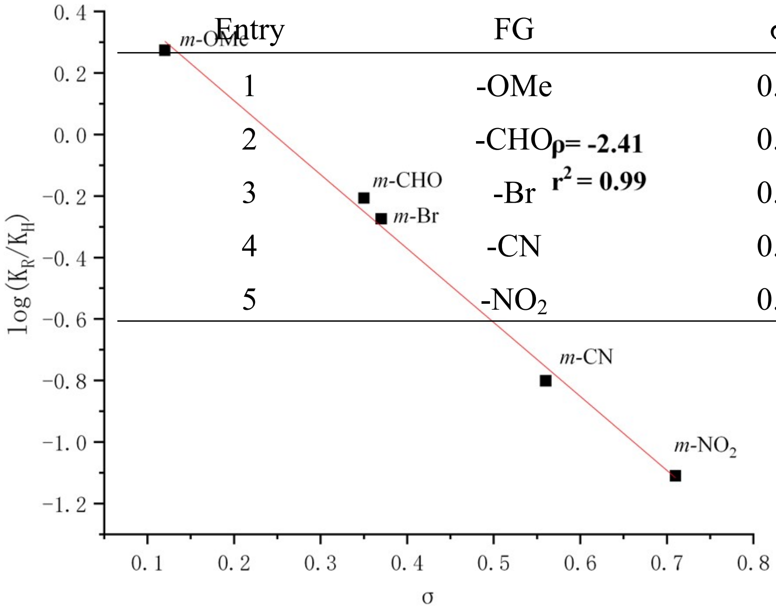

Figure S3. Hammett Plot of catalytic chlorination of anisole and othro-substituted anisoles using the

Hammett Constant σ_p

NMR studies of reactive halogenated thianthrenium salts

The spectra were recorded on a 400 MHz spectrometer at 25 °C. Each component (0.125 mmol) was mixed in $CDCl_3$ (1.0 mL) for 10 min. Chemical shifts values are given in ppm and referred as the internal standard to TMS: 0.00 ppm.

To clearly understand the mechanism of the catalytic halogenation process, the reaction progress was monitored by ^{1}H-NMR (Figure 2A). Each component was mixed in $CDCl_3$ (1.0 mL) for 10 min before the analysis. When substrate **1w** was solely treated with NCS, no chlorinated product was observed. However, upon mixing TT, TfOH with NCS in $CDCl_3$, a new proton signal at 8.21 ppm appeared and the proton signal at 7.48 ppm (H^a-TT) disappeared. We tentatively proposed that this result might be caused by the conversion of TT to the reactive intermediate **I**. Next, we investigated chemical shift changes after adding substrate **1w** to the above mixture of TT, TfOH and NCS. The ^{1}H-NMR spectrum evidenced the regeneration of TT (H^a-TT) and formation of product **1w-Cl** (H^d-1w), which might suggest that it was the reactive intermediate **I** that realized the chlorination of **1w**. One might also think that NCS could be used as an oxidant and TT might be oxidized by NCS in air to form TTSO. To rule out the possibility that the new chemical shift was that of TTSO, we treated TT with solely NCS in $CDCl_3$. The result showed that the proton signal of TTSO at 7.94 ppm (H^e-TTSO) could be detected and no chemical shift of 8.21 ppm was observed. With these regards, we tentatively concluded that the reaction of TT with NCS in the presence of TfOH gave the reactive intermediate **I**, rather than TTSO.

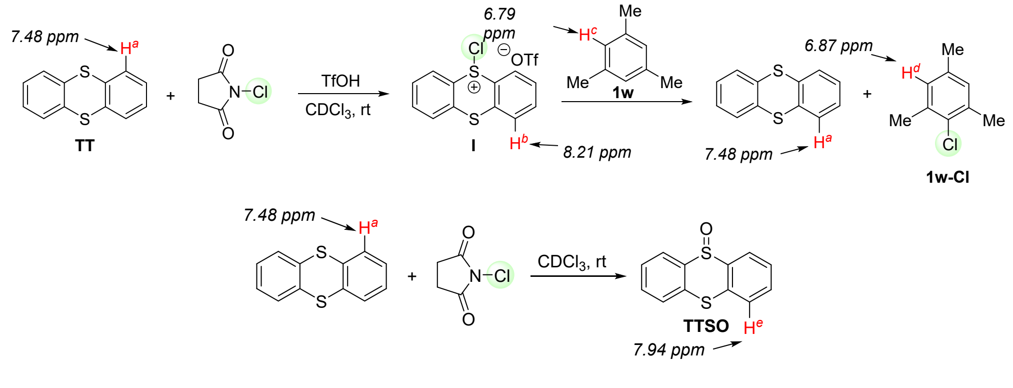

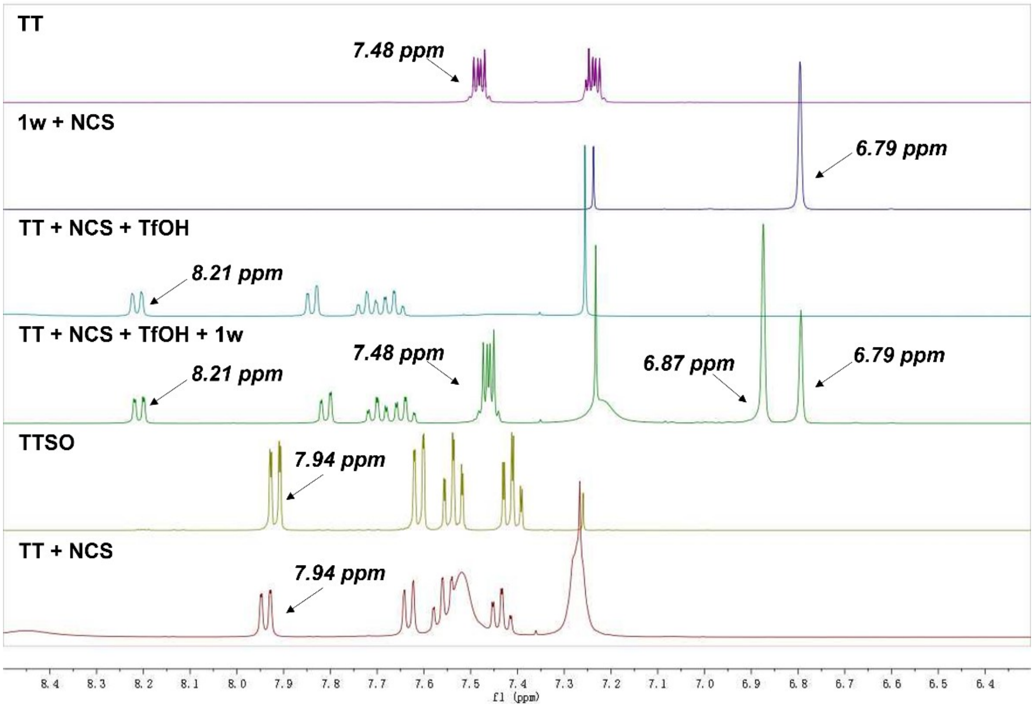

Figure S4. NMR studies of reactive halogenated thianthrenium salts

Intermediate capture

As the intermediate might be unstable at room temperature, the intermediate capture experiment was carried out at -20 °C under argon atmosphere: NCS (66.77 mg, 0.5 mmol, 1.0 equiv,) and thianthracene (108.16 mg, 0.5 mmol, 1.0 equiv) were dissolved in $CDCl_3$ (3.0 mL). With stirring of the mixture, Trifluoromethanesulfonic acid (TfOH) (74.05 mg, 0.5 mmol, 0.05 equiv) was added. The reaction mixture was allowed stirred for 10 minutes at -20 °C. Then the intermediate was detected by high resolution mass spectrometry. (ESI) calcd for $(C_{12}H_8CS_2)^+$: 250.9750, found 250.9758.

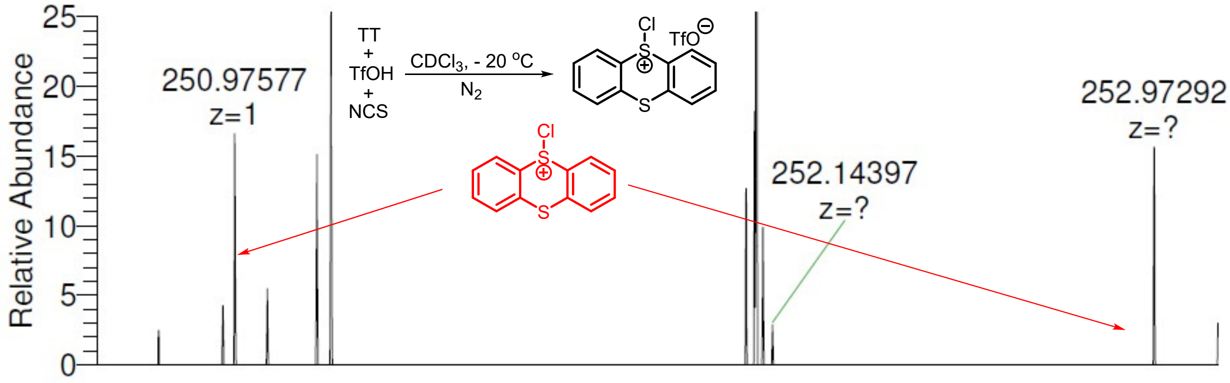

Figure S5. Intermediate capture experiment detected by HRMS

The Kinetic isotope effect (KIE) studies

NCS (80.1 mg, 0.6 mmol, 1.20 equiv,) and thianthracene (5.41 mg, 0.025 mmol, 0.05 equiv) were dissolved in $CDCl_3$ (3.0 mL). With stirring of the mixture, trifluoromethanesulfonic acid (TfOH) (3.75 mg, 0.025

mmol, 0.05 equiv) was added. Then anisole (0.25 mmol) and anisole-2,3,4,5,6-d_5 (0.25 mmol) were added to the mixture and stirred at rt. The yield was determined by ^{1}H NMR spectroscopy using1,1,2,2-tetrachloroethane as an internal standard. The KIE value of 1.09 suggested that the cleavage of a C-H bond was not involved in the rate-limiting step.

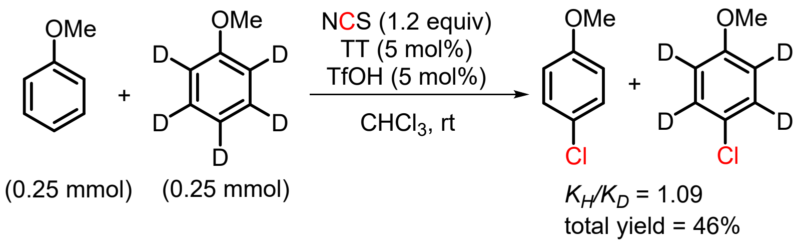

Computational Studies:

All the calculations were performed with the Gaussian 09, Revision D.01 program package.[5] Geometry optimization and frequency analysis were conducted using M06-2X function.[6] During geometry optimization we used 6-31G(d,p) basis set for all atoms. To refine the computed energy, single point calculations were performed using the M06-2X functional with the TZVP basis set for all atoms. Solvation effects were evaluated by performing single-point self-consistent reaction field calculations with the continuum model (SMD).[7] The calculated optimized structures are illustrated by CYLview7.[8]

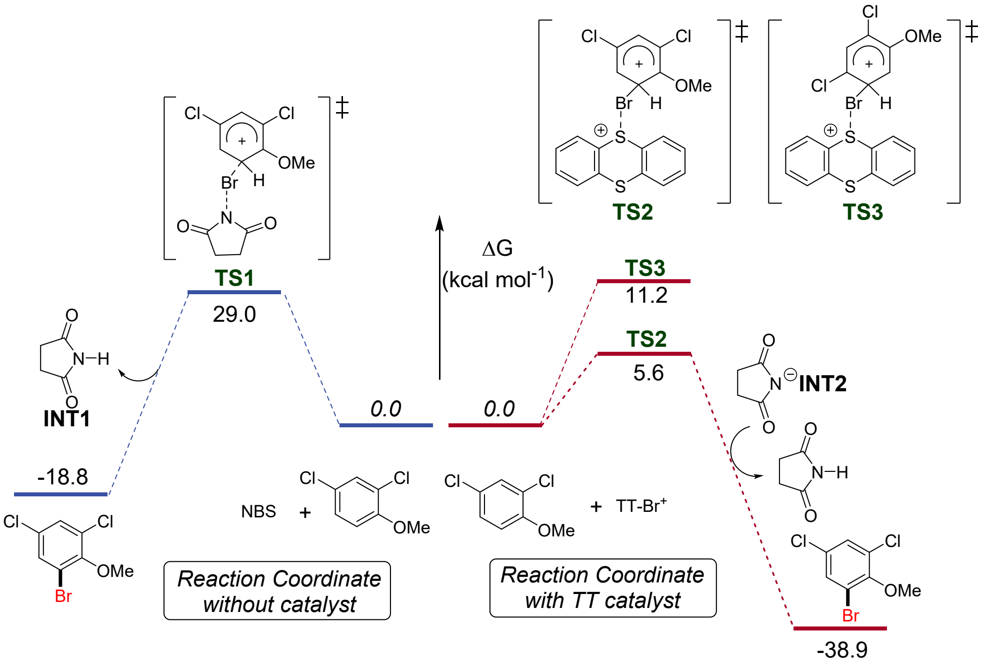

Figure S6. Computational study of the reaction mechanism.

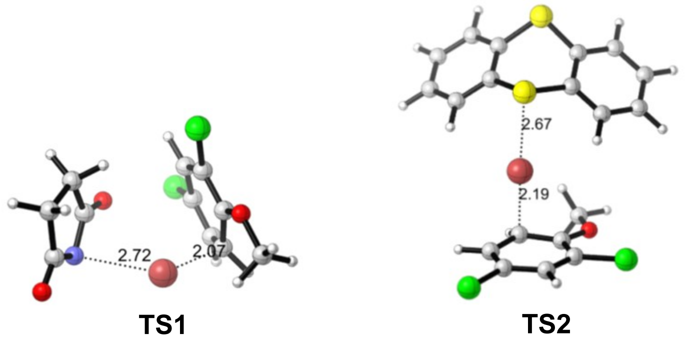

Figure S7. The key transition states in the catalytic cycle.

Table S3. The calculated energies of stationary points (in Hartree/Particle)

Structure	Hcor	Gcor	Eele	Gsol
1q	0.125833	0.080577	-1265.92	-1265.84
NBS	0.090552	0.048712	-2934.18	-2934.13
TS1	0.216024	0.148966	-4200.08	-4199.93
INT1	0.099890	0.063010	-360.614	-360.551
1q-Br	0.116702	0.066195	-3839.52	-3839.46
TT-Br^+	0.178878	0.125349	-3832.39	-3832.26
TS2	0.305536	0.225811	-5098.32	-5098.10
TS3	0.305313	0.226936	-5095.17	-5095.25
INT2	0.086268	0.049672	-360.179	-360.129
TT	0.175736	0.127397	-1258.42	-1258.29

Notes: ZPE = Zero-point correction in the gas phase; Hcor = Thermal correction to enthalpy in the gas phase; Gcor = Thermal correction to Gibbs free energy in the gas phase; Eele = The electronic energies in solvent; Gsol = Gibbs free energies in solvent.

DFT-Computed Cartesian Coordinates (unit: angstrom)

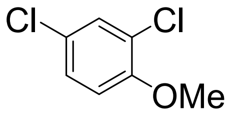

1q

C	-3.77175500	1.55195200	0.00046600
C	-2.38130600	1.59615900	0.00078200
C	-1.75540800	2.83232100	-0.00044100
C	-2.48917500	4.03047900	-0.00201300
C	-3.88375600	3.95093700	-0.00236500
C	-4.52600800	2.71544100	-0.00111700
H	-1.79124900	0.68660500	0.00198900

H	-4.48190000	4.85378000	-0.00349800
H	-5.60924900	2.66710000	-0.00133800
Cl	-0.01571500	2.90382200	0.00002500
Cl	-4.57317500	-0.00363600	0.00228800
O	-1.78584900	5.17804100	-0.00272500
C	-2.51399400	6.39890400	-0.00529000
H	-3.13851800	6.48749600	0.88991600
H	-1.76519200	7.19052800	-0.00578700
H	-3.13676500	6.48472800	-0.90199600

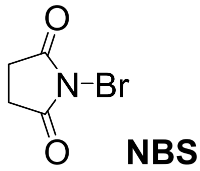

NBS

C	-2.16329500	-0.75568300	-0.01936800
C	-2.63663100	0.70075000	-0.09818500
H	-2.44119100	-1.34444200	-0.89636700
H	-2.52408500	-1.28047700	0.86753400
H	-3.23140000	1.00412800	0.76636400
H	-3.21327900	0.92141000	-0.99874700
C	-0.65065300	-0.69788900	0.04492200
C	-1.37874300	1.54560200	-0.11933300
N	-0.30251800	0.65552300	-0.02898700
Br	1.44253200	1.22397400	-0.00091800
O	-1.27087700	2.74173000	-0.19161400
O	0.13792500	-1.60180400	0.13795600

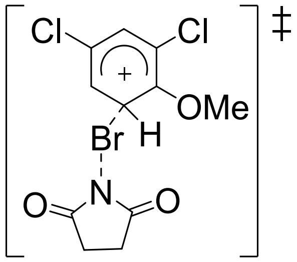

TS1

C	0.83789700	2.40145600	-0.81180500
C	1.43781800	2.53177000	0.52727600
C	-0.58070100	2.03144300	-0.87299400
C	0.77522700	1.93516800	1.62838600

C	-1.16644300	1.48952100	0.21118600
C	-0.45970400	1.37146200	1.45764000
H	-1.08421400	2.10504300	-1.83035100
C	3.15095300	-2.54574000	1.27312800
C	2.94470000	-2.51723900	-0.24919100
N	1.70144400	-2.03838900	-0.59109700
C	1.03716700	-1.68570200	0.54567300
C	1.87482200	-1.91638600	1.80919000
H	3.29702200	-3.58145800	1.59215600
O	3.80294500	-2.88236100	-1.04145800
O	-0.09378800	-1.18955200	0.59082500
H	2.03645100	-0.94587200	2.29058800
H	4.06137700	-1.99369100	1.52053100
H	1.31744200	-2.54180800	2.51110900
Br	1.75869200	0.60037600	-1.26097500
H	1.16216200	3.13524400	-1.54567300
H	-0.94453400	0.87931100	2.29395700
O	2.59276500	3.06728500	0.73102400
C	3.34355500	3.65662300	-0.35622800
H	2.79303800	4.50503800	-0.76656000
H	4.27582700	3.98655200	0.09399600
H	3.53413800	2.89527200	-1.11544500
Cl	1.57336300	1.90715800	3.16157800
Cl	-2.79375600	0.90435600	0.15952900

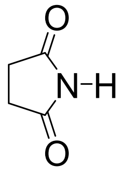

INT1

C	-2.16119300	-0.64591200	-0.51252500
C	-2.52492900	0.32910000	0.61057900
H	-2.84637800	-0.60701600	-1.36201400
H	-2.10196400	-1.68478700	-0.17997000
H	-2.69731700	-0.15905000	1.57215200
H	-3.40238600	0.93920000	0.38416500
C	-0.78252600	-0.21627000	-0.98338000
C	-1.32389600	1.24942000	0.74440900
N	-0.39441000	0.86320100	-0.20751600

O	-1.17480200	2.16068900	1.52545400
O	-0.11771200	-0.70570400	-1.86727100
H	0.49961600	1.32805700	-0.32329000

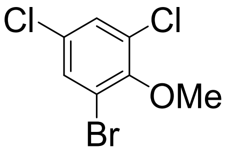

1q-Br

C	-4.01105700	1.69211500	0.00489400
C	-2.66911500	1.75210500	-0.34947600
C	-1.99056500	2.94830100	-0.15503000
C	-2.61666400	4.07559700	0.38659100
C	-3.96773500	3.96360800	0.72774800
C	-4.67802200	2.78444500	0.54536000
H	-2.16092000	0.89093600	-0.76755000
Cl	-0.30859500	3.05371900	-0.57983700
Cl	-4.88107200	0.19792800	-0.22709000
O	-1.92317800	5.21779600	0.60865100
C	-2.00269900	6.15457300	-0.47425700
H	-3.04407600	6.42772200	-0.67155500
H	-1.44638400	7.03655100	-0.15760000
H	-1.55144300	5.73671900	-1.37983600
H	-5.72378800	2.71724500	0.82198900
Br	-4.82939500	5.46188000	1.48019900

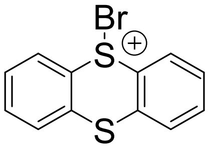

TT-Br⁺

C	-7.05376700	-2.59448000	0.52531600
C	-5.79548100	-2.65689700	-0.06861000
C	-5.24525300	-1.50781100	-0.62879100
C	-5.97950800	-0.32214700	-0.57223800
C	-7.22111400	-0.23022500	0.03805300
C	-7.75644000	-1.39375500	0.58522400
C	-3.57899500	1.08702100	-0.57344900
C	-2.90628300	-0.13463600	-0.62206200
C	-1.63884000	-0.21725000	-0.05330300
H	-1.10293300	-1.16053400	-0.06933000

C	-1.07815400	0.91239000	0.53817300
C	-1.77755600	2.11549800	0.58518100
C	-3.05151500	2.21785300	0.03114400
H	-7.48191200	-3.49207100	0.95791600
H	-5.23809200	-3.58736800	-0.09271600
H	-7.77279900	0.70150400	0.08588100
H	-8.73019400	-1.35146000	1.05980900
H	-0.08917500	0.84540600	0.97822000
H	-1.33685600	2.98722600	1.05530100
H	-3.59073600	3.15724900	0.06958700
S	-5.16213600	1.03182400	-1.43706000
S	-3.65145000	-1.53874300	-1.43154300
Br	-6.20340300	2.80258400	-0.74809000

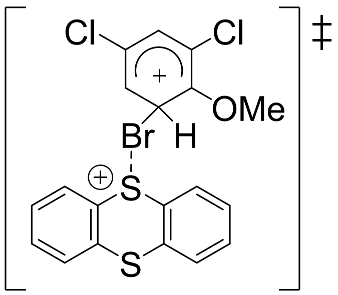

TS2

C	-2.21489400	6.07178500	-1.06848800
C	-3.34922400	5.81132800	-0.20788000
C	-2.35984300	5.84470300	-2.49501900
C	-4.45362400	5.20935500	-0.71504100
C	-3.51789800	5.16656300	-2.96168800
H	-3.27171100	6.07955700	0.83990500
H	-1.54704900	6.87202600	-0.75773400
C	-4.53645500	4.86682100	-2.09709300
H	-5.42261200	4.36056500	-2.46961800
Cl	-5.80958100	4.83591900	0.29755100
Cl	-3.62146300	4.76956500	-4.63804600
O	-1.45431500	6.15287400	-3.37047700
C	-0.26972600	6.88981800	-2.99294800
H	0.31553500	6.30447000	-2.28171100
H	0.28355400	7.02469400	-3.91873100
H	-0.55579400	7.85593800	-2.57218100
Br	-0.92377200	4.32941400	-0.74038300

S	4.06091300	1.94357700	-0.60858900
S	0.88465000	2.38776800	-0.45279100
C	1.96405700	3.18631600	0.72629400
C	3.34323500	2.97510500	0.64816200
C	3.14069400	2.47253300	-2.03504800
C	1.75790700	2.67344800	-1.98735100
C	1.05012600	3.02540700	-3.13726200
H	-0.02261100	3.18096500	-3.09974900
C	1.72869700	3.16418200	-4.34338200
H	1.17462100	3.42591300	-5.23838100
C	3.81376400	2.63796500	-3.24646900
H	4.88982100	2.50049000	-3.28217100
C	3.10677600	2.97104400	-4.39734100
H	3.63728400	3.08787300	-5.33633000
C	1.41735300	3.95811900	1.75089300
H	0.34718800	4.12842700	1.79937900
C	4.17643700	3.54805800	1.61013300
H	5.24982400	3.40236000	1.54247900
C	3.63074400	4.29655000	2.64782100
H	4.28657600	4.72842200	3.39627700
C	2.25511500	4.50244300	2.71829600
H	1.82856400	5.09181000	3.52269600

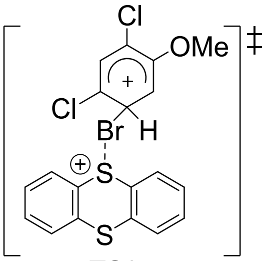

TS3

C	-2.38791100	0.57311500	0.71731800
C	-2.51913800	-0.67395000	-0.03333100
C	-2.86256900	1.75830000	0.22666900
C	-3.33189200	-0.63043500	-1.25112700
C	-3.58108400	1.72682300	-1.01291500
H	-2.66472100	-1.56424600	0.58010400
H	-1.84644100	0.52392600	1.65538200
C	-3.82225400	0.55225500	-1.73949800
H	-4.39042300	0.59315800	-2.66161800

Cl	-3.61885700	-2.09332700	-2.07186900
Cl	-4.16632500	3.19358000	-1.62585400
O	-2.73195000	2.95732200	0.78867600
C	-1.94120100	3.03951900	1.97849000
H	-0.92702000	2.67608800	1.78516500
H	-1.91664000	4.09497200	2.24061200
H	-2.40456100	2.46591000	2.78663500
Br	-0.61733100	-0.92146000	-0.79015600
S	4.81399400	0.47270400	-0.21432500
S	2.22724100	-0.83875900	-1.62959300
C	2.84540000	-1.47986300	-0.08514900
C	3.97004700	-0.90959600	0.51862500
C	3.43089700	1.47654900	-0.70321600
C	2.31360200	0.91982600	-1.33341500
C	1.27557300	1.74219900	-1.77489500
H	0.40905300	1.30908200	-2.26276400
C	1.35841300	3.11894400	-1.59391300
H	0.55423800	3.75485400	-1.94916600
C	3.49252300	2.85622500	-0.49949000
H	4.35070100	3.28389600	0.00955600
C	2.46360000	3.67480400	-0.95380200
H	2.52456900	4.74705700	-0.80016900
C	2.22740500	-2.59117800	0.49044200
H	1.34439300	-3.02189000	0.02837500
C	4.47358600	-1.45873200	1.69887200
H	5.33437800	-1.00114500	2.17621400
C	3.87710200	-2.58707500	2.25144600
H	4.28314900	-3.01610200	3.16141800
C	2.75525800	-3.15222500	1.64891700
H	2.28232500	-4.02613300	2.08411400

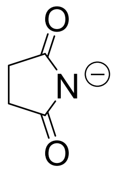

INT2

C	-2.11277000	-0.63119300	-0.49713500
C	-2.50737500	0.32864300	0.61236300
H	-2.84510400	-0.70949000	-1.30574100

H	-1.90155400	-1.64698400	-0.14961700
H	-2.56298100	-0.12898600	1.60414000
H	-3.45745300	0.84196000	0.43655000
C	-0.81214400	-0.01209900	-1.04860900
C	-1.36816400	1.36683900	0.59547000
N	-0.45052200	1.11184700	-0.37504400
O	-1.33944400	2.30667000	1.39058800
O	-0.20587300	-0.52063100	-1.99219700

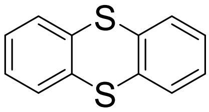

TT

C	-7.09621500	-2.77860500	0.40691900
C	-5.78249200	-2.81746900	-0.04967300
C	-5.18242100	-1.65998200	-0.54873800
C	-5.89089400	-0.45404700	-0.55260000
C	-7.19623900	-0.41325200	-0.05928600
C	-7.80243700	-1.57743500	0.40197600
C	-3.52883700	0.93388900	-0.54989000
C	-2.82029000	-0.27199800	-0.54585900
C	-1.51714900	-0.31070300	-0.04659700
H	-0.97941100	-1.25362700	-0.02612000
C	-0.91312100	0.85533600	0.41282300
C	-1.61952700	2.05641400	0.41011900
C	-2.93117700	2.09335000	-0.05251300
H	-7.56118000	-3.68534600	0.77968800
H	-5.21948600	-3.74549800	-0.02864800
H	-7.73396600	0.52978400	-0.04476500
H	-8.82259900	-1.54113900	0.76965400
H	0.10542100	0.82055800	0.78510100
H	-1.15634300	2.96462100	0.78153600
H	-3.49441700	3.02134300	-0.03749400
S	-5.17251100	1.02731100	-1.22707500
S	-3.53553900	-1.75601300	-1.21769100

IV. Reference

[1] Chan Y.-C., Yeung Y.-Y. Halogen Bond Catalyzed Bromocarbocyclization. *Angew. Chem. Int. Ed.* **2018**, *57*, 3483-3487.

[2] Jammi S., Mouysset D., Siri D., Bertrand M. P., Feray L. Theoretical Support for the Involvement of a Radical Pathway in the Formation of Allenylzincs from Propargyl Iodides and Dialkylzincs: Influence of Zinc Coordination. *J. Org. Chem.* **2013**, *78*, 1589-1603.

[3] Aukland M. H., Talbot F. J. T., Fernández-Salas J. A., Ball M., Pulis A. P., Procter D. J. An Interrupted Pummerer/Nickel-Catalysed Cross-Coupling Sequence. *Angew. Chem. Int. Ed.* **2018**, *57*, 9785-9789.

[4] Hu Z., Belitz F., Zhang G., Papp F., Gooßen L. J. Ru-Catalyzed (E)-Specific ortho-C–H Alkenylation of Arenecarboxylic Acids by Coupling with Alkenyl Bromides. *Org. Lett.* **2021**, *23*, 3541-3545.

[5] M. J. Frisch, G. W. Trucks, H. B. Schlegel, G. E. Scuseria, M. A. Robb, J. R. Cheeseman, G. Scalmani, V. Barone, B. Mennucci, G. A. Petersson, H. Nakatsuji, M. Caricato, X. Li, H. P. Hratchian, A. F. Izmaylov, J. Bloino, G. Zheng, J. L. Sonnenberg, M. Hada, M. Ehara, K. Toyota, R. Fukuda, J. Hasegawa, M. Ishida, T. Nakajima, Y. Honda, O. Kitao, H. Nakai, T. Vreven, J. A. Montgomery, Jr., J. E. Peralta, F. Ogliaro, M. Bearpark, J. J. Heyd, E. Brothers, K. N. Kudin, V. N. Staroverov, T. Keith, R. Kobayashi, J. Normand, K. Raghavachari, A. Rendell, J. C. Burant, S. S. Iyengar, J. Tomasi, M. Cossi, N. Rega, J. M. Millam, M. Klene, J. E. Knox, J. B. Cross, V. Bakken, C. Adamo, J. Jaramillo, R. Gomperts, R. E. Stratmann, O. Yazyev, A. J. Austin, R. Cammi, C. Pomelli, J. W. Ochterski, R. L. Martin, K. Morokuma, V. G. Zakrzewski, G. A. Voth, P. Salvador, J. J. Dannenberg, S. Dapprich, A. D. Daniels, O. Farkas, J. B. Foresman, J. V. Ortiz, J. Cioslowski, and D. J. Fox, Gaussian, Inc., Wallingford CT, 2013.

[6] Zhao, Y.; Truhlar, D. G. Theor. *Chem. Account.* **2008**, *120*, 215-241.

[7] Marenich, A. V., Cramer, C. J., Truhlar, D. G. *J. Phys. Chem. B.* **2009**, *113*, 6378-6396.
[8] Legault, C. Y. CYL View, version 1.0 b; Universite de Sherbrooke, Sherbrooke, Quebec, Canada, 2009; http://www.cylview.org.

V. NMR Spectra of Starting Materials and Products:

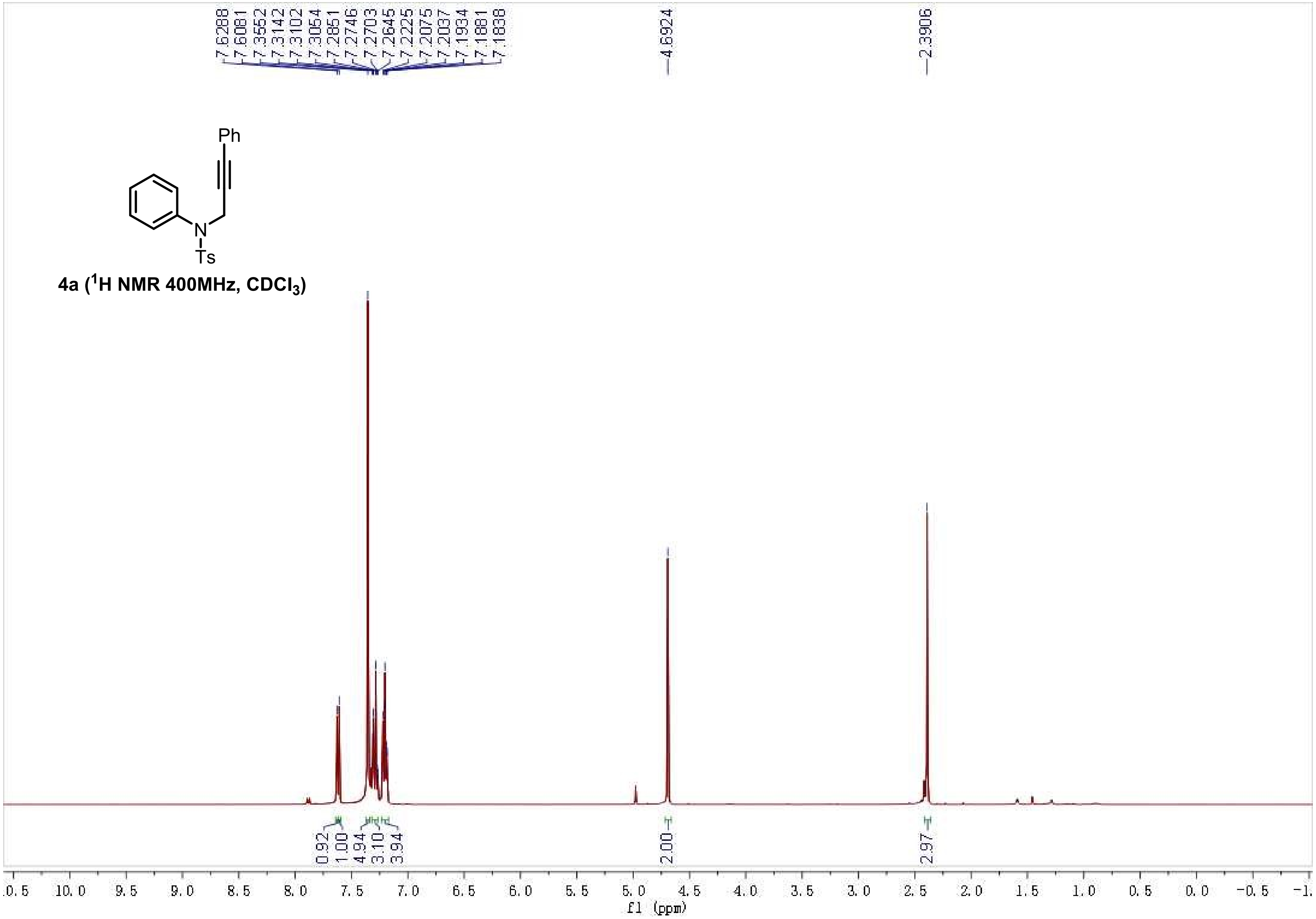

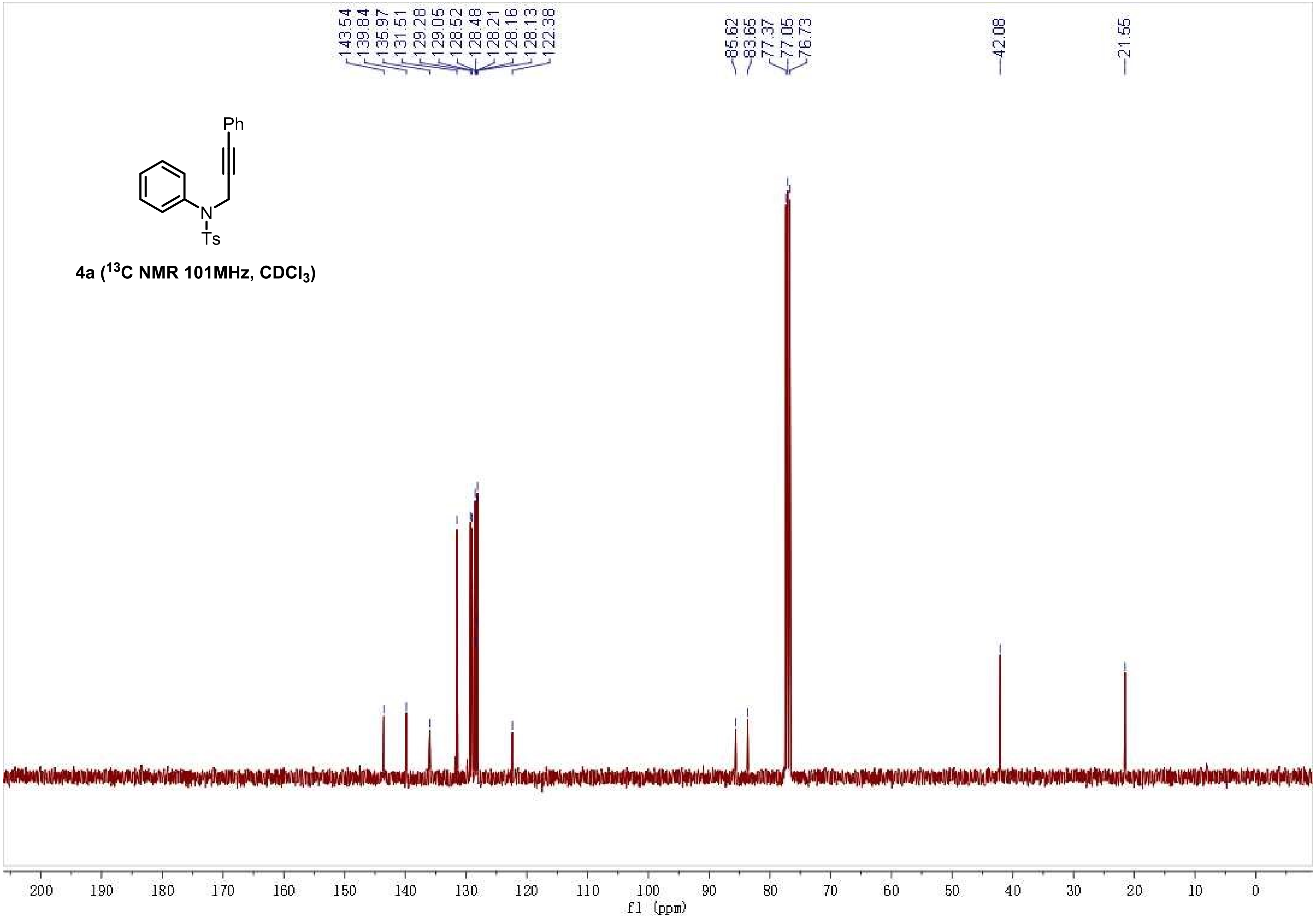

4a (^{13}C NMR 101MHz, $CDCl_3$)

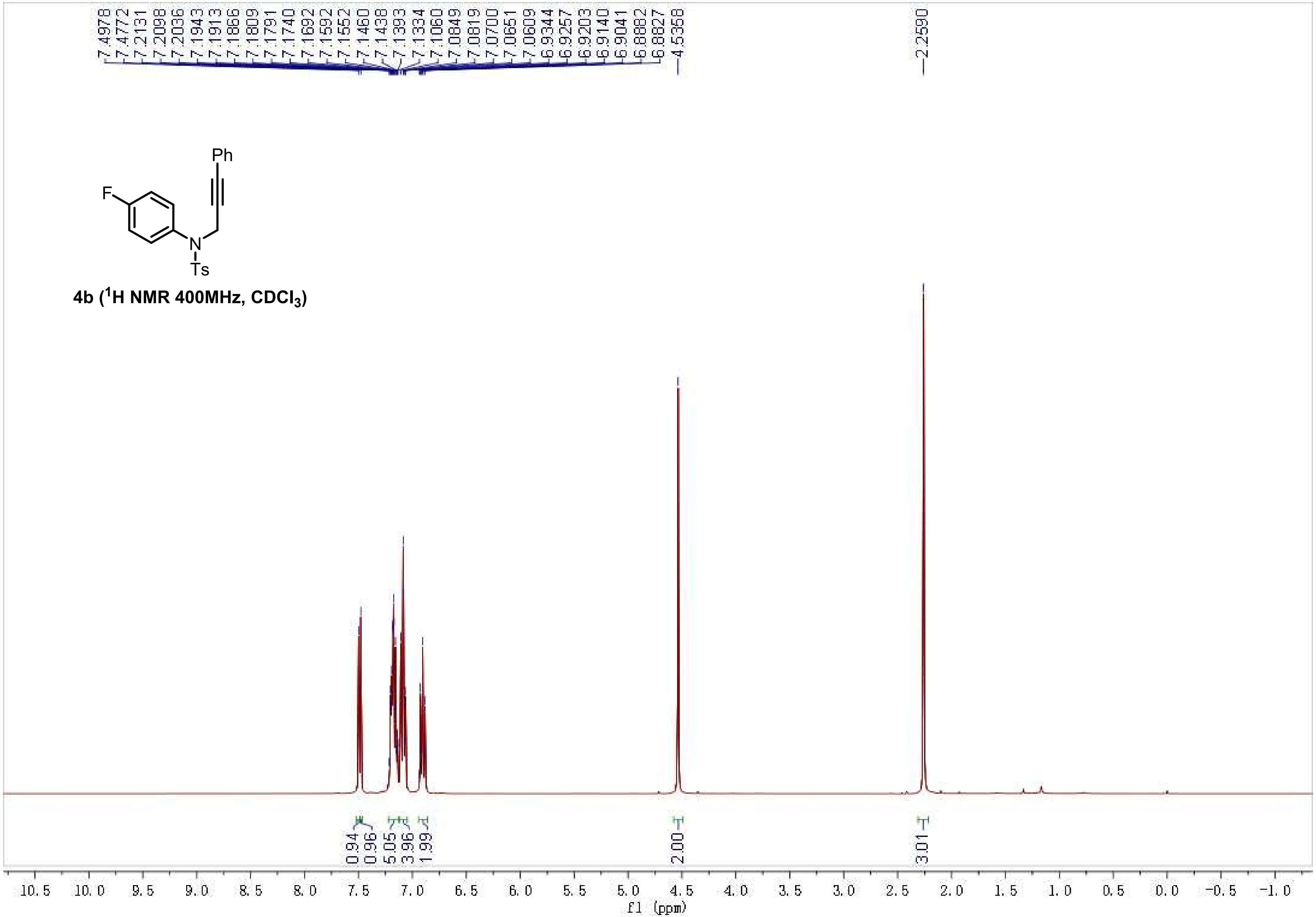

7.4978
7.4772
7.2131
7.2098
7.2036
7.1943
7.1913
7.1866
7.1809
7.1791
7.1740
7.1692
7.1592
7.1552
7.1460
7.1438
7.1393
7.1334
7.1060
7.0849
7.0819
7.0700
7.0651
7.0609
6.9344
6.9257
6.9203
6.9140
6.9041
6.8882
6.8827
4.5358
2.2590
Ph
F
N
Ts
4b (¹H NMR 400MHz, CDCl₃)
0.94
0.96
5.05
3.96
1.99
2.00
3.01
10.5 10.0 9.5 9.0 8.5 8.0 7.5 7.0 6.5 6.0 5.5 5.0 4.5 4.0 3.5 3.0 2.5 2.0 1.5 1.0 0.5 0.0 -0.5 -1.0
f1 (ppm)

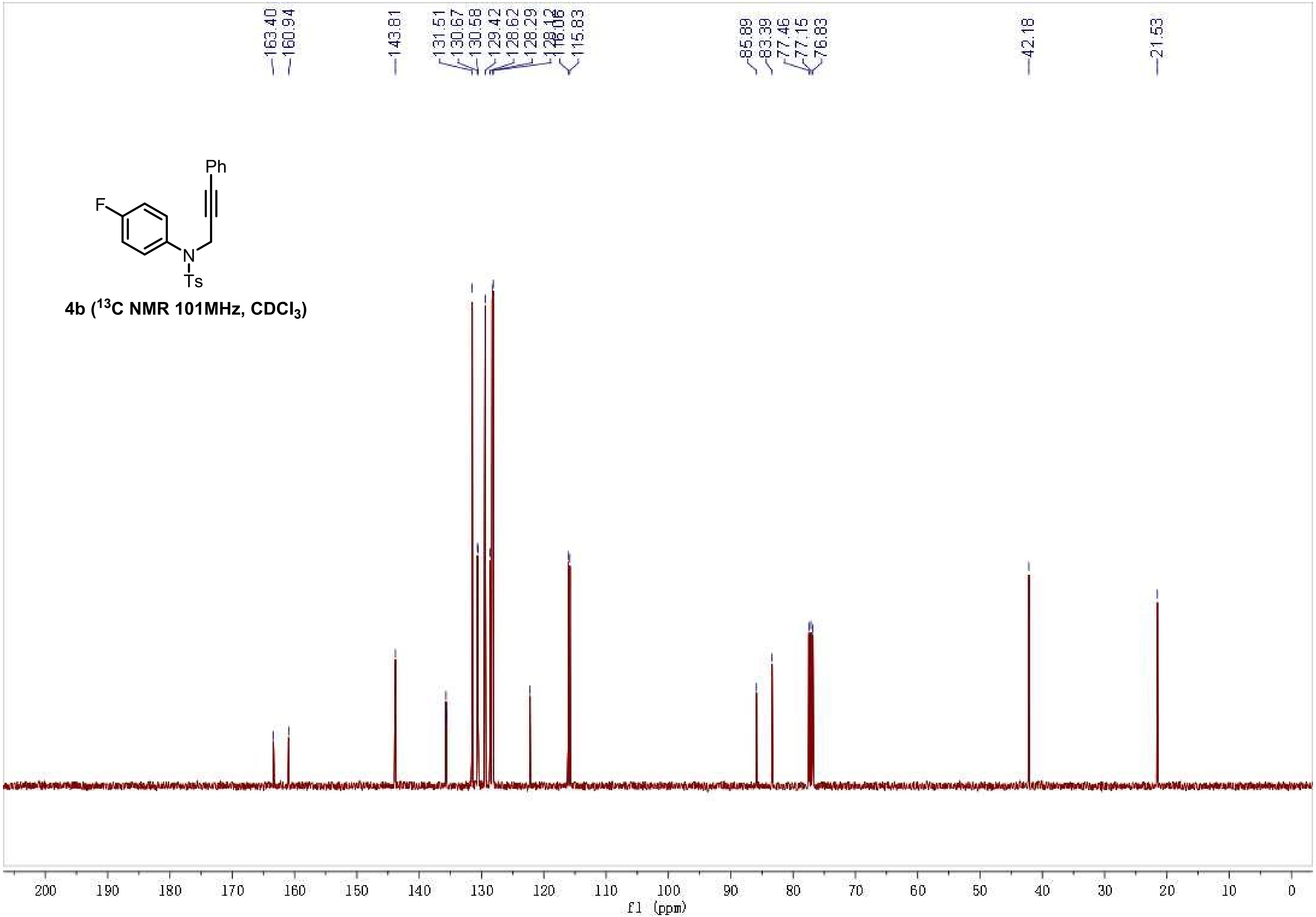

4b (^{13}C NMR 101MHz, $CDCl_3$)

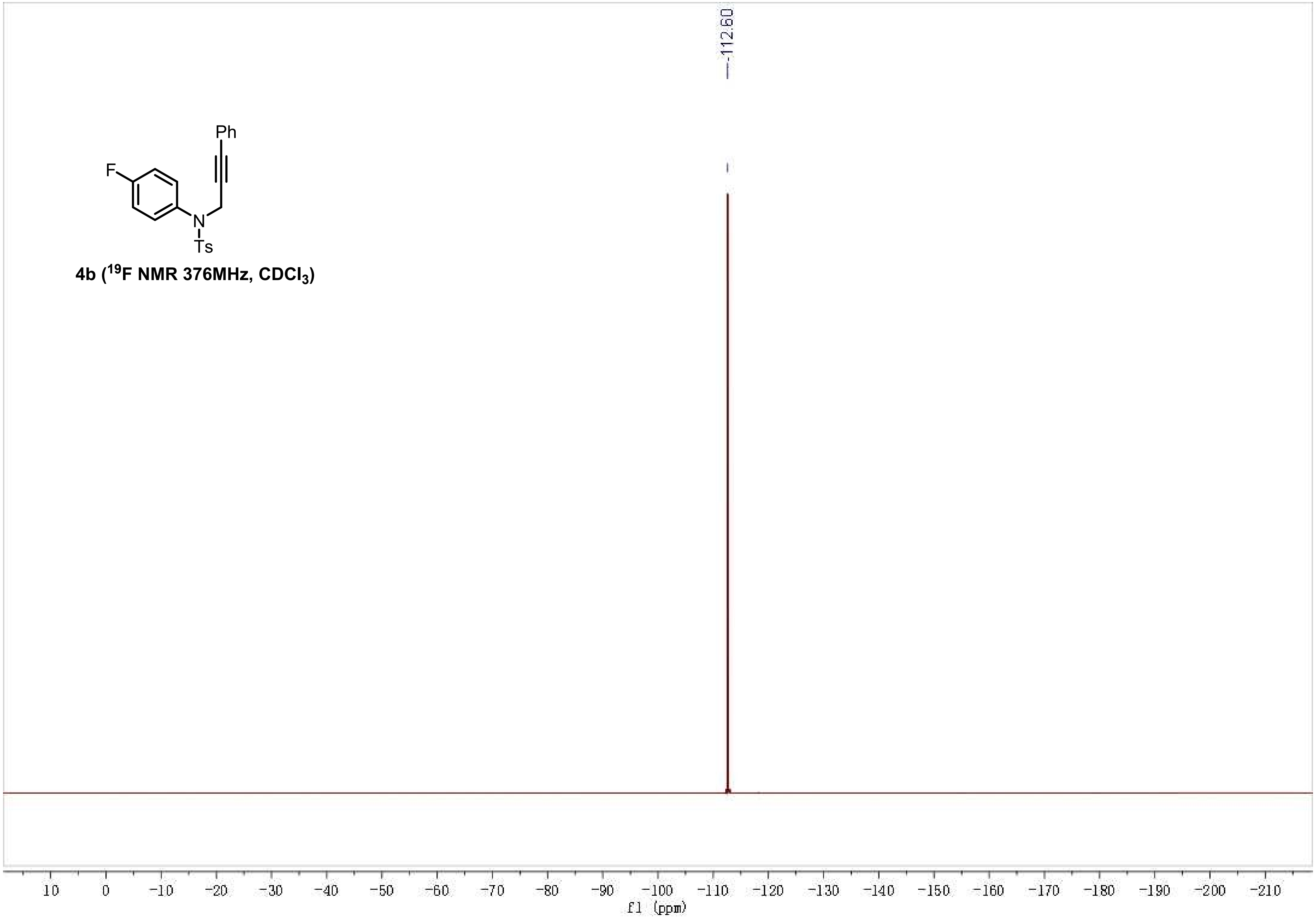

4b (^{19}F NMR 376MHz, $CDCl_3$)

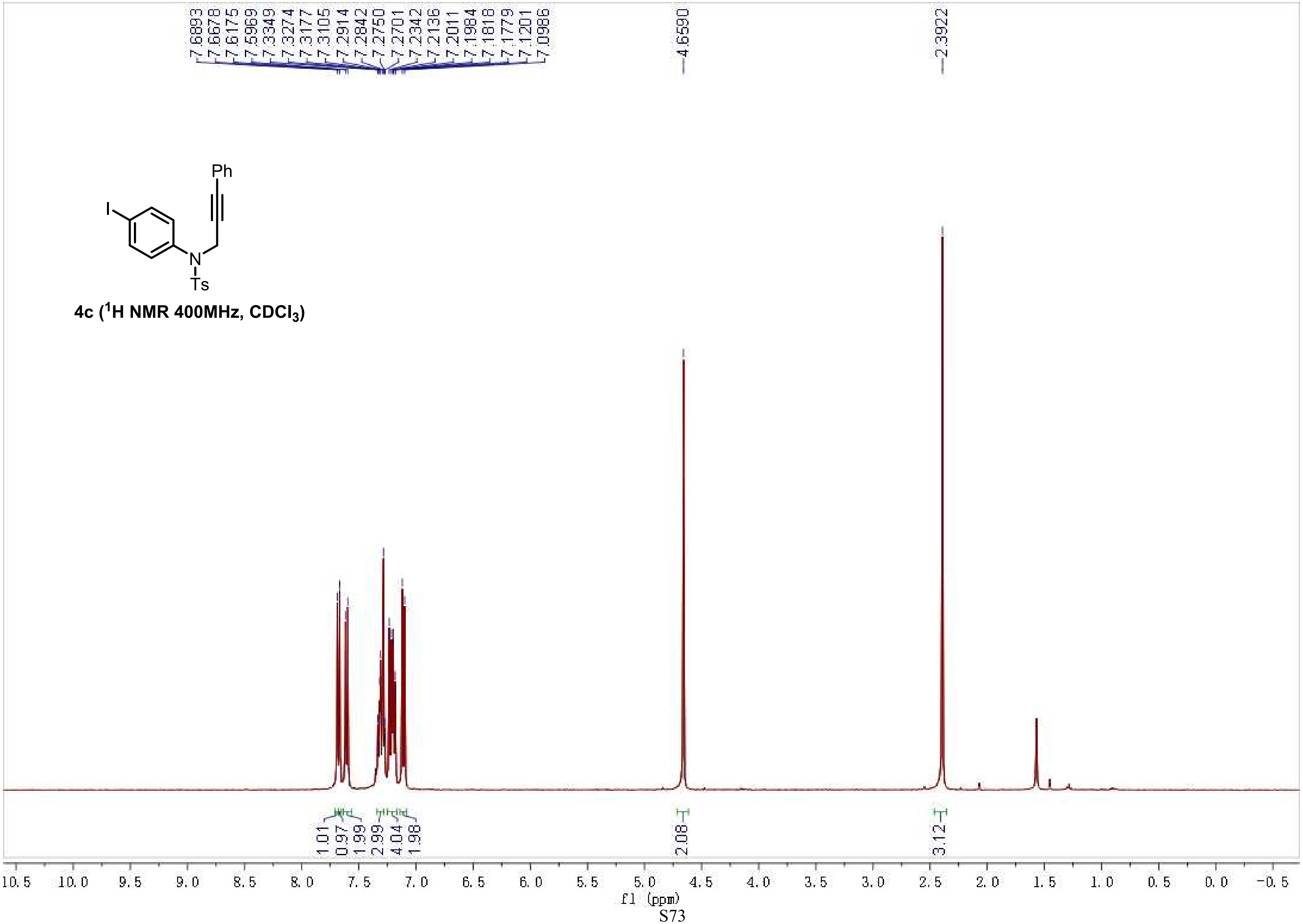

4c (^{1}H NMR 400MHz, $CDCl_3$)

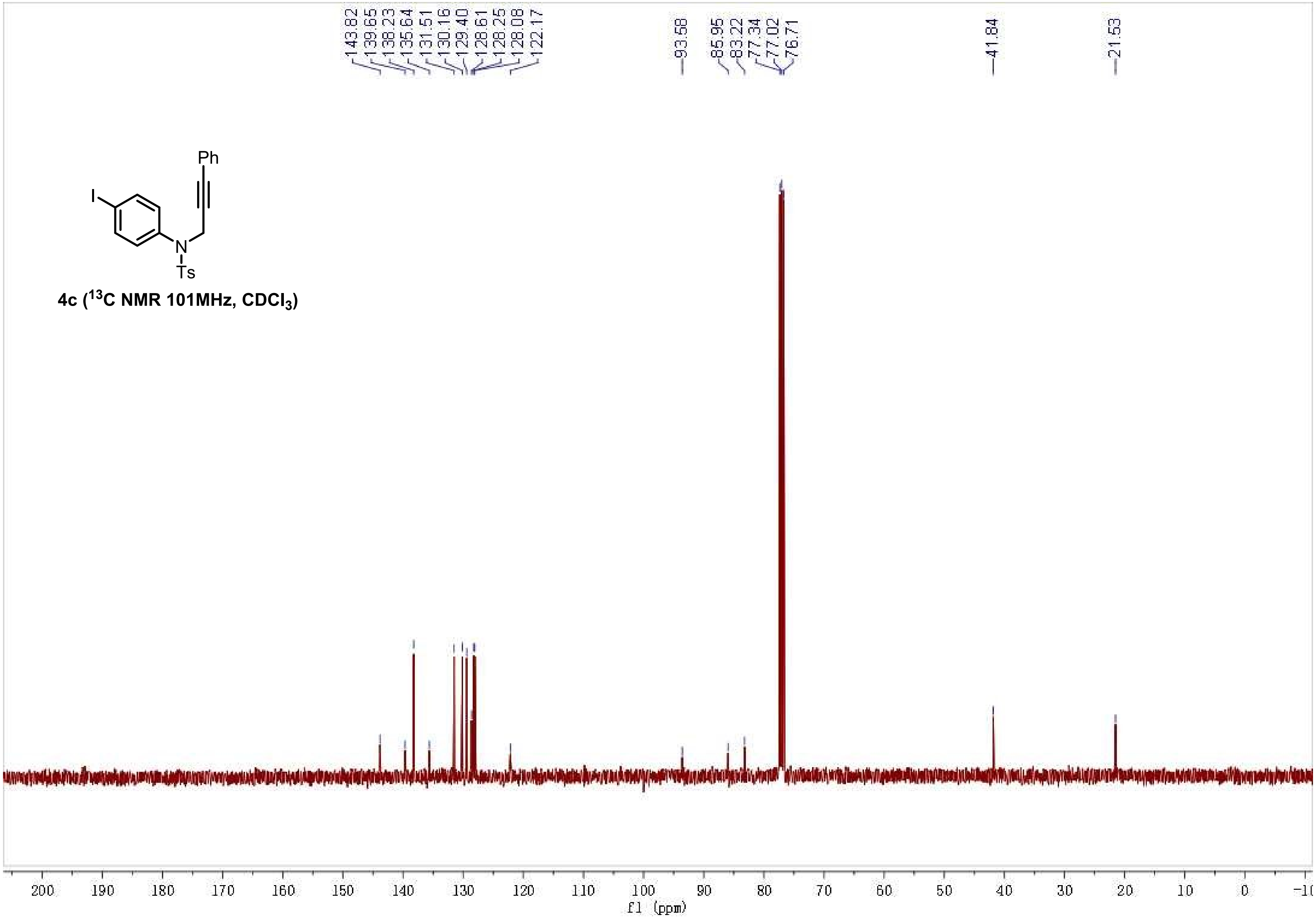

4c (^{13}C NMR 101MHz, $CDCl_3$)

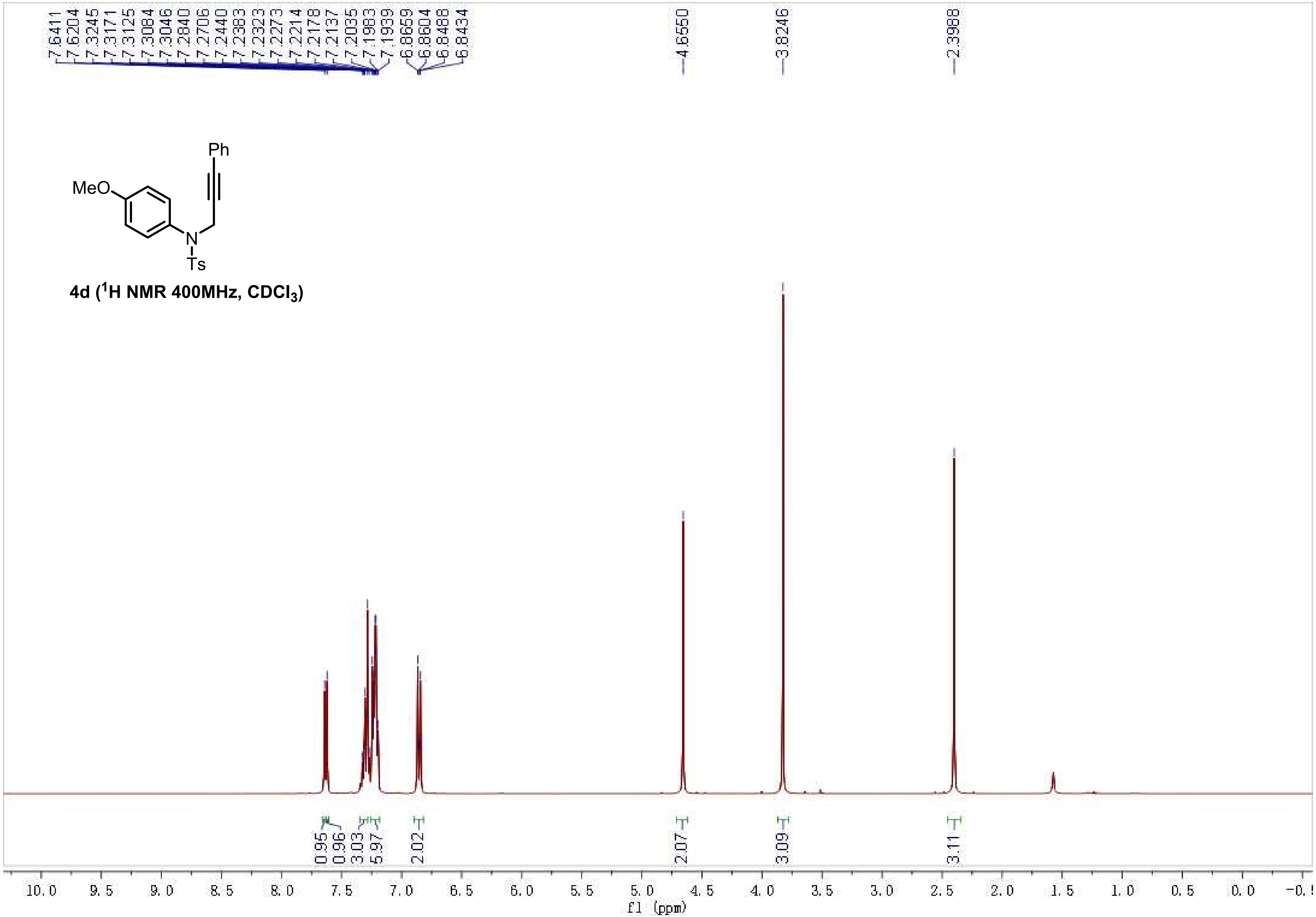

4d (^{1}H NMR 400MHz, $CDCl_3$)

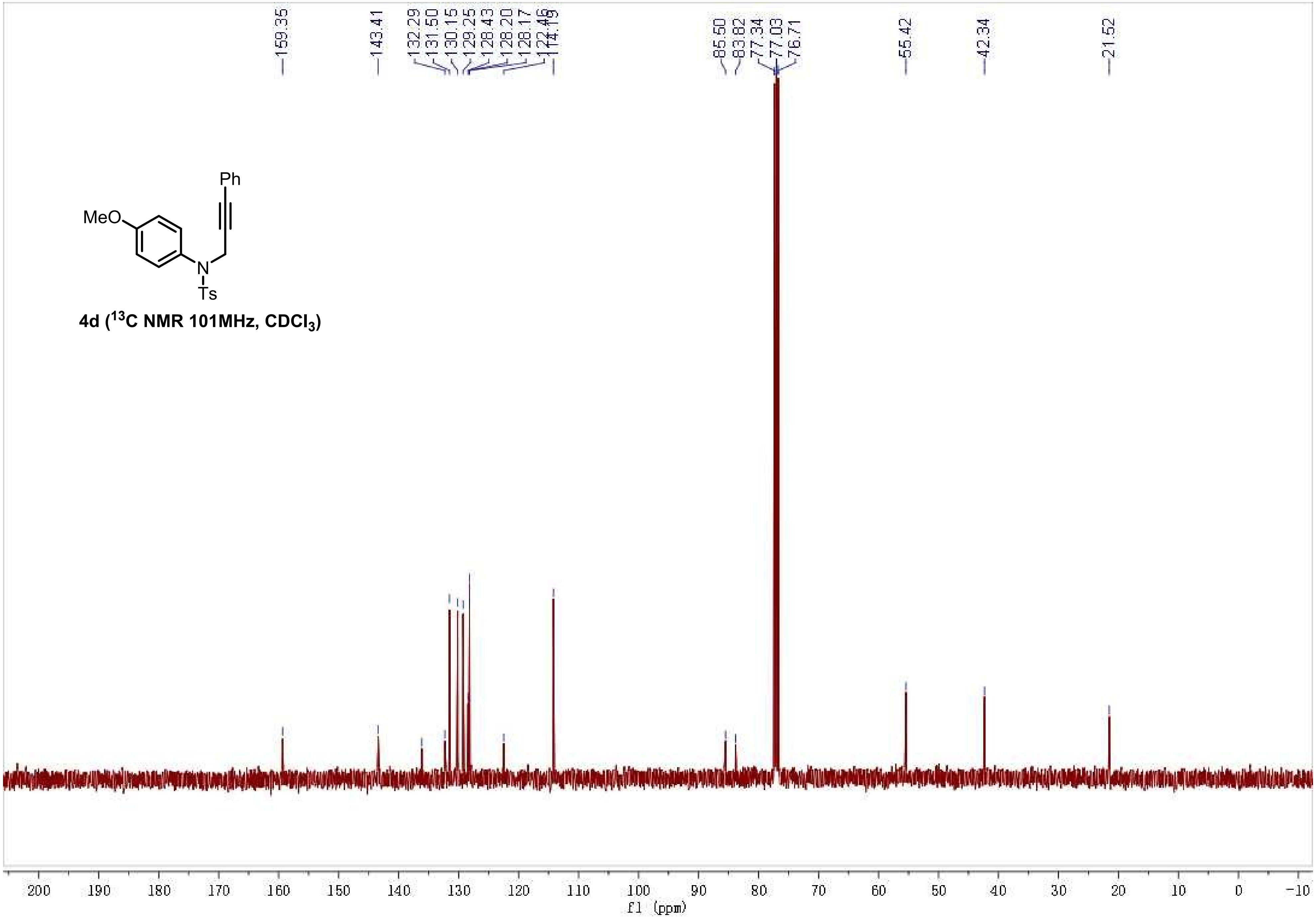

159.35
143.41
132.29
131.50
130.15
129.25
128.43
128.20
128.17
85.50
83.82
77.34
77.03
76.71
55.42
42.34
21.52
Ph
MeO
N
Ts
4d (13C NMR 101MHz, CDCl3)
200 190 180 170 160 150 140 130 120 110 100 90 80 70 60 50 40 30 20 10 0 -10
f1 (ppm)

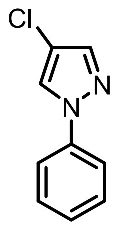

1a-Cl (^{1}H NMR 400MHz, $CDCl_3$)

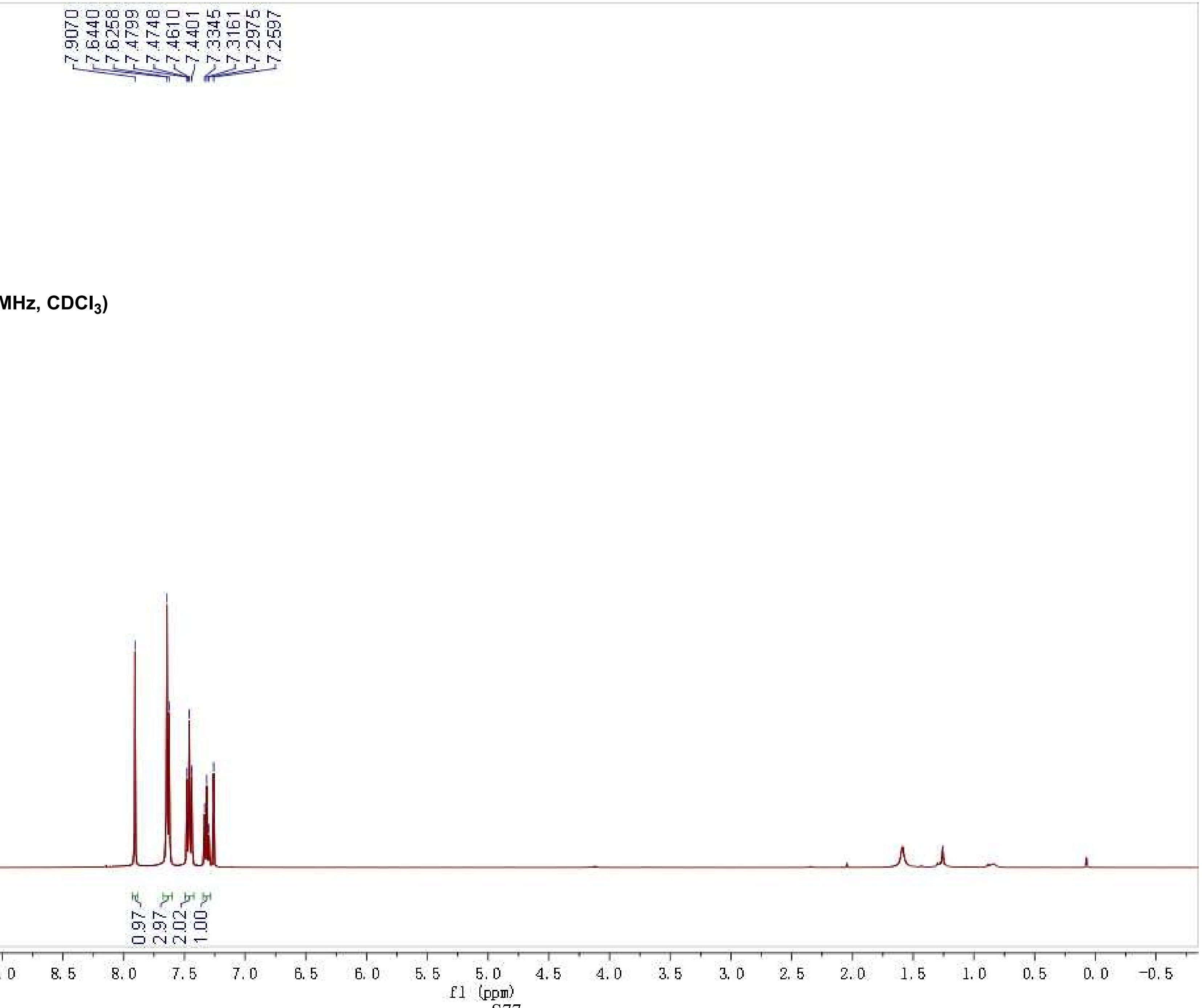

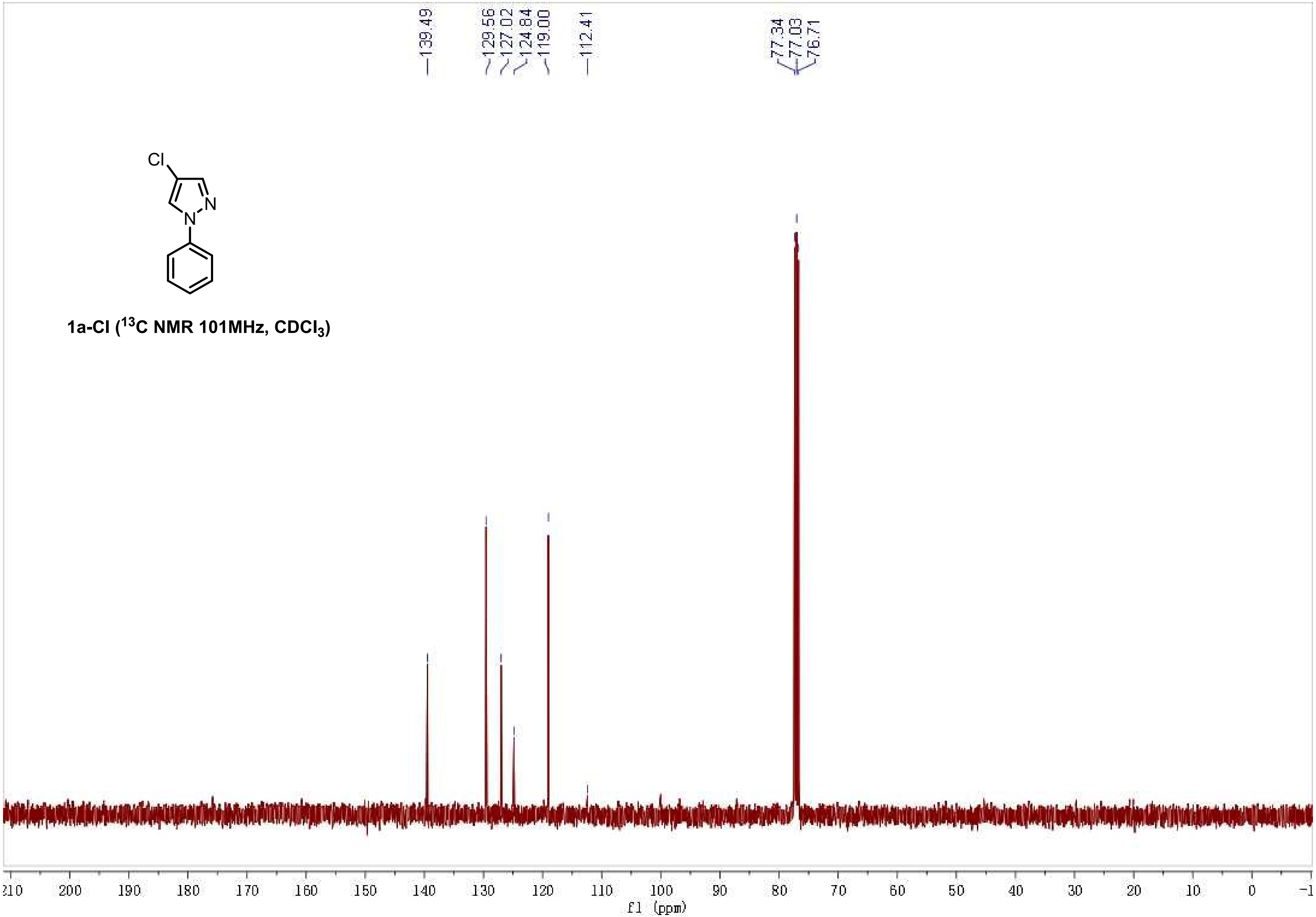

1a-Cl (^{13}C NMR 101MHz, $CDCl_3$)

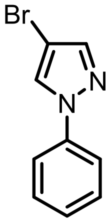

1a-Br (^{1}H NMR 400MHz, $CDCl_3$)

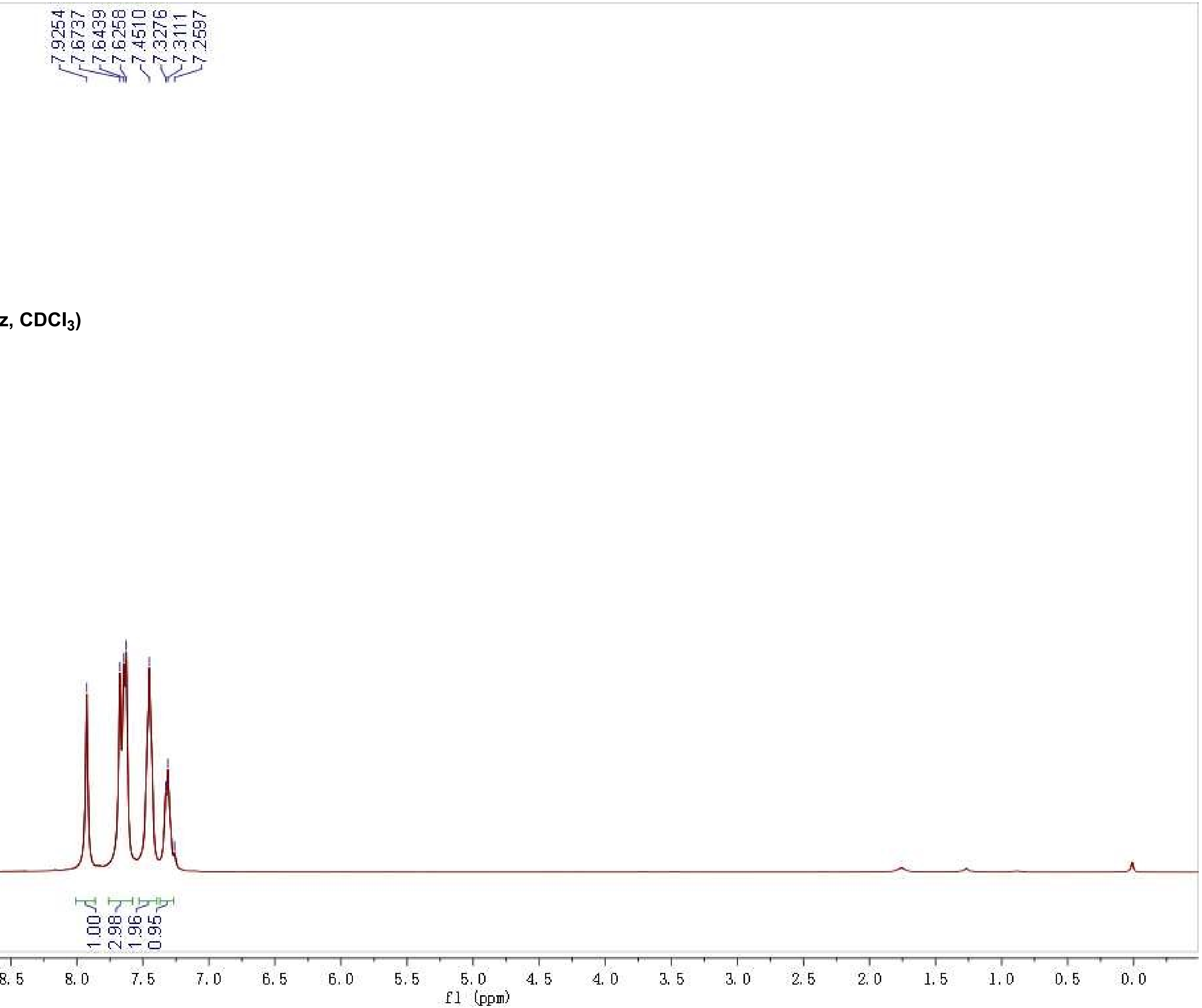

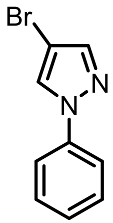

1a-Br (^{13}C NMR 101MHz, $CDCl_3$)

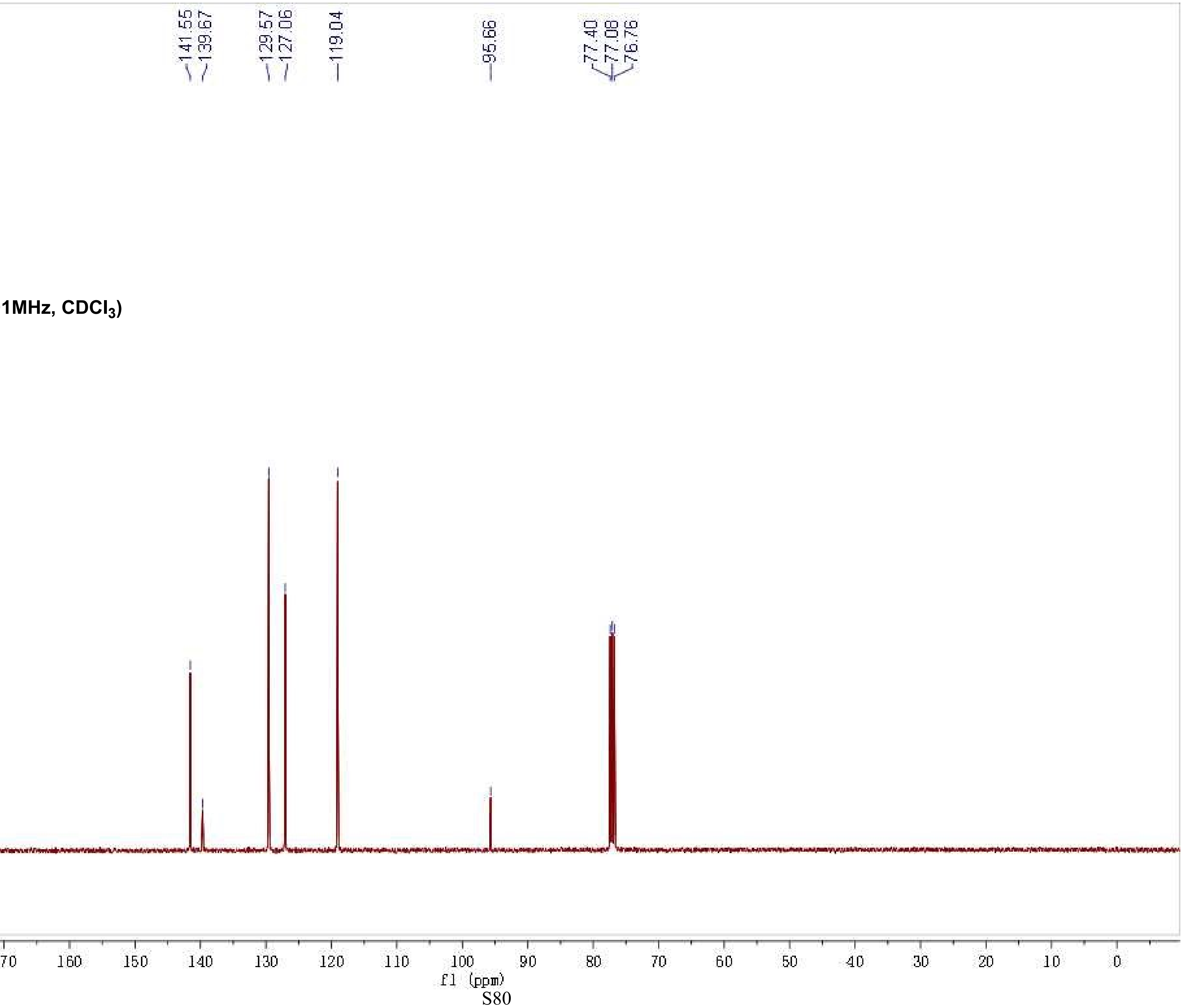

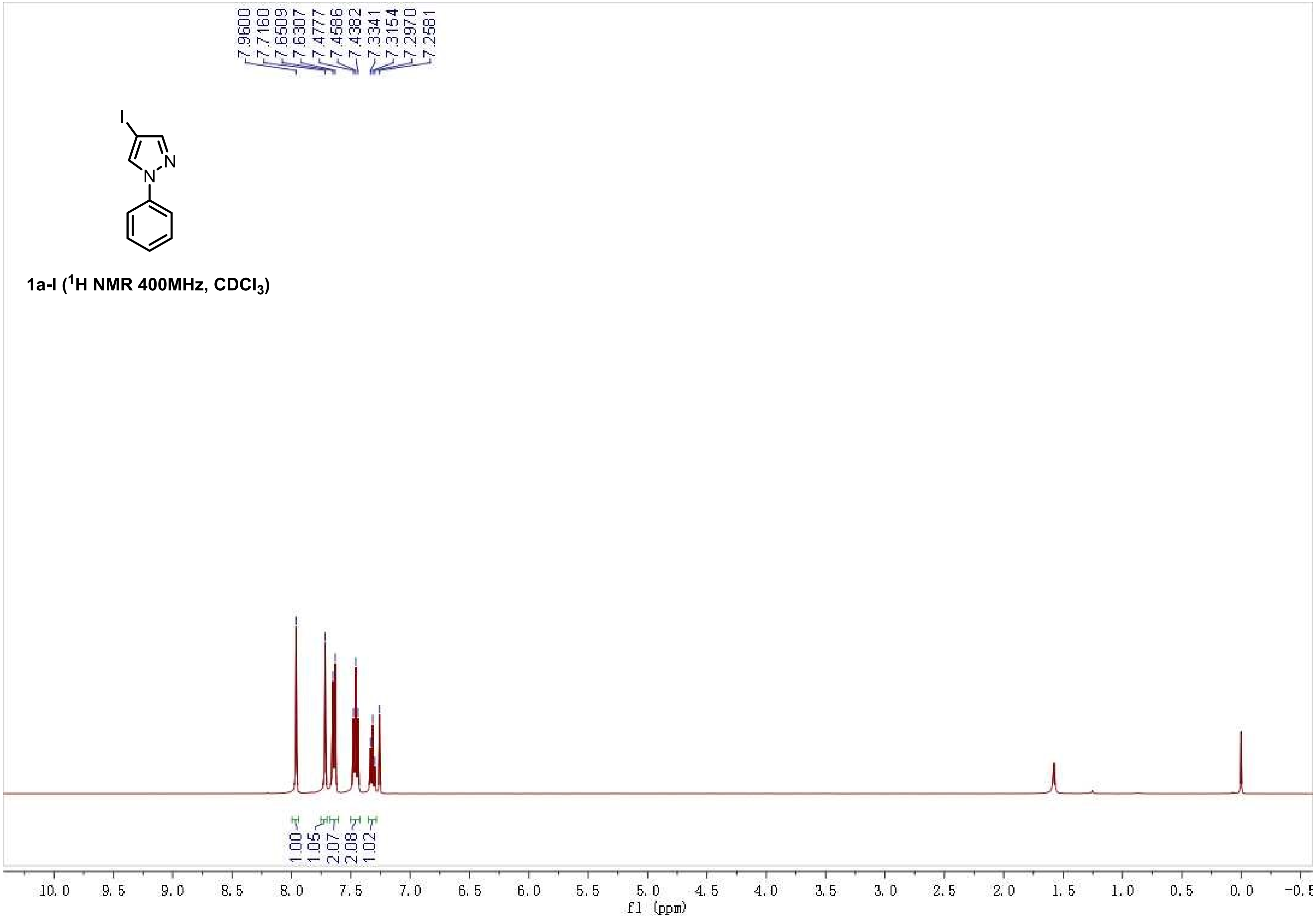

1a-I (^{1}H NMR 400MHz, CDCl$_3$)

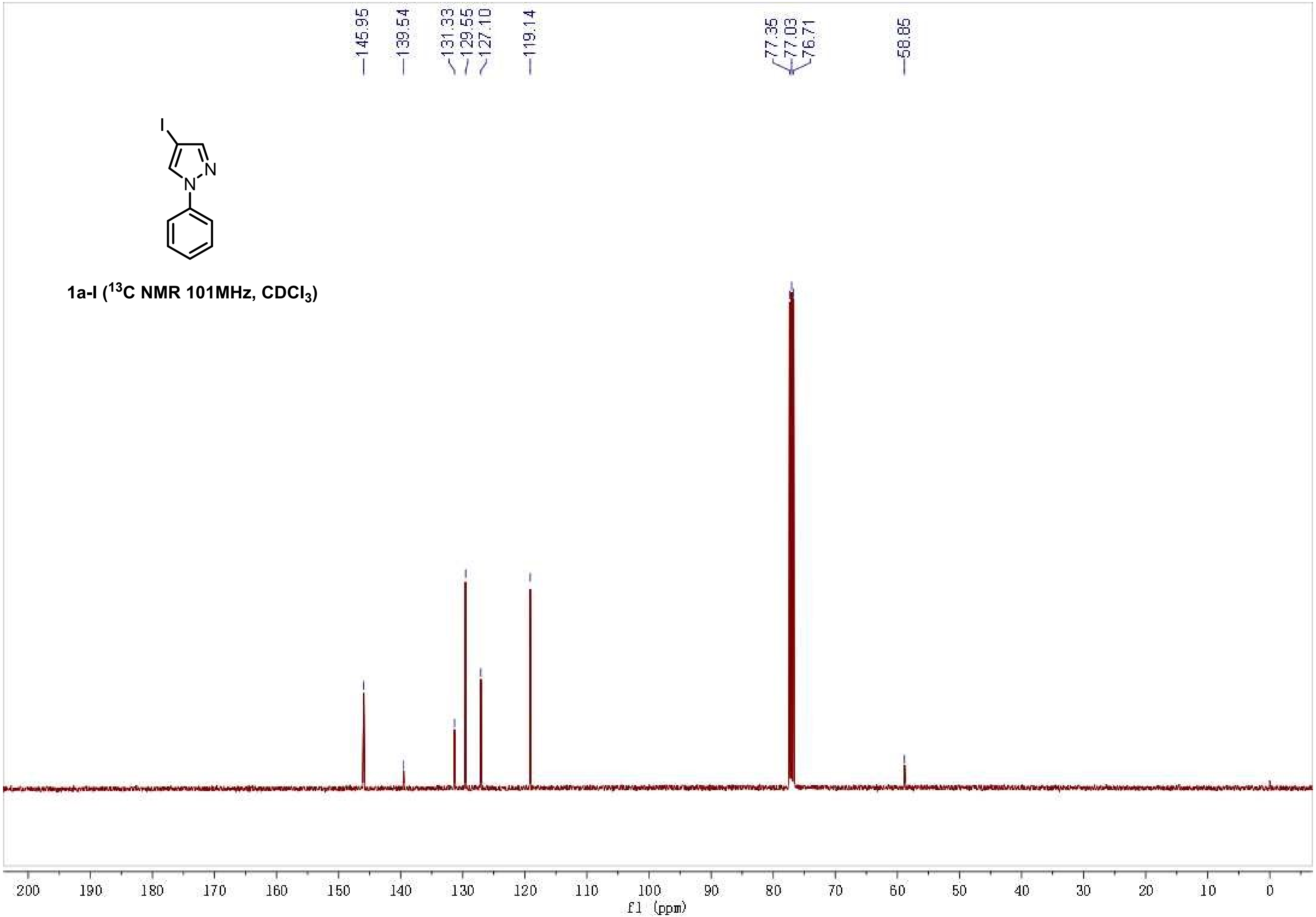

1a-I (^{13}C NMR 101MHz, $CDCl_3$)

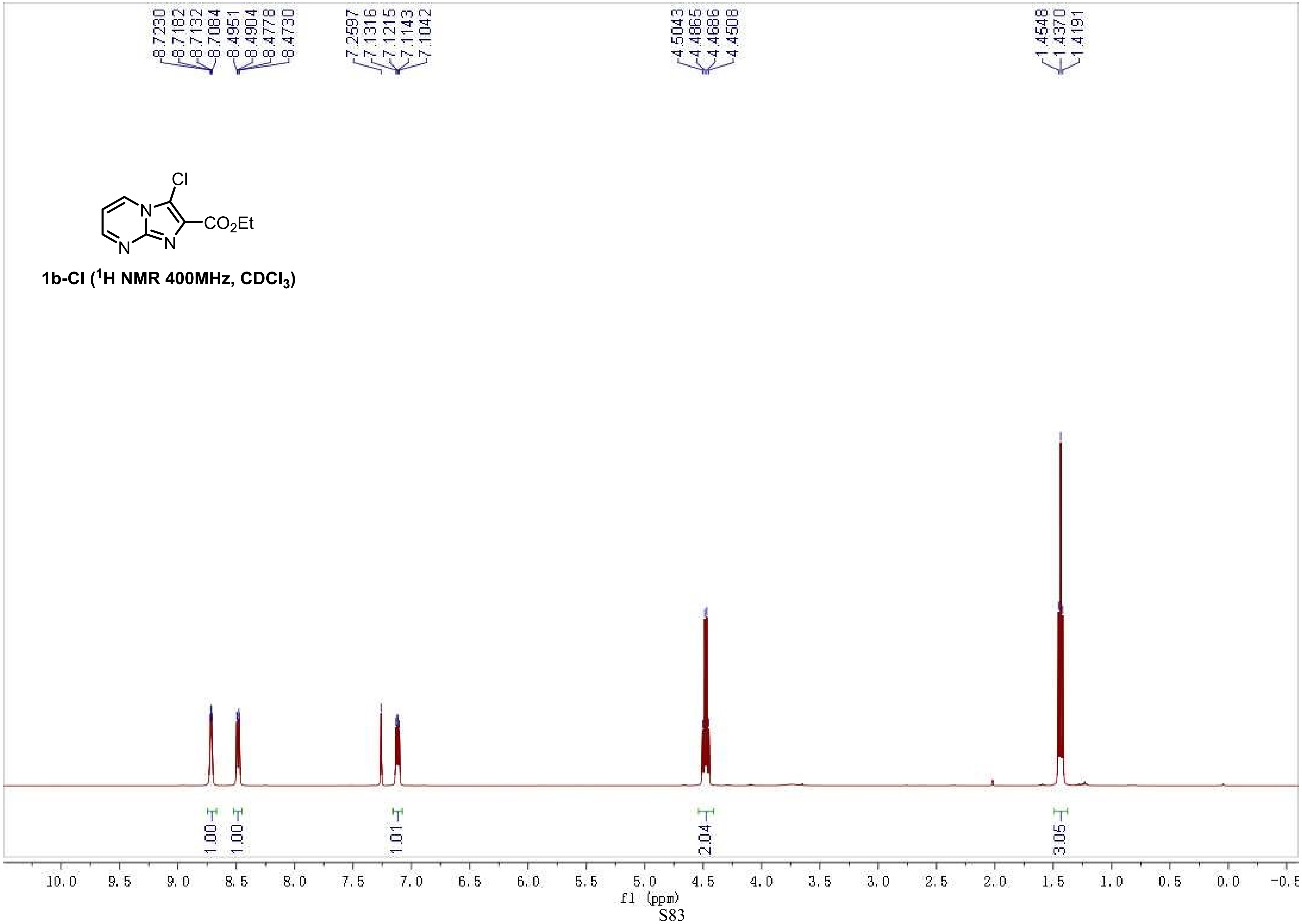

8.7230
8.7182
8.7132
8.7084
8.4951
8.4904
8.4778
8.4730
7.2597
7.1316
7.1215
7.1143
7.1042
4.5043
4.4865
4.4686
4.4508
1.4548
1.4370
1.4191
Cl
CO2Et
N
N
N
1b-Cl (1H NMR 400MHz, CDCl3)
1.00
1.00
1.01
2.04
3.05
10.0 9.5 9.0 8.5 8.0 7.5 7.0 6.5 6.0 5.5 5.0 4.5 4.0 3.5 3.0 2.5 2.0 1.5 1.0 0.5 0.0 -0.5
f1 (ppm)

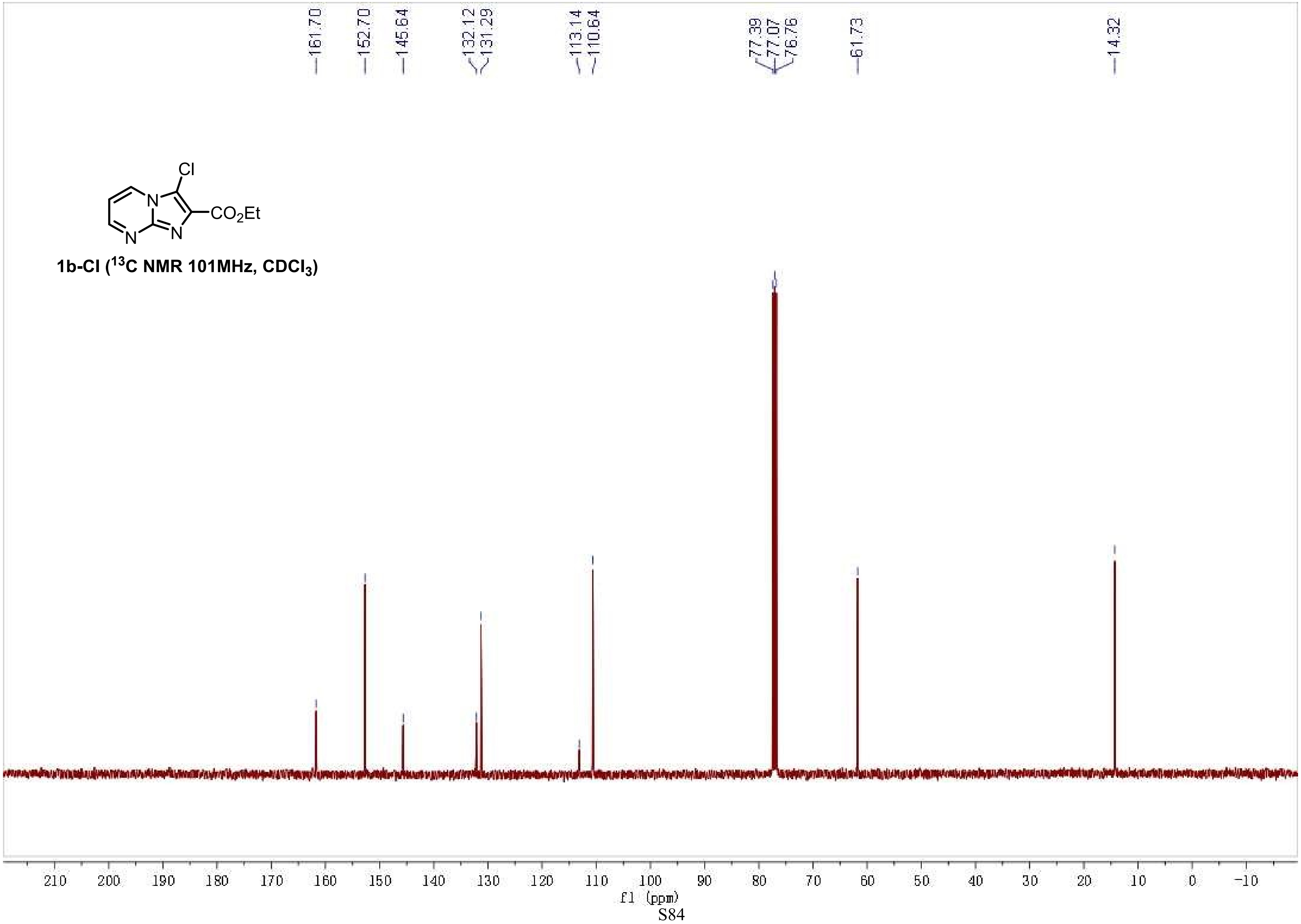

1b-Cl (^{13}C NMR 101MHz, $CDCl_3$)

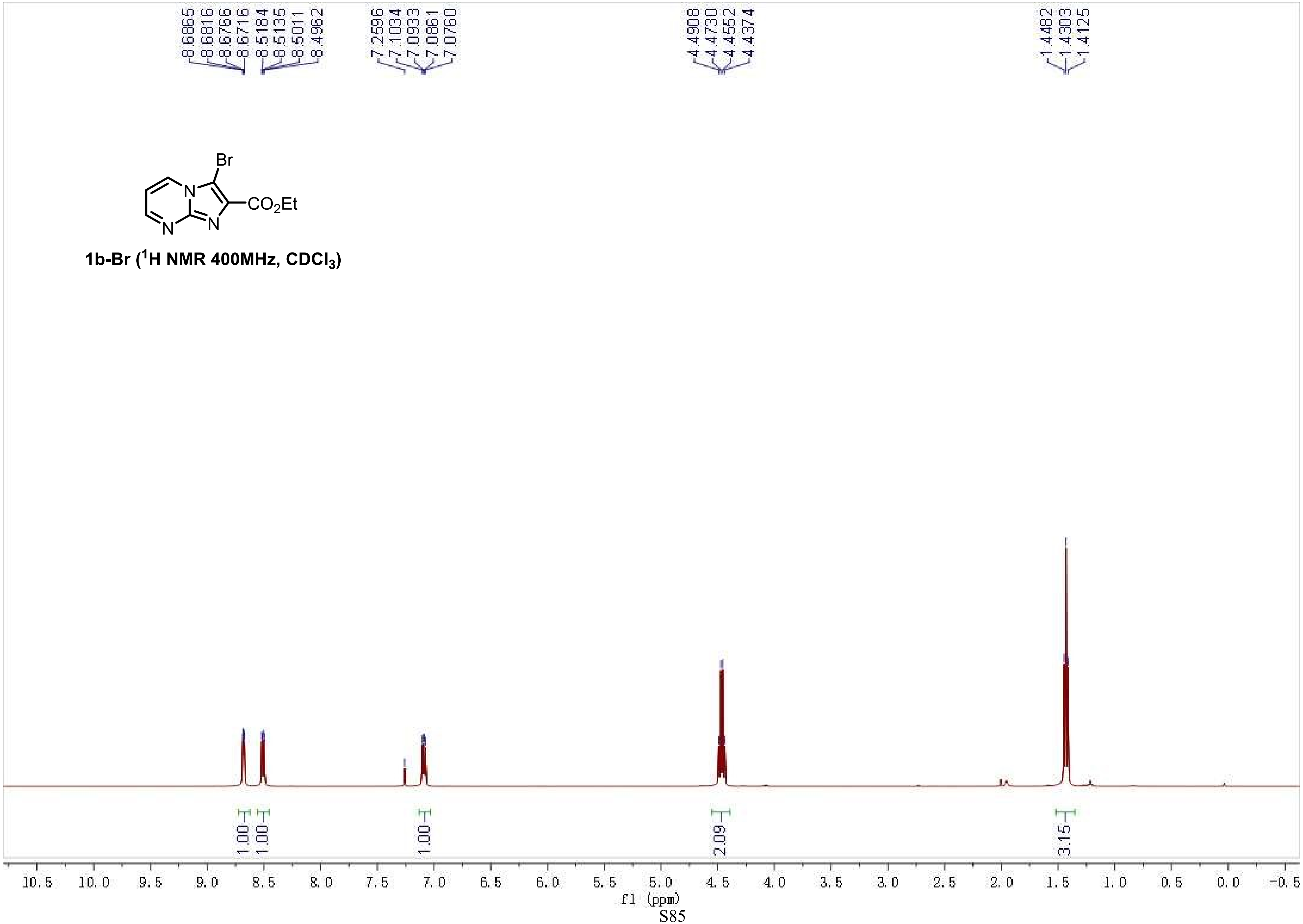

1b-Br (^{1}H NMR 400MHz, $CDCl_3$)

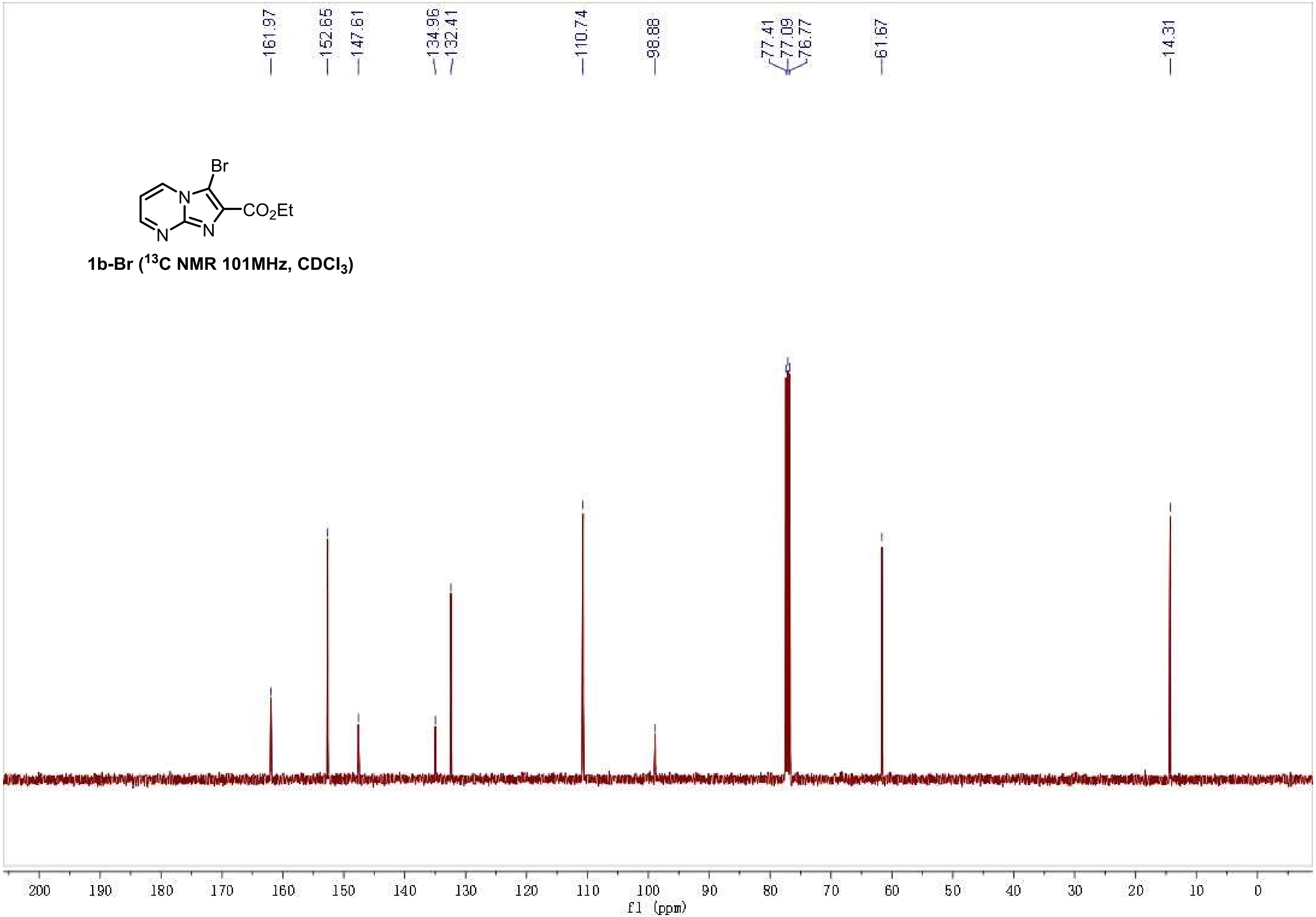

1b-Br (^{13}C NMR 101MHz, $CDCl_3$)

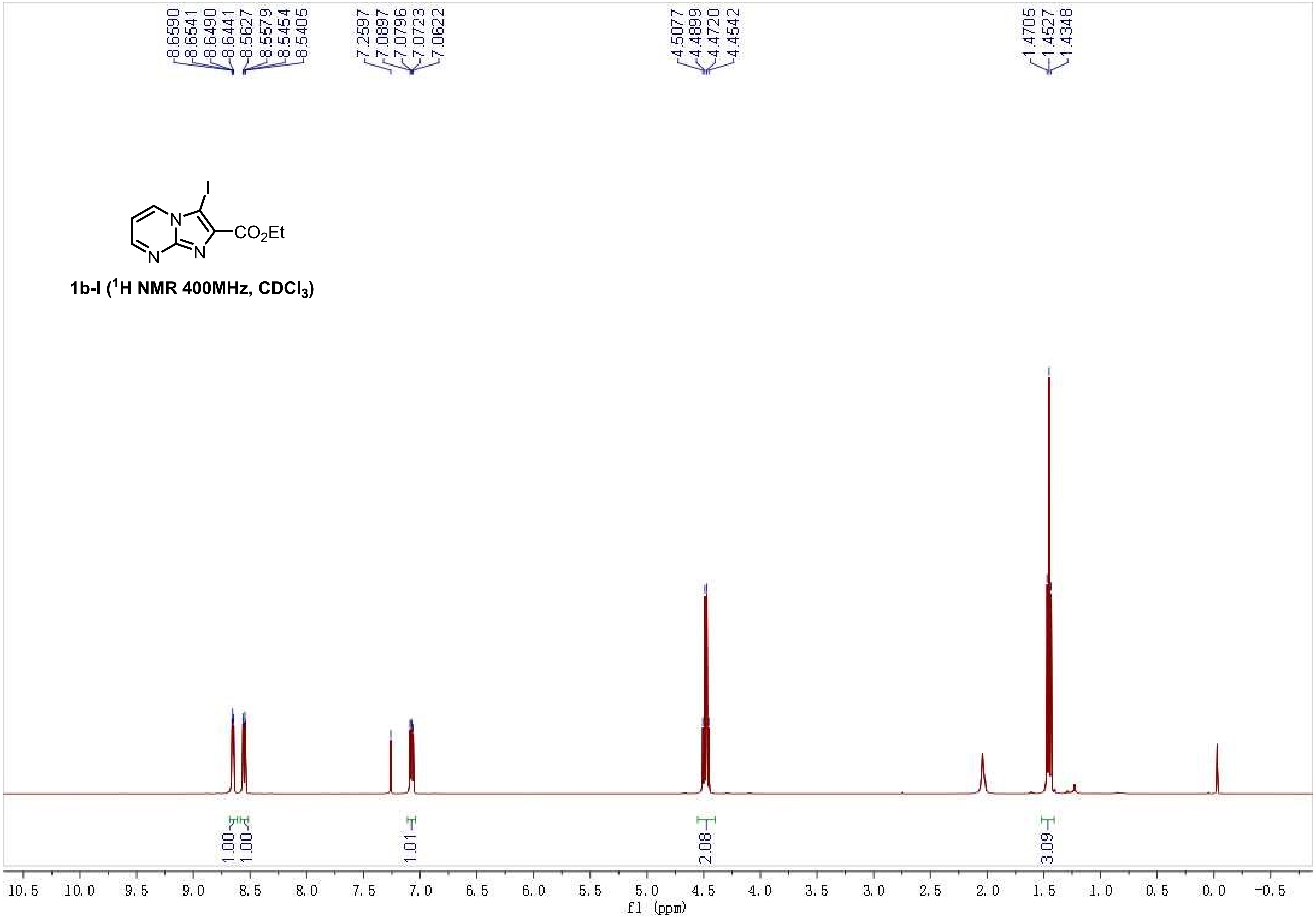
8.6590
8.6541
8.6490
8.6441
8.5627
8.5579
8.5454
8.5405
7.2597
7.0897
7.0796
7.0723
7.0622
4.5077
4.4899
4.4720
4.4542
1.4705
1.4527
1.4348
I
CO2Et
N
N
N
1b-I (1H NMR 400MHz, CDCl3)
1.00
1.00
1.01
2.08
3.09
10.5
10.0
9.5
9.0
8.5
8.0
7.5
7.0
6.5
6.0
5.5
5.0
4.5
4.0
3.5
3.0
2.5
2.0
1.5
1.0
0.5
0.0
-0.5
f1 (ppm)

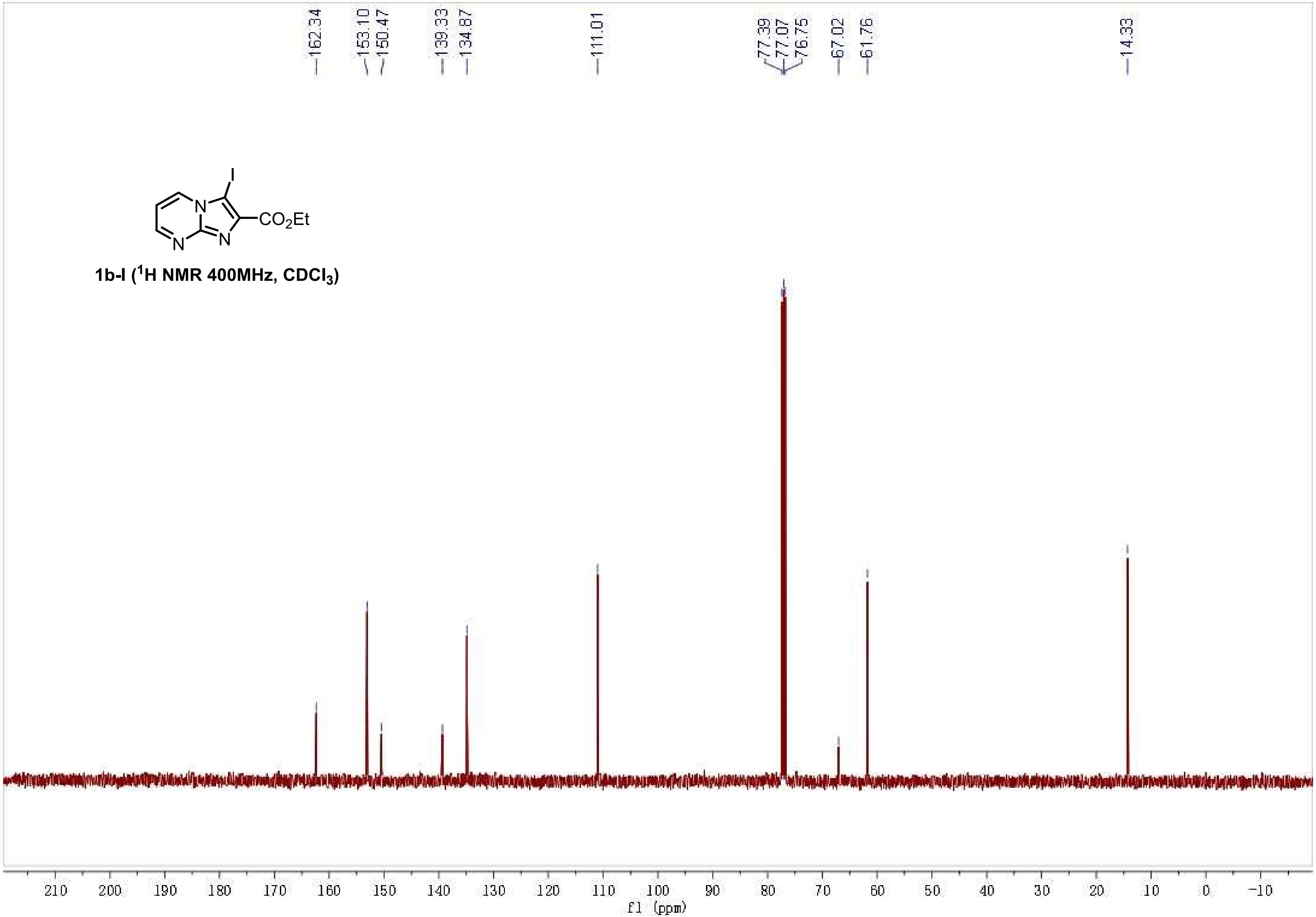

1b-I (^{1}H NMR 400MHz, $CDCl_3$)

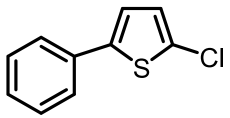

1c-Cl (^{1}H NMR 400MHz, $CDCl_3$)

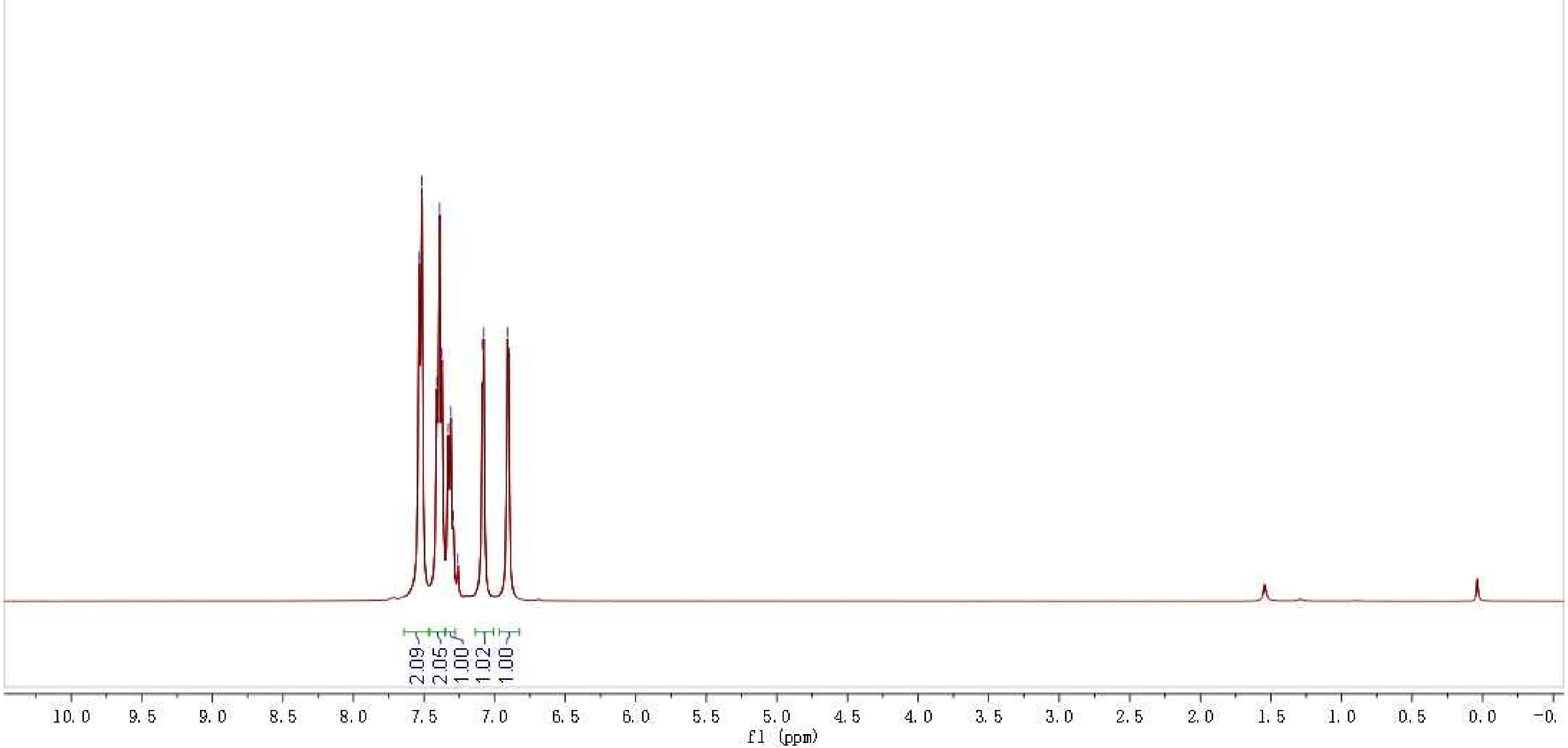

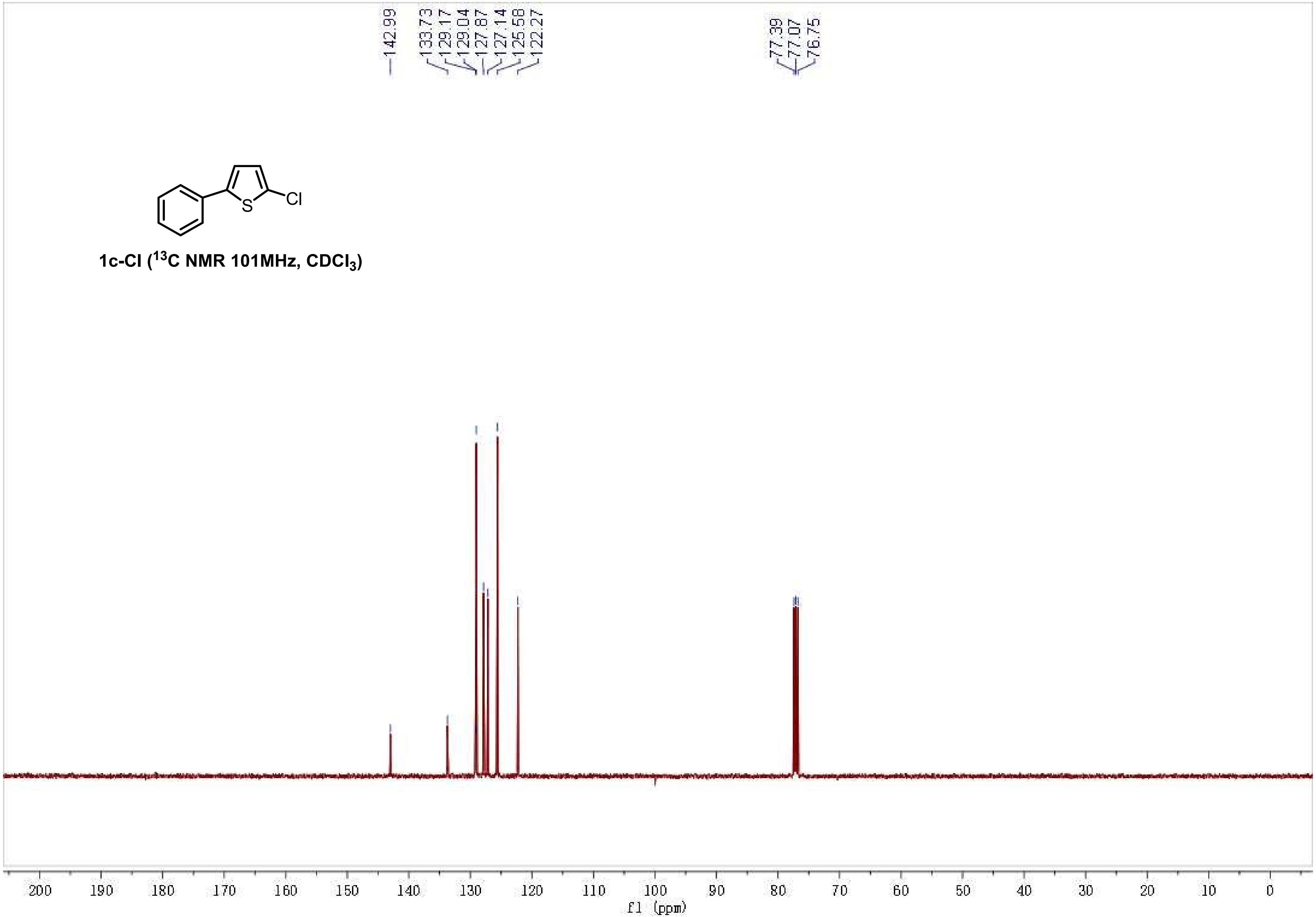

1c-Cl (^{13}C NMR 101MHz, $CDCl_3$)

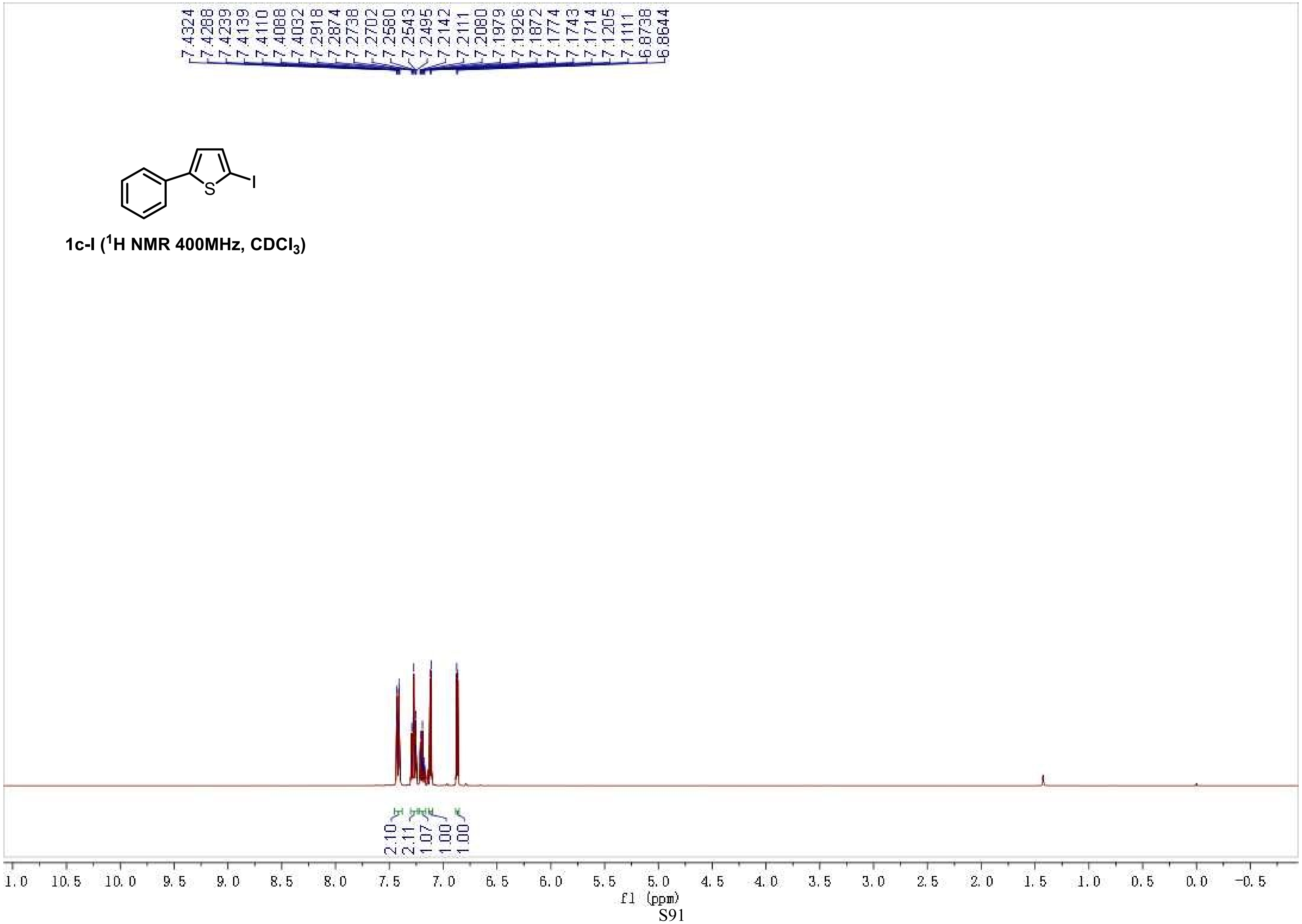

1c-I (^1H NMR 400MHz, $CDCl_3$)

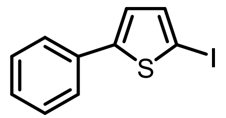

1c-I (^{13}C NMR 101MHz, $CDCl_3$)

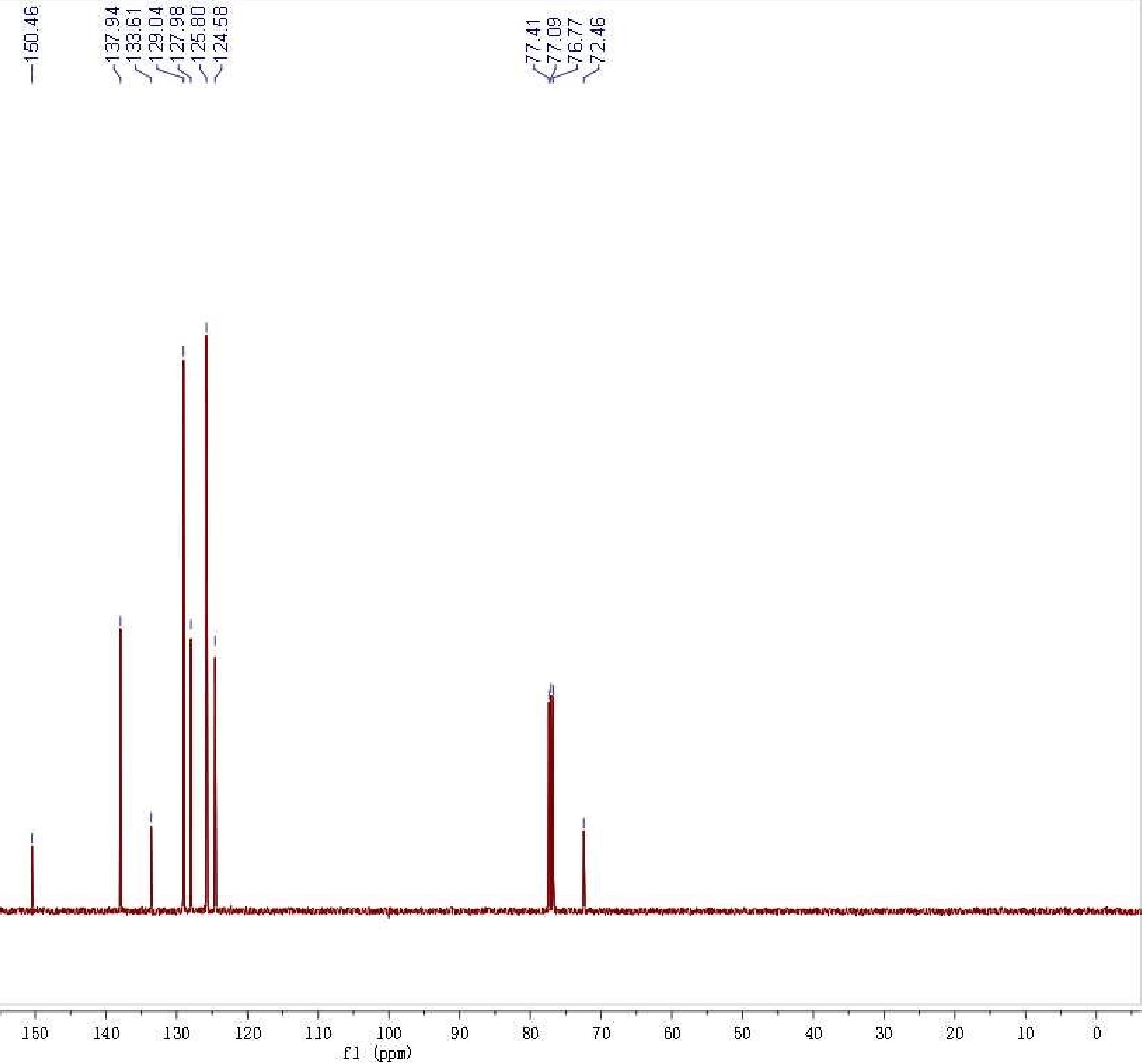

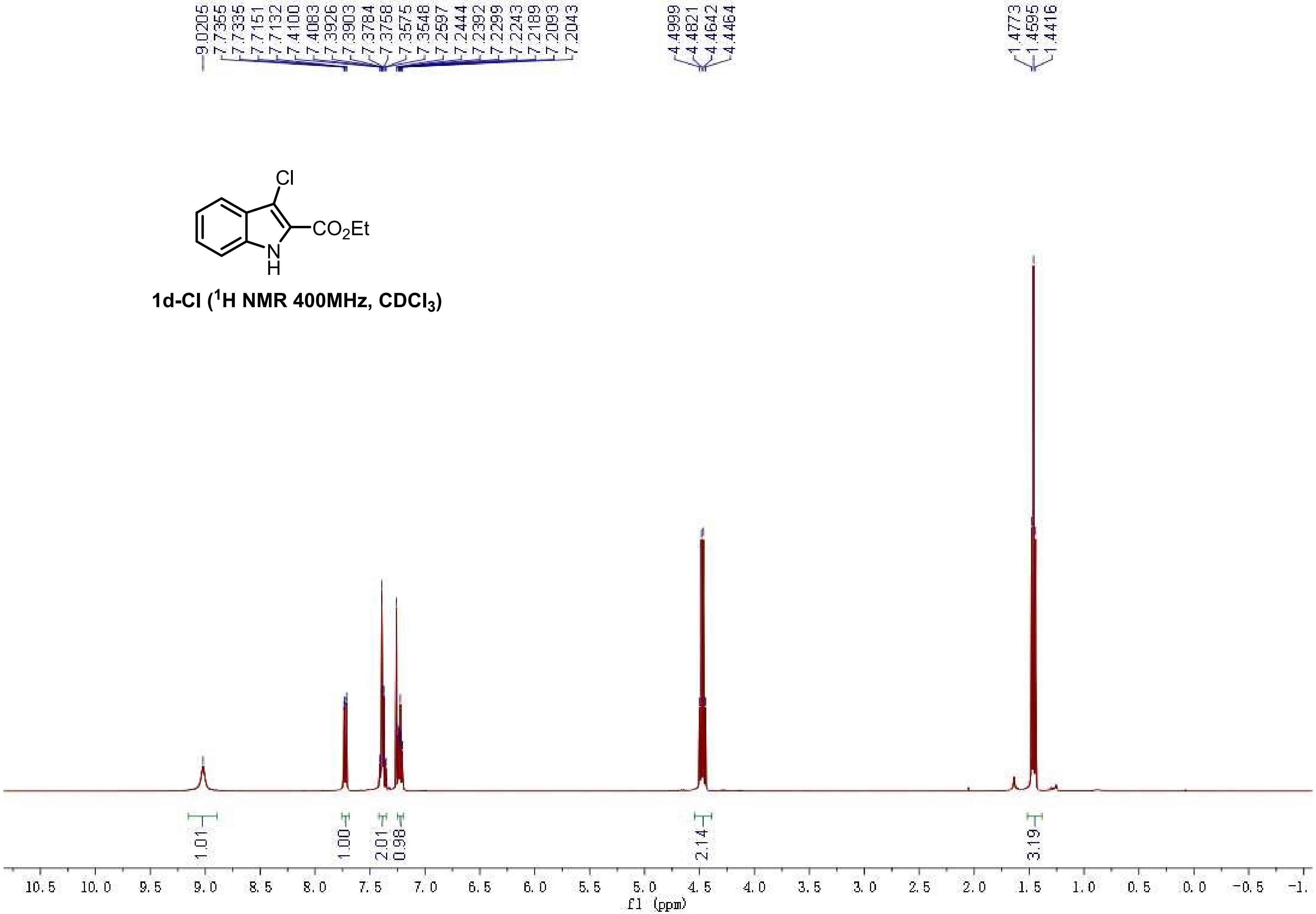

1d-Cl (^{1}H NMR 400MHz, $CDCl_3$)

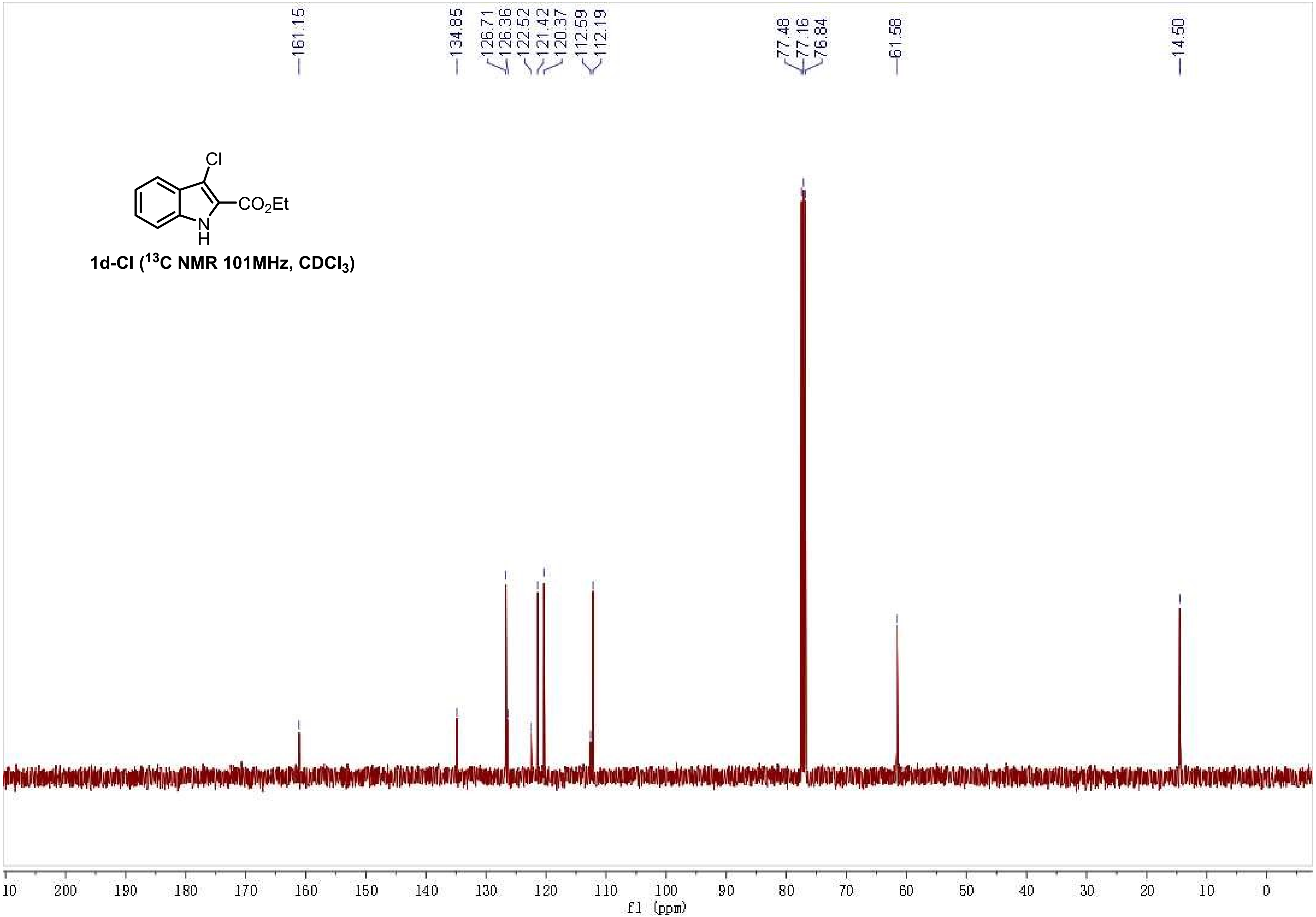

1d-Cl (^{13}C NMR 101MHz, $CDCl_3$)

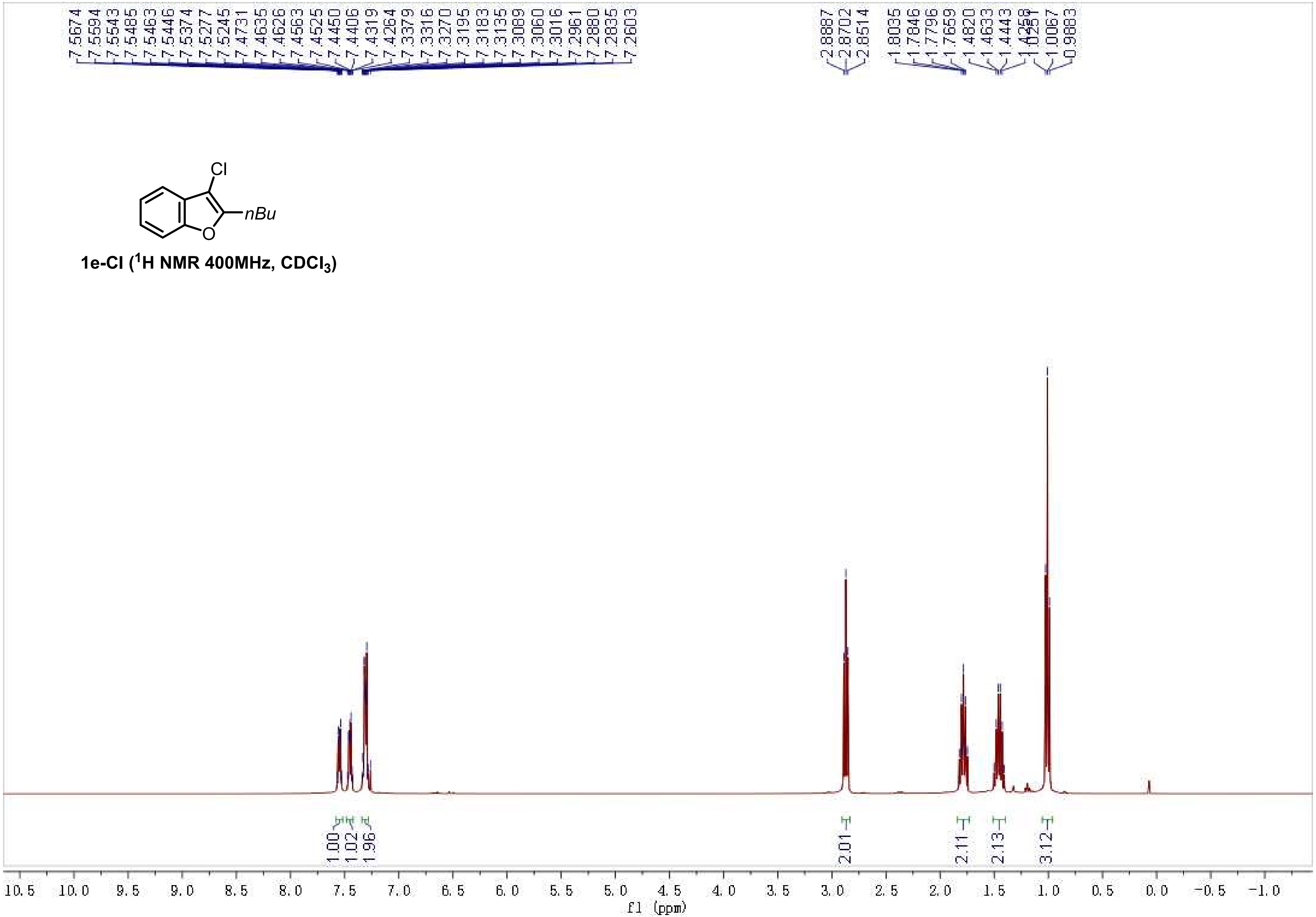

1e-Cl (^{1}H NMR 400MHz, $CDCl_3$)

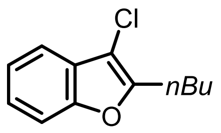

1e-Cl (^{13}C NMR 101MHz, $CDCl_3$)

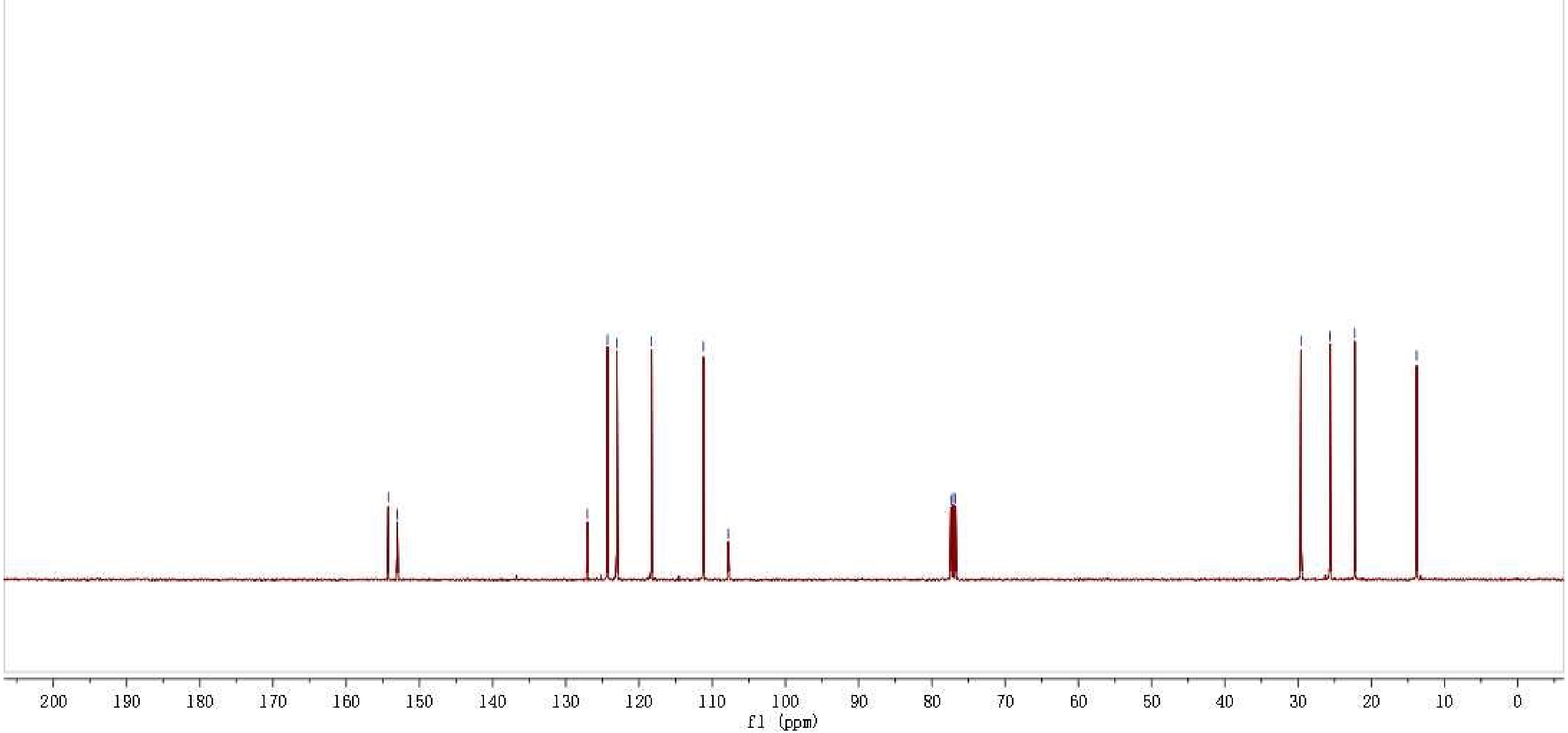

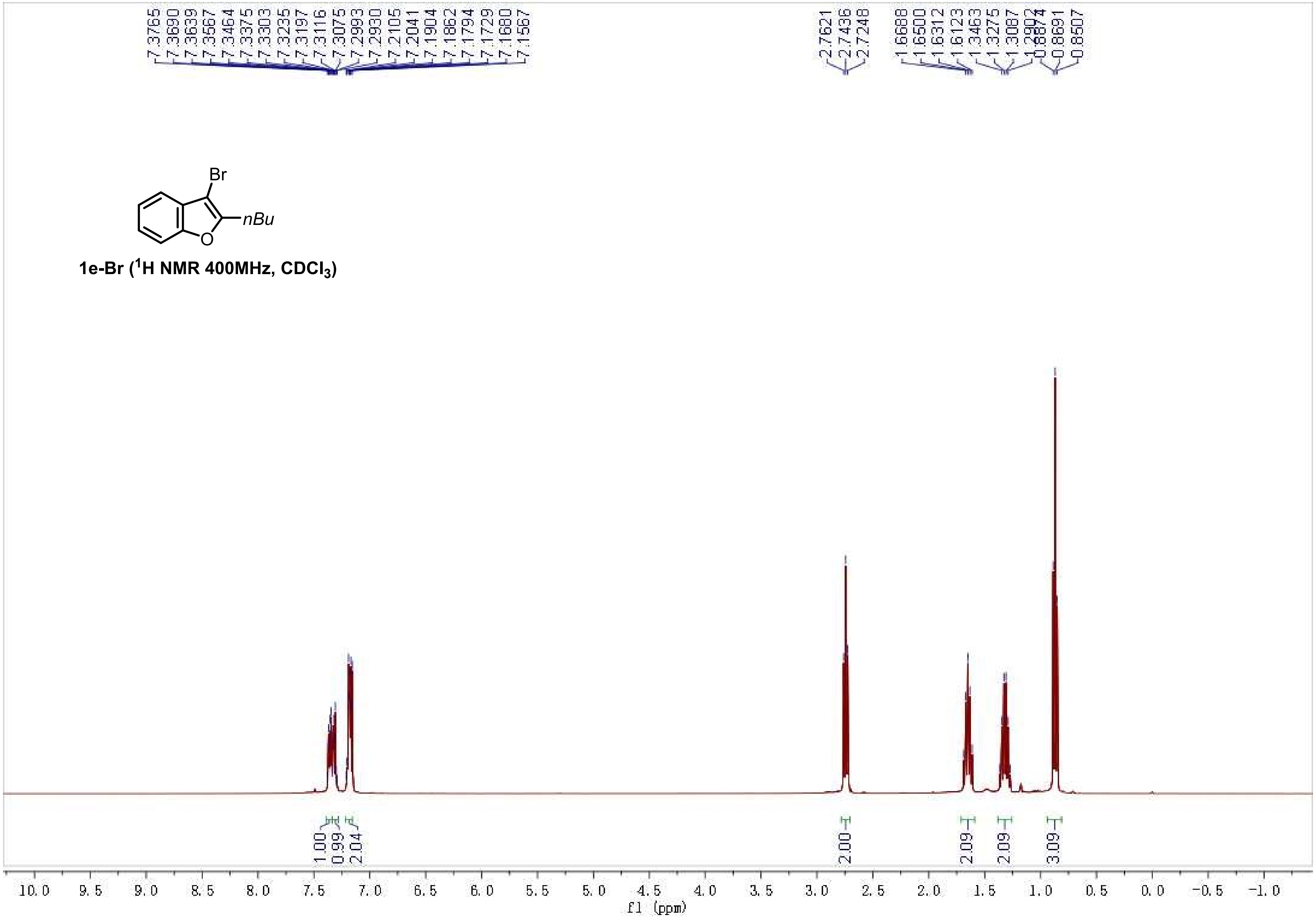

1e-Br (^{1}H NMR 400MHz, $CDCl_3$)

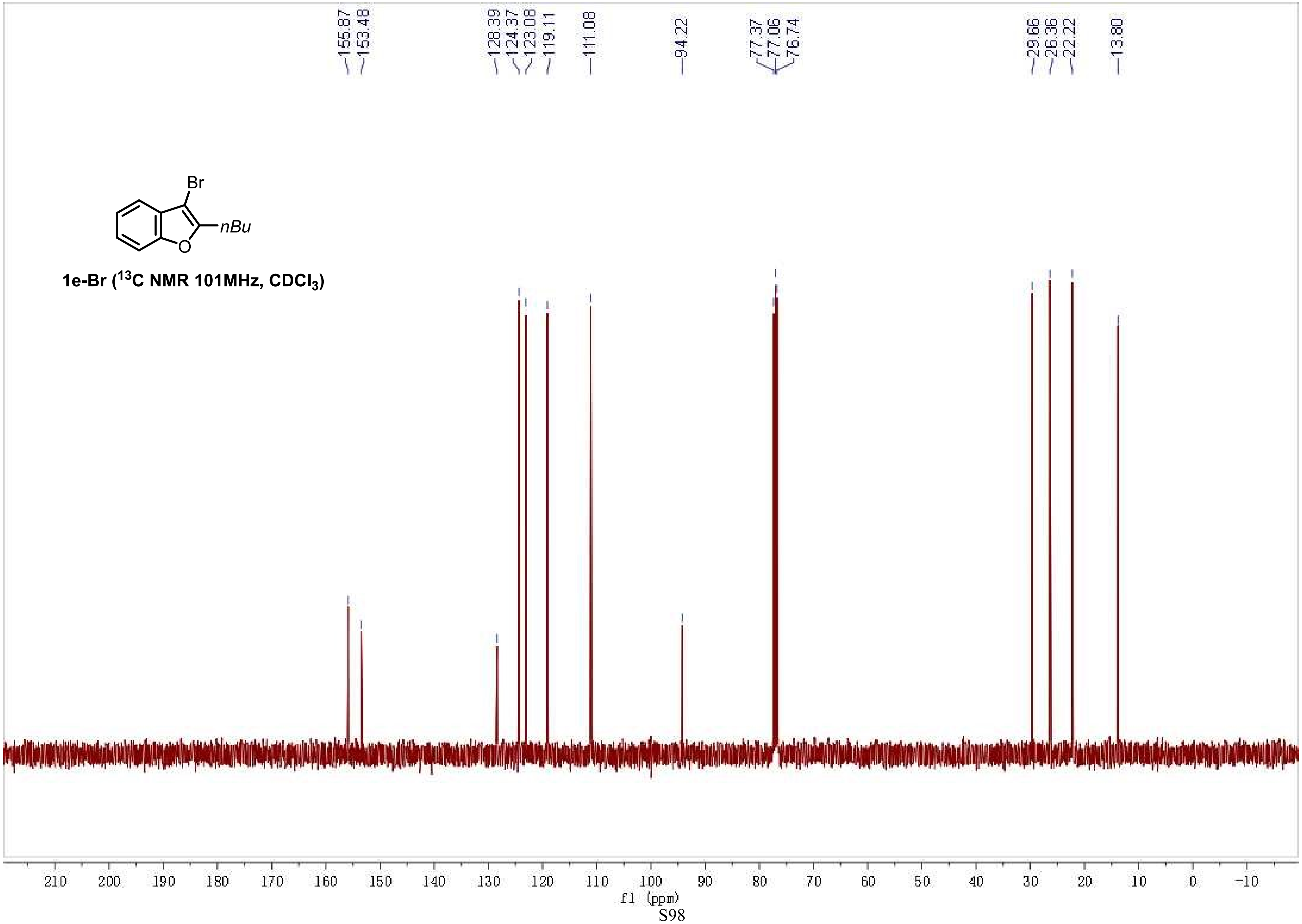

1e-Br (^{13}C NMR 101MHz, $CDCl_3$)

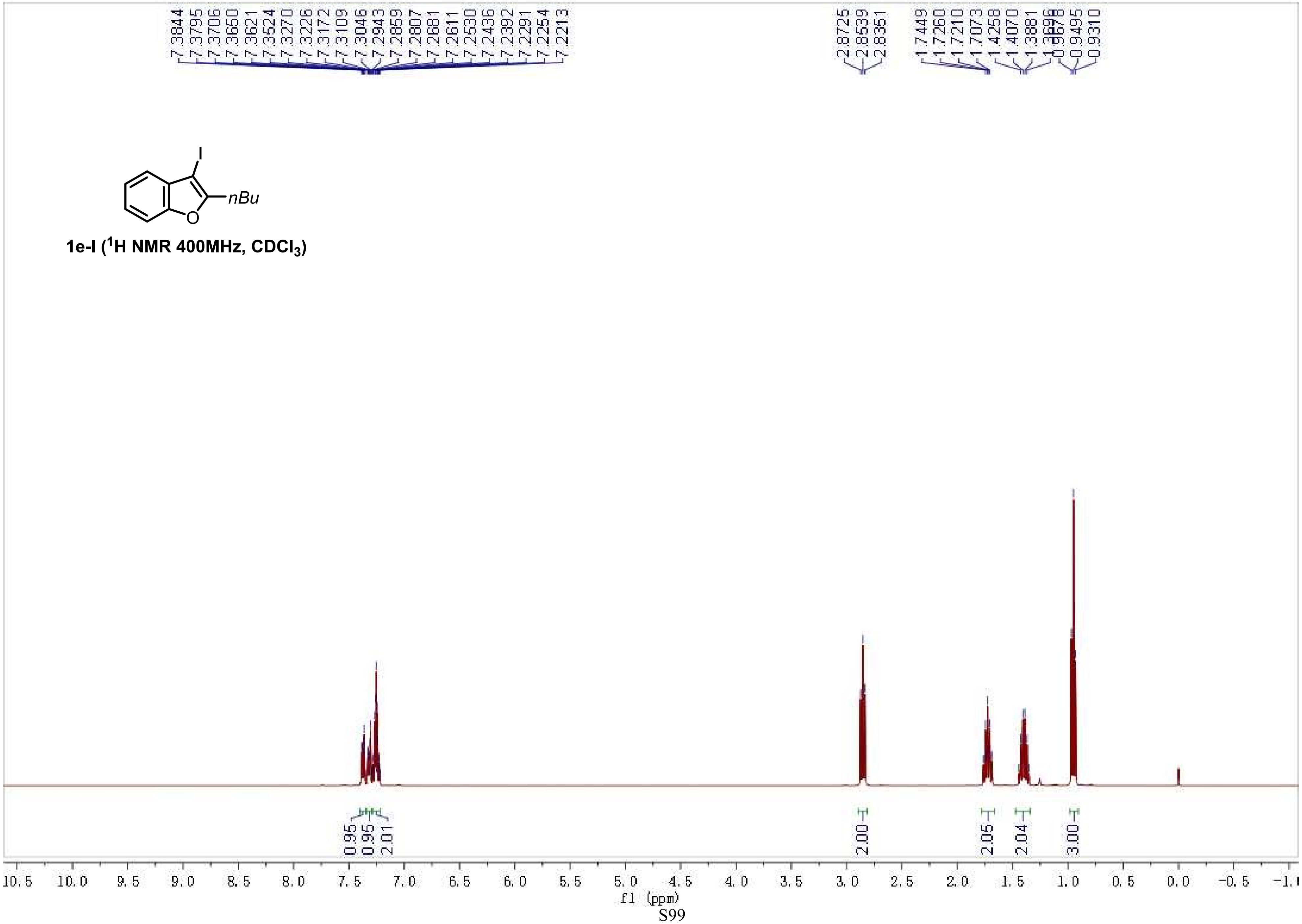
7.3844
7.3795
7.3706
7.3650
7.3621
7.3524
7.3270
7.3226
7.3172
7.3109
7.3046
7.2943
7.2859
7.2807
7.2681
7.2611
7.2530
7.2436
7.2392
7.2291
7.2254
7.2213
2.8725
2.8539
2.8351
1.7449
1.7260
1.7210
1.7073
1.4258
1.4070
1.3881
1.3696
0.9678
0.9495
0.9310
I
nBu
O
1e-I (^{1}H NMR 400MHz, $CDCl_3$)
0.95
0.95
2.01
2.00
2.05
2.04
3.00
10.5 10.0 9.5 9.0 8.5 8.0 7.5 7.0 6.5 6.0 5.5 5.0 4.5 4.0 3.5 3.0 2.5 2.0 1.5 1.0 0.5 0.0 -0.5 -1.0
f1 (ppm)

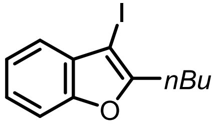

1e-I (13C NMR 101MHz, CDCl$_3$)

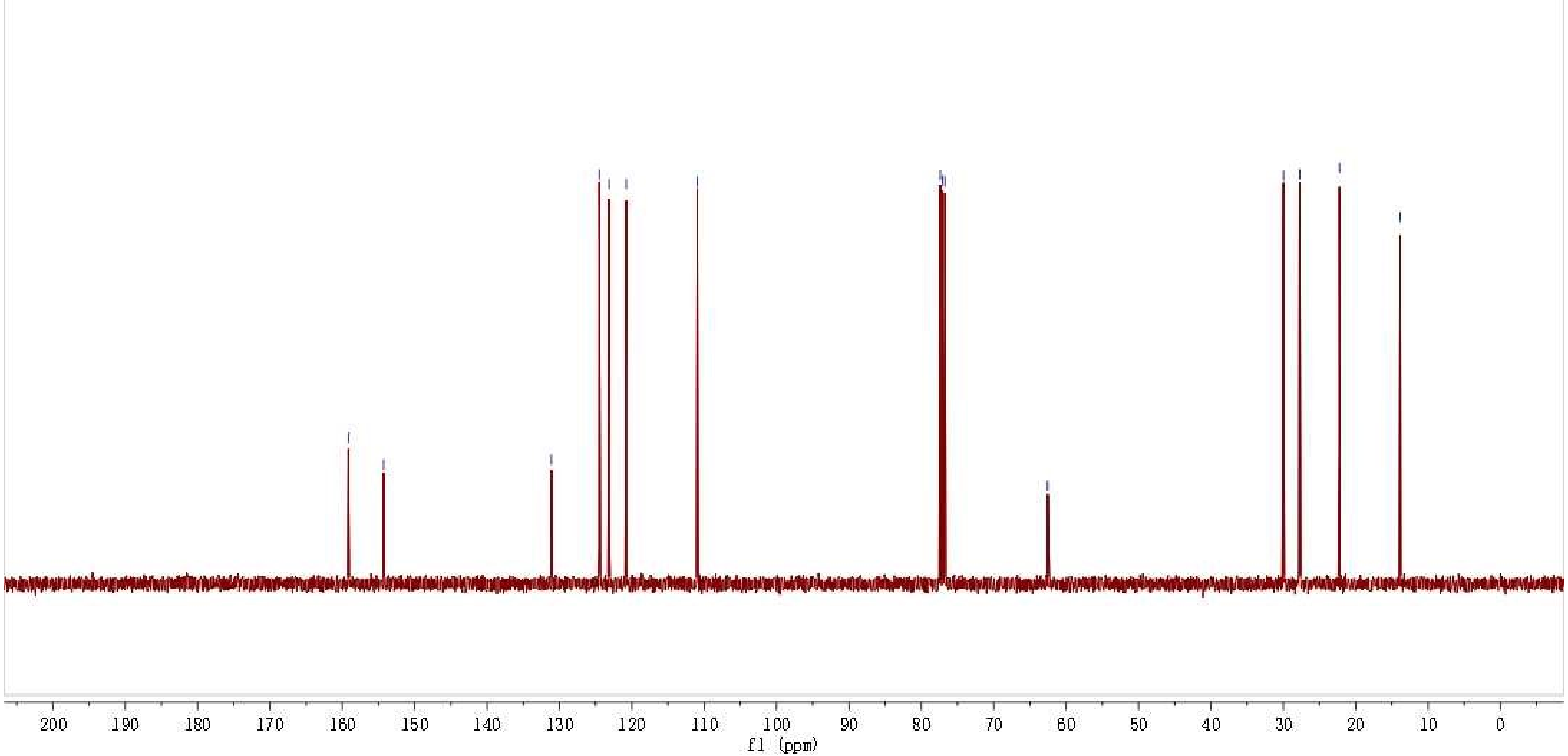

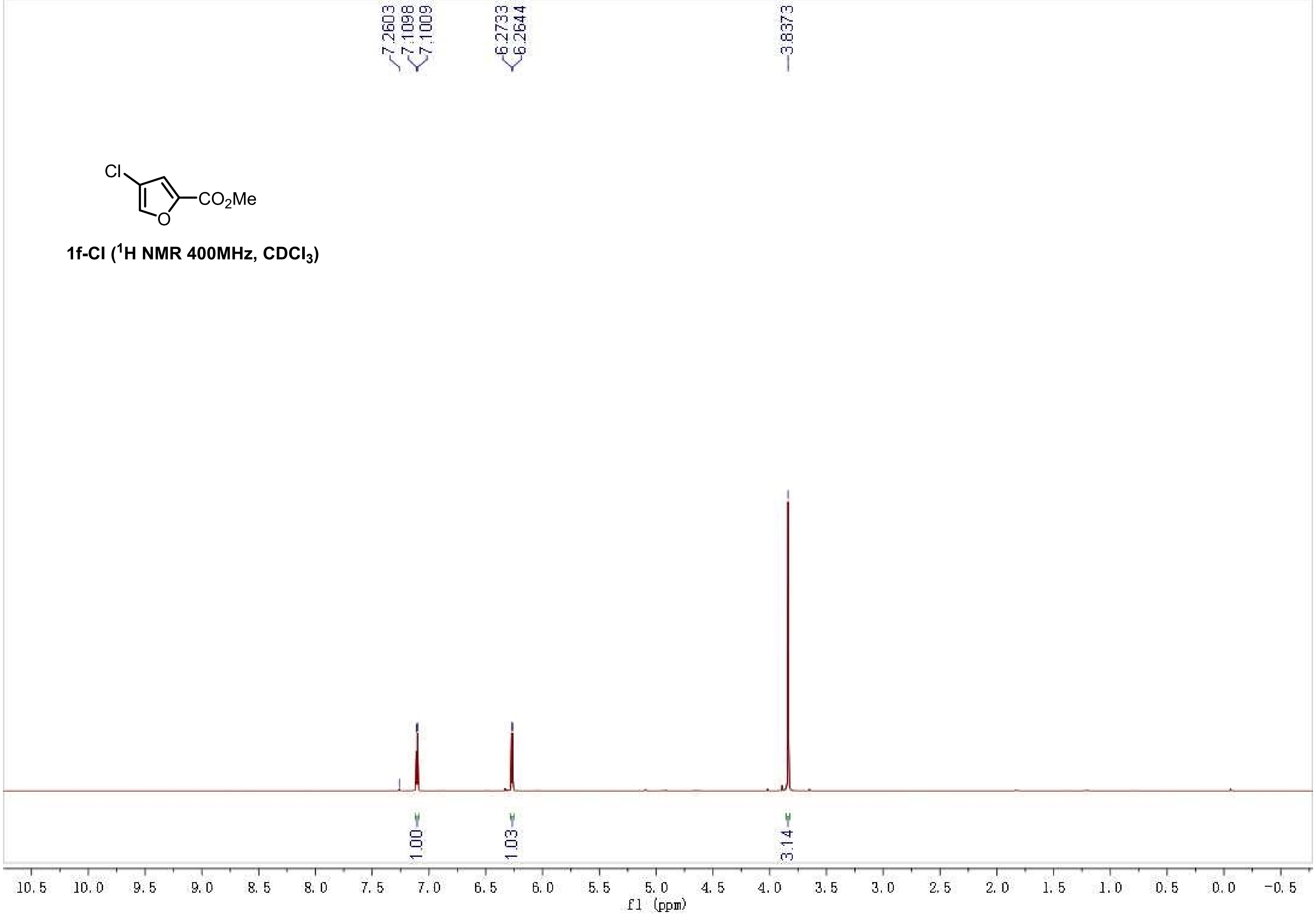

1f-Cl (^1H NMR 400MHz, $CDCl_3$)

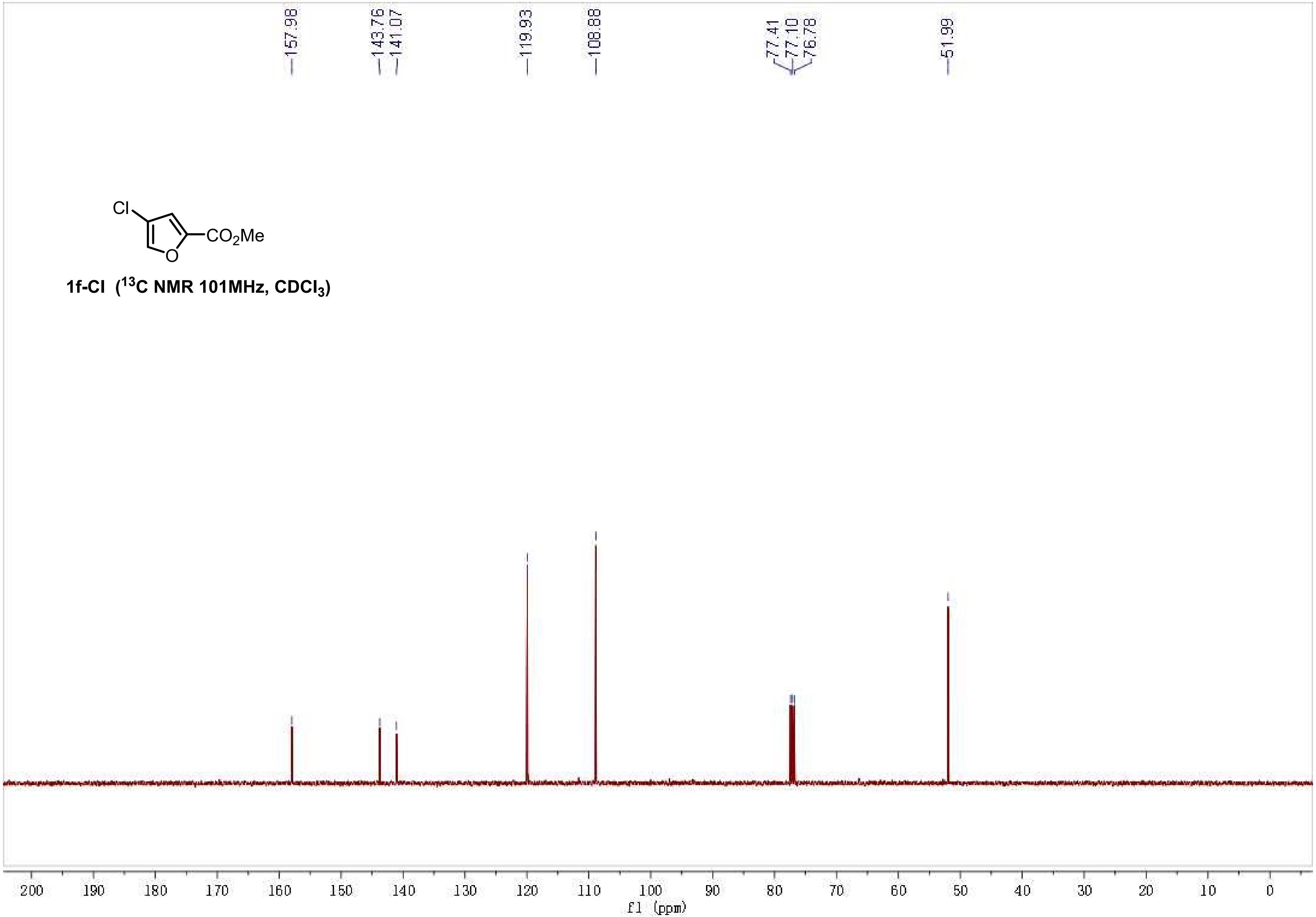

1f-Cl (^{13}C NMR 101MHz, $CDCl_3$)

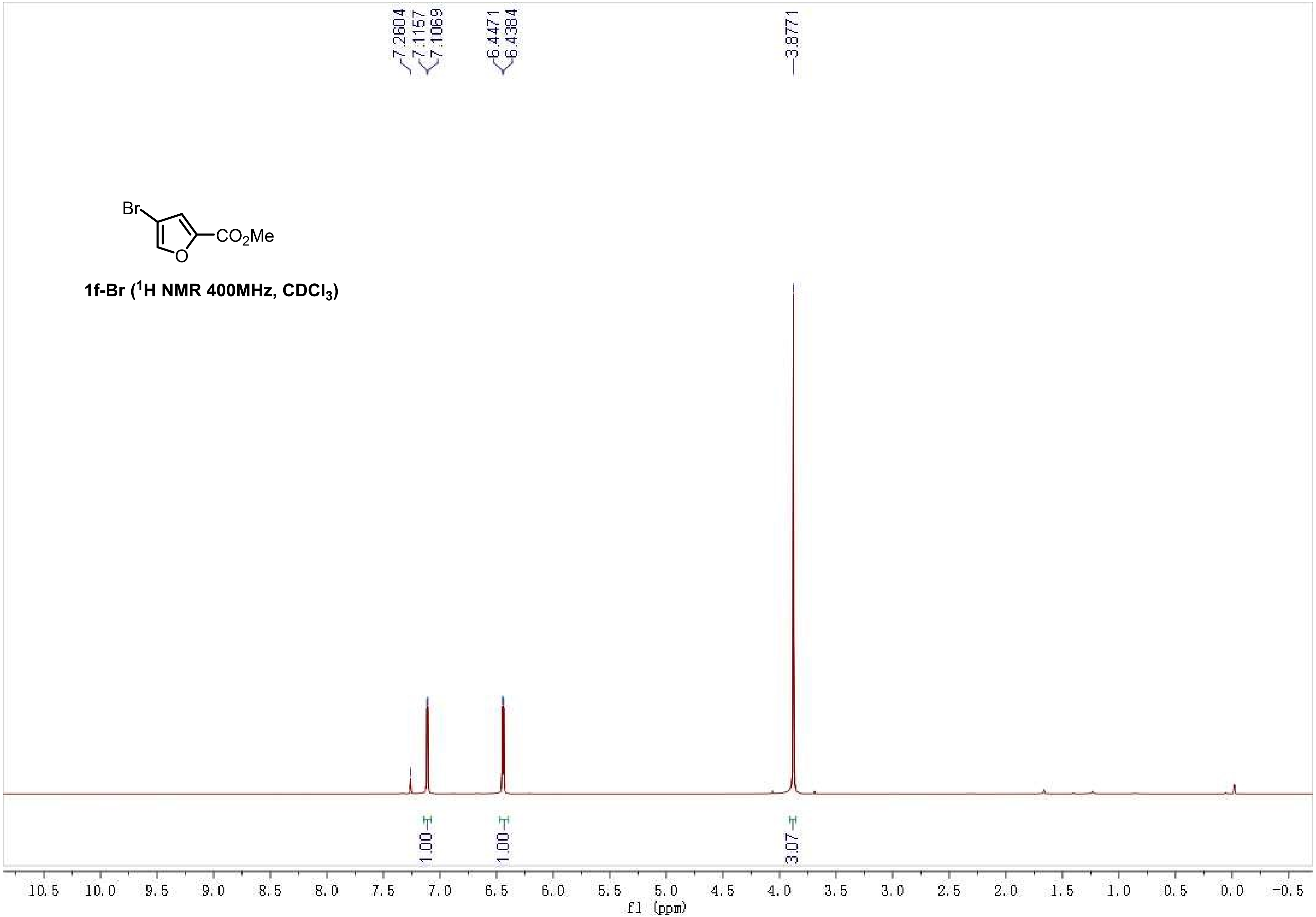

7.2604
7.1157
7.1069
6.4471
6.4384
3.8771
Br
CO2Me
O
1f-Br (1H NMR 400MHz, CDCl3)
1.00
1.00
3.07
10.5 10.0 9.5 9.0 8.5 8.0 7.5 7.0 6.5 6.0 5.5 5.0 4.5 4.0 3.5 3.0 2.5 2.0 1.5 1.0 0.5 0.0 -0.5
f1 (ppm)

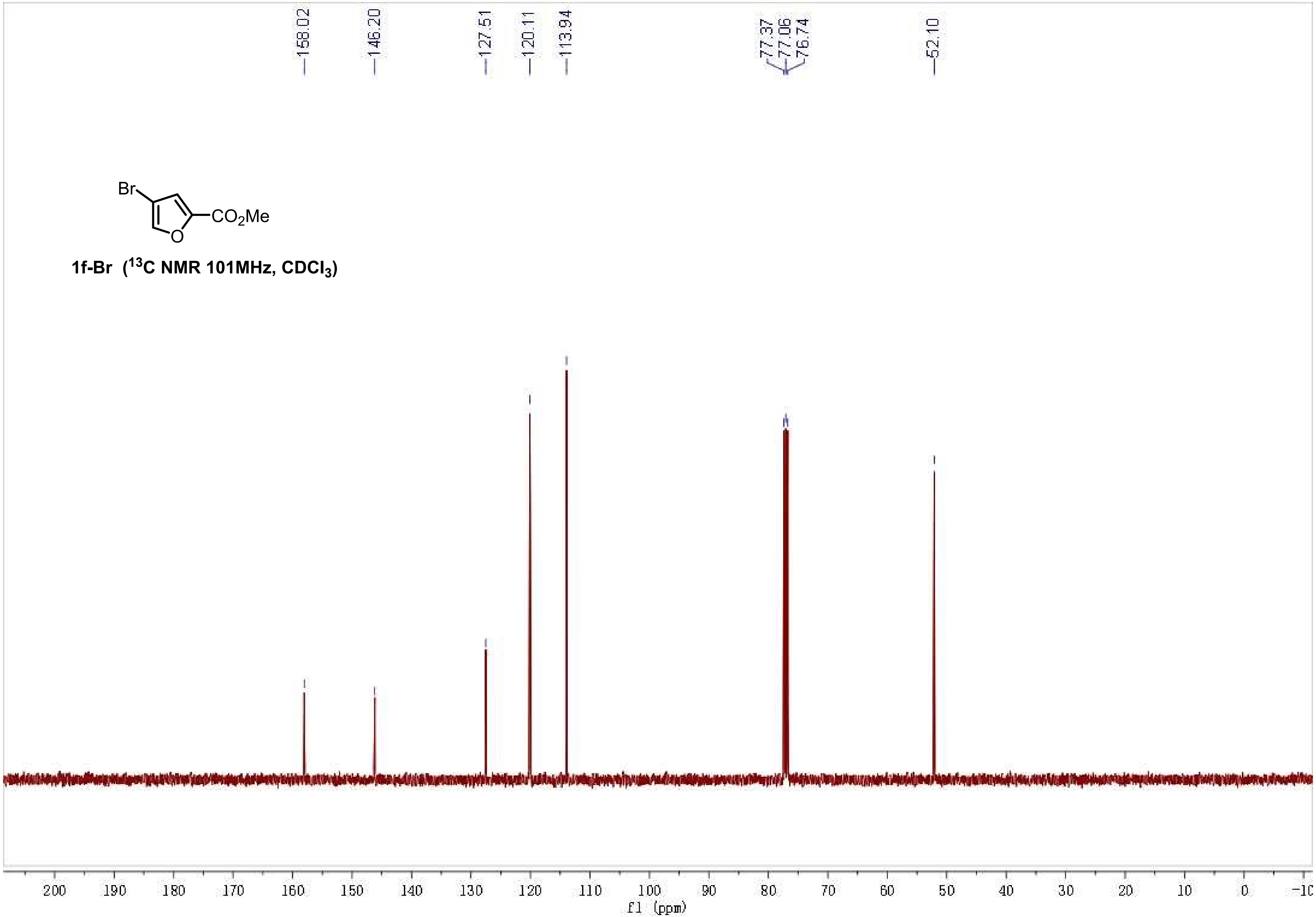

1f-Br (^{13}C NMR 101MHz, $CDCl_3$)

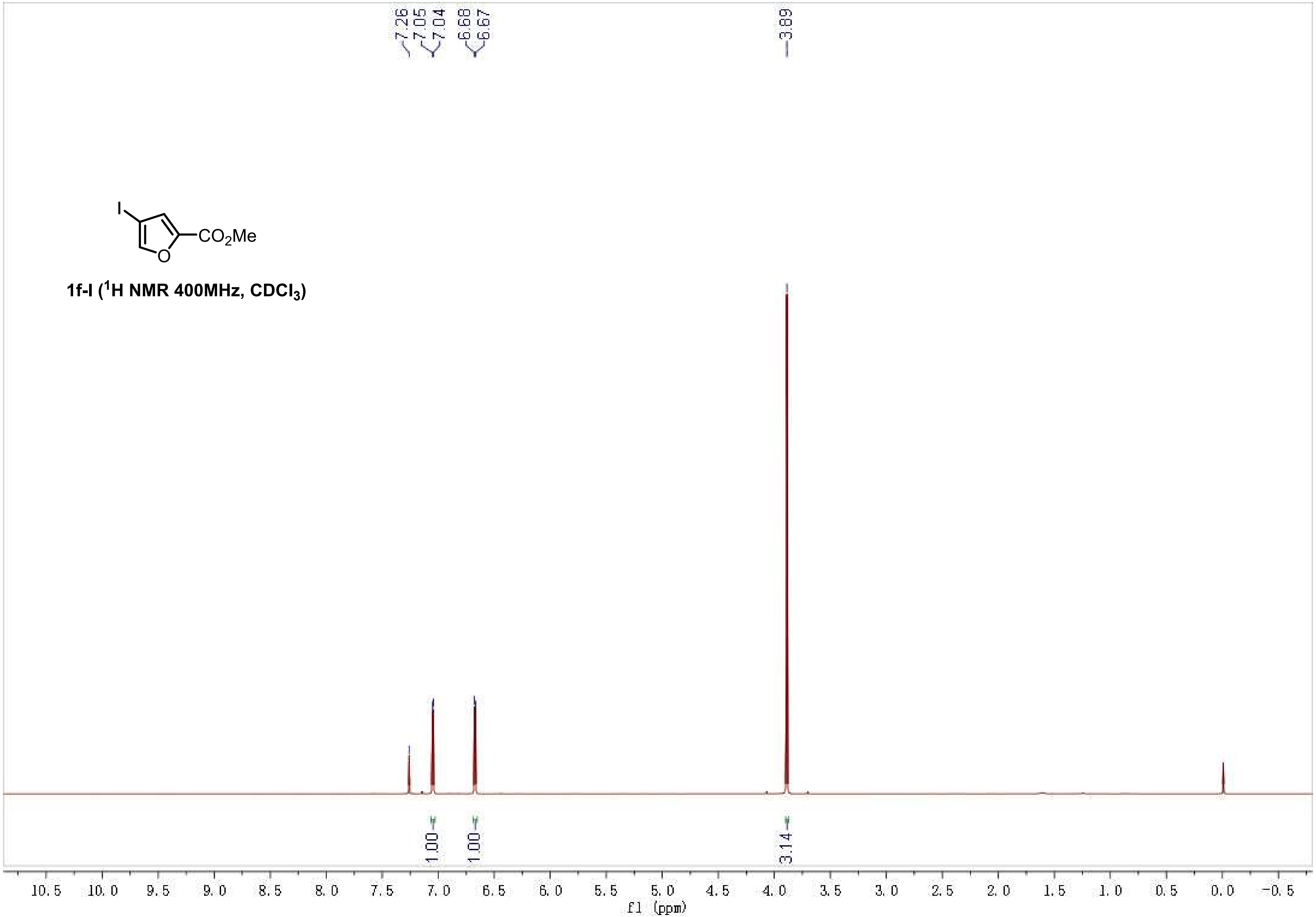

1f-I (^{1}H NMR 400MHz, $CDCl_3$)

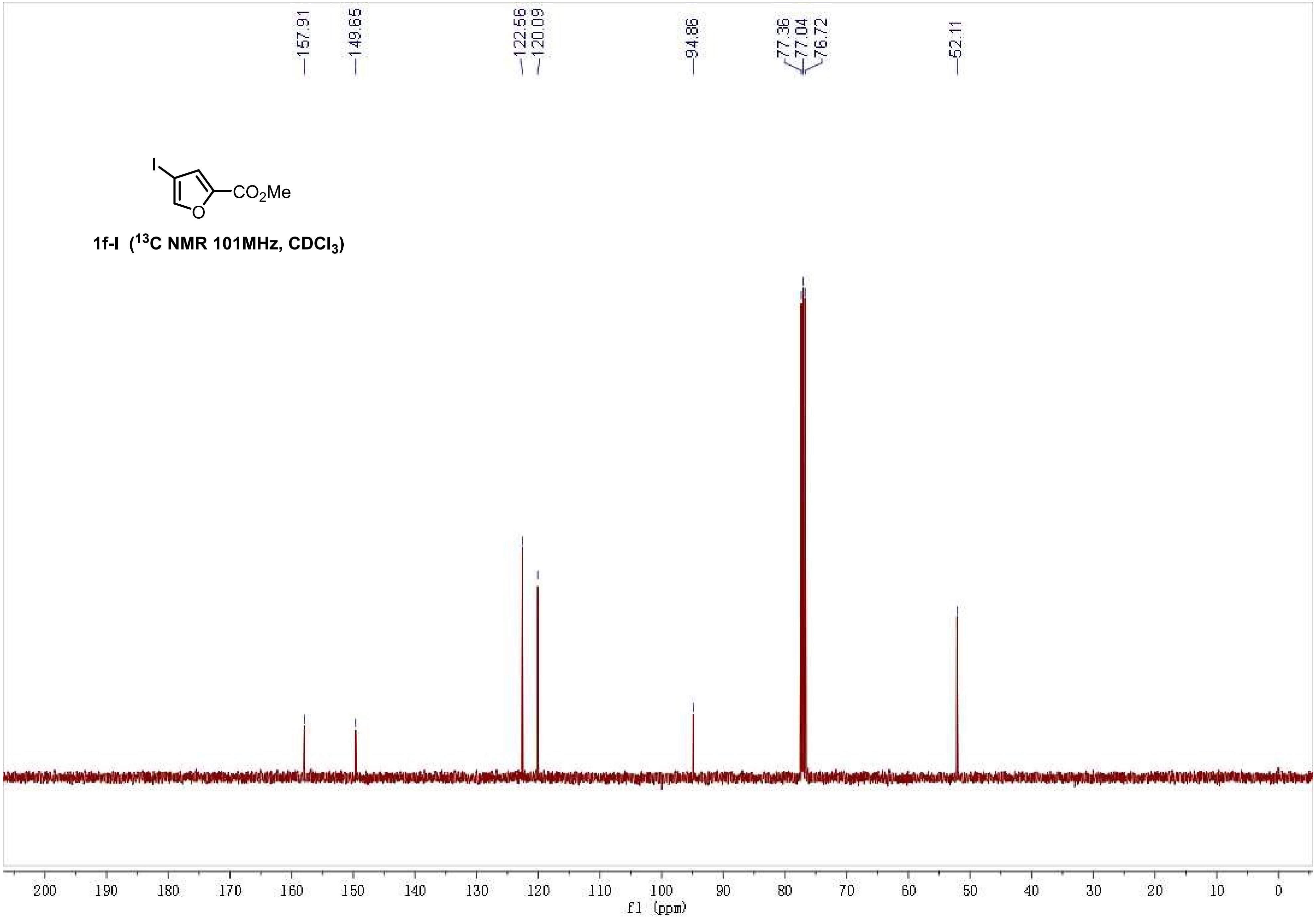

1f-I (^{13}C NMR 101MHz, $CDCl_3$)

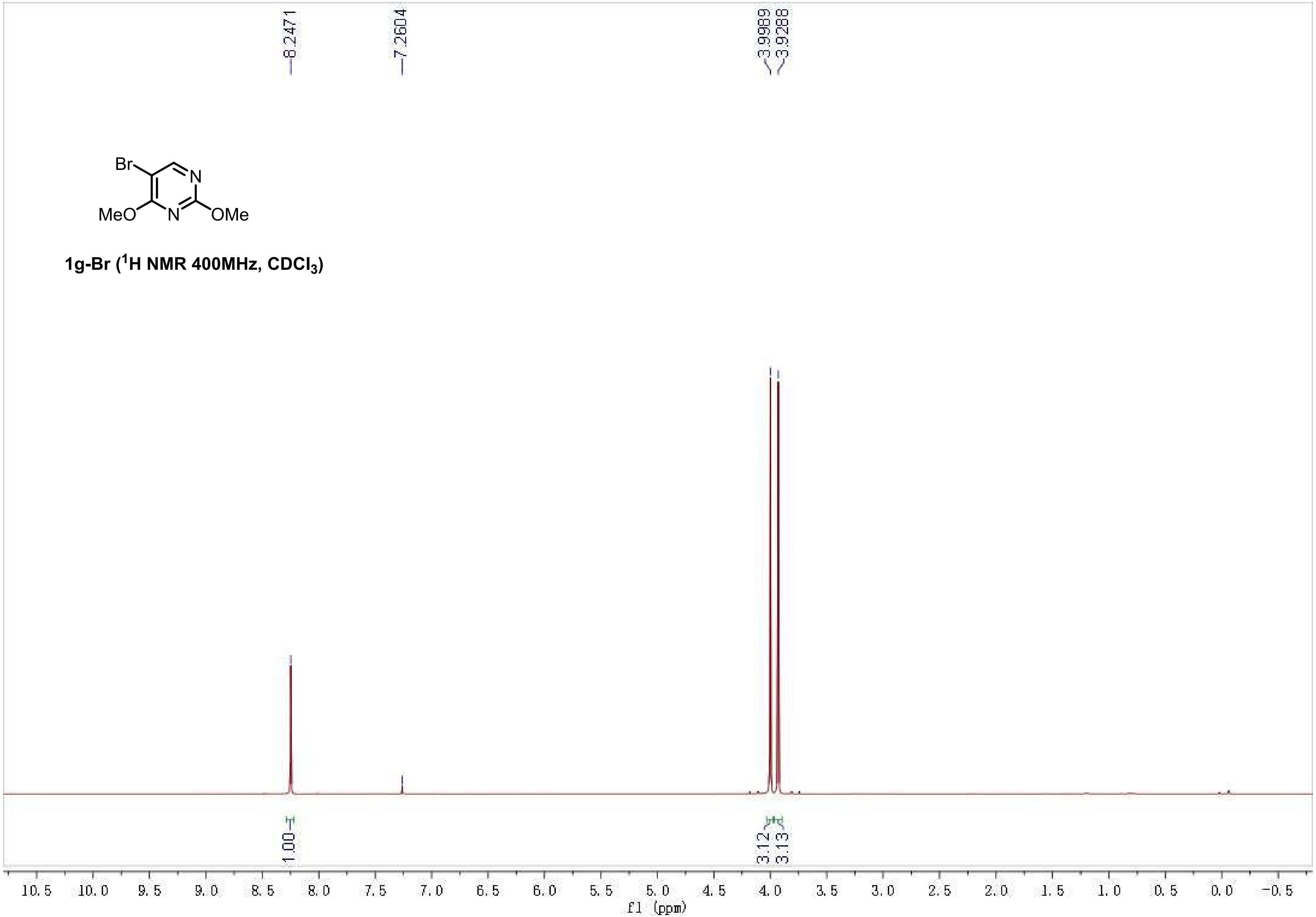

1g-Br (^{1}H NMR 400MHz, $CDCl_3$)

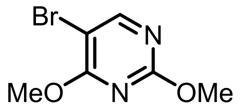

1g- Br (^13^C NMR 101MHz, CDCl$_3$)

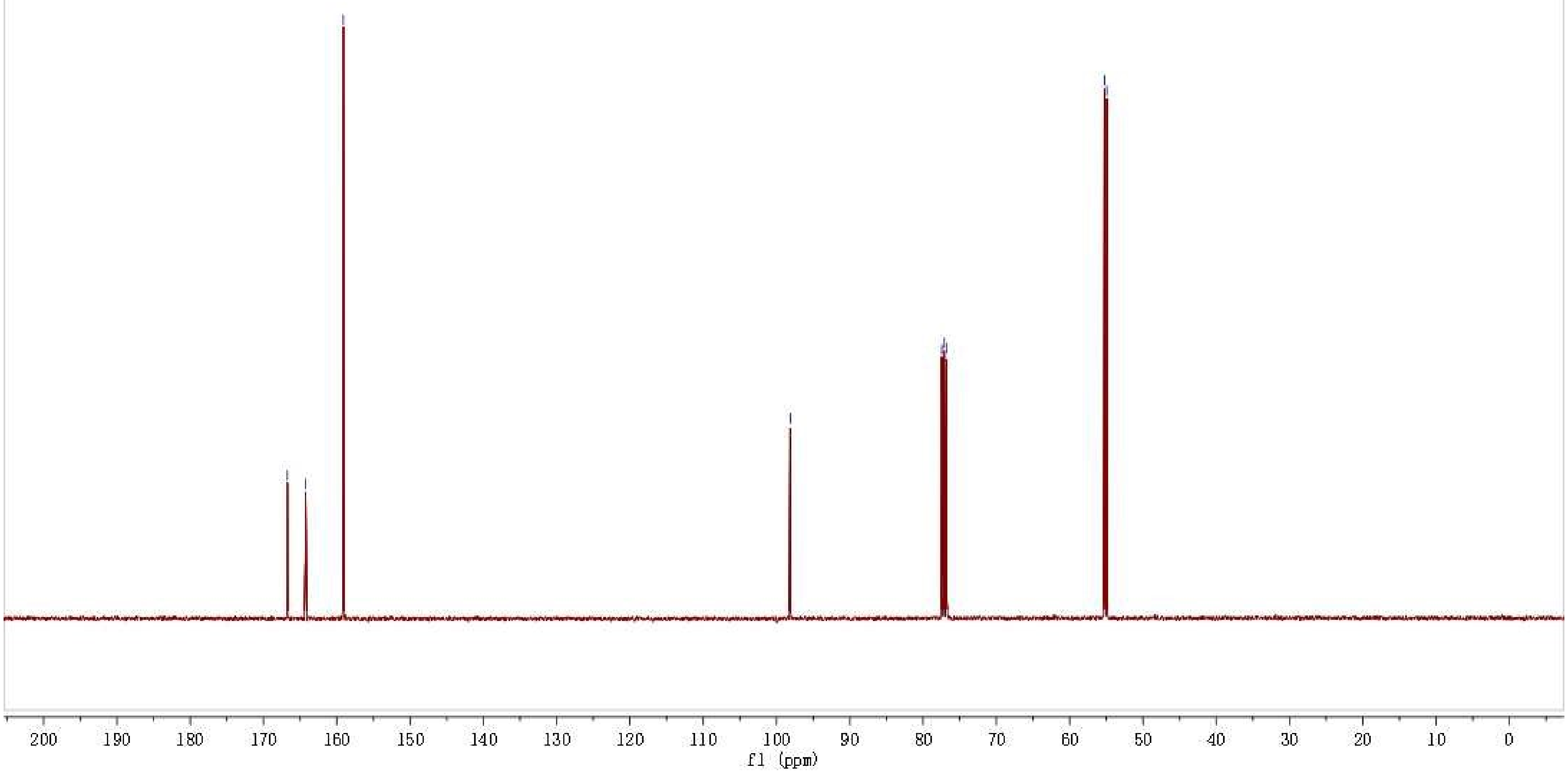

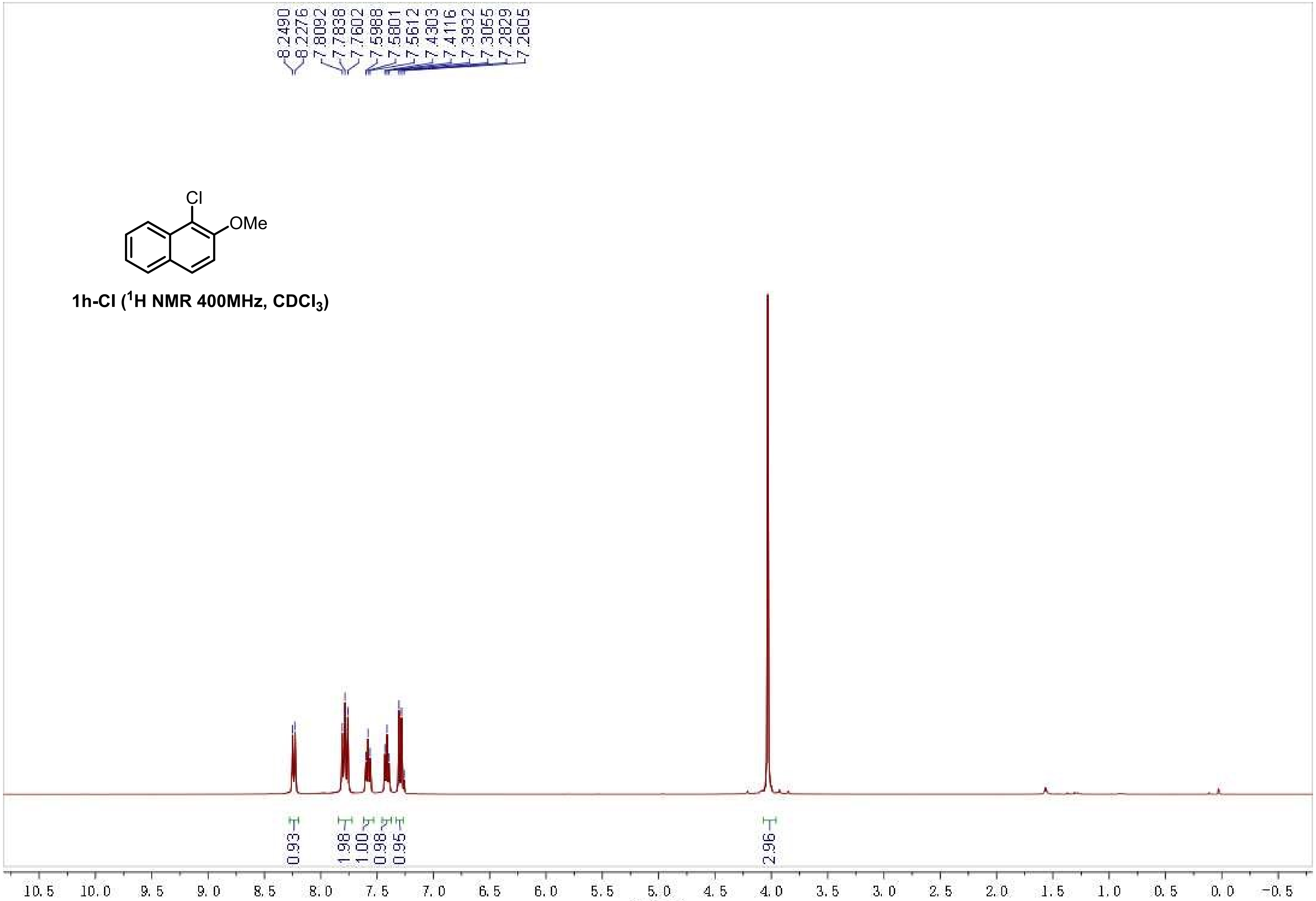

1h-Cl (^{1}H NMR 400MHz, $CDCl_3$)

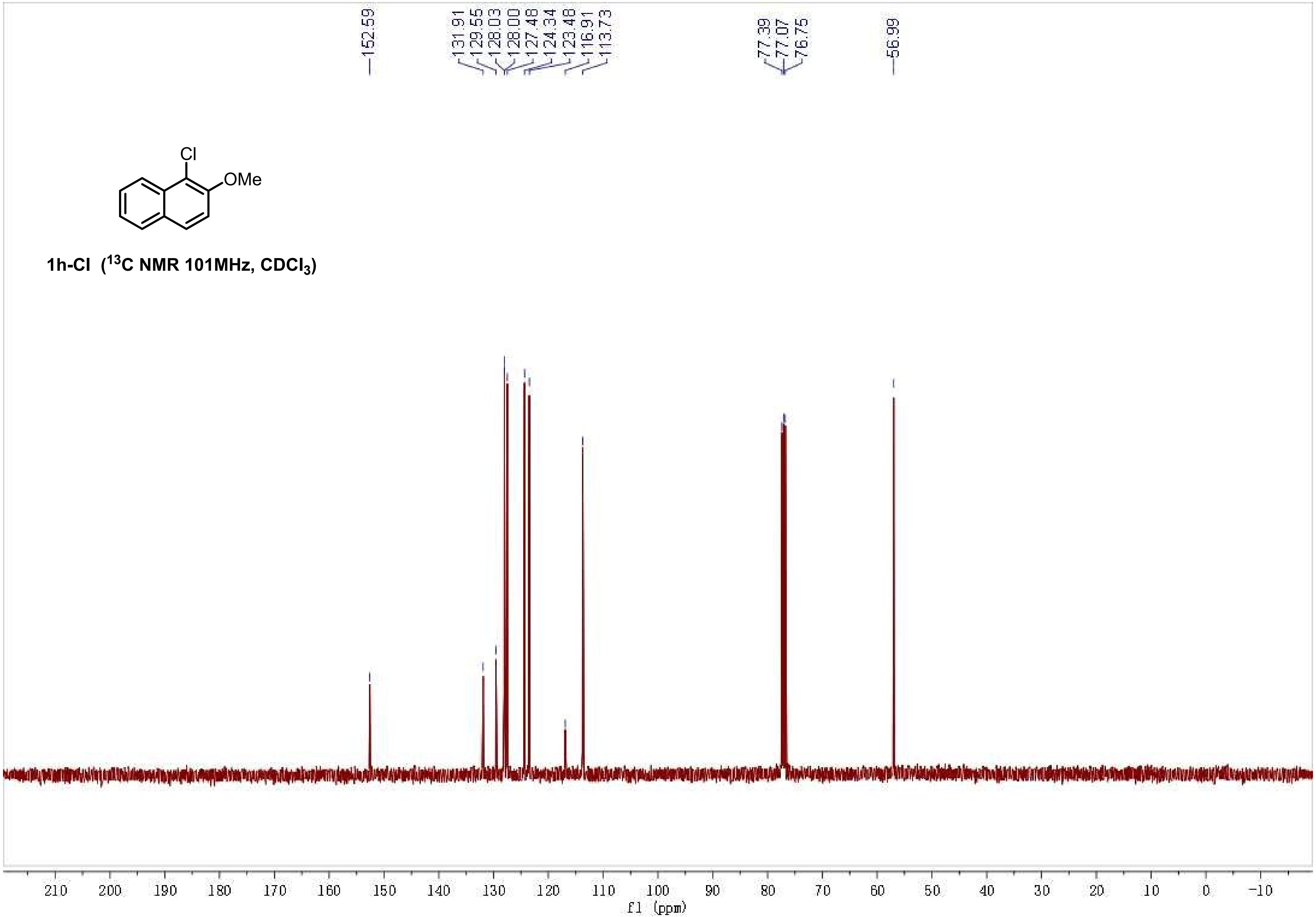

1h-Cl (^{13}C NMR 101MHz, $CDCl_3$)

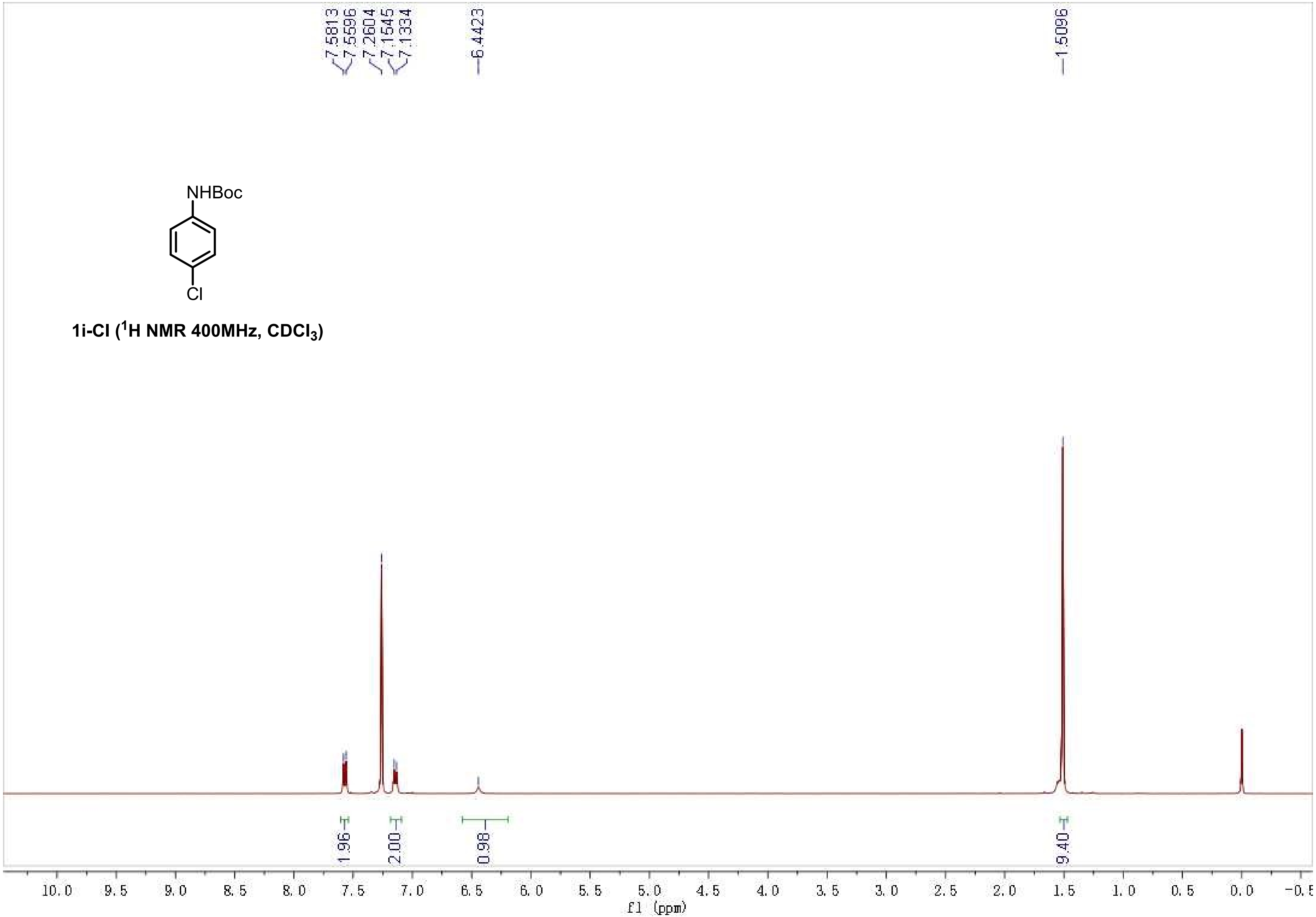
7.5813
7.5596
7.2604
7.1545
7.1334
6.4423
1.5096
NHBoc
Cl
1i-Cl (^{1}H NMR 400MHz, $CDCl_3$)
1.96
2.00
0.98
9.40
10.0 9.5 9.0 8.5 8.0 7.5 7.0 6.5 6.0 5.5 5.0 4.5 4.0 3.5 3.0 2.5 2.0 1.5 1.0 0.5 0.0 -0.5
f1 (ppm)

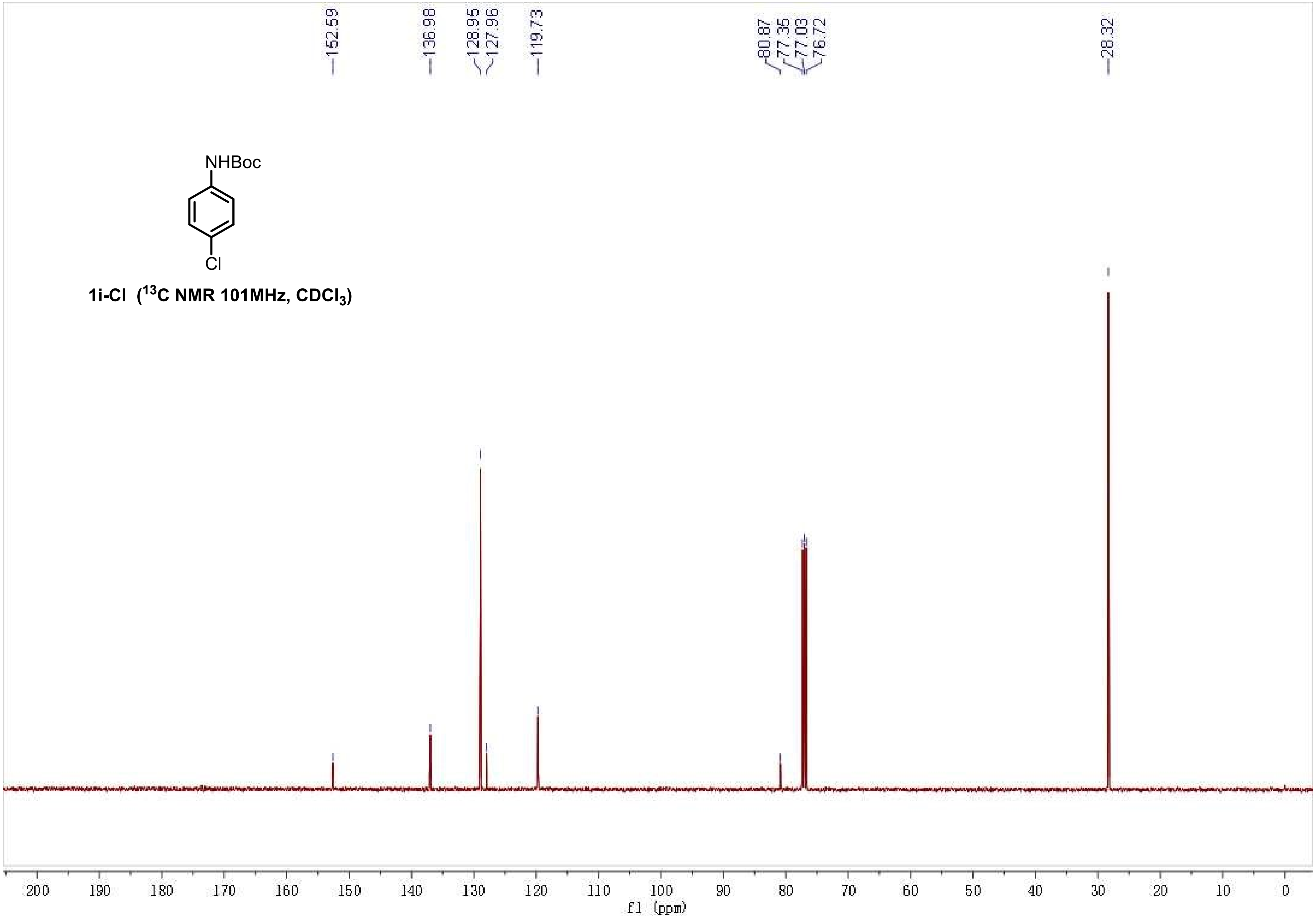

1i-Cl (^{13}C NMR 101MHz, $CDCl_3$)

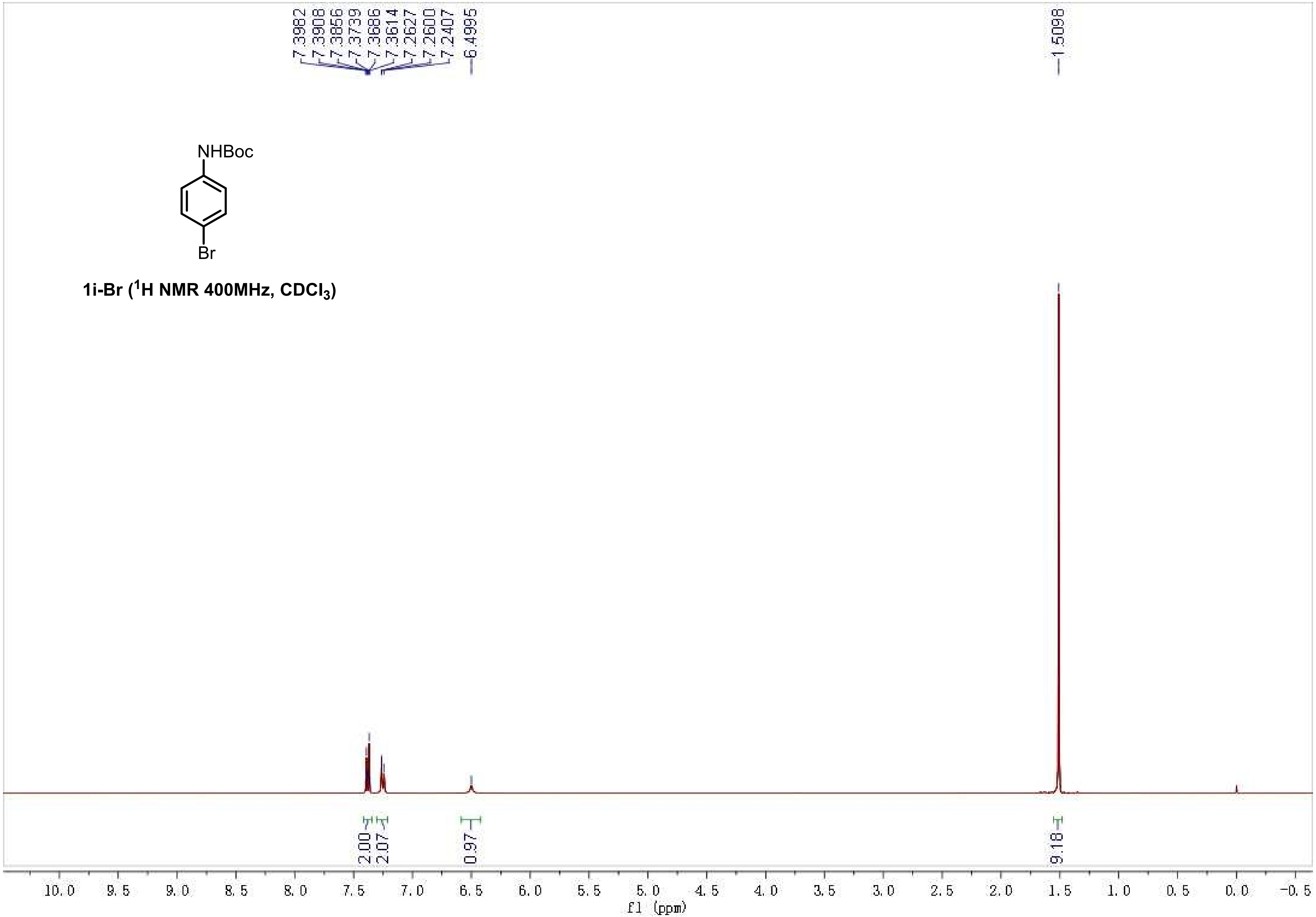
7.3982
7.3908
7.3856
7.3739
7.3686
7.3614
7.2627
7.2600
7.2407
6.4995
1.5098
NHBoc
Br
1i-Br (^{1}H NMR 400MHz, $CDCl_3$)
2.00
2.07
0.97
9.18
10.0 9.5 9.0 8.5 8.0 7.5 7.0 6.5 6.0 5.5 5.0 4.5 4.0 3.5 3.0 2.5 2.0 1.5 1.0 0.5 0.0 -0.5
f1 (ppm)

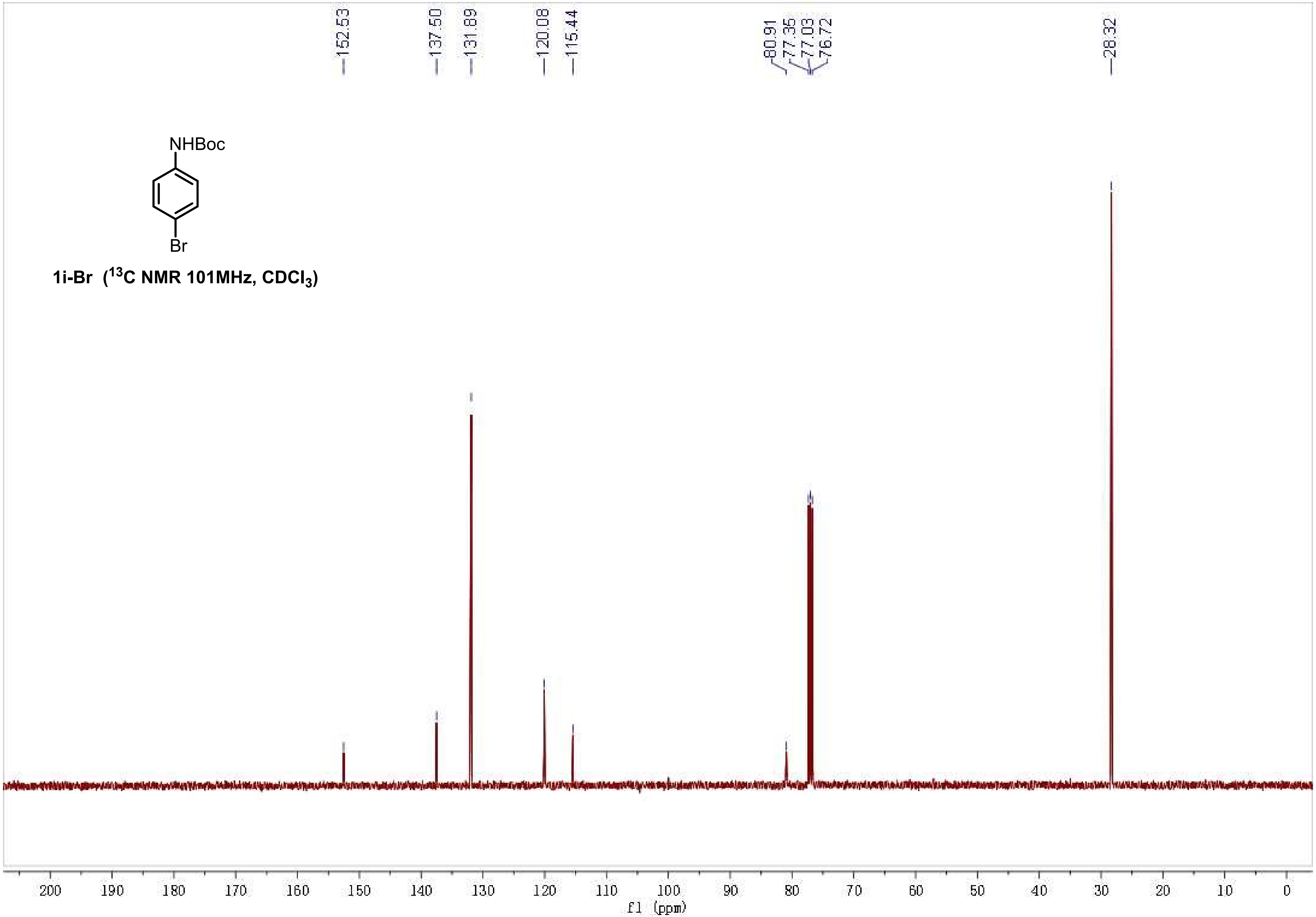

1i-Br (^{13}C NMR 101MHz, $CDCl_3$)

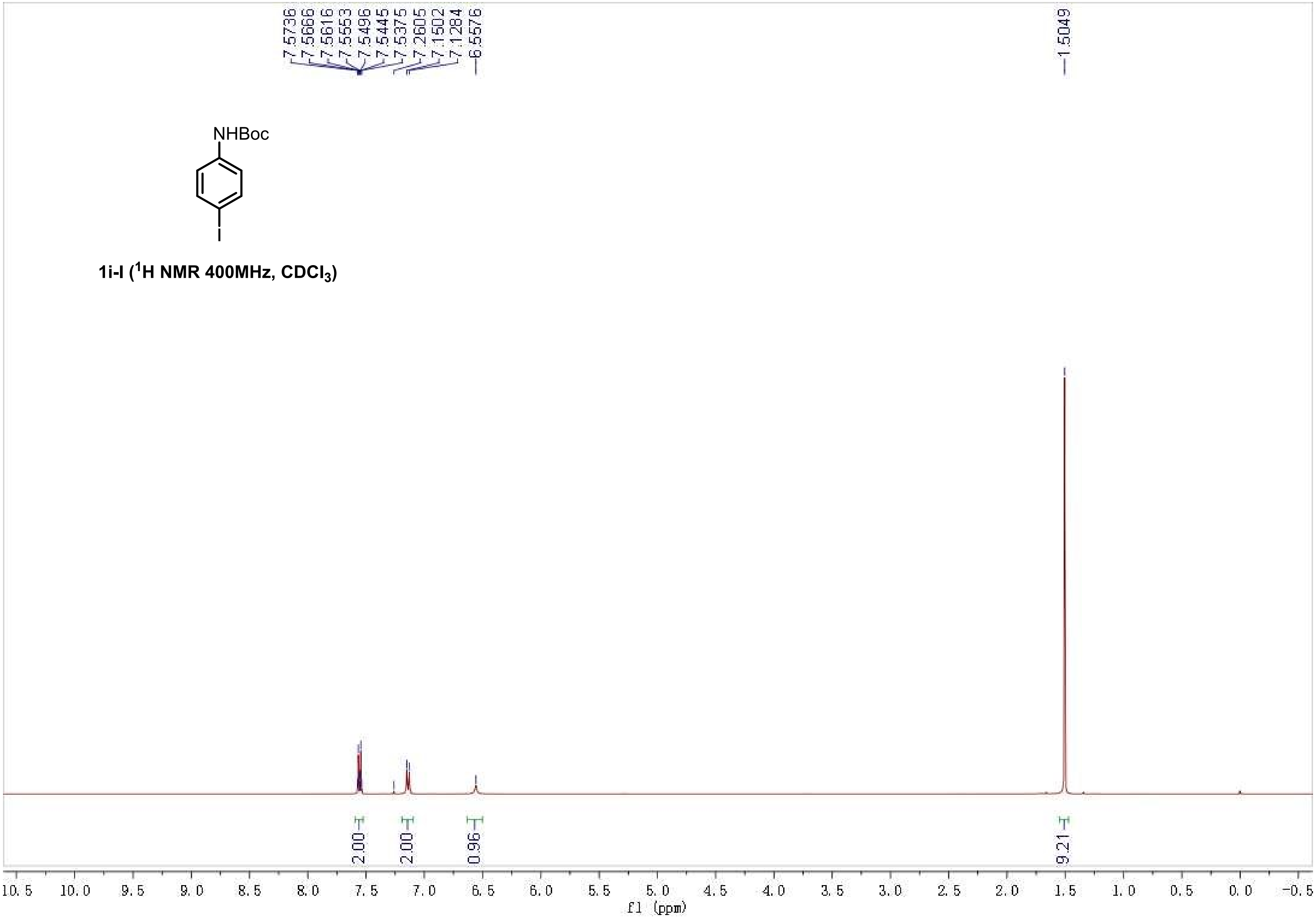

1i-I (^{1}H NMR 400MHz, $CDCl_3$)

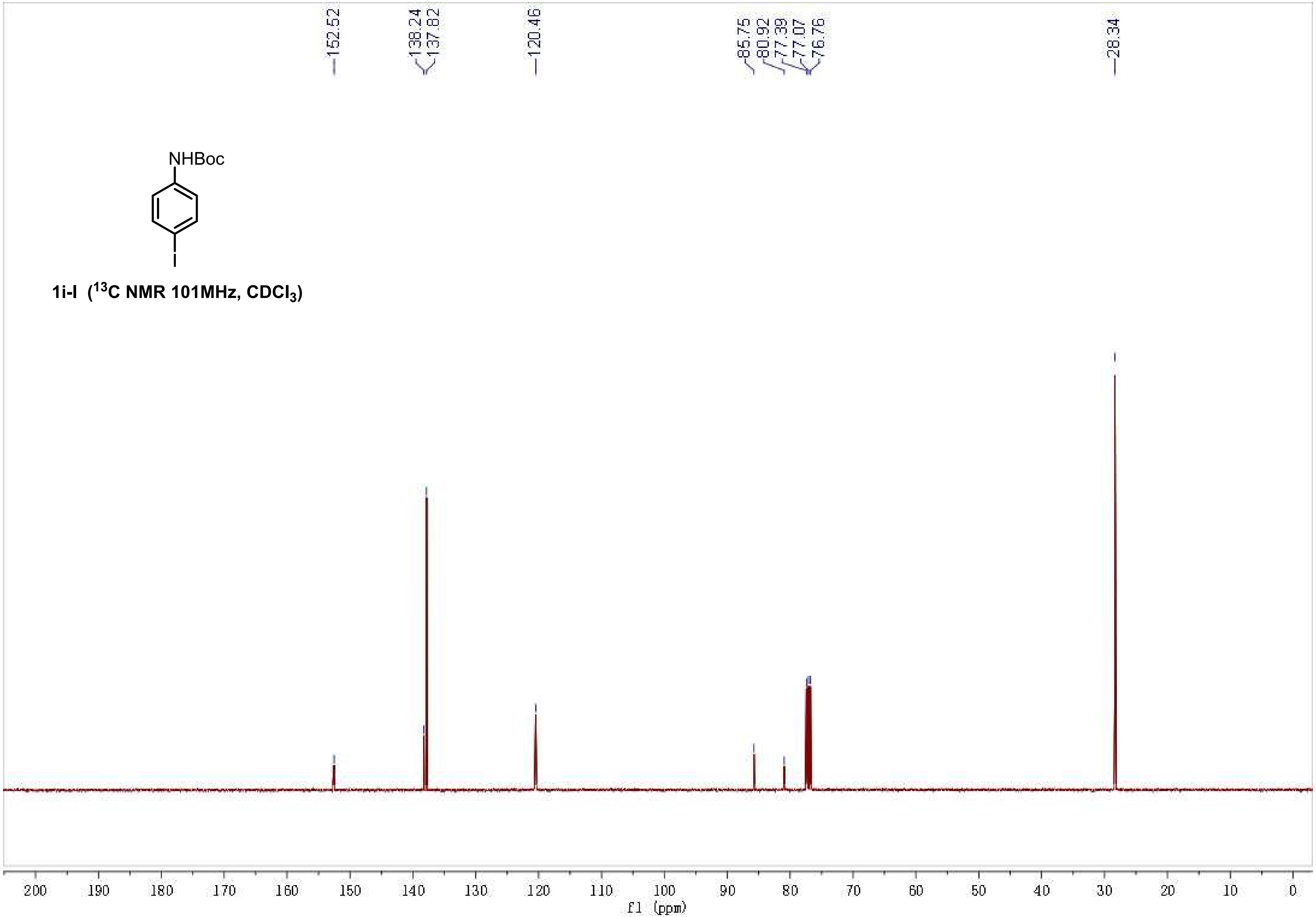

1i-I (^{13}C NMR 101MHz, $CDCl_3$)

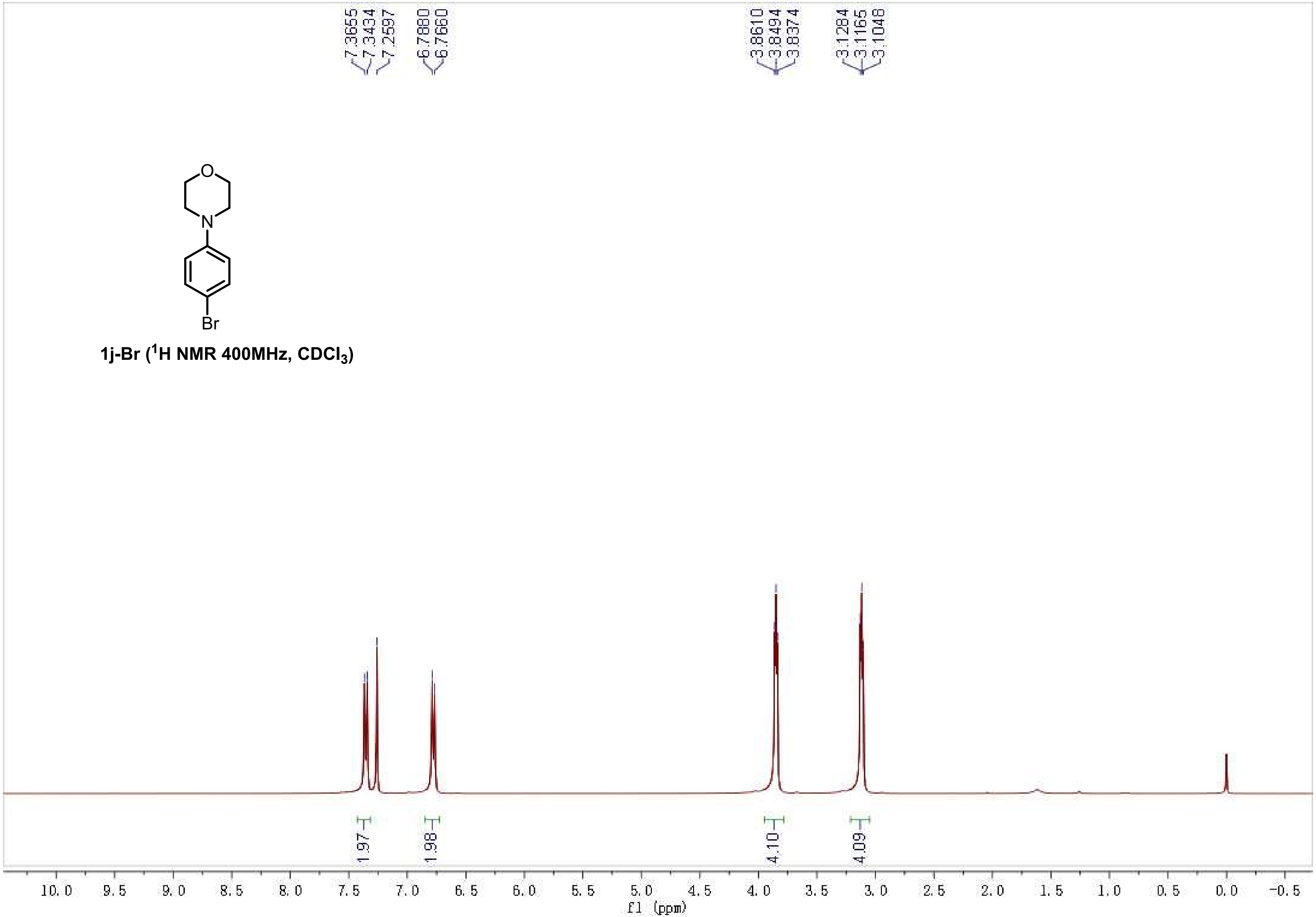

1j-Br (^{1}H NMR 400MHz, $CDCl_3$)

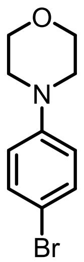

1j-Br (^{13}C NMR 101MHz, $CDCl_3$)

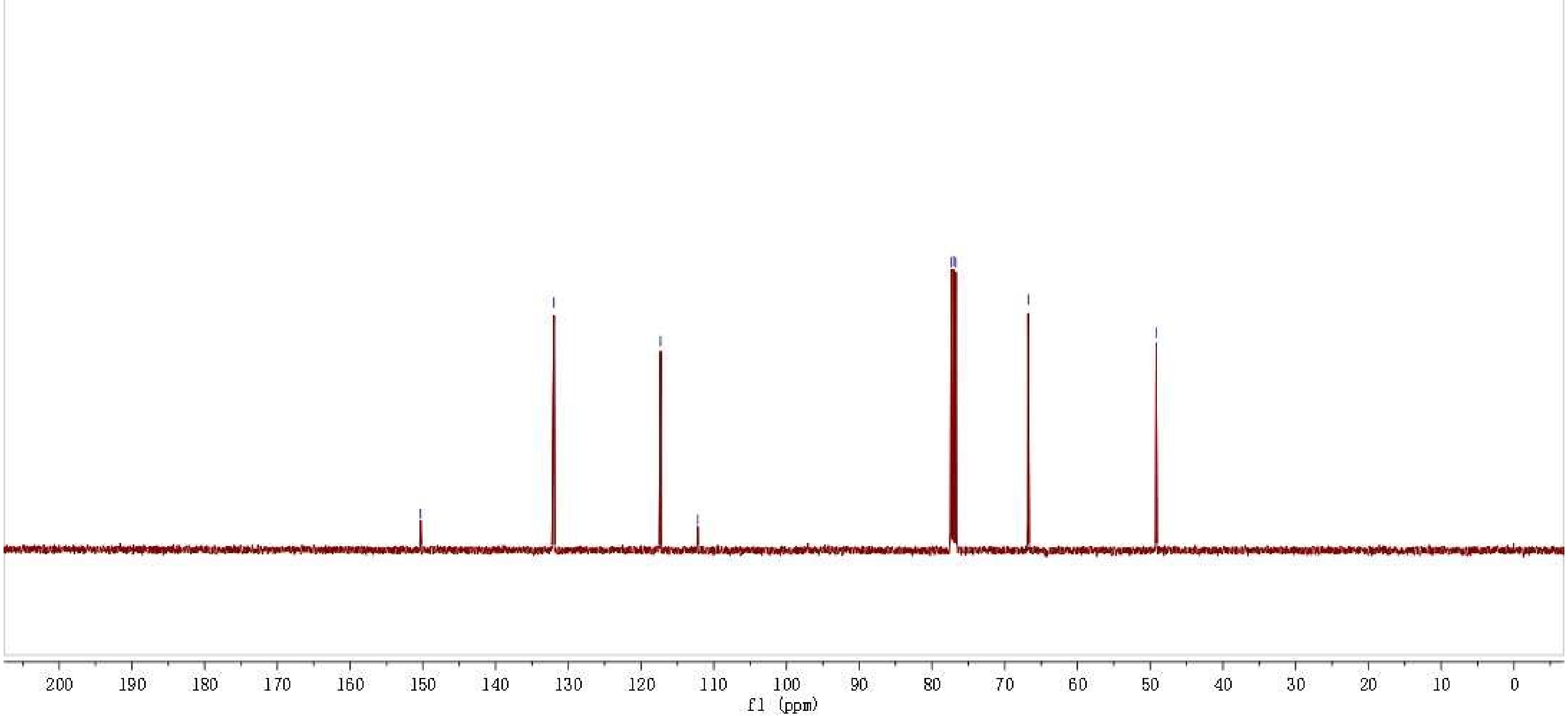

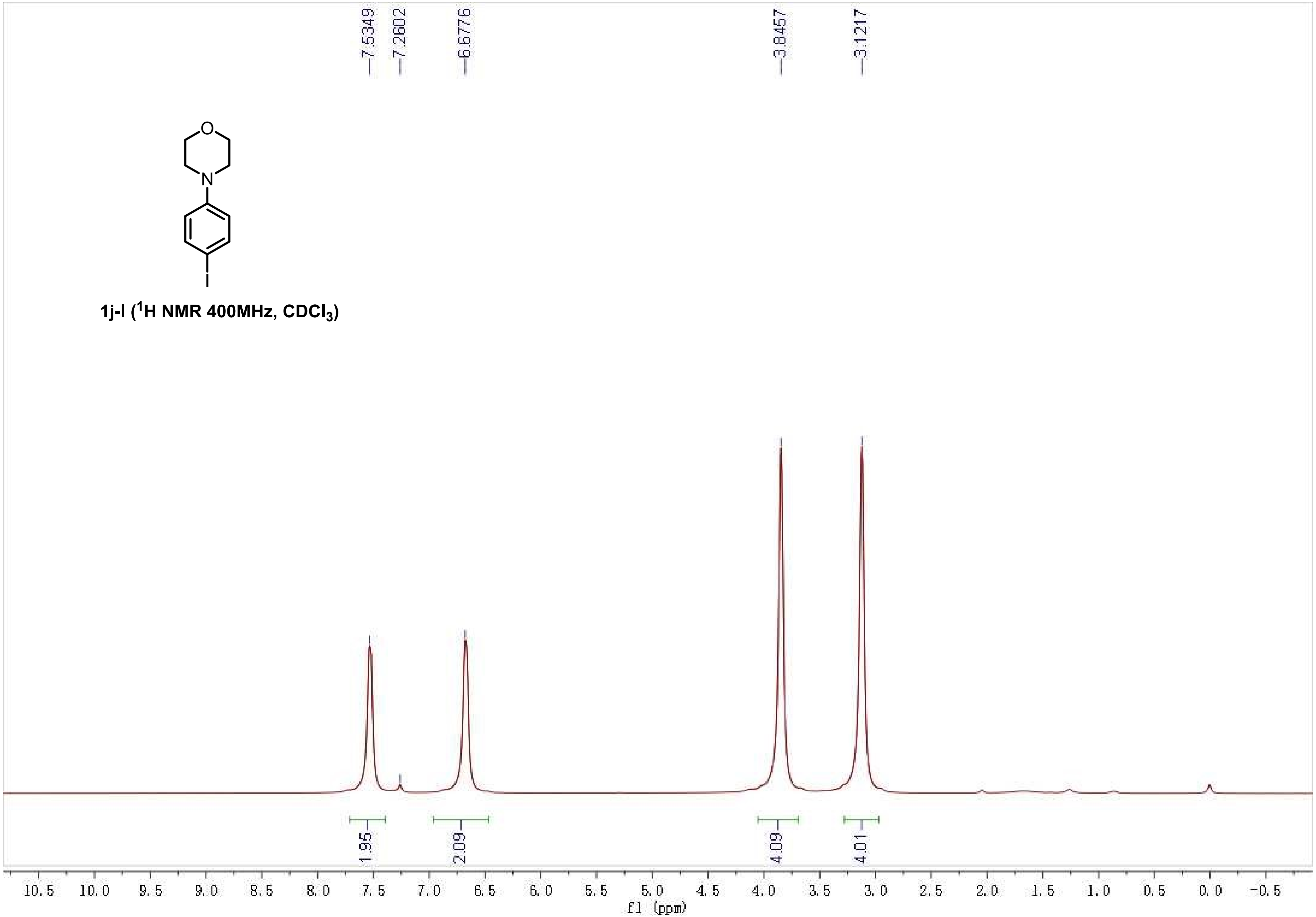

1j-I (^{1}H NMR 400MHz, $CDCl_3$)

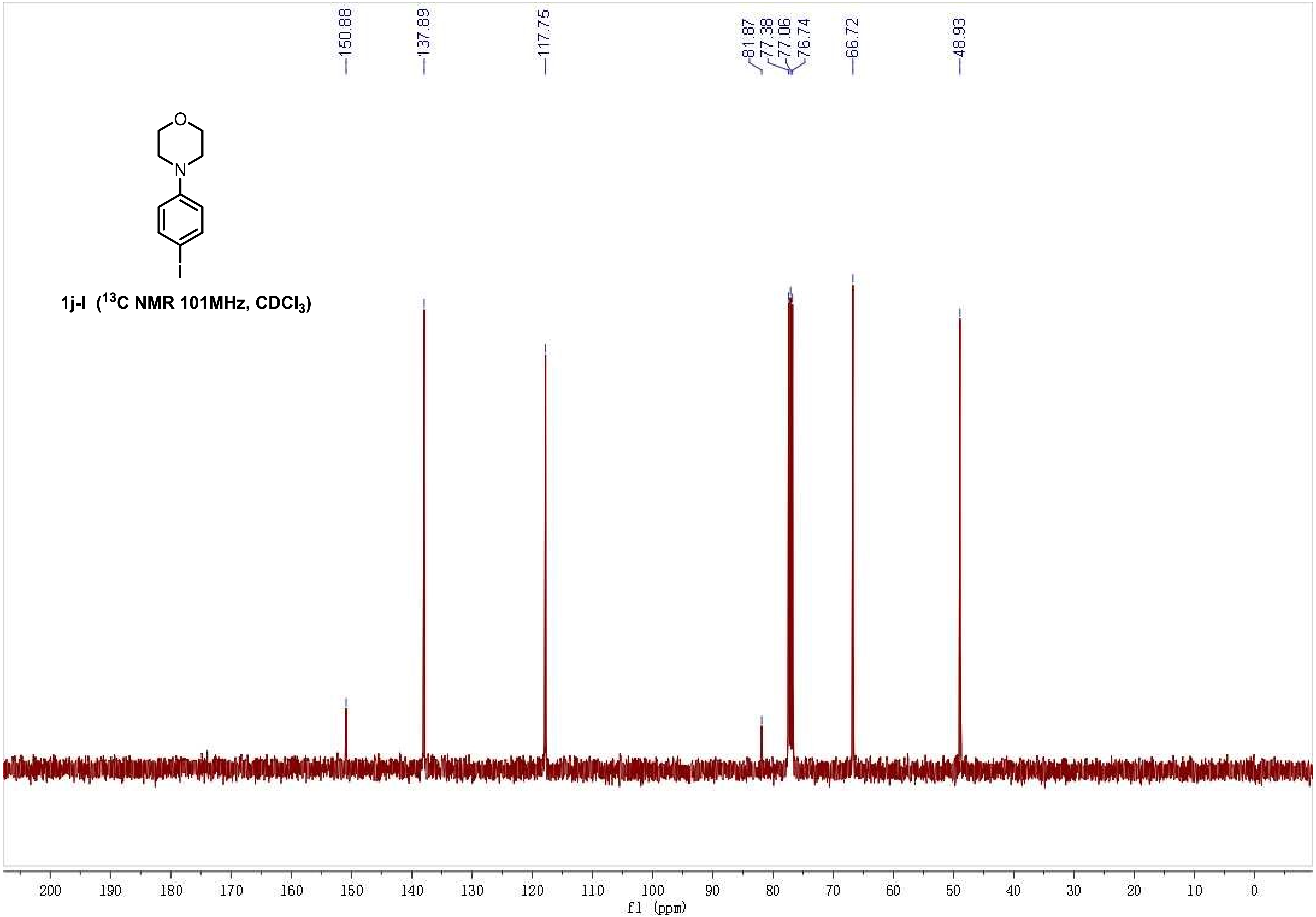

1j-I (^{13}C NMR 101MHz, $CDCl_3$)

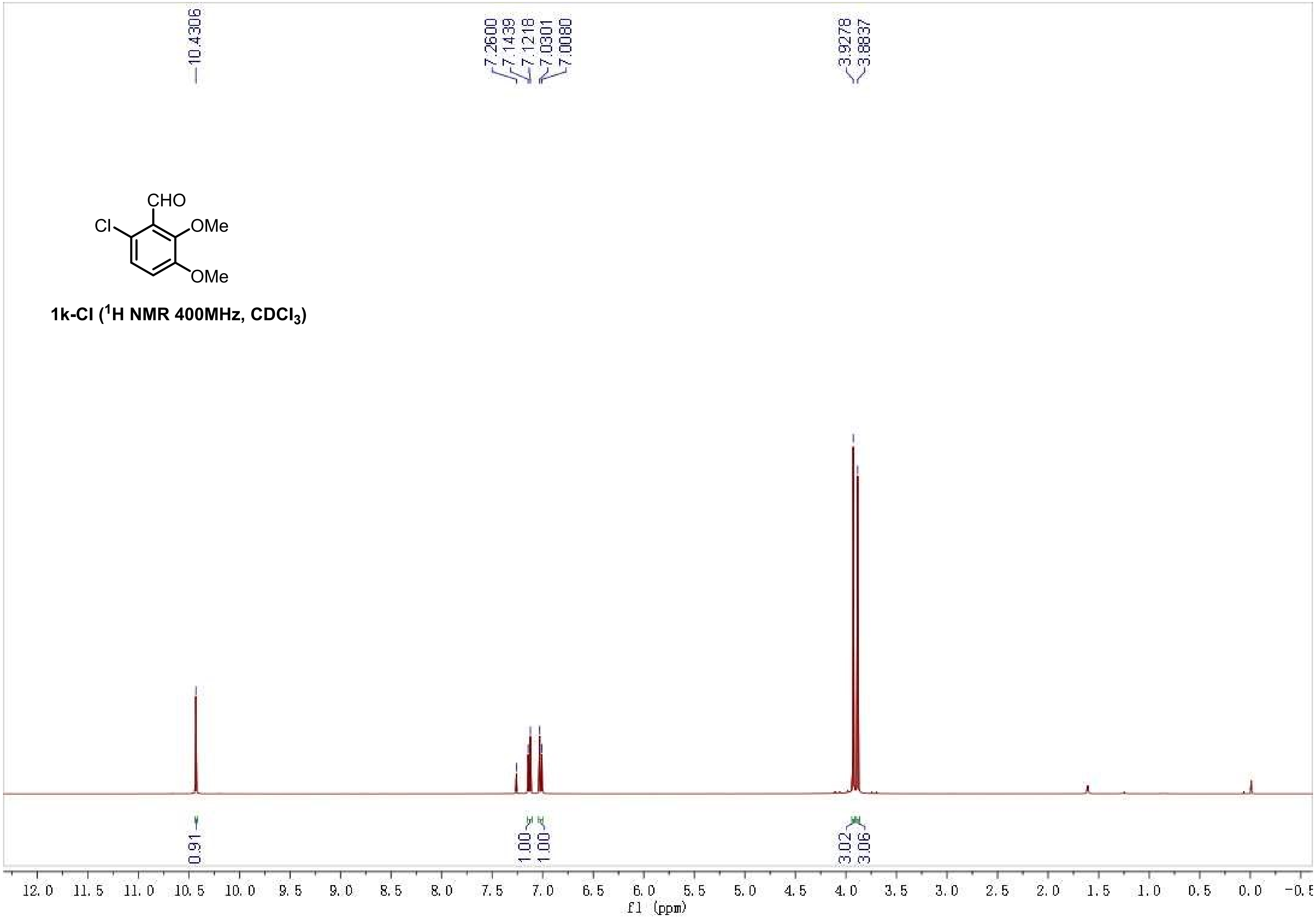

1k-Cl (^{1}H NMR 400MHz, $CDCl_3$)

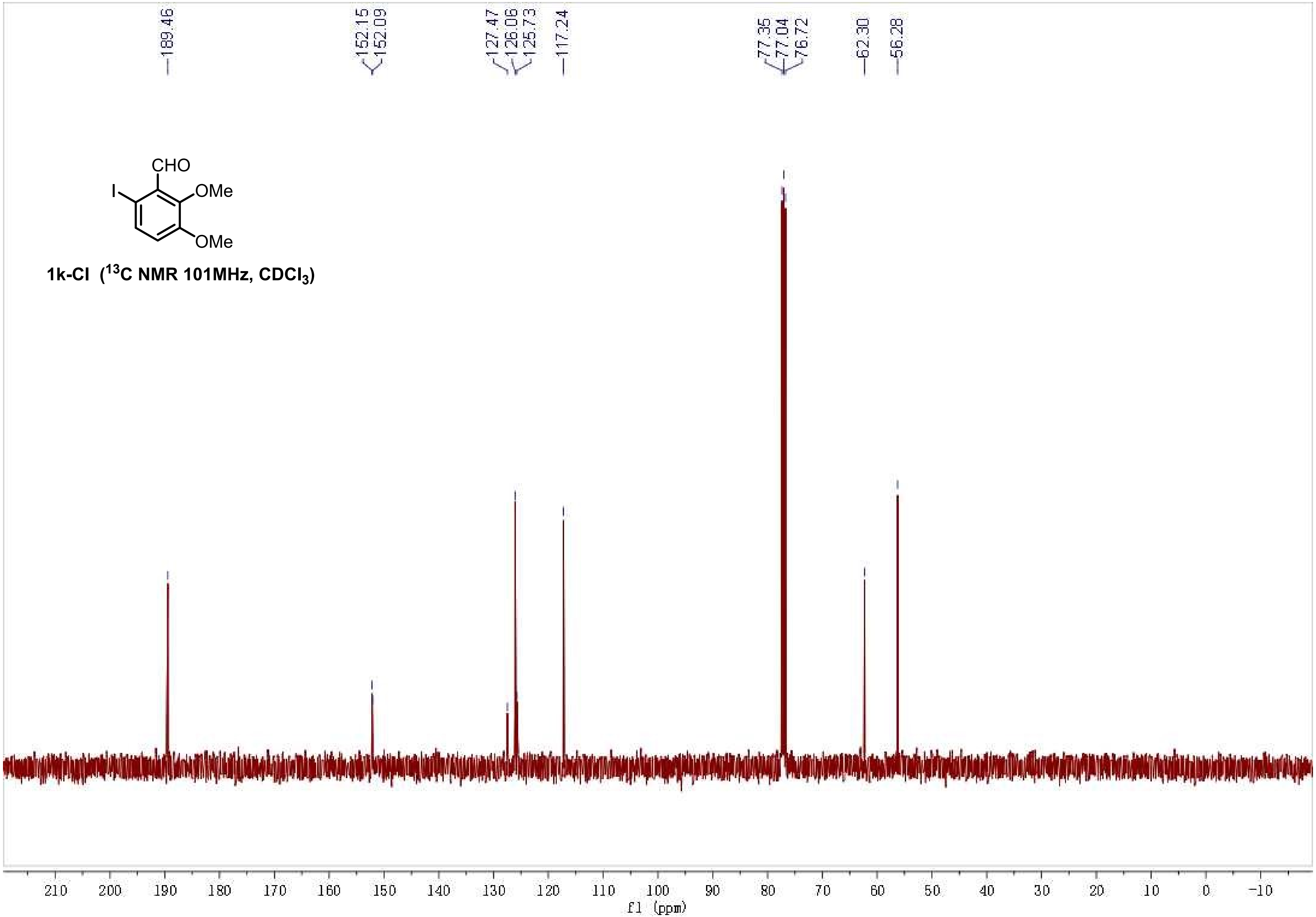

1k-Cl (${}^{13}C$ NMR 101MHz, $CDCl_3$)

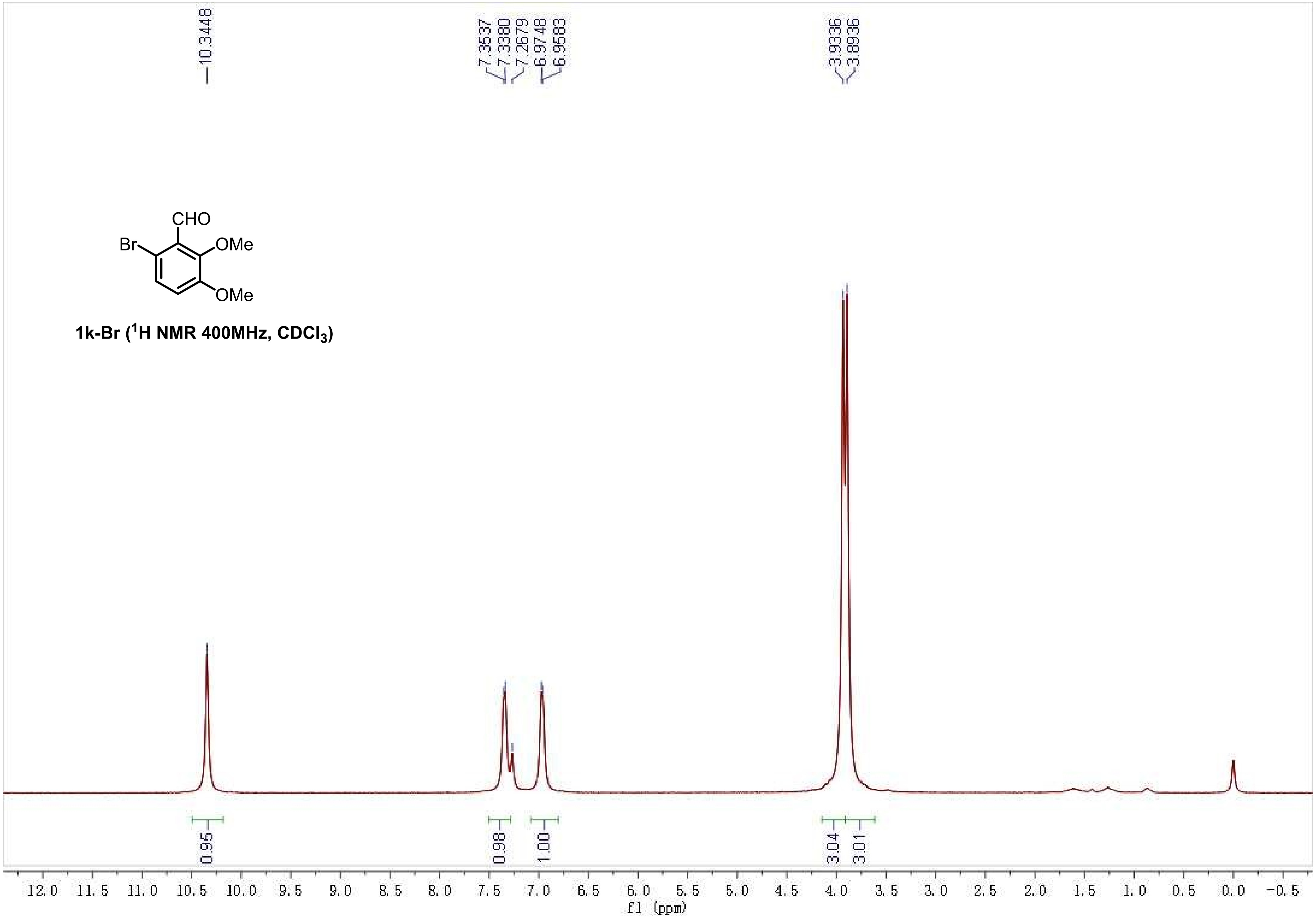

1k-Br (^{1}H NMR 400MHz, $CDCl_3$)

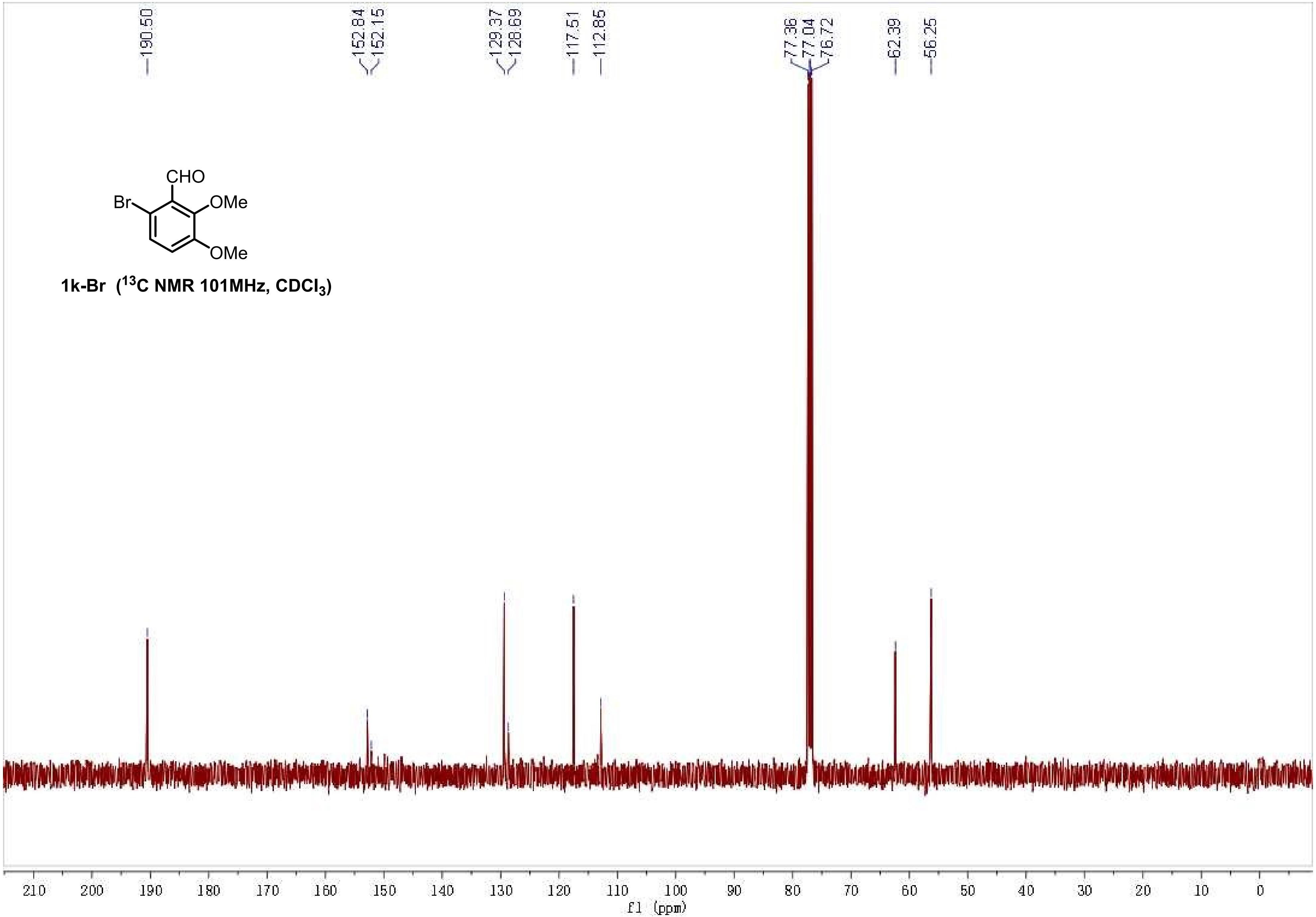

190.50
152.84
152.15
129.37
128.69
117.51
112.85
77.36
77.04
76.72
62.39
56.25
CHO
Br
OMe
OMe
1k-Br (13C NMR 101MHz, CDCl3)
210 200 190 180 170 160 150 140 130 120 110 100 90 80 70 60 50 40 30 20 10 0
f1 (ppm)

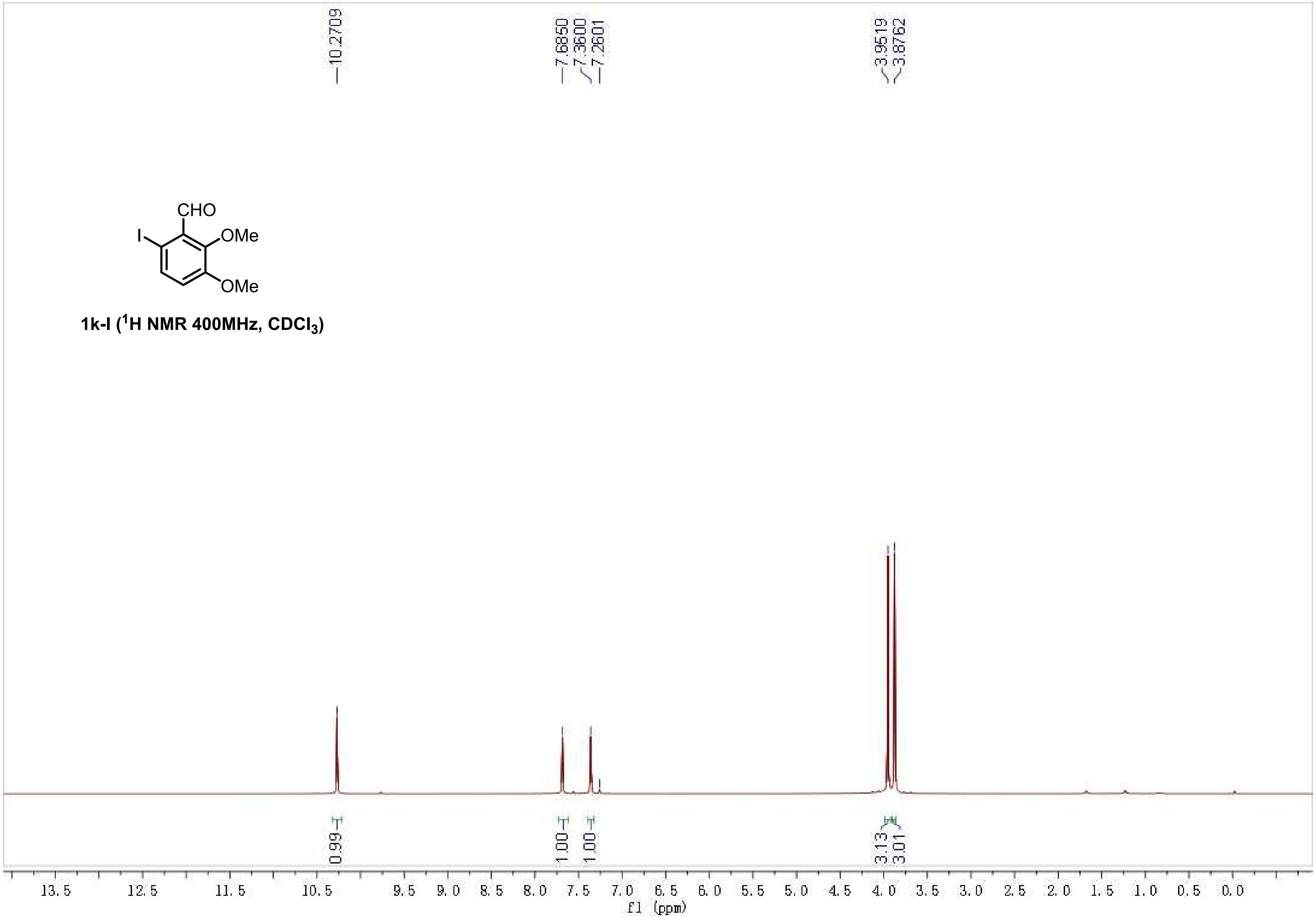

1k-I (^{1}H NMR 400MHz, $CDCl_3$)

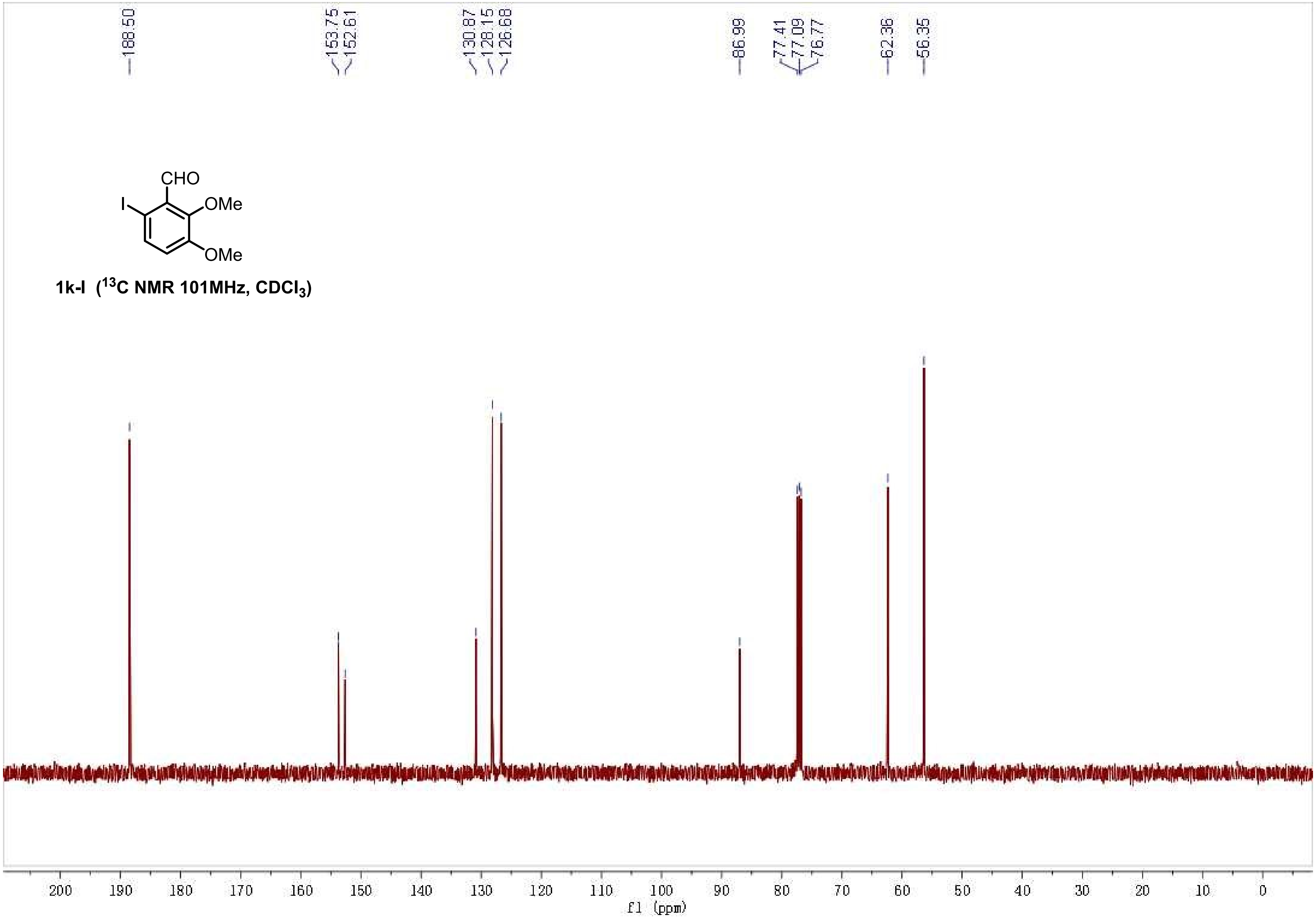

1k-I (**^{13}C NMR 101MHz, $CDCl_3$**)

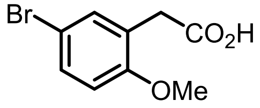

1l-Br (^{1}H NMR 400MHz, $CDCl_3$)

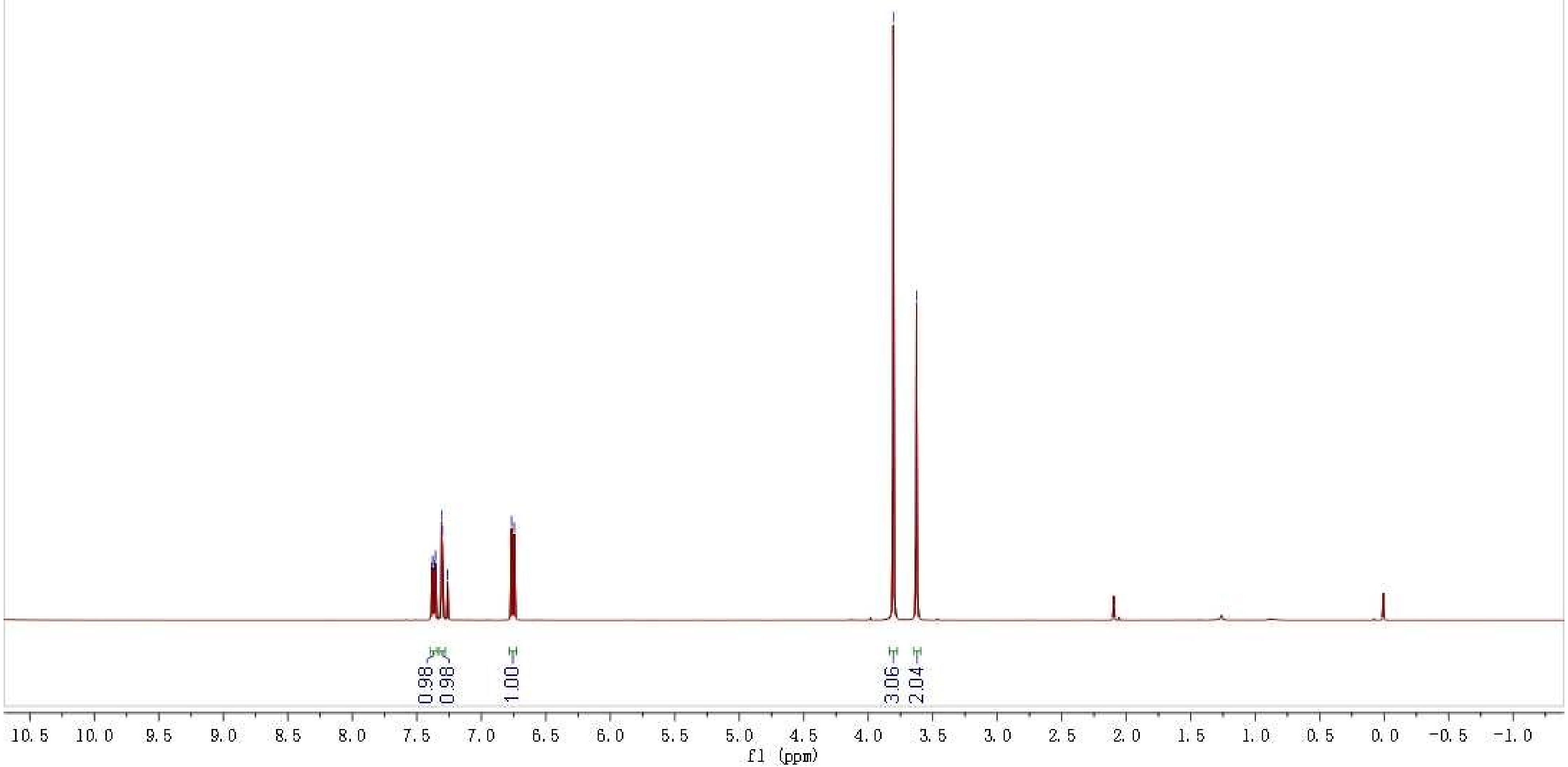

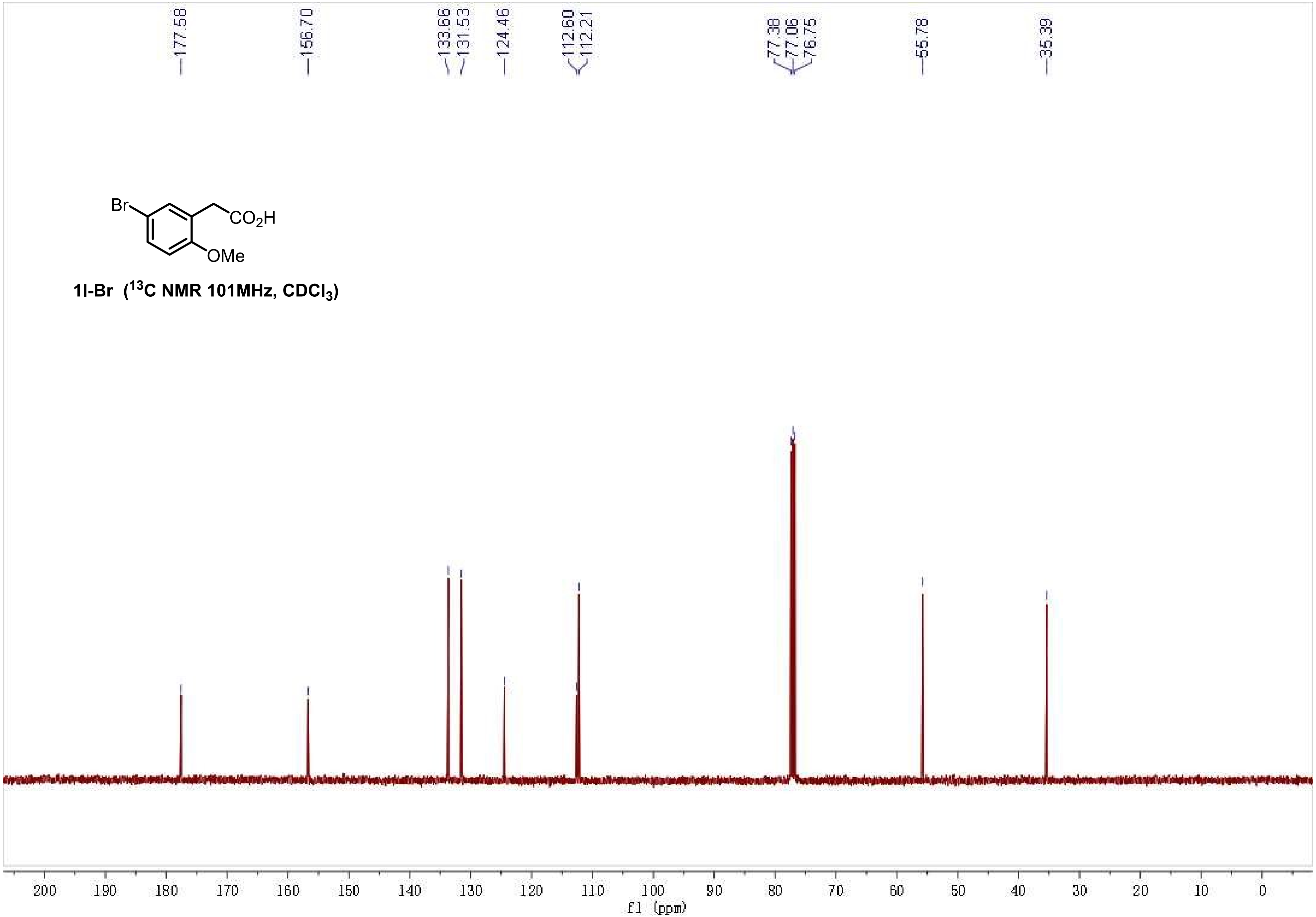

1l-Br (^{13}C NMR 101MHz, $CDCl_3$)

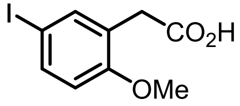

1l-I (^{1}H NMR 400MHz, $CDCl_3$)

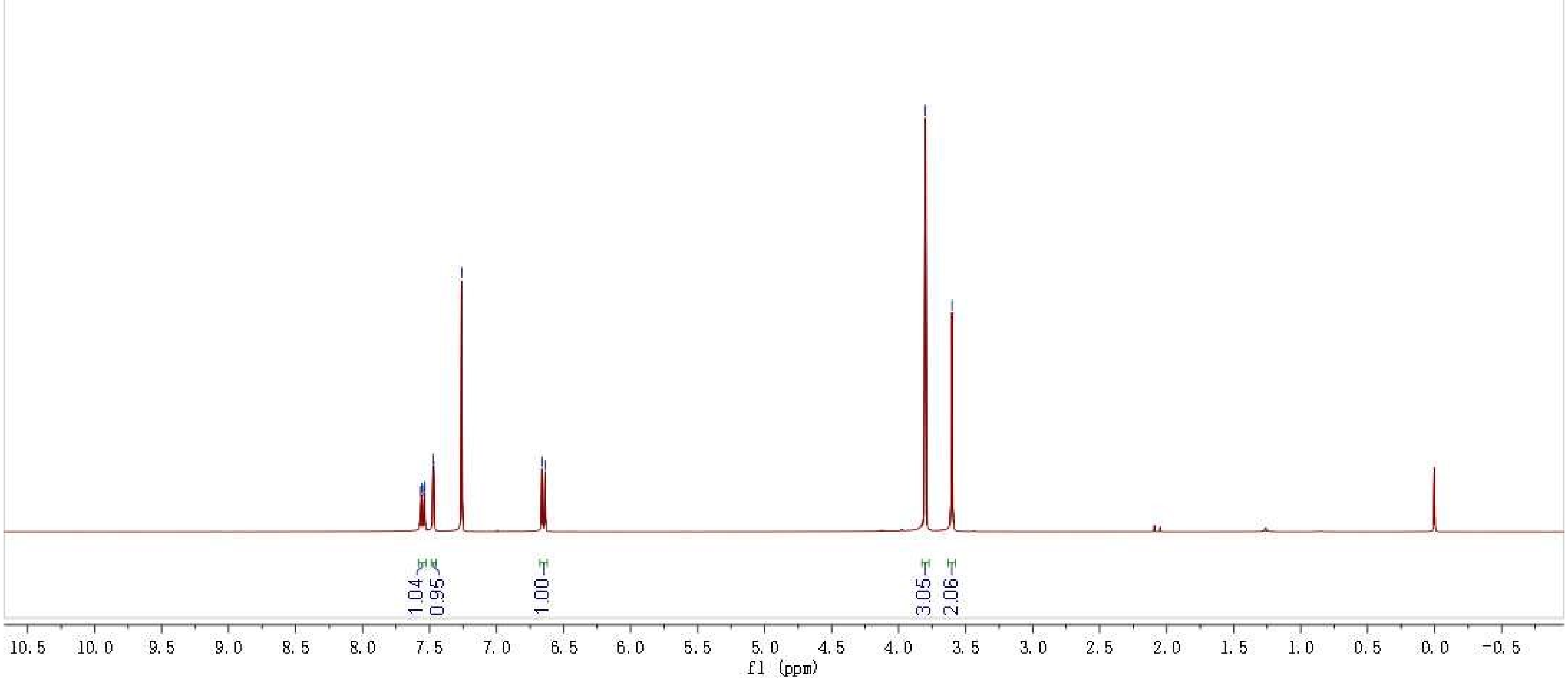

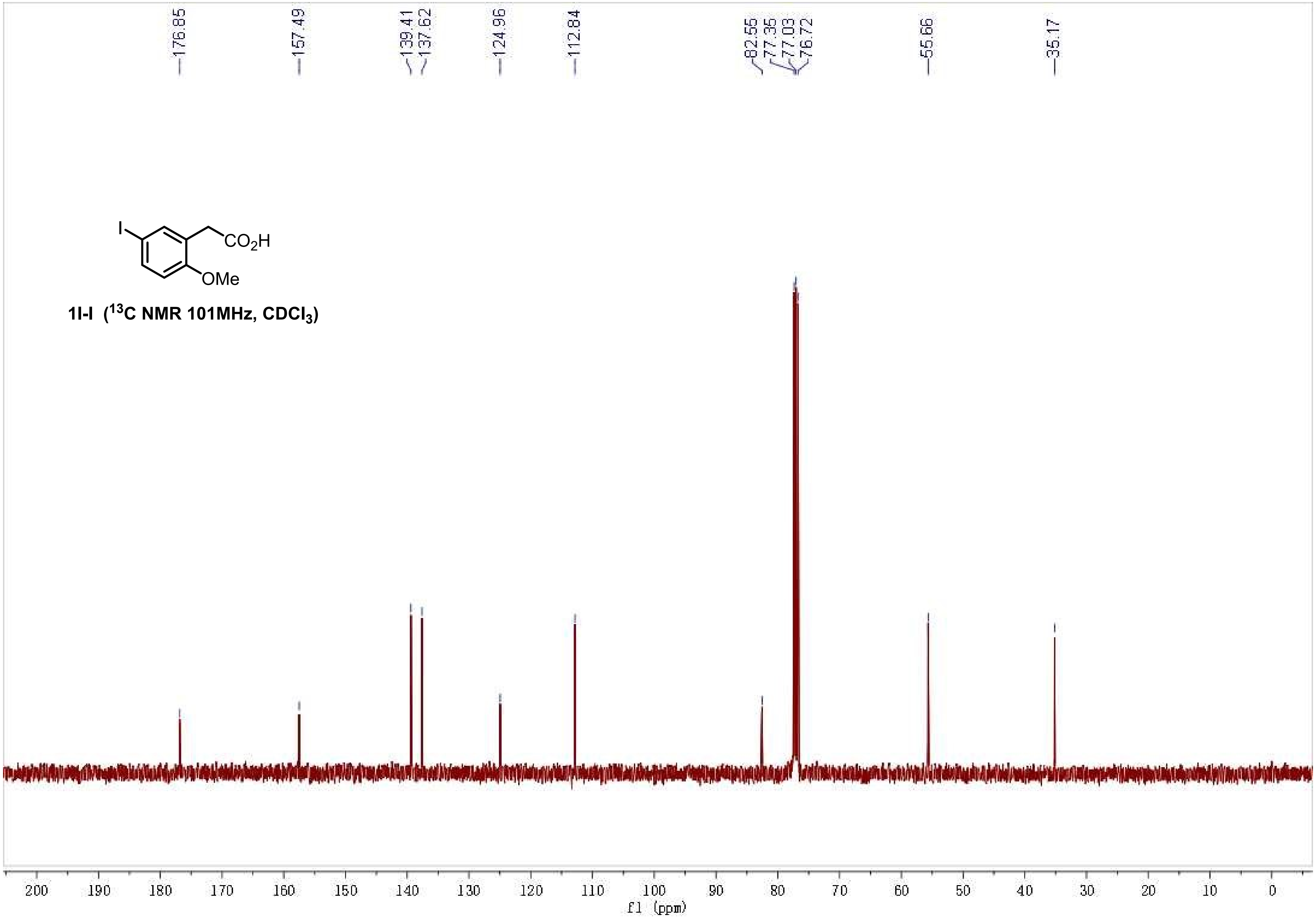

1l-I (^{13}C NMR 101MHz, $CDCl_3$)

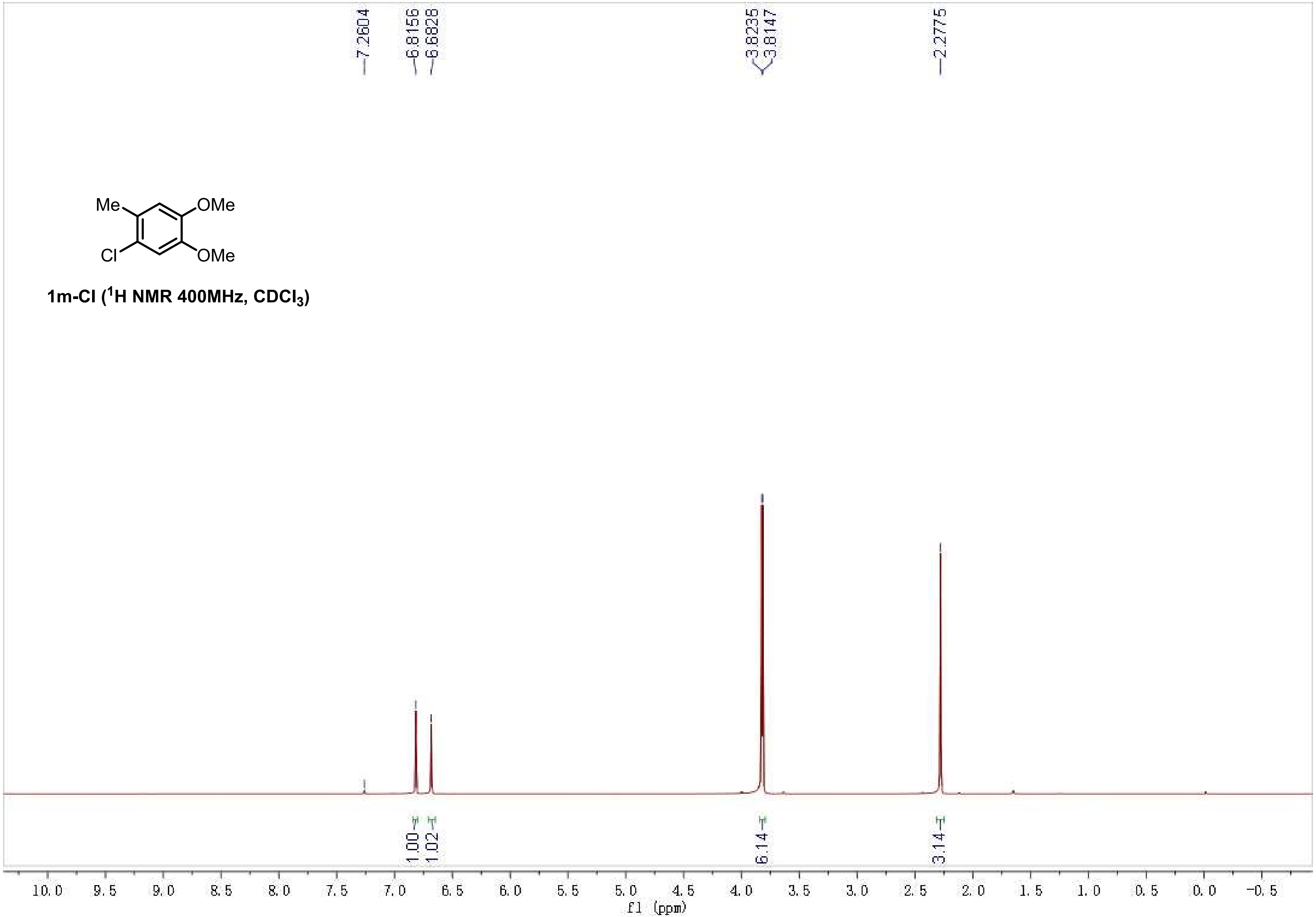
7.2604
6.8156
6.6828
3.8235
3.8147
2.2775
Me
OMe
Cl
OMe
1m-Cl (1H NMR 400MHz, CDCl3)
1.00
1.02
6.14
3.14
10.0 9.5 9.0 8.5 8.0 7.5 7.0 6.5 6.0 5.5 5.0 4.5 4.0 3.5 3.0 2.5 2.0 1.5 1.0 0.5 0.0 -0.5
f1 (ppm)

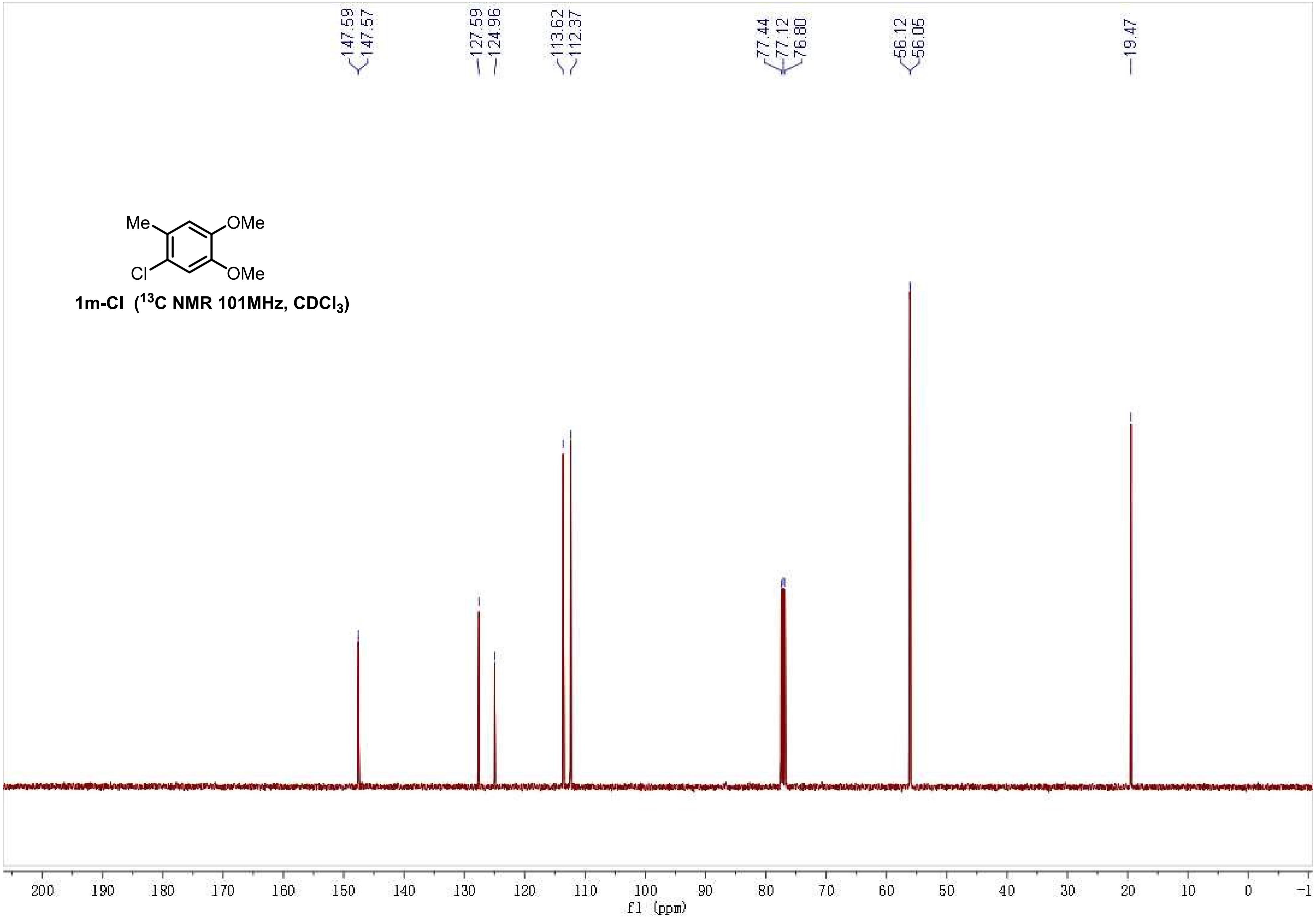
147.59
147.57
127.59
124.96
113.62
112.37
77.44
77.12
76.80
56.12
56.05
19.47
Me
OMe
Cl
OMe
1m-Cl (^{13}C NMR 101MHz, $CDCl_3$)
200 190 180 170 160 150 140 130 120 110 100 90 80 70 60 50 40 30 20 10 0 -1
f1 (ppm)

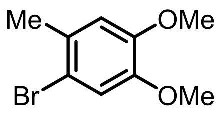

1m-Br (^{1}H NMR 400MHz, $CDCl_3$)

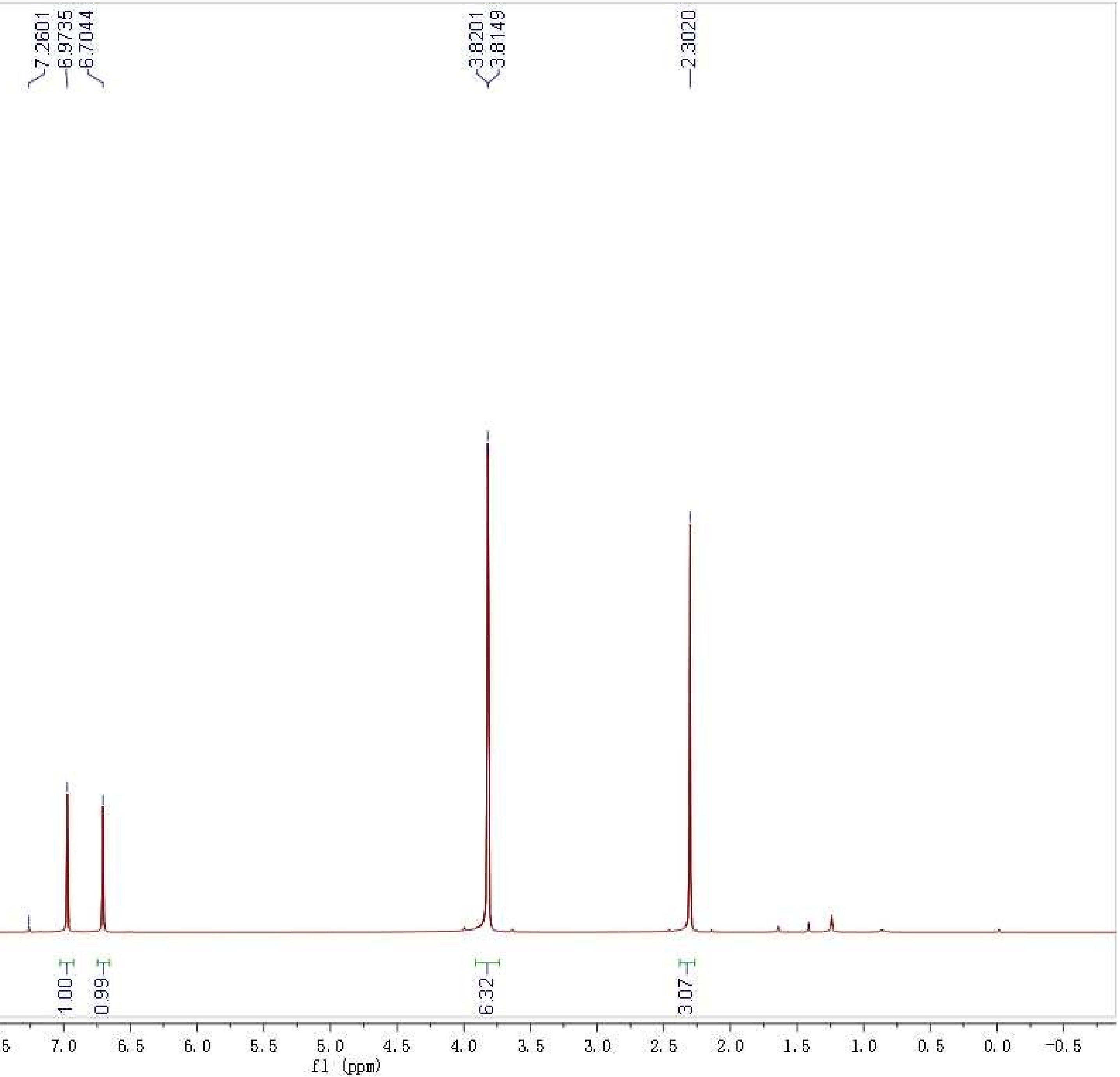

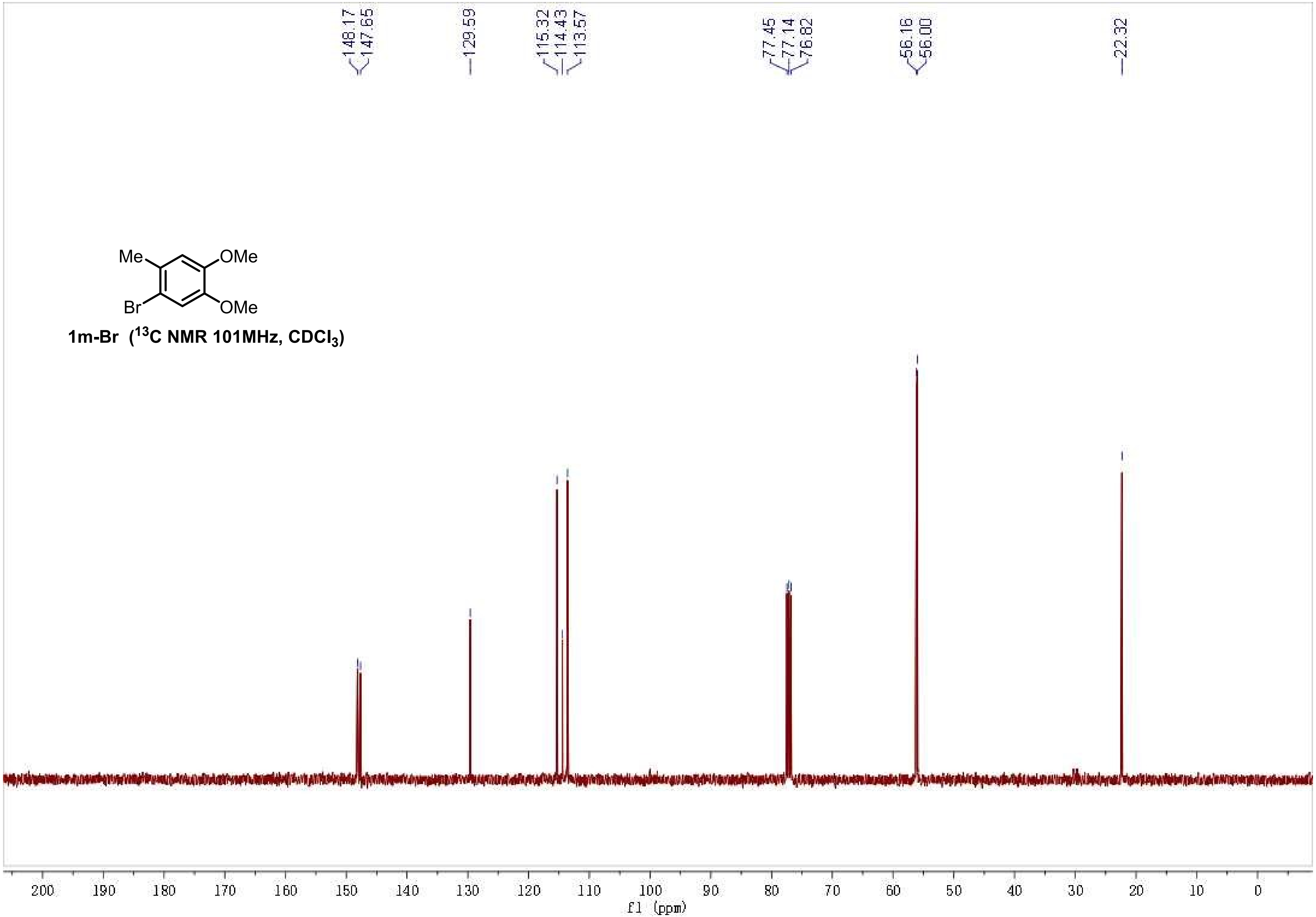
148.17
147.65
129.59
115.32
114.43
113.57
77.45
77.14
76.82
56.16
56.00
22.32
Me
OMe
Br
OMe
1m-Br (13C NMR 101MHz, CDCl3)
200 190 180 170 160 150 140 130 120 110 100 90 80 70 60 50 40 30 20 10 0
f1 (ppm)

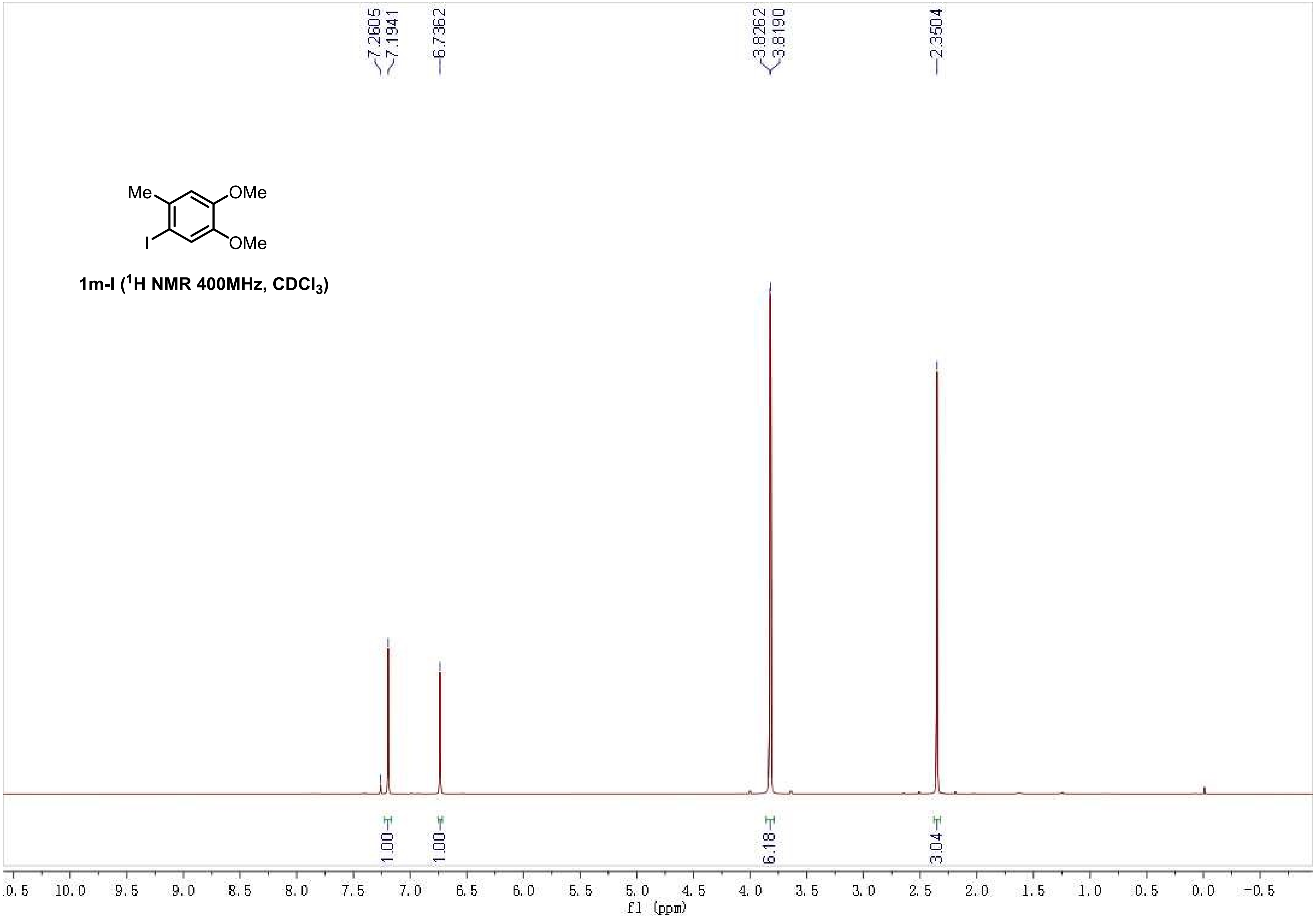
7.2605
7.1941
6.7362
3.8262
3.8190
2.3504
Me
OMe
I
OMe
1m-I (1H NMR 400MHz, CDCl3)
1.00
1.00
6.18
3.04
10.5
10.0
9.5
9.0
8.5
8.0
7.5
7.0
6.5
6.0
5.5
5.0
4.5
4.0
3.5
3.0
2.5
2.0
1.5
1.0
0.5
0.0
-0.5
f1 (ppm)

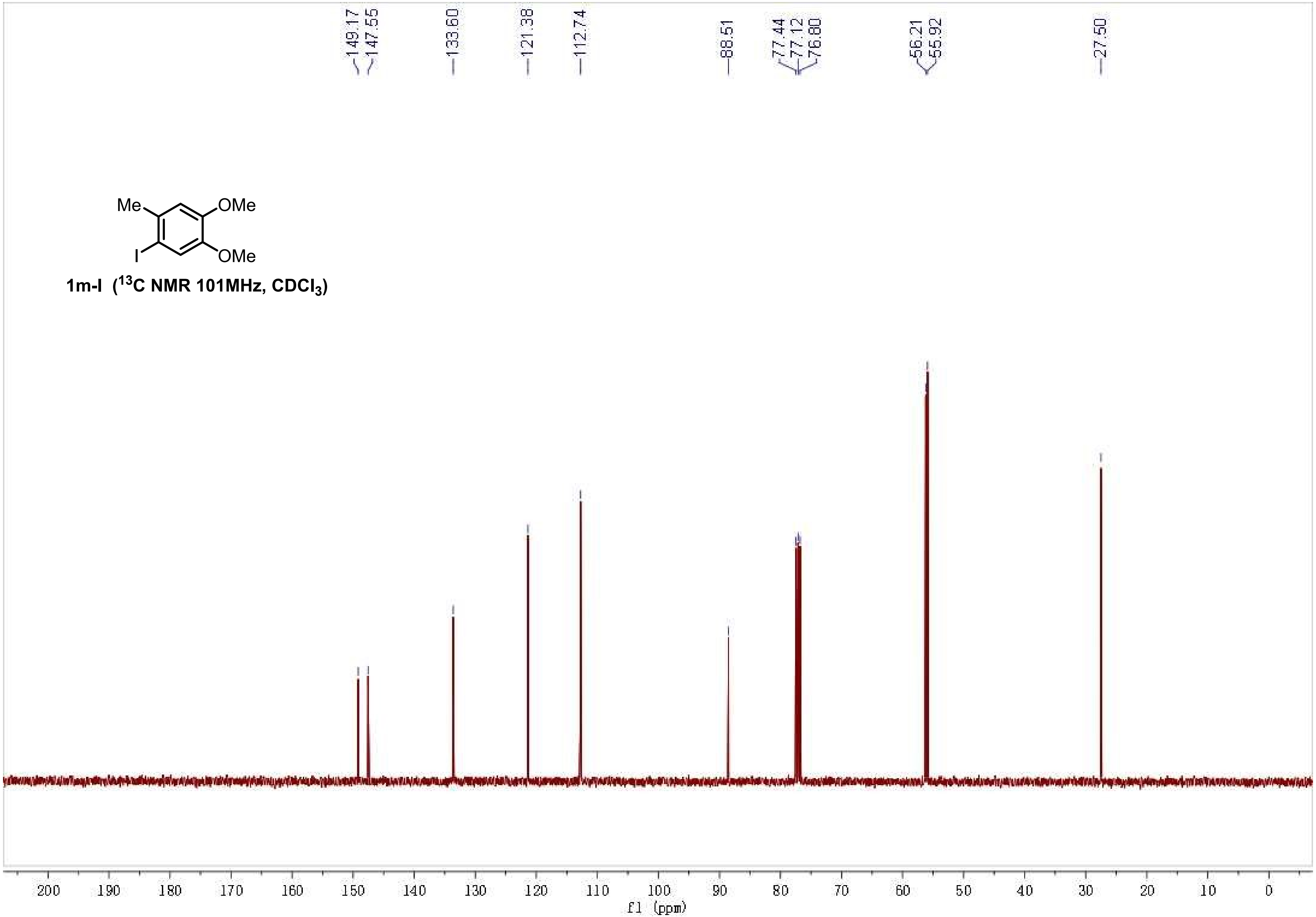

1m-I (${}^{13}C$ NMR 101MHz, $CDCl_3$)

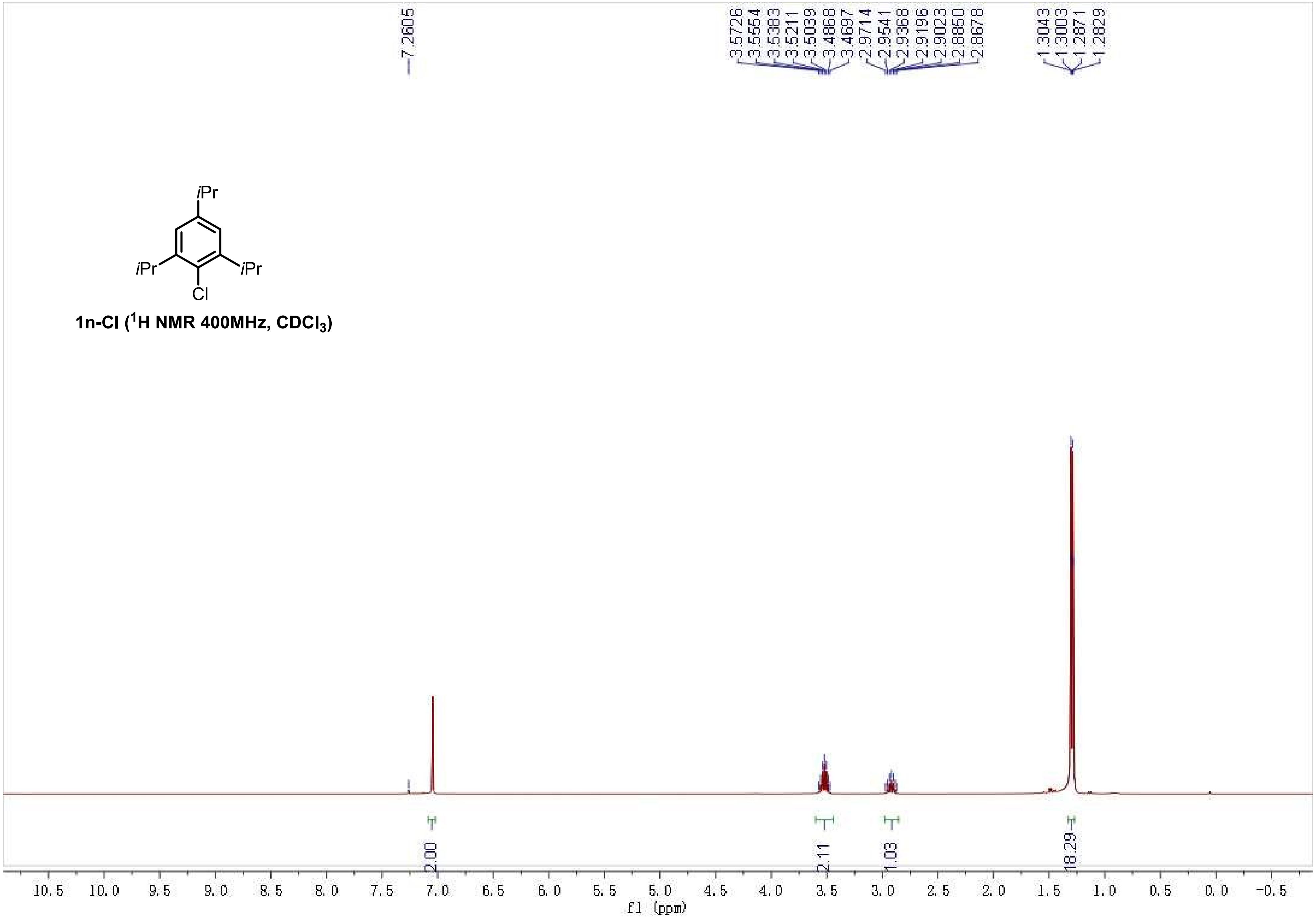

1n-Cl (^{1}H NMR 400MHz, $CDCl_3$)

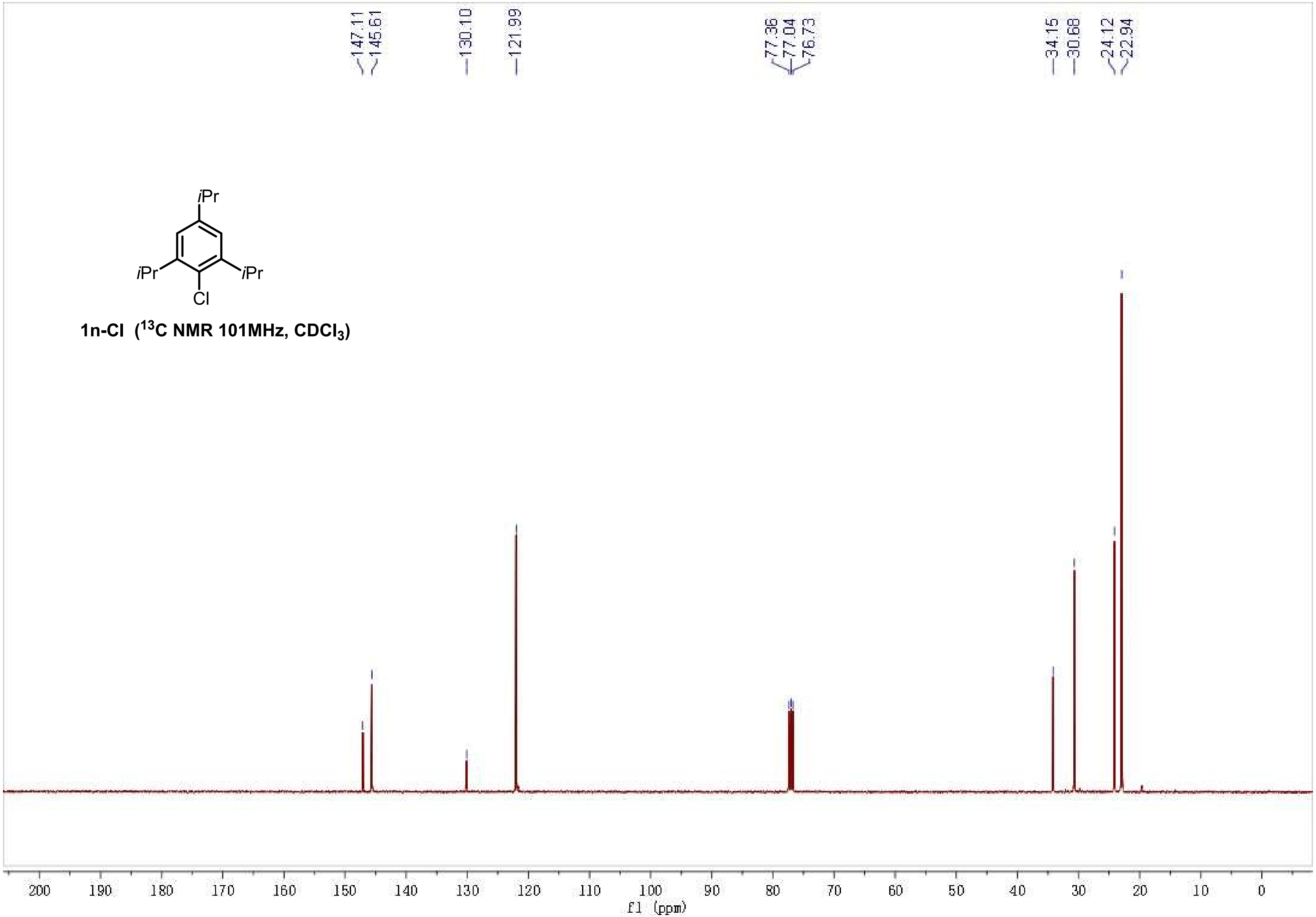

1n-Cl (^{13}C NMR 101MHz, $CDCl_3$)

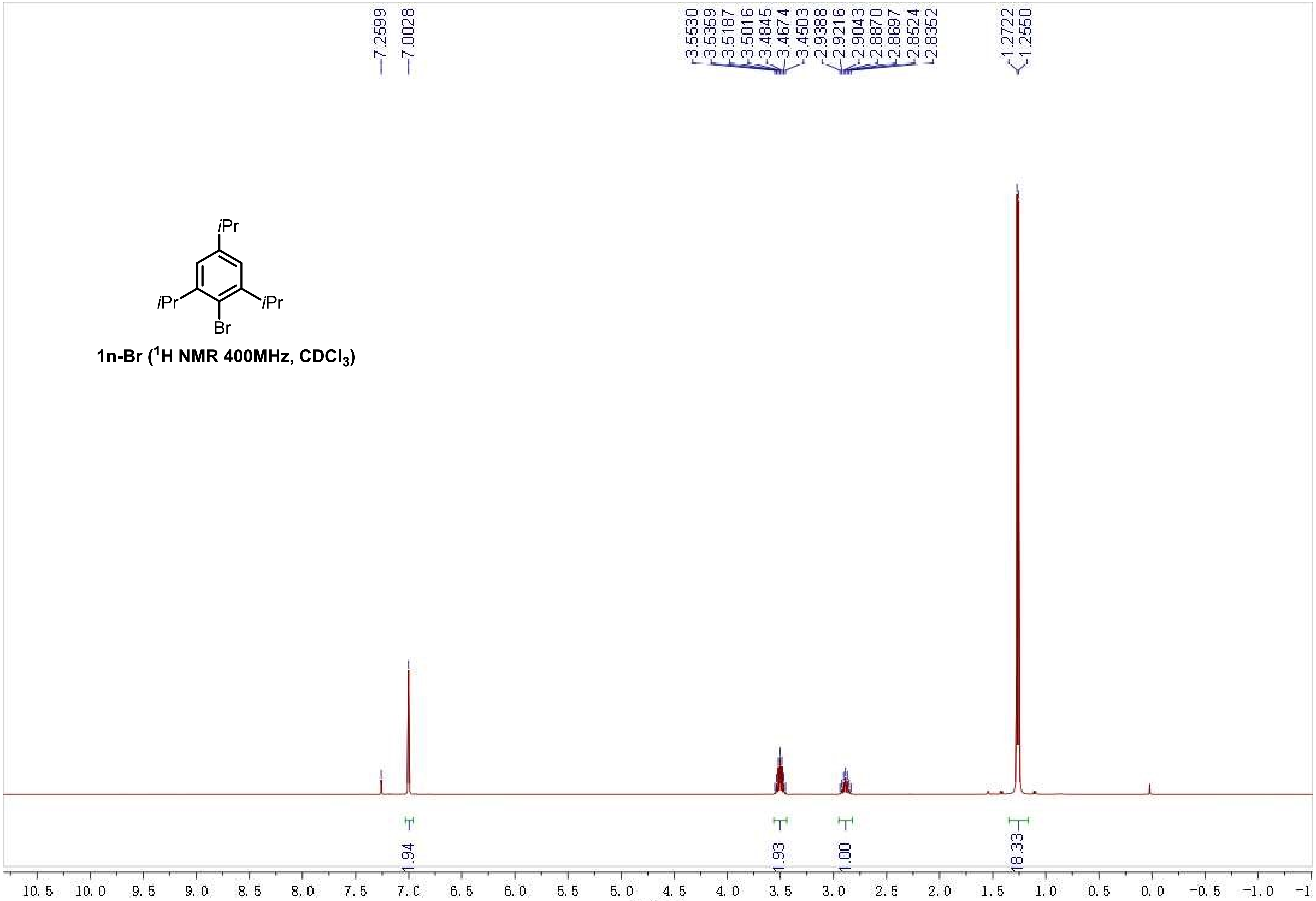

1n-Br (^{1}H NMR 400MHz, $CDCl_3$)

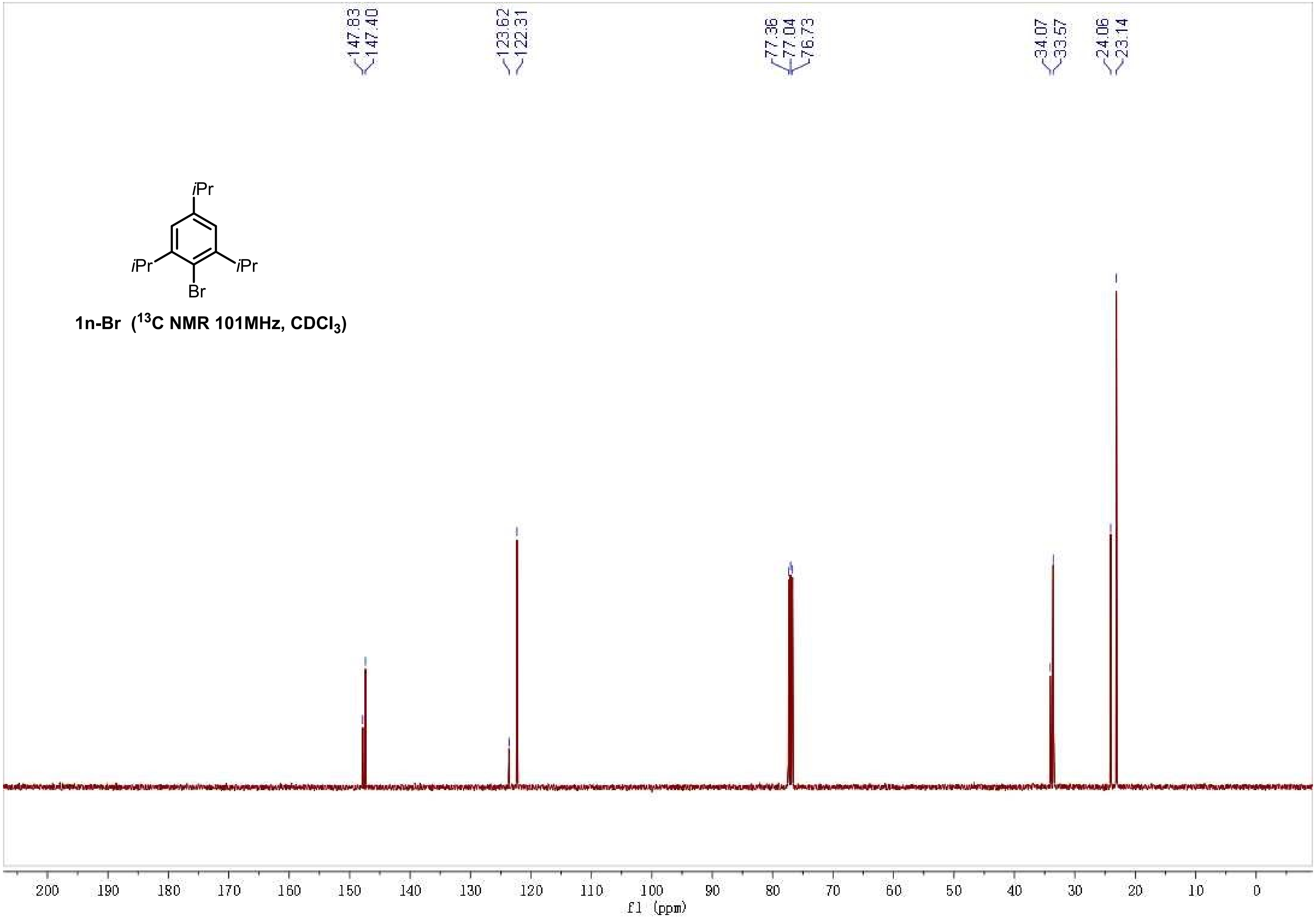

1n-Br (^{13}**C NMR 101MHz, CDCl$_3$)**

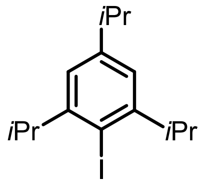

1n-I (^{1}H NMR 400MHz, $CDCl_3$)

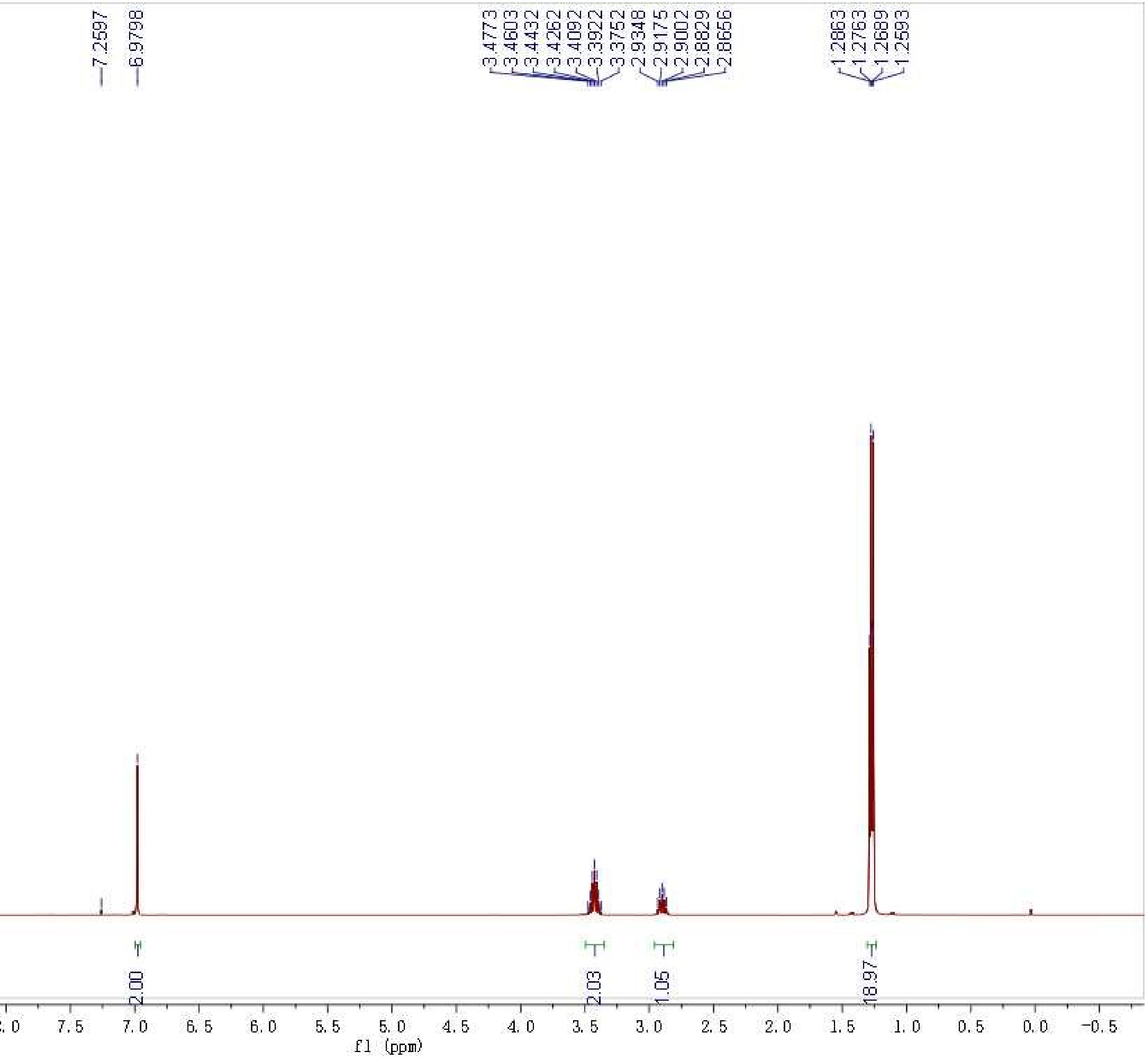

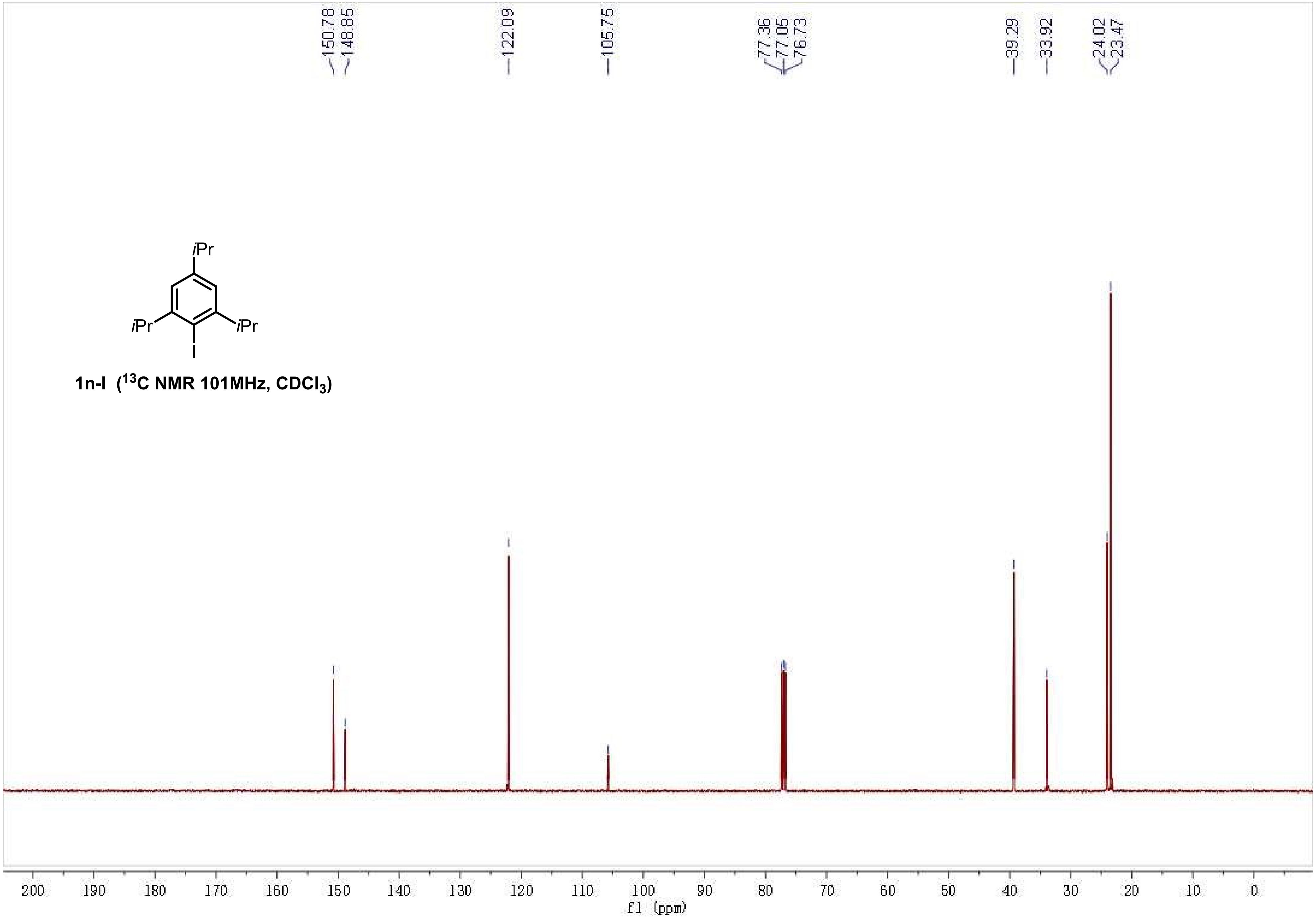

1n-I (**^{13}C NMR 101MHz, CDCl$_3$**)

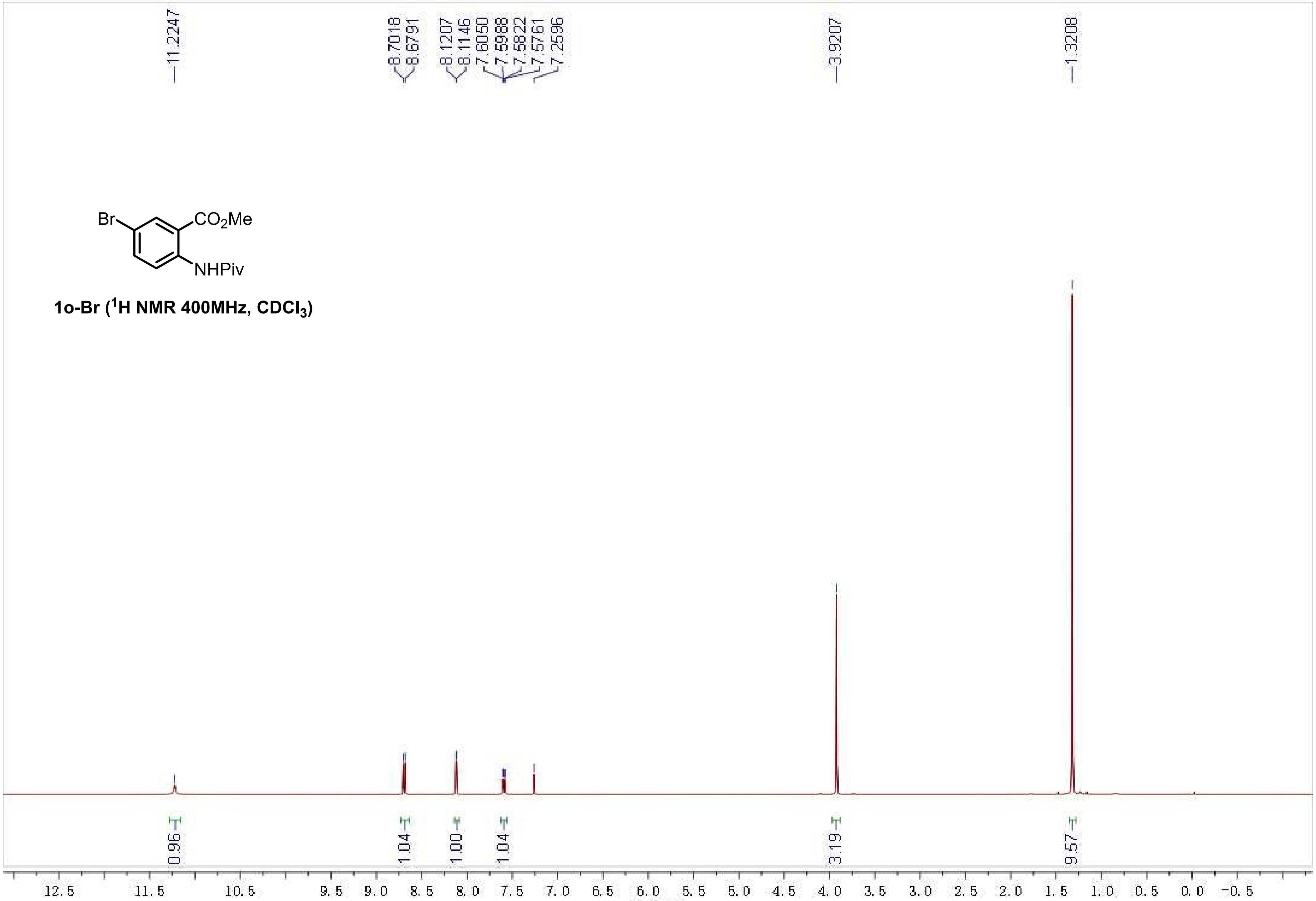

1o-Br (^{1}H NMR 400MHz, $CDCl_3$)

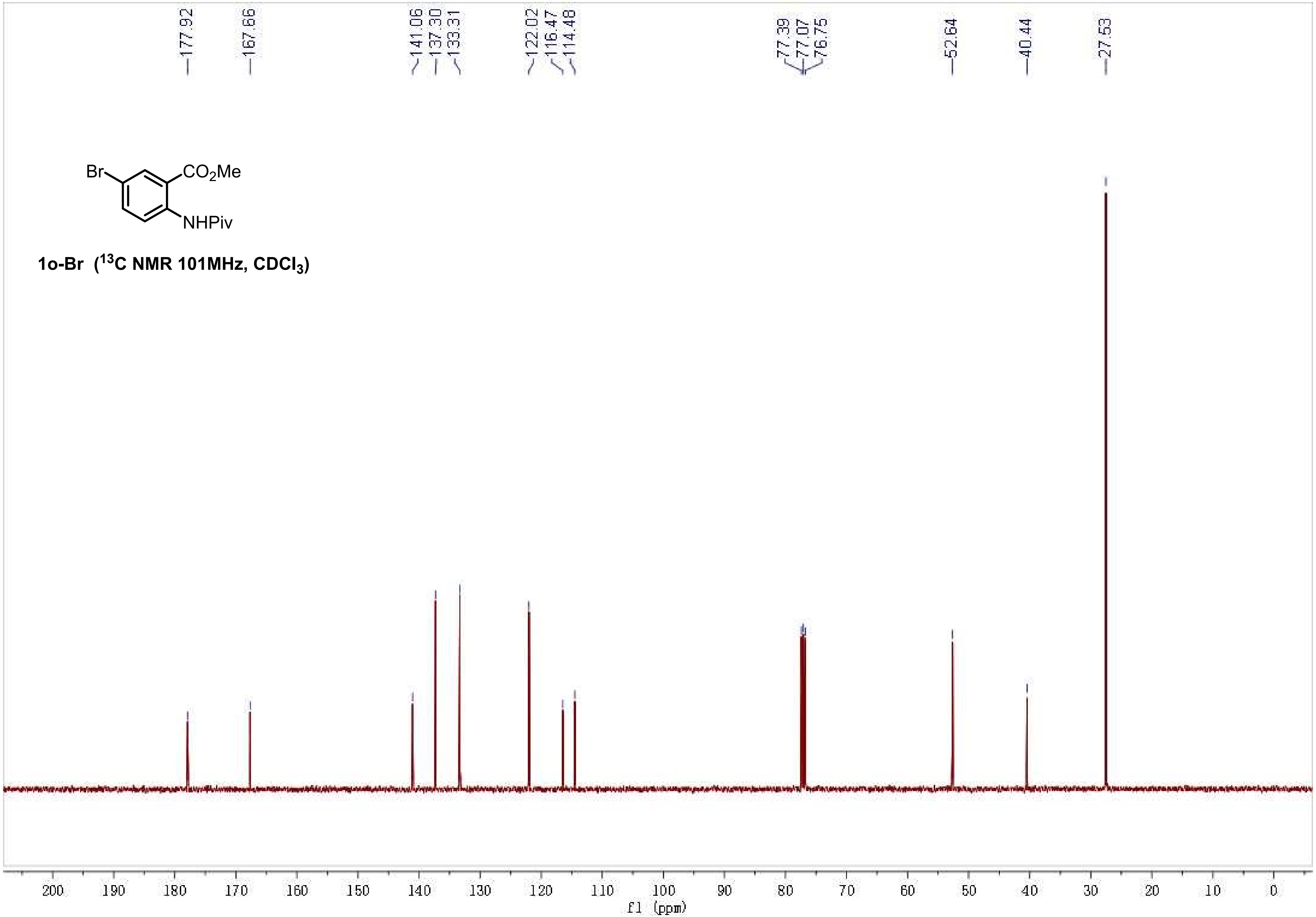

1o-Br (^{13}C NMR 101MHz, $CDCl_3$)

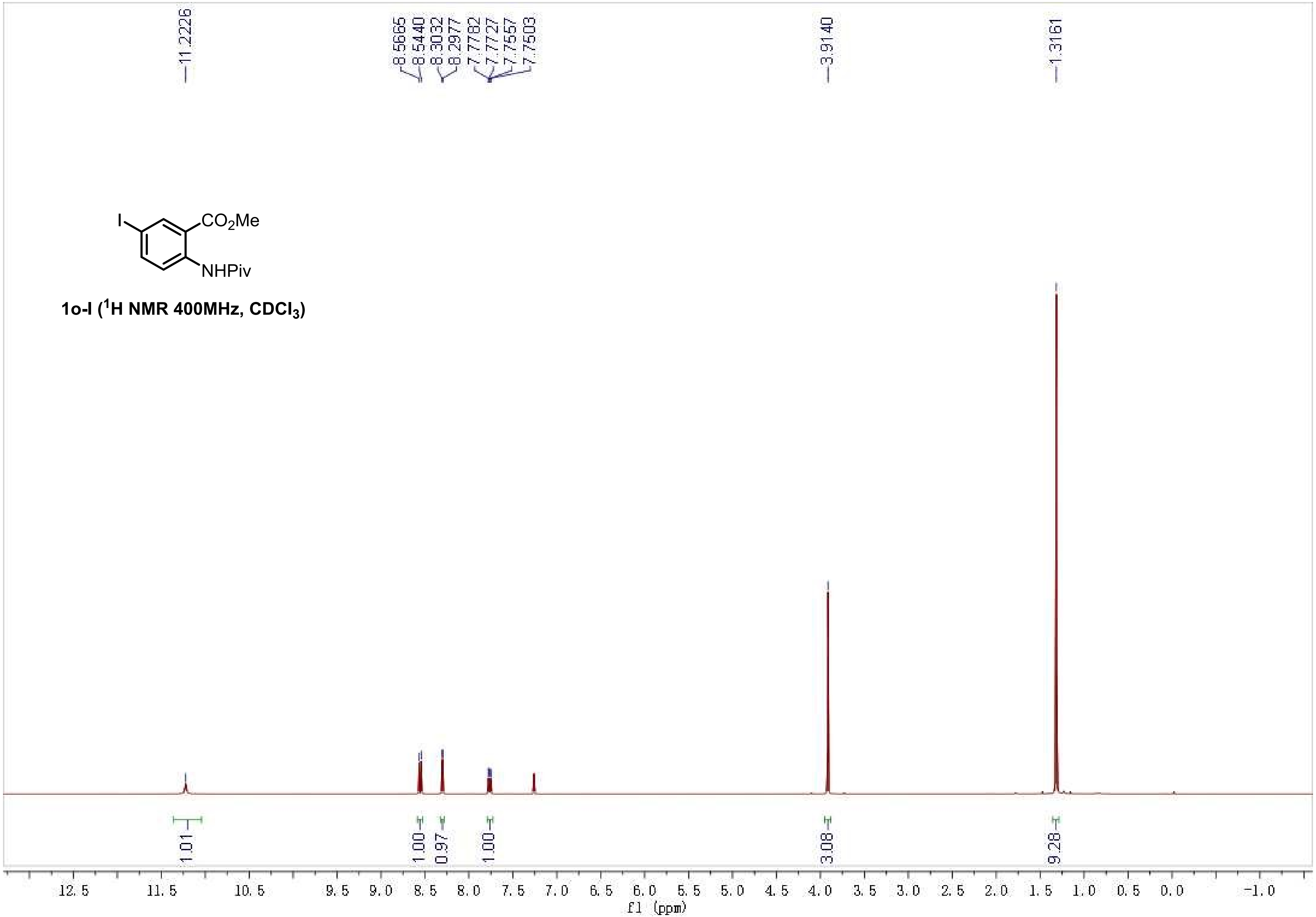

1o-I (^{1}H NMR 400MHz, $CDCl_3$)

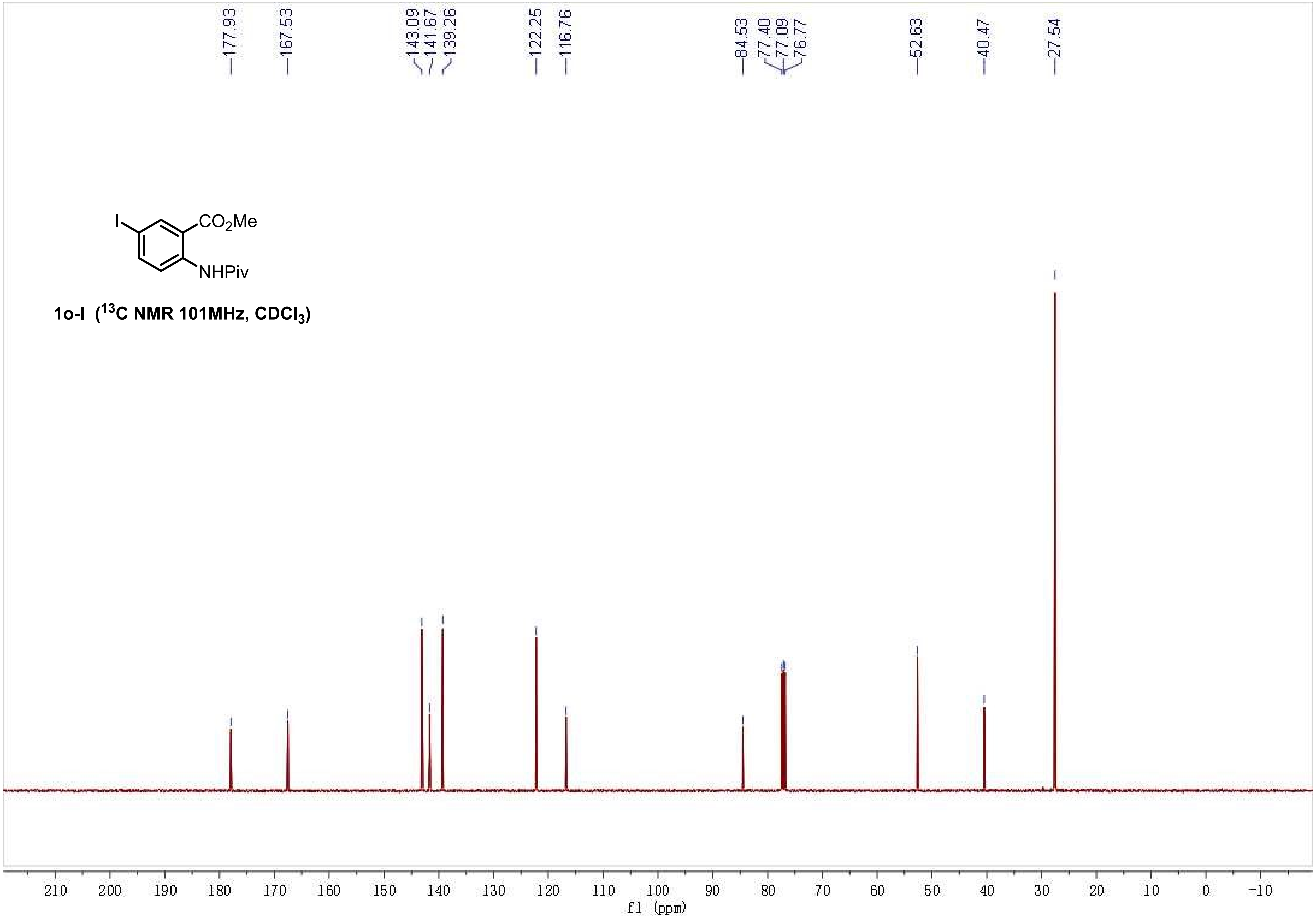
177.93
167.53
143.09
141.67
139.26
122.25
116.76
84.53
77.40
77.09
76.77
52.63
40.47
27.54
I
CO_2Me
NHPiv
1o-I (^{13}C NMR 101MHz, $CDCl_3$)
210 200 190 180 170 160 150 140 130 120 110 100 90 80 70 60 50 40 30 20 10 0 -10
f1 (ppm)

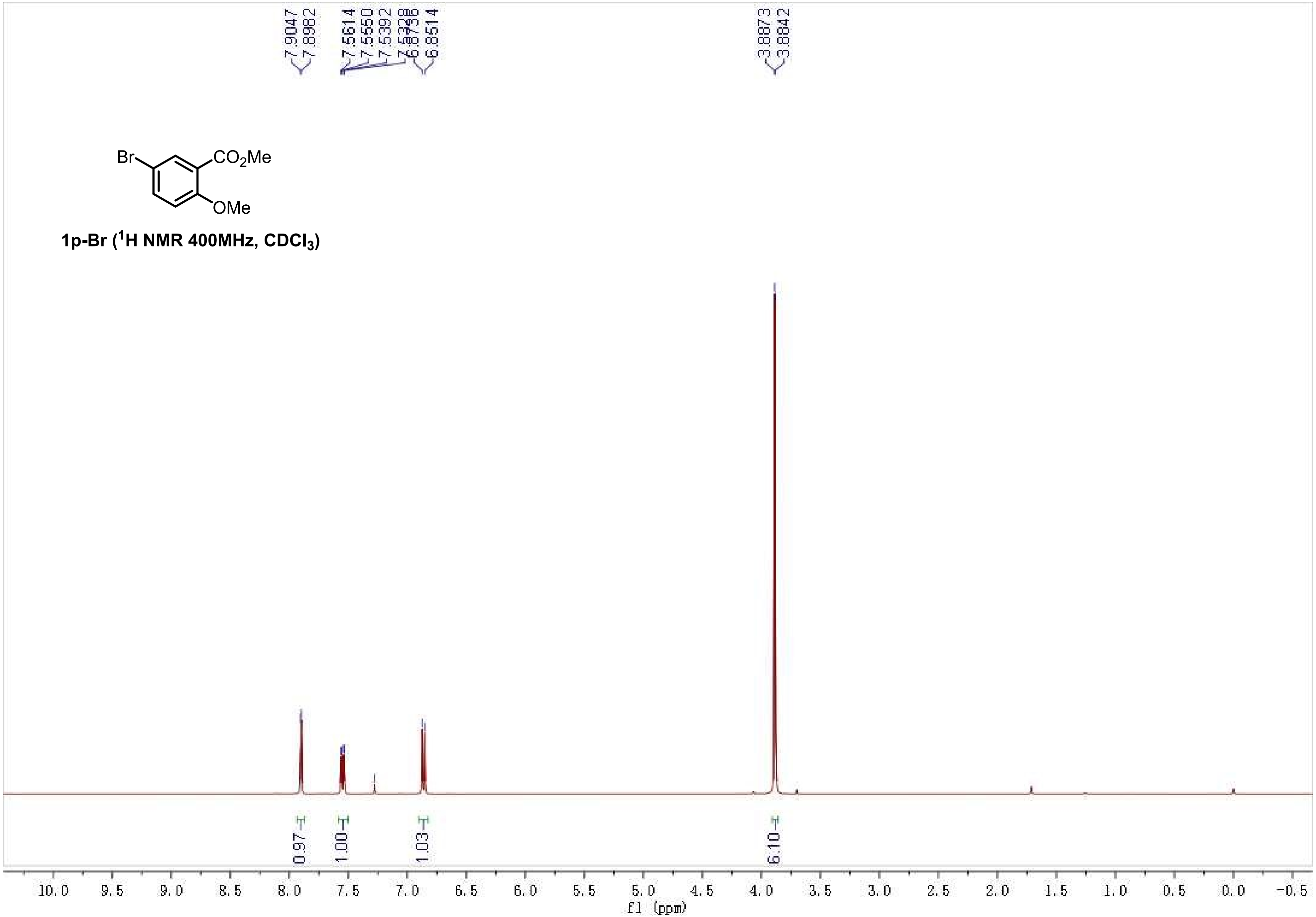

1p-Br (^{1}H NMR 400MHz, $CDCl_3$)

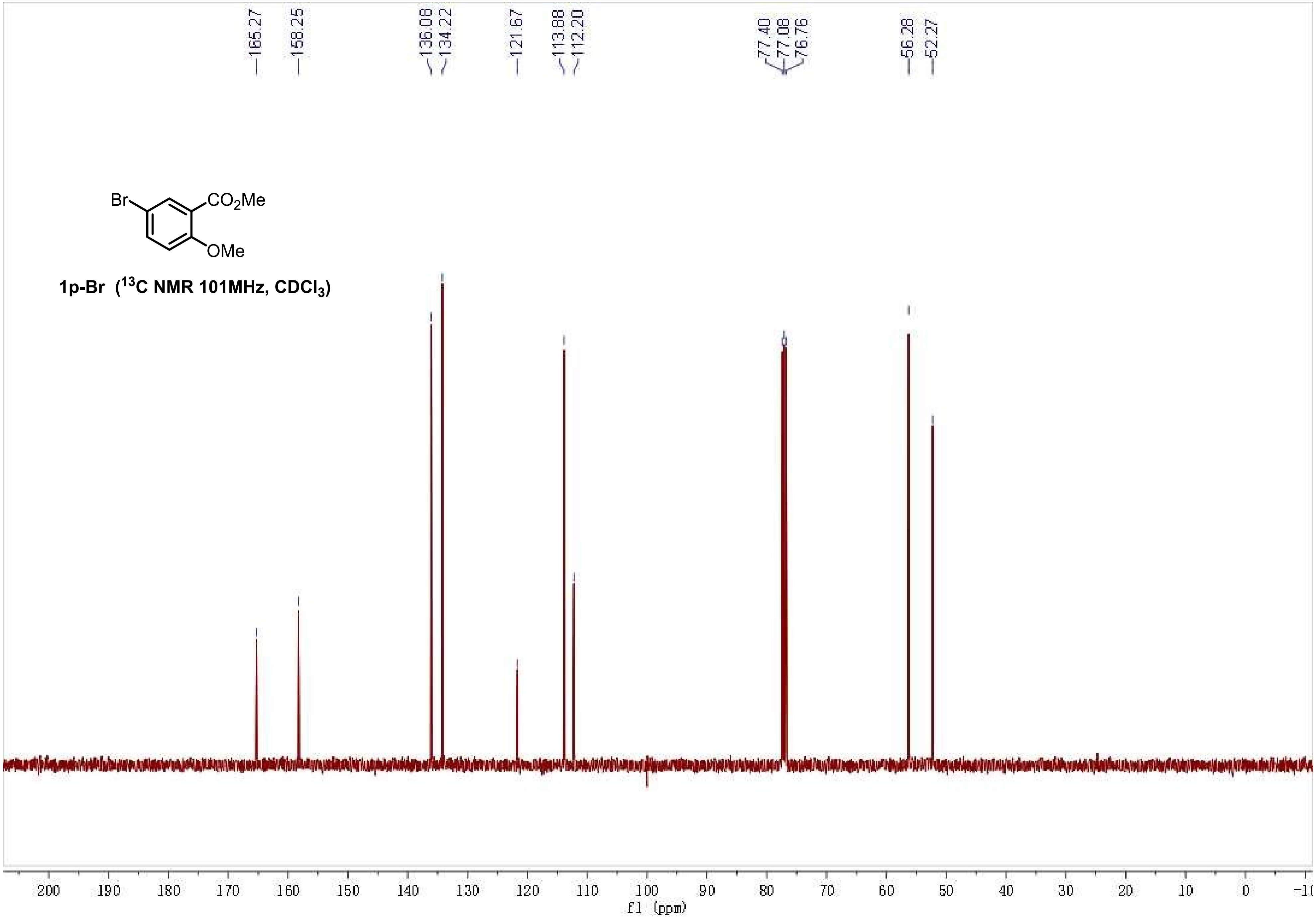
165.27
158.25
136.08
134.22
121.67
113.88
112.20
77.40
77.08
76.76
56.28
52.27
Br
CO2Me
OMe
1p-Br (13C NMR 101MHz, CDCl3)
200 190 180 170 160 150 140 130 120 110 100 90 80 70 60 50 40 30 20 10 0 -10
f1 (ppm)

1p-I (^1H NMR 400MHz, $CDCl_3$)

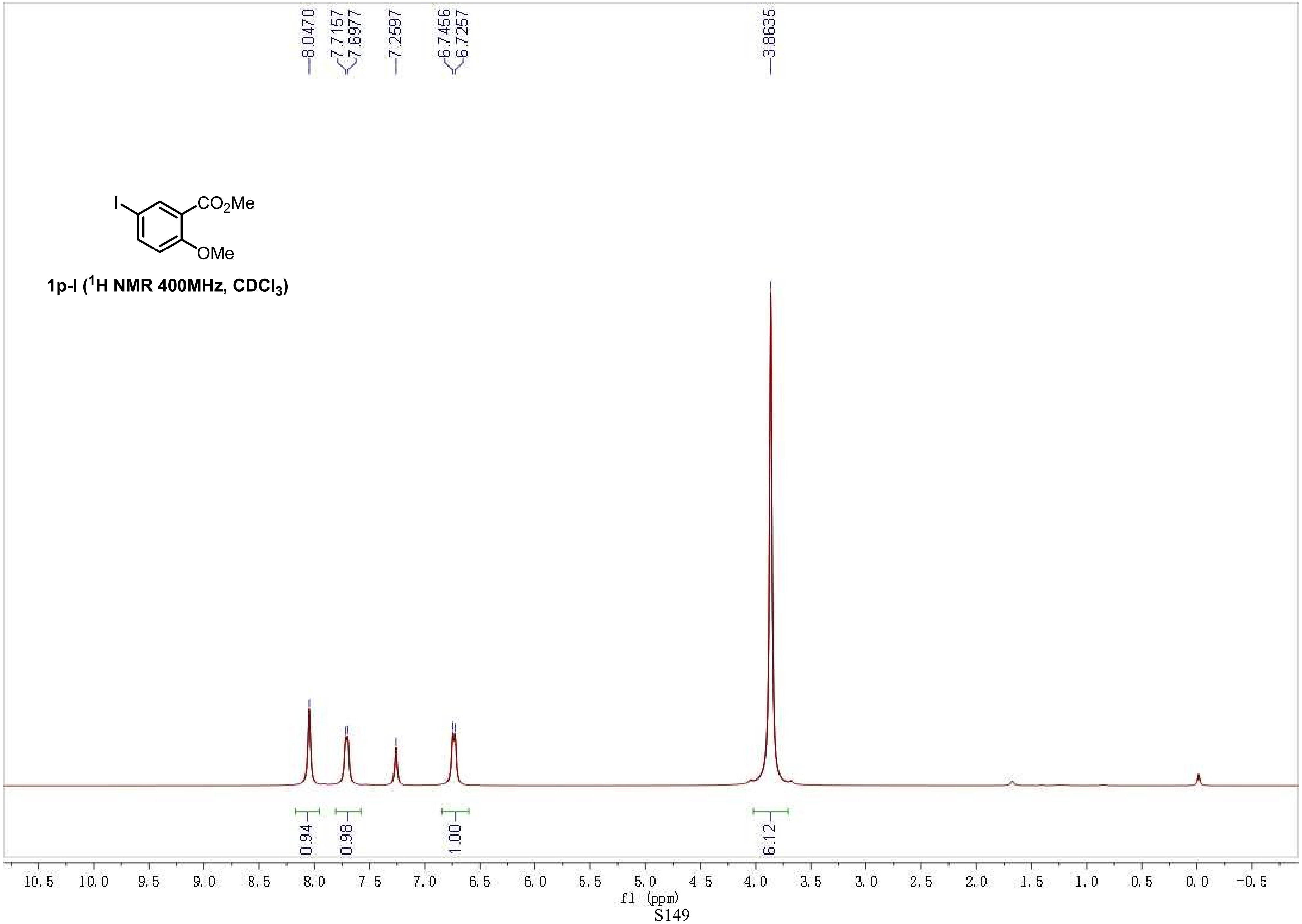

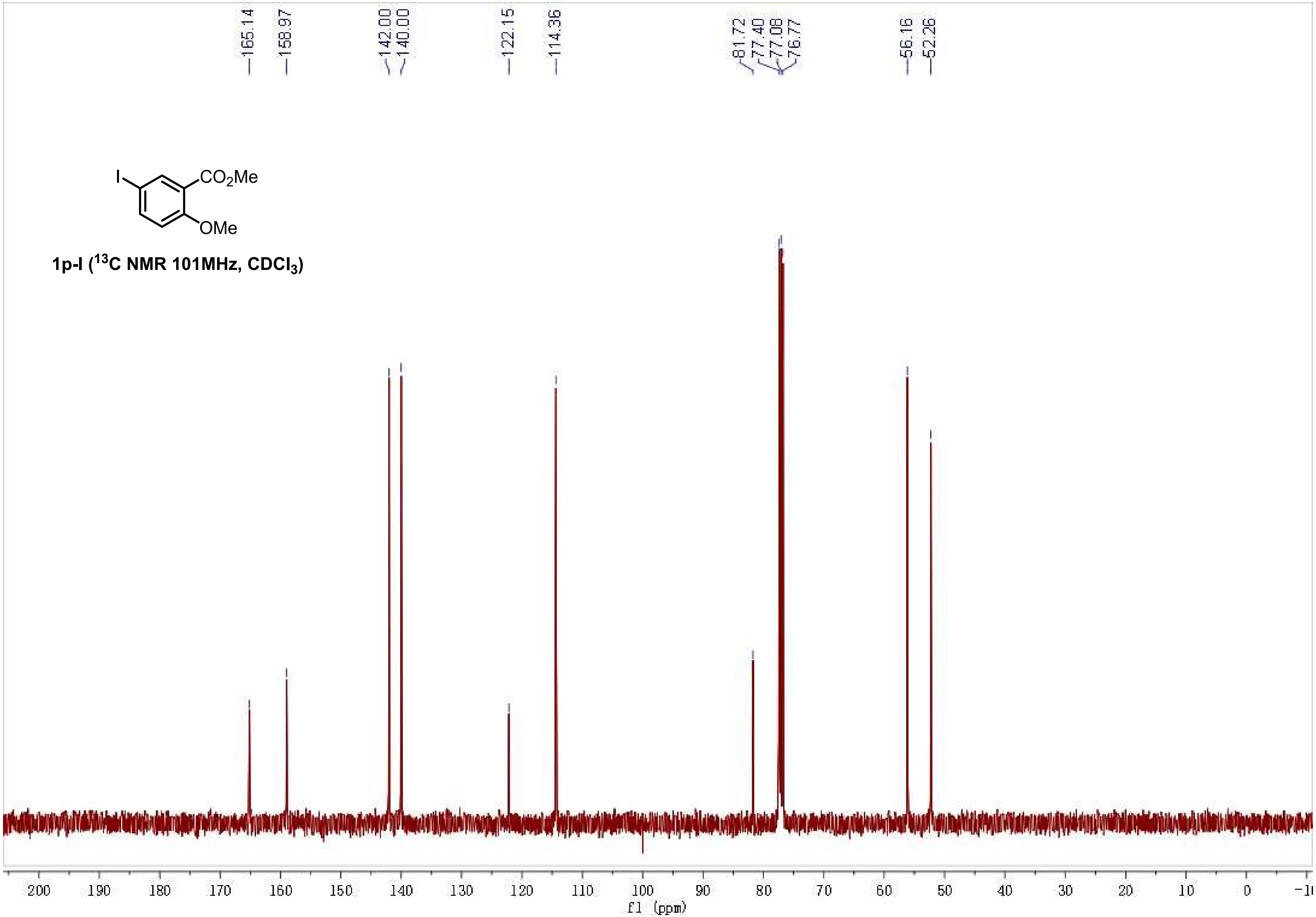

1p-I (^{13}C NMR 101MHz, $CDCl_3$)

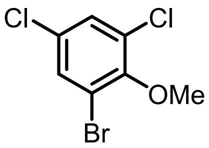

1q-Br (^1H NMR 400MHz, $CDCl_3$)

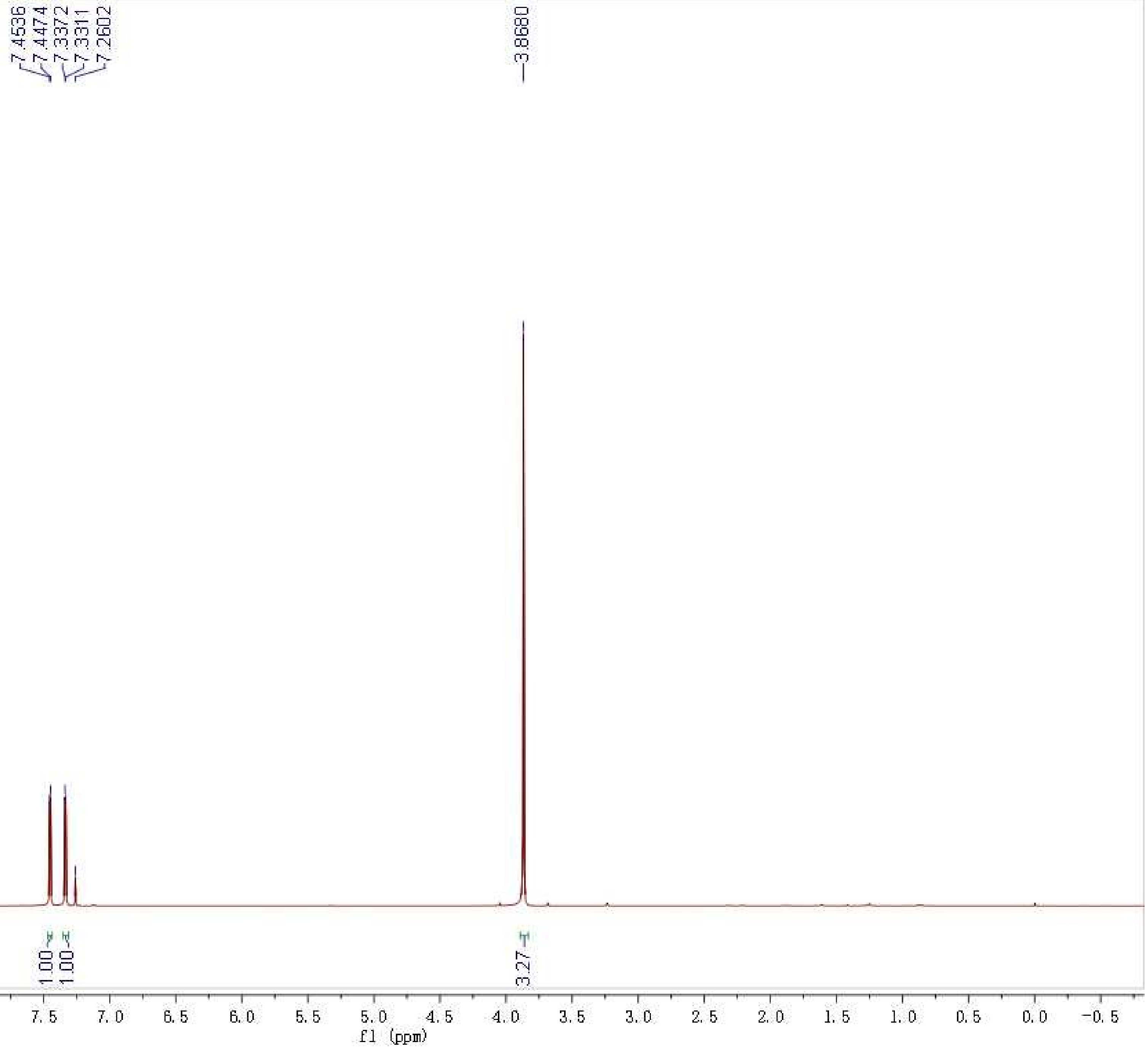

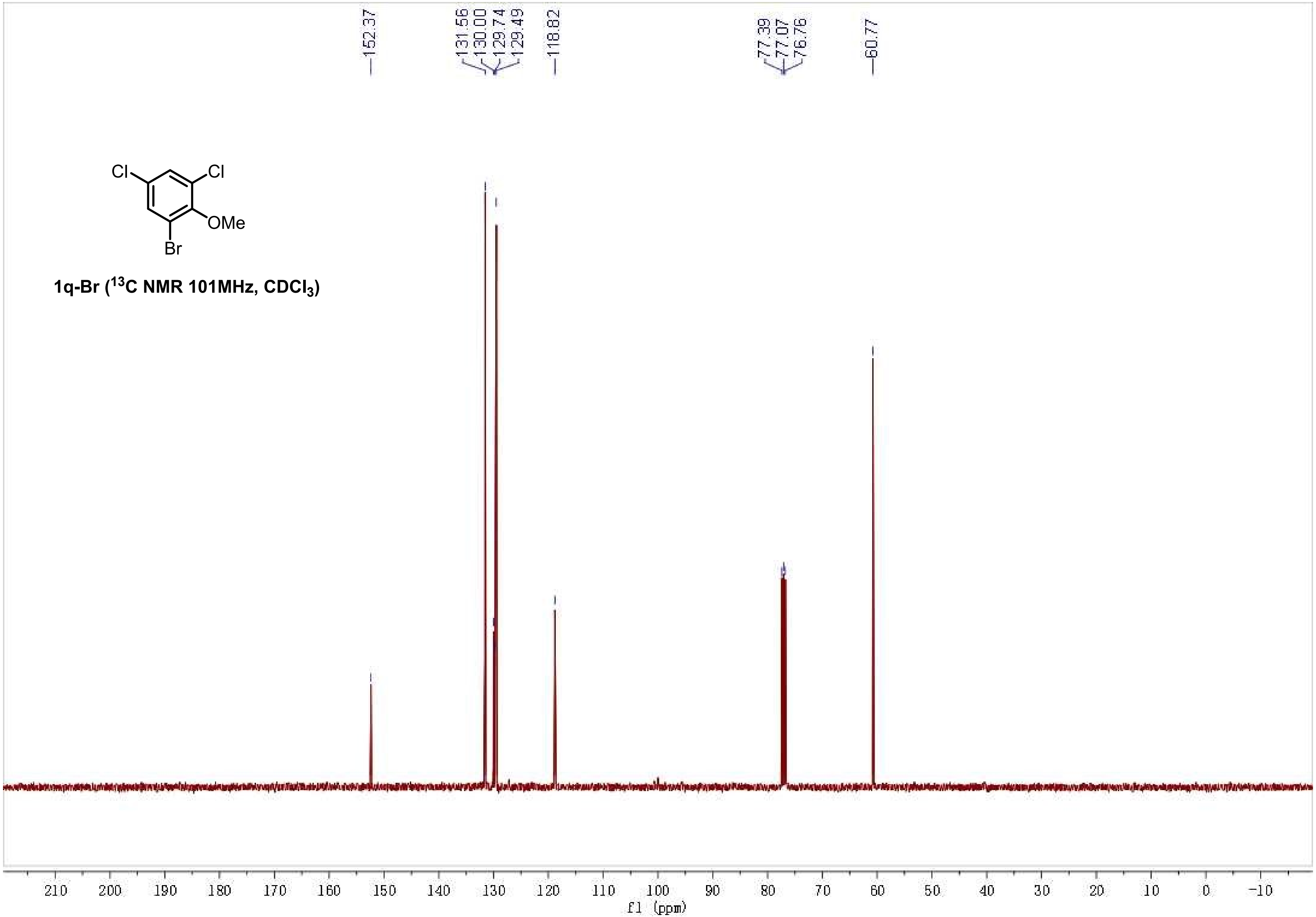
152.37
131.56
130.00
129.74
129.49
118.82
77.39
77.07
76.76
60.77
Cl
Cl
OMe
Br
1q-Br (^{13}C NMR 101MHz, $CDCl_3$)
210 200 190 180 170 160 150 140 130 120 110 100 90 80 70 60 50 40 30 20 10 0 -10
f1 (ppm)

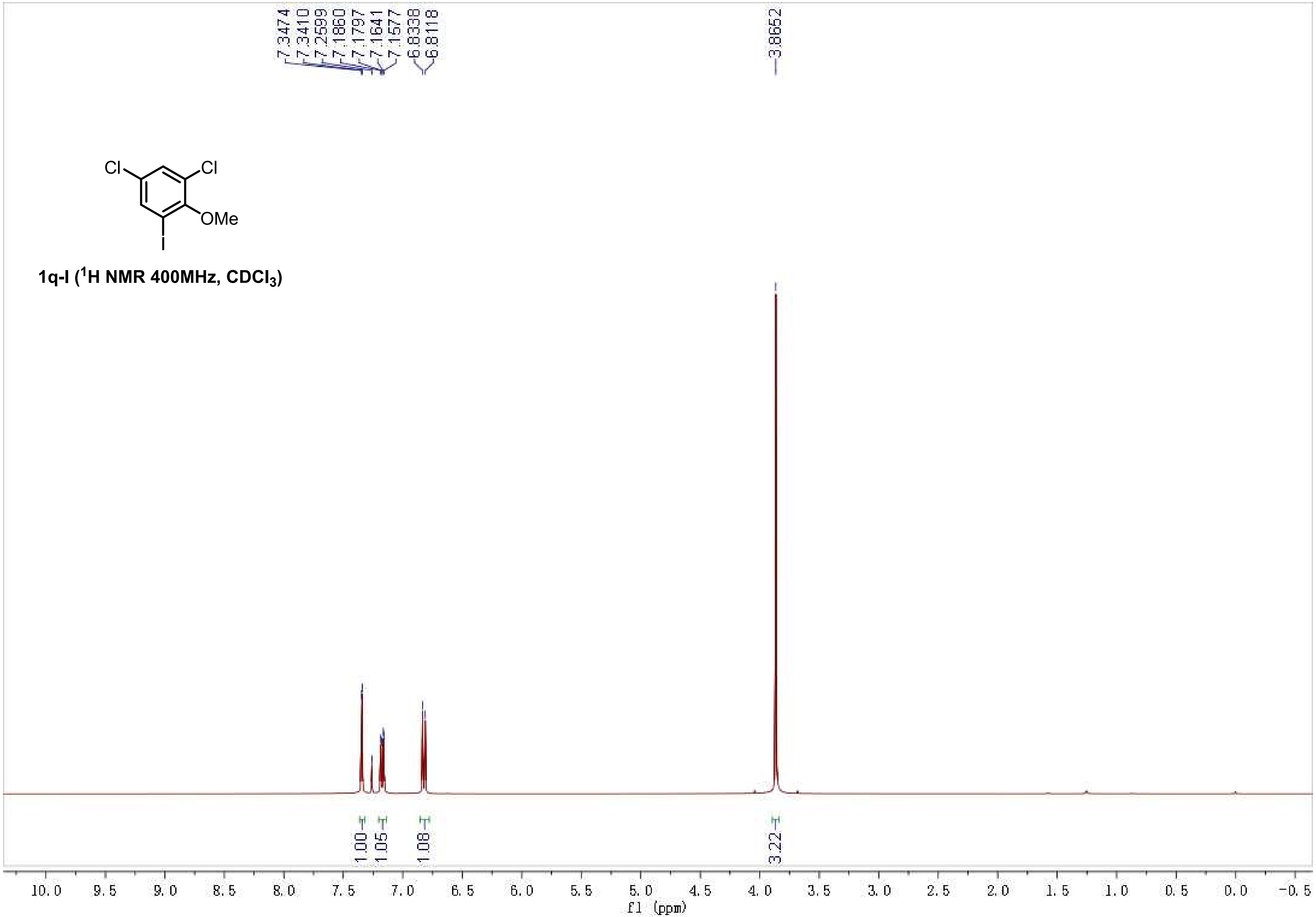

7.3474
7.3410
7.2599
7.1860
7.1797
7.1641
7.1577
6.8338
6.8118
3.8652
Cl
Cl
OMe
I
1q-I (1H NMR 400MHz, CDCl3)
1.00
1.05
1.08
3.22
10.0 9.5 9.0 8.5 8.0 7.5 7.0 6.5 6.0 5.5 5.0 4.5 4.0 3.5 3.0 2.5 2.0 1.5 1.0 0.5 0.0 -0.5
f1 (ppm)

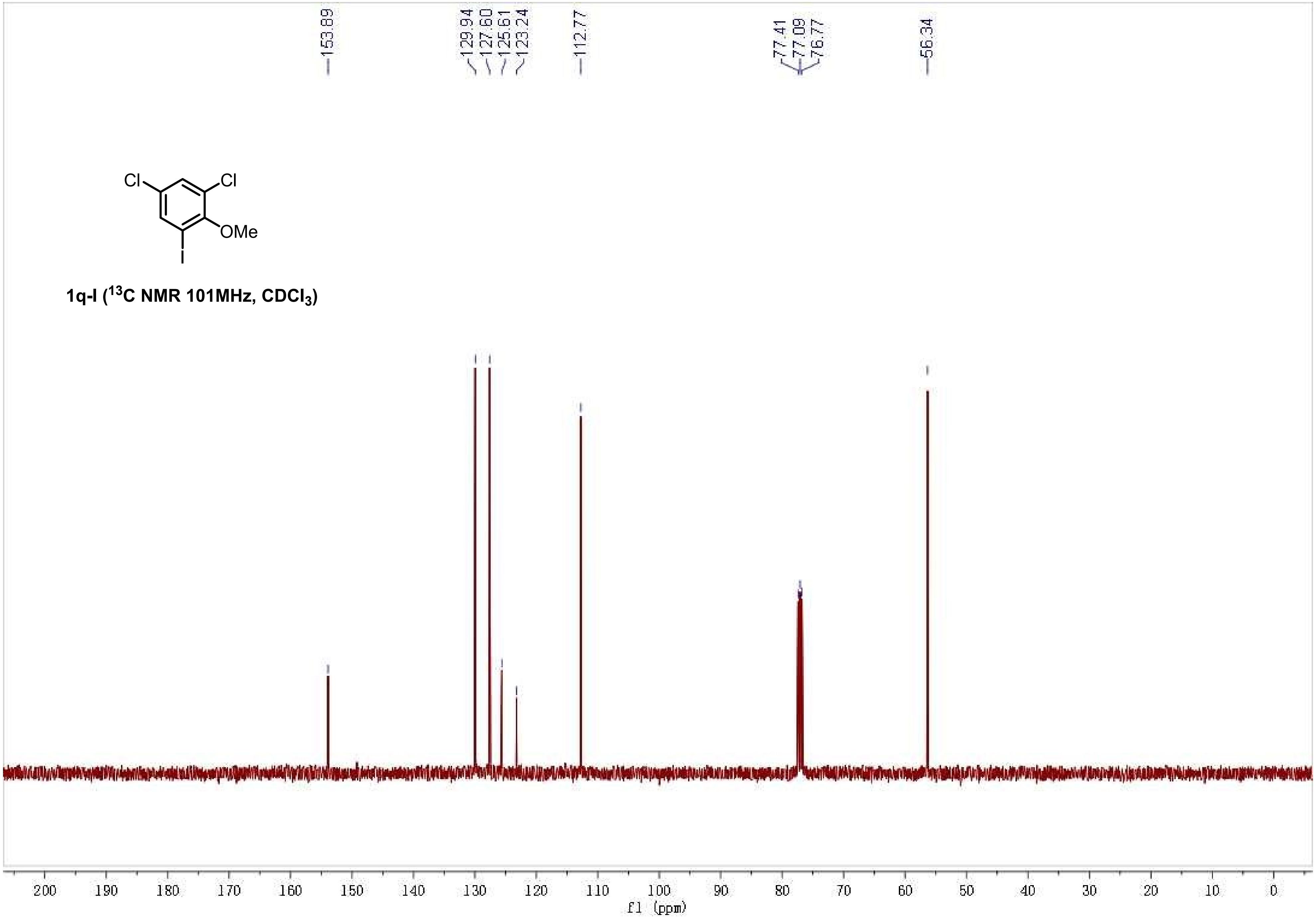

1q-I (^{13}C NMR 101MHz, $CDCl_3$)

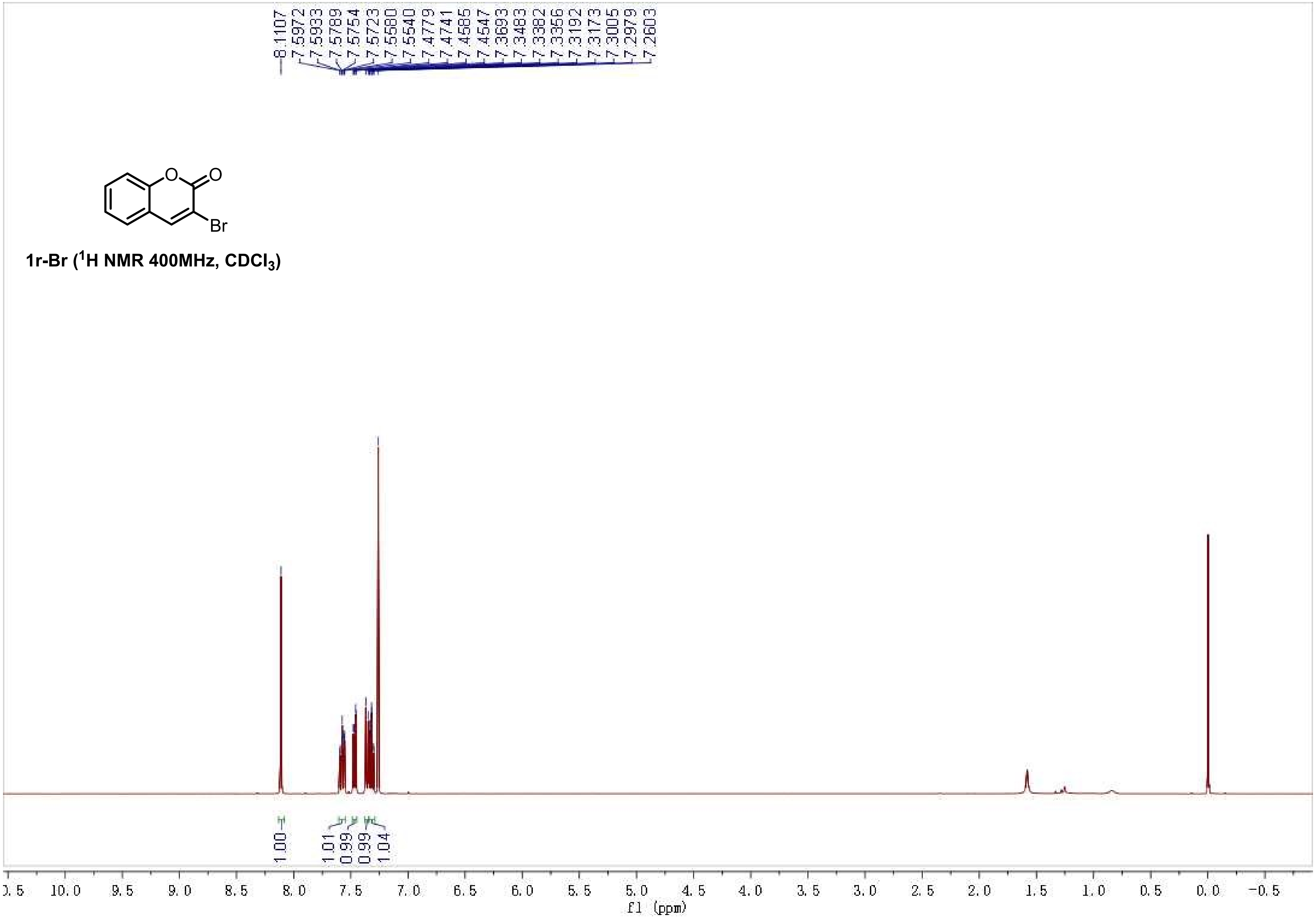

1r-Br (^{1}H NMR 400MHz, $CDCl_3$)

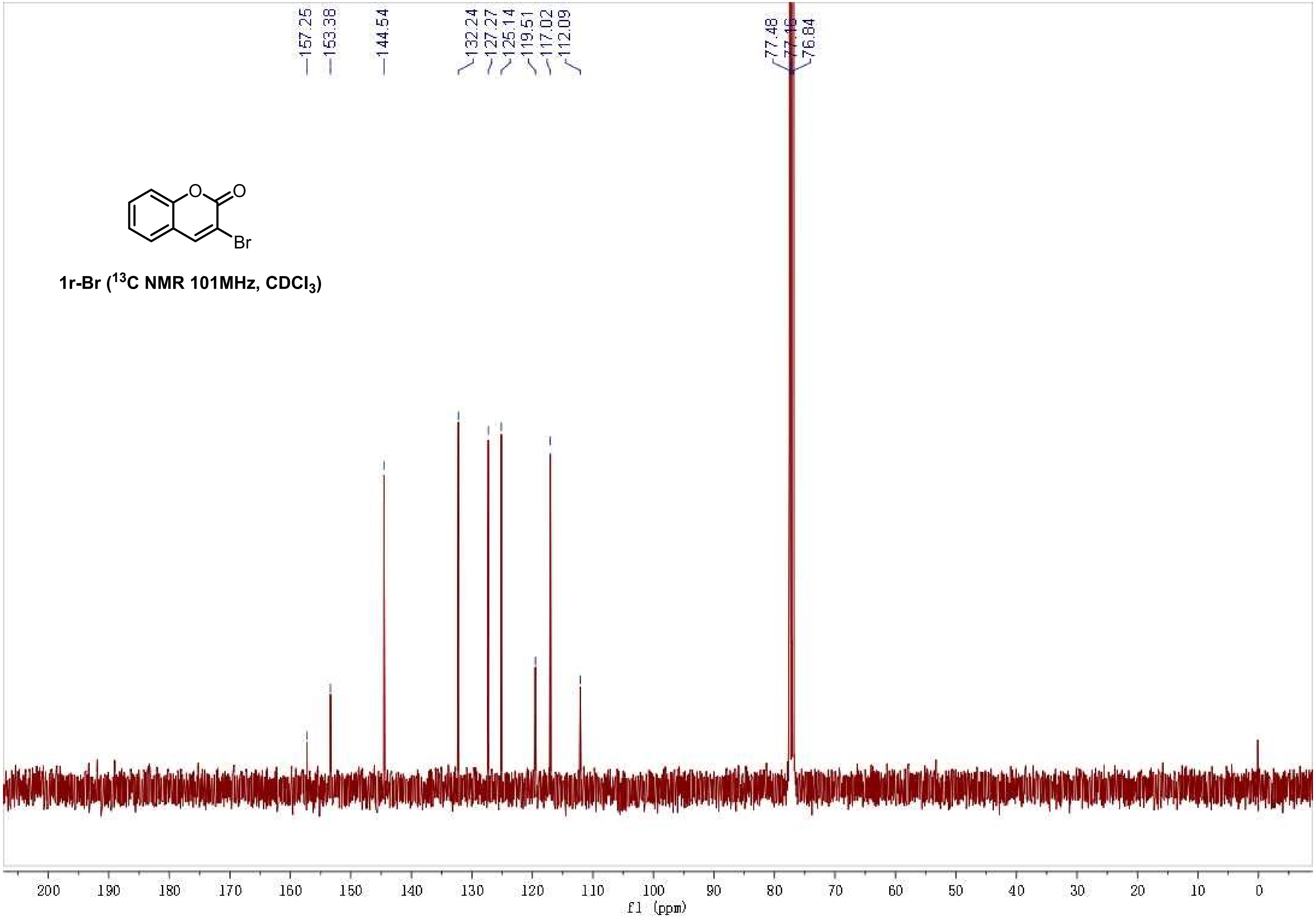

1r-Br (^{13}C NMR 101MHz, $CDCl_3$)

1s-Cl_2 (^{1}H NMR 400MHz, $CDCl_3$)

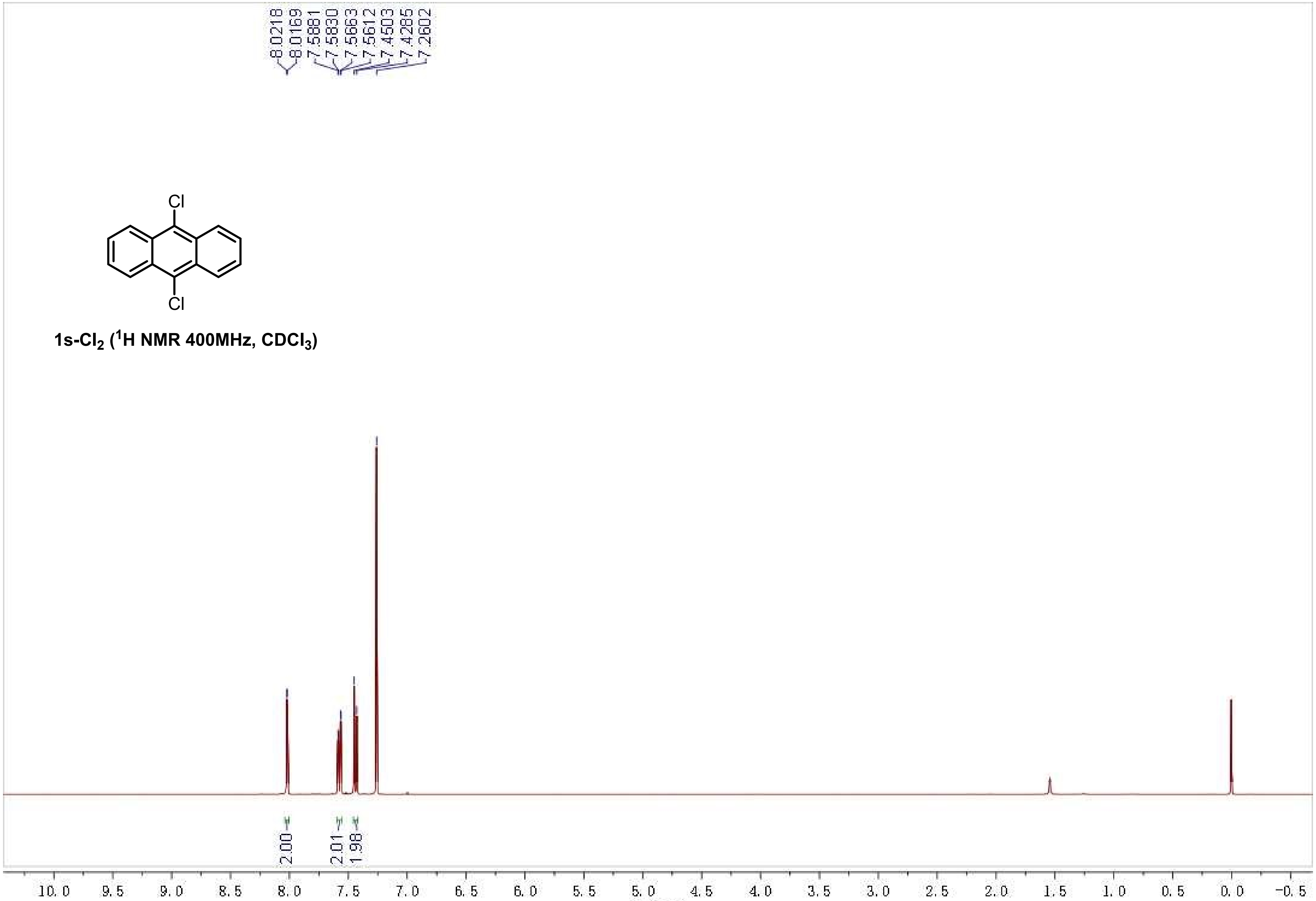

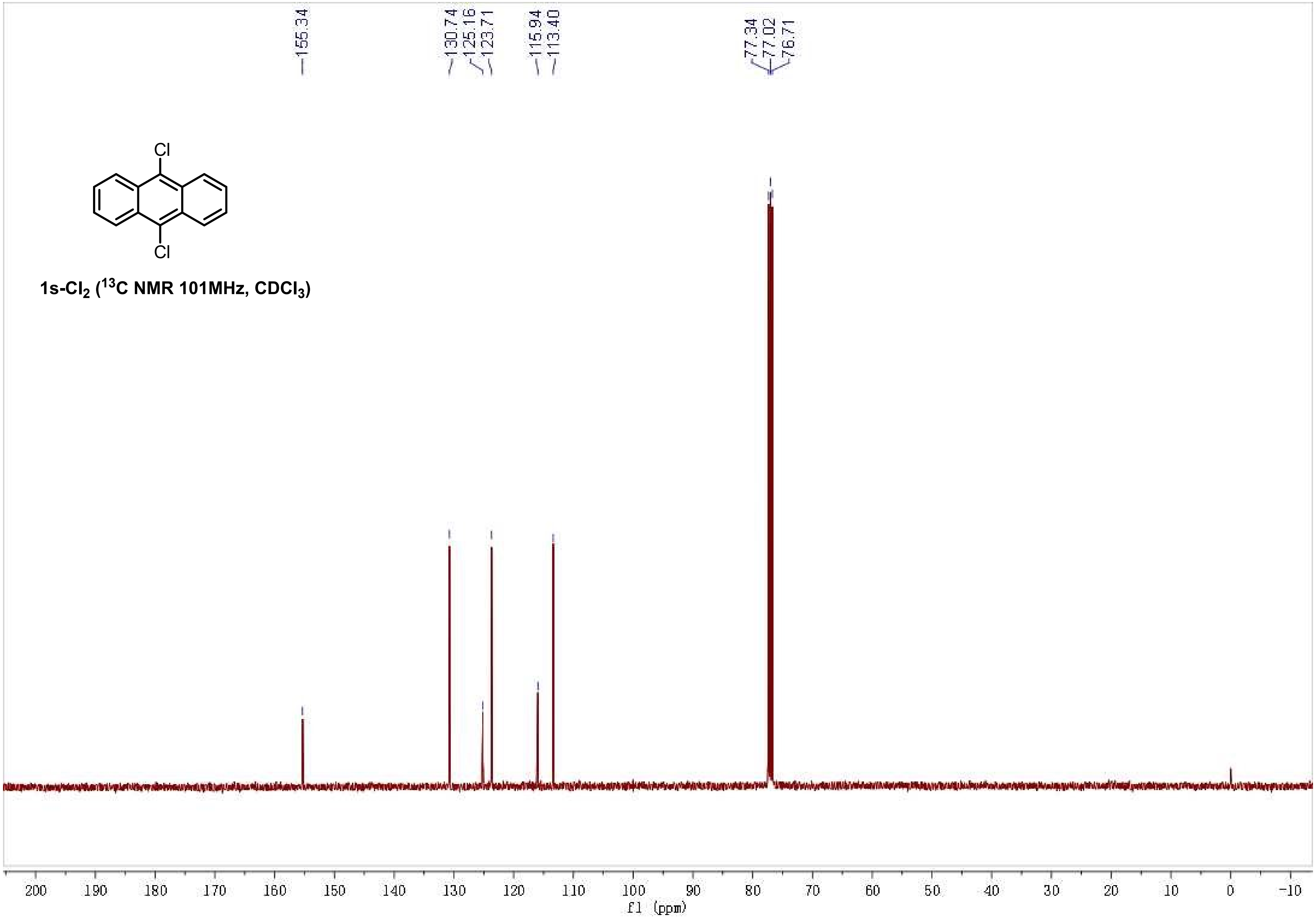

1s-Cl_2 (^{13}C NMR 101MHz, $CDCl_3$)

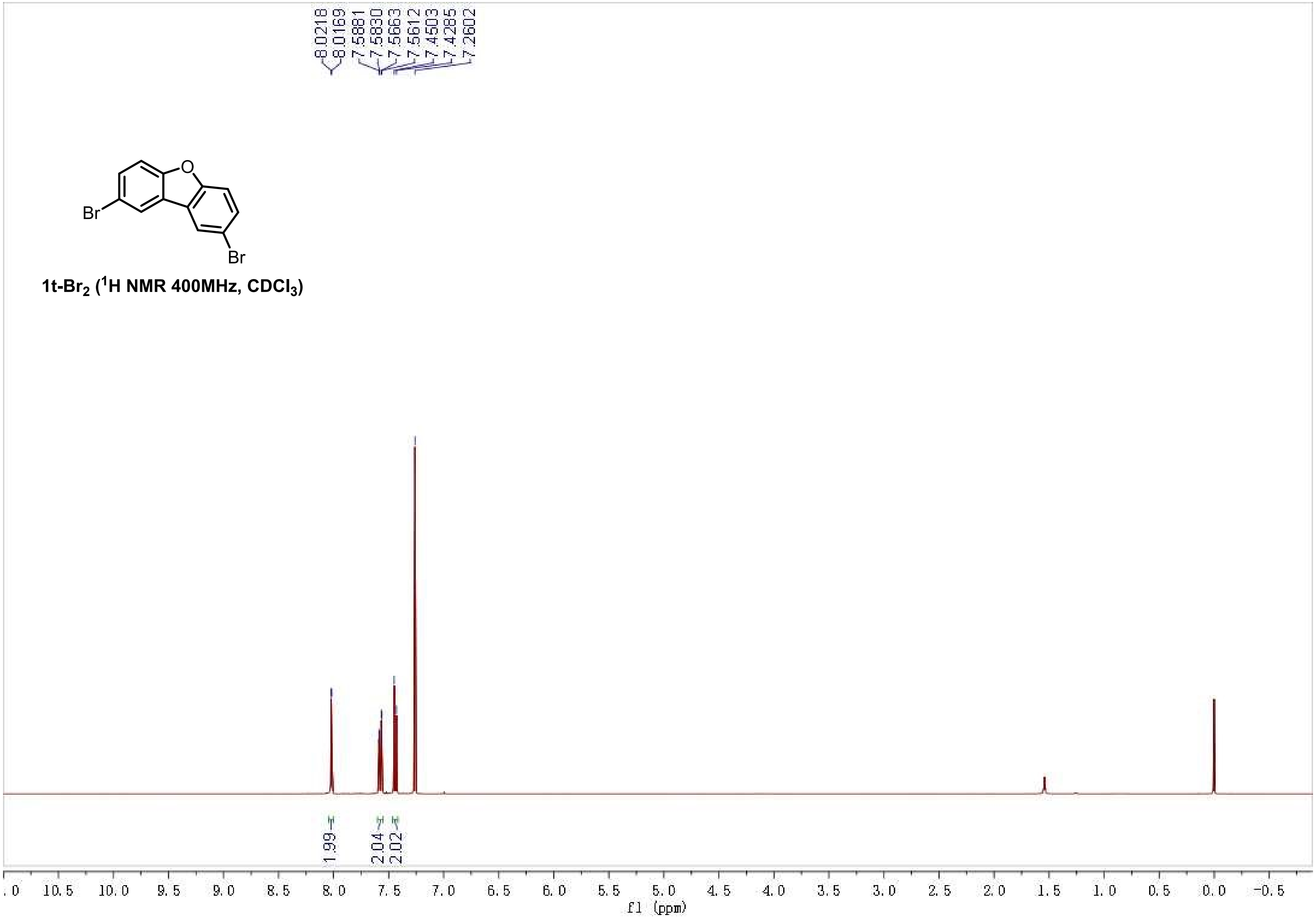

1t-Br$_2$ (^{1}H NMR 400MHz, $CDCl_3$)

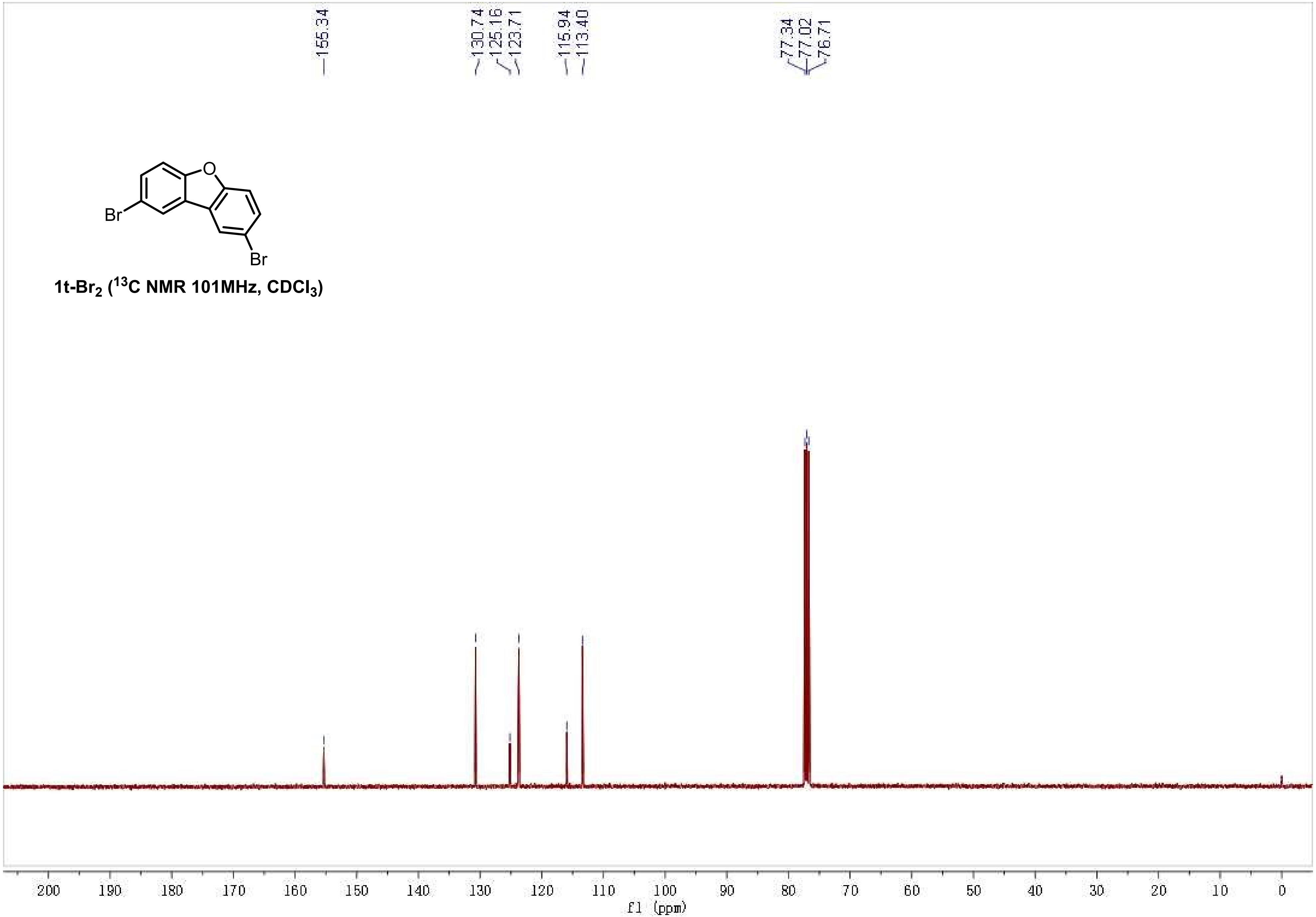
155.34
130.74
125.16
123.71
115.94
113.40
77.34
77.02
76.71
Br
O
Br
1t-Br$_2$ (^{13}C NMR 101MHz, CDCl$_3$)
200 190 180 170 160 150 140 130 120 110 100 90 80 70 60 50 40 30 20 10 0
f1 (ppm)

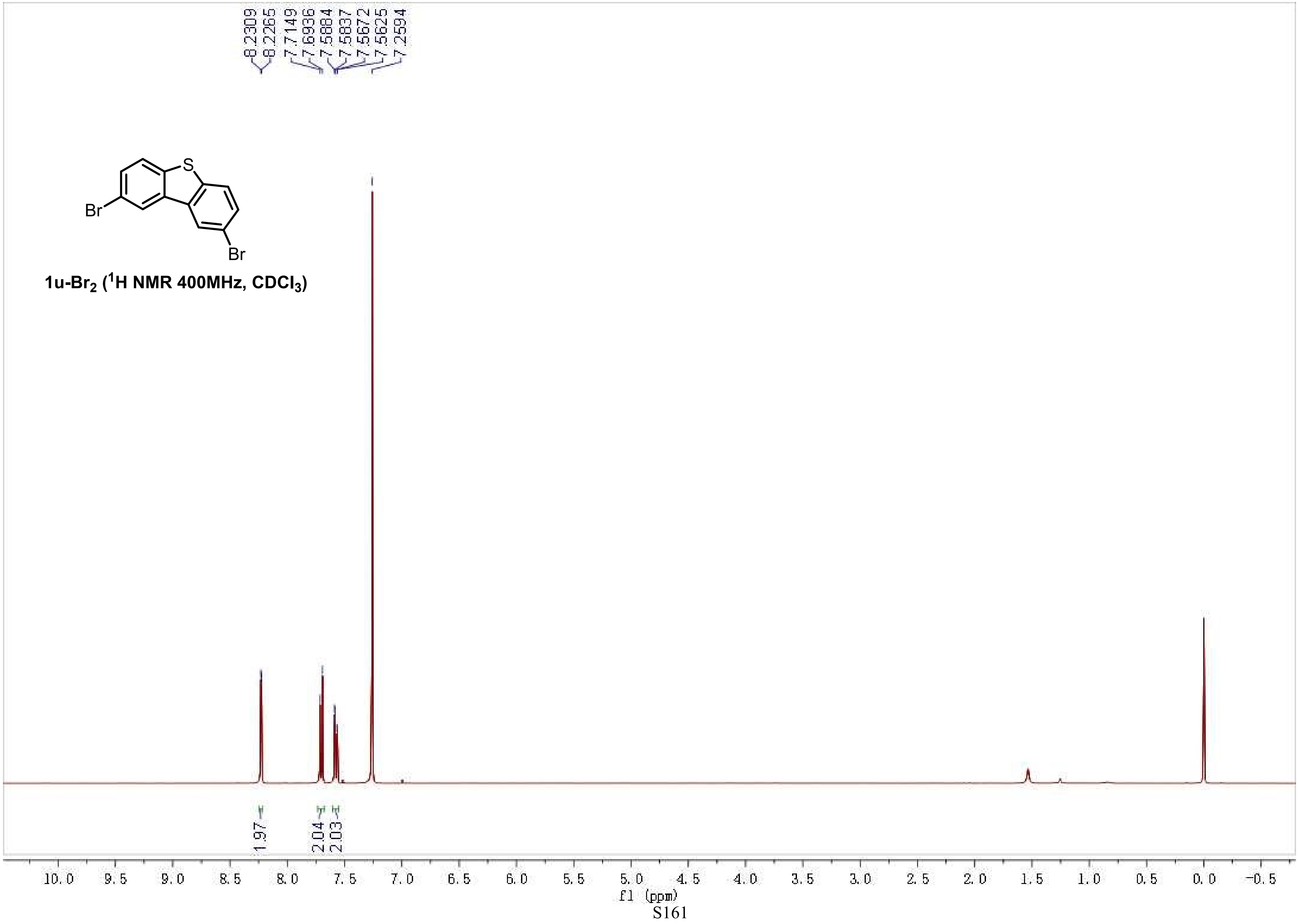
8.2309
8.2265
7.7149
7.6936
7.5884
7.5837
7.5672
7.5625
7.2594
Br
S
Br
1u-Br$_2$ (^{1}H NMR 400MHz, CDCl$_3$)
1.97
2.04
2.03
10.0
9.5
9.0
8.5
8.0
7.5
7.0
6.5
6.0
5.5
5.0
4.5
4.0
3.5
3.0
2.5
2.0
1.5
1.0
0.5
0.0
-0.5
f1 (ppm)

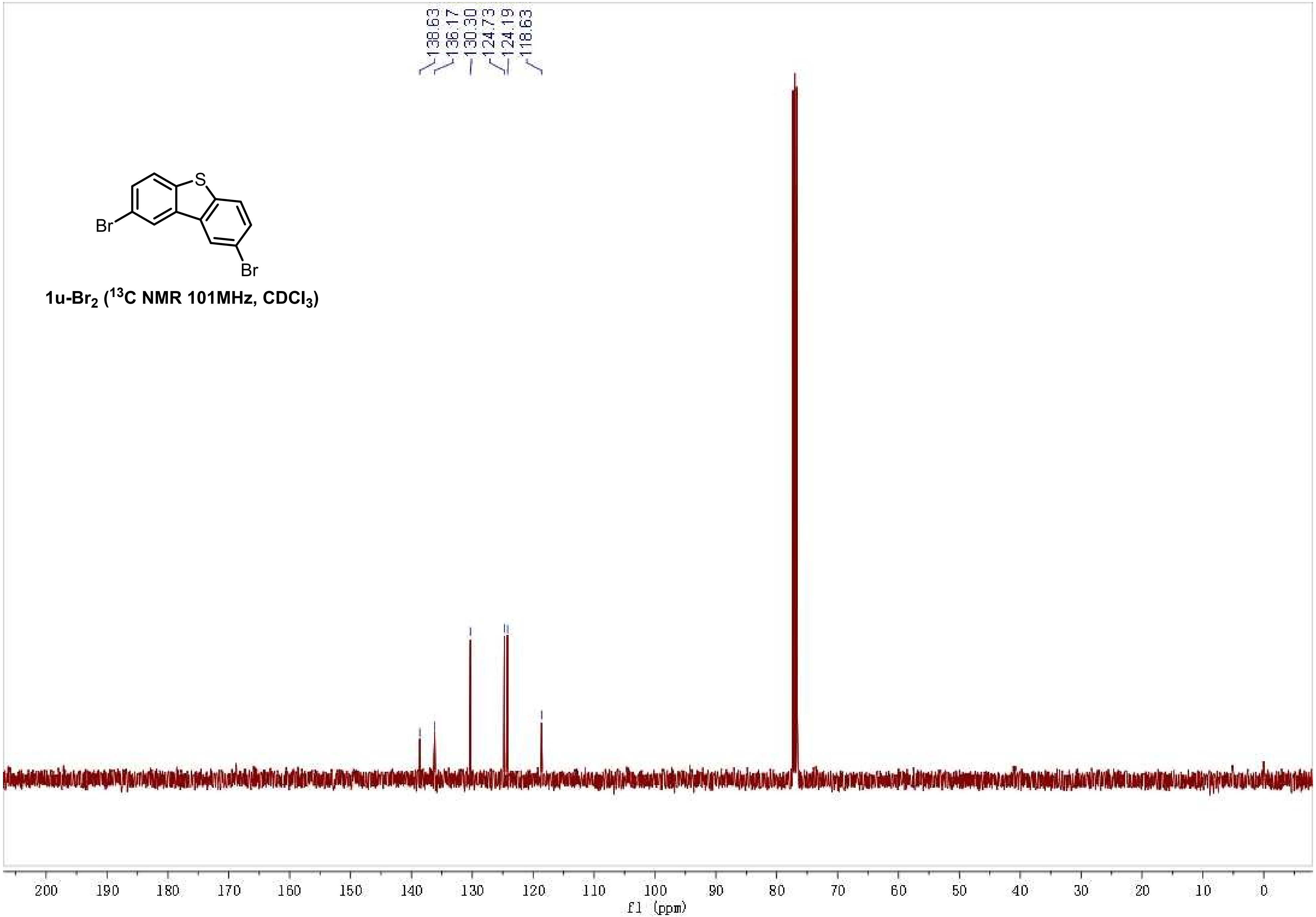
138.63
136.17
130.30
124.73
124.19
118.63
Br
S
Br
1u-Br$_2$ (^{13}C NMR 101MHz, CDCl$_3$)
200 190 180 170 160 150 140 130 120 110 100 90 80 70 60 50 40 30 20 10 0
f1 (ppm)

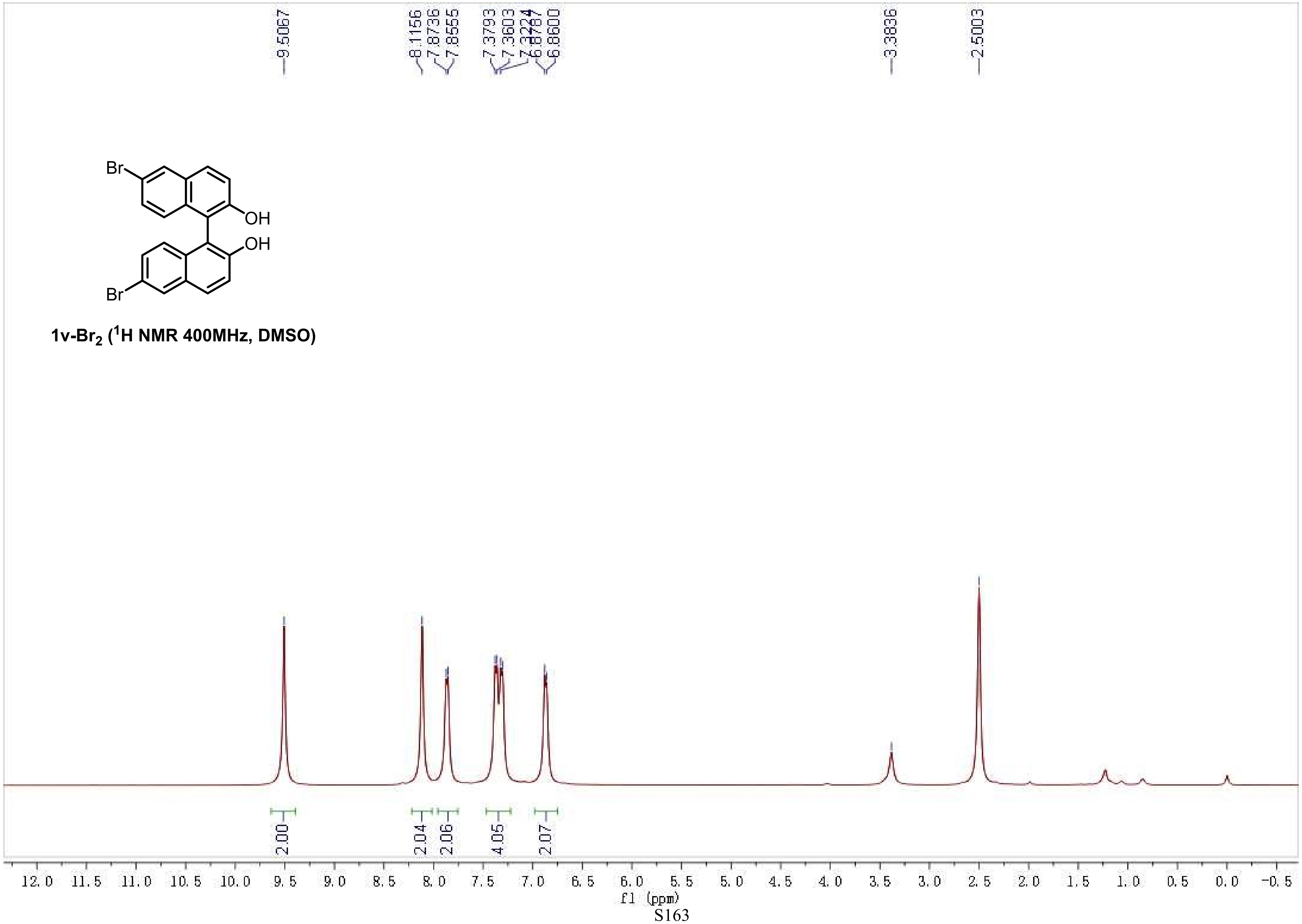

9.5067
8.1156
7.8736
7.8555
7.3793
7.3603
7.3224
6.8787
6.8600
3.3836
2.5003
Br
OH
OH
Br
1v-Br$_2$ (^{1}H NMR 400MHz, DMSO)
2.00
2.04
2.06
4.05
2.07
12.0 11.5 11.0 10.5 10.0 9.5 9.0 8.5 8.0 7.5 7.0 6.5 6.0 5.5 5.0 4.5 4.0 3.5 3.0 2.5 2.0 1.5 1.0 0.5 0.0 -0.5
f1 (ppm)

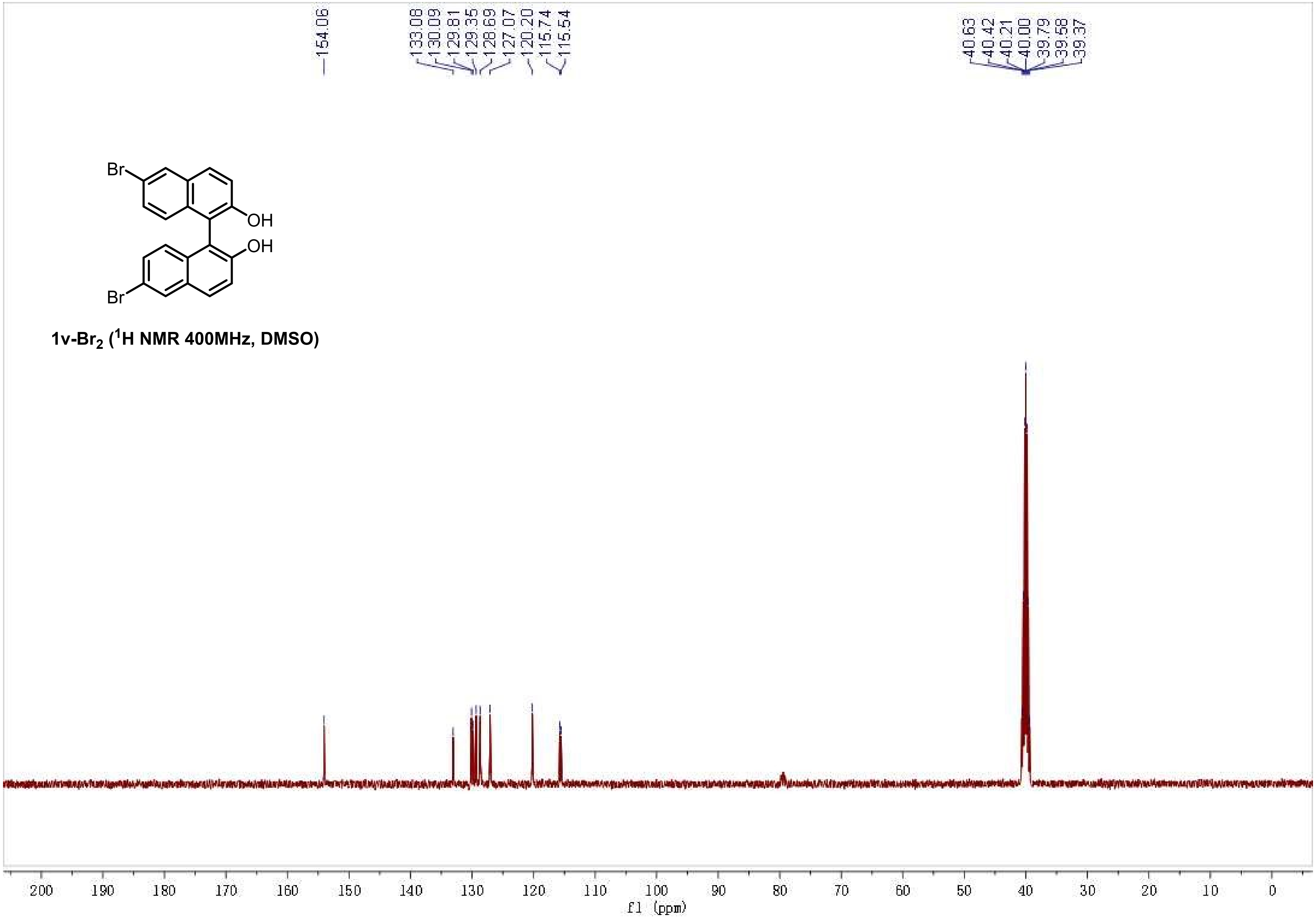

1v-Br$_2$ (^{1}H NMR 400MHz, DMSO)

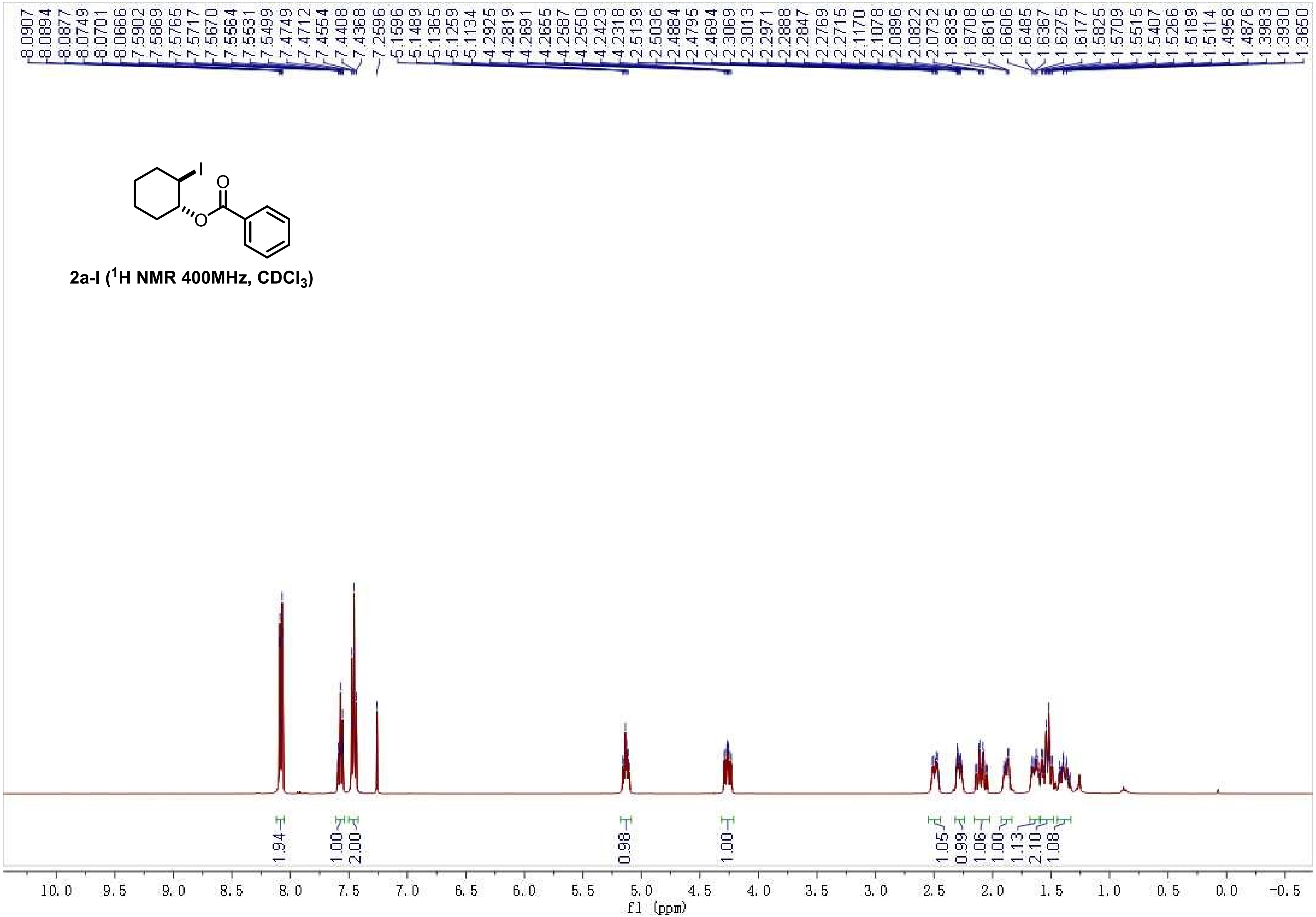
2a-I (^{1}H NMR 400MHz, $CDCl_3$)
f1 (ppm)

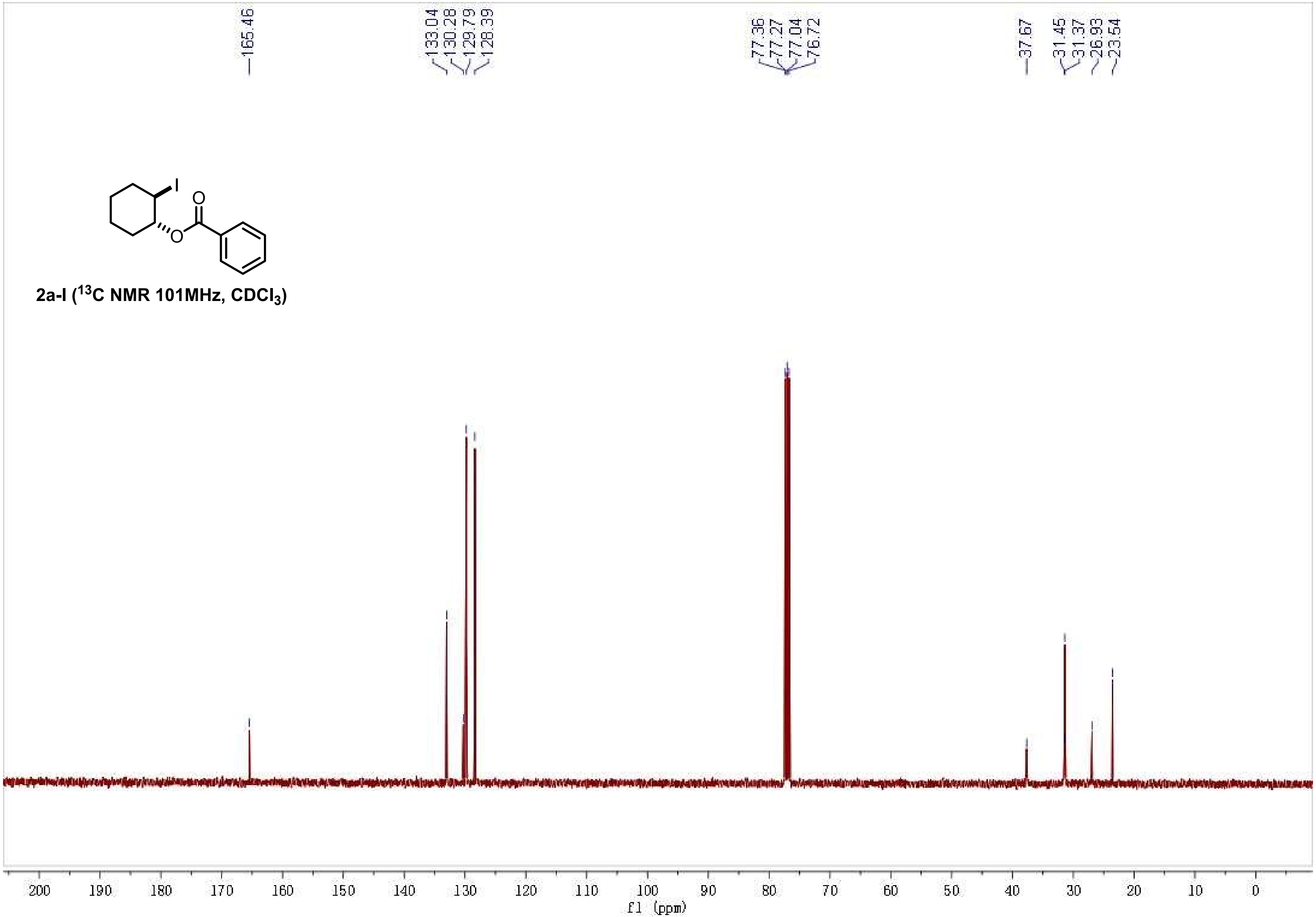

2a-I (^{13}C NMR 101MHz, $CDCl_3$)

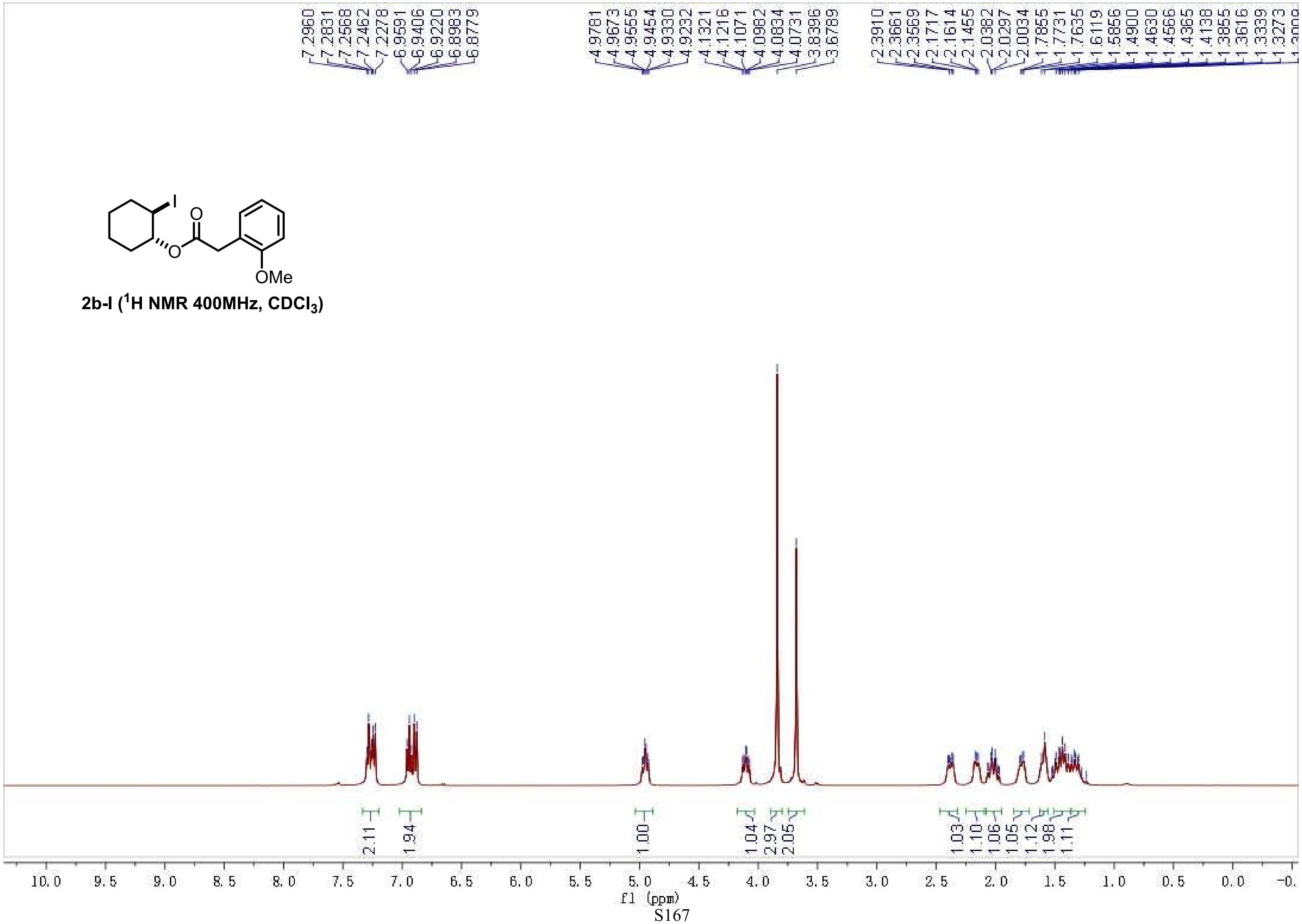

2b-I (^{1}H NMR 400MHz, $CDCl_3$)

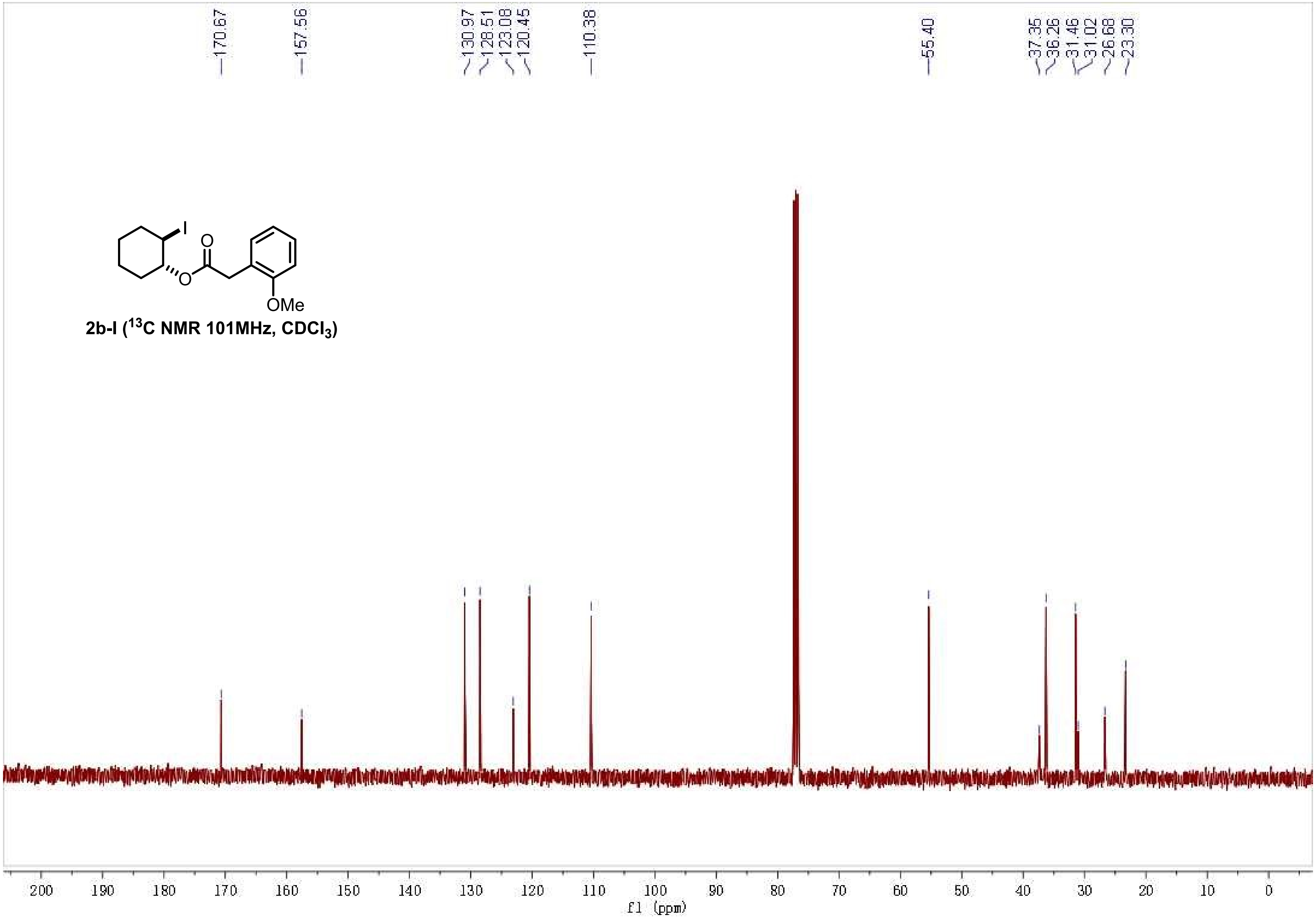

2b-I (^{13}C NMR 101MHz, $CDCl_3$)

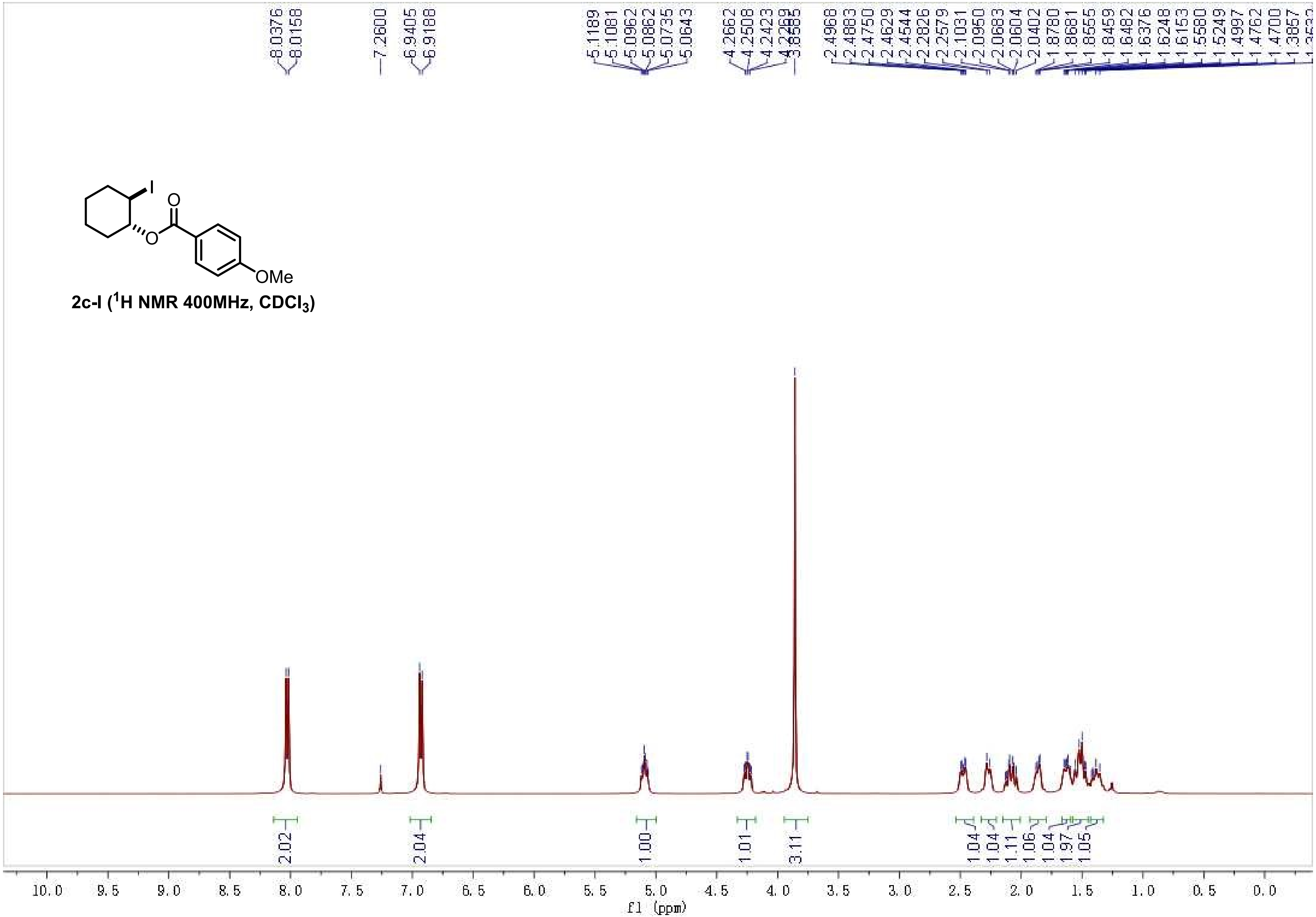

2c-I (^{1}H NMR 400MHz, $CDCl_3$)

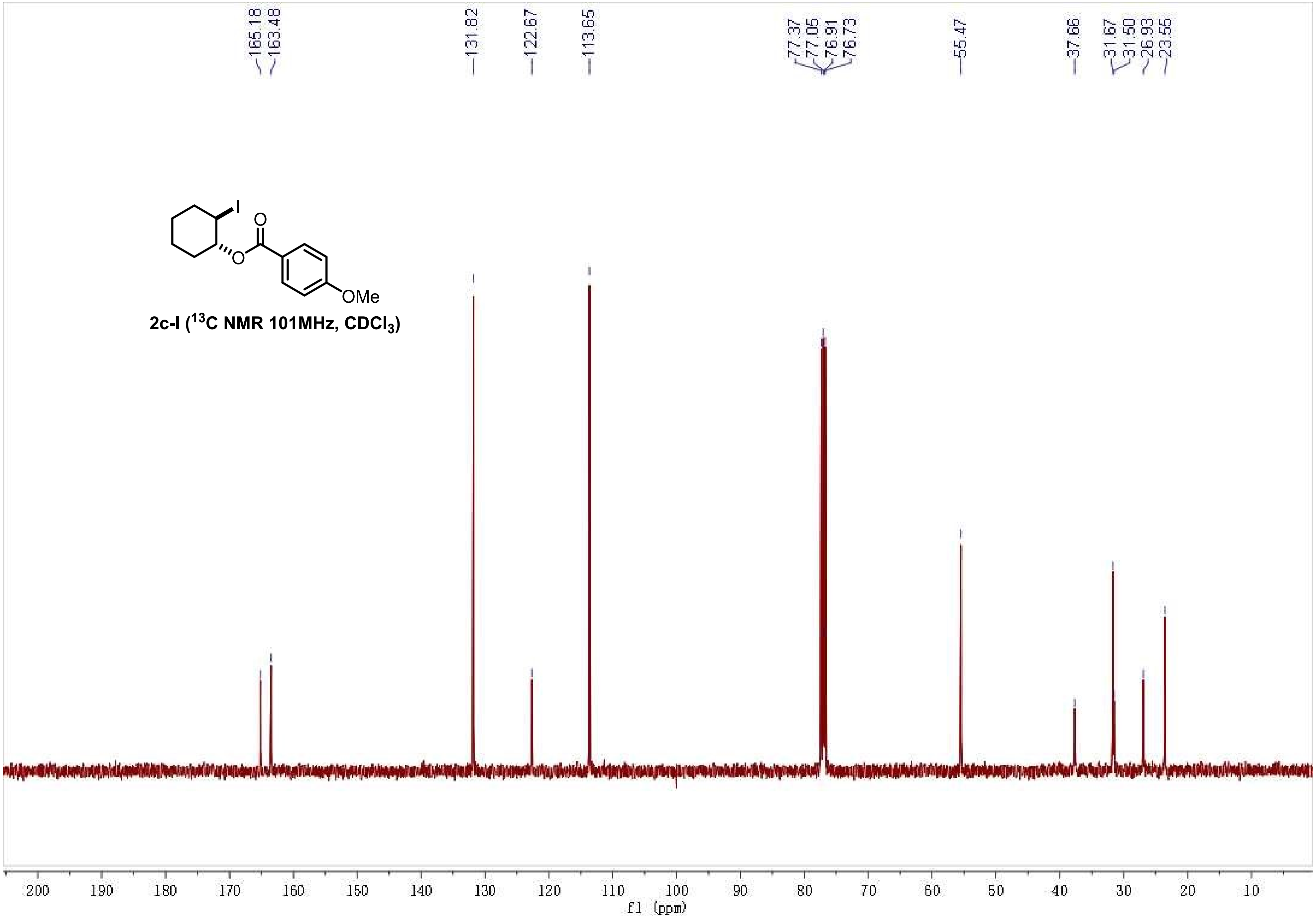
165.18
163.48
131.82
122.67
113.65
77.37
77.05
76.91
76.73
55.47
37.66
31.67
31.50
26.93
23.55
O
O
I
OMe
2c-I (13C NMR 101MHz, CDCl3)
200 190 180 170 160 150 140 130 120 110 100 90 80 70 60 50 40 30 20 10
f1 (ppm)

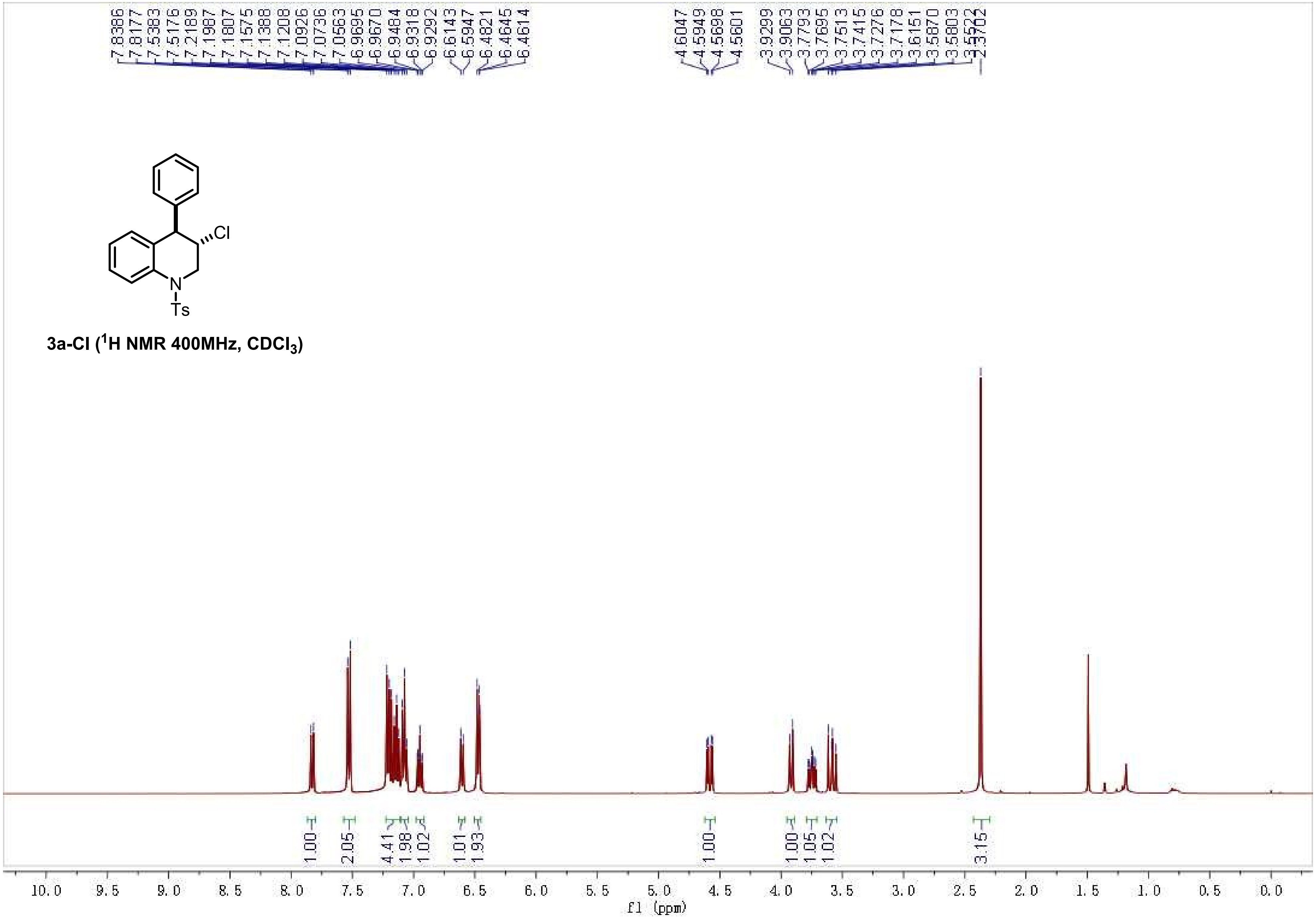
7.8386
7.8177
7.5383
7.5176
7.2189
7.1987
7.1807
7.1575
7.1388
7.1208
7.0926
7.0736
7.0563
6.9695
6.9670
6.9484
6.9318
6.9292
6.6143
6.5947
6.4821
6.4645
6.4614
4.6047
4.5949
4.5698
4.5601
3.9299
3.9063
3.7793
3.7695
3.7513
3.7415
3.7276
3.7178
3.6151
3.5870
3.5803
3.5522
2.3702
Cl
N
Ts
3a-Cl (^{1}H NMR 400MHz, $CDCl_3$)
1.00
2.05
4.41
1.98
1.02
1.01
1.93
1.00
1.00
1.05
1.02
3.15
10.0 9.5 9.0 8.5 8.0 7.5 7.0 6.5 6.0 5.5 5.0 4.5 4.0 3.5 3.0 2.5 2.0 1.5 1.0 0.5 0.0
f1 (ppm)

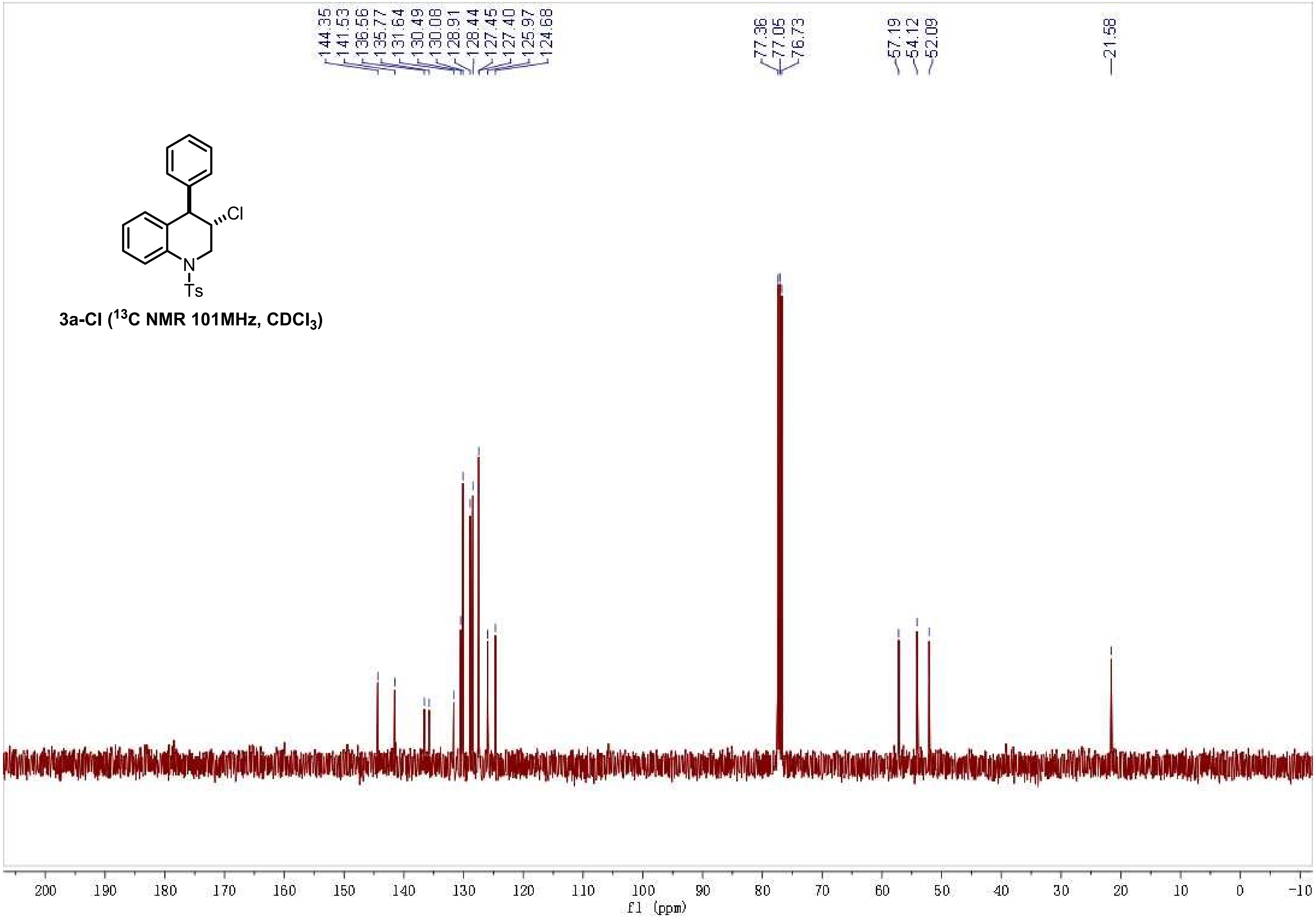

3a-Cl (^{13}C NMR 101MHz, $CDCl_3$)

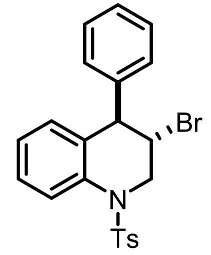

3a-Br (^{1}H NMR 400MHz, $CDCl_3$)

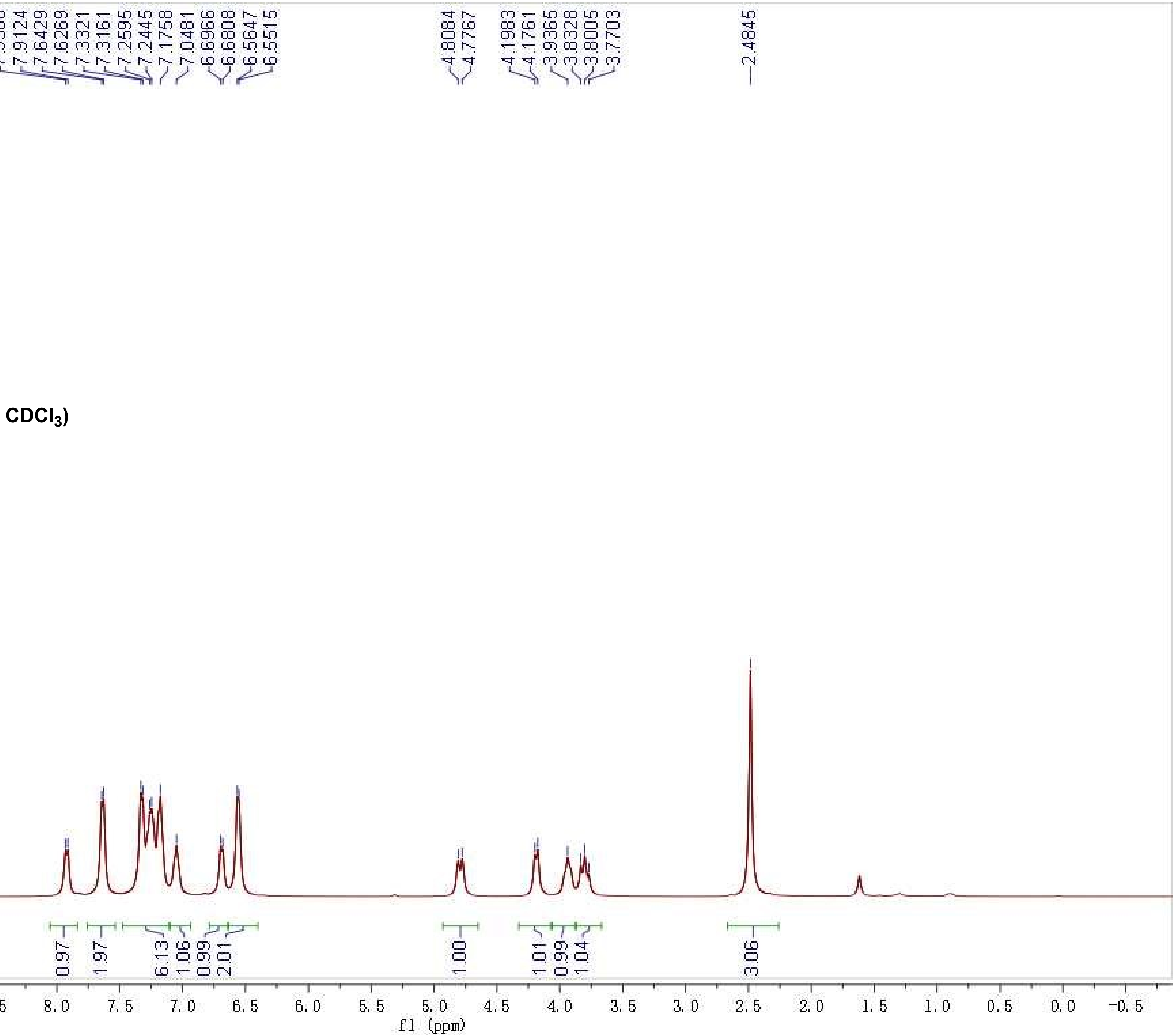

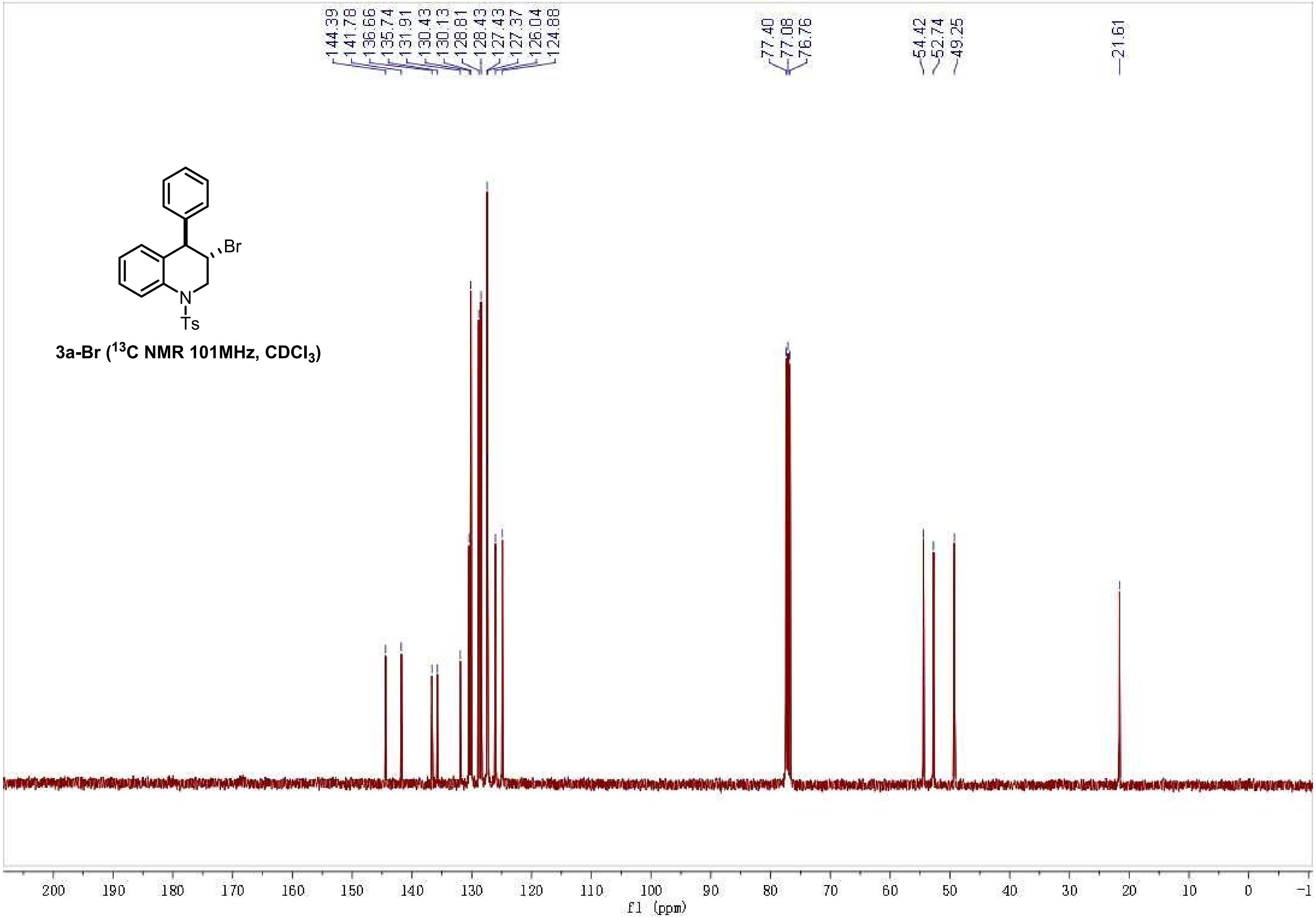

3a-Br (^{13}C NMR 101MHz, $CDCl_3$)

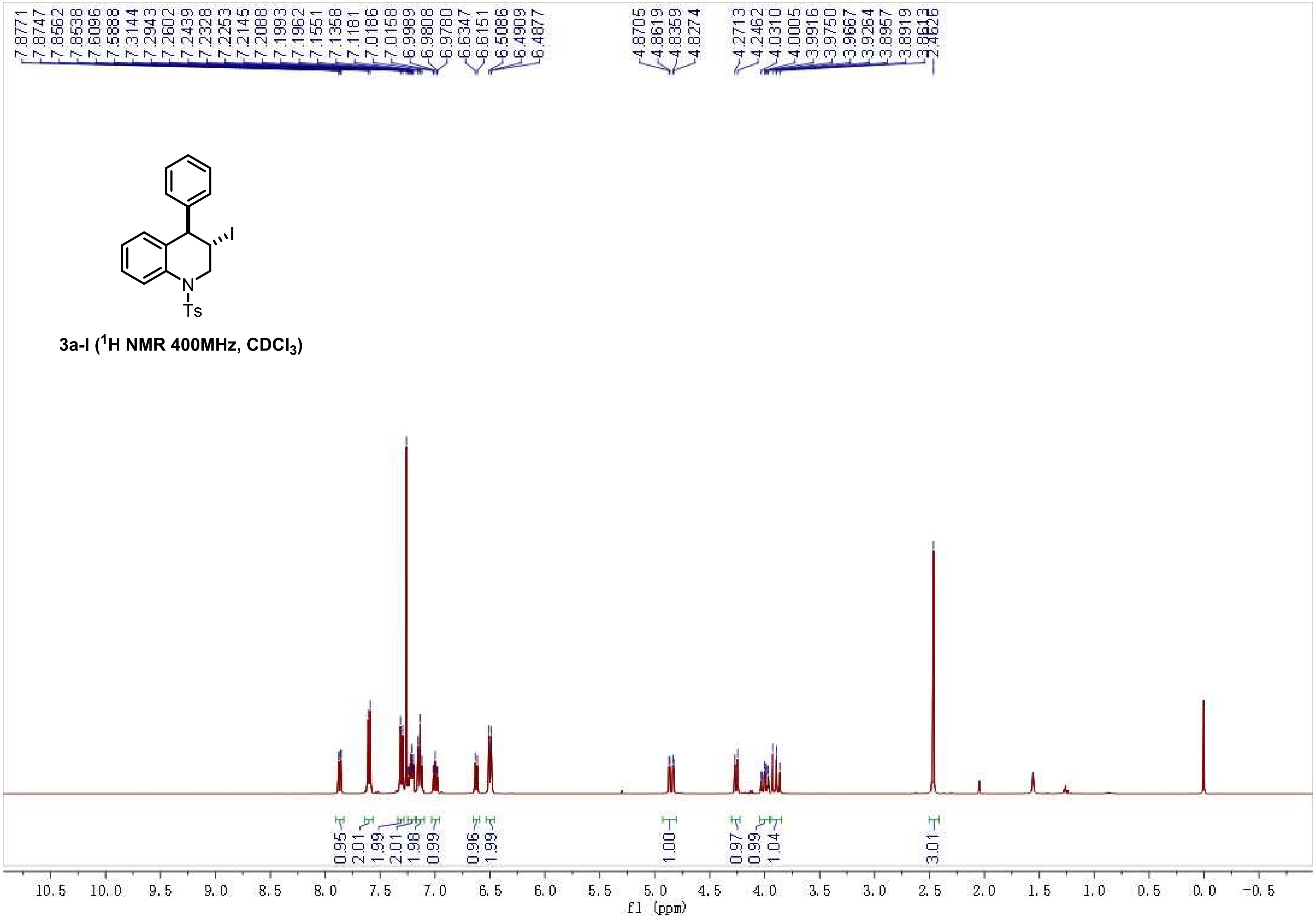

3a-I (^{1}H NMR 400MHz, $CDCl_3$)

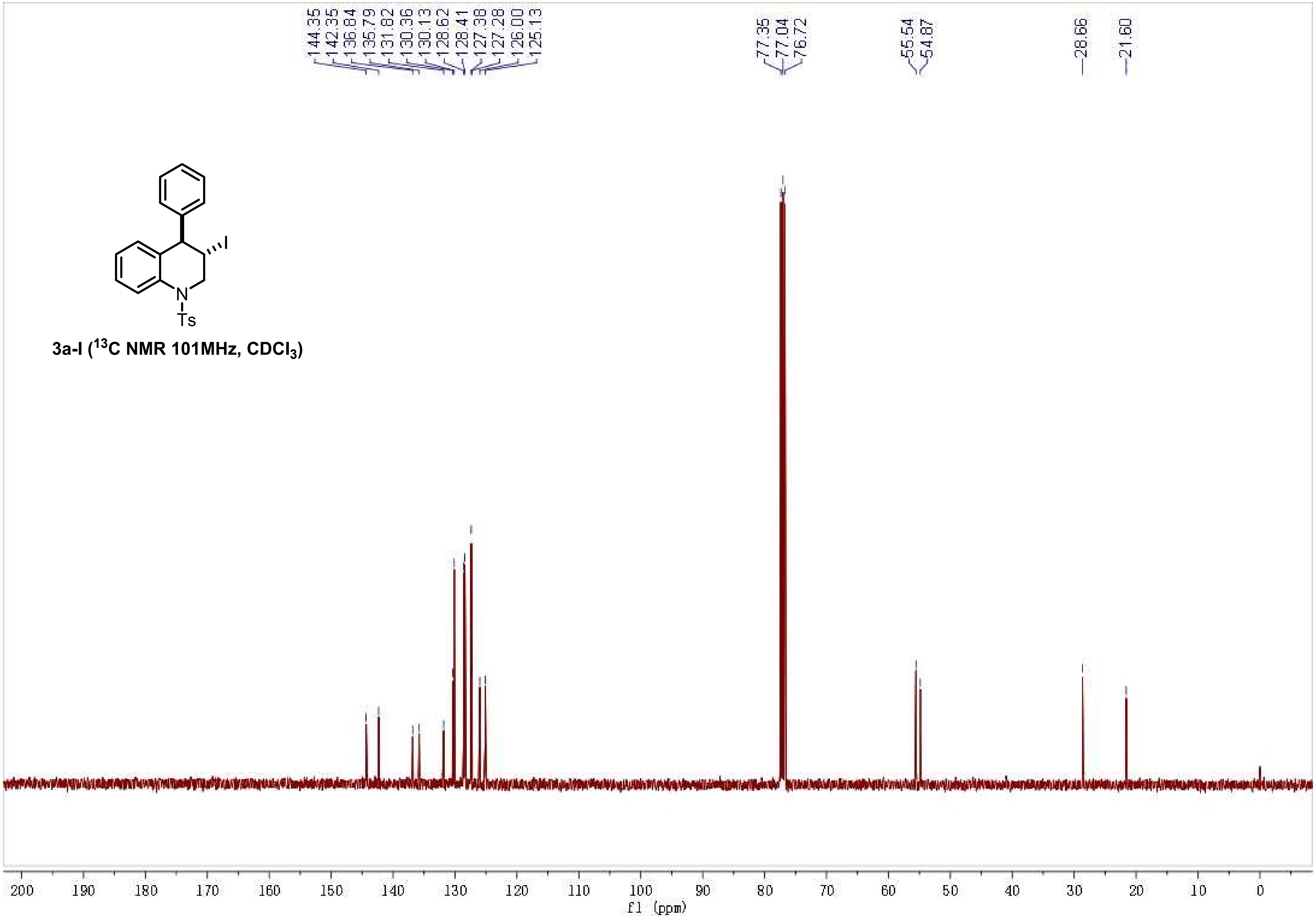

3a-I (^{13}C NMR 101MHz, CDCl$_3$)

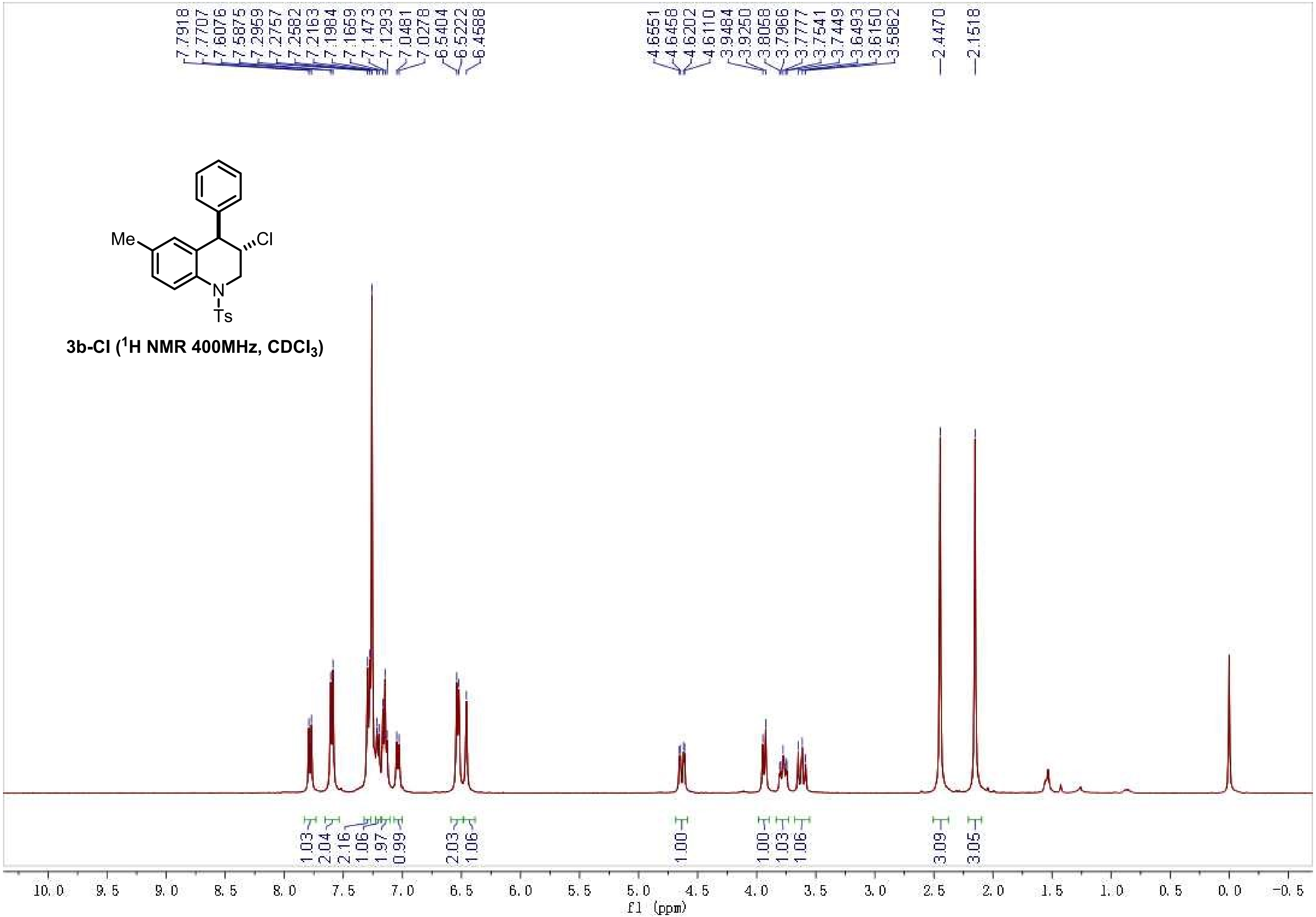
7.7918
7.7707
7.6076
7.5875
7.2959
7.2757
7.2582
7.2163
7.1984
7.1659
7.1473
7.1293
7.0481
7.0278
6.5404
6.5222
6.4588
4.6551
4.6458
4.6202
4.6110
3.9484
3.9250
3.8058
3.7966
3.7777
3.7541
3.7449
3.6493
3.6150
3.5862
2.4470
2.1518
Me
Cl
N
Ts
3b-Cl (1H NMR 400MHz, CDCl3)
1.03
2.04
2.16
1.06
1.97
0.99
2.03
1.06
1.00
1.00
1.03
1.06
3.09
3.05
10.0
9.5
9.0
8.5
8.0
7.5
7.0
6.5
6.0
5.5
5.0
4.5
4.0
3.5
3.0
2.5
2.0
1.5
1.0
0.5
0.0
-0.5
f1 (ppm)

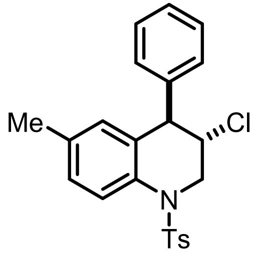

3b-Cl (^{13}C NMR 101MHz, $CDCl_3$)

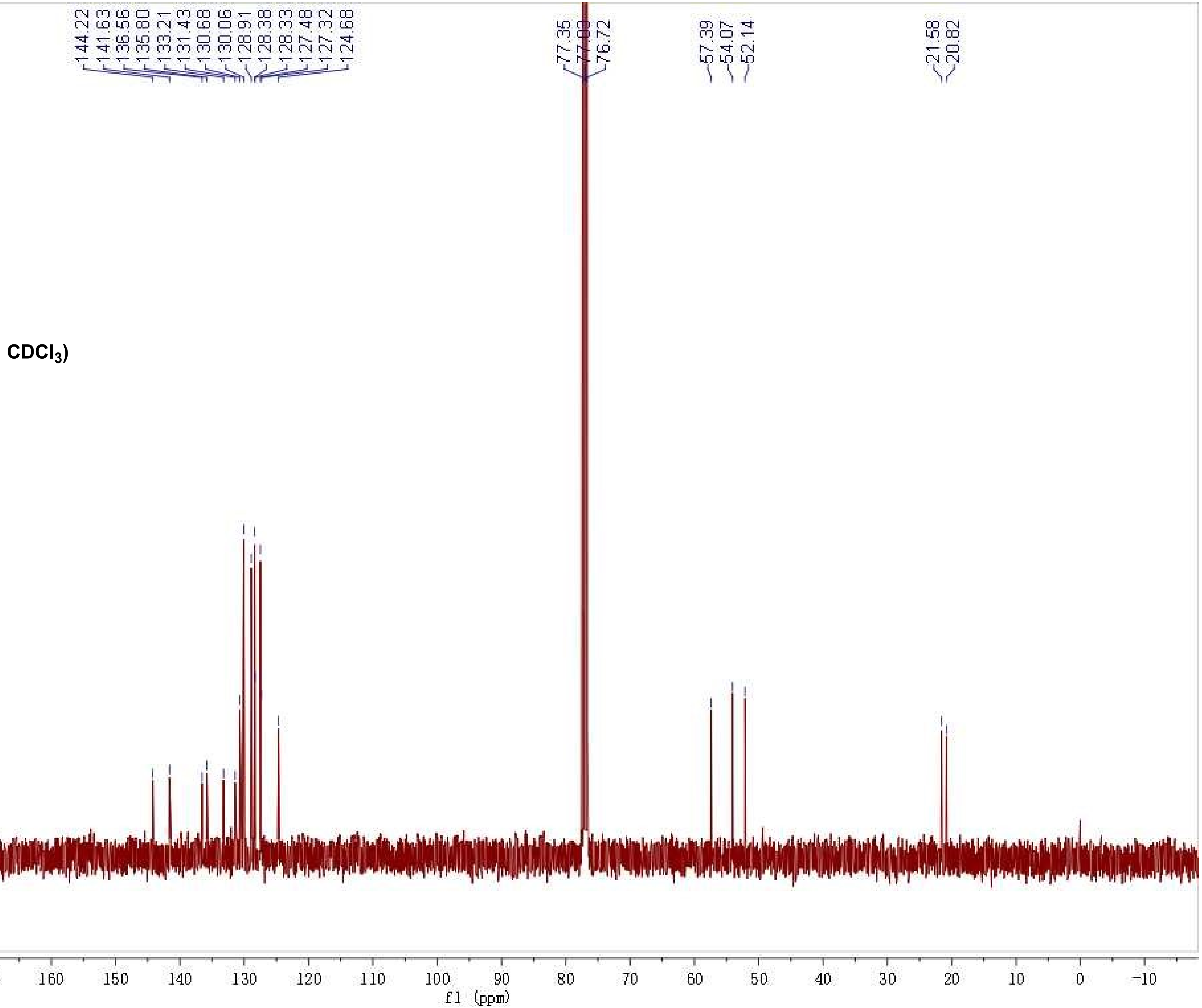

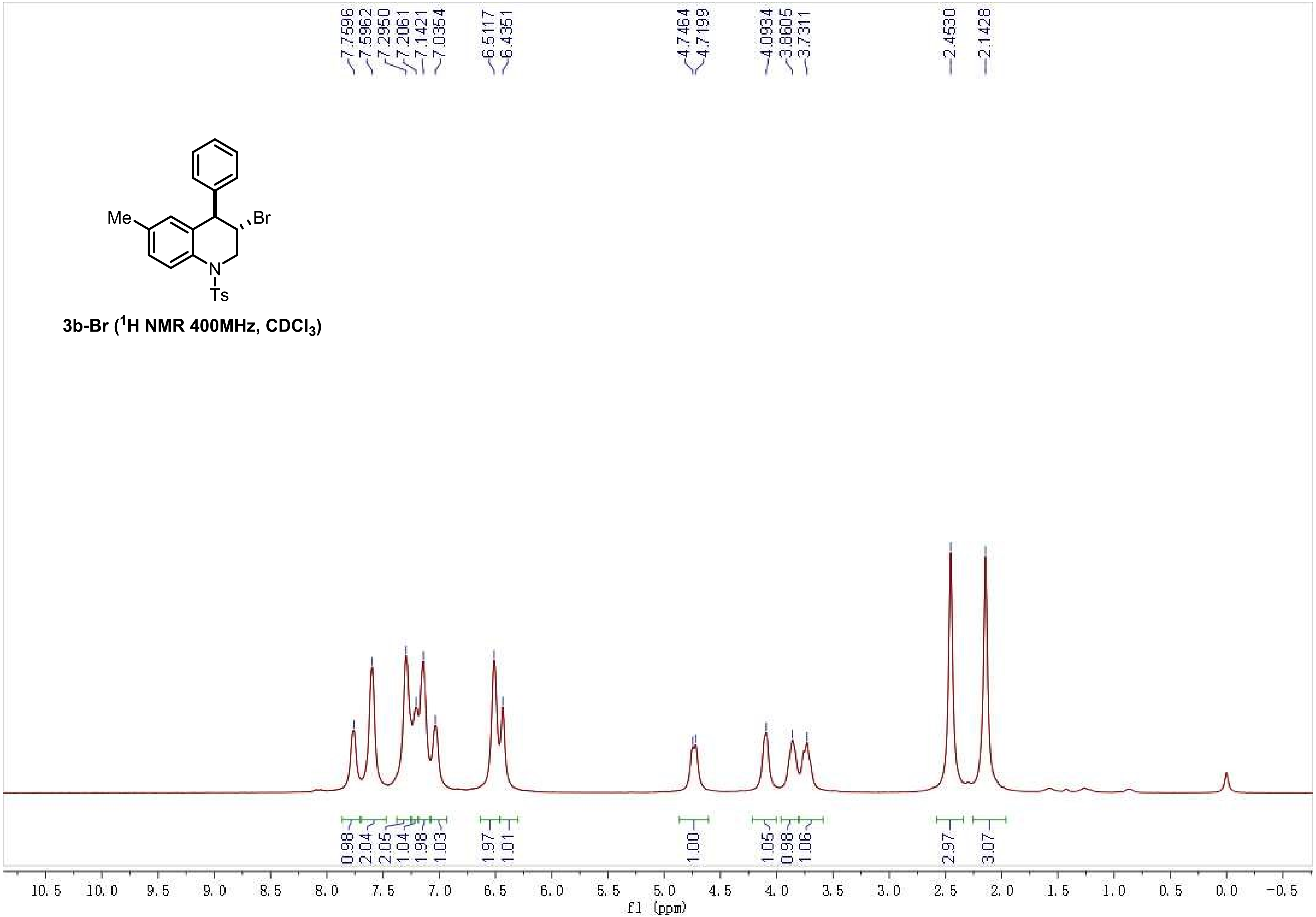

3b-Br (^{1}H NMR 400MHz, $CDCl_3$)

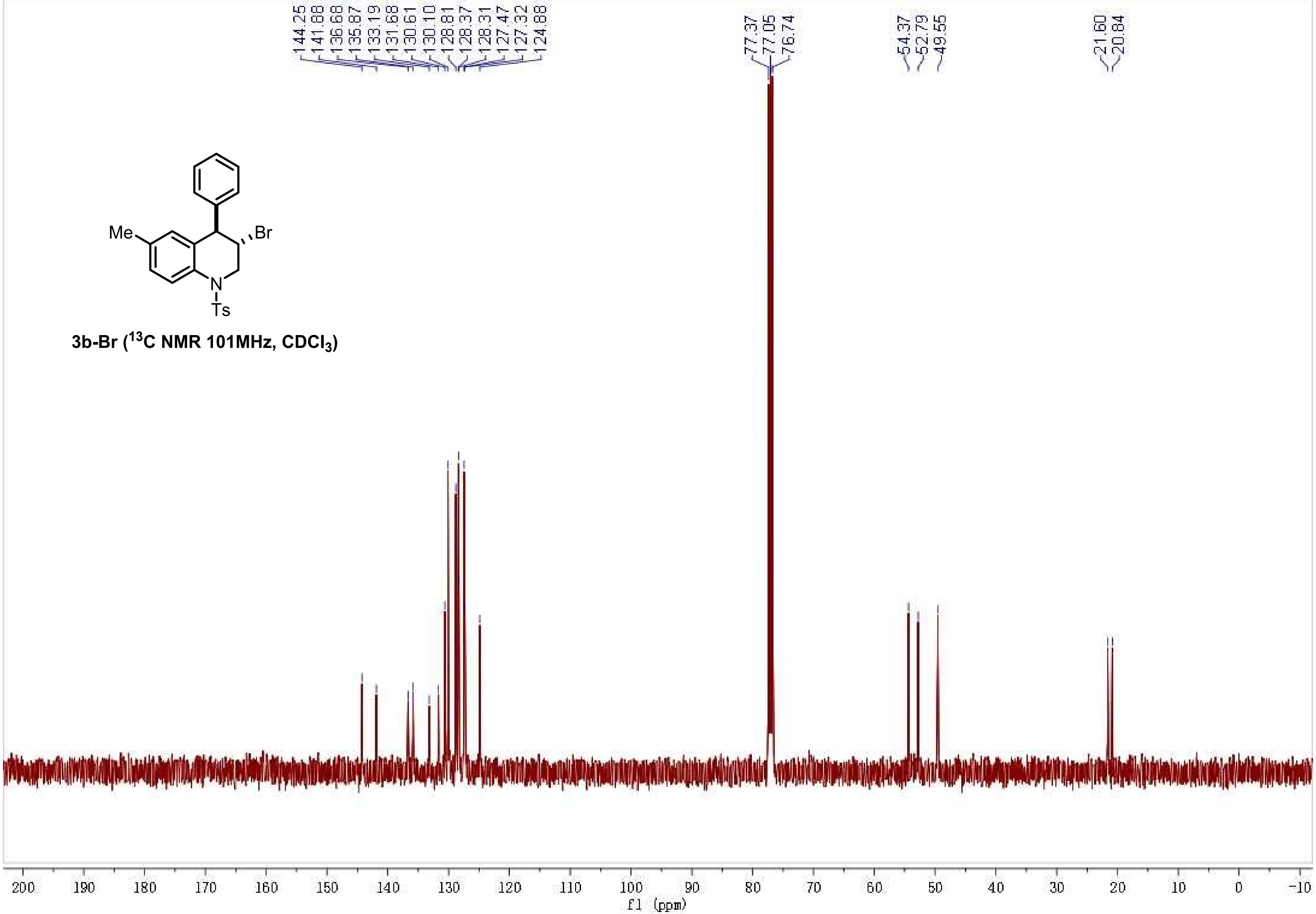

3b-Br (^{13}C NMR 101MHz, CDCl$_3$)

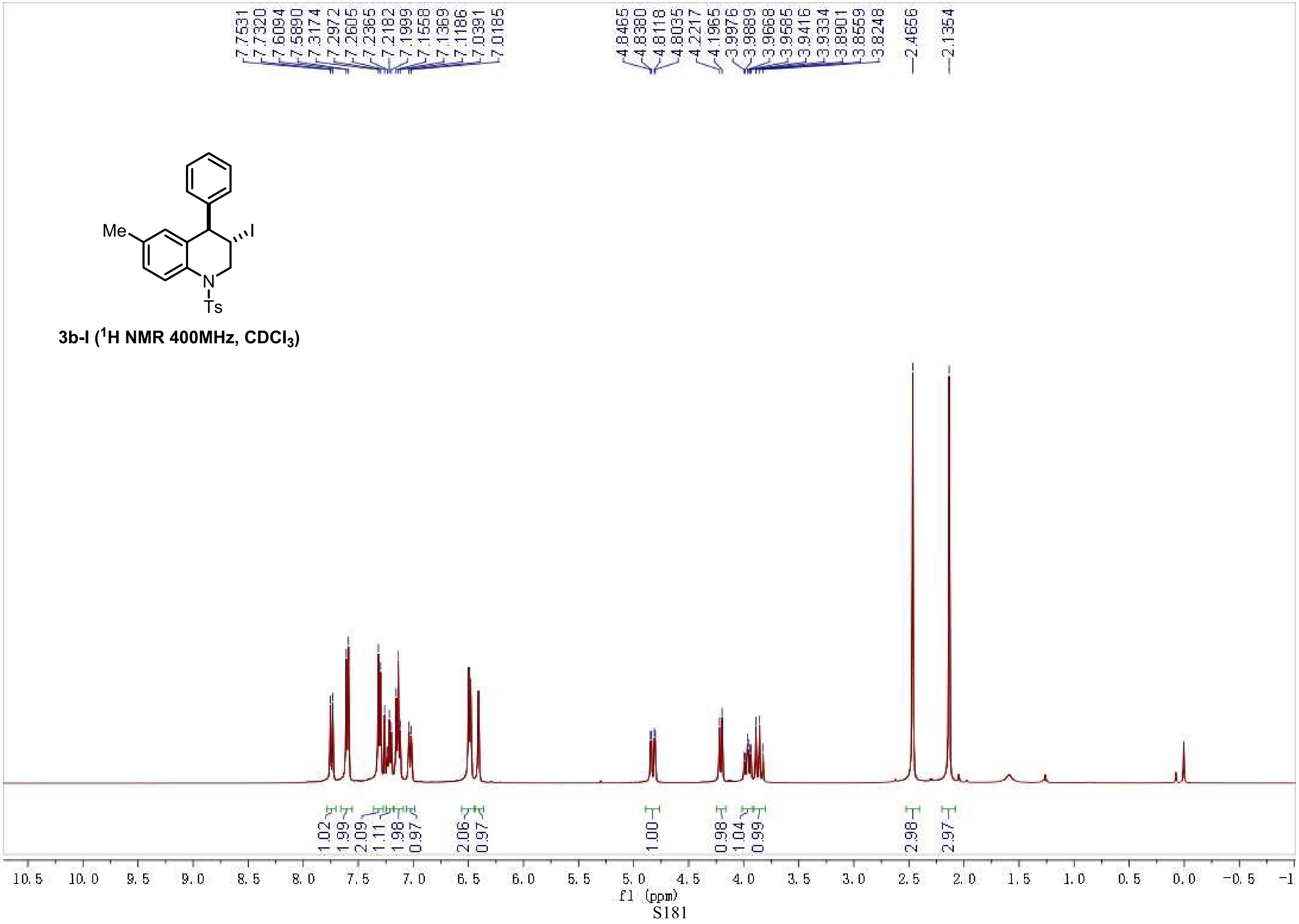

3b-I (^{1}H NMR 400MHz, $CDCl_3$)

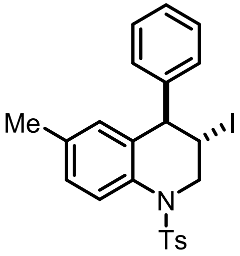

3b-I (^{13}C NMR 101MHz, $CDCl_3$)

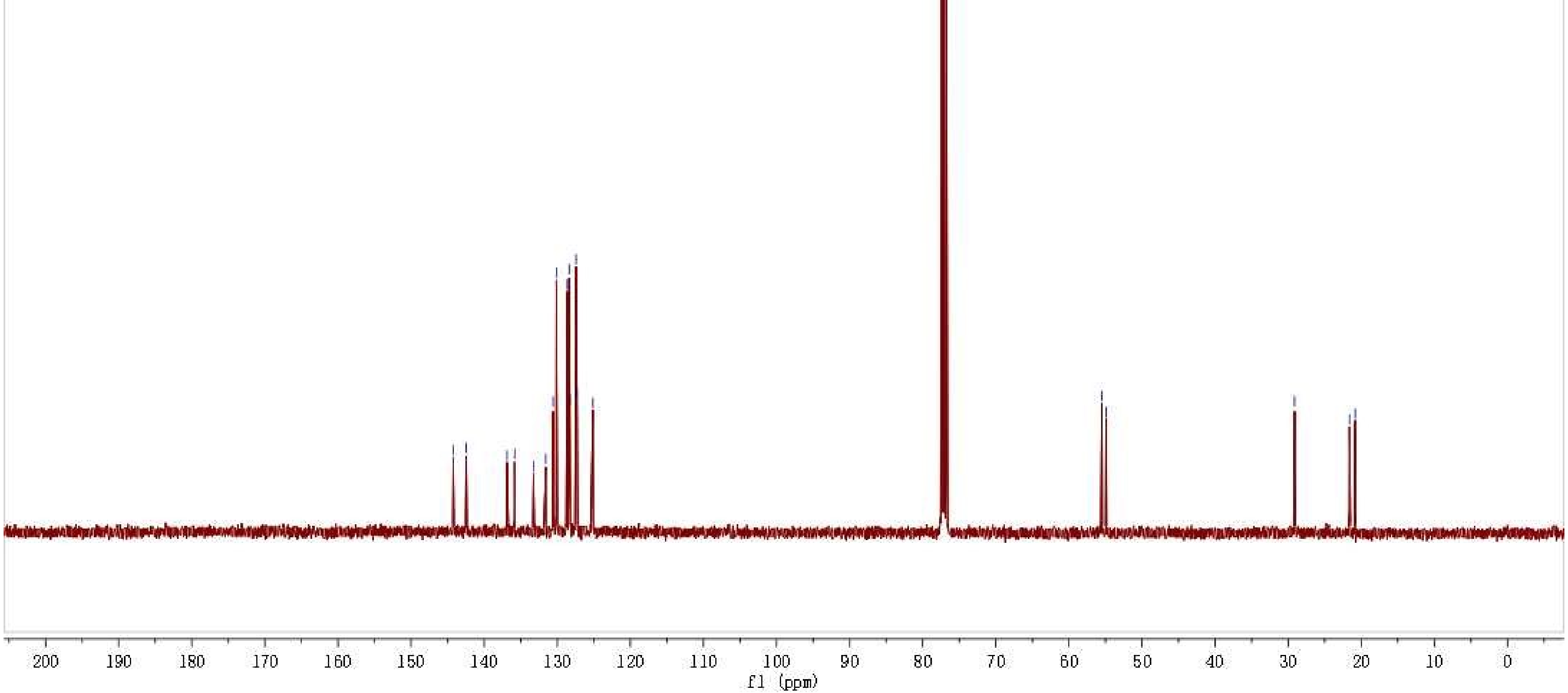

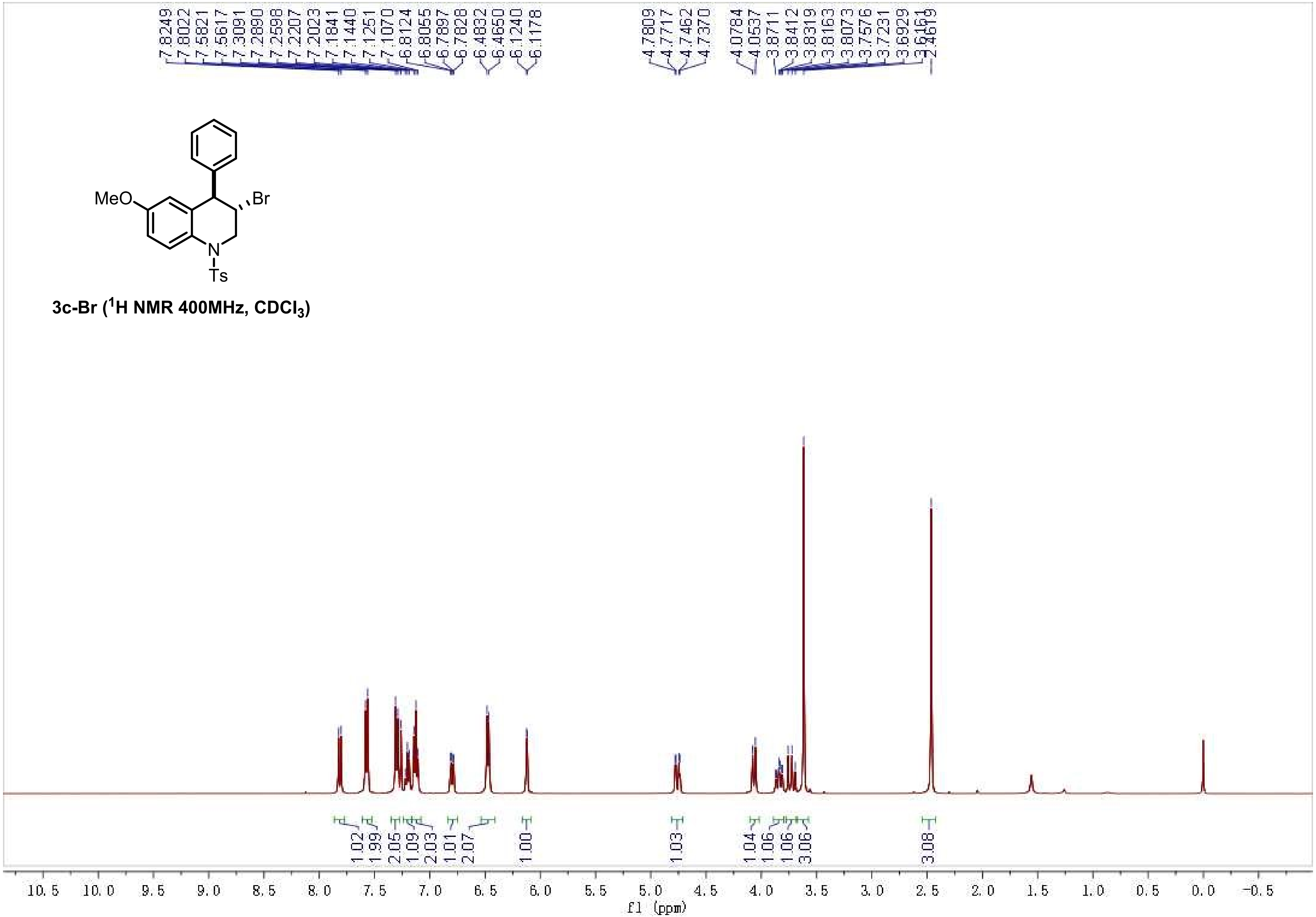

3c-Br (^{1}H NMR 400MHz, $CDCl_3$)

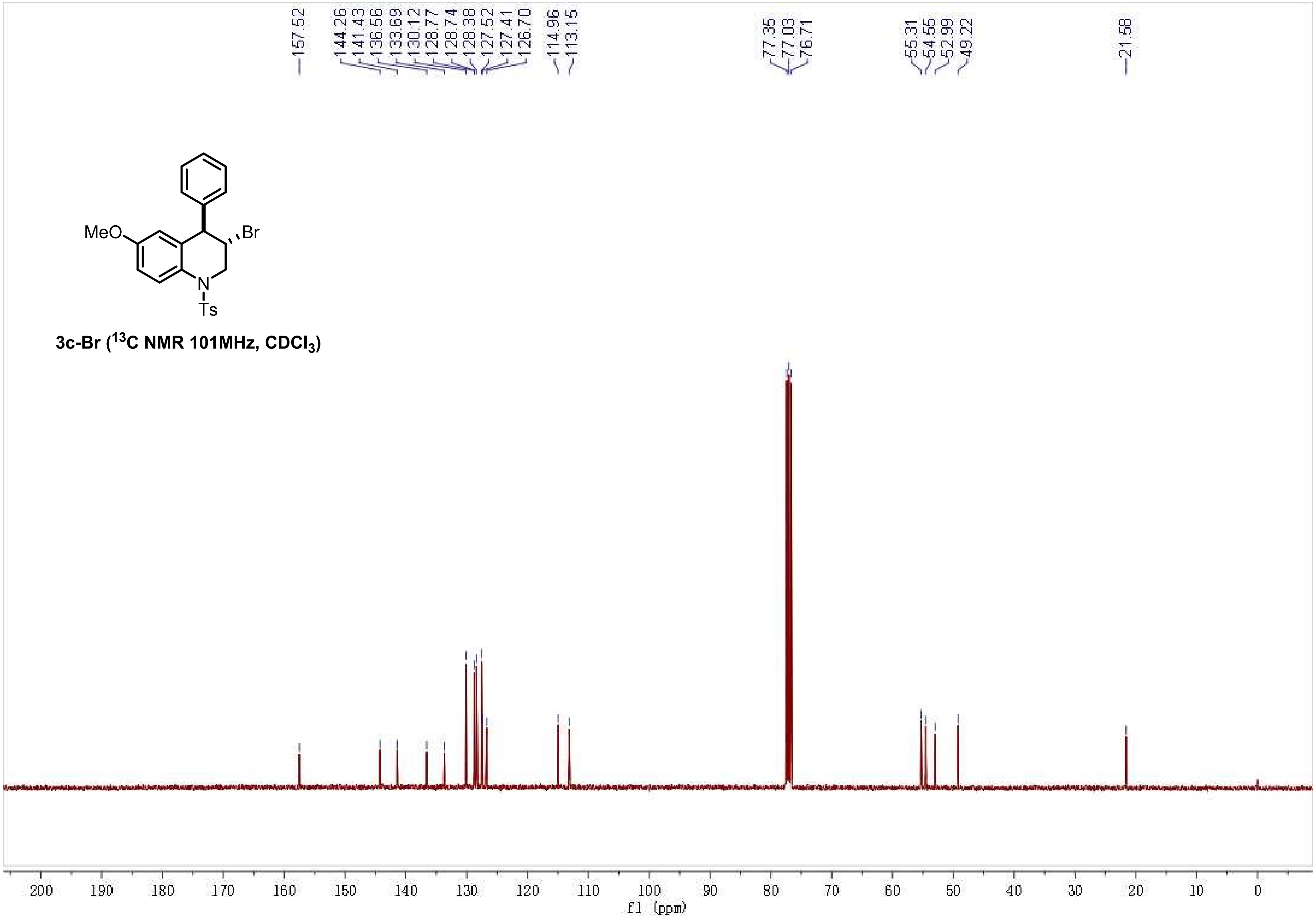
157.52
144.26
141.43
136.56
133.69
130.12
128.77
128.74
128.38
127.52
127.41
126.70
114.96
113.15
77.35
77.03
76.71
55.31
54.55
52.99
49.22
21.58
MeO
Br
N
Ts
3c-Br (^{13}C NMR 101MHz, $CDCl_3$)
200 190 180 170 160 150 140 130 120 110 100 90 80 70 60 50 40 30 20 10 0
f1 (ppm)

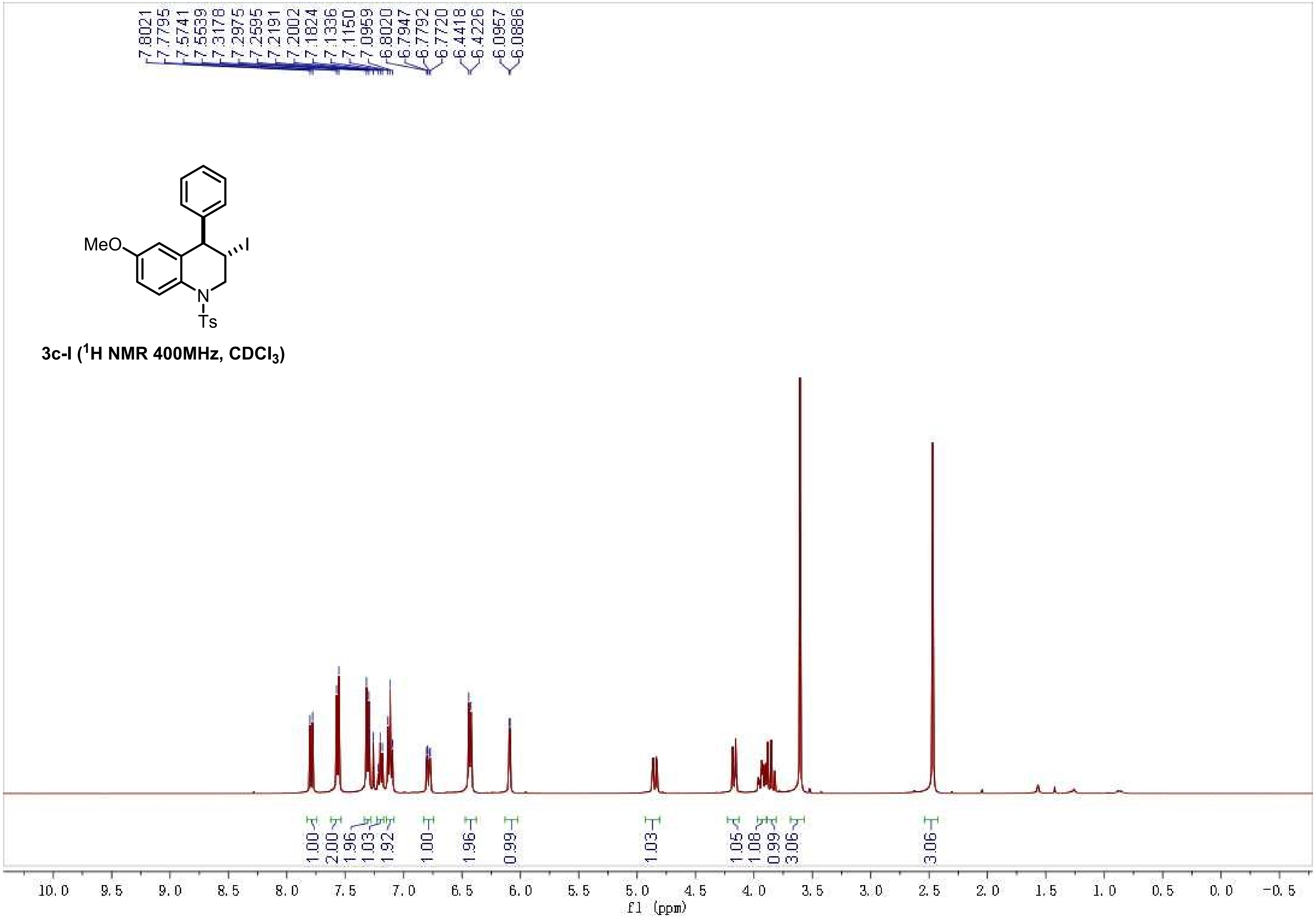

3c-I (^{1}H NMR 400MHz, $CDCl_3$)

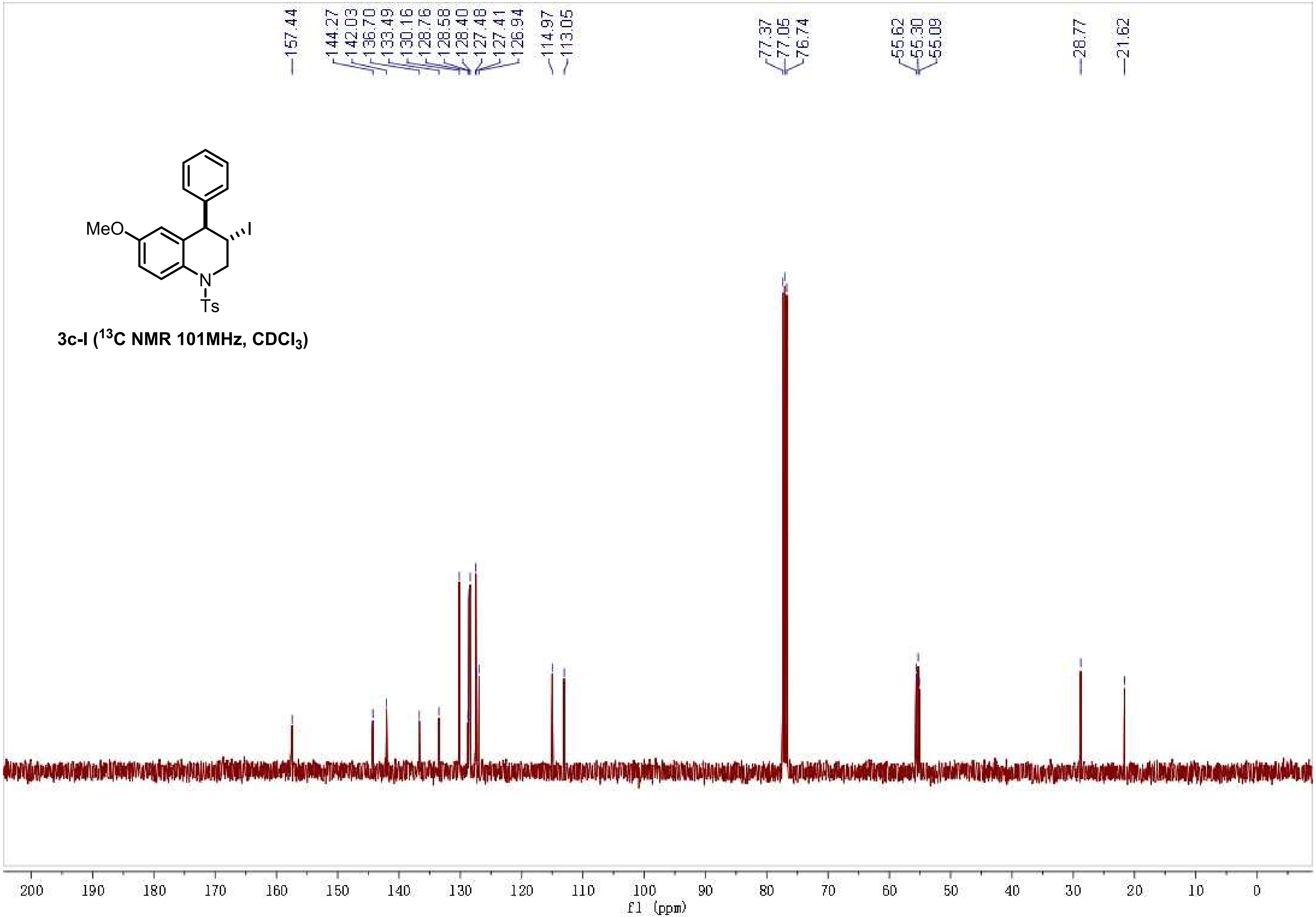

3c-I (^{13}C NMR 101MHz, $CDCl_3$)

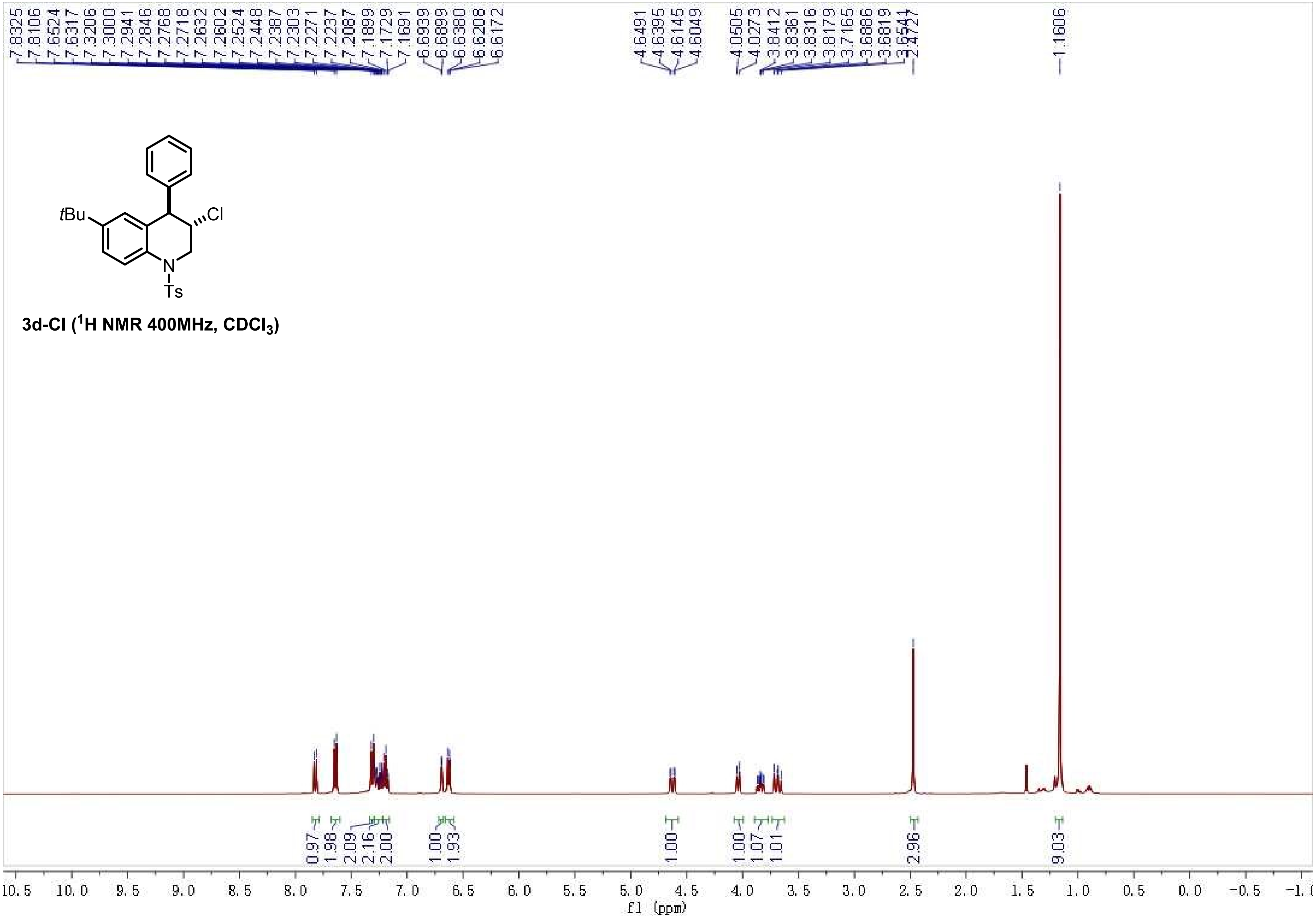
tBu
Cl
N
Ts
3d-Cl (1H NMR 400MHz, CDCl3)
7.8325
7.8106
7.6524
7.6317
7.3206
7.3000
7.2941
7.2846
7.2768
7.2718
7.2632
7.2602
7.2524
7.2448
7.2387
7.2303
7.2271
7.2237
7.2087
7.1899
7.1729
7.1691
6.6939
6.6899
6.6380
6.6208
6.6172
4.6491
4.6395
4.6145
4.6049
4.0505
4.0273
3.8412
3.8361
3.8316
3.8179
3.7165
3.6886
3.6819
3.6541
2.4727
1.1606
0.97
1.98
2.09
2.16
2.00
1.00
1.93
1.00
1.00
1.07
1.01
2.96
9.03
f1 (ppm)

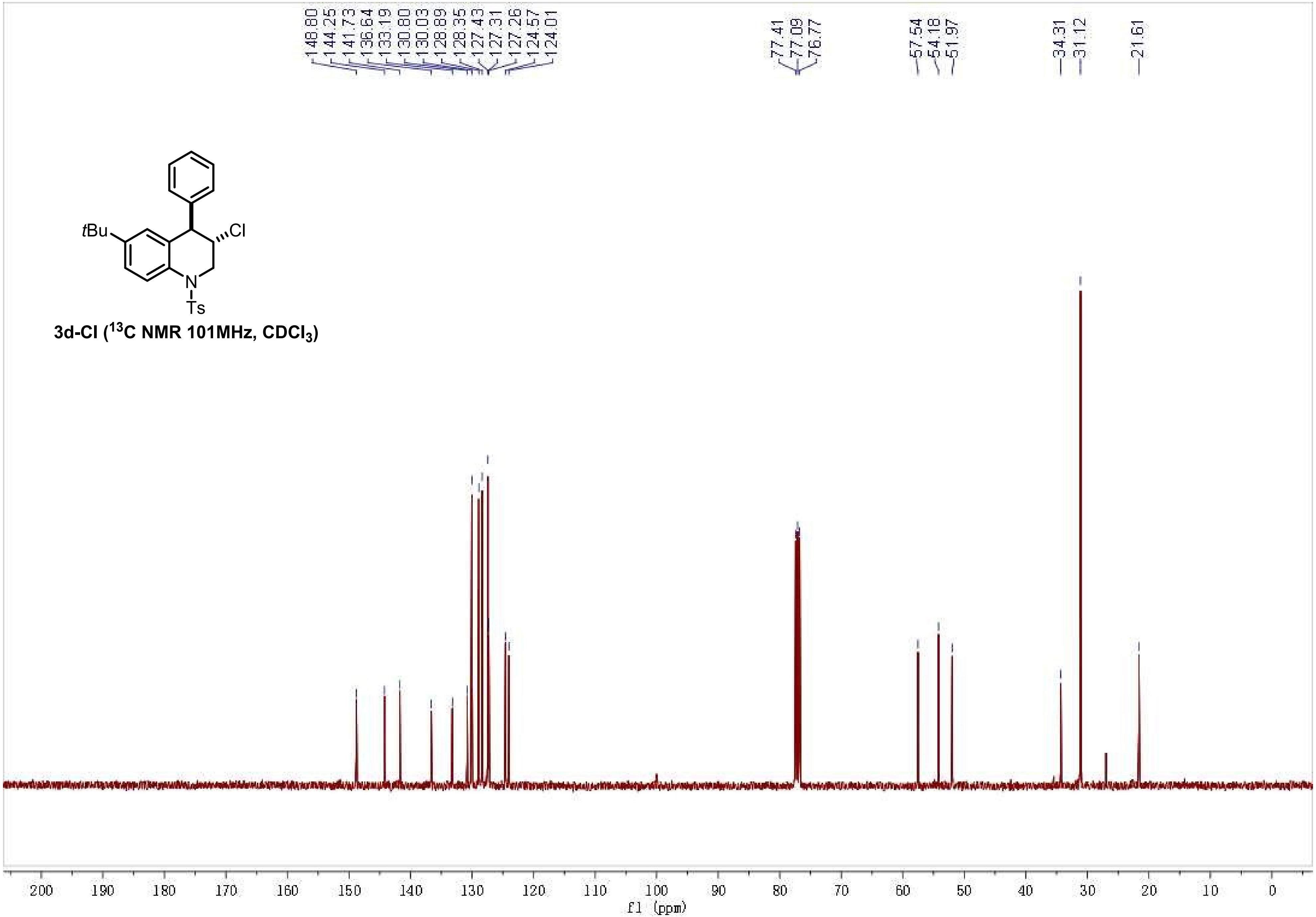

3d-Cl (^{13}C NMR 101MHz, $CDCl_3$)

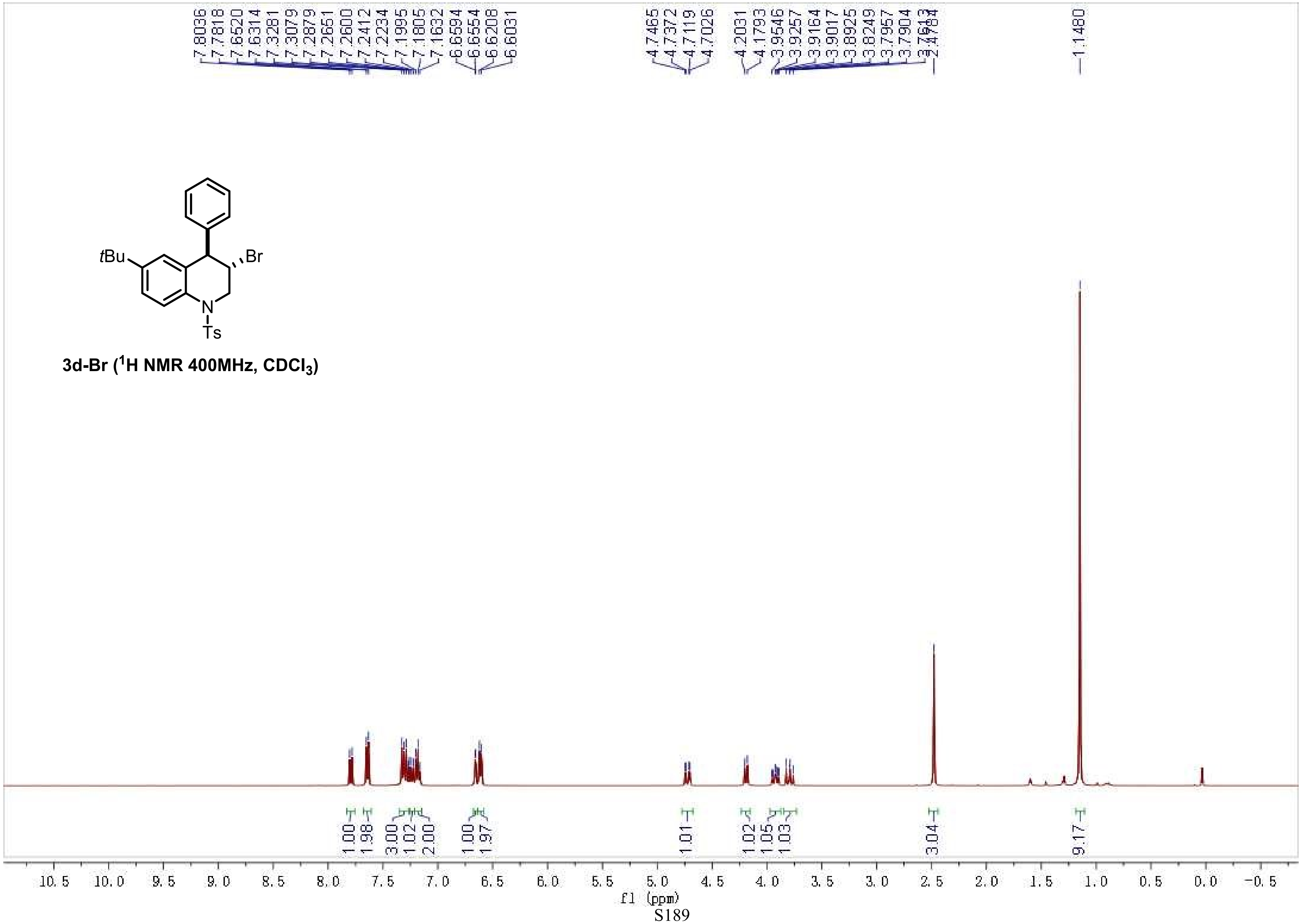

3d-Br (^{1}H NMR 400MHz, $CDCl_3$)

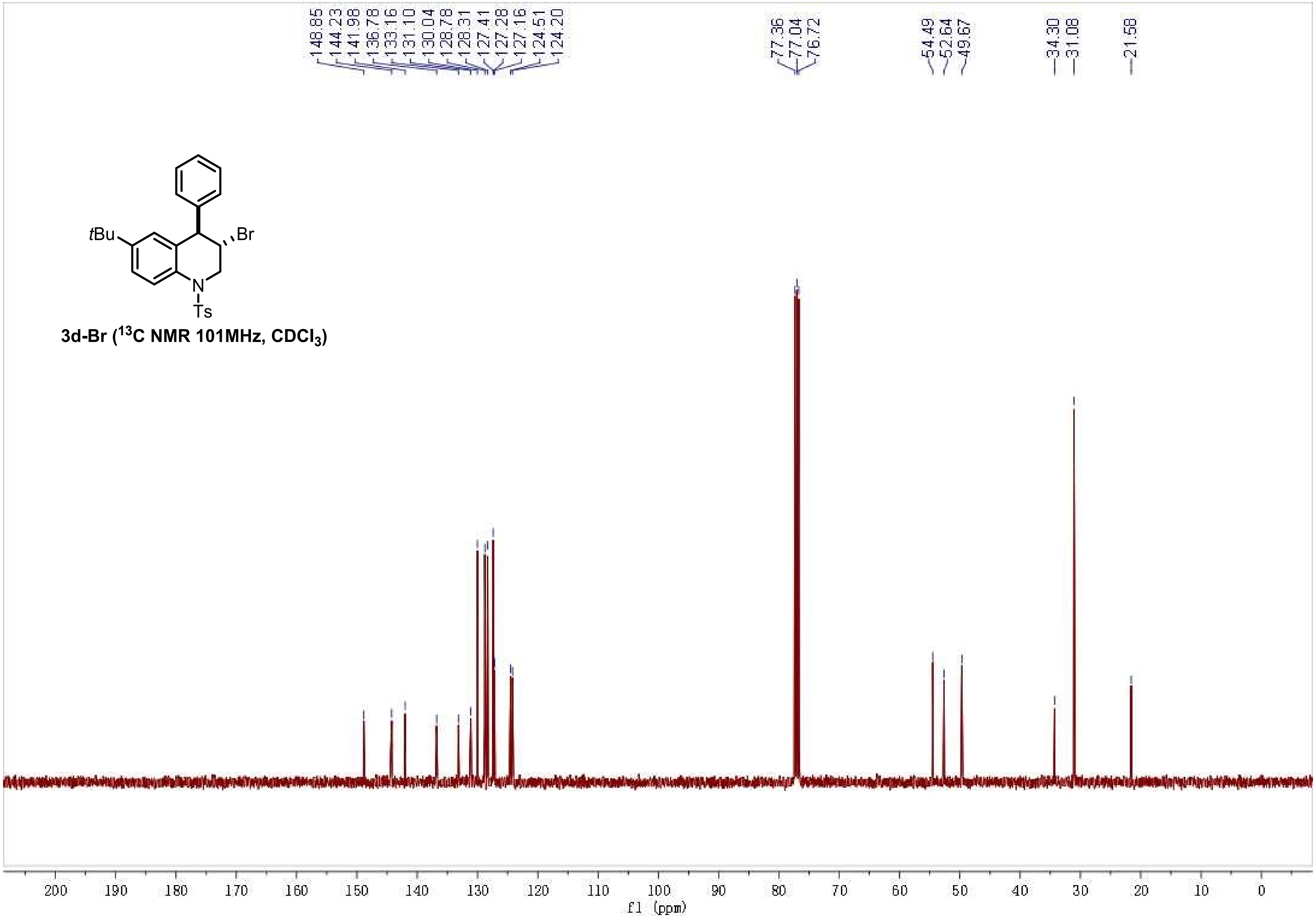

3d-Br (^{13}C NMR 101MHz, $CDCl_3$)

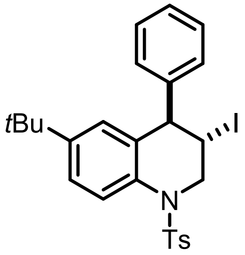

3d-I (^{1}H NMR 400MHz, $CDCl_3$)

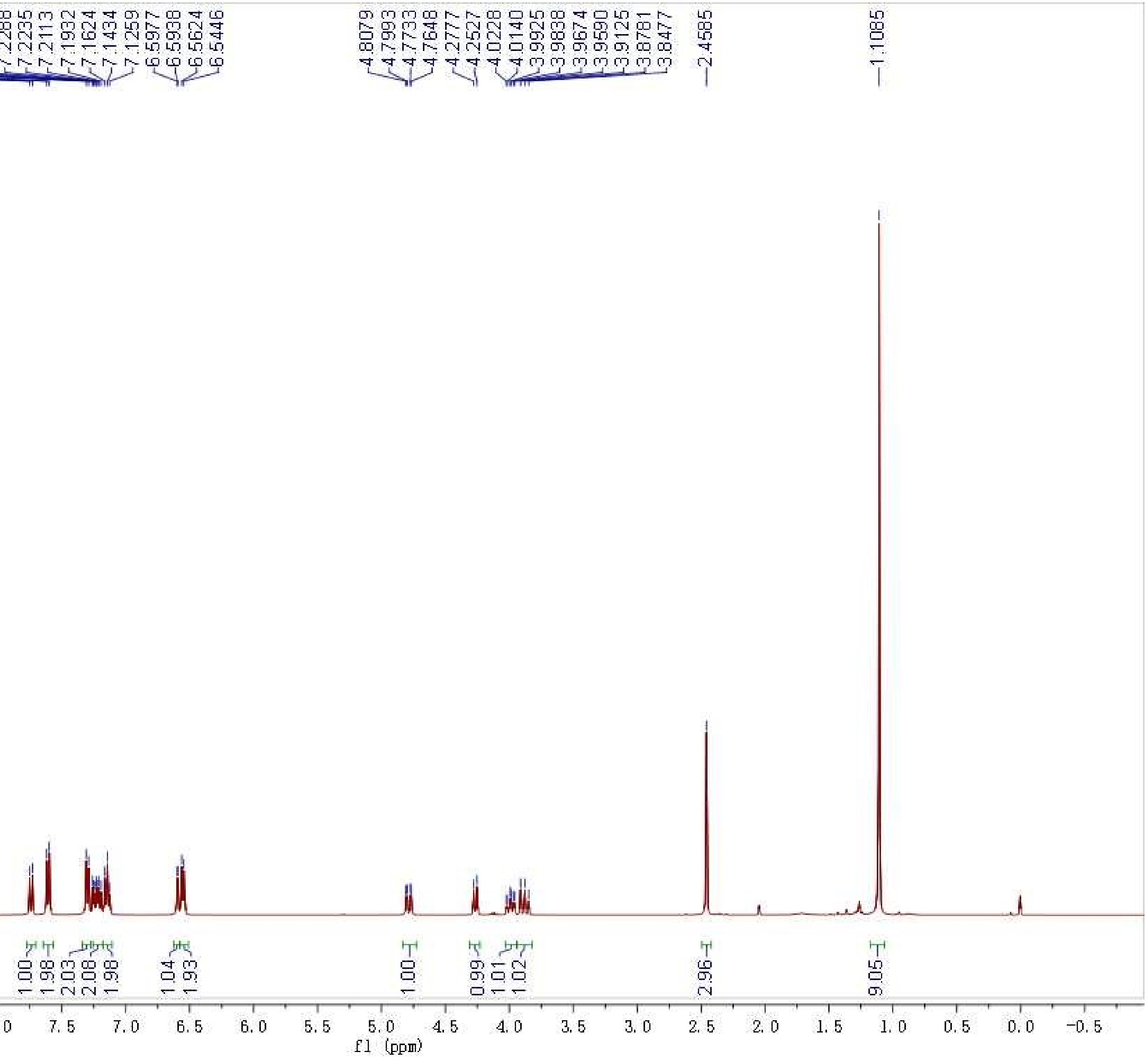

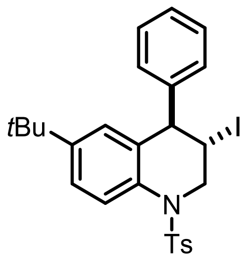

3d-I (^{13}C NMR 101MHz, $CDCl_3$)

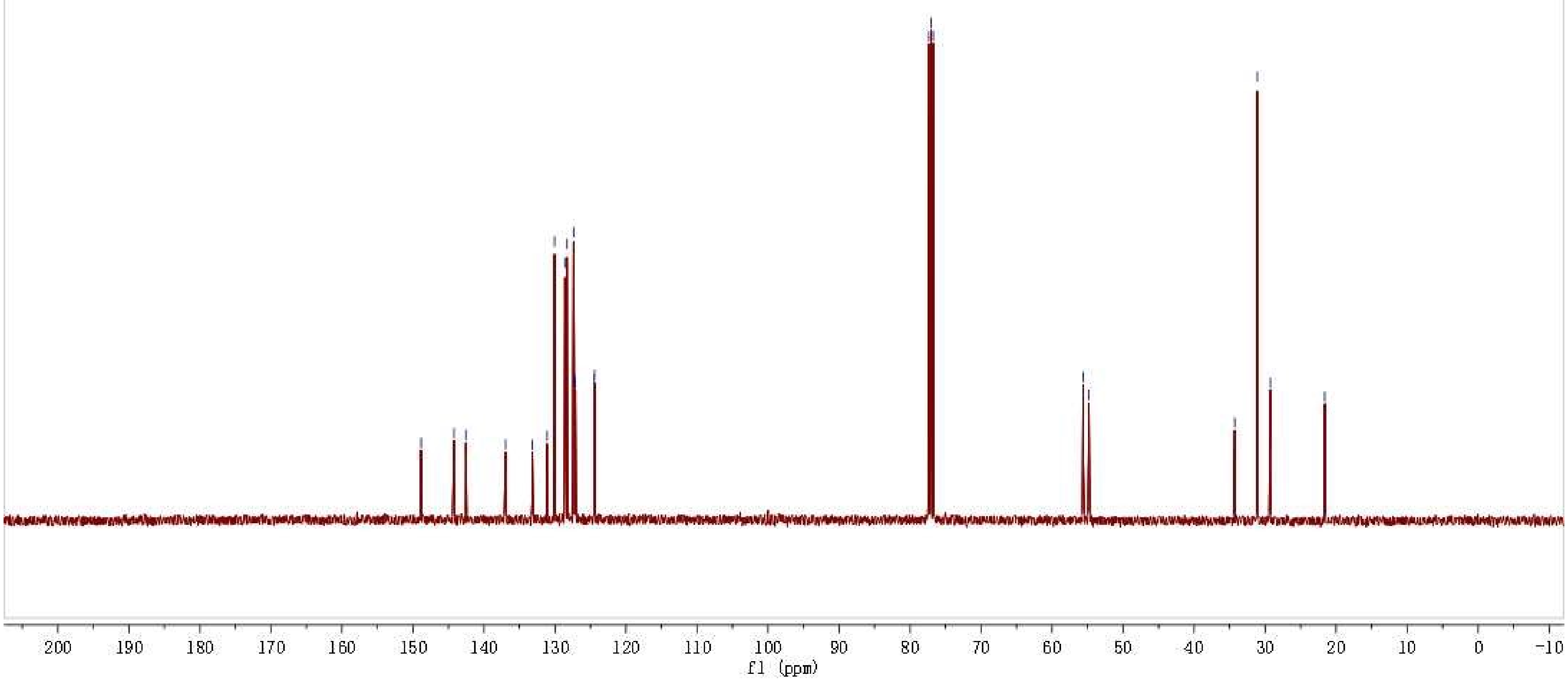

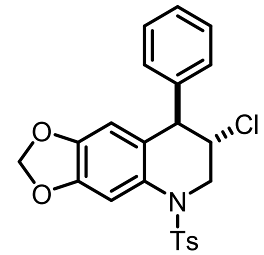

3e-Cl (^{1}H NMR 400MHz, $CDCl_3$)

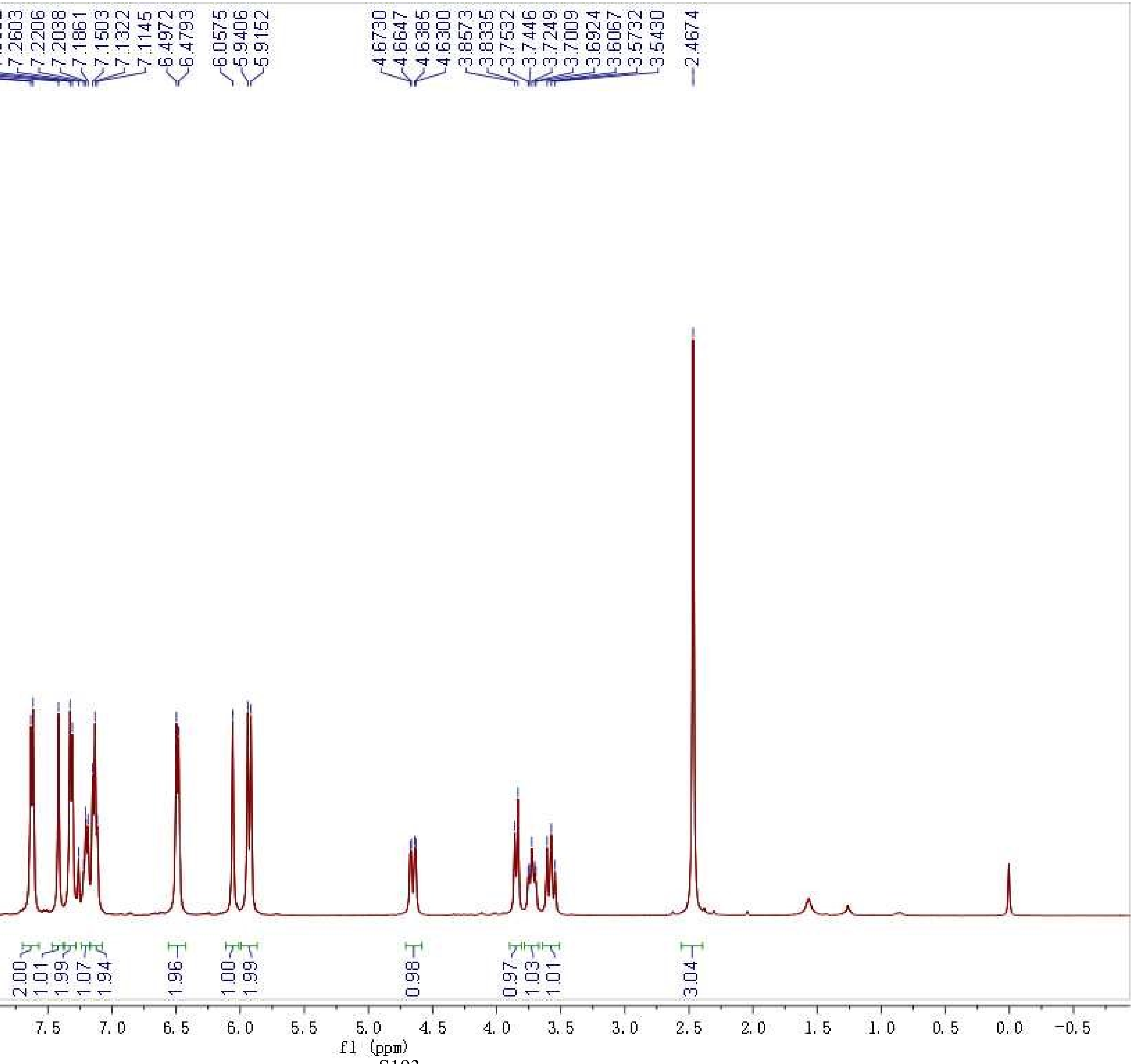

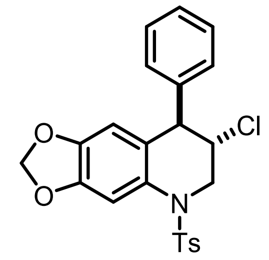

3e-Cl (^{13}C NMR 101MHz, $CDCl_3$)

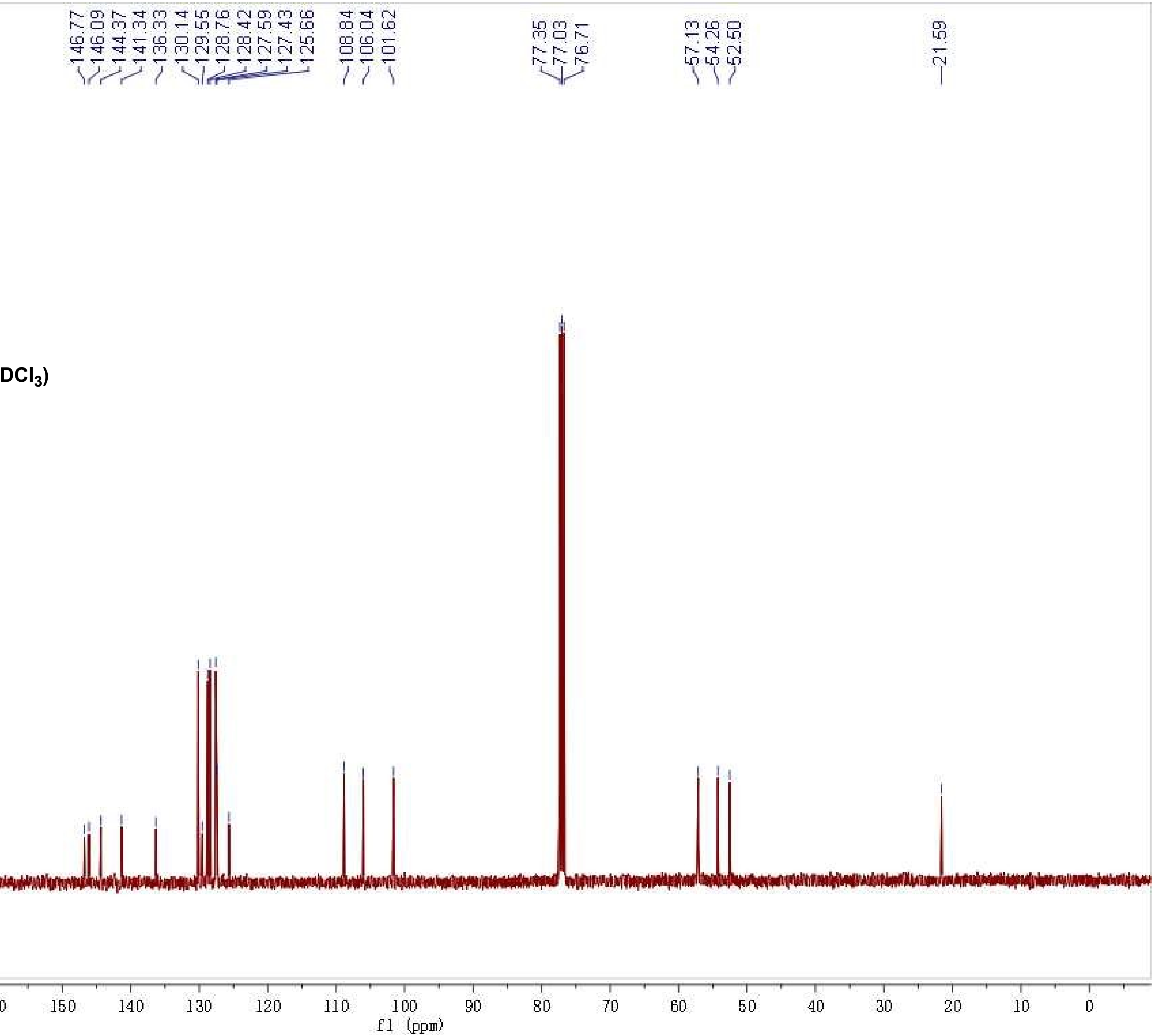

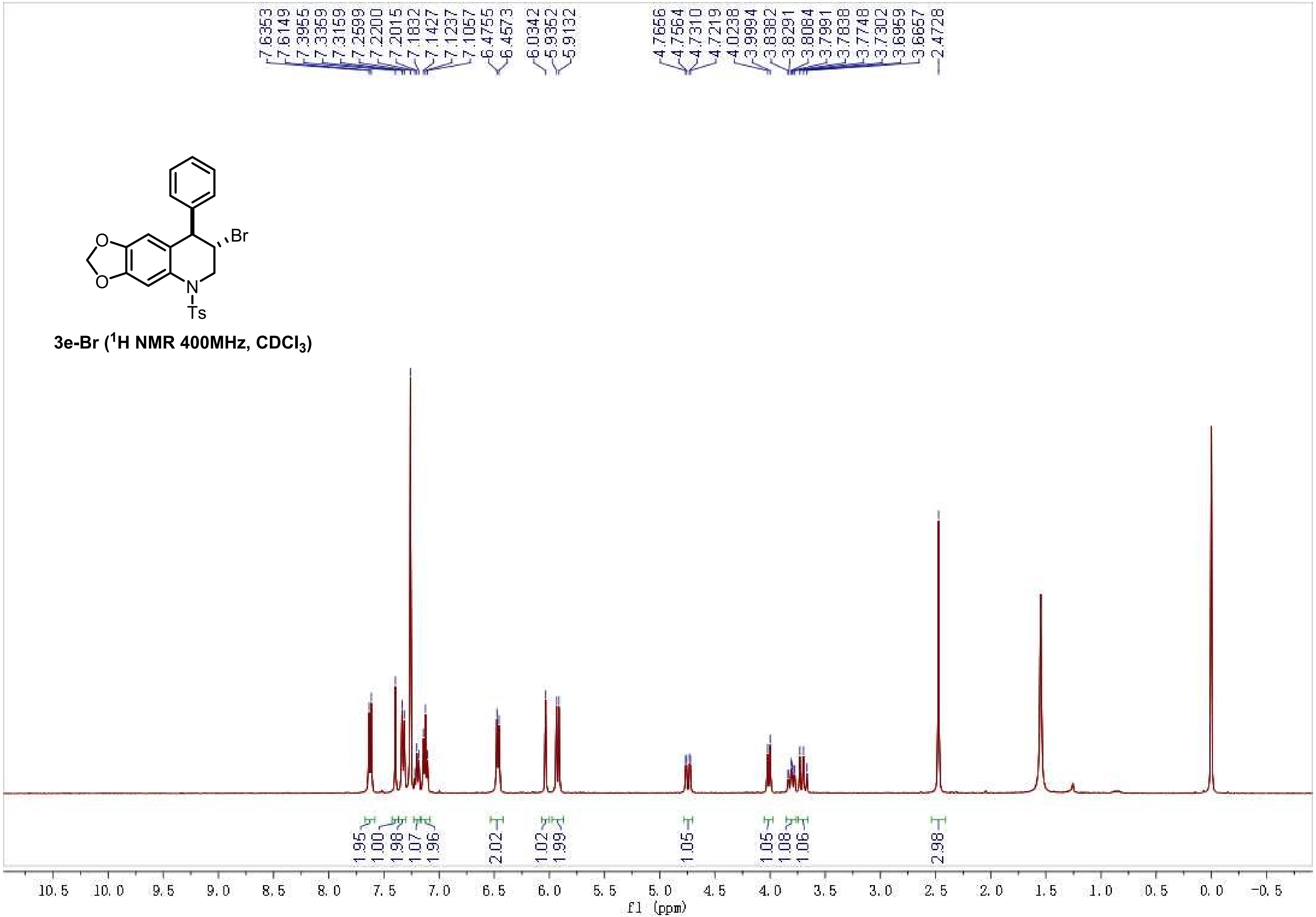

3e-Br (^{1}H NMR 400MHz, $CDCl_3$)

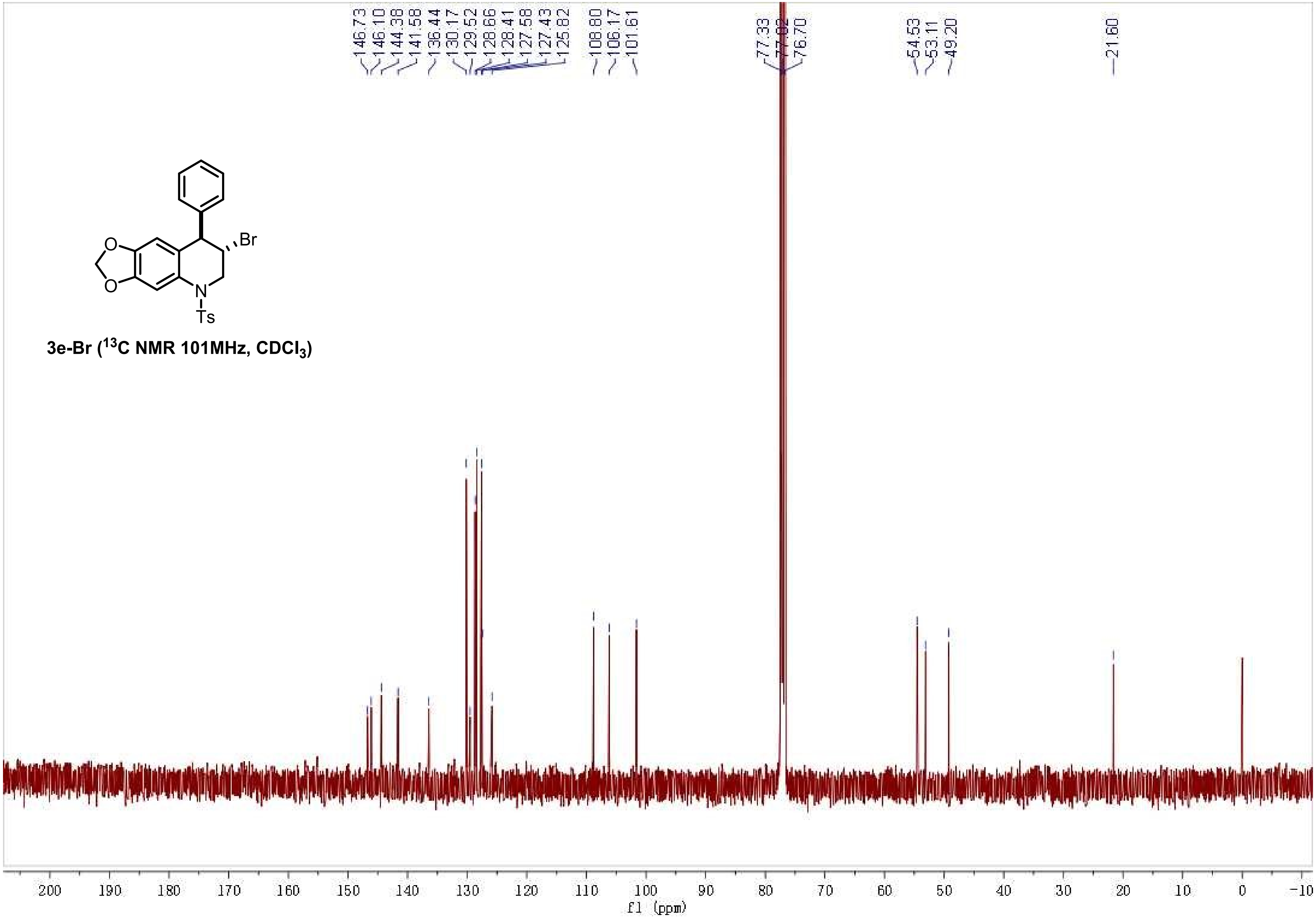

3e-Br (^{13}C NMR 101MHz, $CDCl_3$)

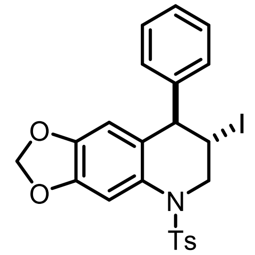

3e-I (^{1}H NMR 400MHz, $CDCl_3$)

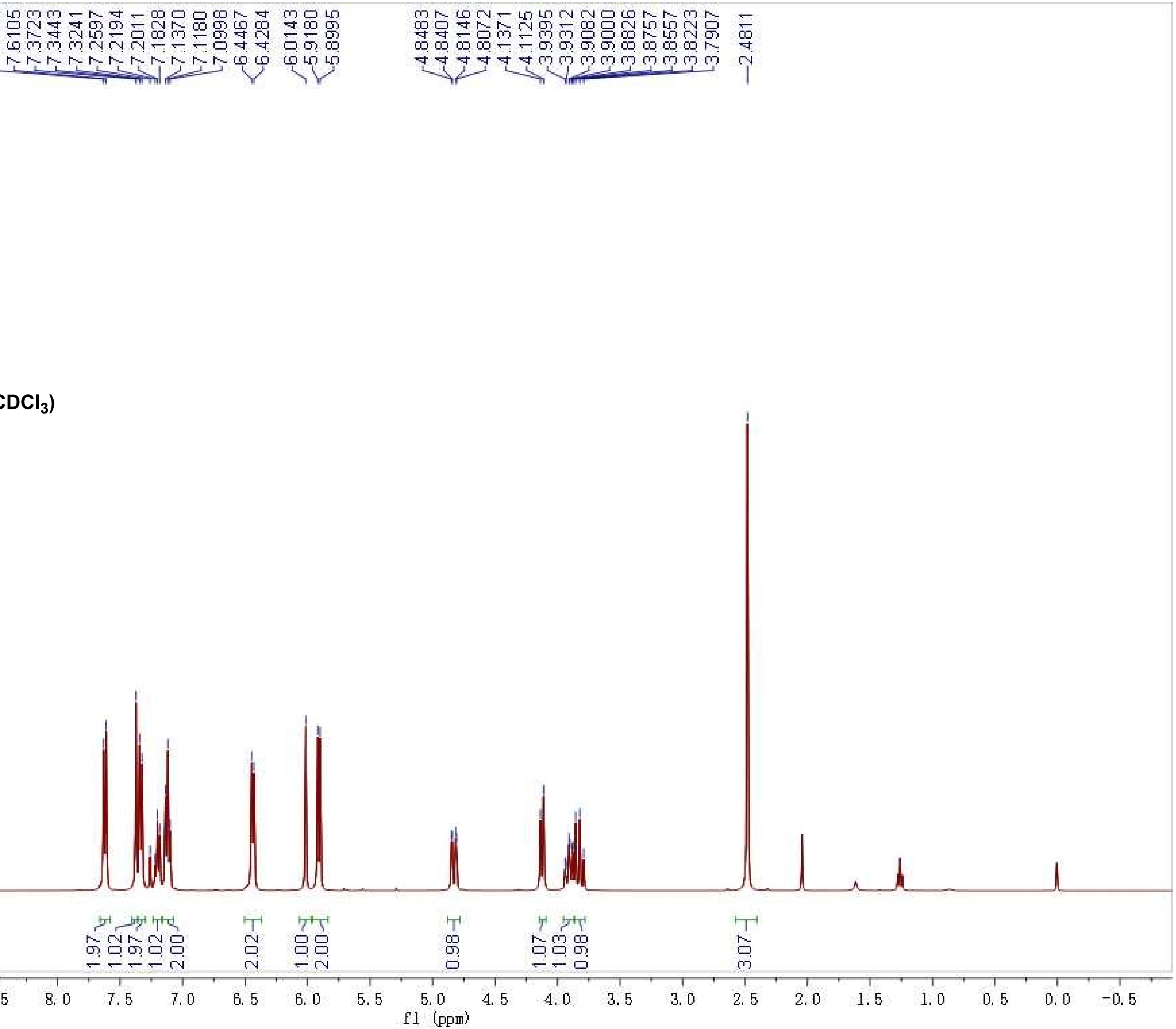

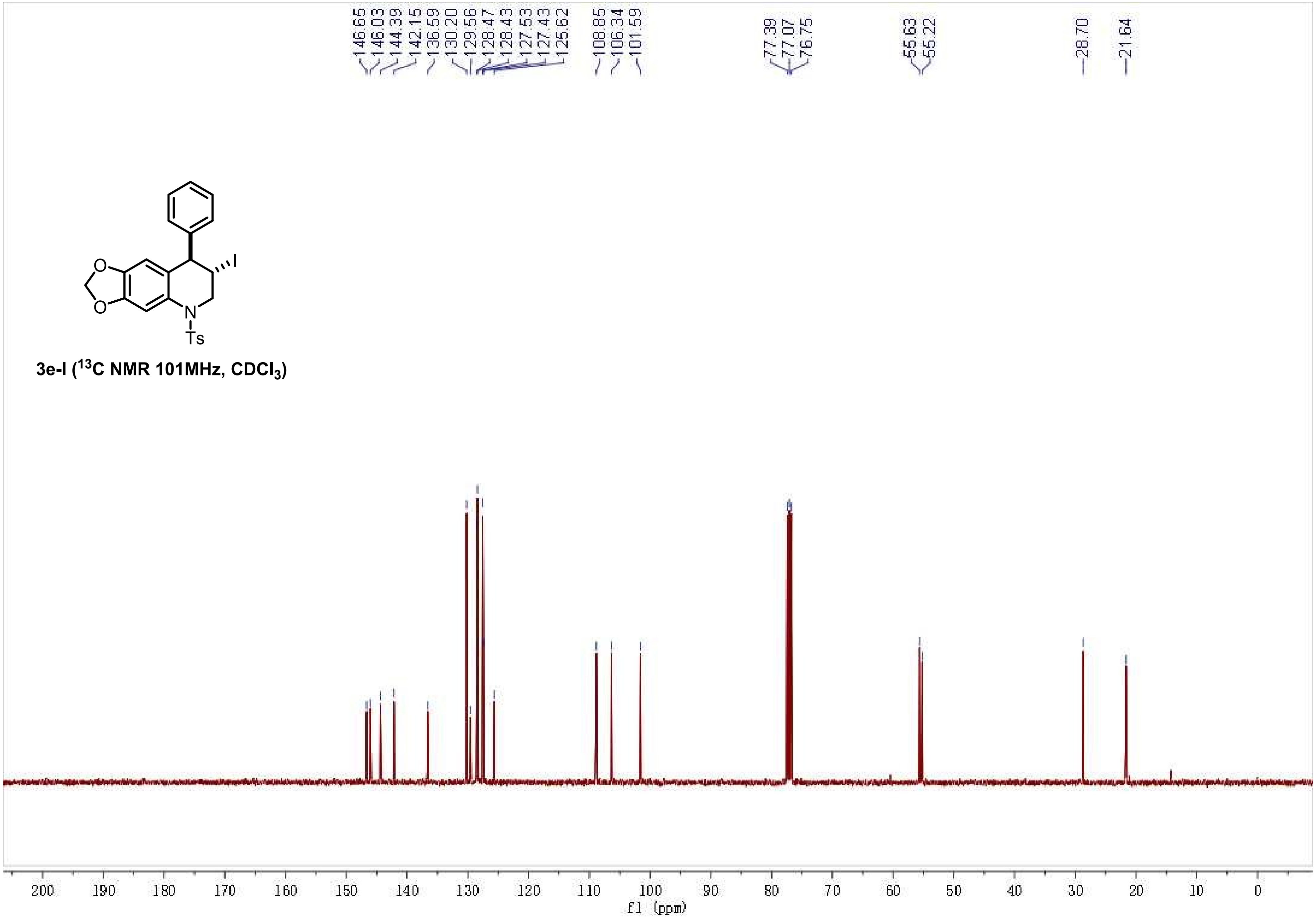

3e-I (^{13}C NMR 101MHz, $CDCl_3$)

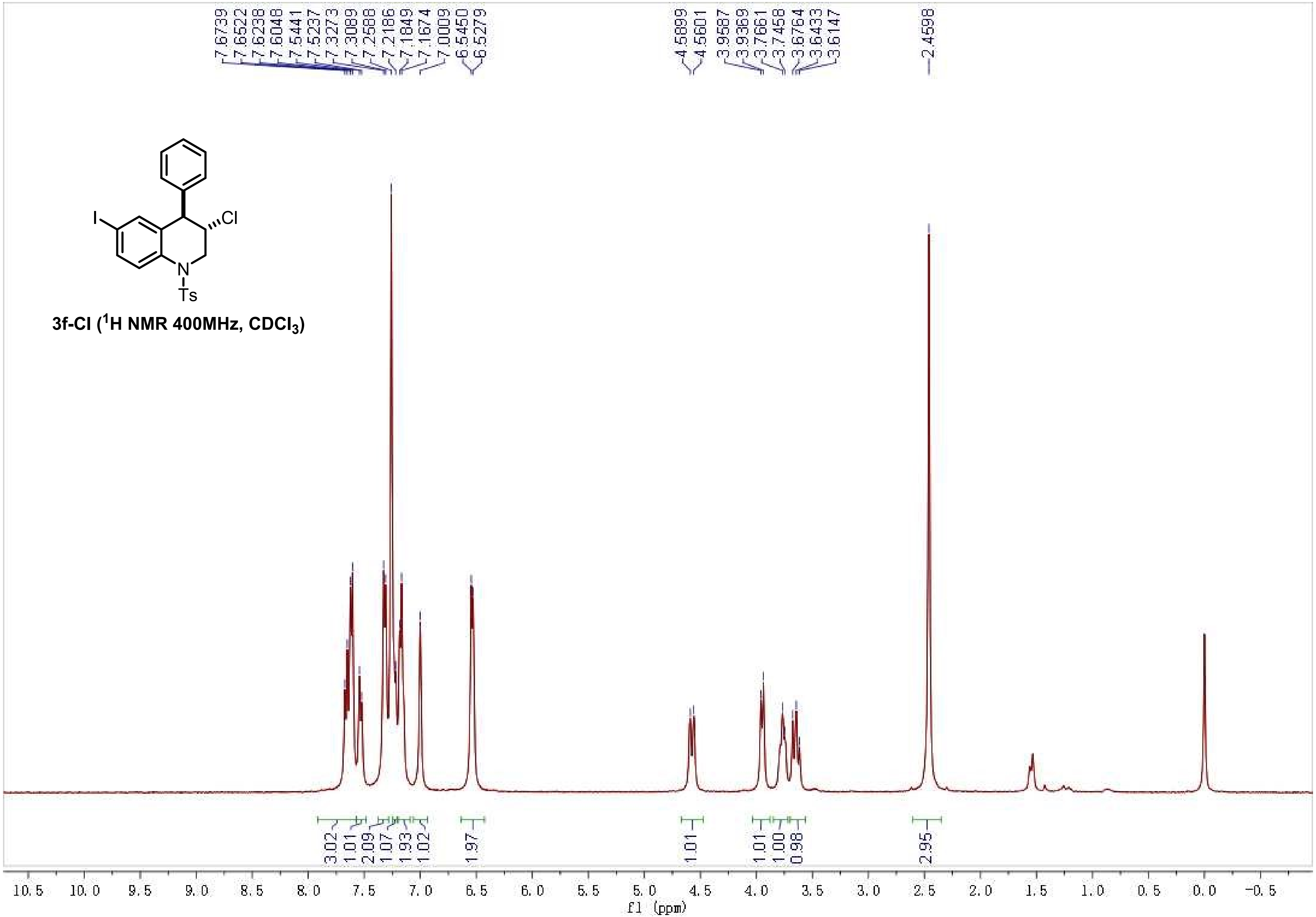

7.6739
7.6522
7.6238
7.6048
7.5441
7.5237
7.3273
7.3089
7.2588
7.2186
7.1849
7.1674
7.0009
6.5450
6.5279
4.5899
4.5601
3.9587
3.9369
3.7661
3.7458
3.6764
3.6433
3.6147
2.4598
I
Cl
N
Ts
3f-Cl (^{1}H NMR 400MHz, CDCl$_3$)
3.02
1.01
2.09
1.07
1.93
1.02
1.97
1.01
1.01
1.00
0.98
2.95
10.5 10.0 9.5 9.0 8.5 8.0 7.5 7.0 6.5 6.0 5.5 5.0 4.5 4.0 3.5 3.0 2.5 2.0 1.5 1.0 0.5 0.0 -0.5
f1 (ppm)

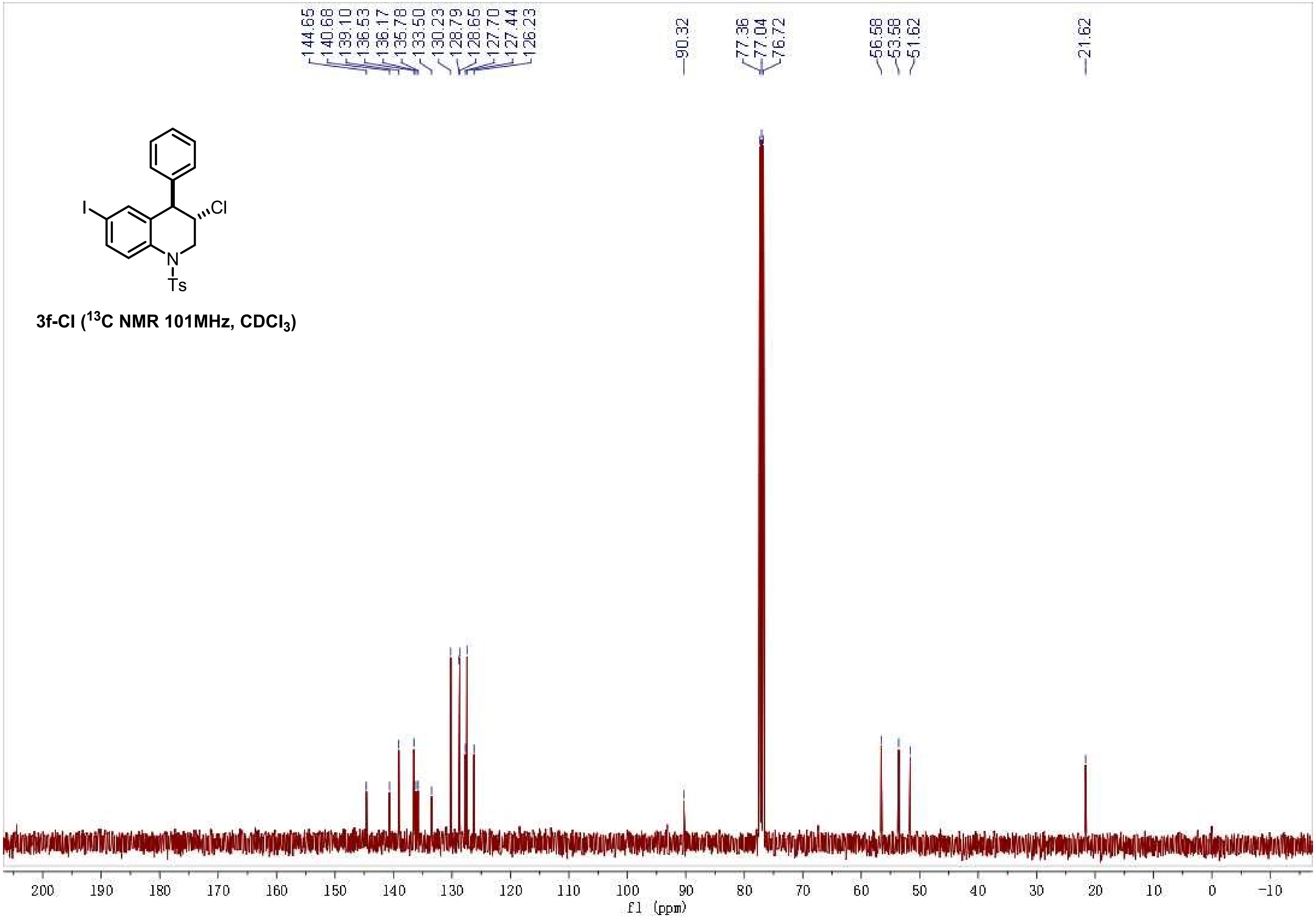

3f-Cl (^{13}C NMR 101MHz, $CDCl_3$)

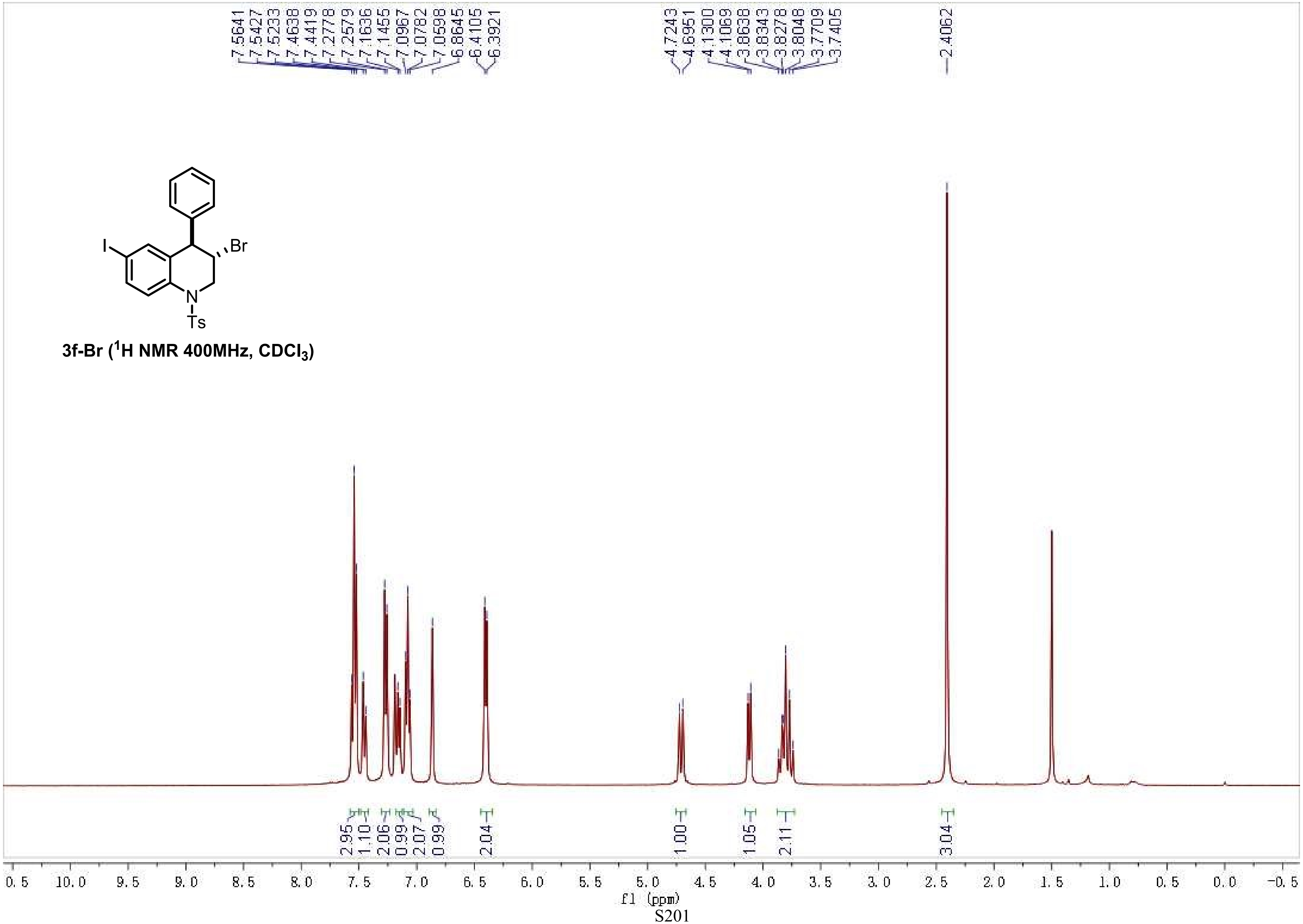

3f-Br (^{1}H NMR 400MHz, $CDCl_3$)

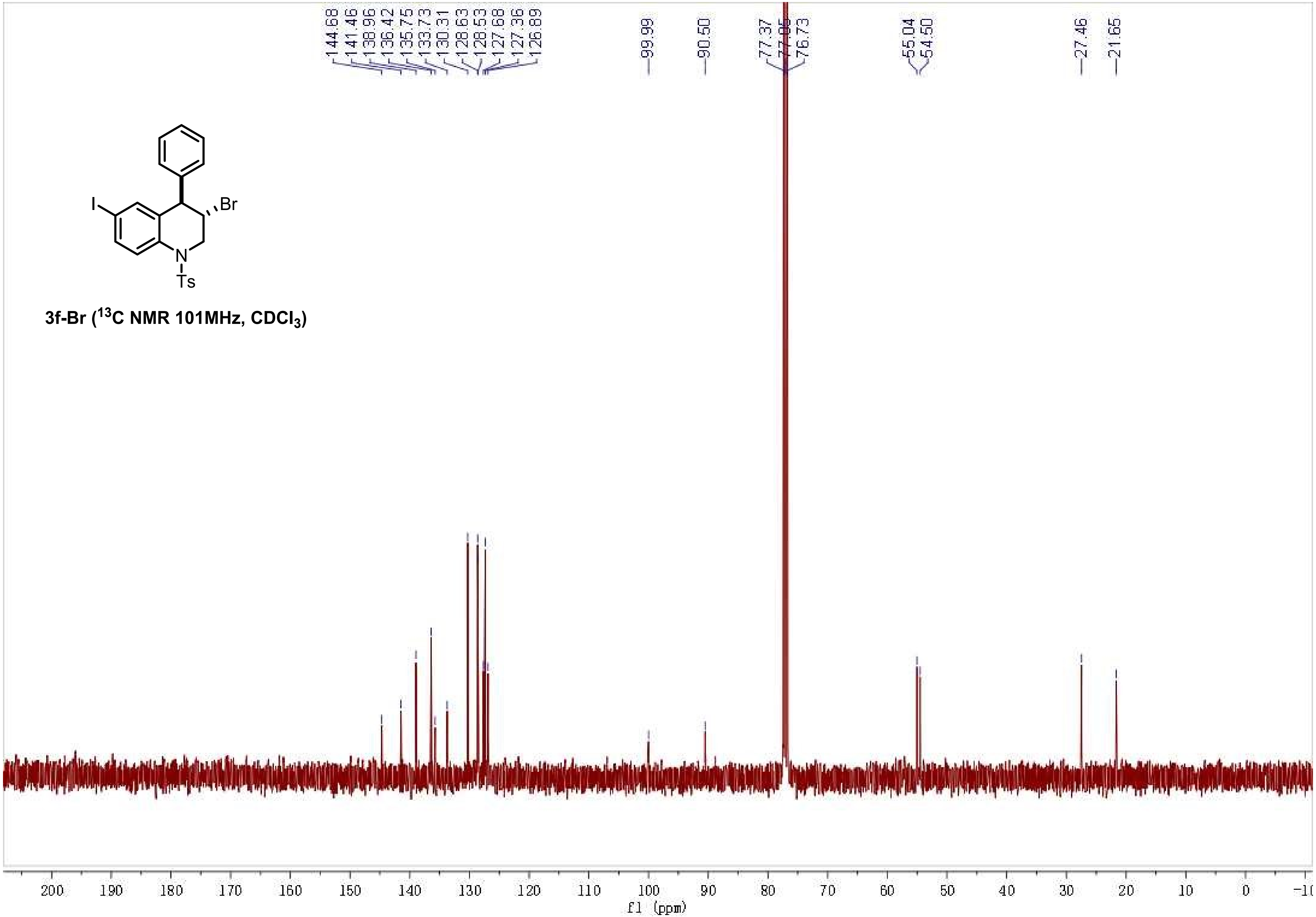

3f-Br (^{13}C NMR 101MHz, $CDCl_3$)

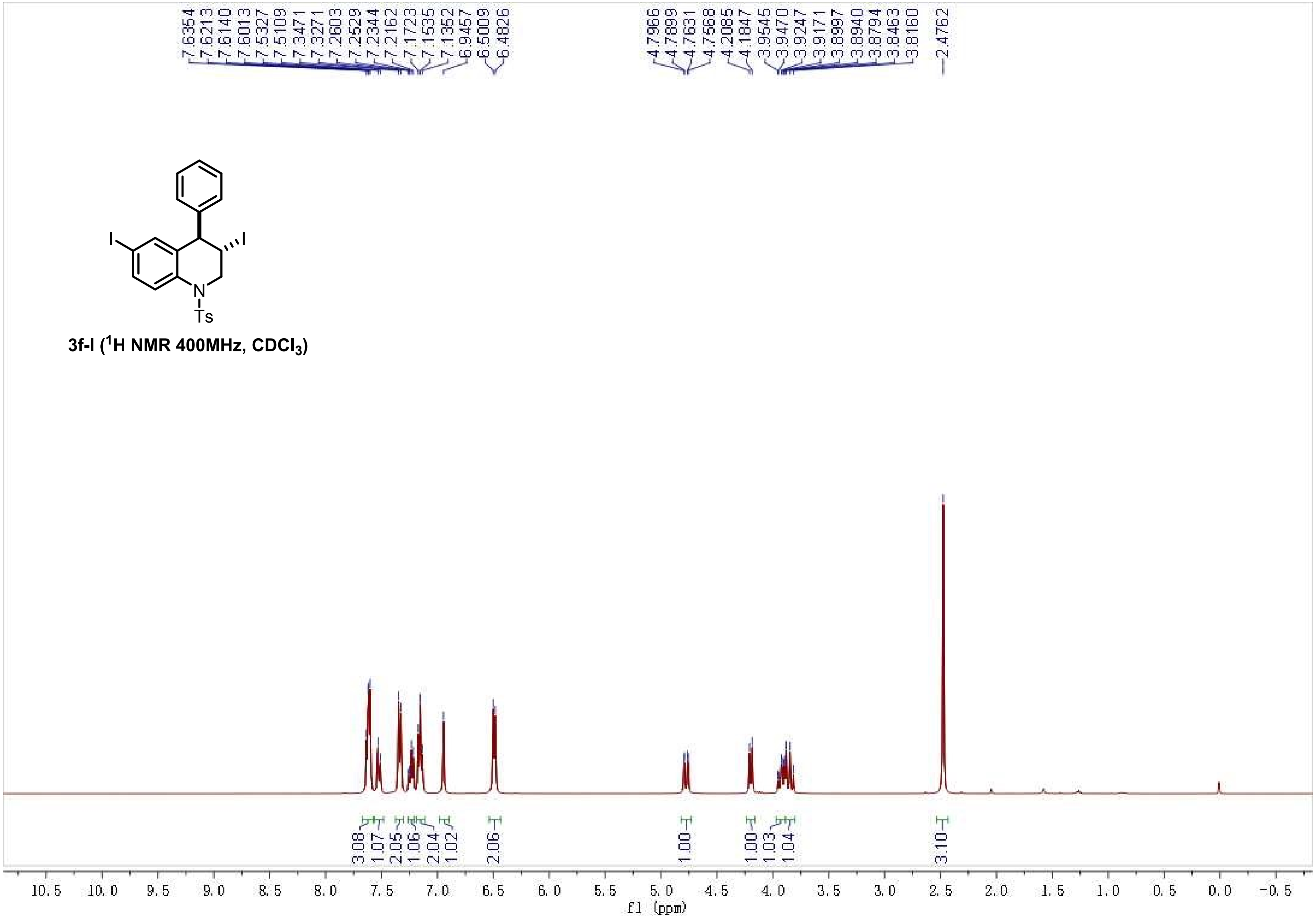

3f-I (^{1}H NMR 400MHz, $CDCl_3$)

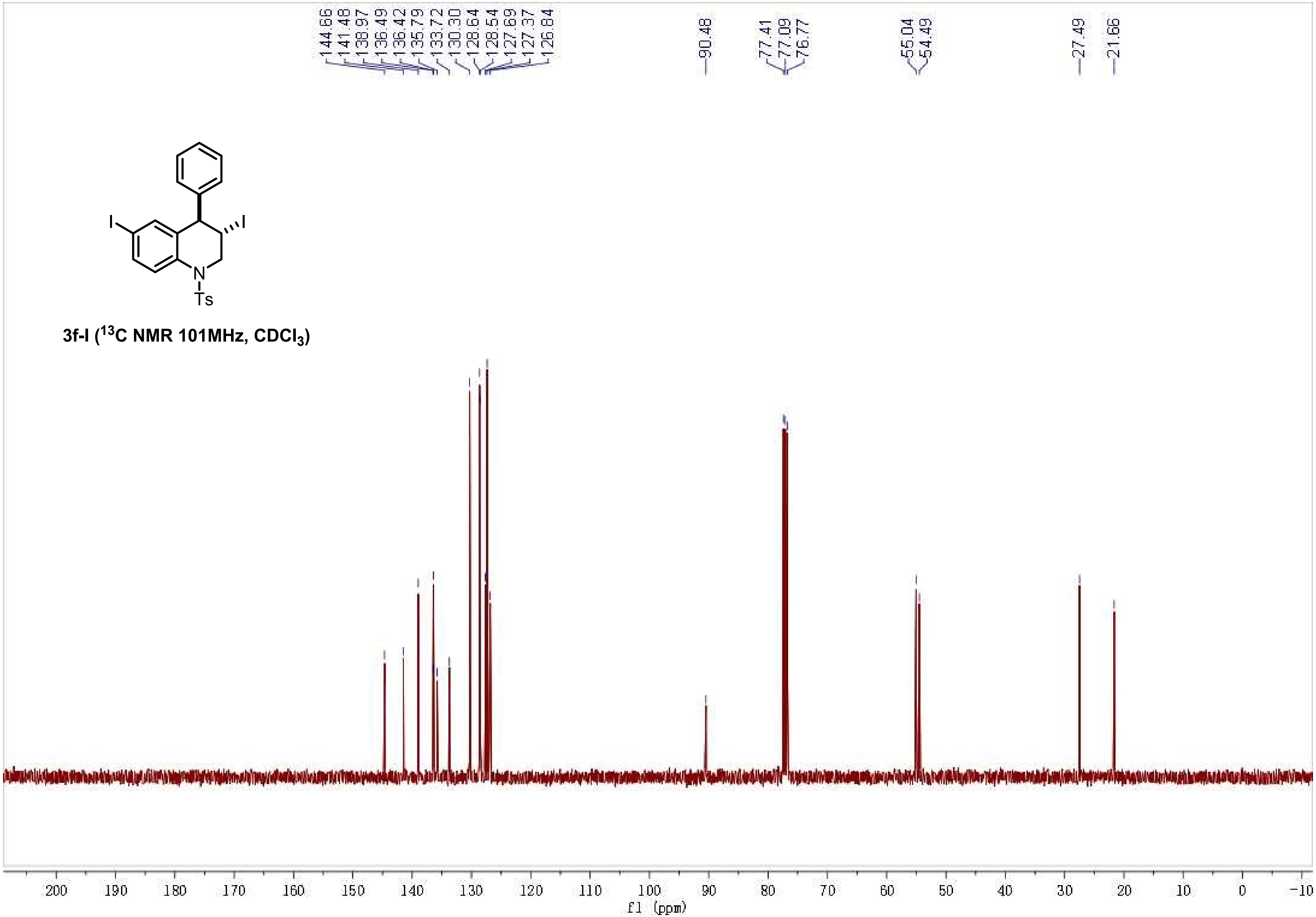

144.66
141.48
138.97
136.49
136.42
135.79
133.72
130.30
128.64
128.54
127.69
127.37
126.84
90.48
77.41
77.09
76.77
55.04
54.49
27.49
21.66
3f-I (13C NMR 101MHz, CDCl3)
200 190 180 170 160 150 140 130 120 110 100 90 80 70 60 50 40 30 20 10 0 -10
f1 (ppm)

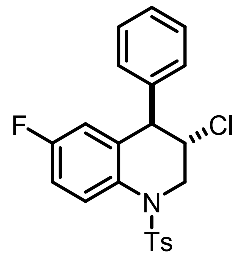

3g-Cl (^{1}H NMR 400MHz, $CDCl_3$)

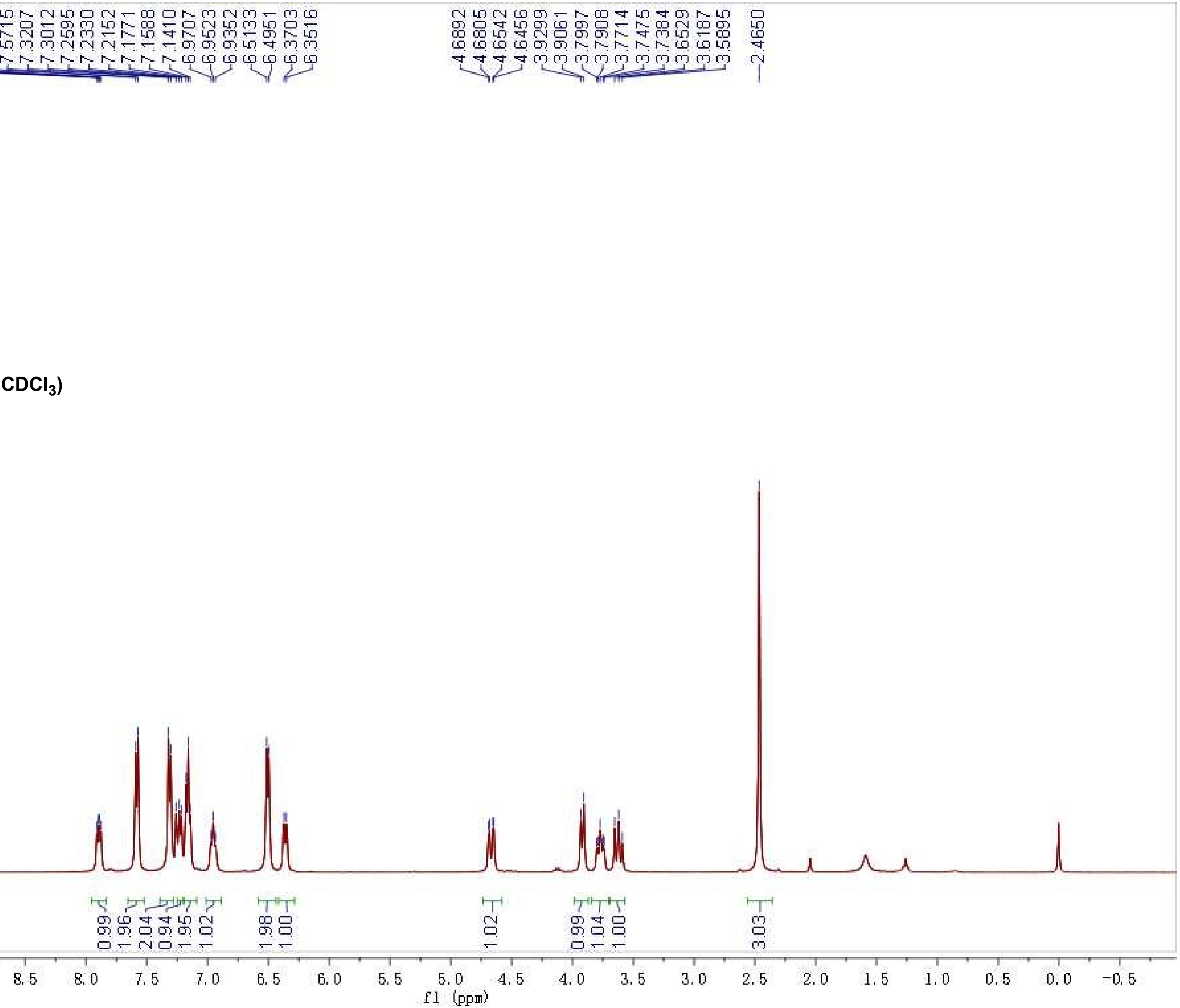

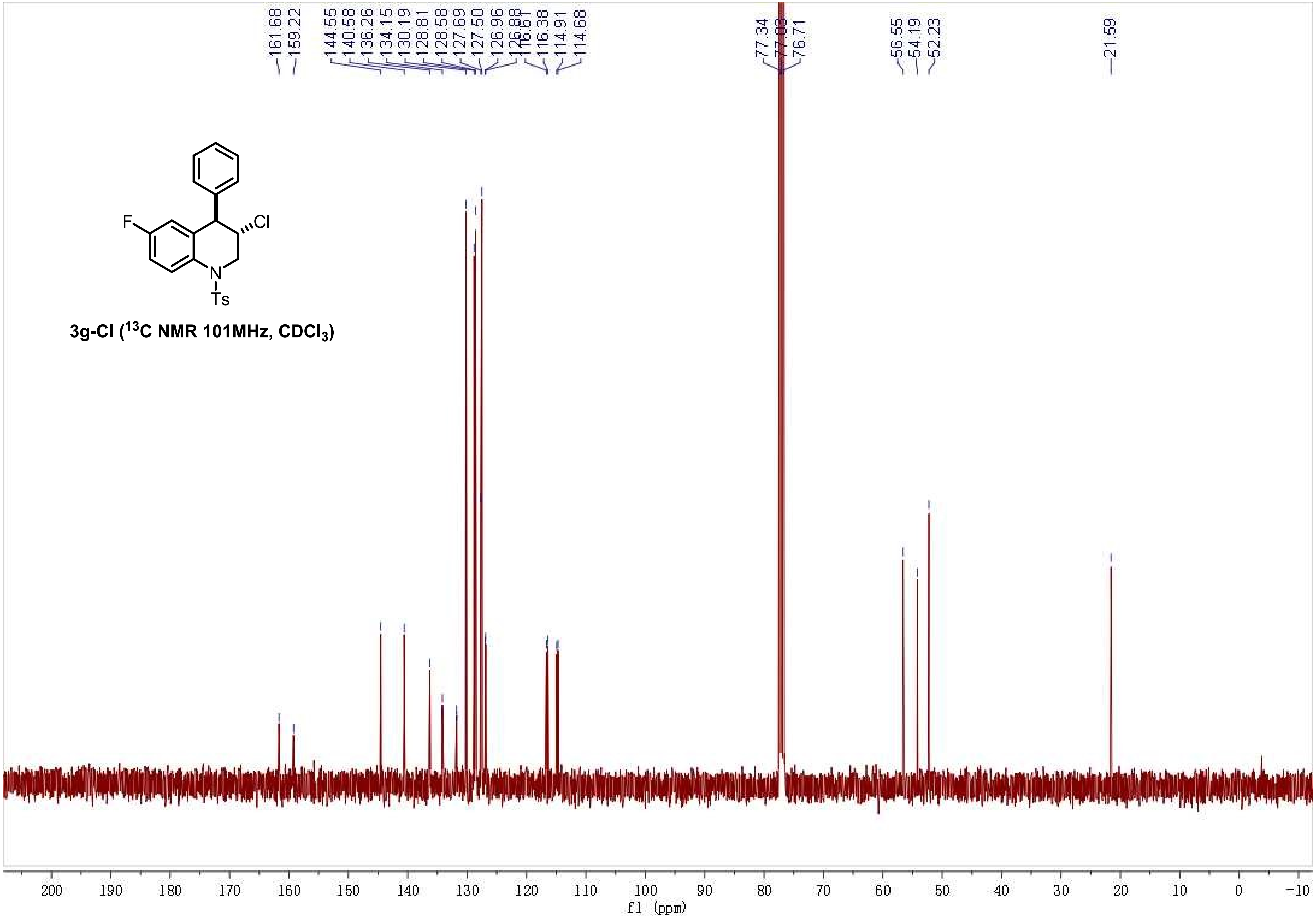

3g-Cl (^{13}C NMR 101MHz, $CDCl_3$)

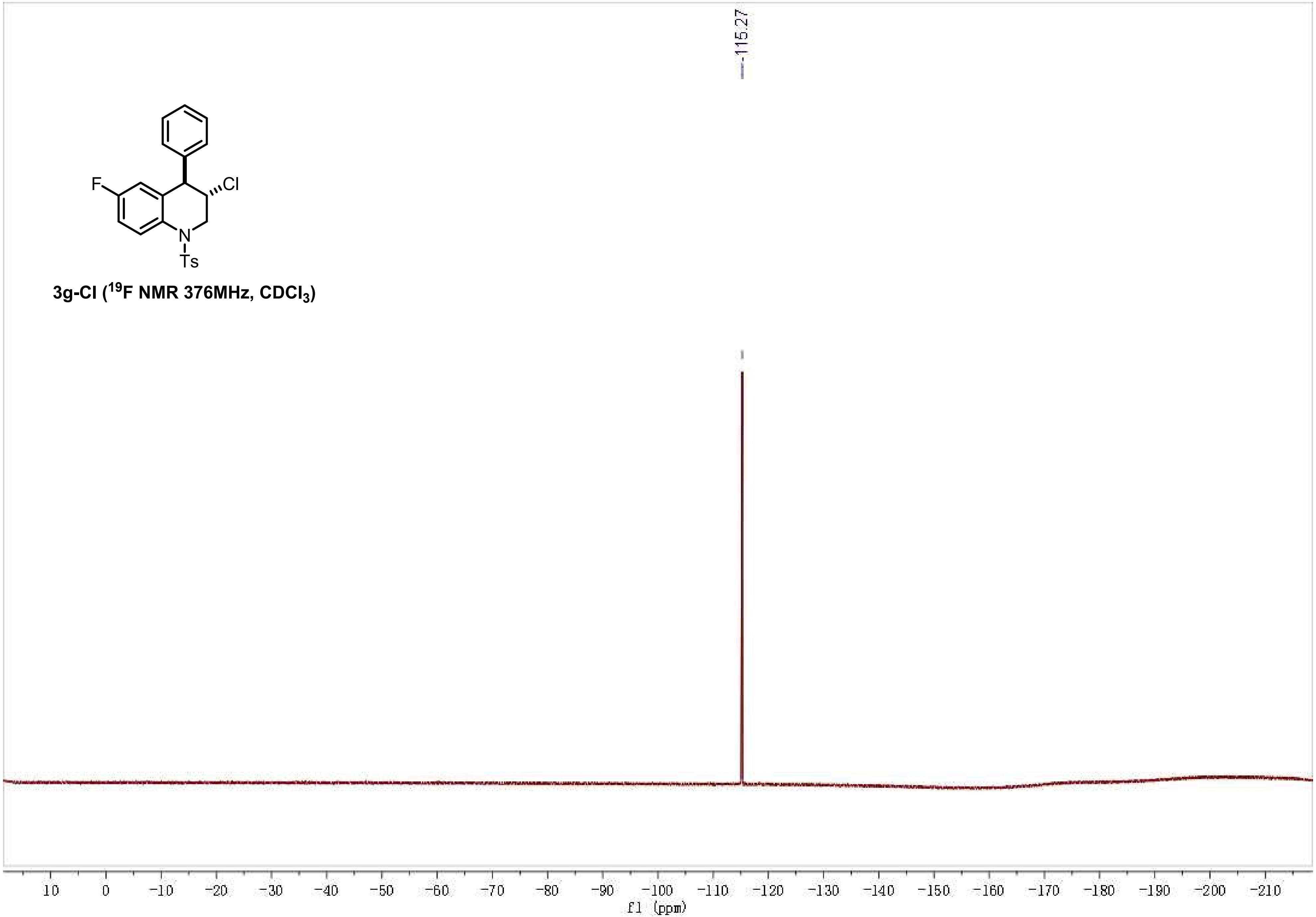

3g-Cl (^{19}F NMR 376MHz, $CDCl_3$)

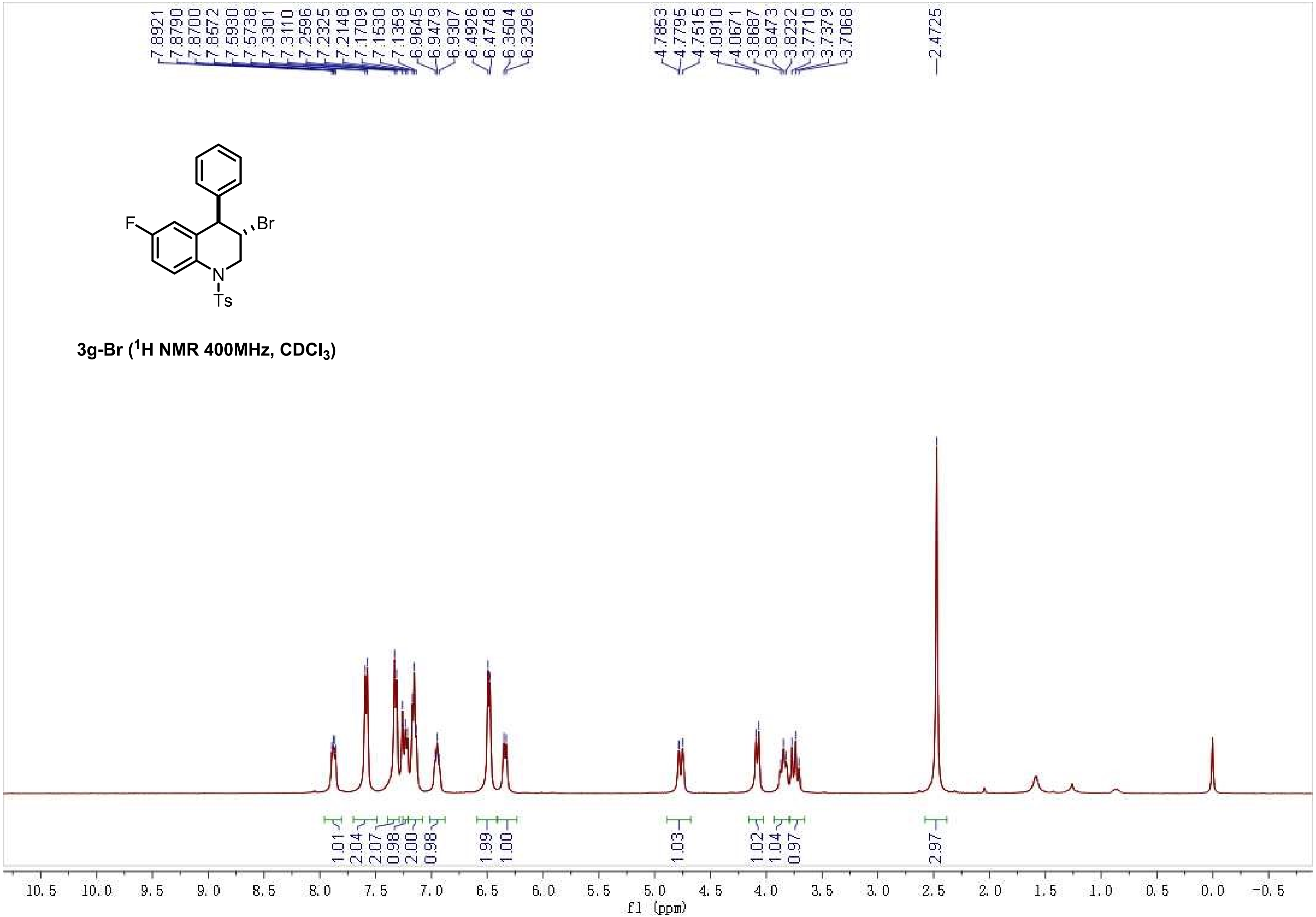

3g-Br (^{1}H NMR 400MHz, $CDCl_3$)

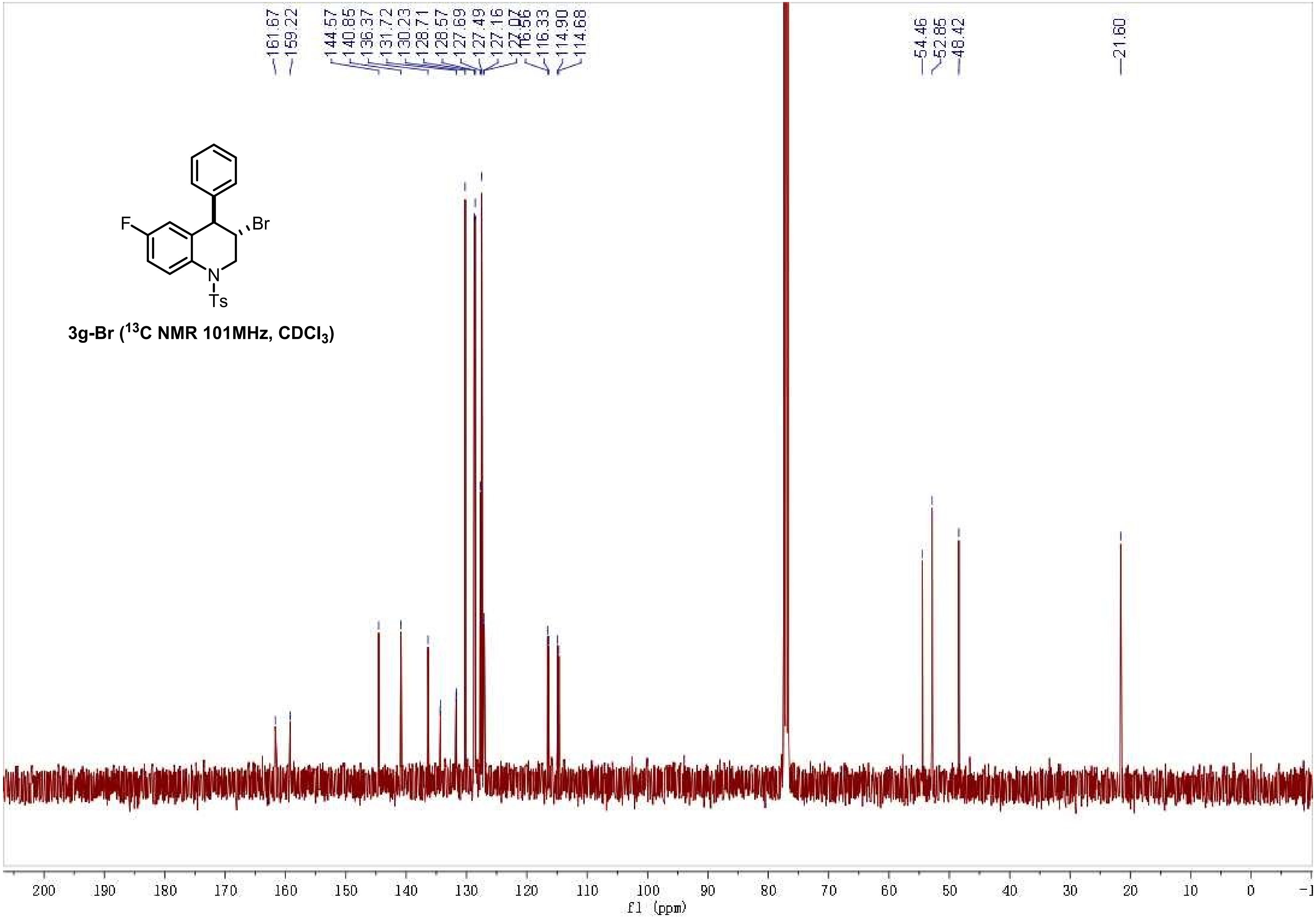

3g-Br (^{13}C NMR 101MHz, $CDCl_3$)

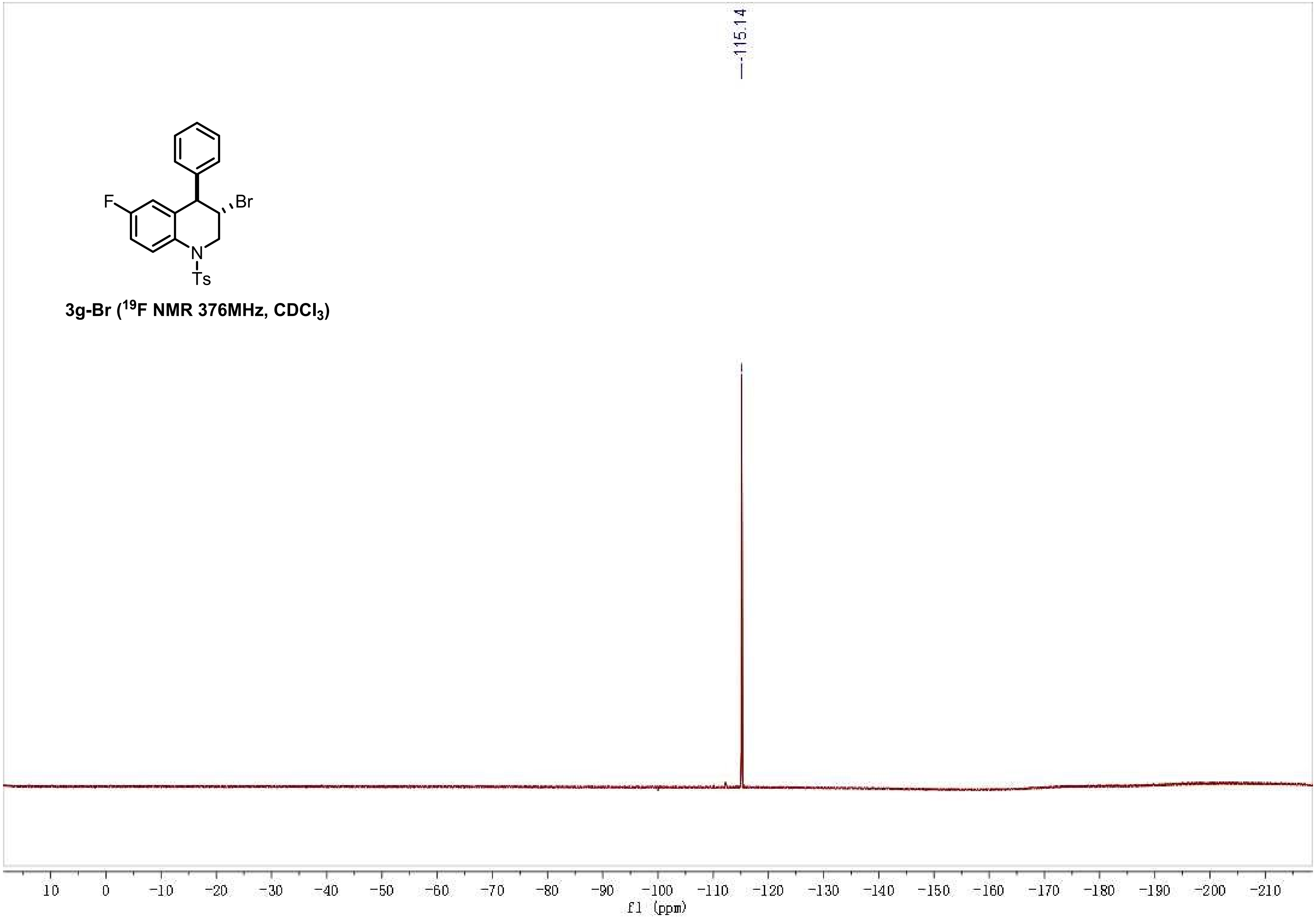

3g-Br (^{19}F NMR 376MHz, $CDCl_3$)

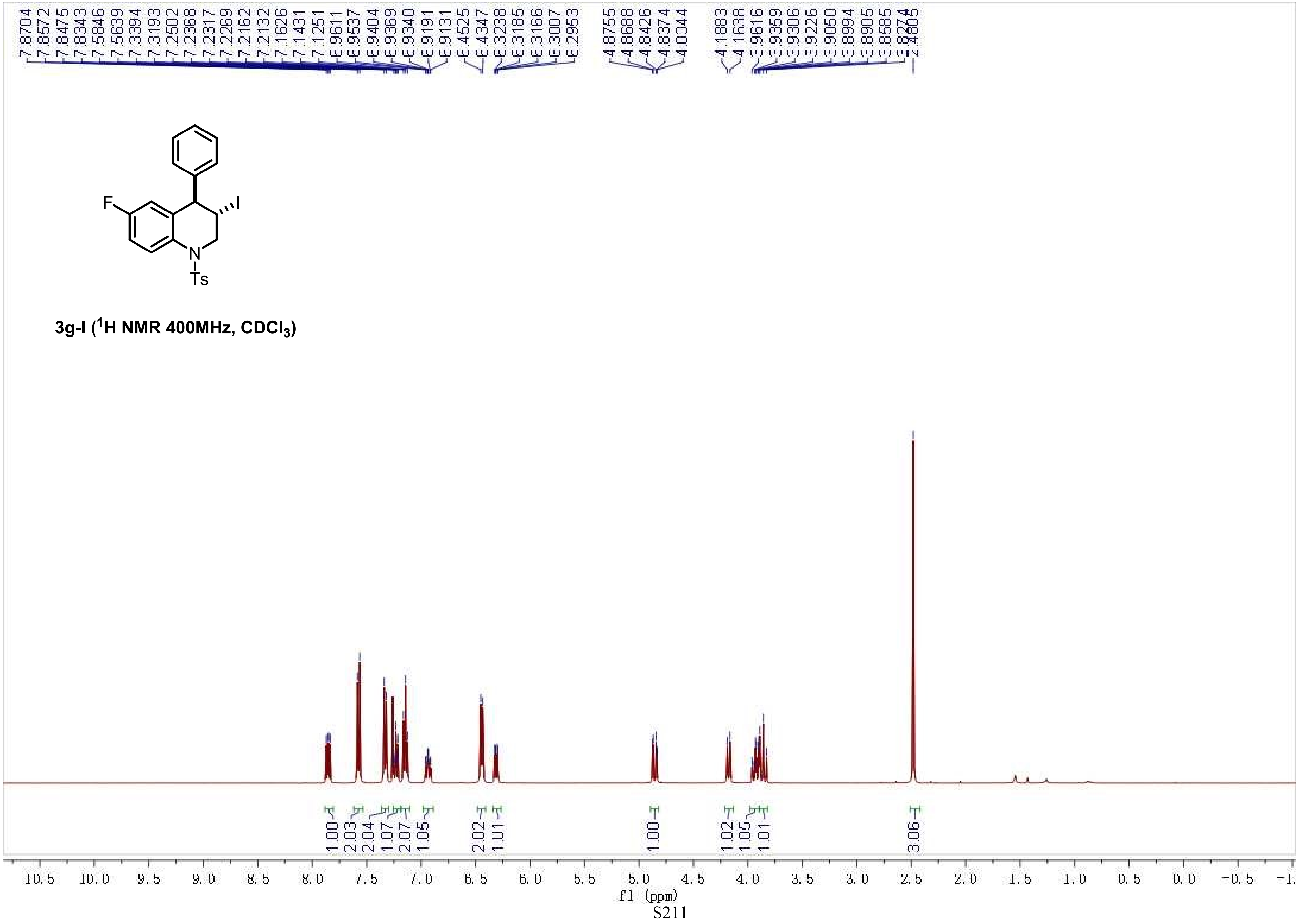

3g-I (^{1}H NMR 400MHz, $CDCl_3$)

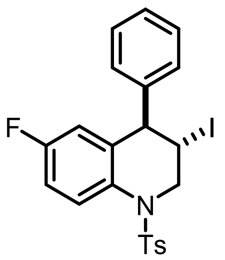

3g-I (^{13}C NMR 101MHz, $CDCl_3$)

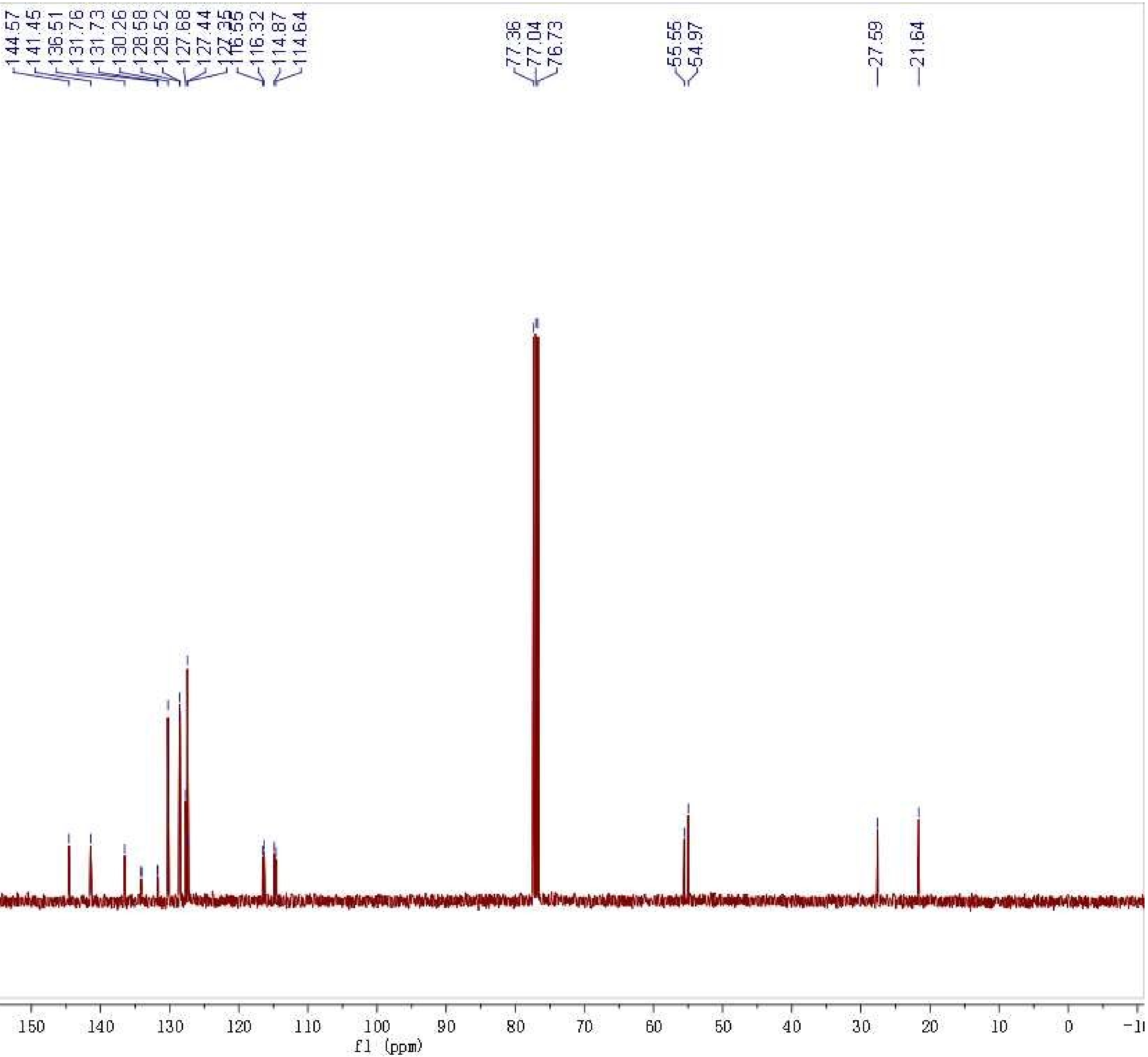

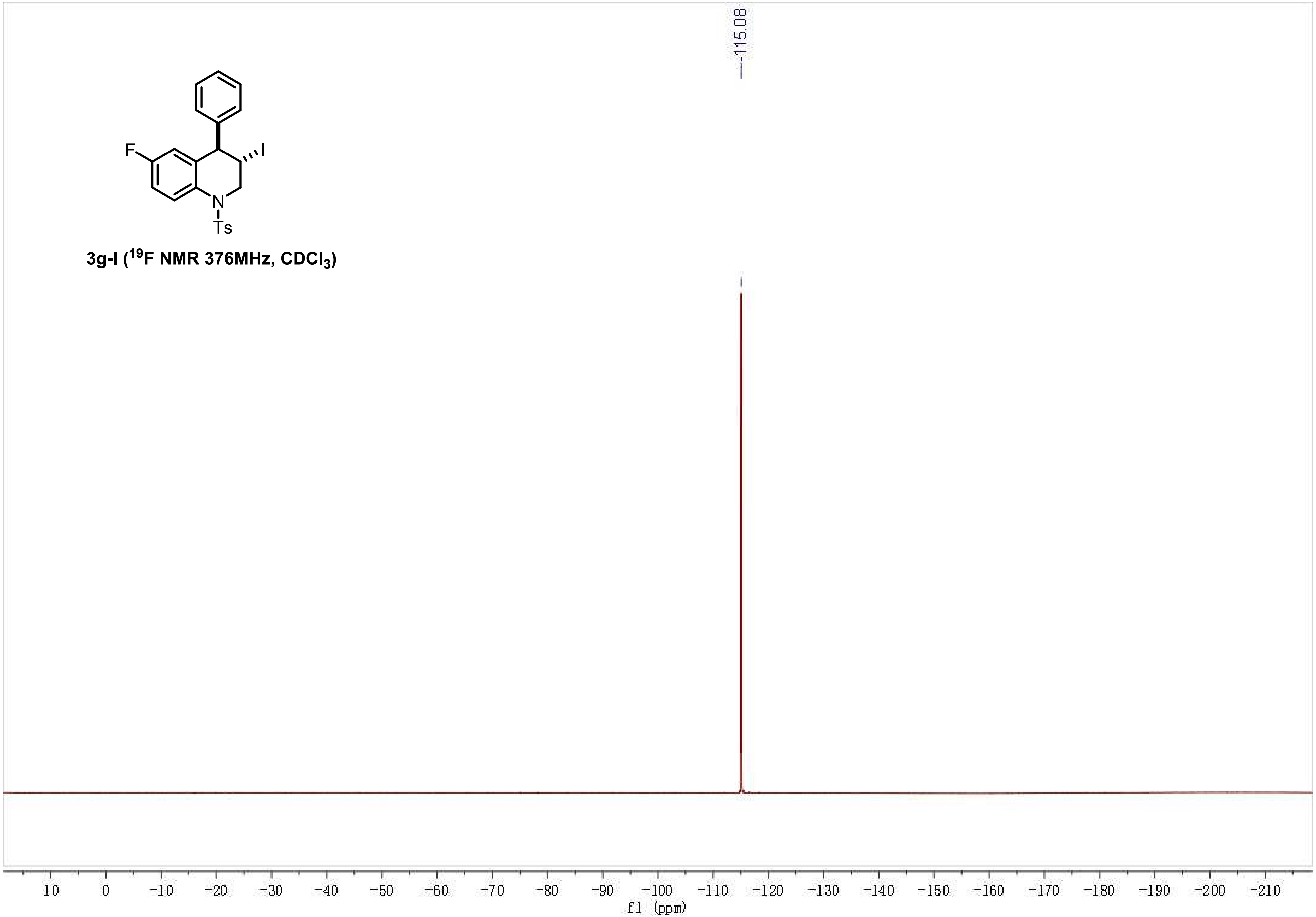

3g-I (^{19}F NMR 376MHz, $CDCl_3$)

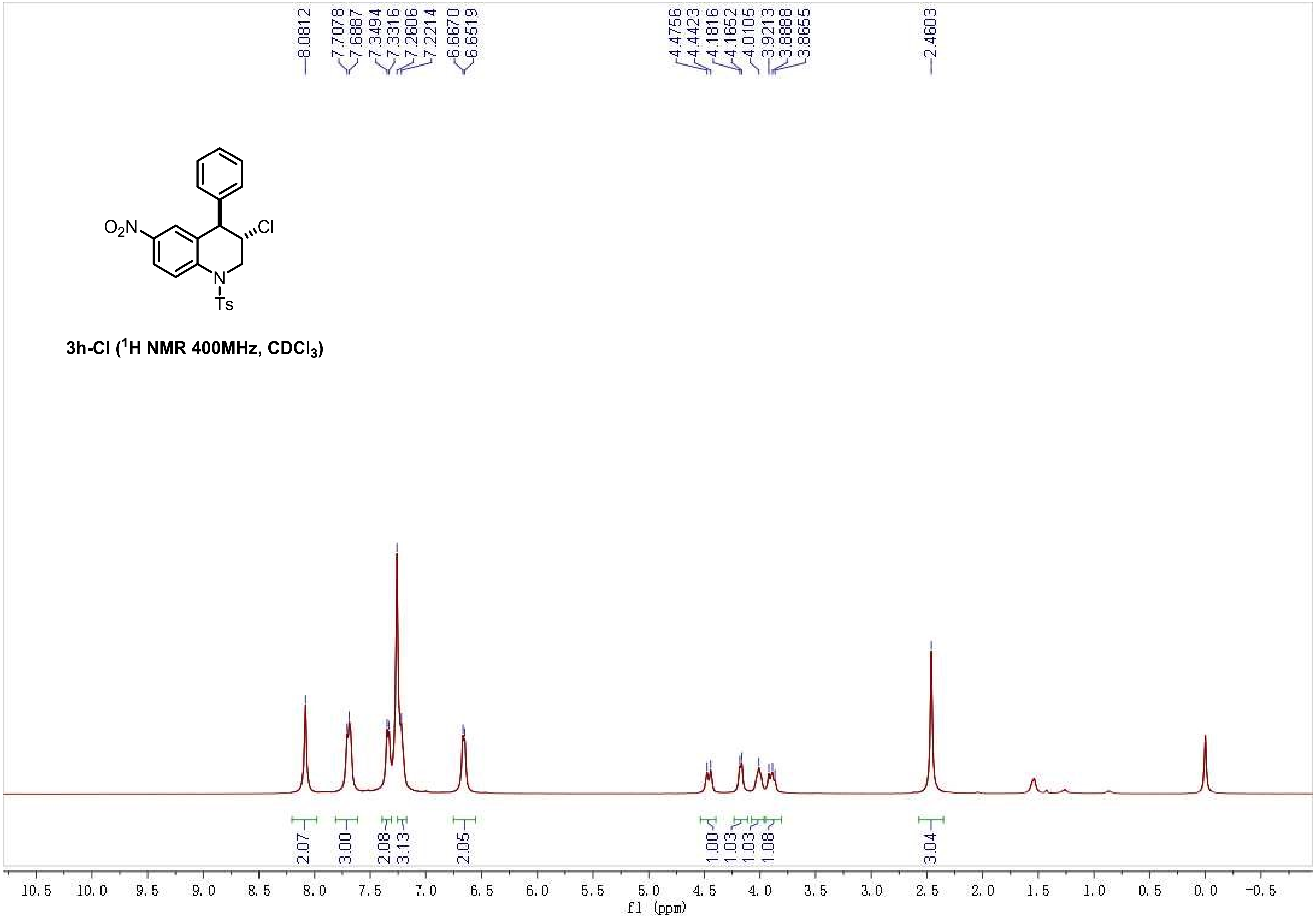

3h-Cl (^{1}H NMR 400MHz, $CDCl_3$)

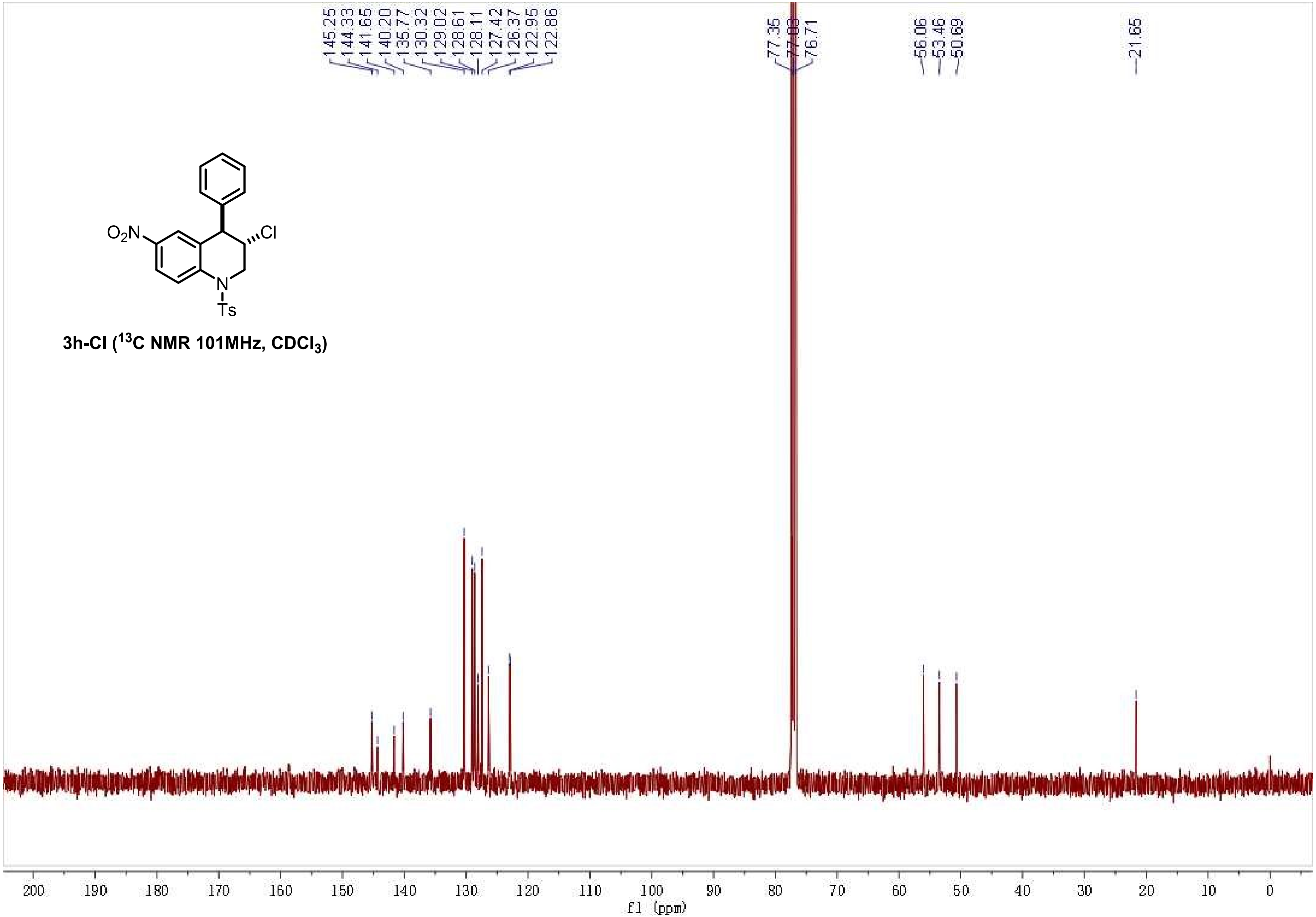

3h-Cl (^{13}C NMR 101MHz, $CDCl_3$)

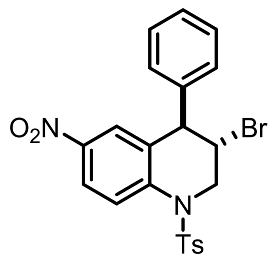

3h-Br (^{1}H NMR 400MHz, $CDCl_3$)

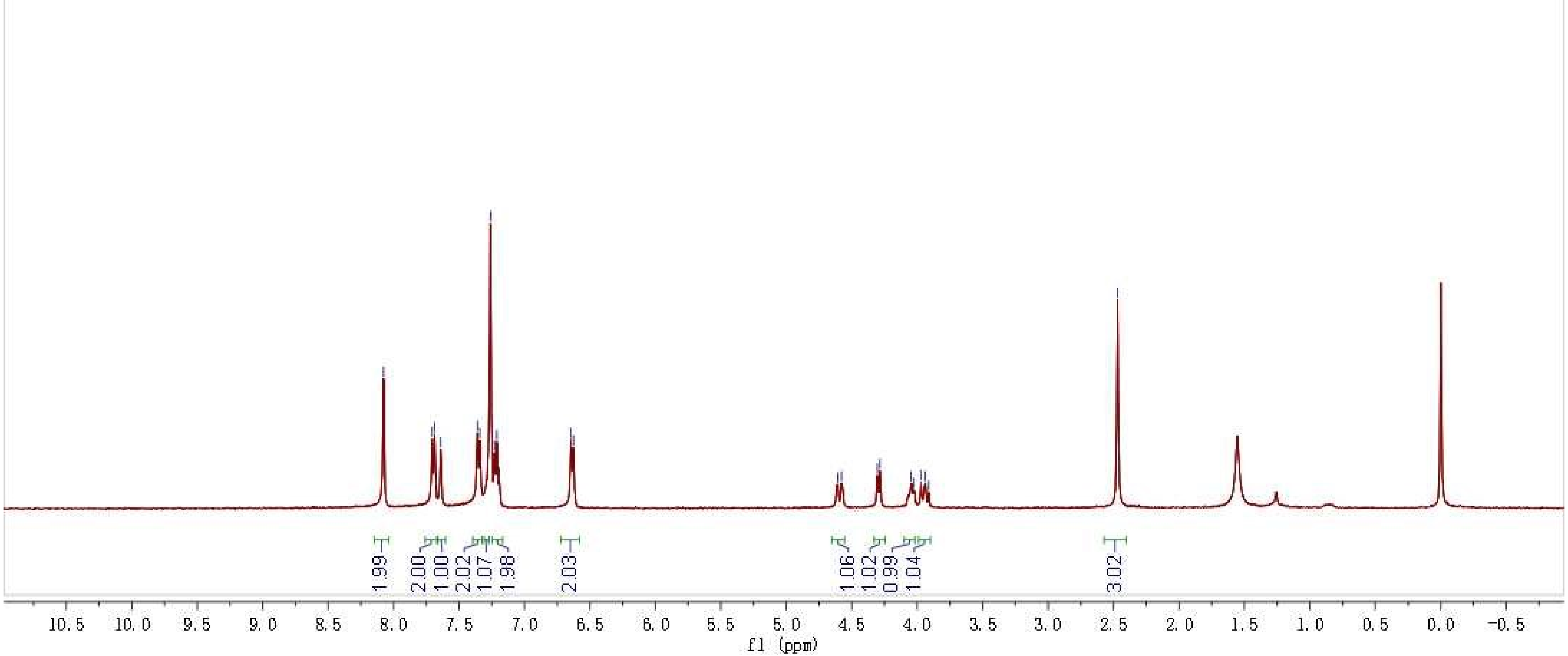

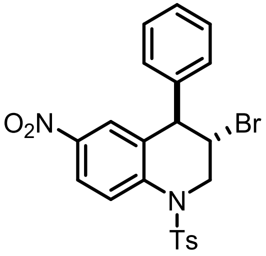

3h-Br (^{13}C NMR 101MHz, $CDCl_3$)

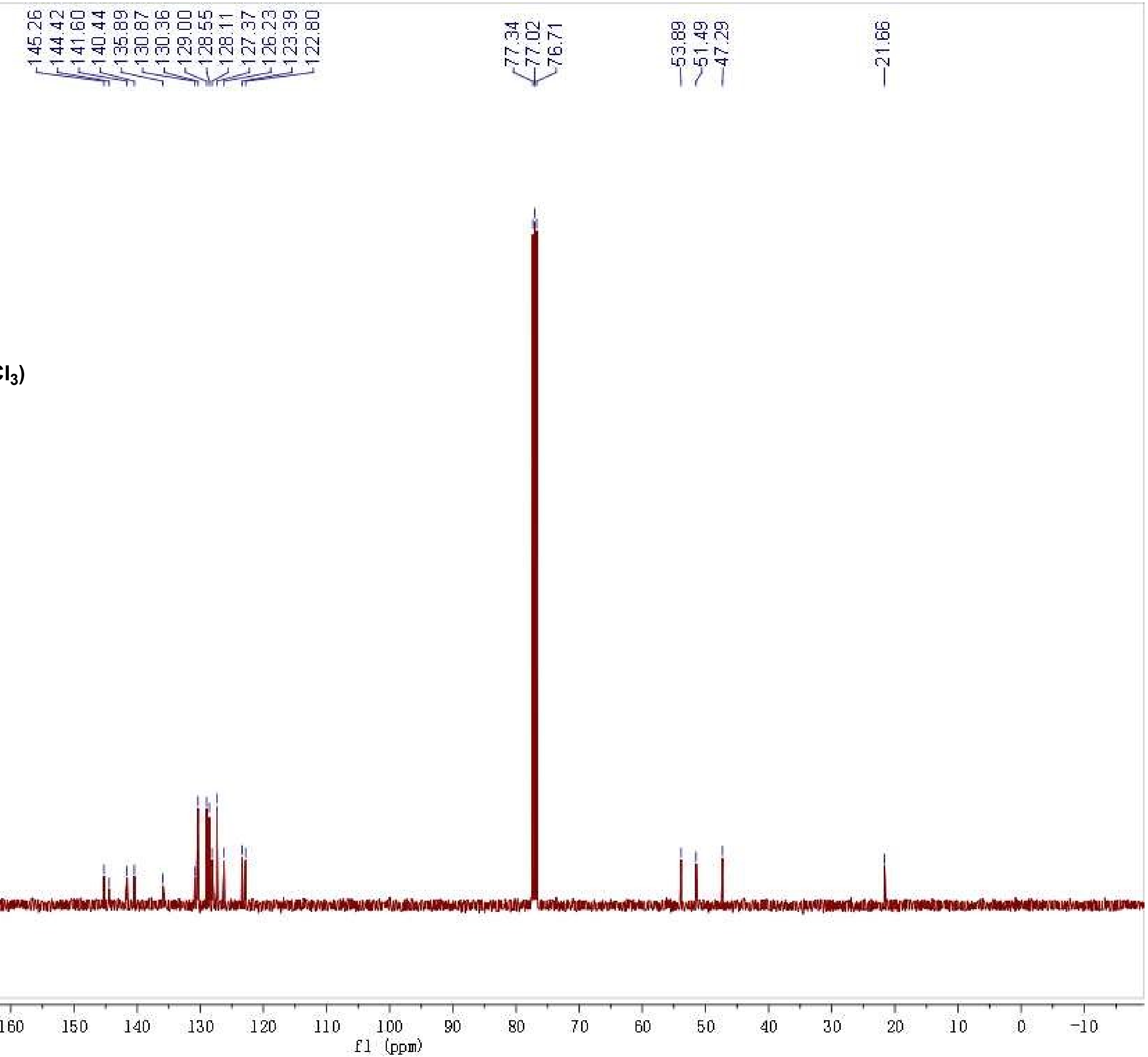

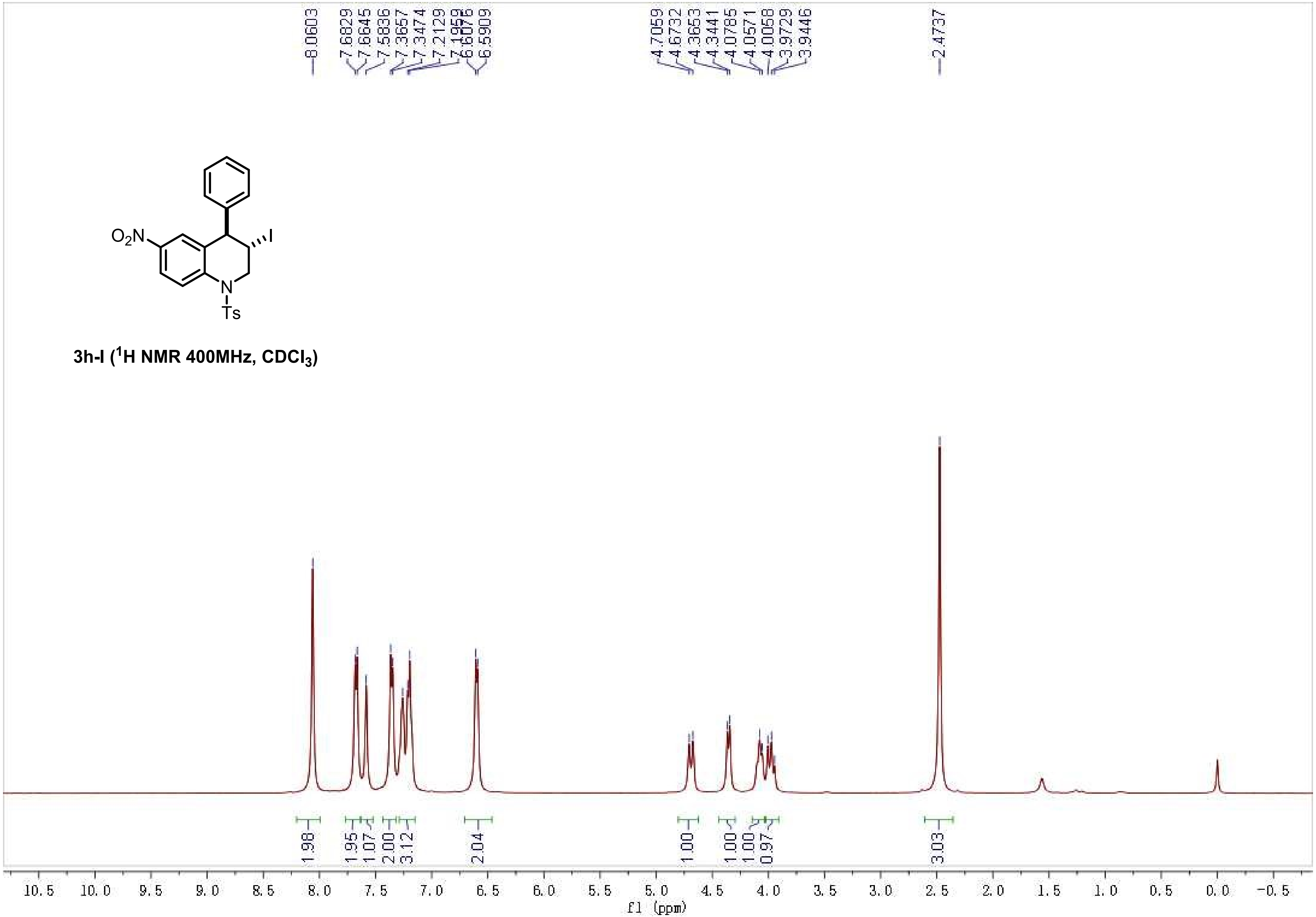

3h-I (^{1}H NMR 400MHz, $CDCl_3$)

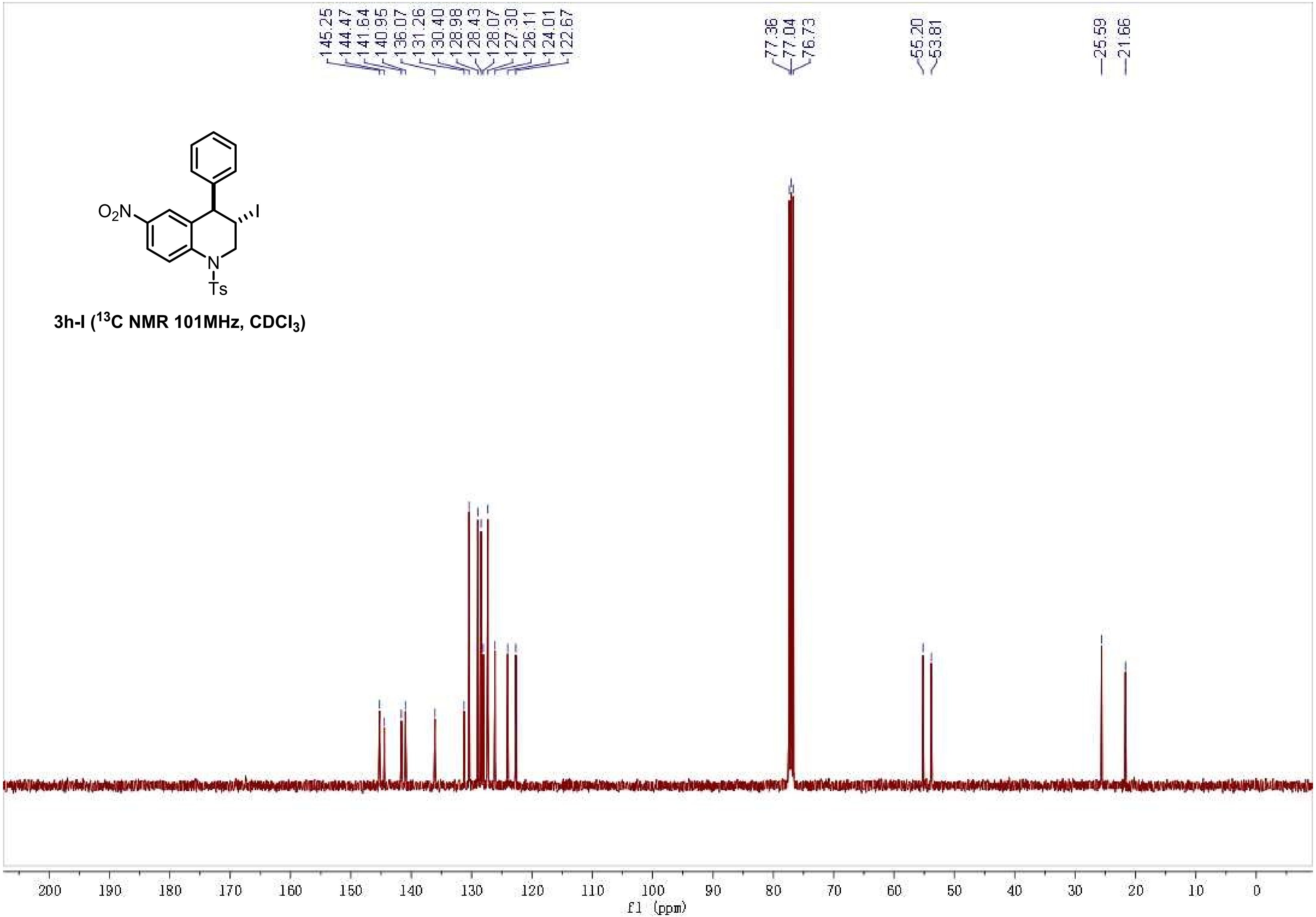
O2N
I
N
Ts
3h-I (13C NMR 101MHz, CDCl3)
145.25
144.47
141.64
140.95
136.07
131.26
130.40
128.98
128.43
128.07
127.30
126.11
124.01
122.67
77.36
77.04
76.73
55.20
53.81
25.59
21.66
200
190
180
170
160
150
140
130
120
110
100
90
80
70
60
50
40
30
20
10
0
f1 (ppm)

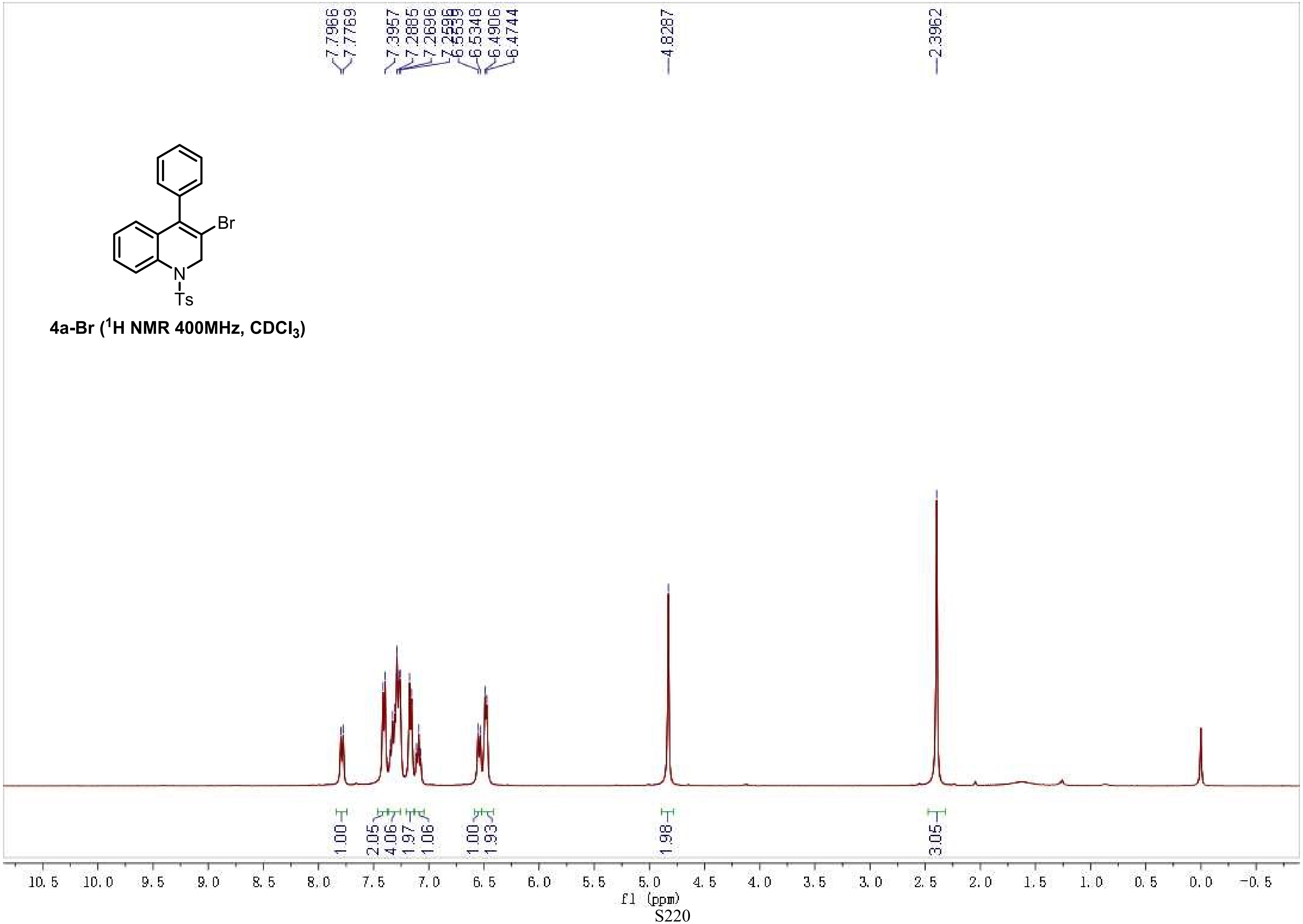

7.7966
7.7769
7.3957
7.2885
7.2696
6.5348
6.4906
6.4744
4.8287
2.3962
Br
N
Ts
4a-Br (^{1}H NMR 400MHz, $CDCl_3$)
1.00
2.05
4.06
1.97
1.06
1.00
1.93
1.98
3.05
10.5 10.0 9.5 9.0 8.5 8.0 7.5 7.0 6.5 6.0 5.5 5.0 4.5 4.0 3.5 3.0 2.5 2.0 1.5 1.0 0.5 0.0 -0.5
f1 (ppm)

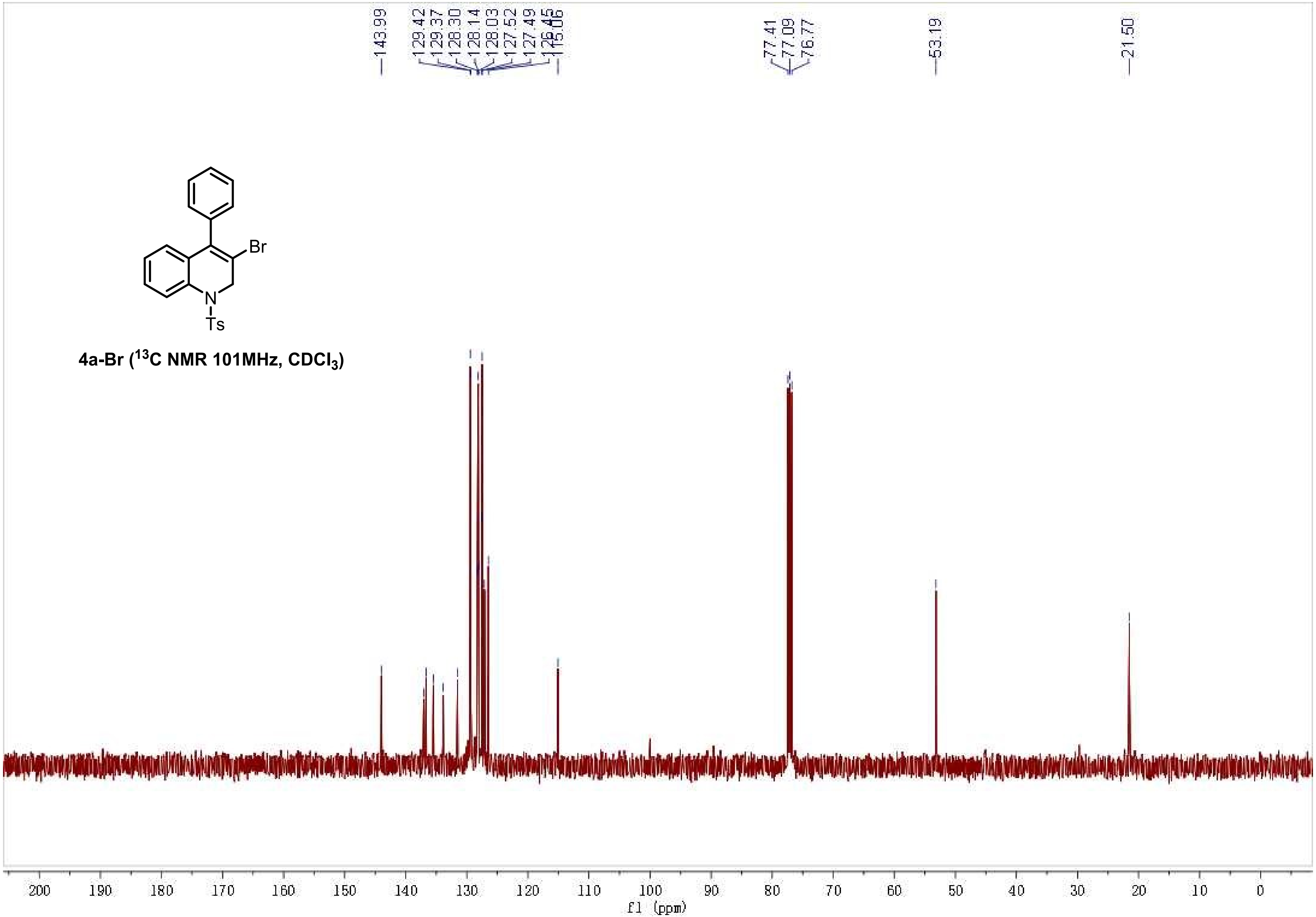

4a-Br (^{13}C NMR 101MHz, $CDCl_3$)

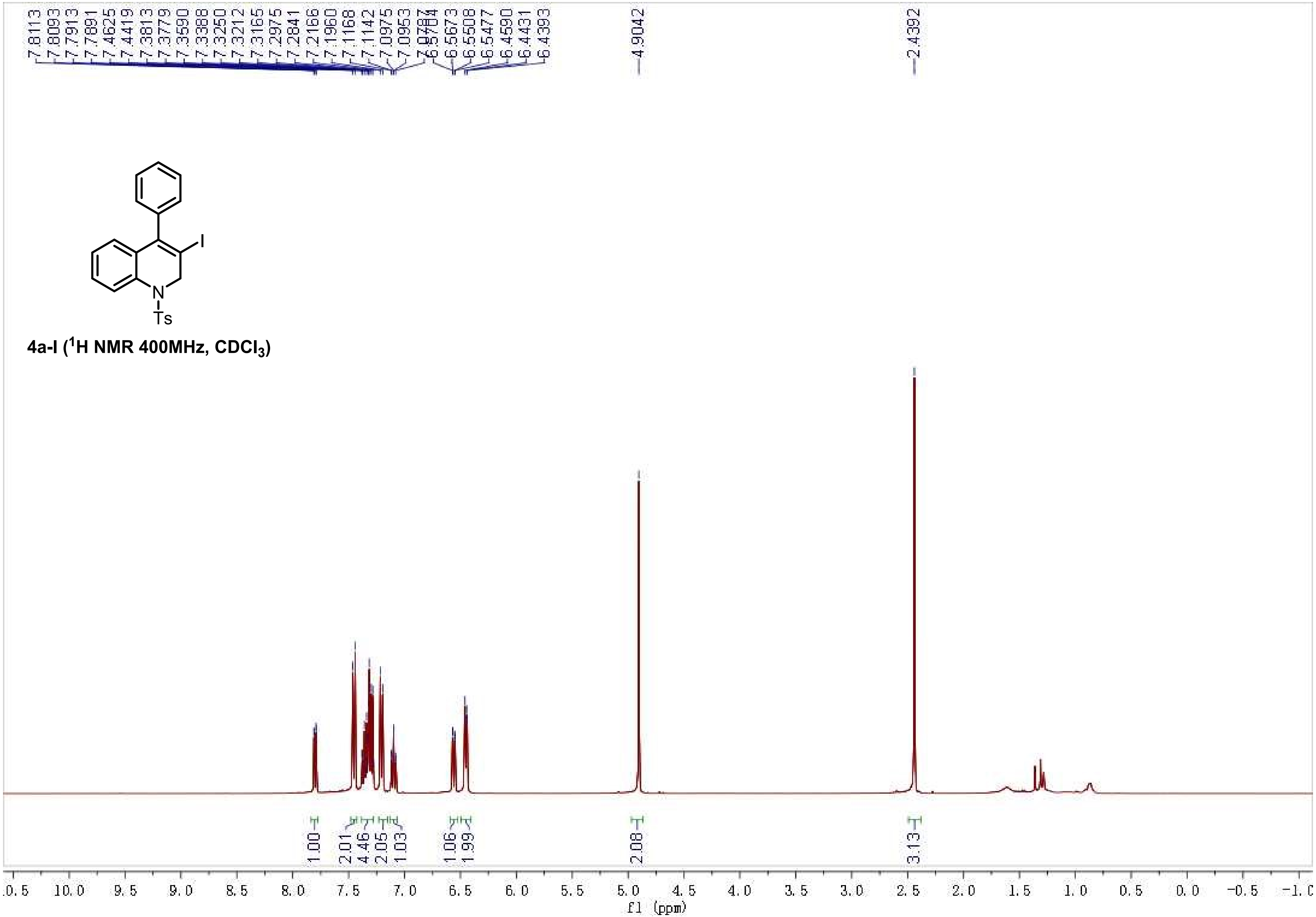
7.8113
7.8093
7.7913
7.7891
7.4625
7.4419
7.3813
7.3779
7.3590
7.3388
7.3250
7.3212
7.3165
7.2975
7.2841
7.2166
7.1960
7.1168
7.1142
7.0975
7.0953
7.0787
6.5704
6.5673
6.5508
6.5477
6.4590
6.4431
6.4393
4.9042
2.4392
I
N
Ts
4a-I (1H NMR 400MHz, CDCl3)
1.00
2.01
4.46
2.05
1.03
1.06
1.99
2.08
3.13
f1 (ppm)

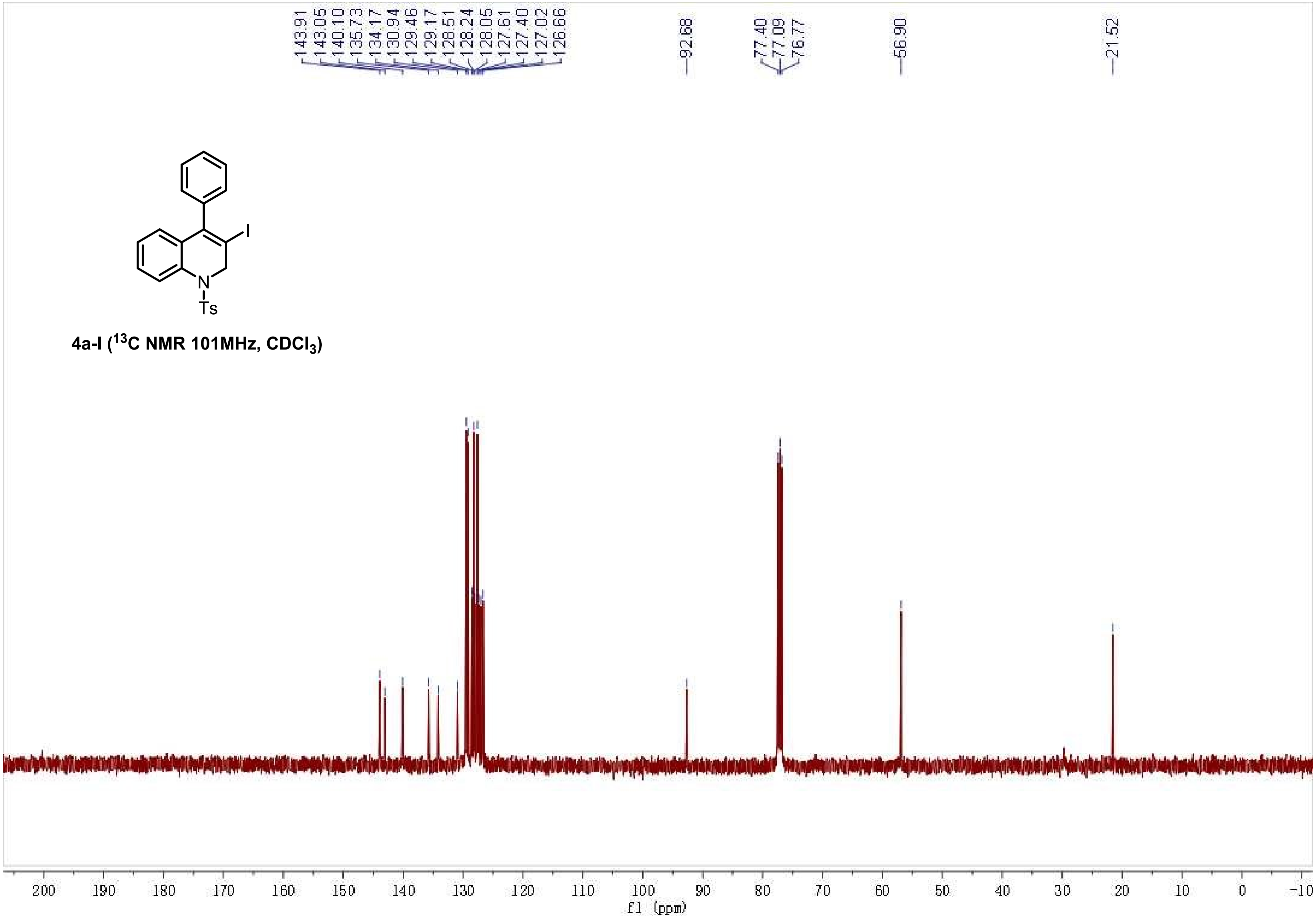
143.91
143.05
140.10
135.73
134.17
130.94
129.46
129.17
128.51
128.24
128.05
127.61
127.40
127.02
126.66
92.68
77.40
77.09
76.77
56.90
21.52
I
N
Ts
4a-I (13C NMR 101MHz, CDCl3)
f1 (ppm)

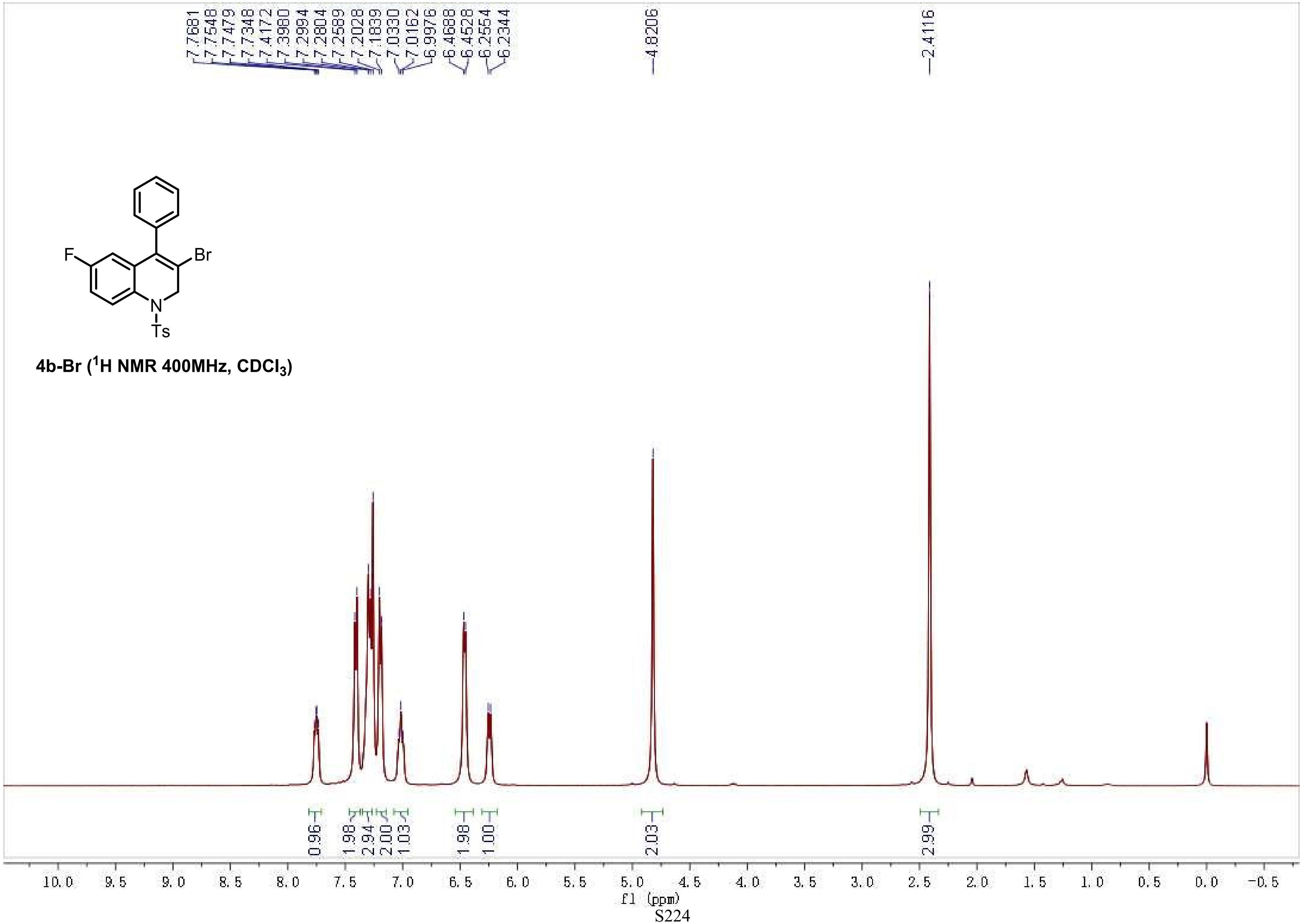

4b-Br (^{1}H NMR 400MHz, $CDCl_3$)

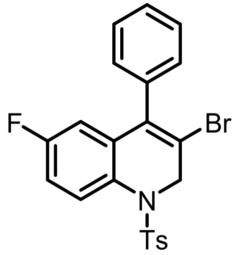

4b-Br (^{13}C NMR 101MHz, $CDCl_3$)

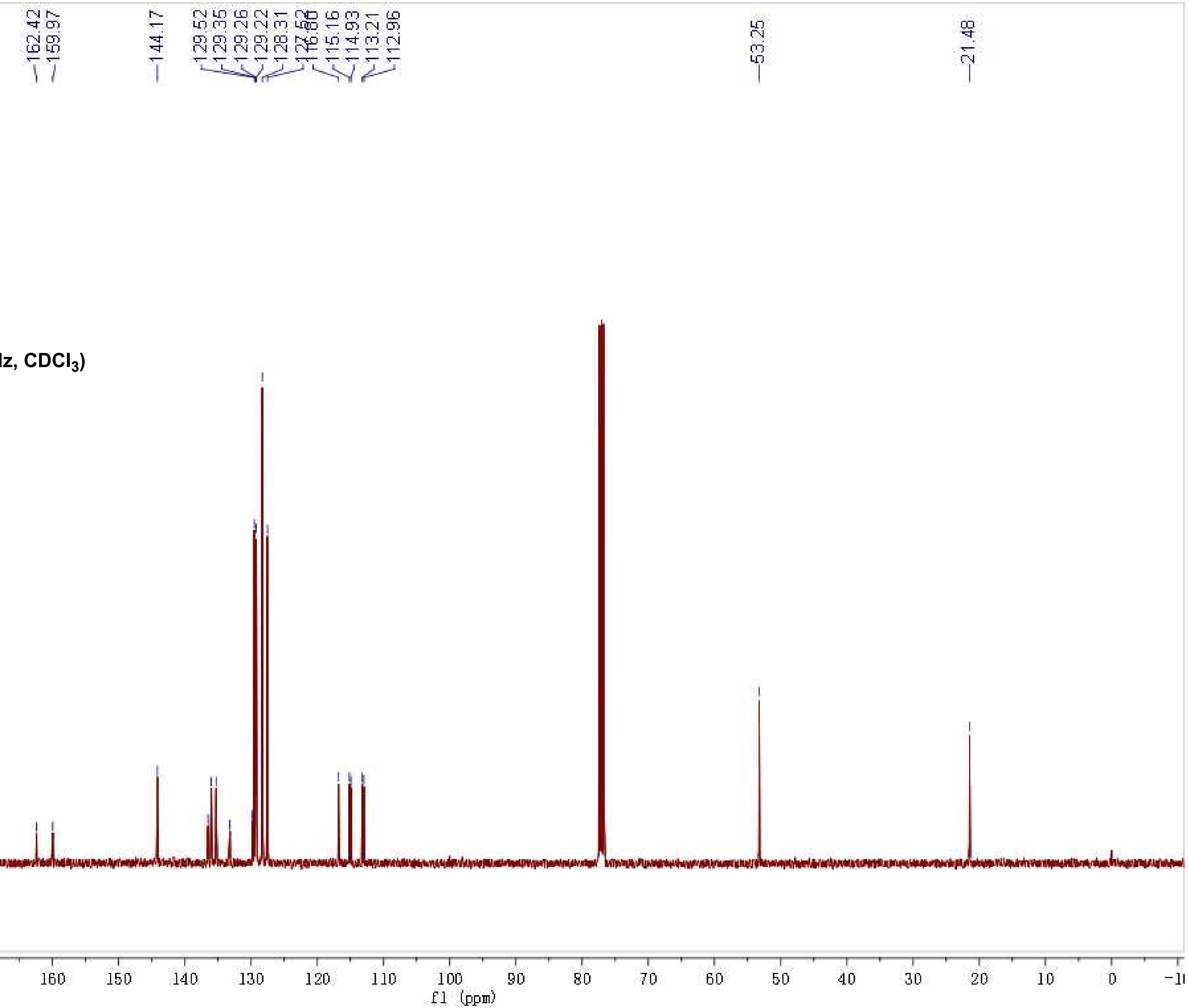

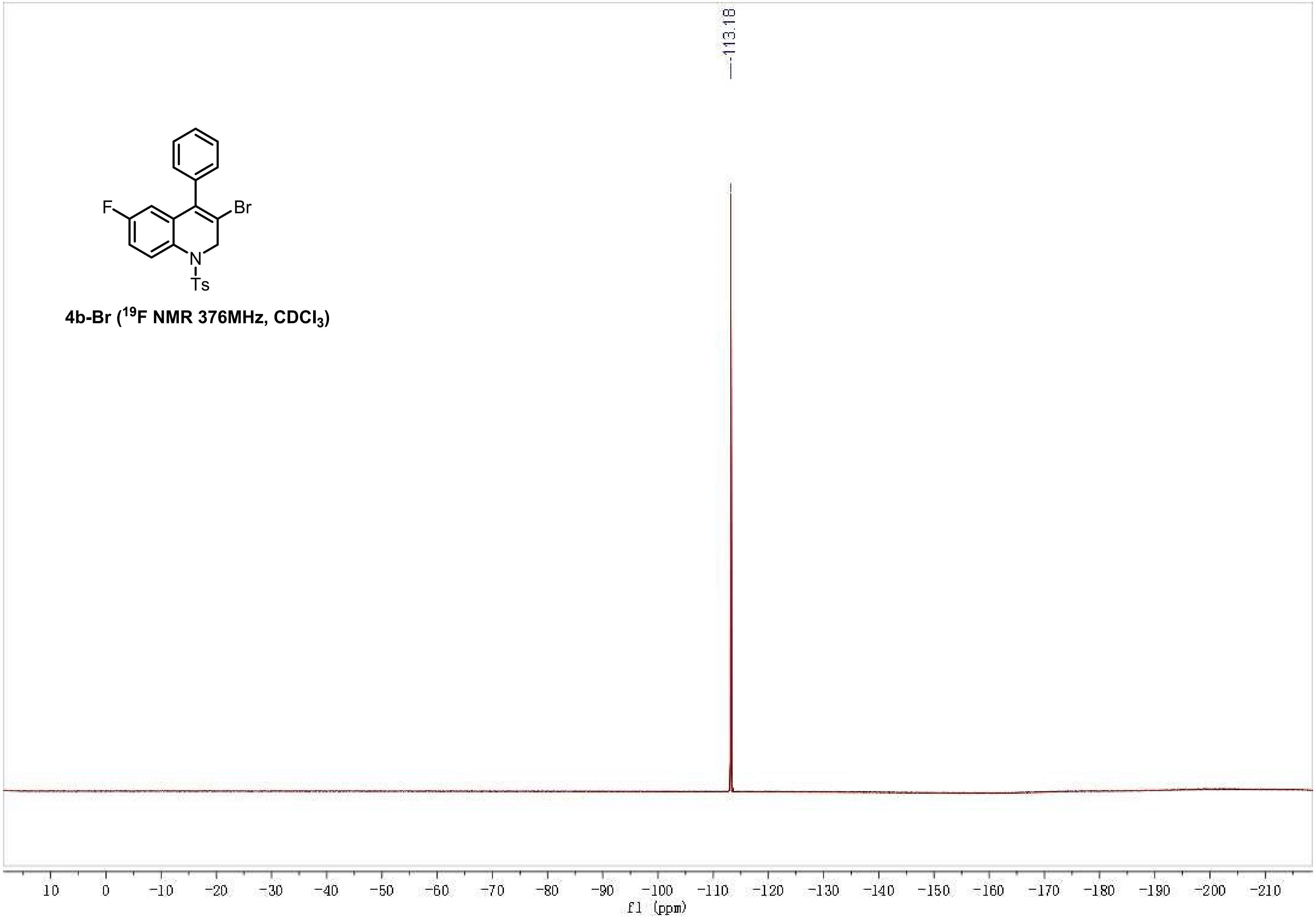

4b-Br (^{19}F NMR 376MHz, $CDCl_3$)

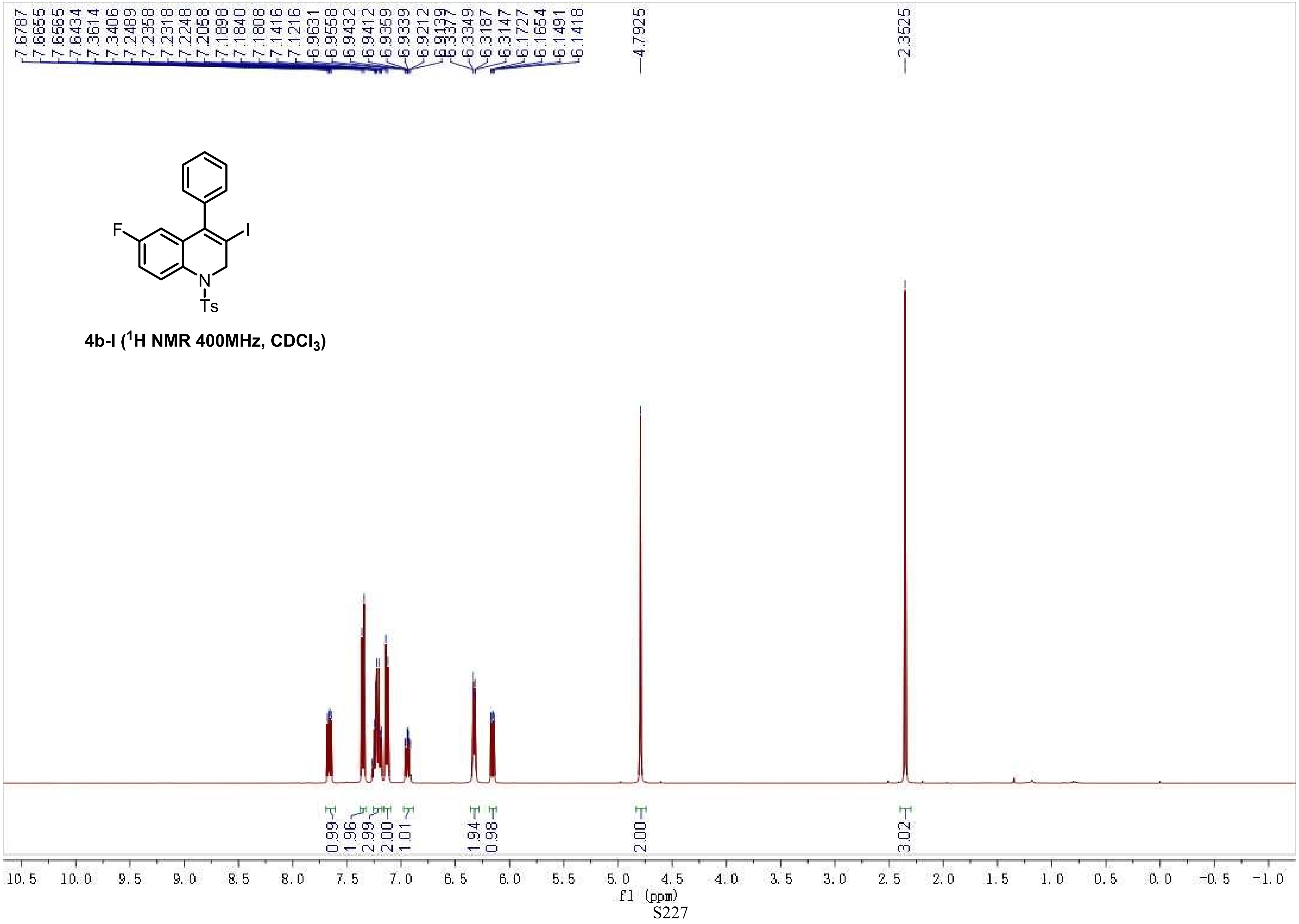
7.6787
7.6655
7.6565
7.6434
7.3614
7.3406
7.2489
7.2358
7.2318
7.2248
7.2058
7.1898
7.1840
7.1808
7.1416
7.1216
6.9631
6.9558
6.9432
6.9412
6.9359
6.9339
6.9212
6.9139
6.3377
6.3349
6.3187
6.3147
6.1727
6.1654
6.1491
6.1418
4.7925
2.3525
F
I
N
Ts
4b-I (^{1}H NMR 400MHz, $CDCl_3$)
0.99
1.96
2.99
2.00
1.01
1.94
0.98
2.00
3.02
10.5 10.0 9.5 9.0 8.5 8.0 7.5 7.0 6.5 6.0 5.5 5.0 4.5 4.0 3.5 3.0 2.5 2.0 1.5 1.0 0.5 0.0 -0.5 -1.0
f1 (ppm)

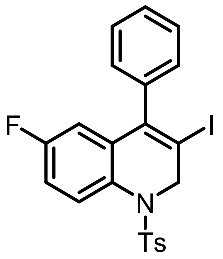

4b-I (^{13}C NMR 101MHz, $CDCl_3$)

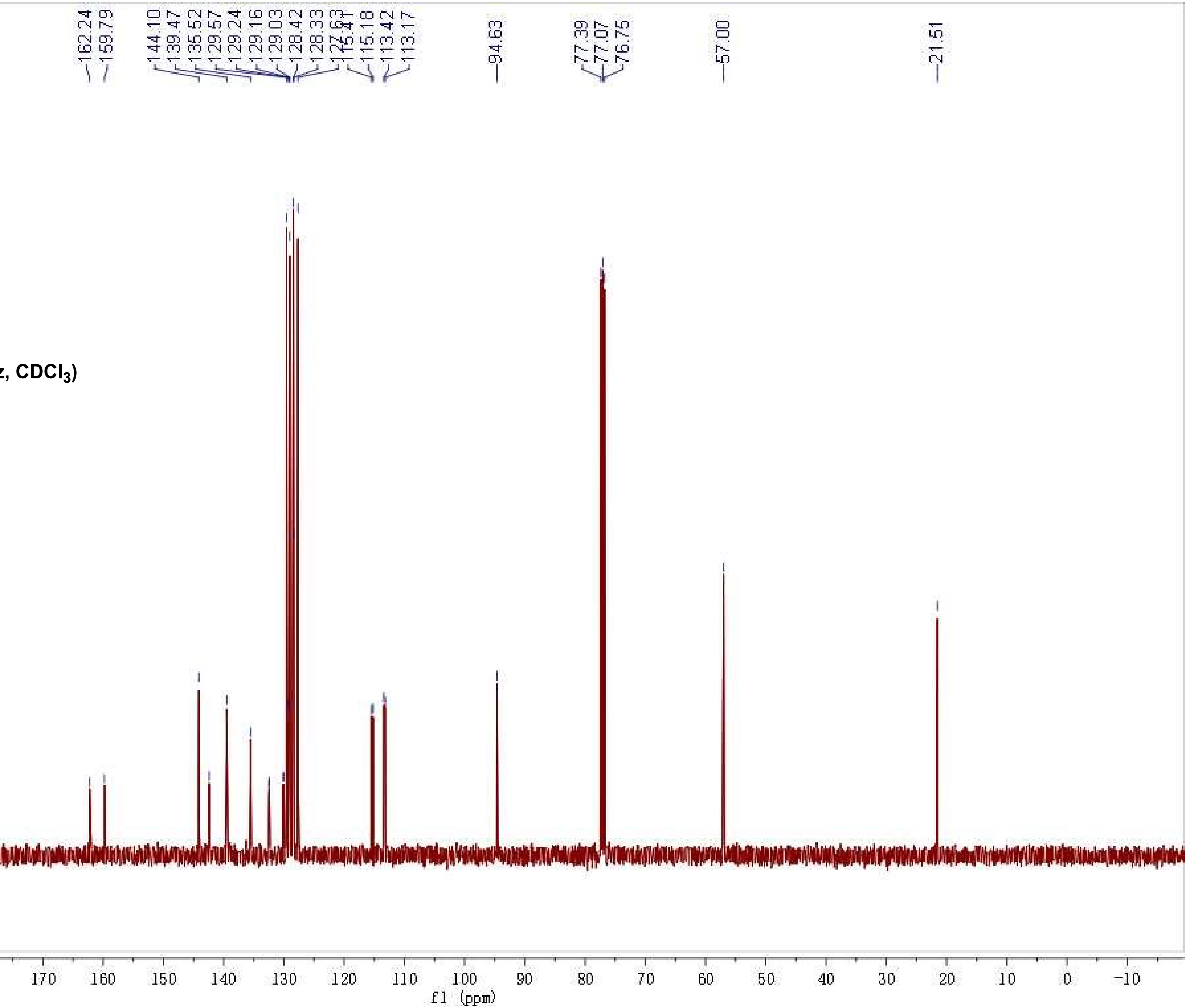

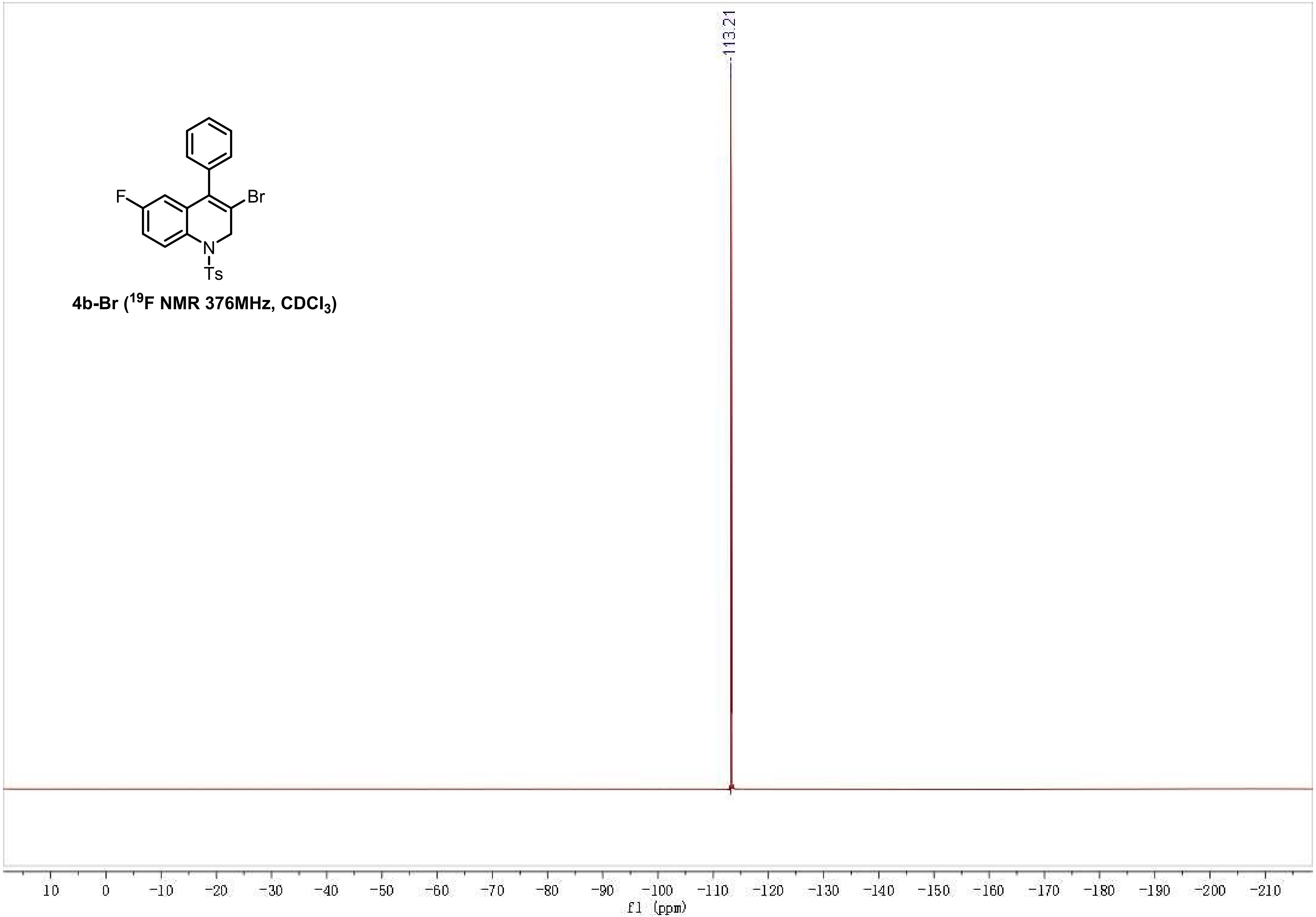

4b-Br (^{19}F NMR 376MHz, $CDCl_3$)

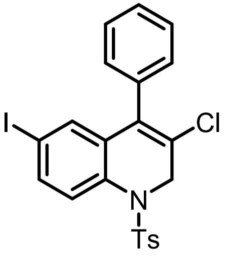

4c-Cl (^{1}H NMR 400MHz, $CDCl_3$)

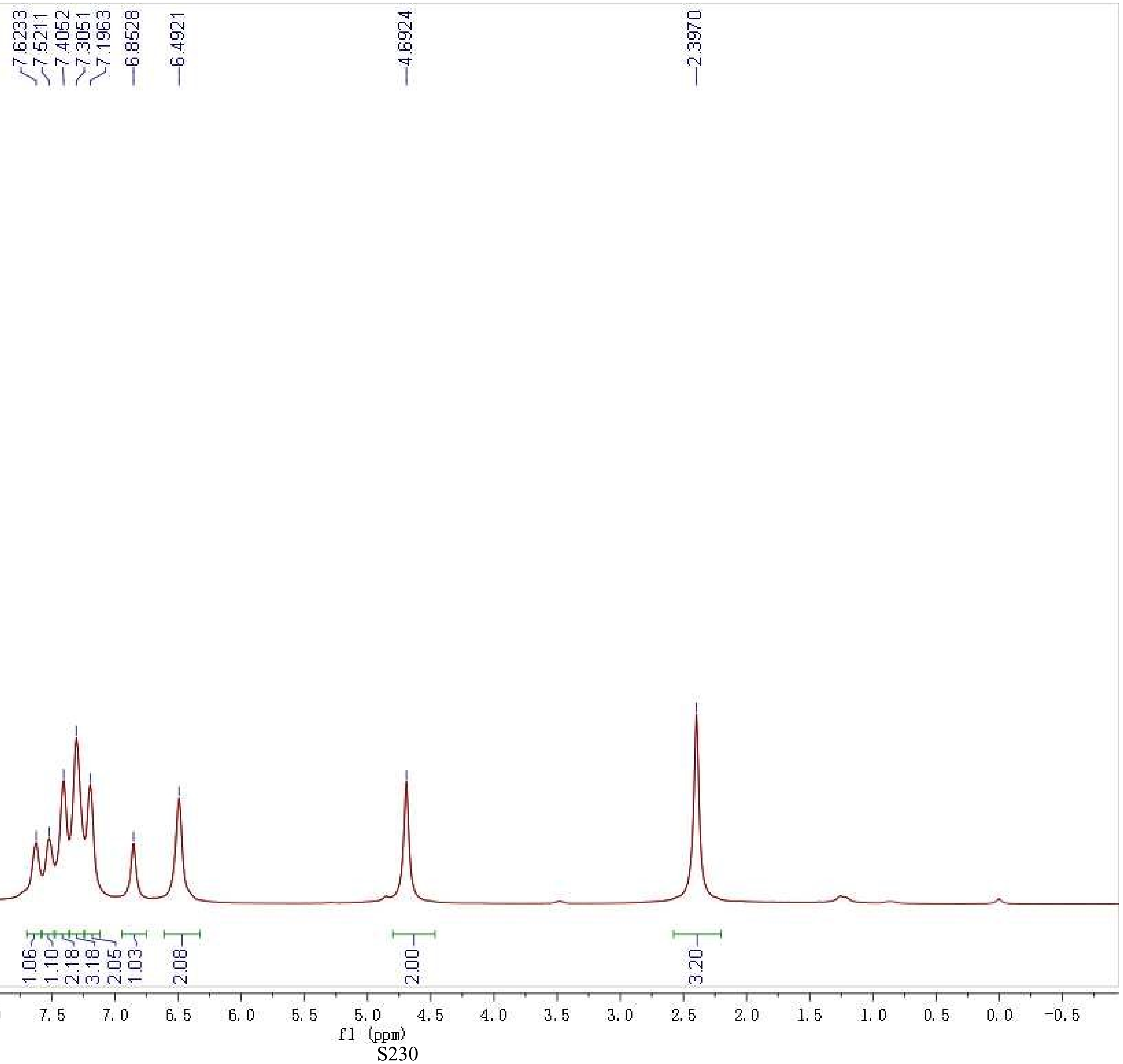

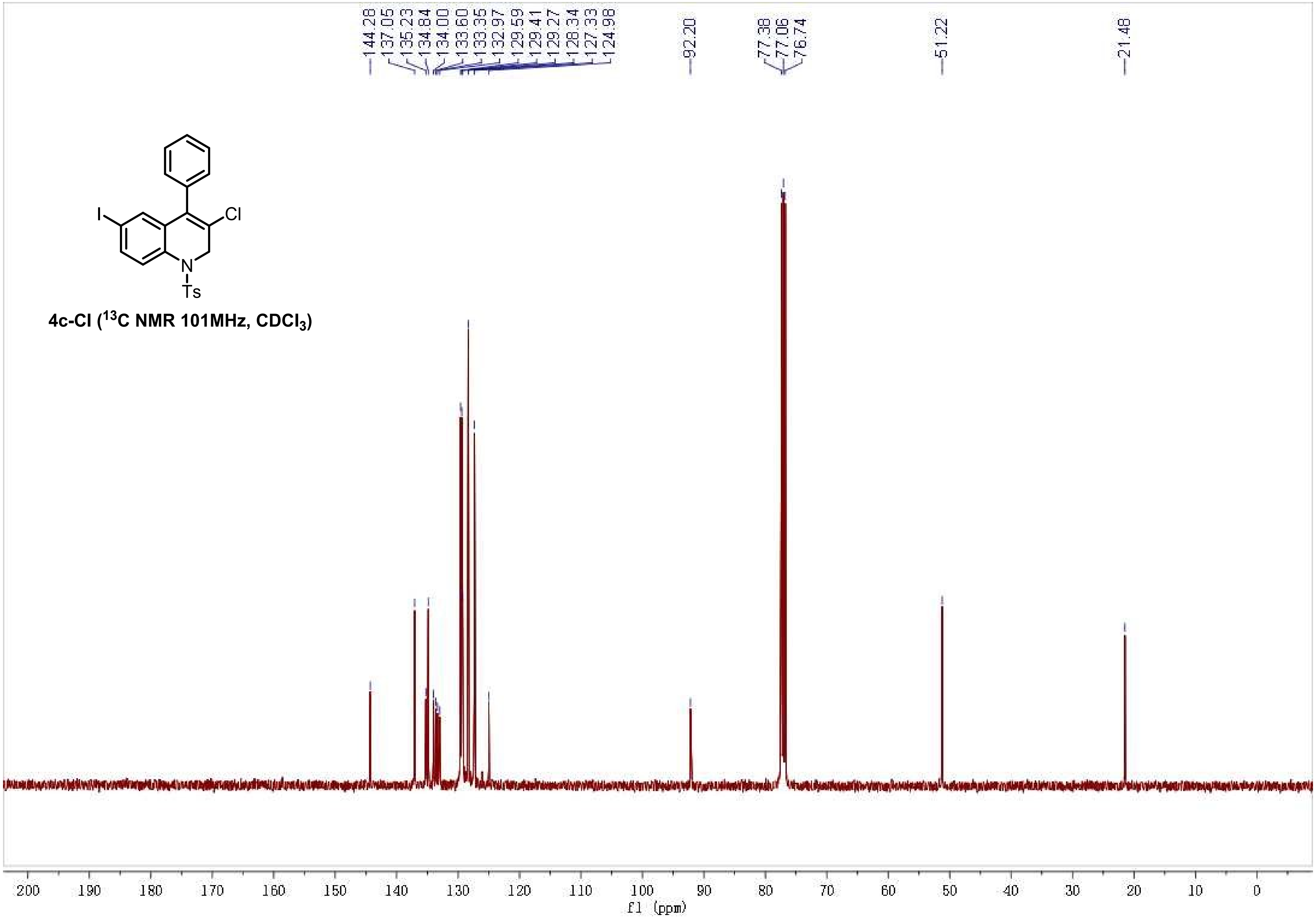
144.28
137.05
135.23
134.84
134.00
133.60
133.35
132.97
129.59
129.41
129.27
128.34
127.33
124.98
92.20
77.38
77.06
76.74
51.22
21.48
I
Cl
N
Ts
4c-Cl (^{13}C NMR 101MHz, $CDCl_3$)
200 190 180 170 160 150 140 130 120 110 100 90 80 70 60 50 40 30 20 10 0
f1 (ppm)

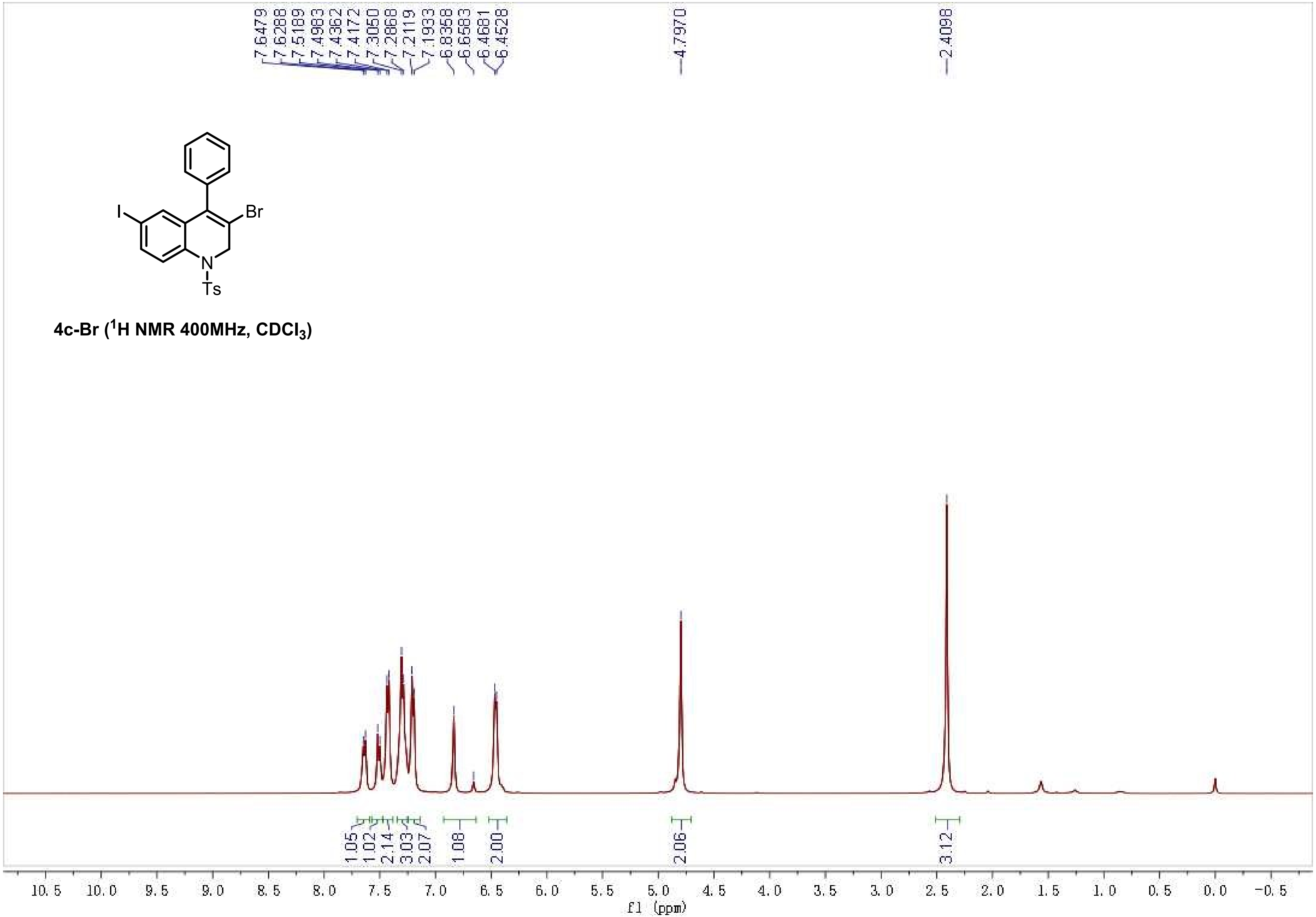

4c-Br (^{1}H NMR 400MHz, $CDCl_3$)

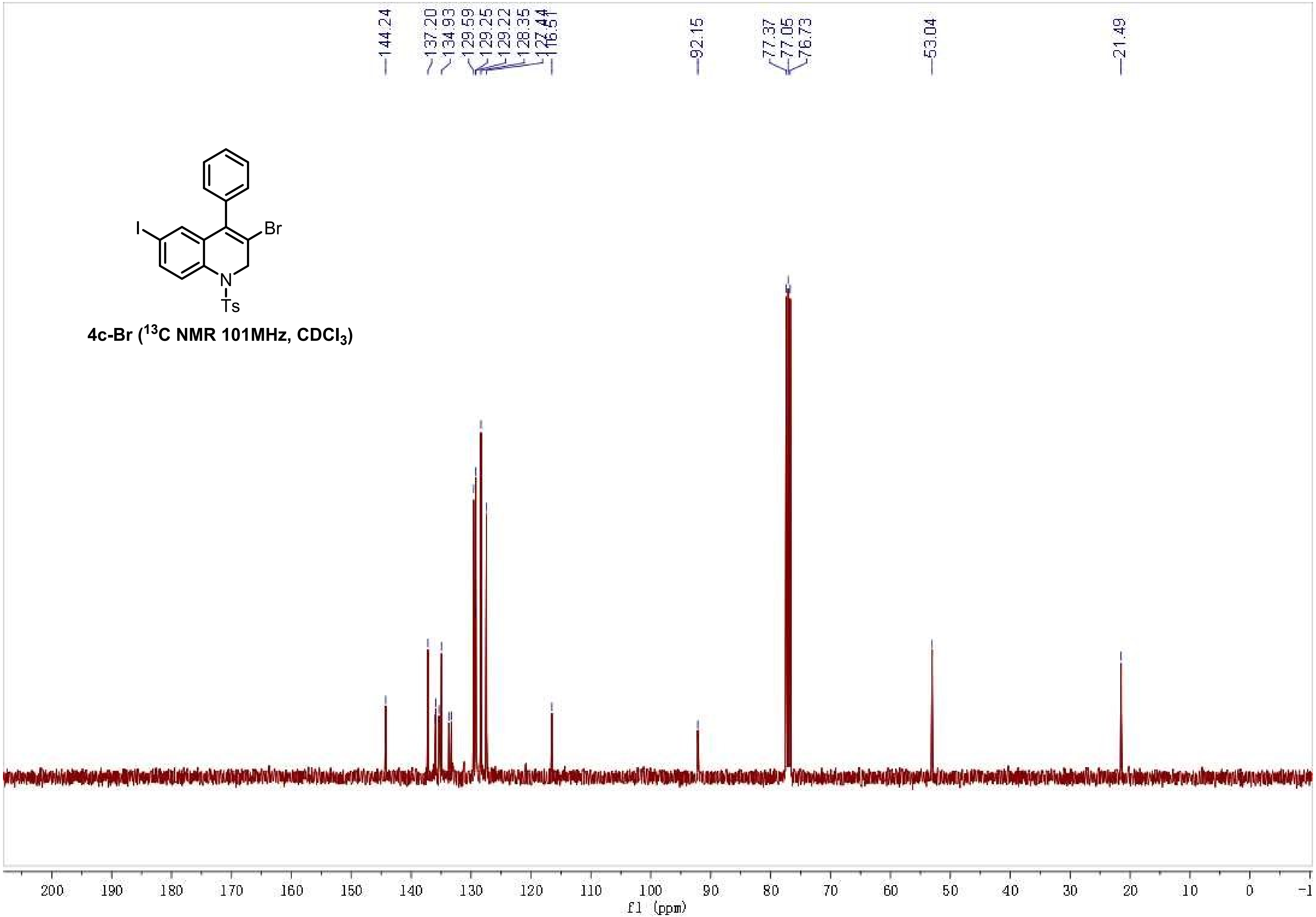

4c-Br (^{13}C NMR 101MHz, $CDCl_3$)

4c-I (^{1}H NMR 400MHz, $CDCl_3$)

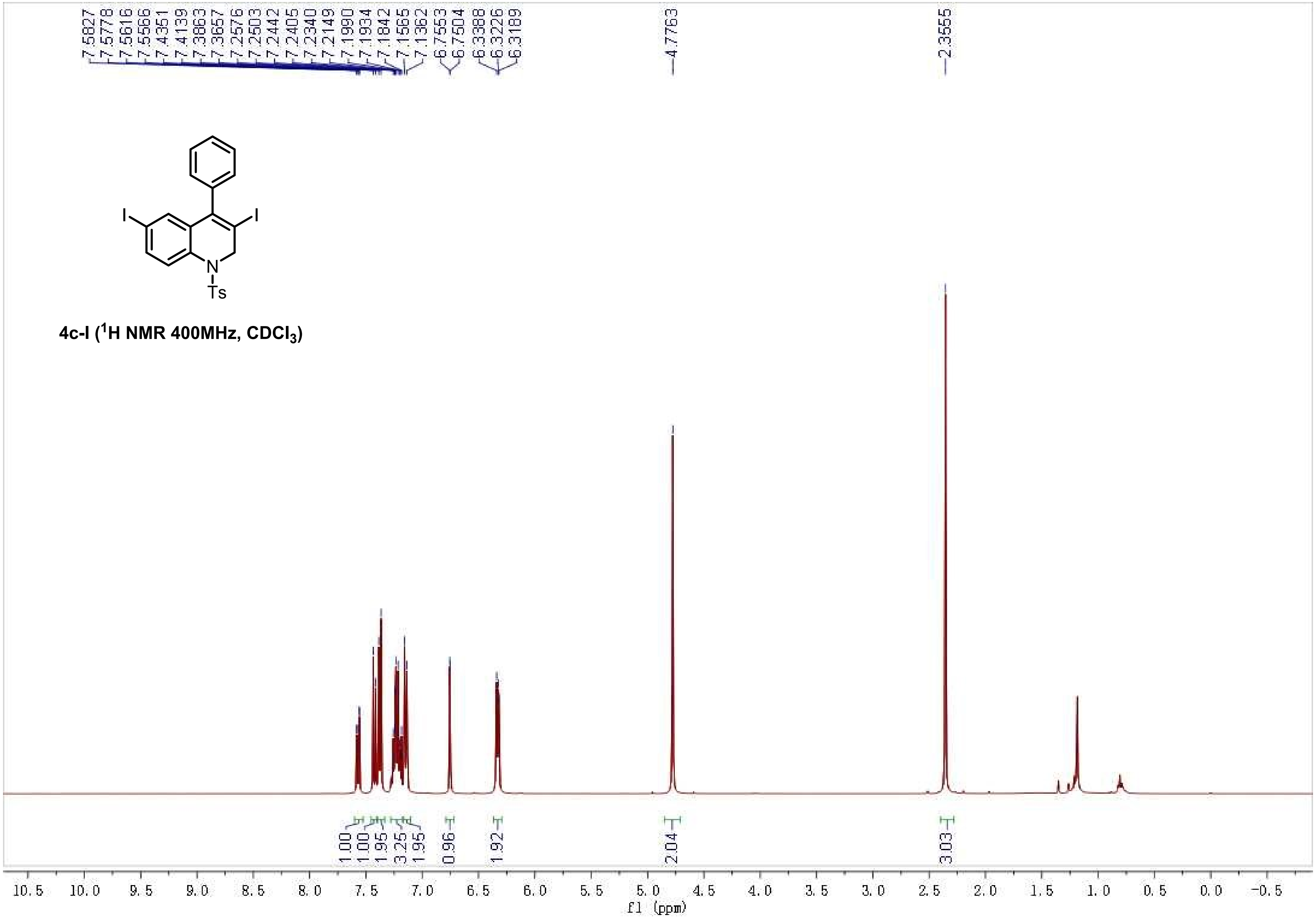

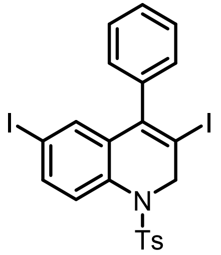

4c-I (^{13}C NMR 101MHz, $CDCl_3$)

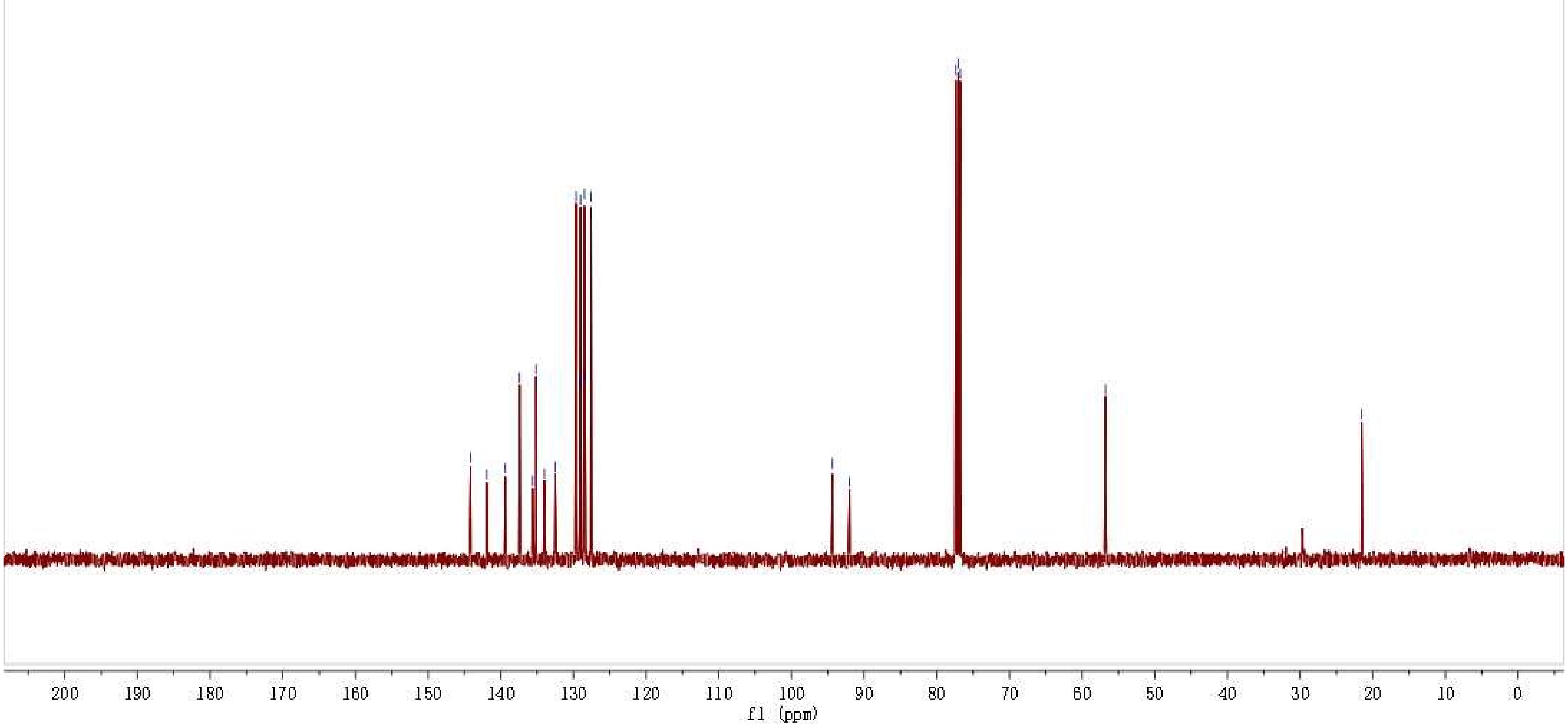

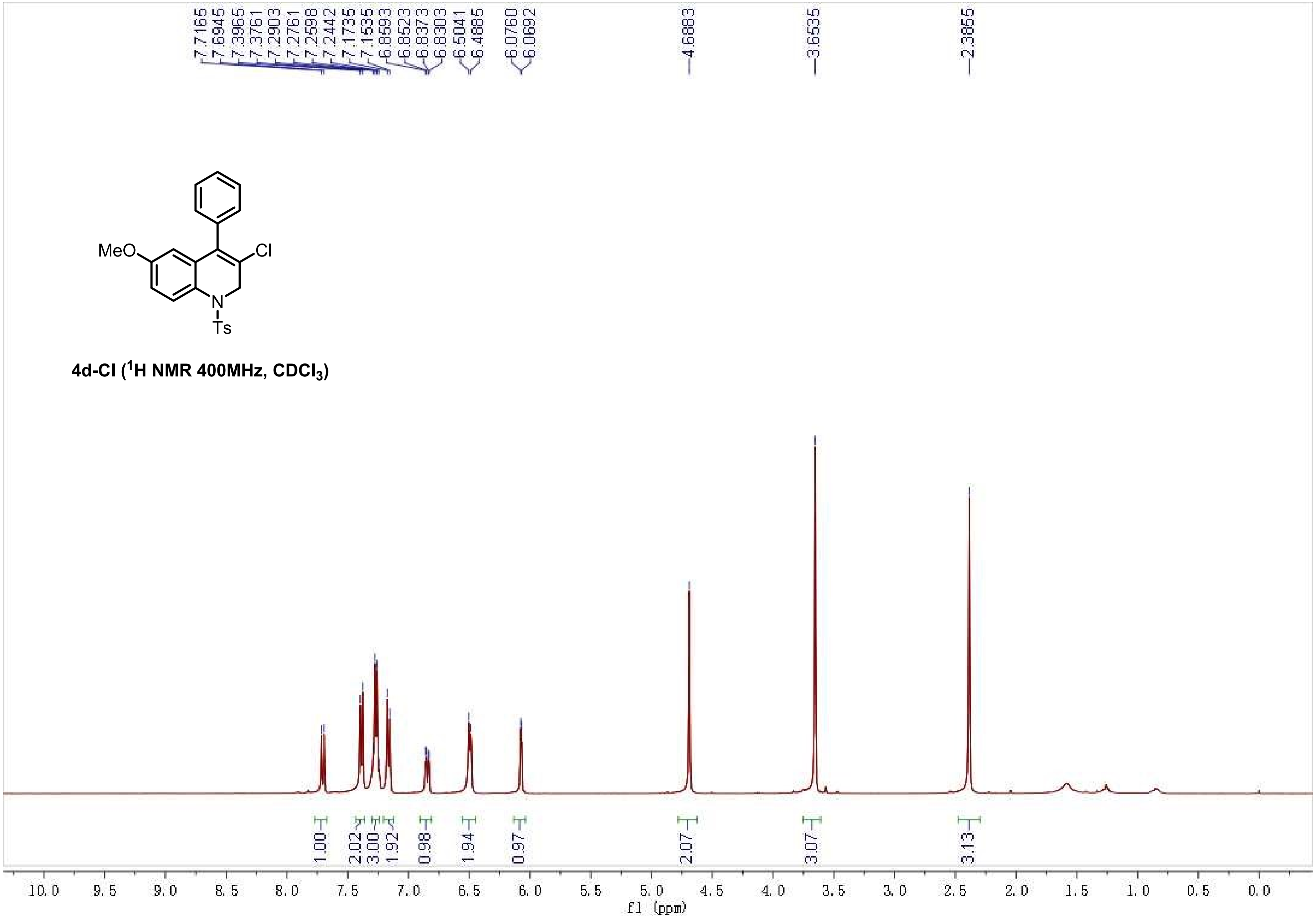

4d-Cl (^{1}H NMR 400MHz, $CDCl_3$)

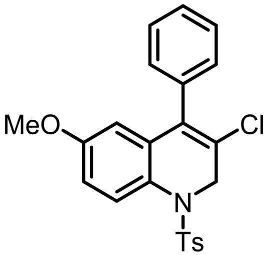

4d-Cl (^{13}C NMR 101MHz, $CDCl_3$)

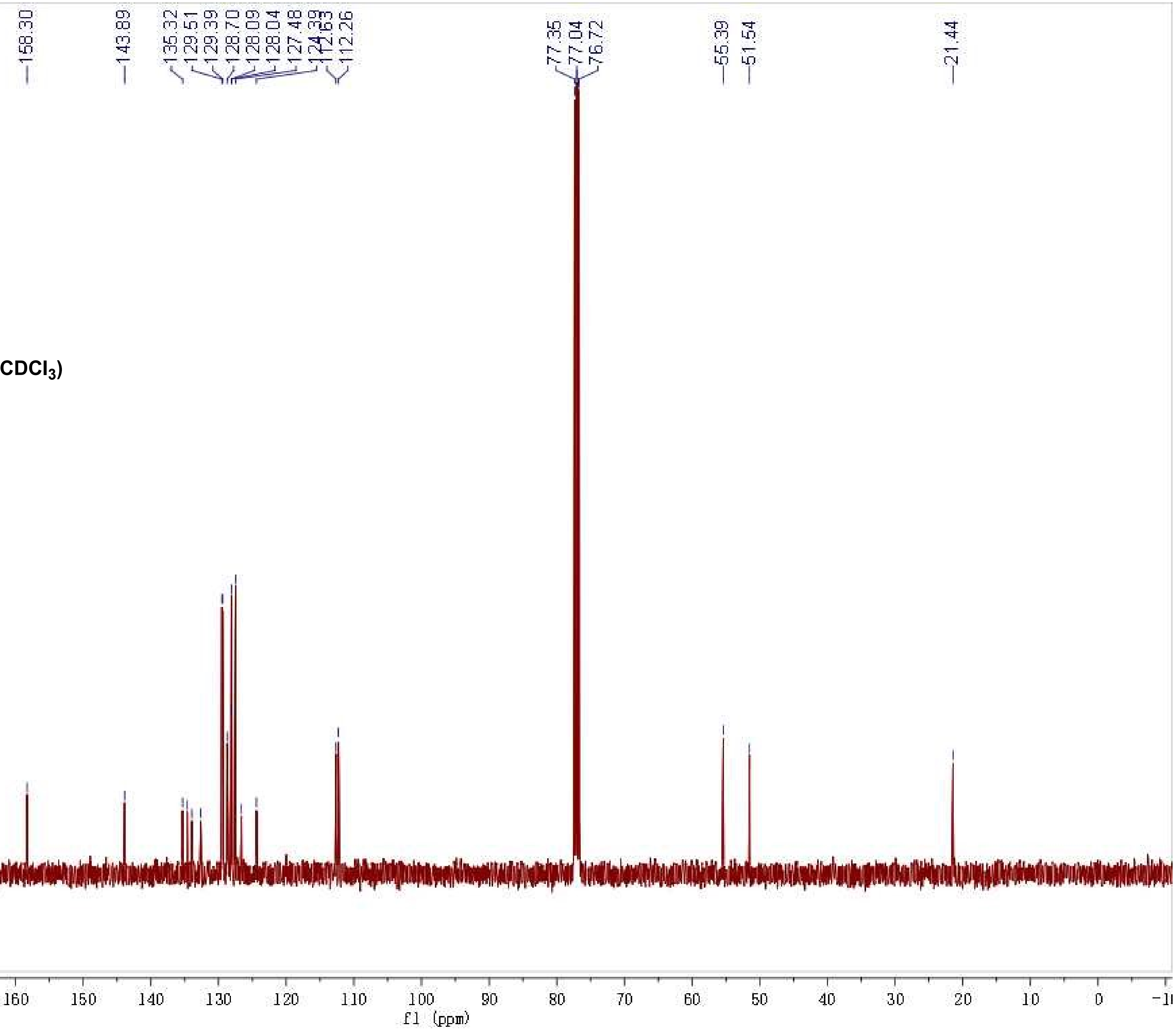

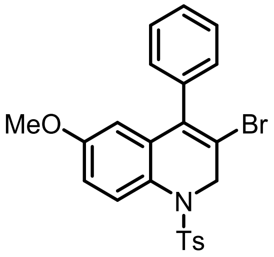

4d-Br (^{1}H NMR 400MHz, $CDCl_3$)

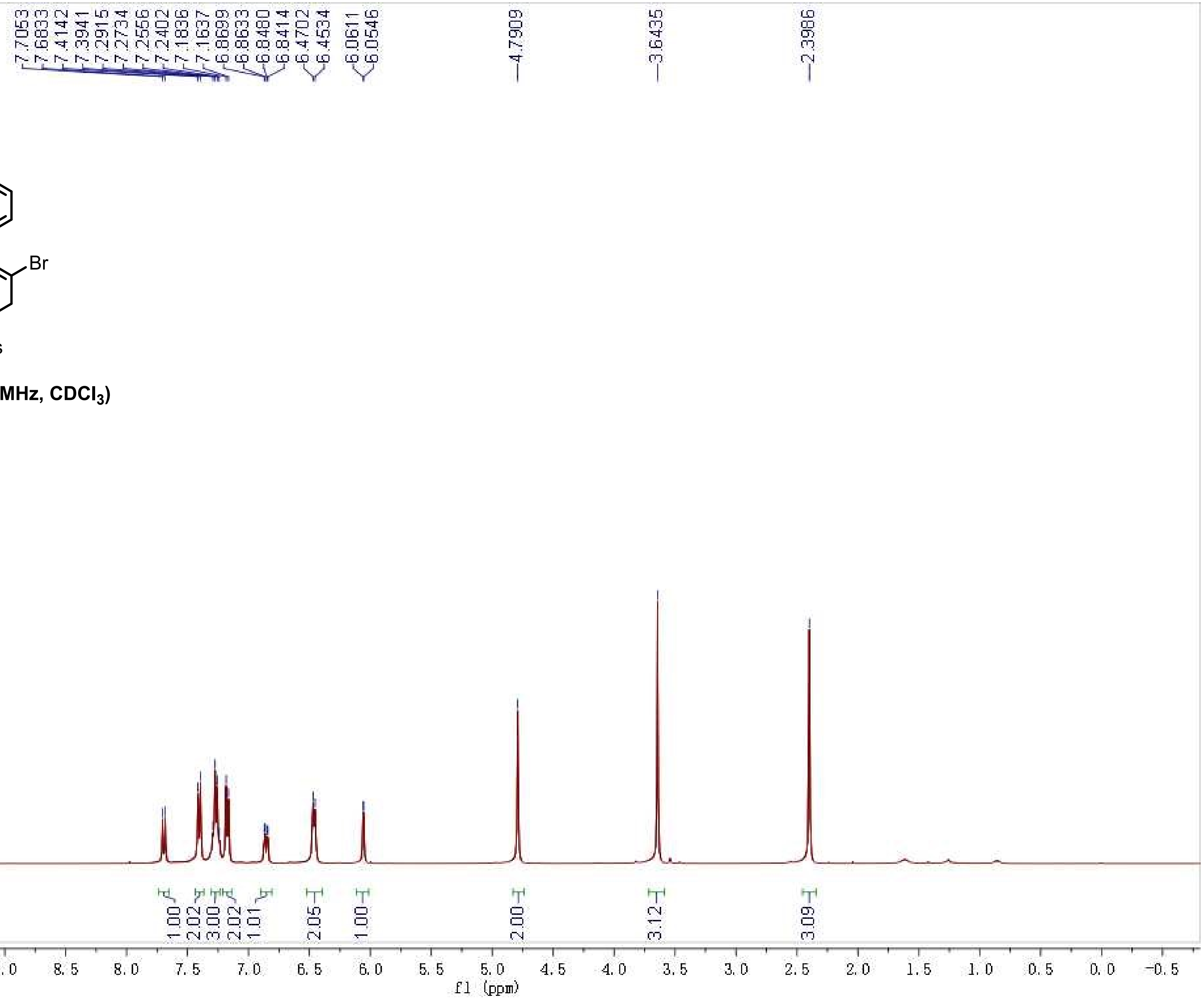

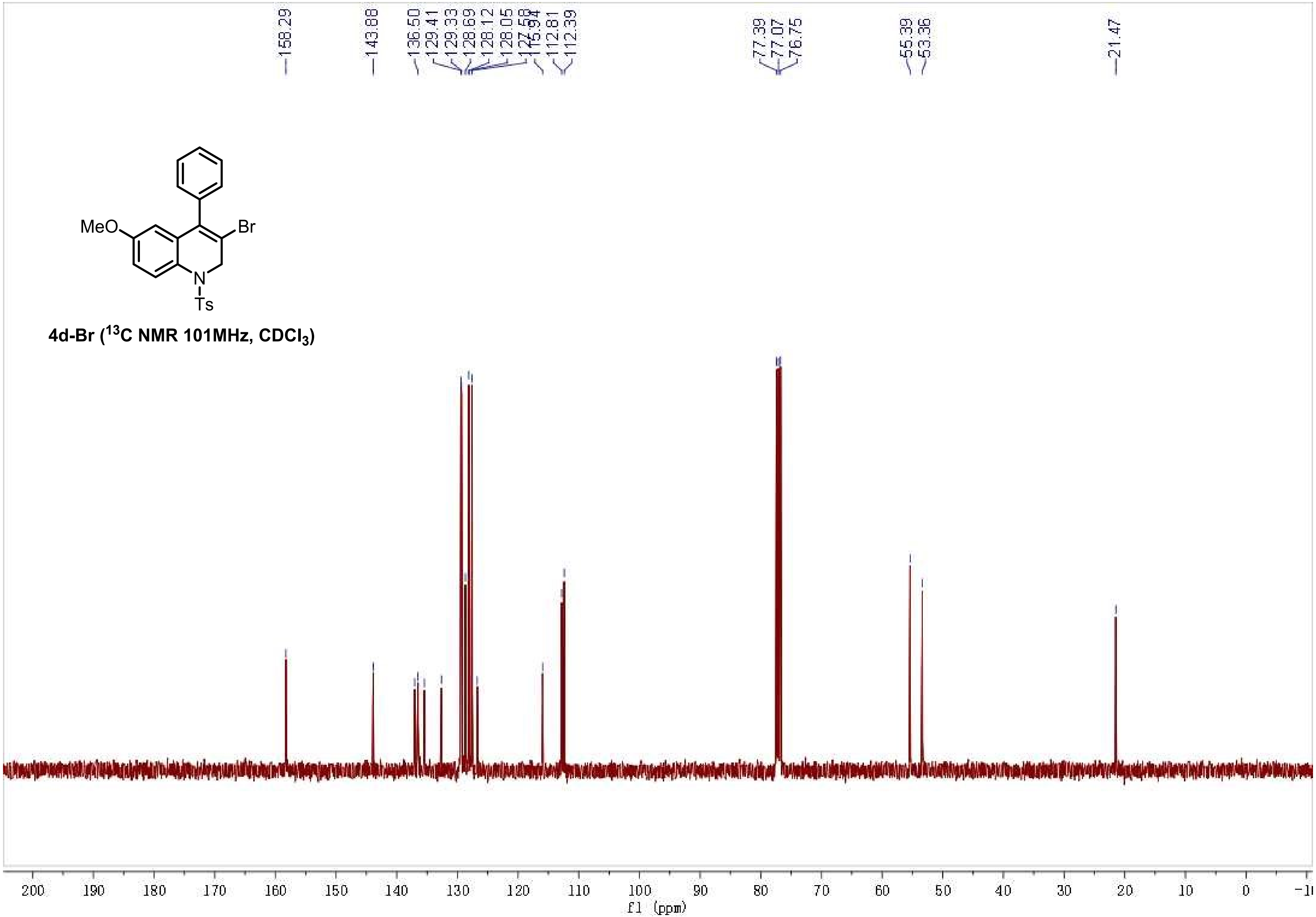
158.29
143.88
136.50
129.41
129.33
128.69
128.12
128.05
127.58
115.94
112.81
112.39
77.39
77.07
76.75
55.39
53.36
21.47
MeO
Br
N
Ts
4d-Br (^{13}C NMR 101MHz, CDCl$_3$)
200 190 180 170 160 150 140 130 120 110 100 90 80 70 60 50 40 30 20 10 0 -10
f1 (ppm)

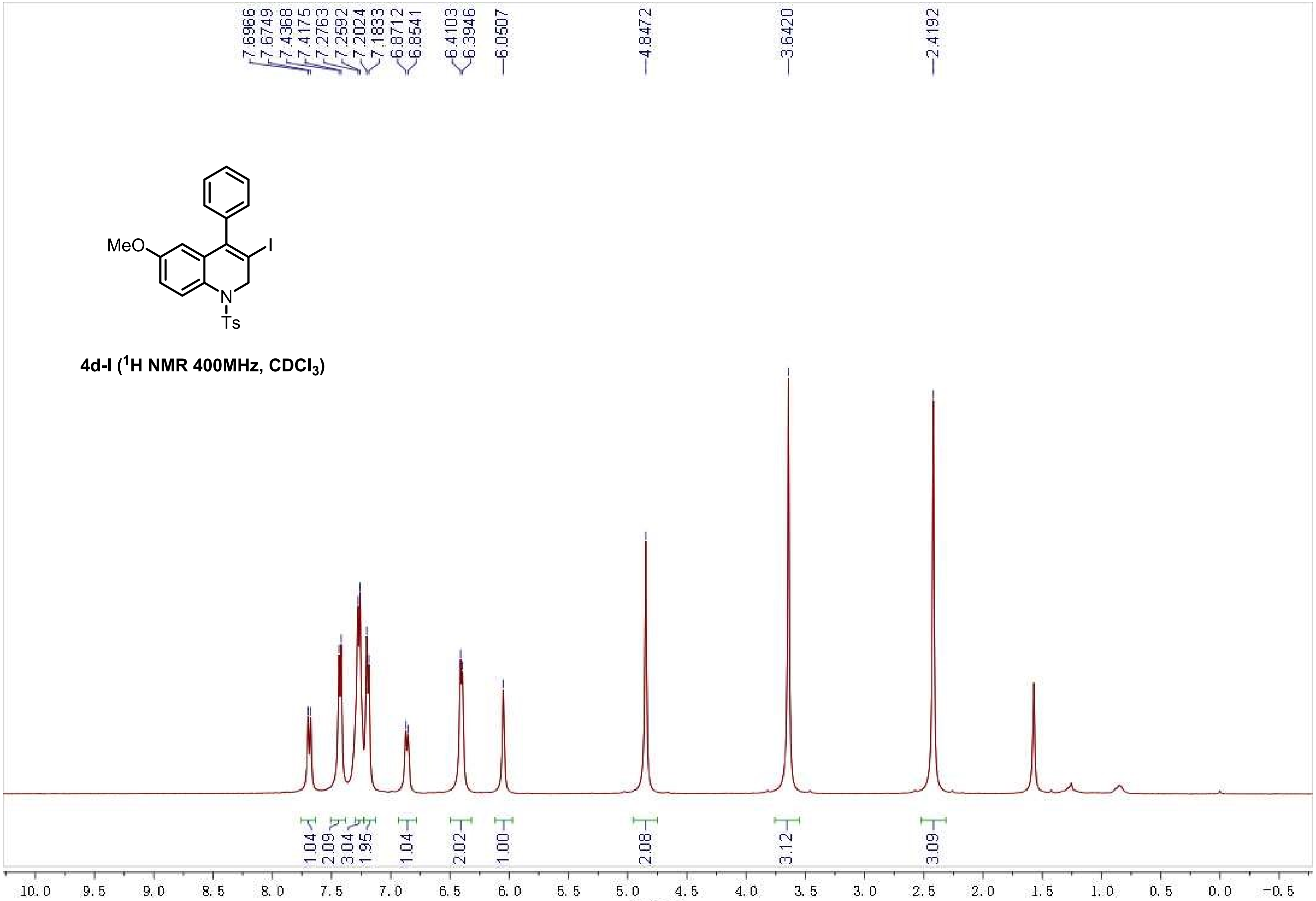

4d-I (^{1}H NMR 400MHz, $CDCl_3$)

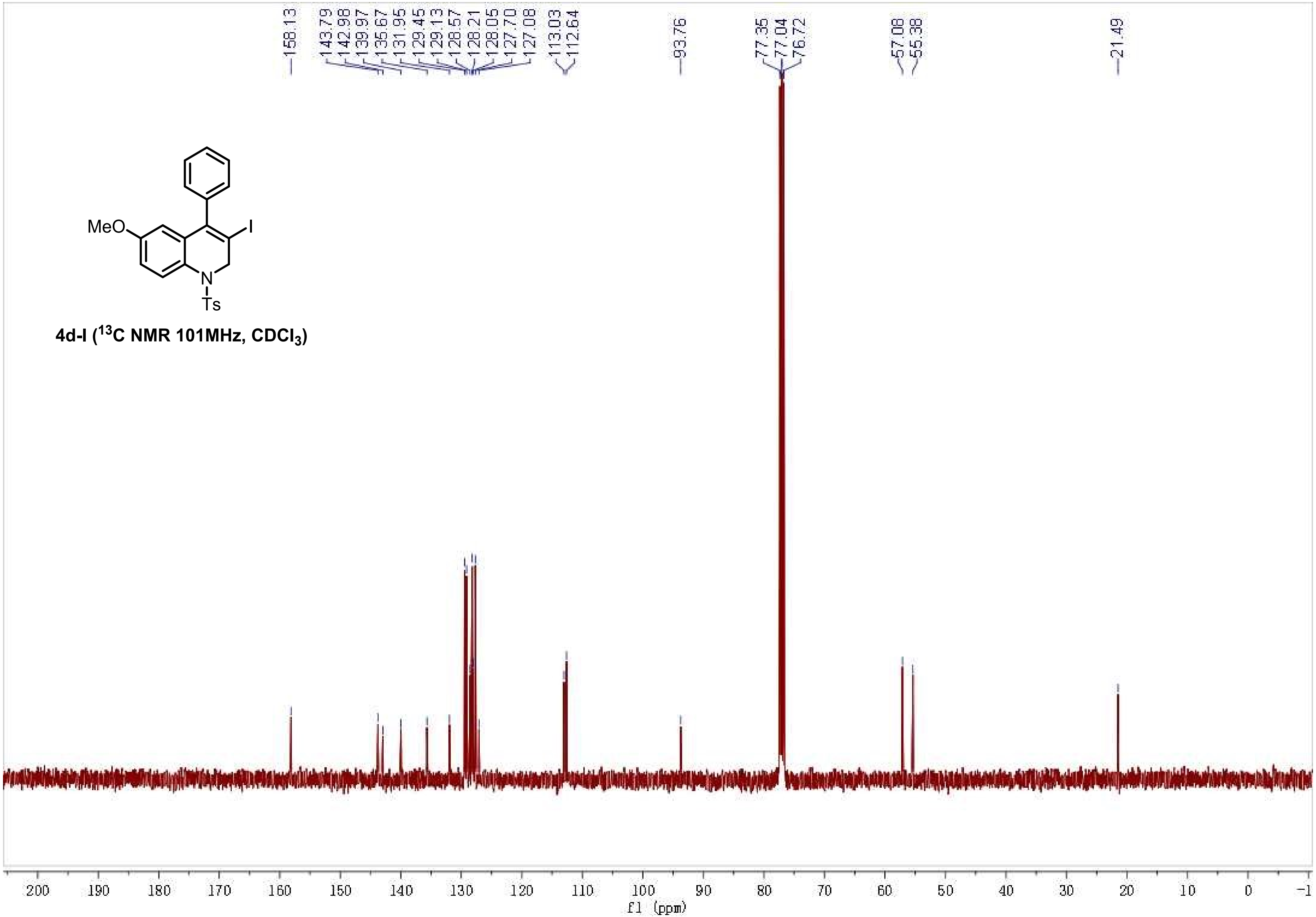

4d-I (^{13}C NMR 101MHz, $CDCl_3$)

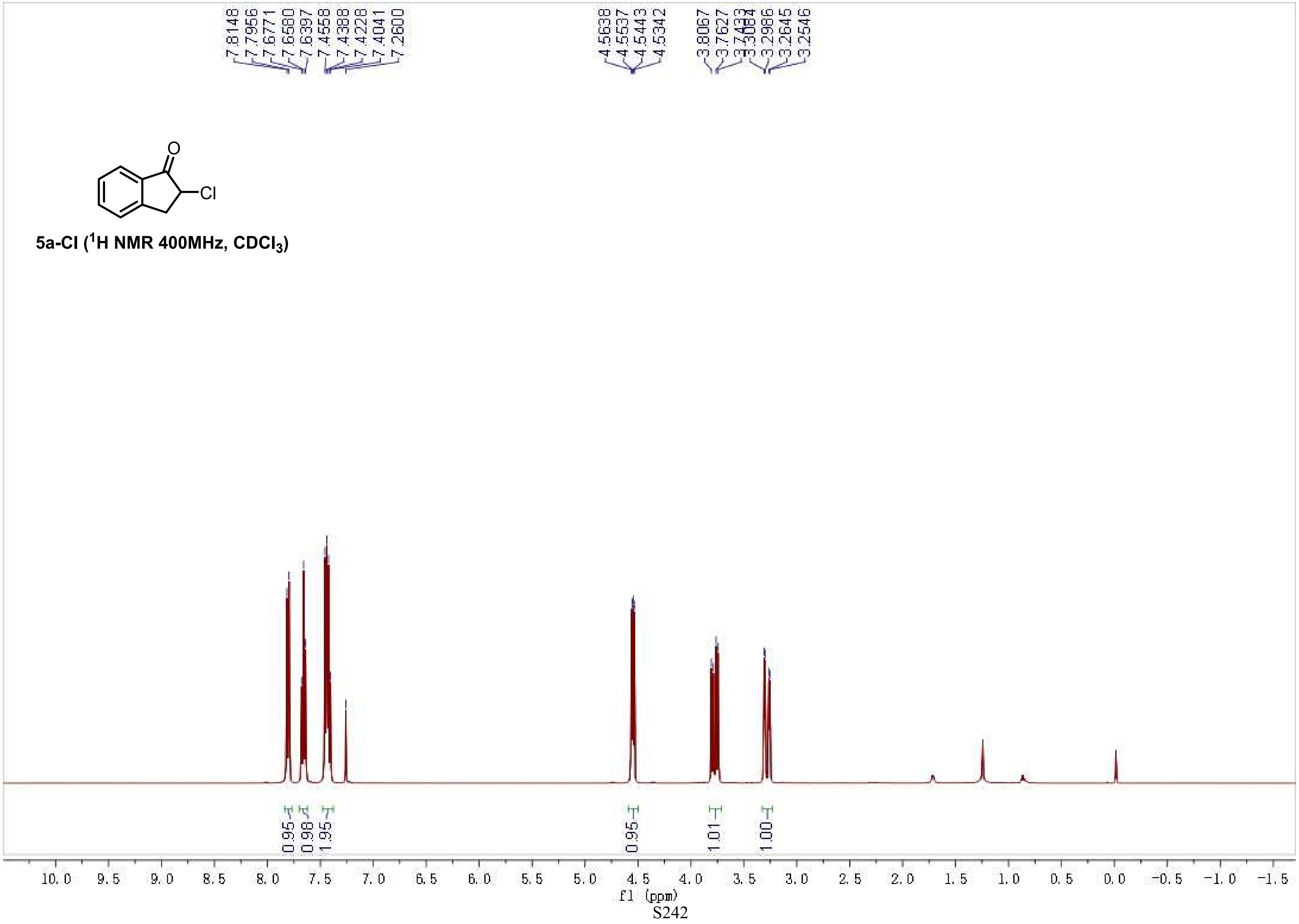

7.8148
7.7956
7.6771
7.6580
7.6397
7.4558
7.4388
7.4228
7.4041
7.2600
4.5638
4.5537
4.5443
4.5342
3.8067
3.7627
O
Cl
5a-Cl (^{1}H NMR 400MHz, $CDCl_3$)
0.95
0.98
1.95
0.95
1.01
1.00
10.0 9.5 9.0 8.5 8.0 7.5 7.0 6.5 6.0 5.5 5.0 4.5 4.0 3.5 3.0 2.5 2.0 1.5 1.0 0.5 0.0 -0.5 -1.0 -1.5
f1 (ppm)

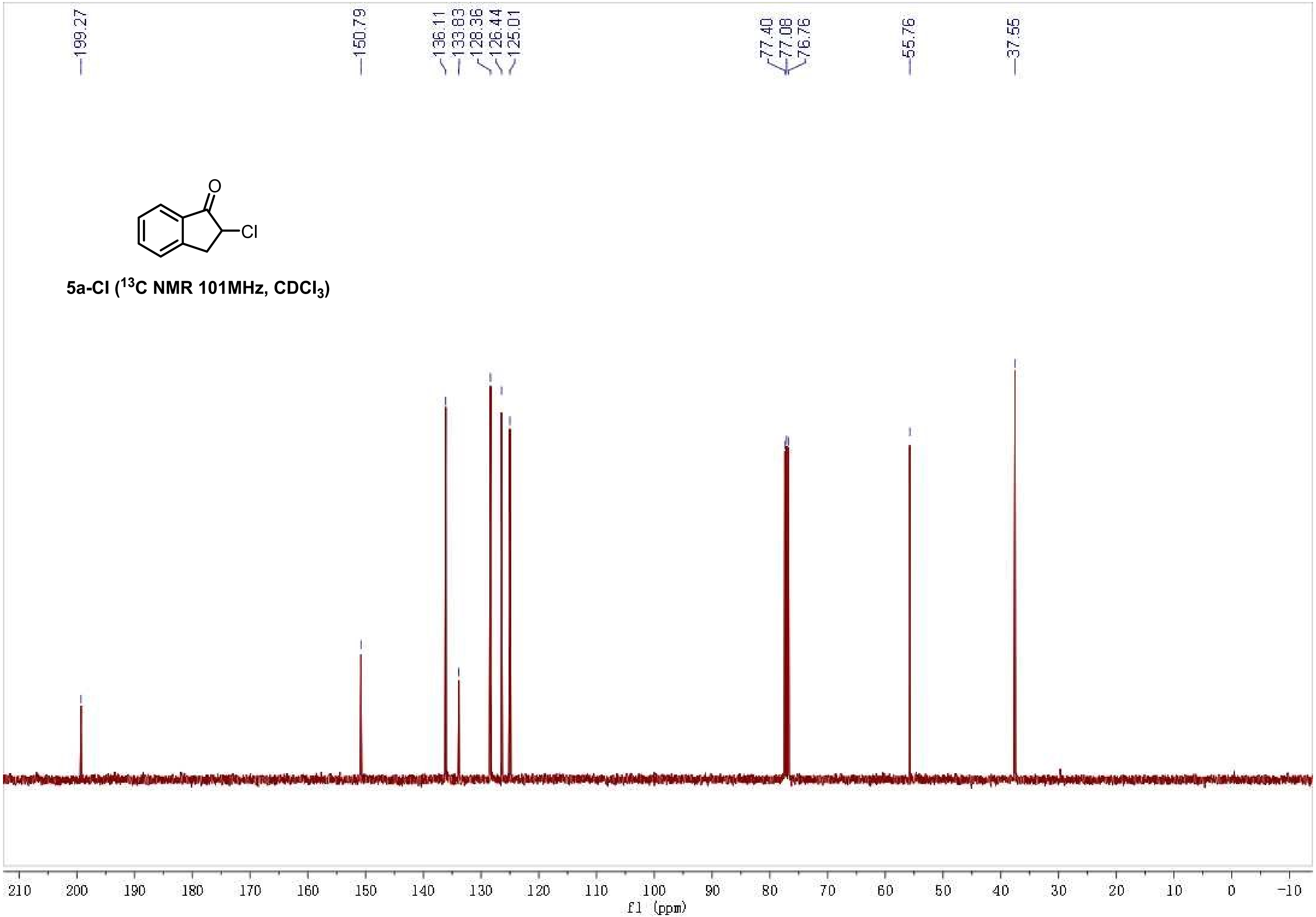

5a-Cl (^{13}C NMR 101MHz, $CDCl_3$)

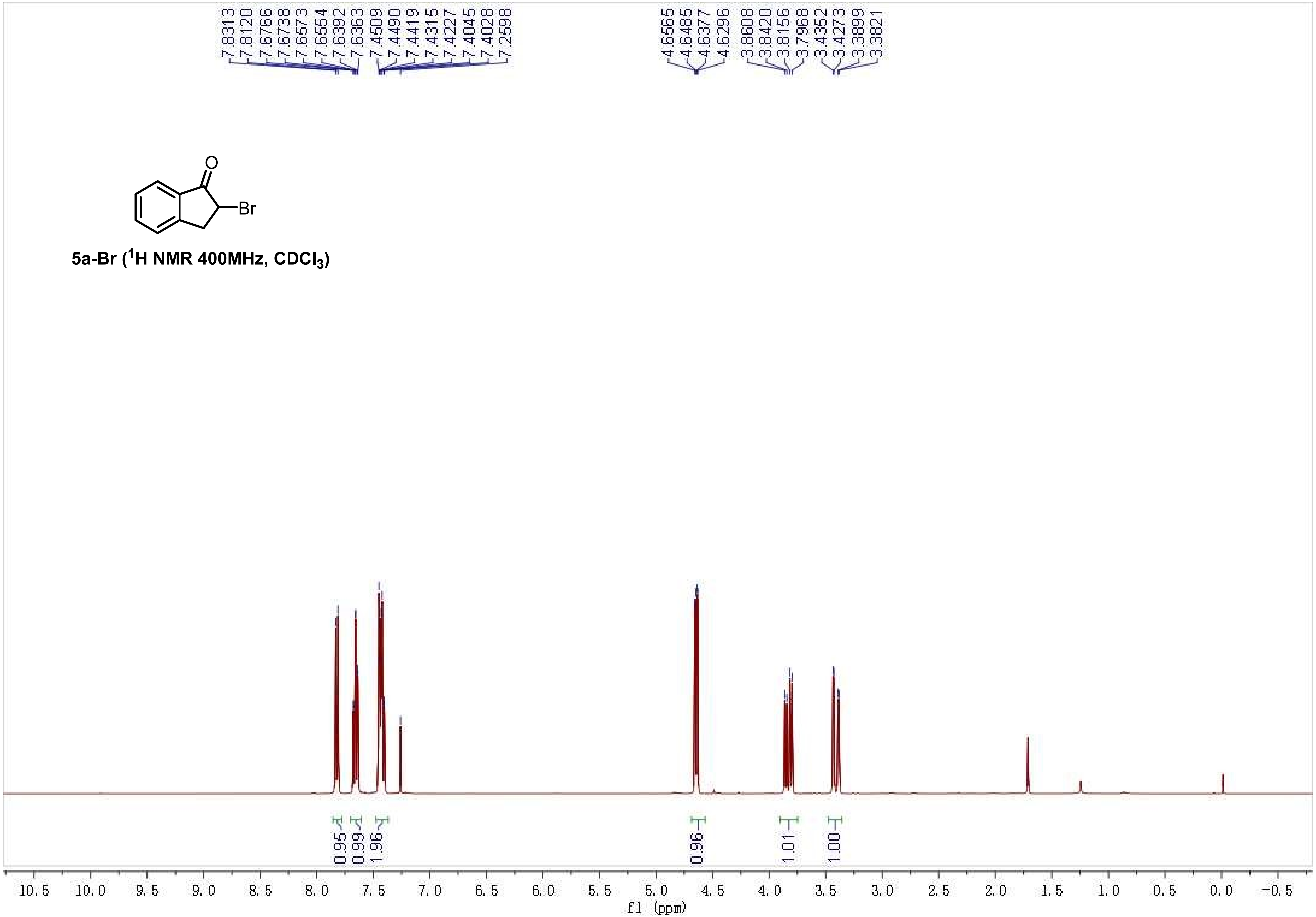
7.8313
7.8120
7.6766
7.6738
7.6573
7.6554
7.6392
7.6363
7.4509
7.4490
7.4419
7.4315
7.4227
7.4045
7.4028
7.2598
4.6565
4.6485
4.6377
4.6296
3.8608
3.8420
3.8156
3.7968
3.4352
3.4273
3.3899
3.3821
O
Br
5a-Br (1H NMR 400MHz, CDCl3)
0.95
0.99
1.96
0.96
1.01
1.00
10.5 10.0 9.5 9.0 8.5 8.0 7.5 7.0 6.5 6.0 5.5 5.0 4.5 4.0 3.5 3.0 2.5 2.0 1.5 1.0 0.5 0.0 -0.5
f1 (ppm)

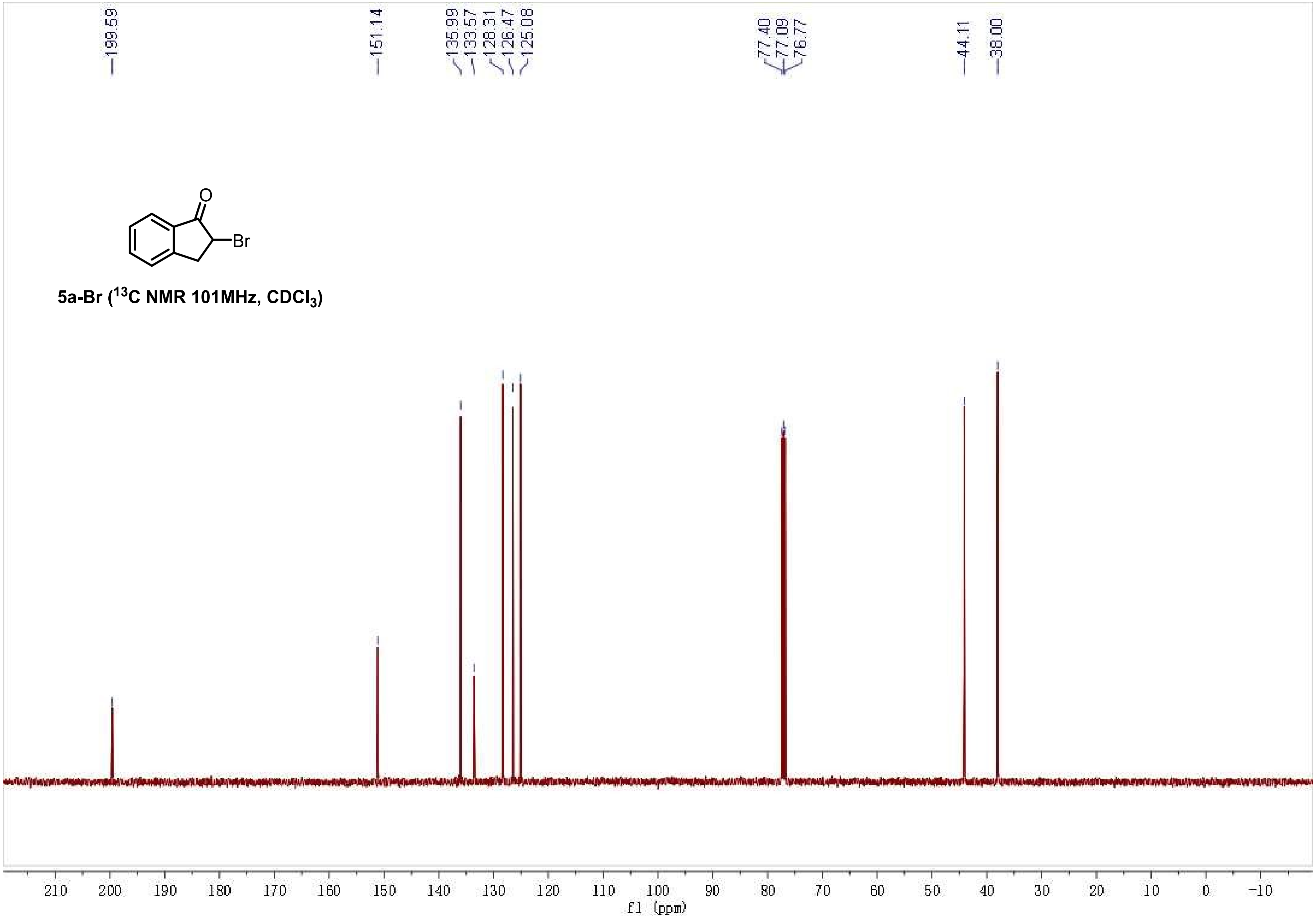

5a-Br (^{13}C NMR 101MHz, $CDCl_3$)

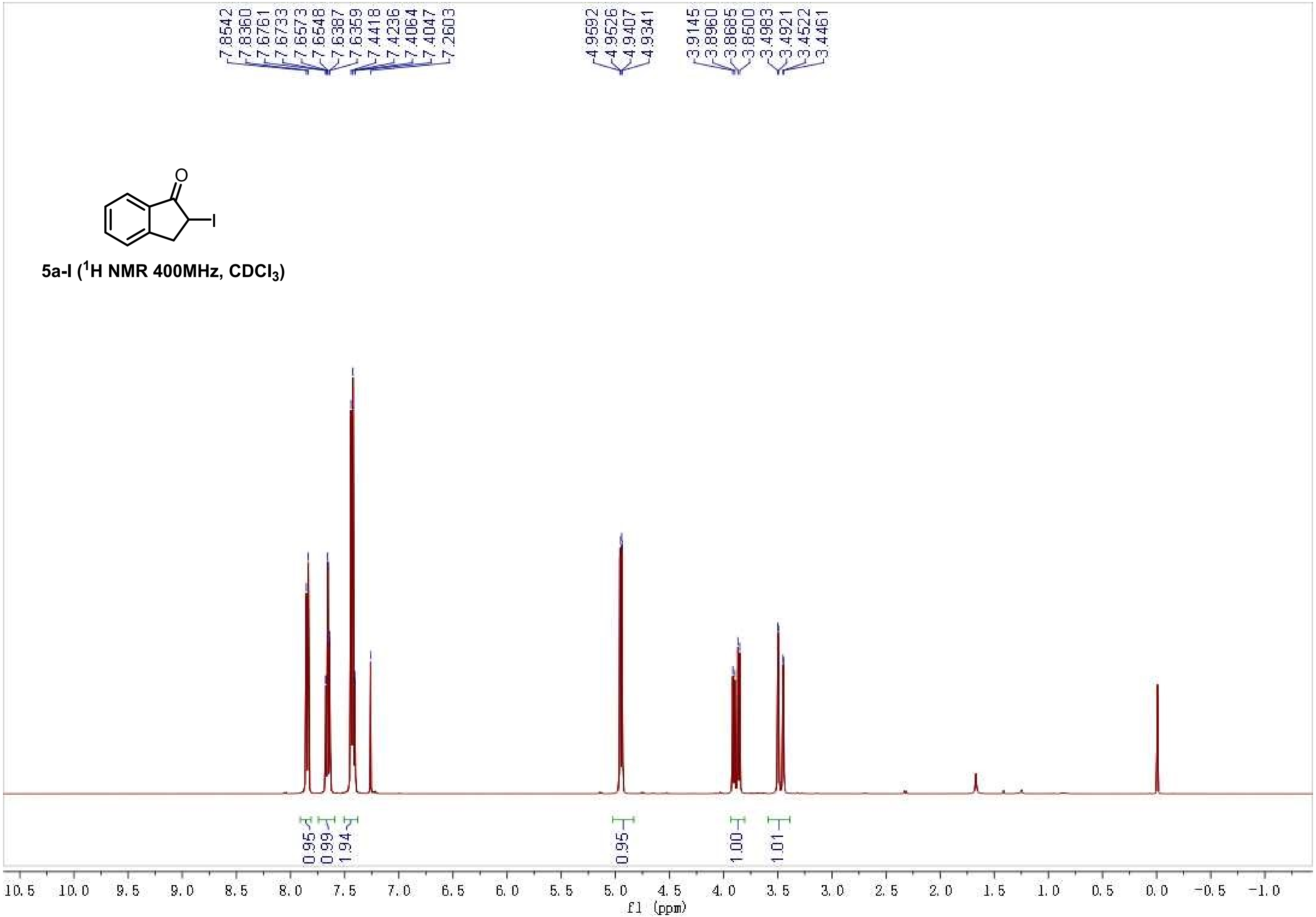
7.8542
7.8360
7.6761
7.6733
7.6573
7.6548
7.6387
7.6359
7.4418
7.4236
7.4064
7.4047
7.2603
4.9592
4.9526
4.9407
4.9341
3.9145
3.8960
3.8685
3.8500
3.4983
3.4921
3.4522
3.4461
O
I
5a-I (^{1}H NMR 400MHz, $CDCl_3$)
0.95
0.99
1.94
0.95
1.00
1.01
10.5
10.0
9.5
9.0
8.5
8.0
7.5
7.0
6.5
6.0
5.5
5.0
4.5
4.0
3.5
3.0
2.5
2.0
1.5
1.0
0.5
0.0
-0.5
-1.0
f1 (ppm)

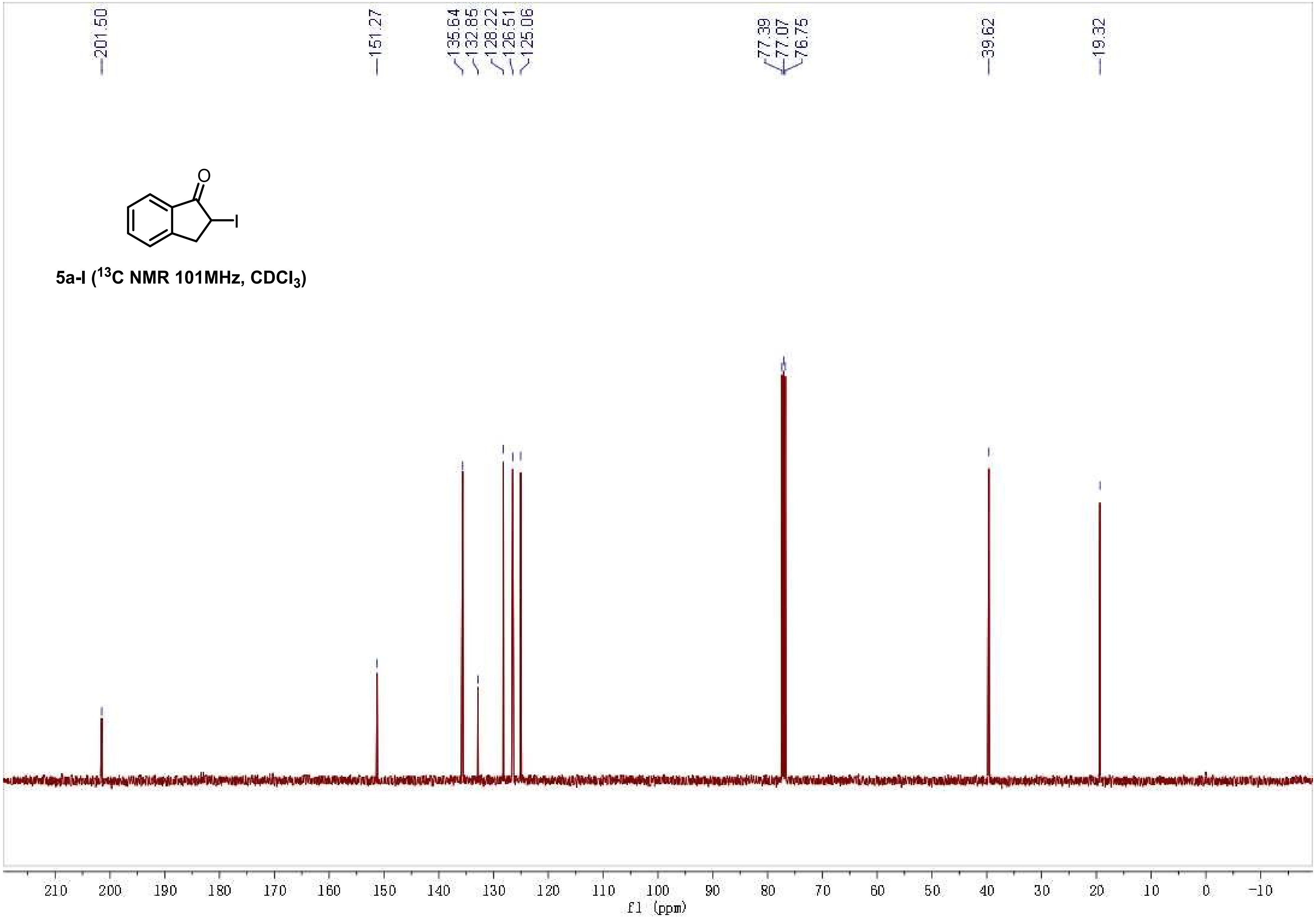
201.50
151.27
135.64
132.85
128.22
126.51
125.06
77.39
77.07
76.75
39.62
19.32
O
I
5a-I (^{13}C NMR 101MHz, $CDCl_3$)
210 200 190 180 170 160 150 140 130 120 110 100 90 80 70 60 50 40 30 20 10 0 -10
f1 (ppm)

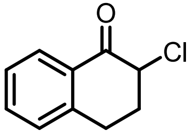

5b-Cl (^{1}H NMR 400MHz, $CDCl_3$)

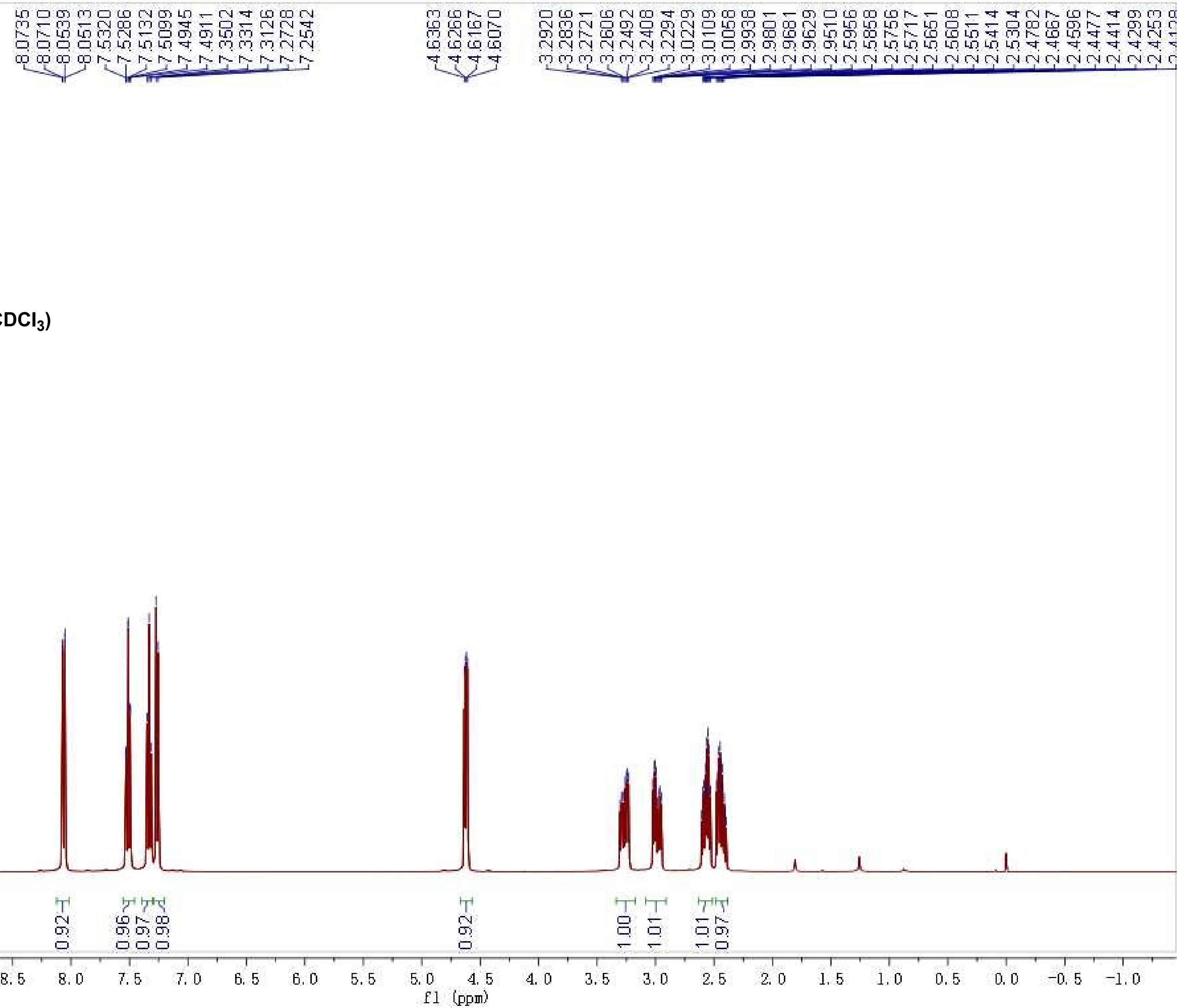

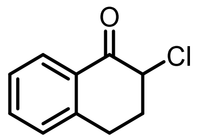

5b-Cl (^{13}C NMR 101MHz, $CDCl_3$)

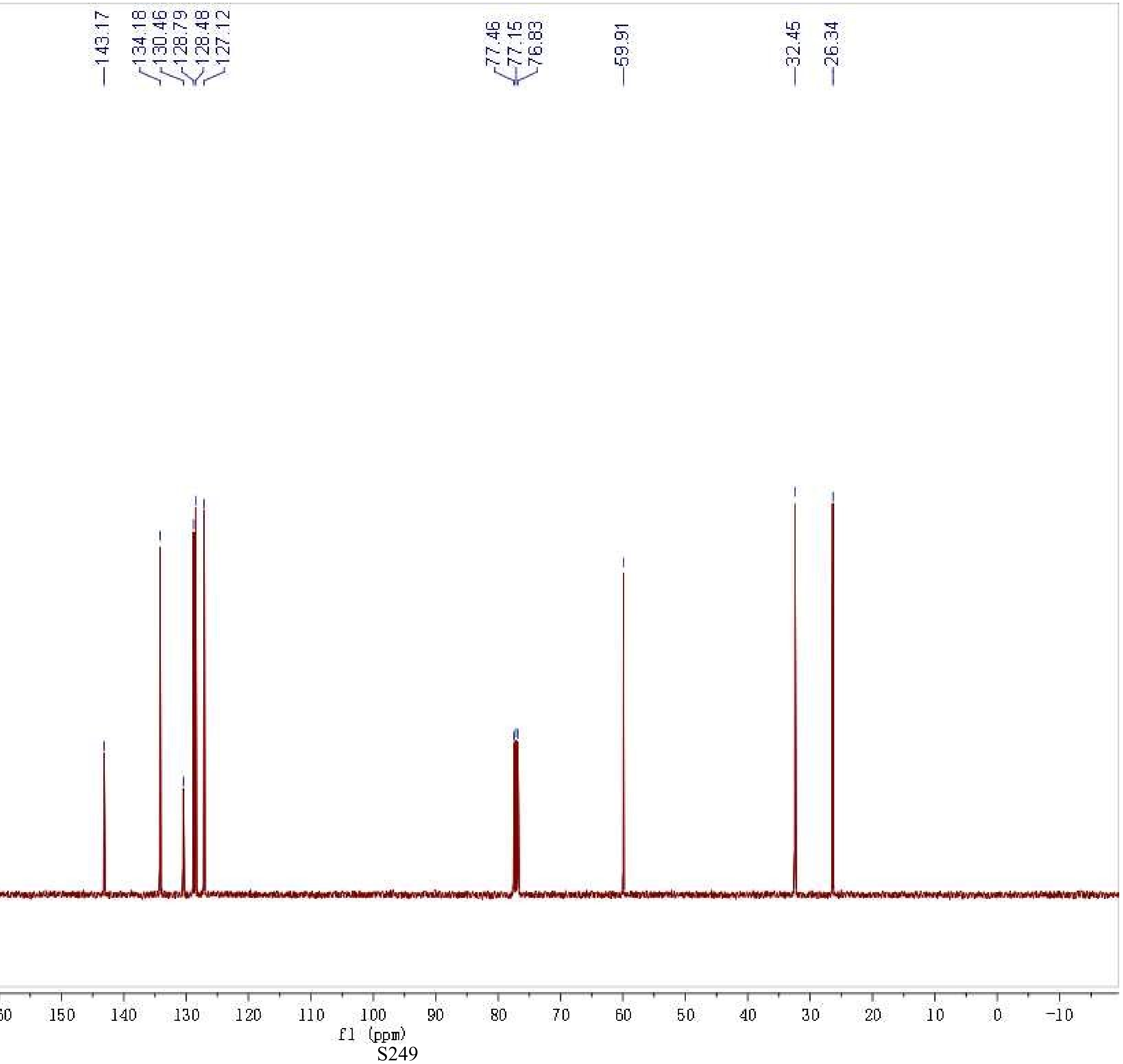

5b-Br (^{1}H NMR 400MHz, $CDCl_3$)

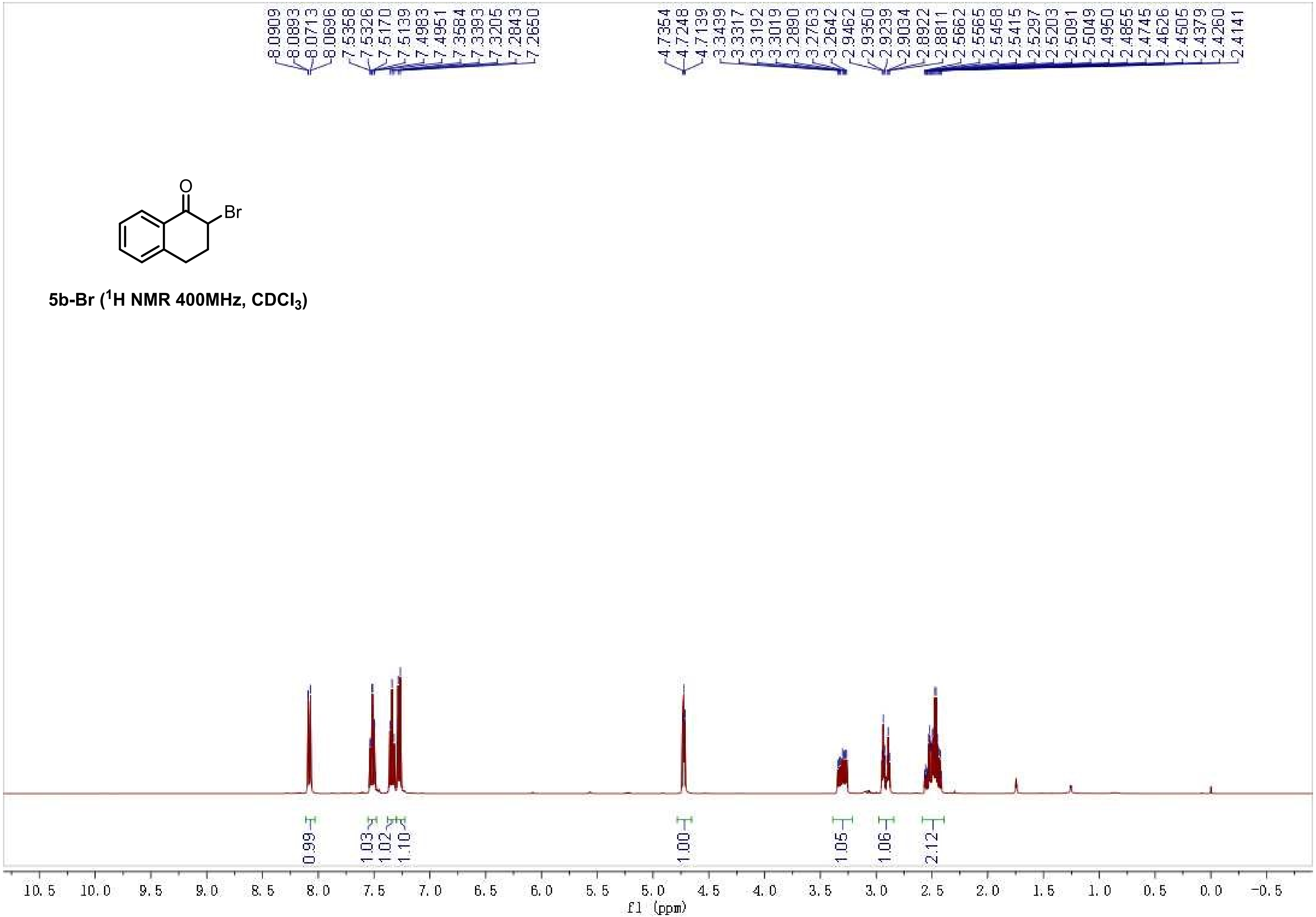

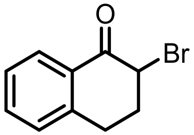

5b-Br (^{13}C NMR 101MHz, $CDCl_3$)

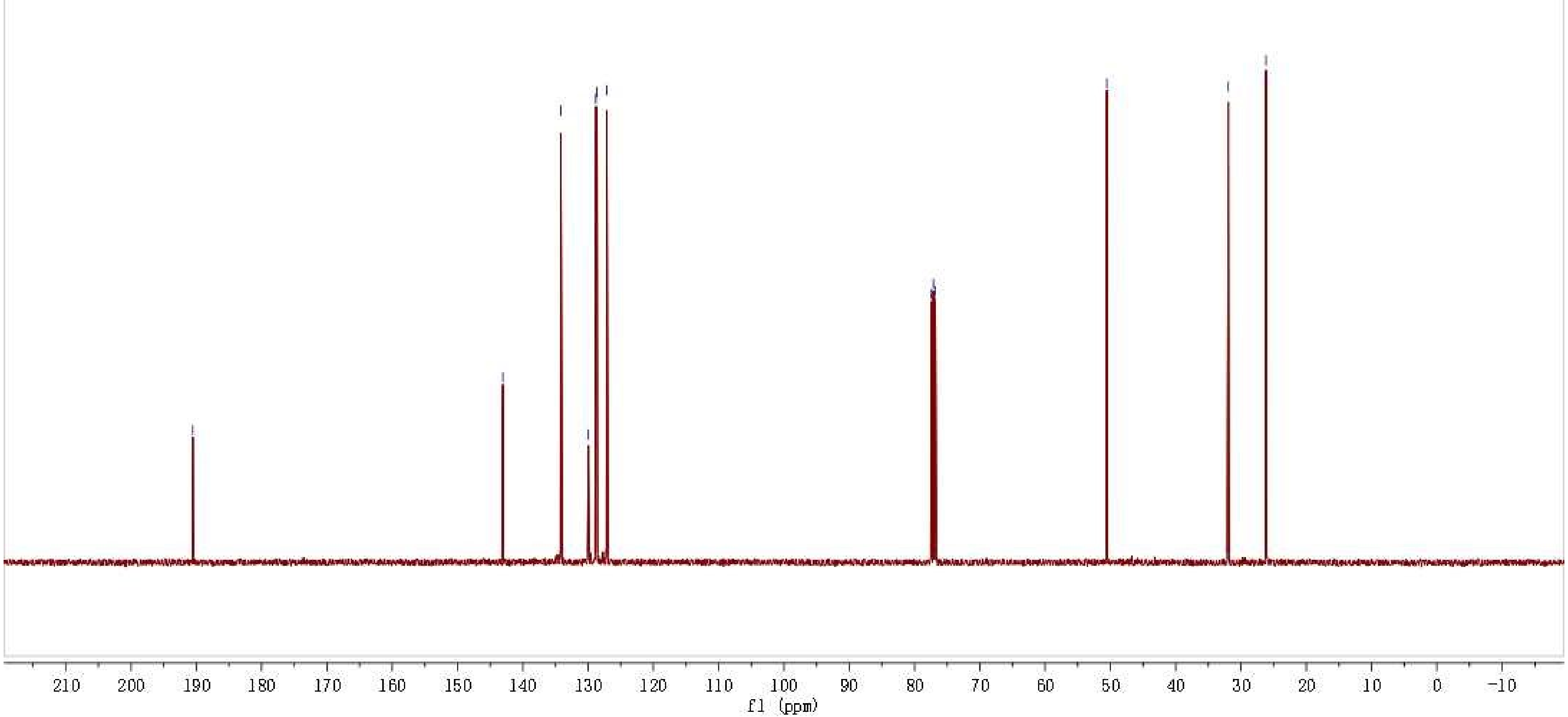

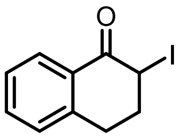

5b-I (^{1}H NMR 400MHz, $CDCl_3$)

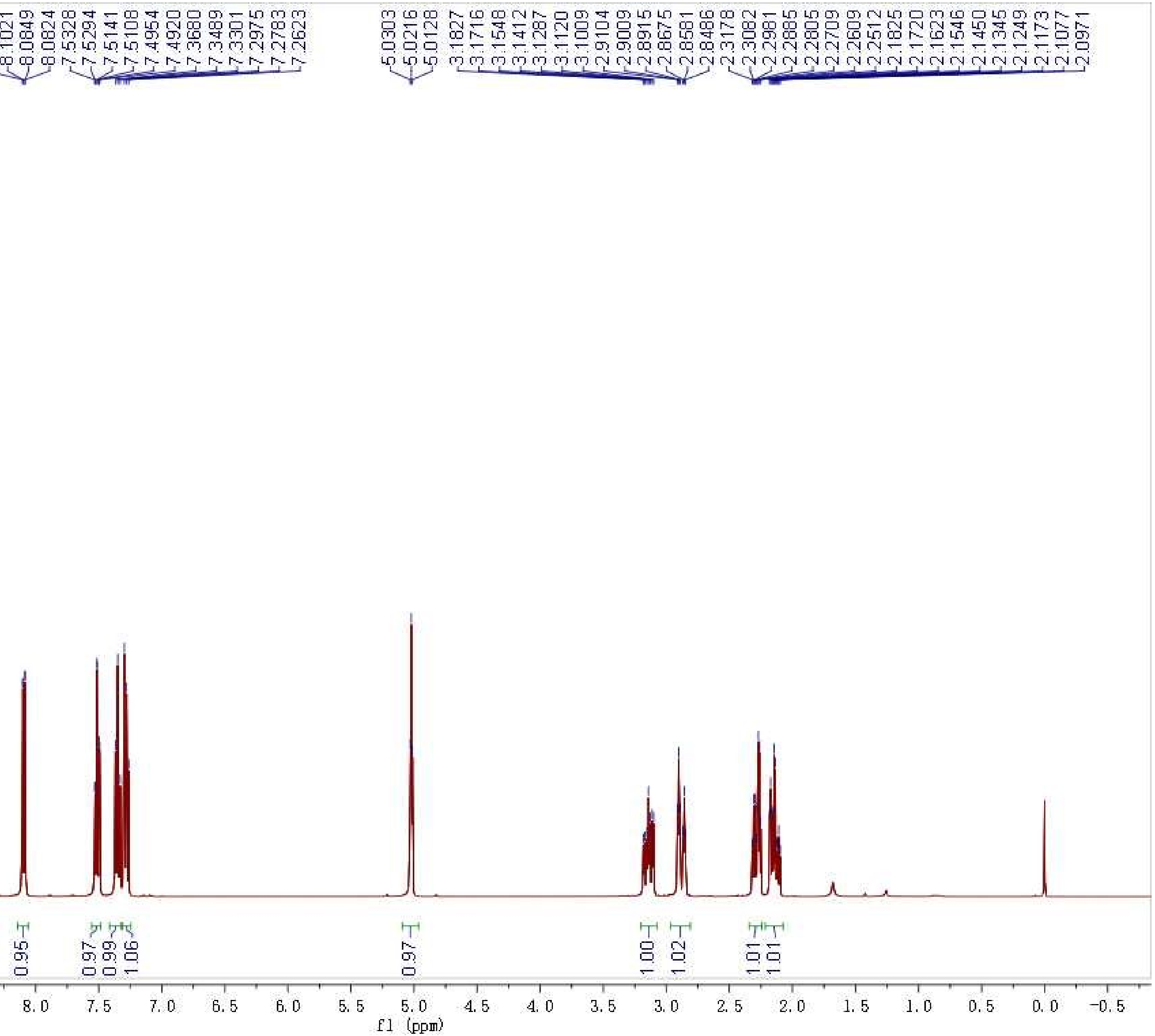

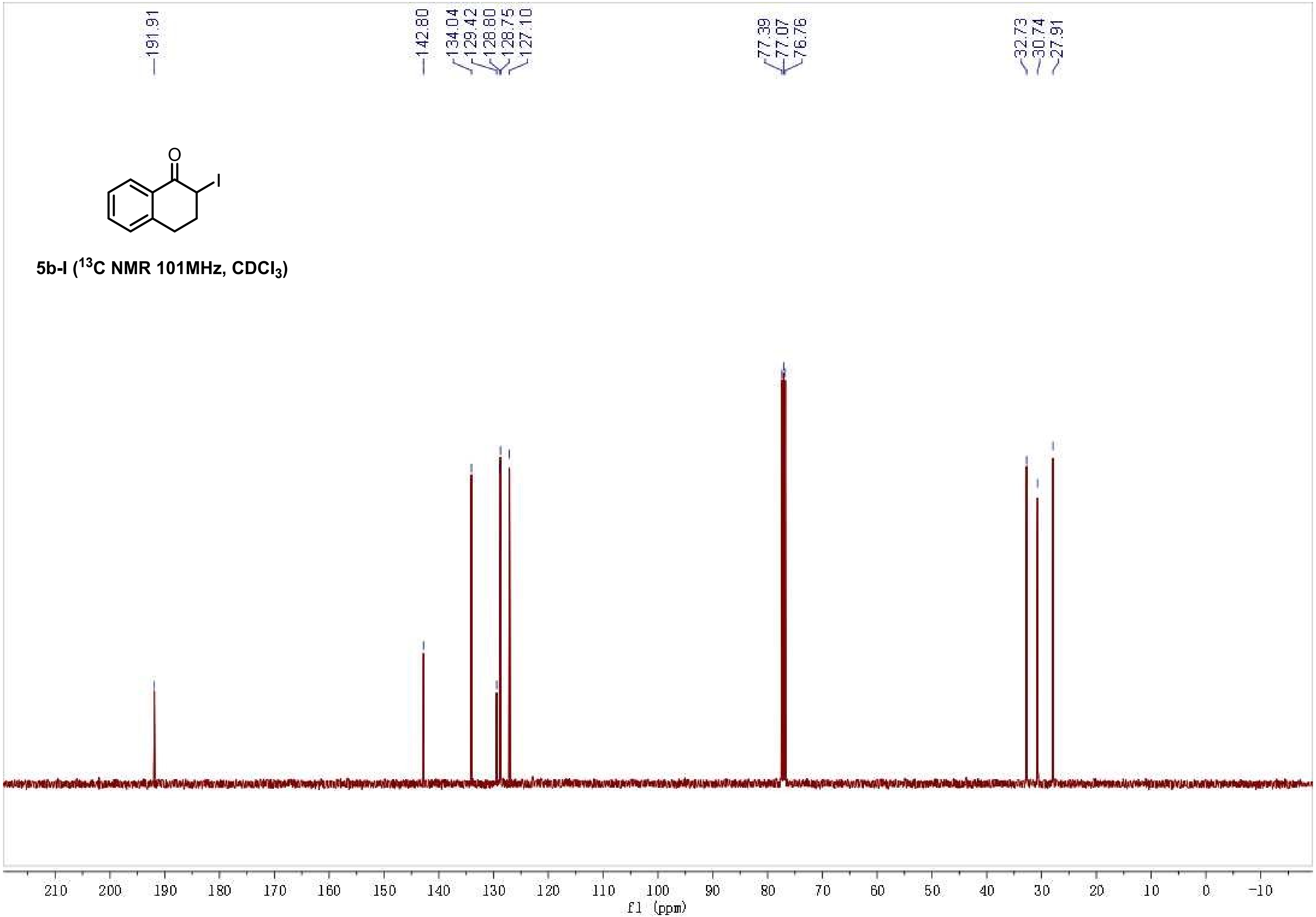

5b-I (^{13}C NMR 101MHz, $CDCl_3$)

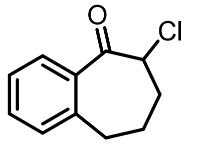

5c-Cl (^1H NMR 400MHz, $CDCl_3$)

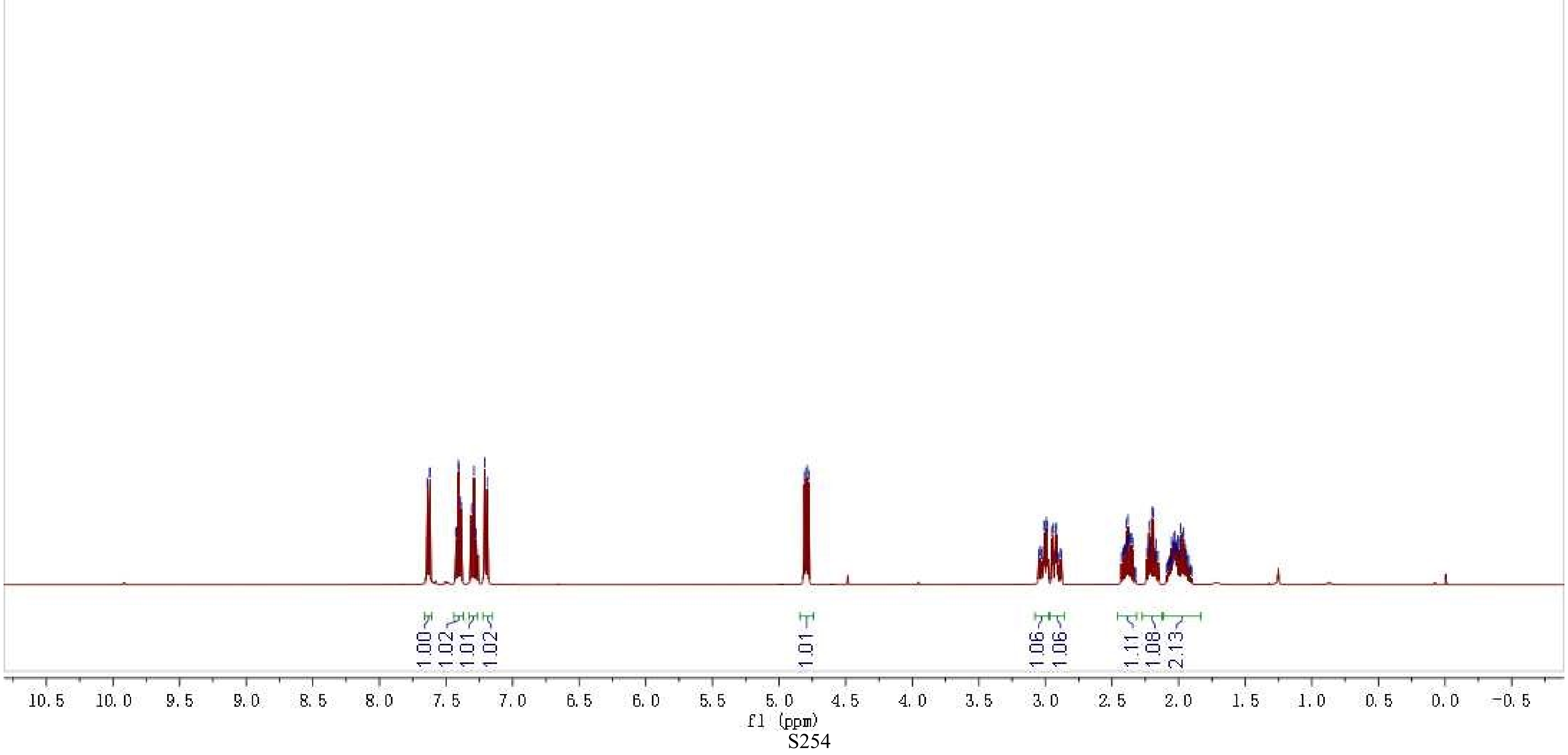

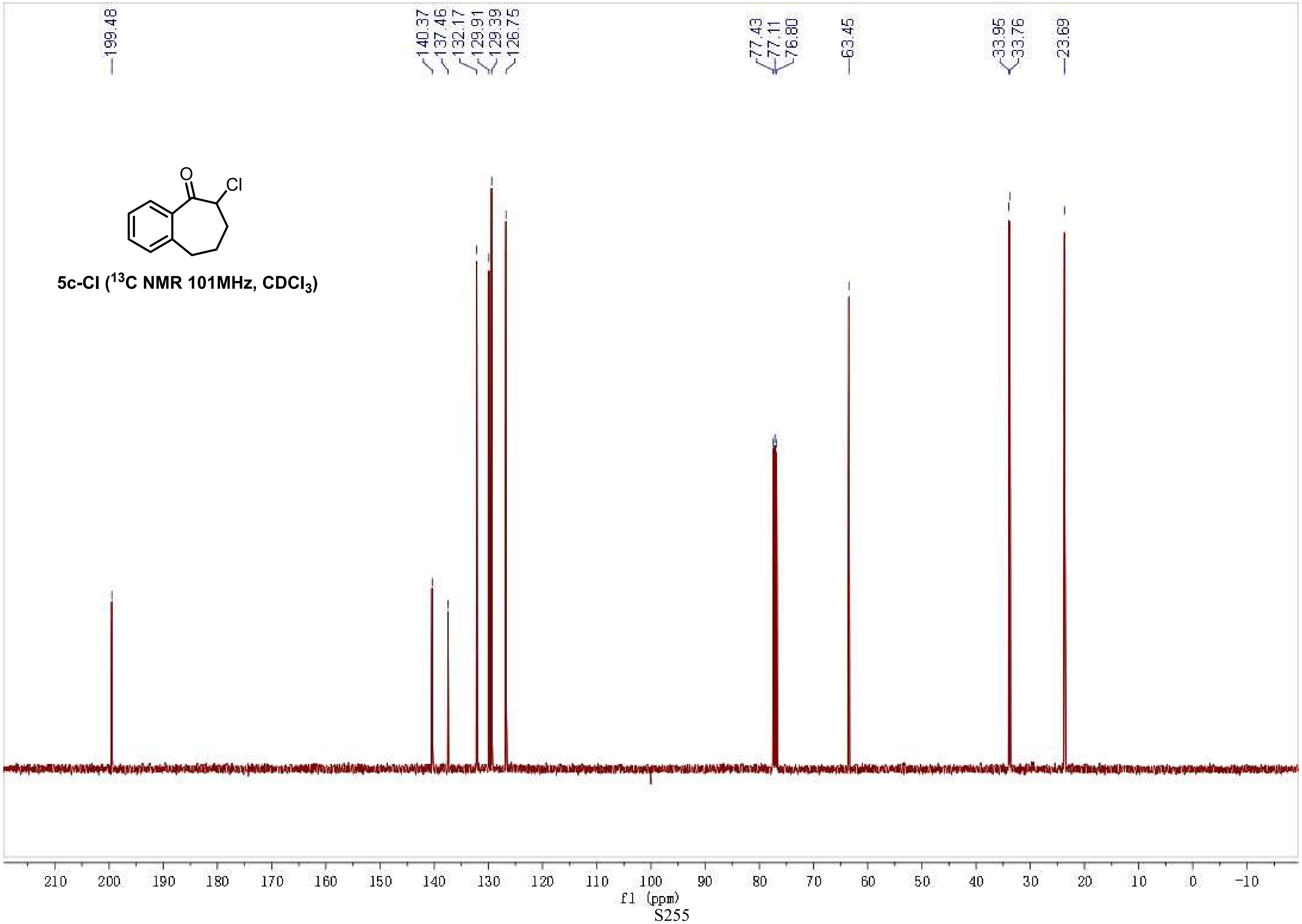
199.48
140.37
137.46
132.17
129.91
129.39
126.75
77.43
77.11
76.80
63.45
33.95
33.76
23.69
O
Cl
5c-Cl (13C NMR 101MHz, CDCl3)
210
200
190
180
170
160
150
140
130
120
110
100
90
80
70
60
50
40
30
20
10
0
-10
f1 (ppm)

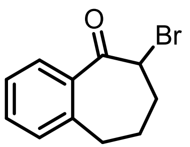

5c-Br (^1H NMR 400MHz, $CDCl_3$)

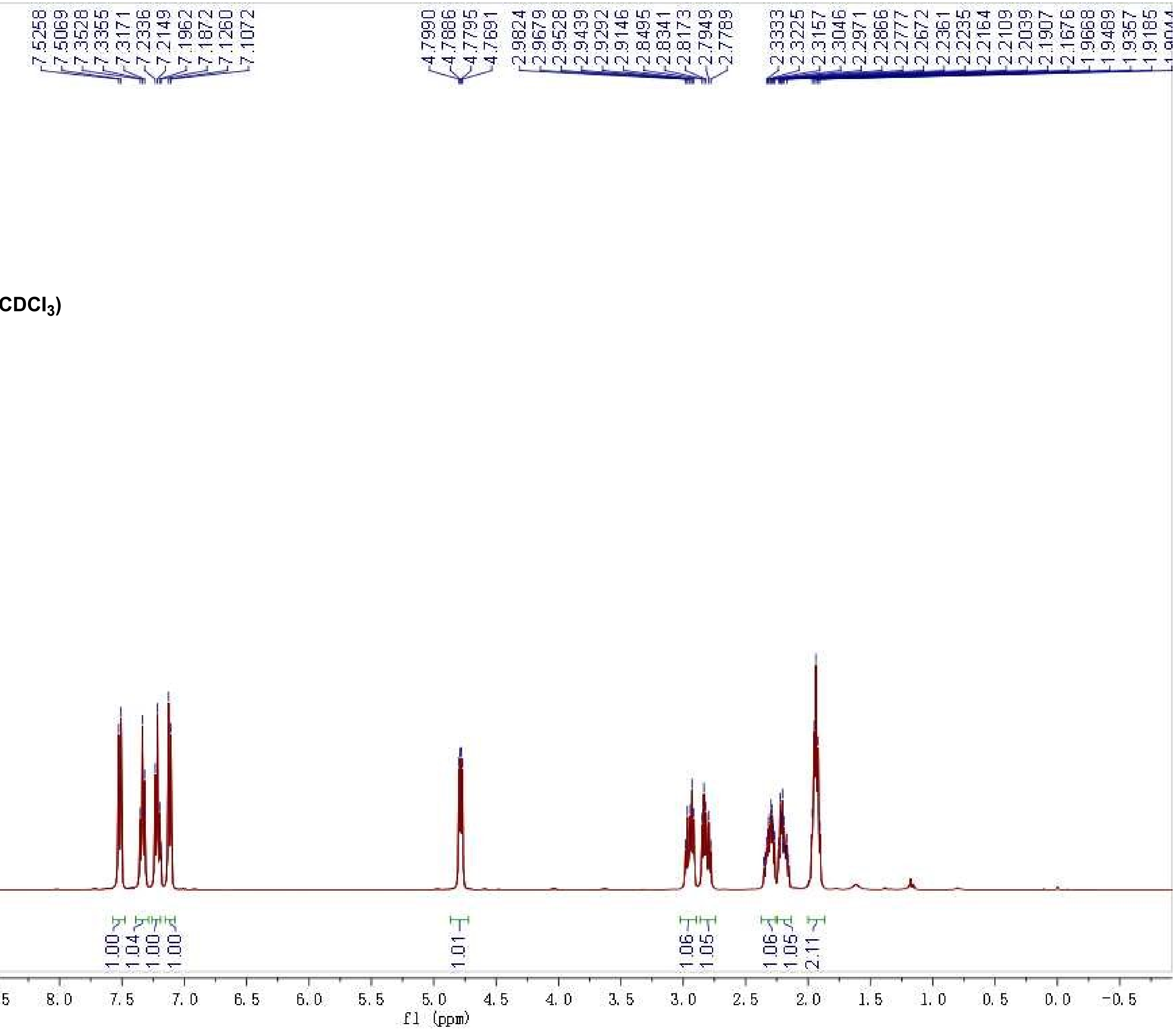

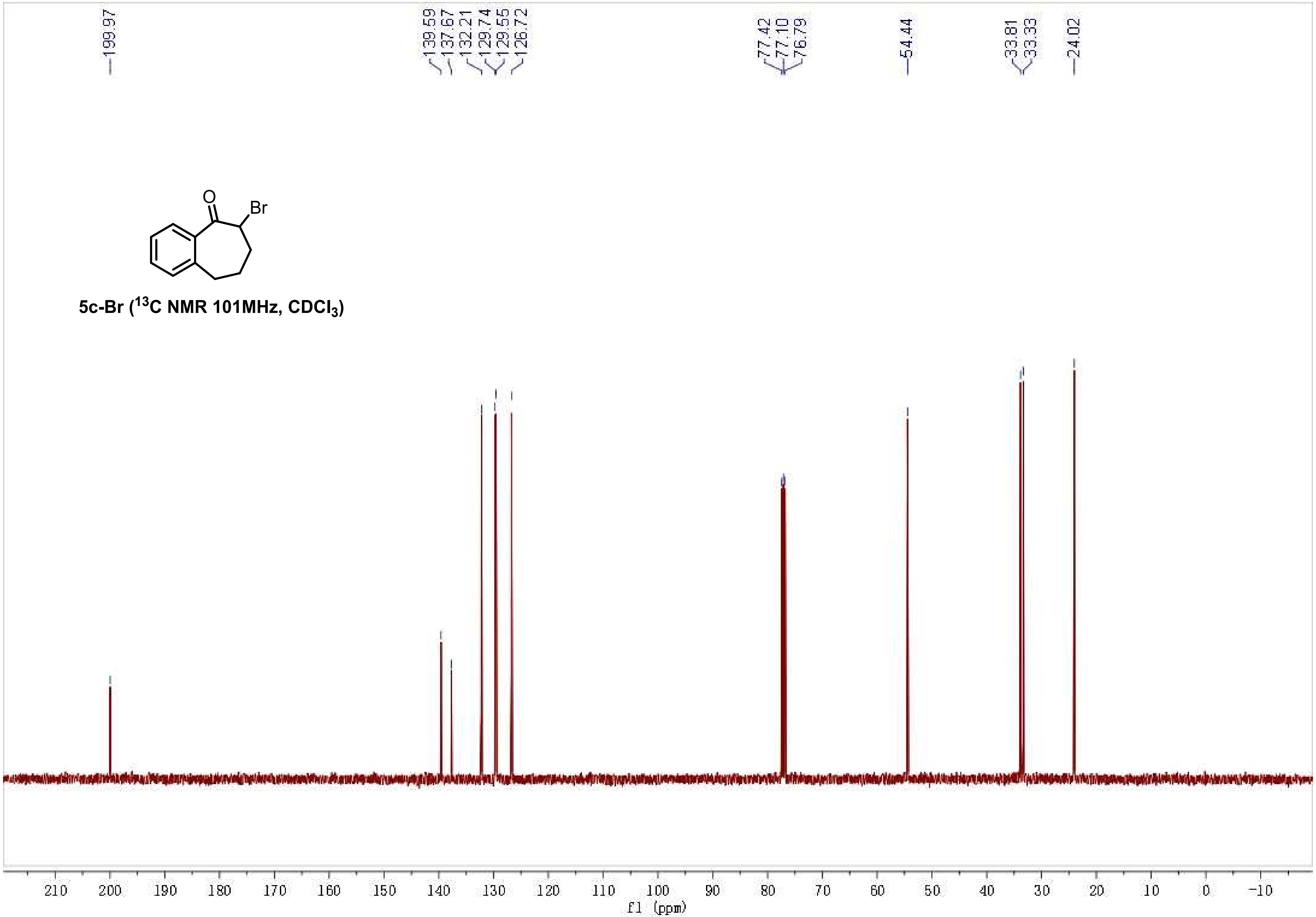

5c-Br (^{13}C NMR 101MHz, $CDCl_3$)

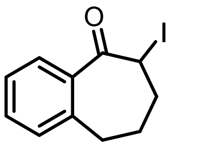

5c-I (^1H NMR 400MHz, $CDCl_3$)

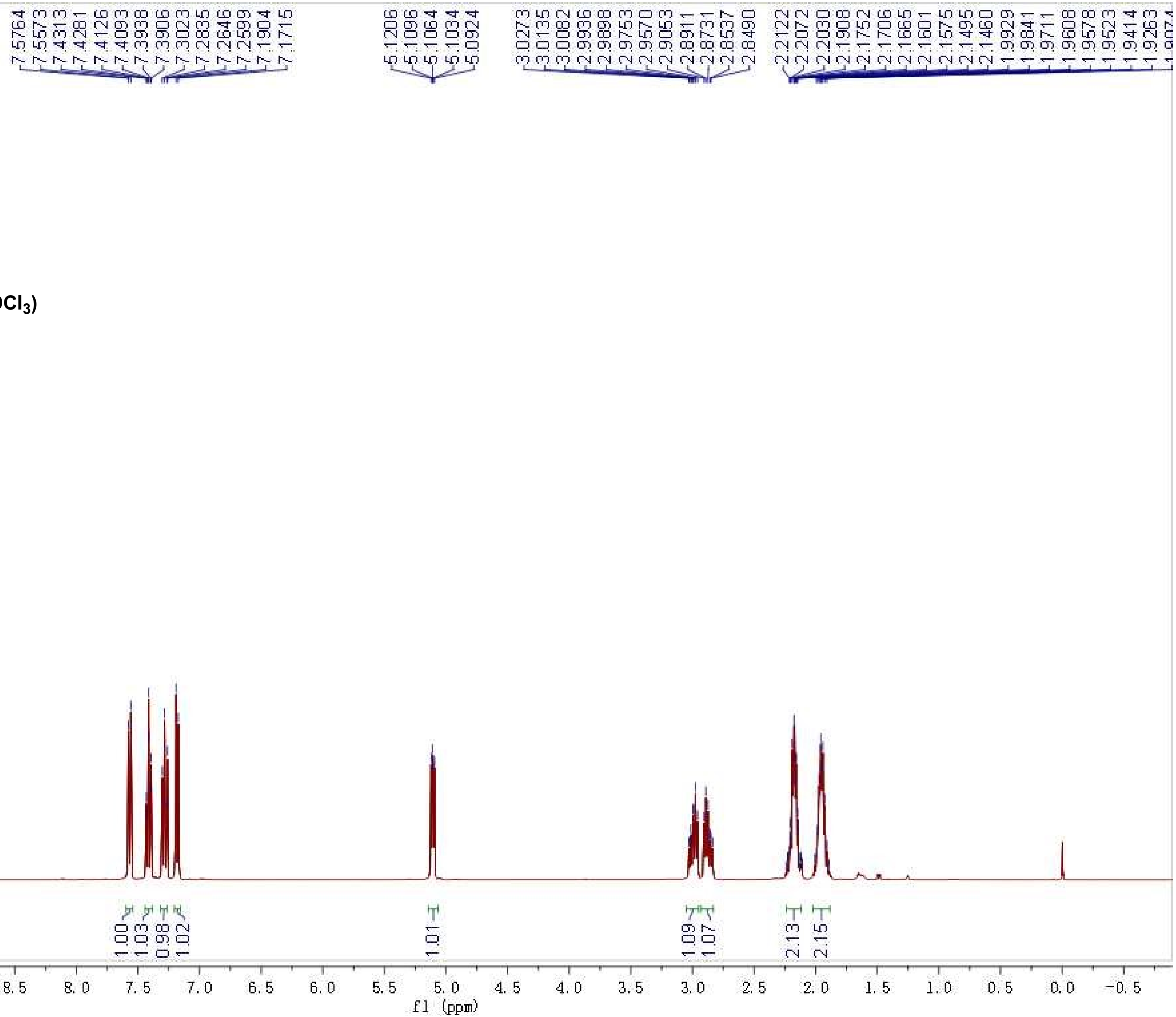

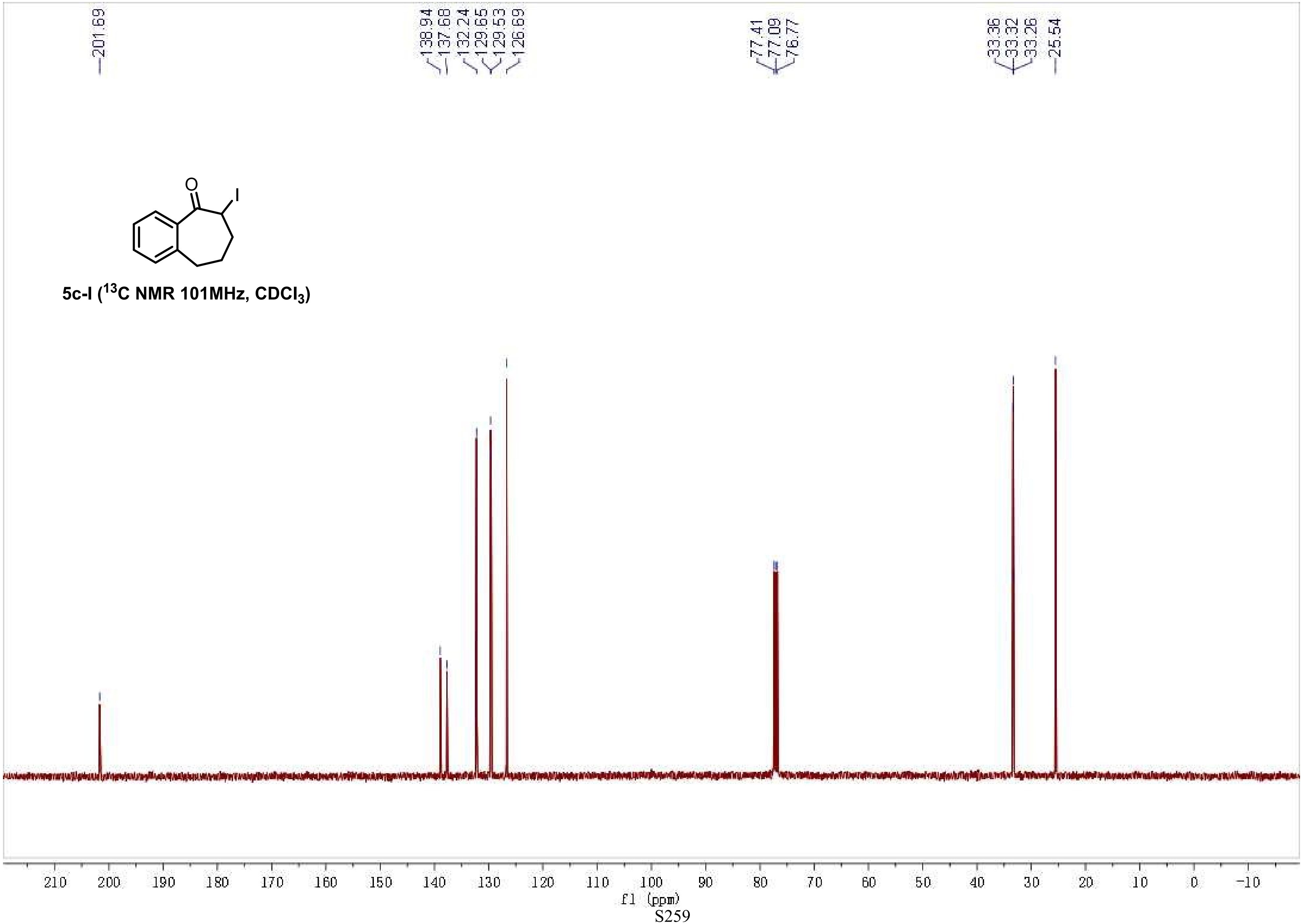

5c-I (^{13}C NMR 101MHz, $CDCl_3$)

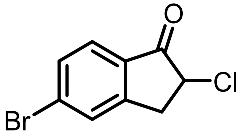

5d-Cl (^{1}H NMR 400MHz, $CDCl_3$)

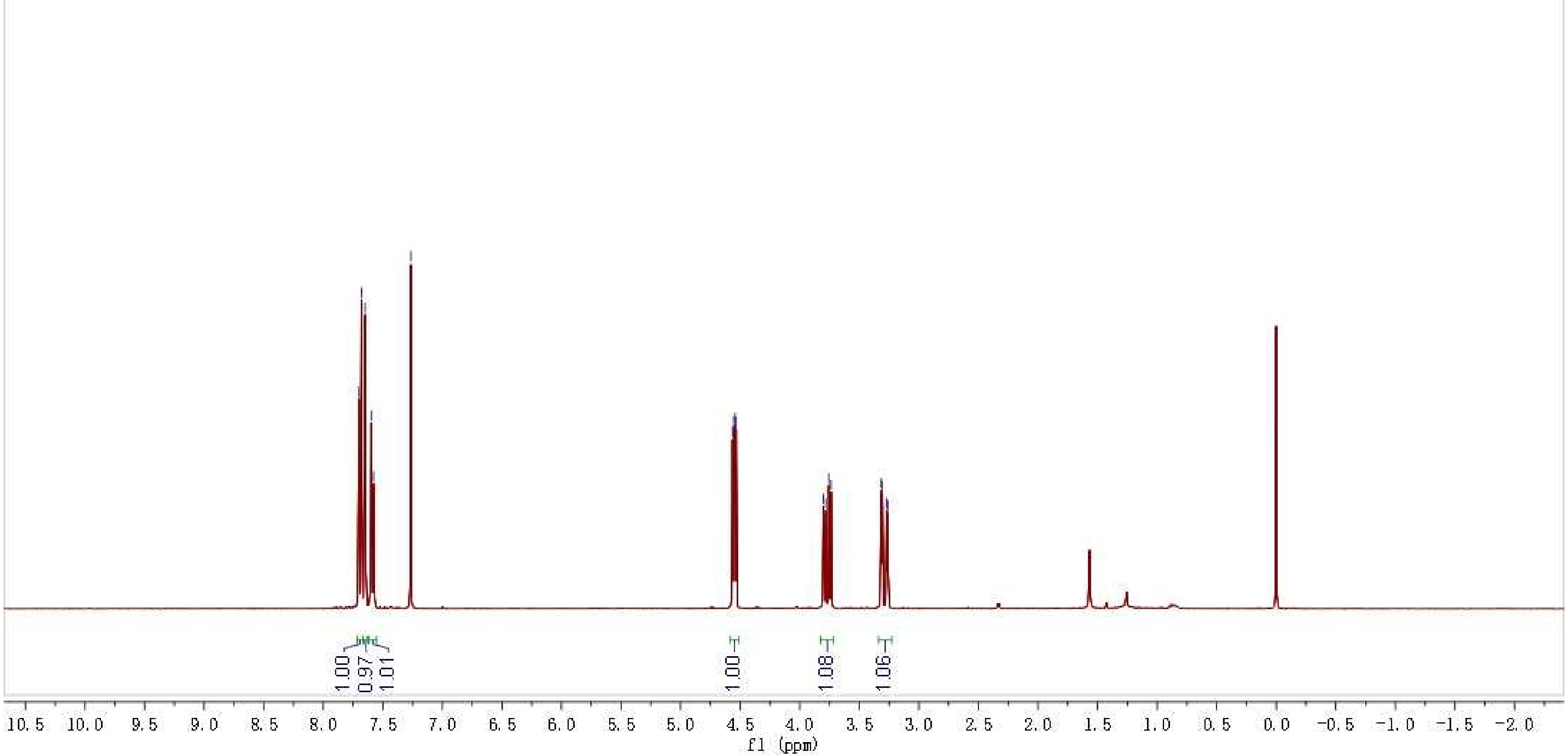

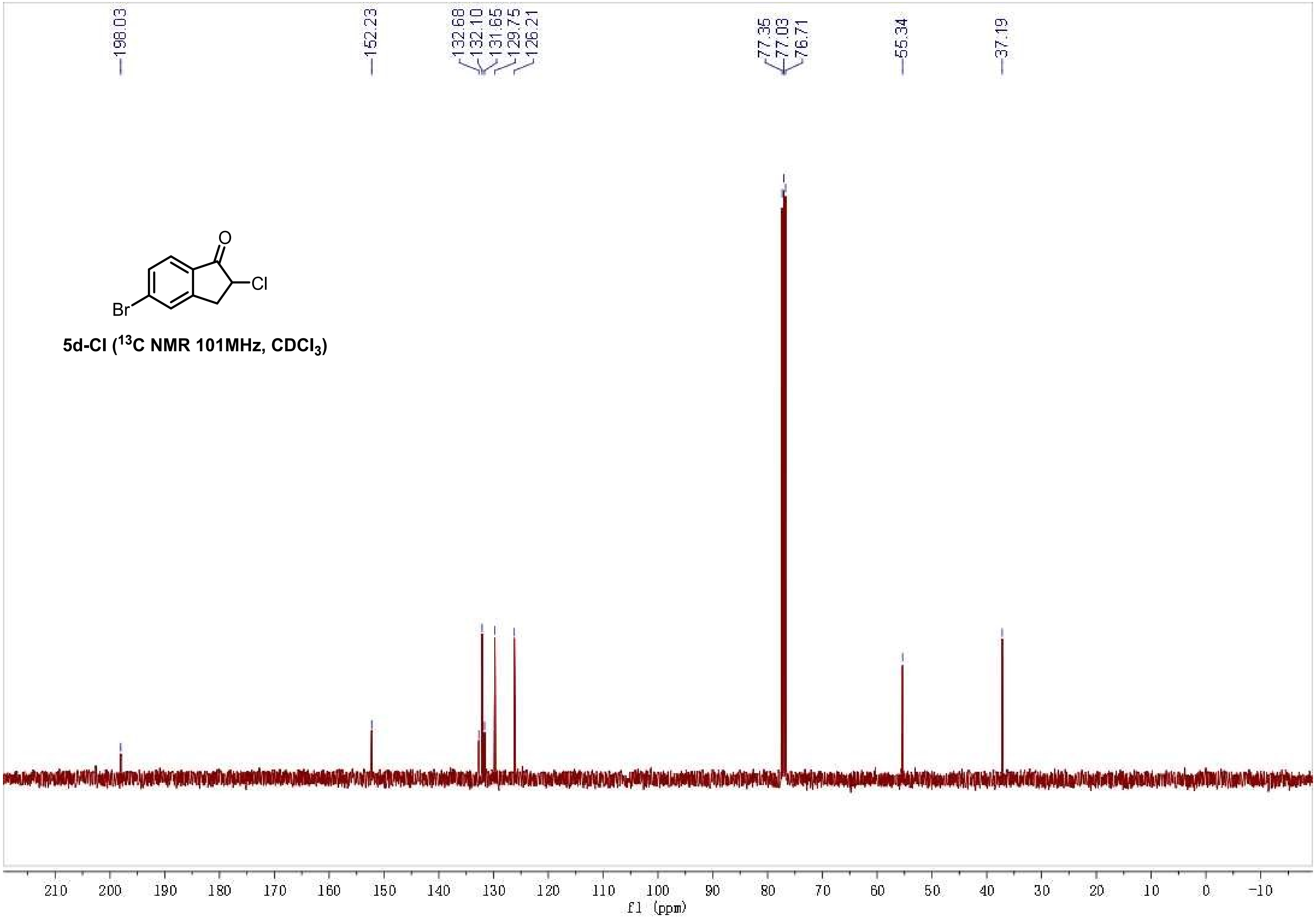

5d-Cl (^{13}C NMR 101MHz, $CDCl_3$)

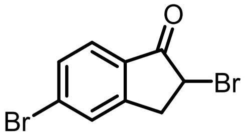

5d-Br (^{1}H NMR 400MHz, $CDCl_3$)

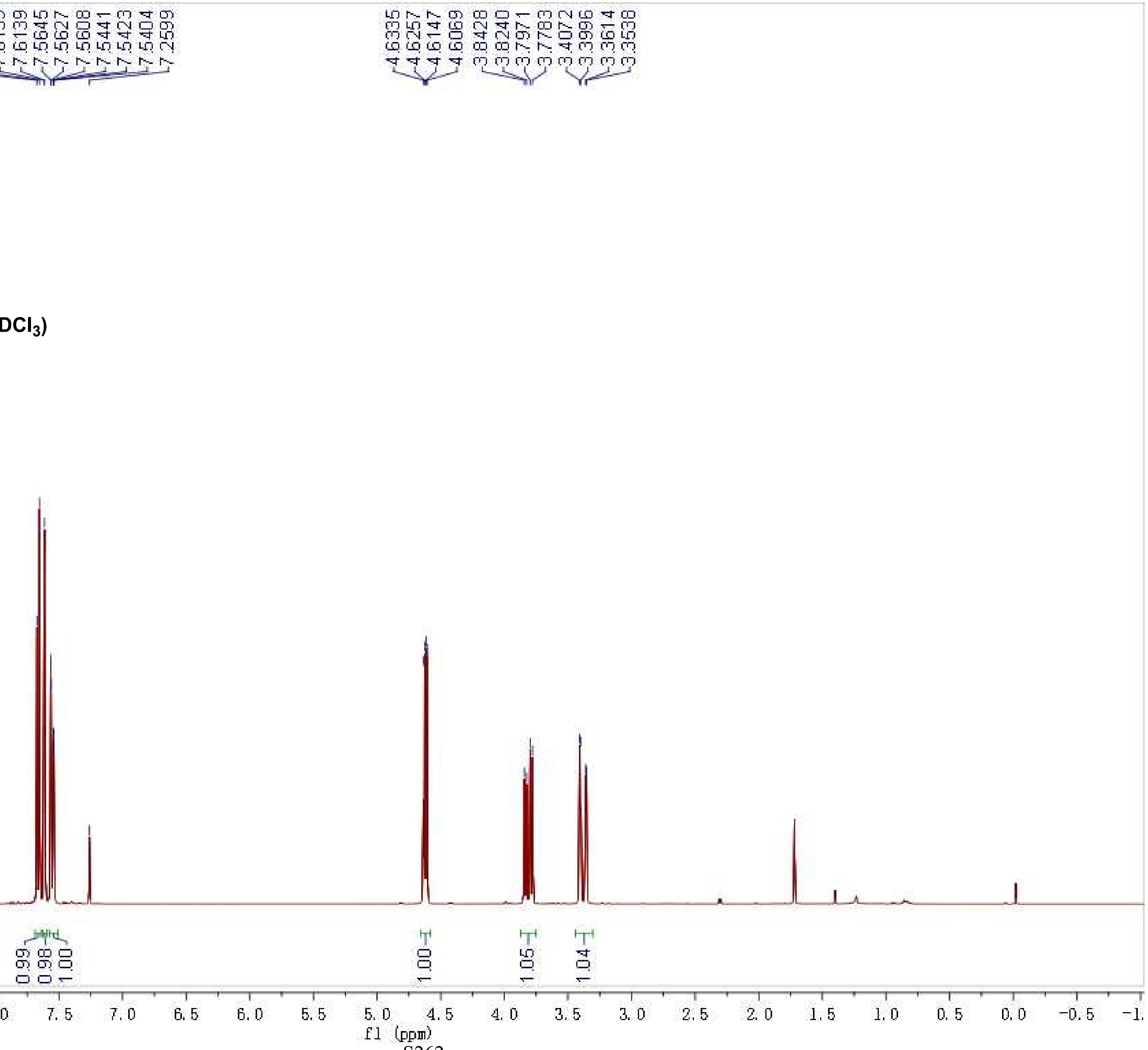

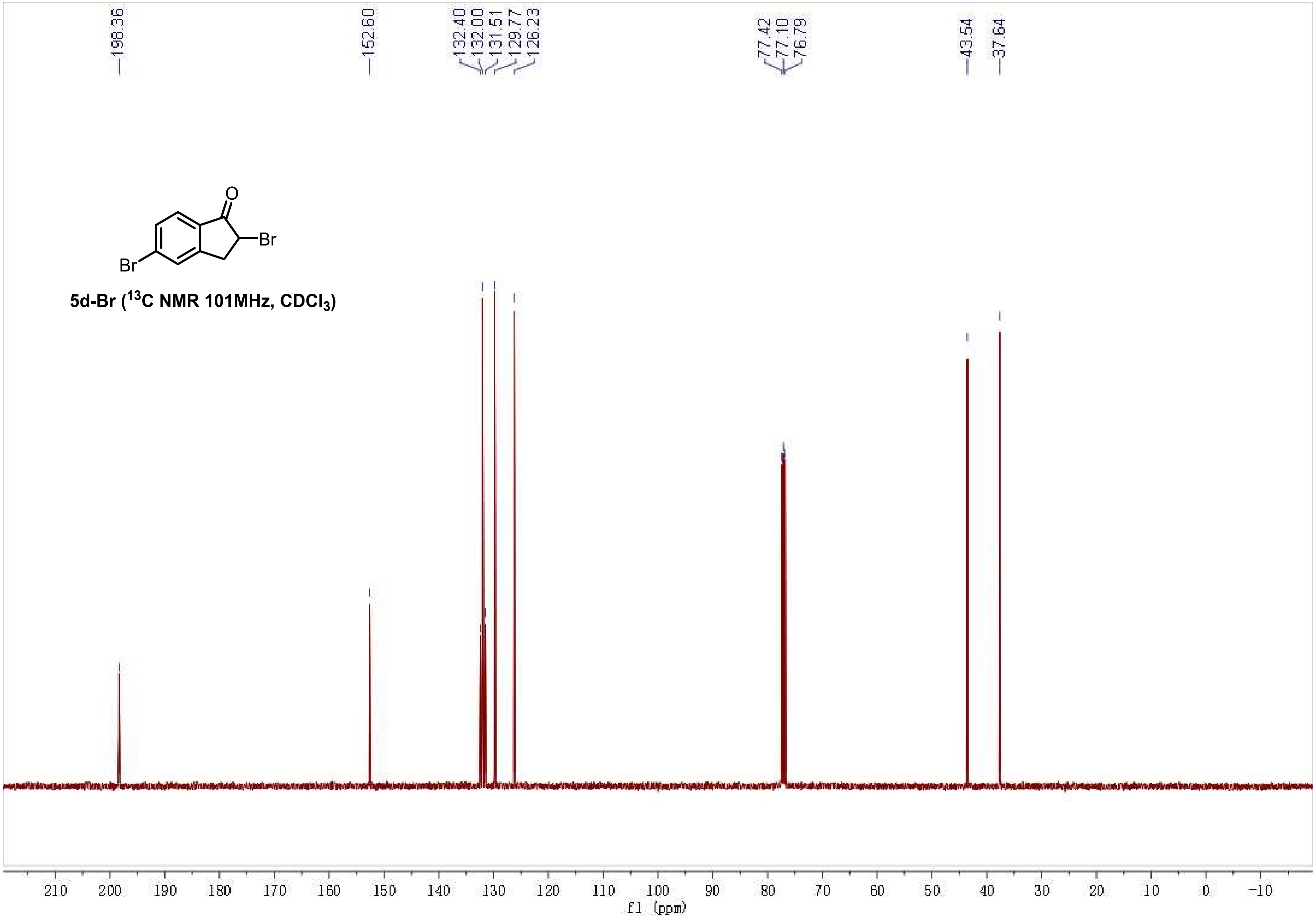

5d-Br (^{13}C NMR 101MHz, $CDCl_3$)

5d-I (^{1}H NMR 400MHz, $CDCl_3$)

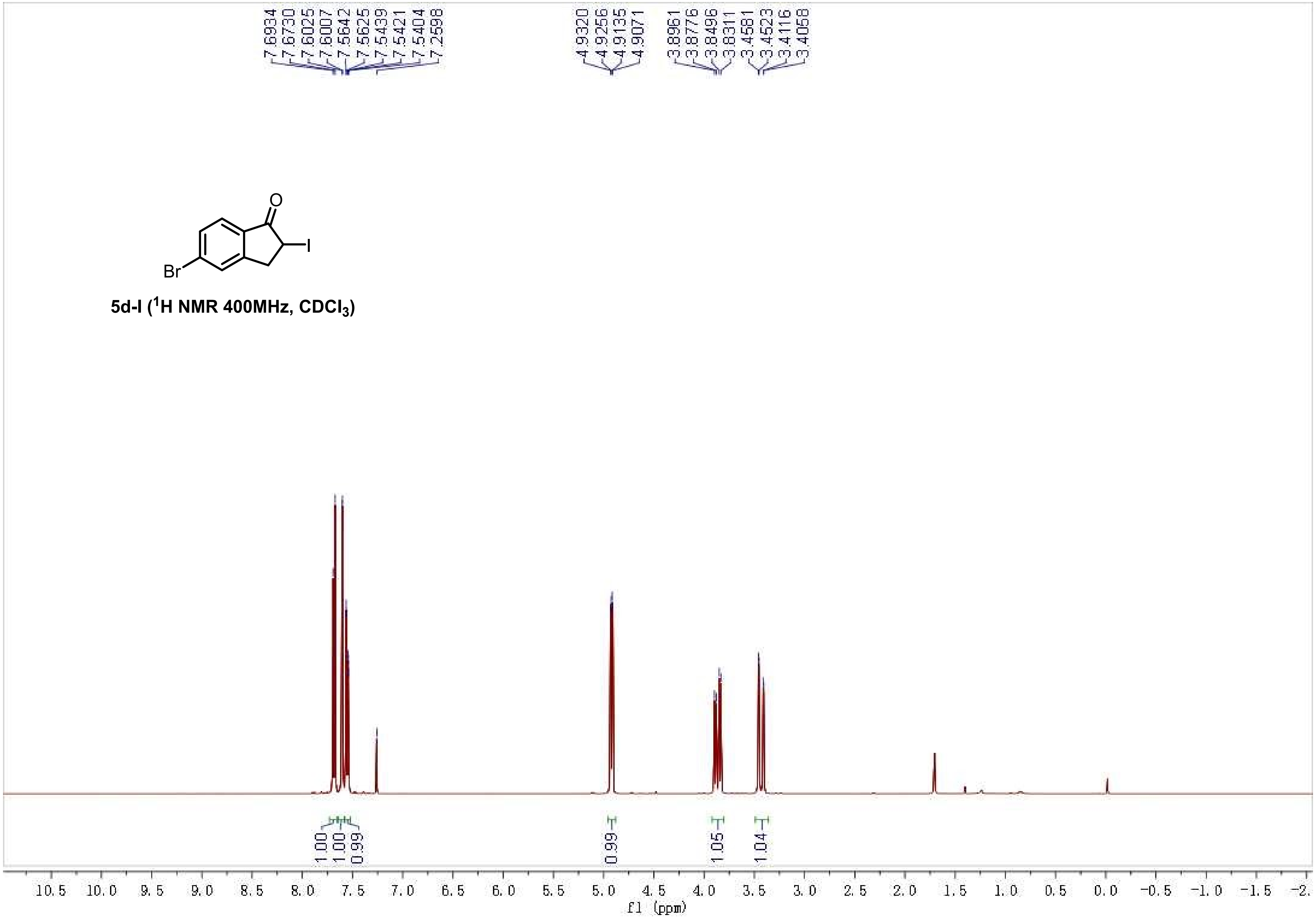

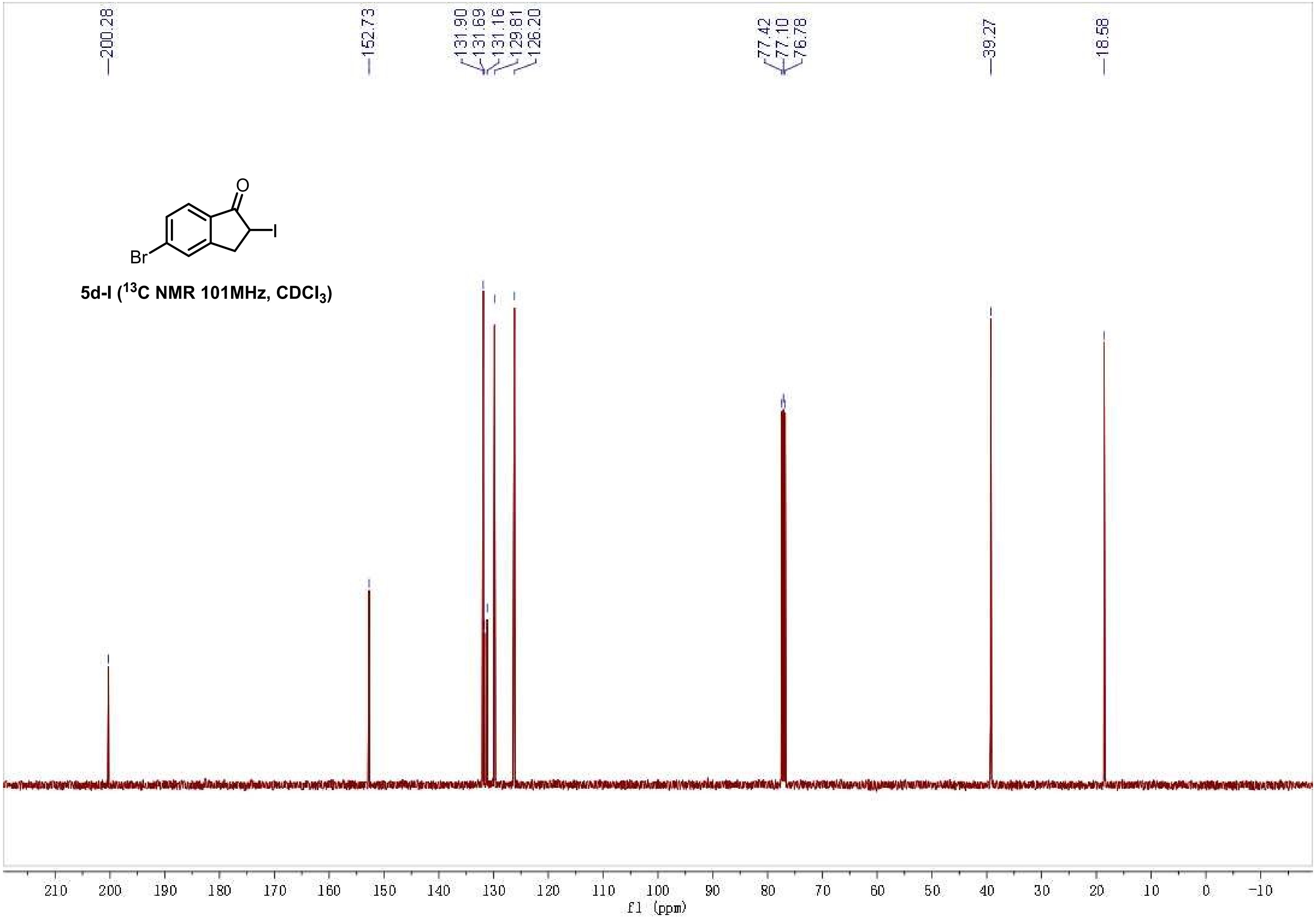

200.28
152.73
131.90
131.69
131.16
129.81
126.20
77.42
77.10
76.78
39.27
18.58
O
I
Br
5d-I (^{13}C NMR 101MHz, $CDCl_3$)
210 200 190 180 170 160 150 140 130 120 110 100 90 80 70 60 50 40 30 20 10 0 -10
f1 (ppm)

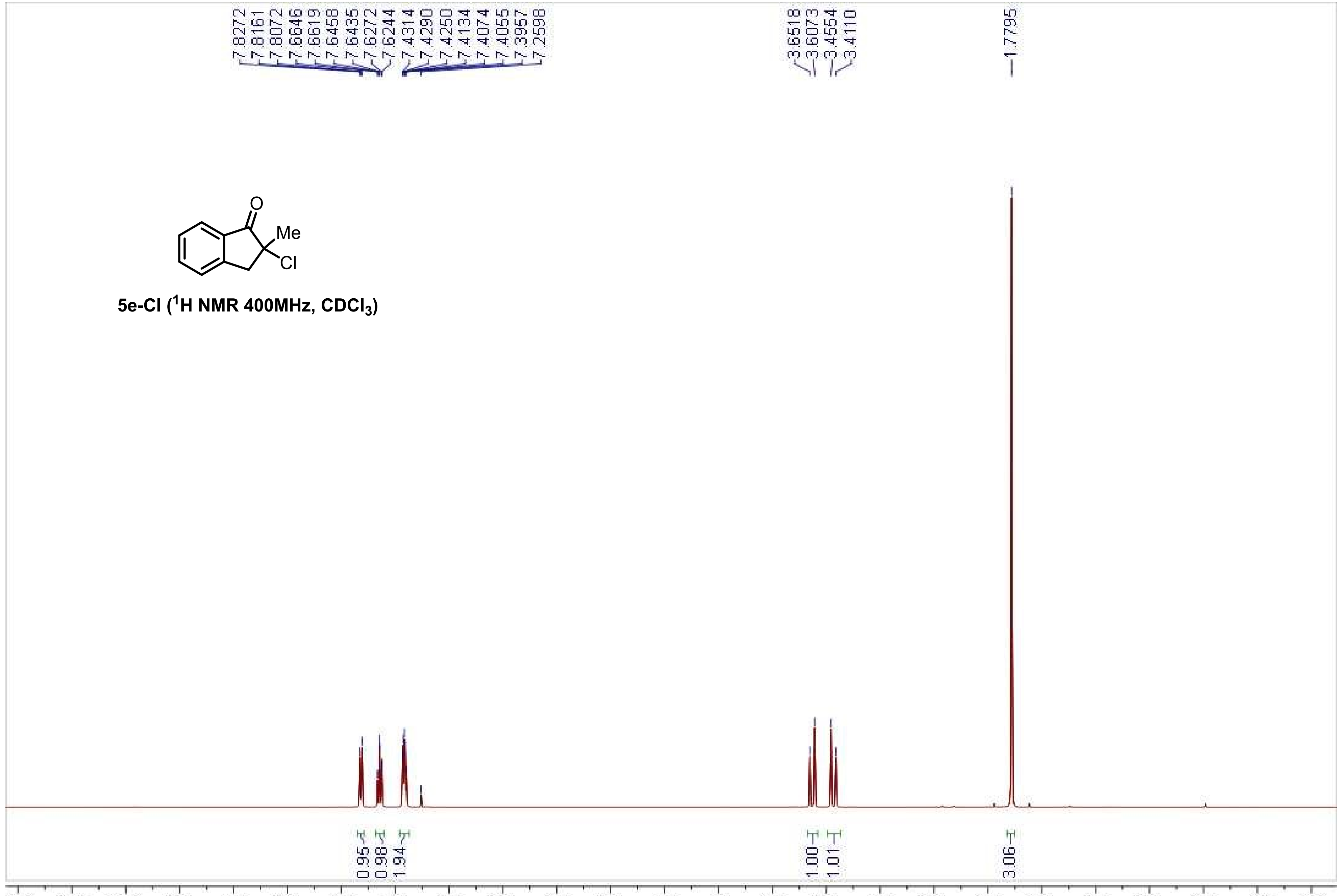
7.8272
7.8161
7.8072
7.6646
7.6619
7.6458
7.6435
7.6272
7.6244
7.4314
7.4290
7.4250
7.4134
7.4074
7.4055
7.3957
7.2598
3.6518
3.6073
3.4554
3.4110
1.7795
O
Me
Cl
5e-Cl (1H NMR 400MHz, CDCl3)
0.95
0.98
1.94
1.00
1.01
3.06
11.0 10.5 10.0 9.5 9.0 8.5 8.0 7.5 7.0 6.5 6.0 5.5 5.0 4.5 4.0 3.5 3.0 2.5 2.0 1.5 1.0 0.5 0.0 -0.5 -1.0
f1 (ppm)

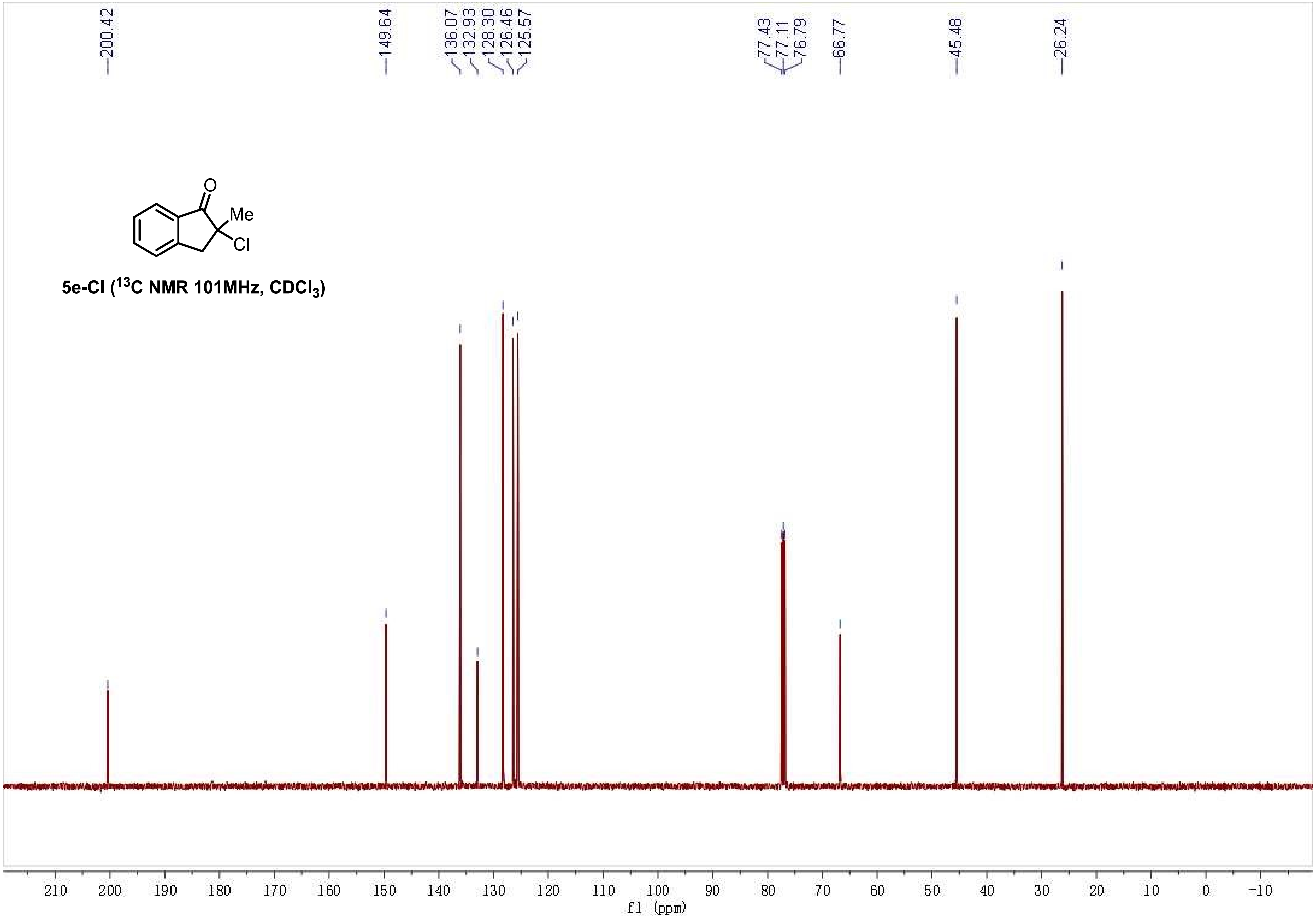
200.42
149.64
136.07
132.93
128.30
126.46
125.57
77.43
77.11
76.79
66.77
45.48
26.24
O
Me
Cl
5e-Cl (13C NMR 101MHz, CDCl3)
210 200 190 180 170 160 150 140 130 120 110 100 90 80 70 60 50 40 30 20 10 0 -10
f1 (ppm)

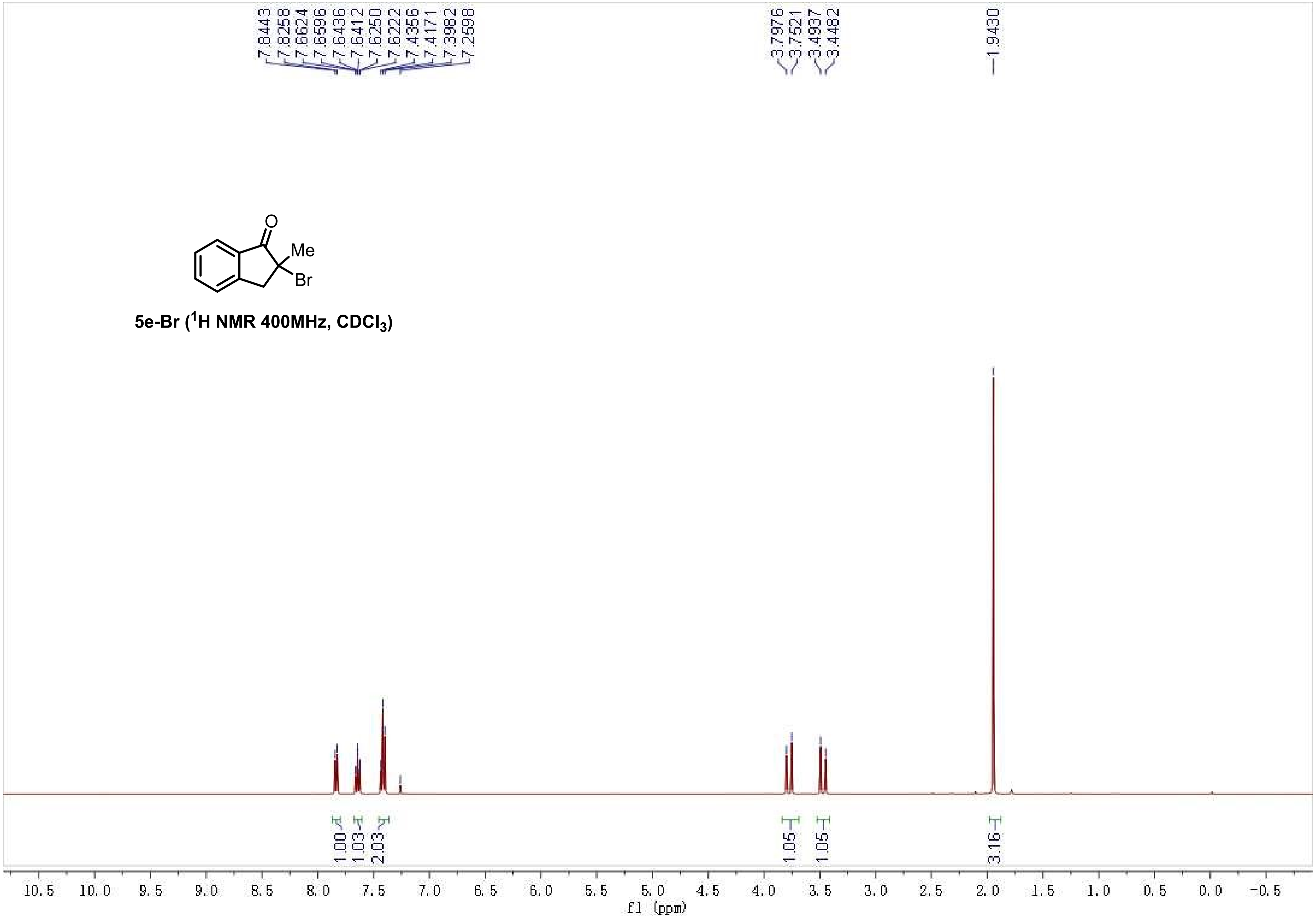

5e-Br (^{1}H NMR 400MHz, $CDCl_3$)

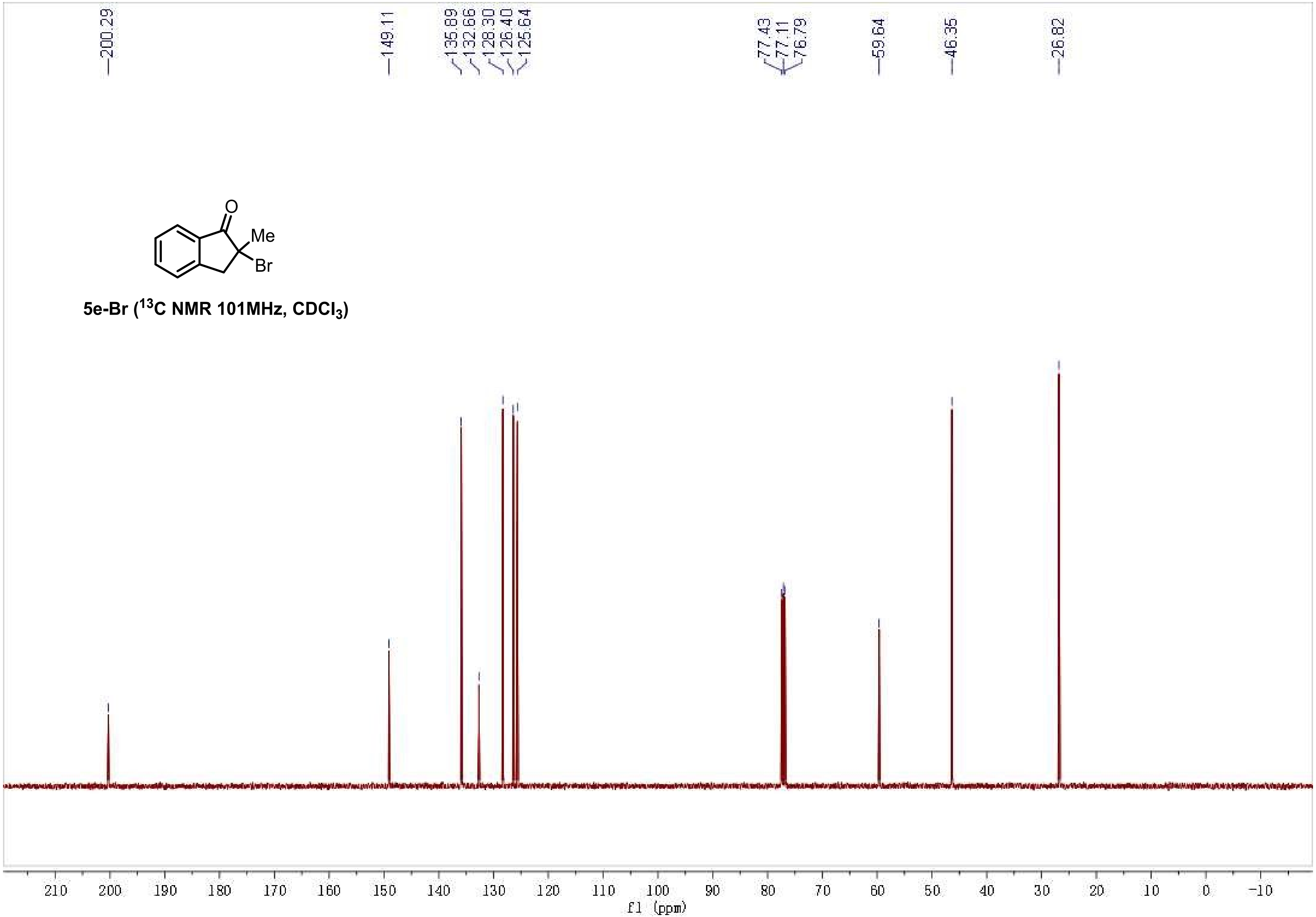

5e-Br (^{13}C NMR 101MHz, $CDCl_3$)

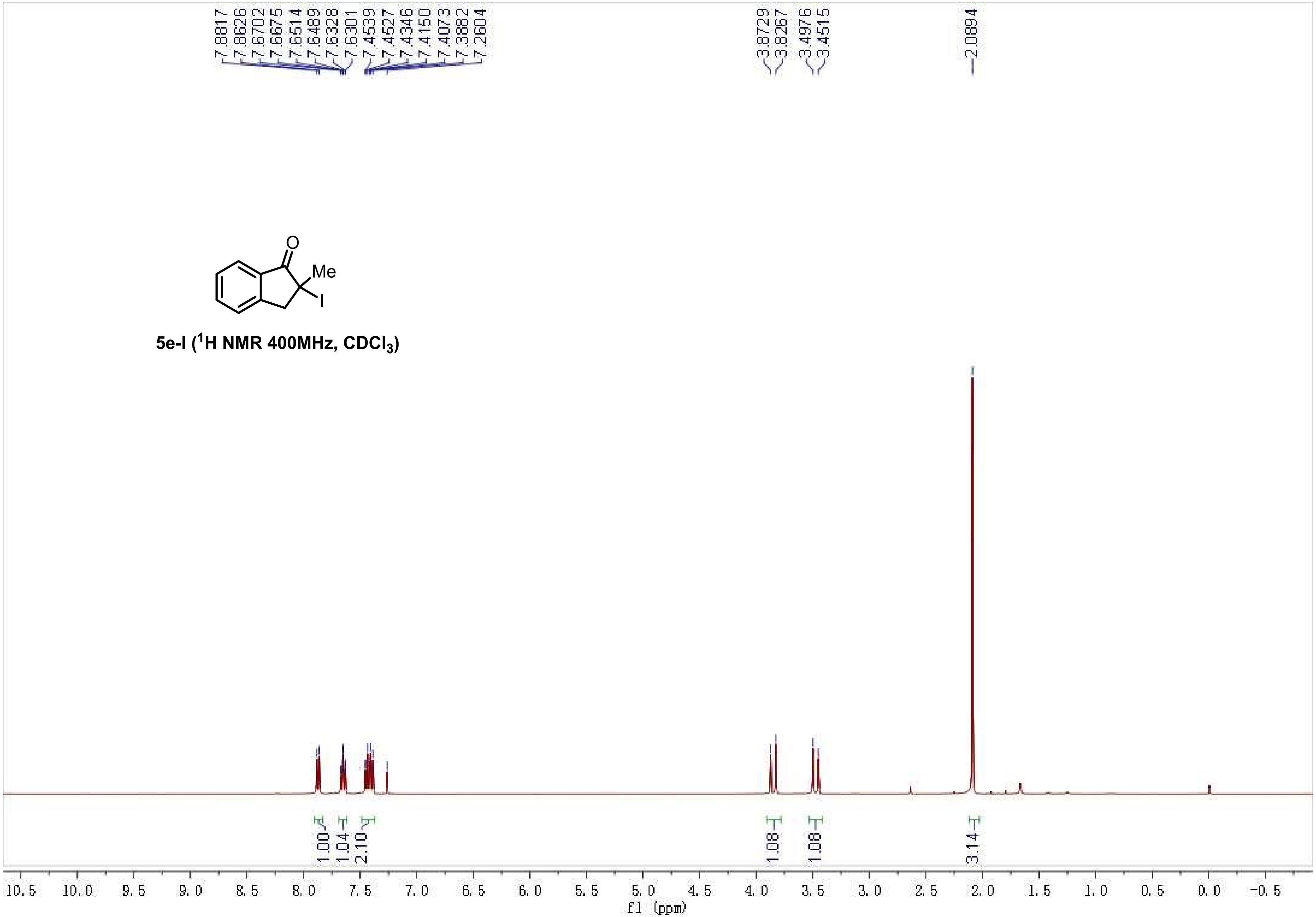

5e-I (^{1}H NMR 400MHz, CDCl$_3$)

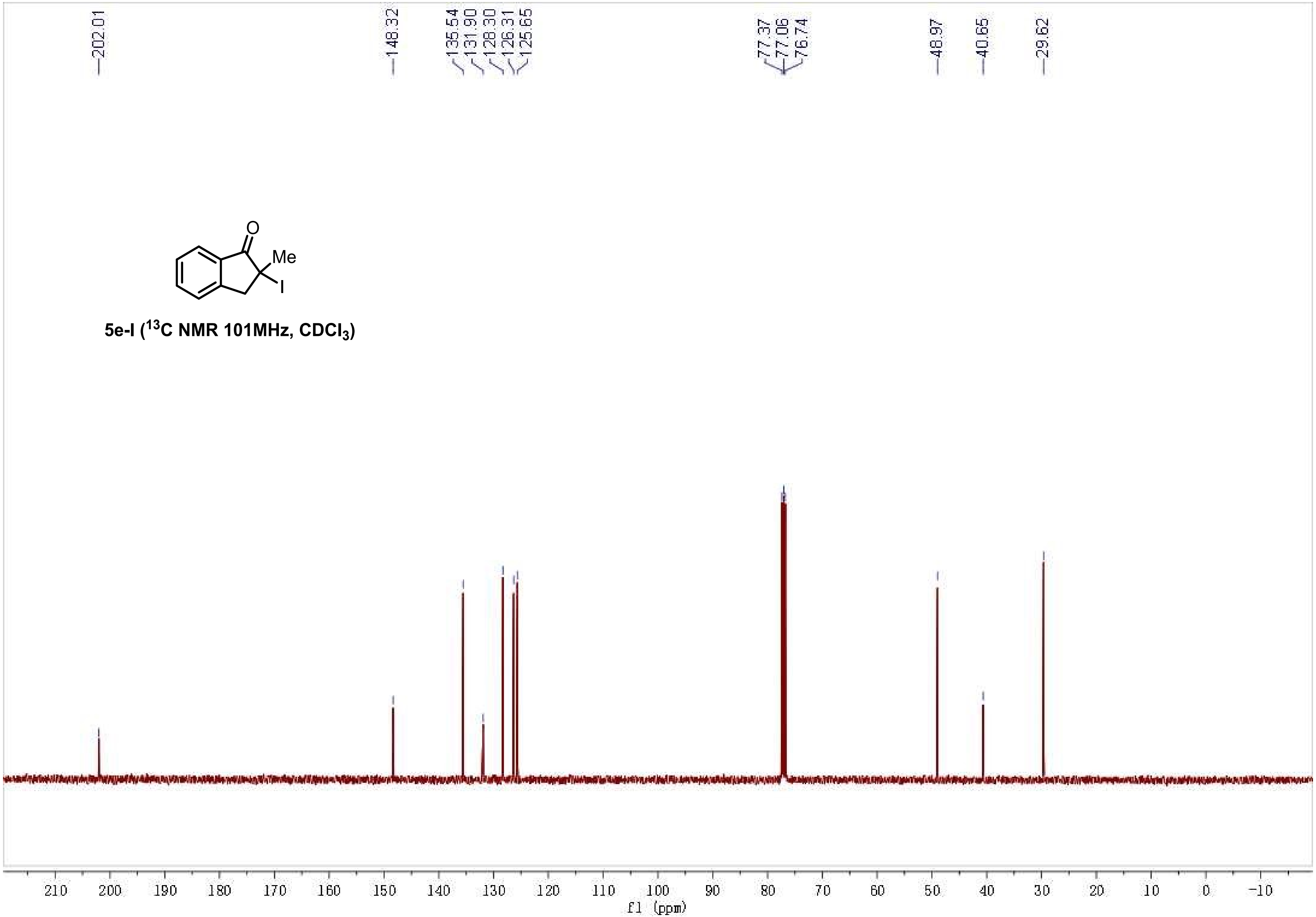

5e-I (^{13}C NMR 101MHz, $CDCl_3$)

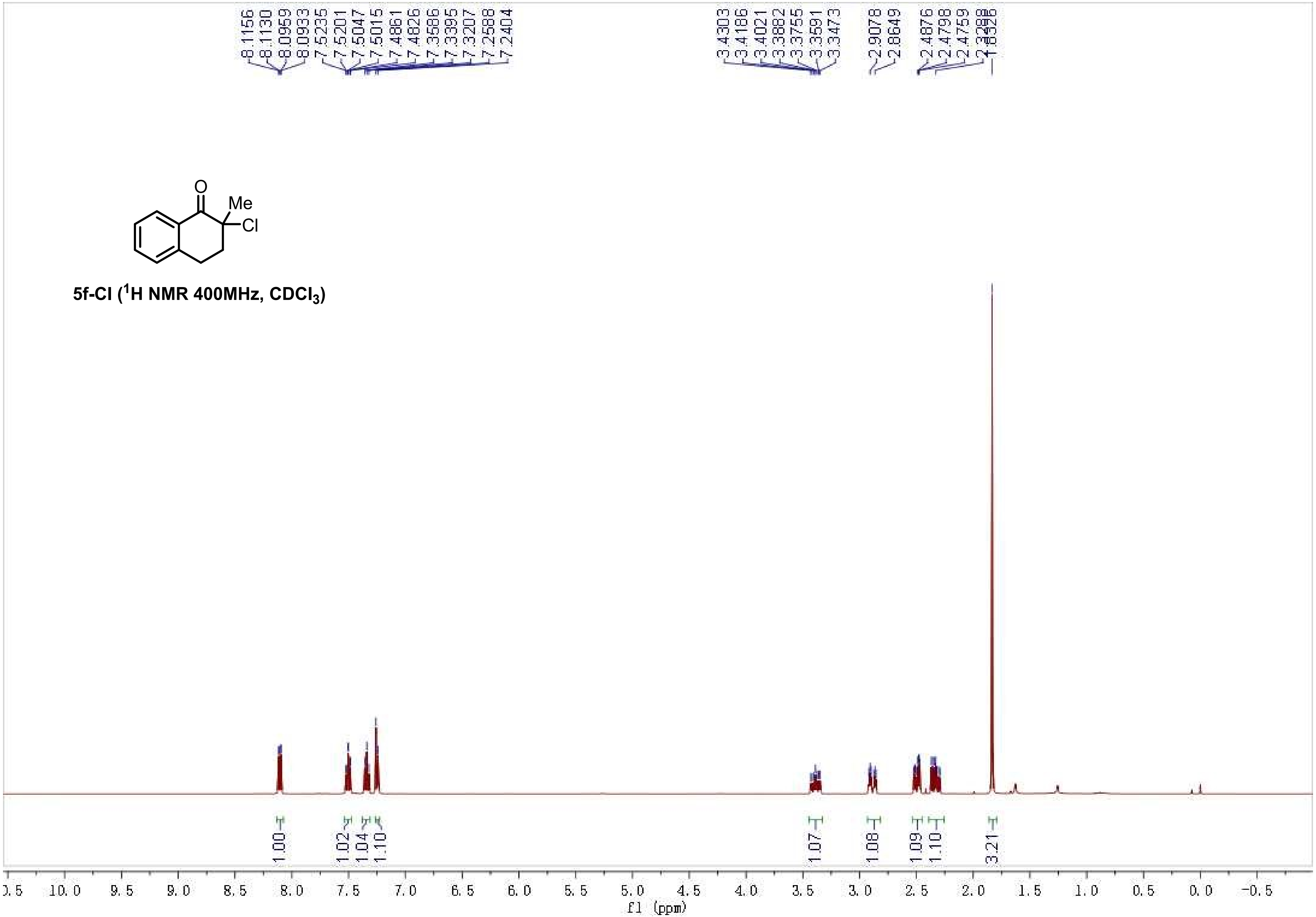

5f-Cl (^{1}H NMR 400MHz, $CDCl_3$)

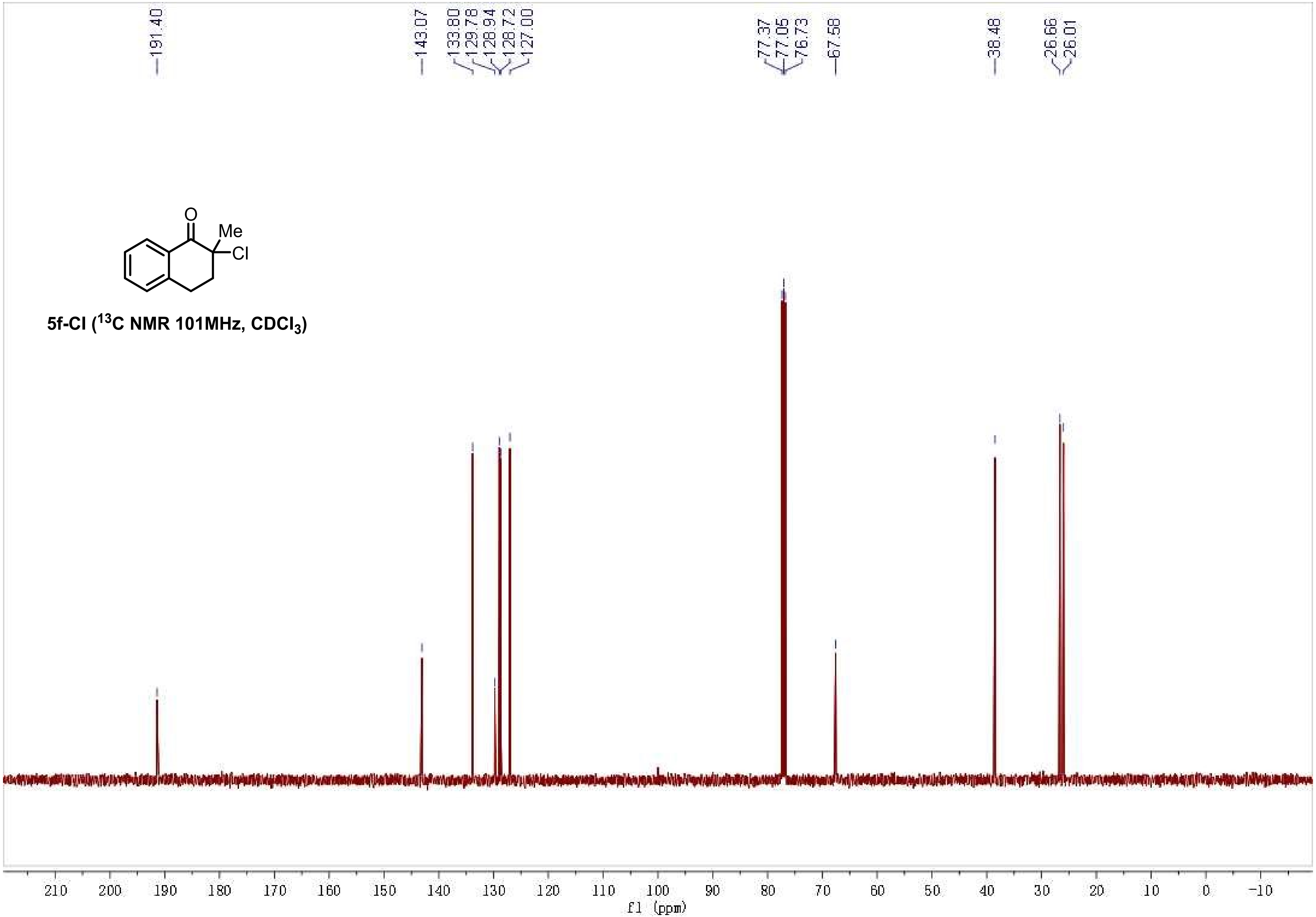

5f-Cl (^{13}C NMR 101MHz, $CDCl_3$)

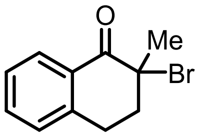

5f-Br (^1H NMR 400MHz, $CDCl_3$)

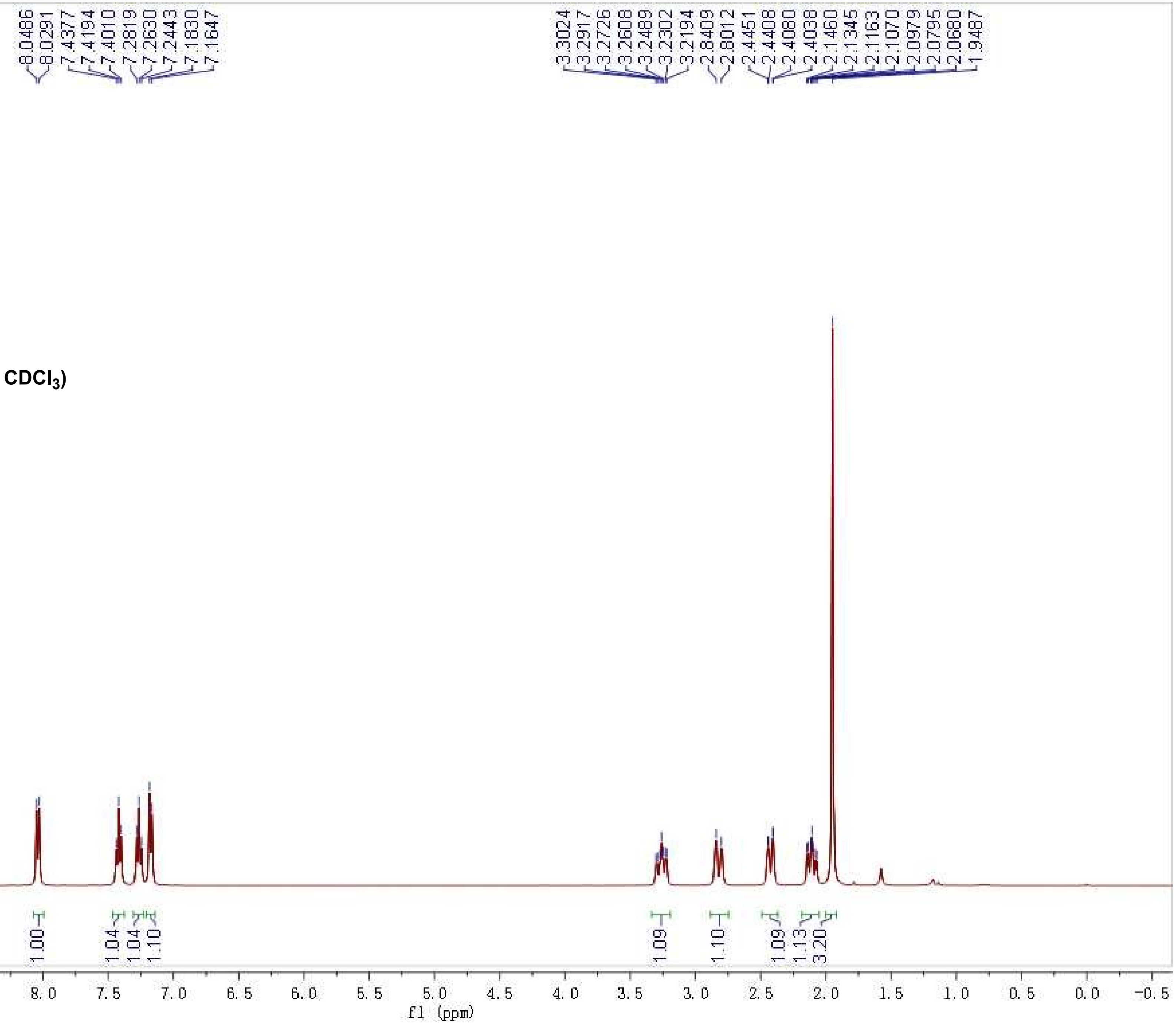

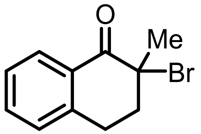

5f-Br (^{13}C NMR 101MHz, $CDCl_3$)

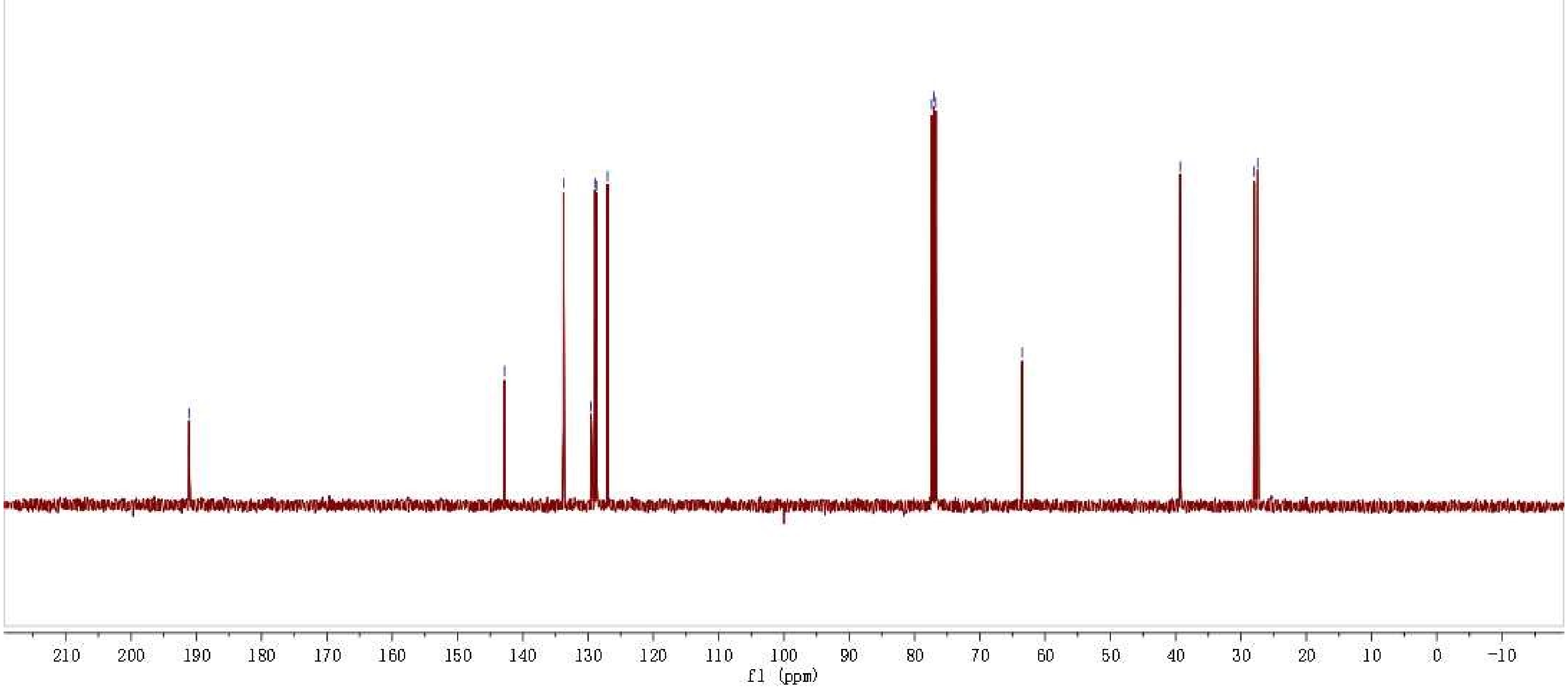

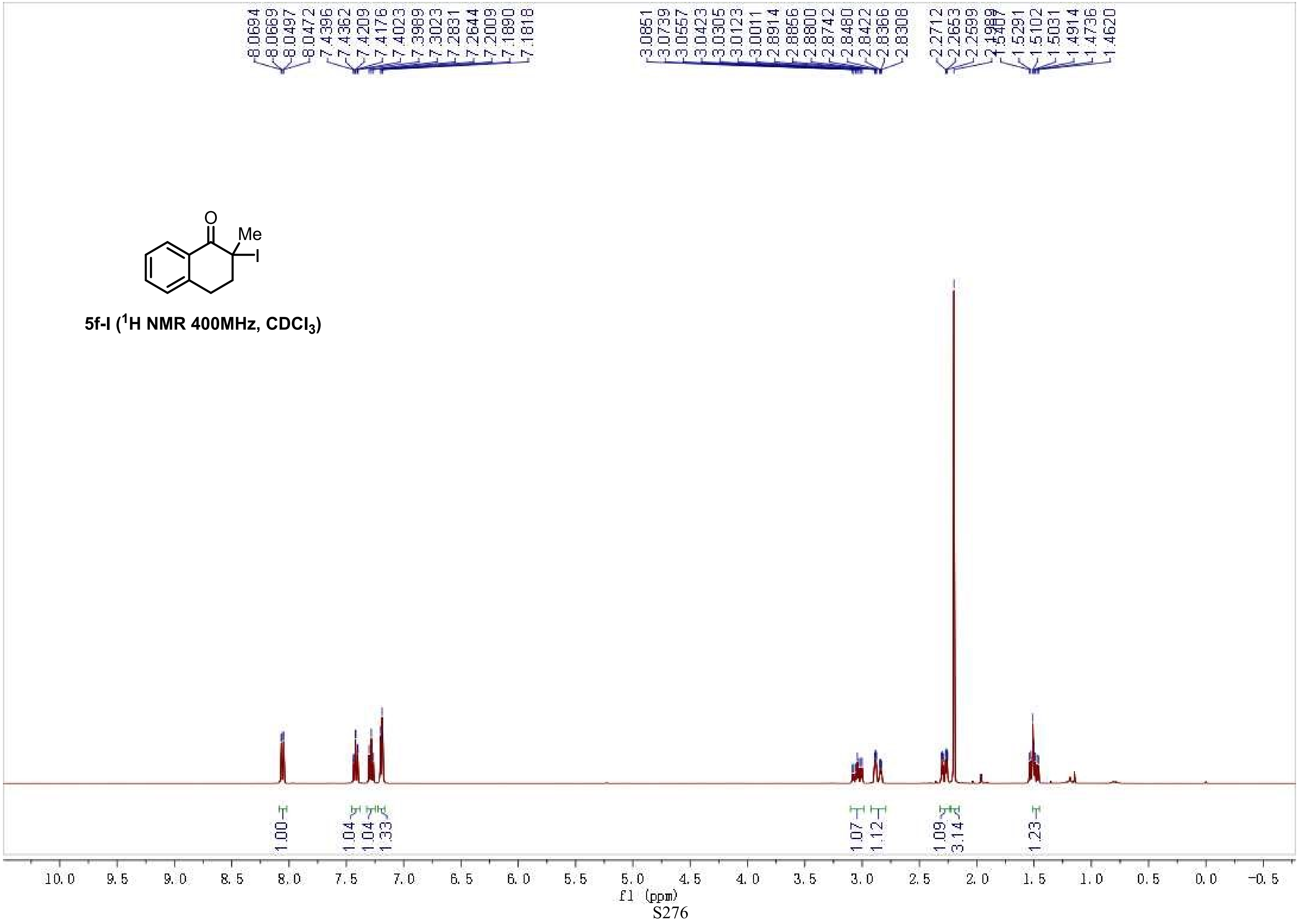

5f-I (^{1}H NMR 400MHz, $CDCl_3$)

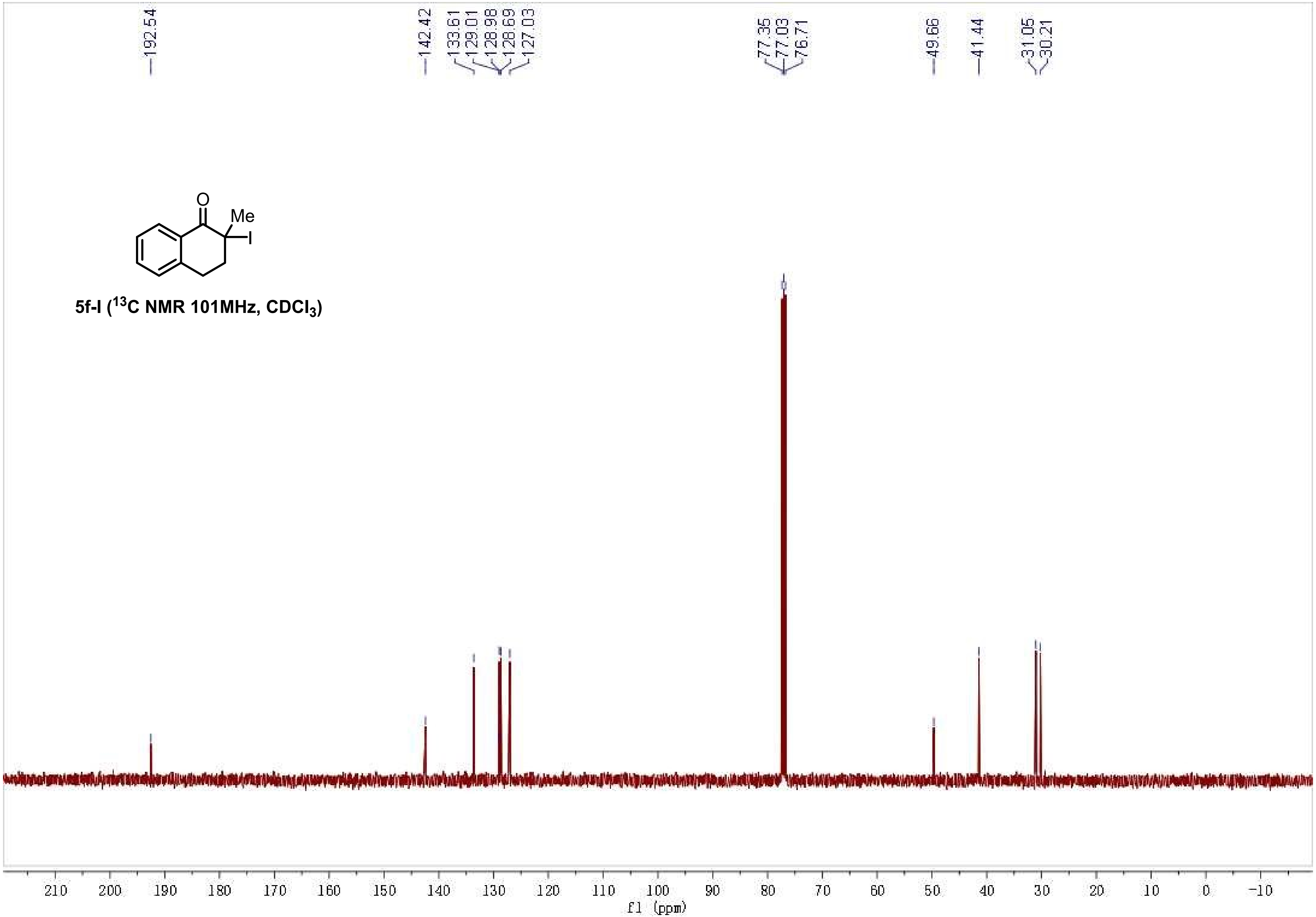

192.54
142.42
133.61
129.01
128.98
128.69
127.03
77.35
77.03
76.71
49.66
41.44
31.05
30.21
Me
O
I
5f-I (^{13}C NMR 101MHz, $CDCl_3$)
210 200 190 180 170 160 150 140 130 120 110 100 90 80 70 60 50 40 30 20 10 0 -10
f1 (ppm)

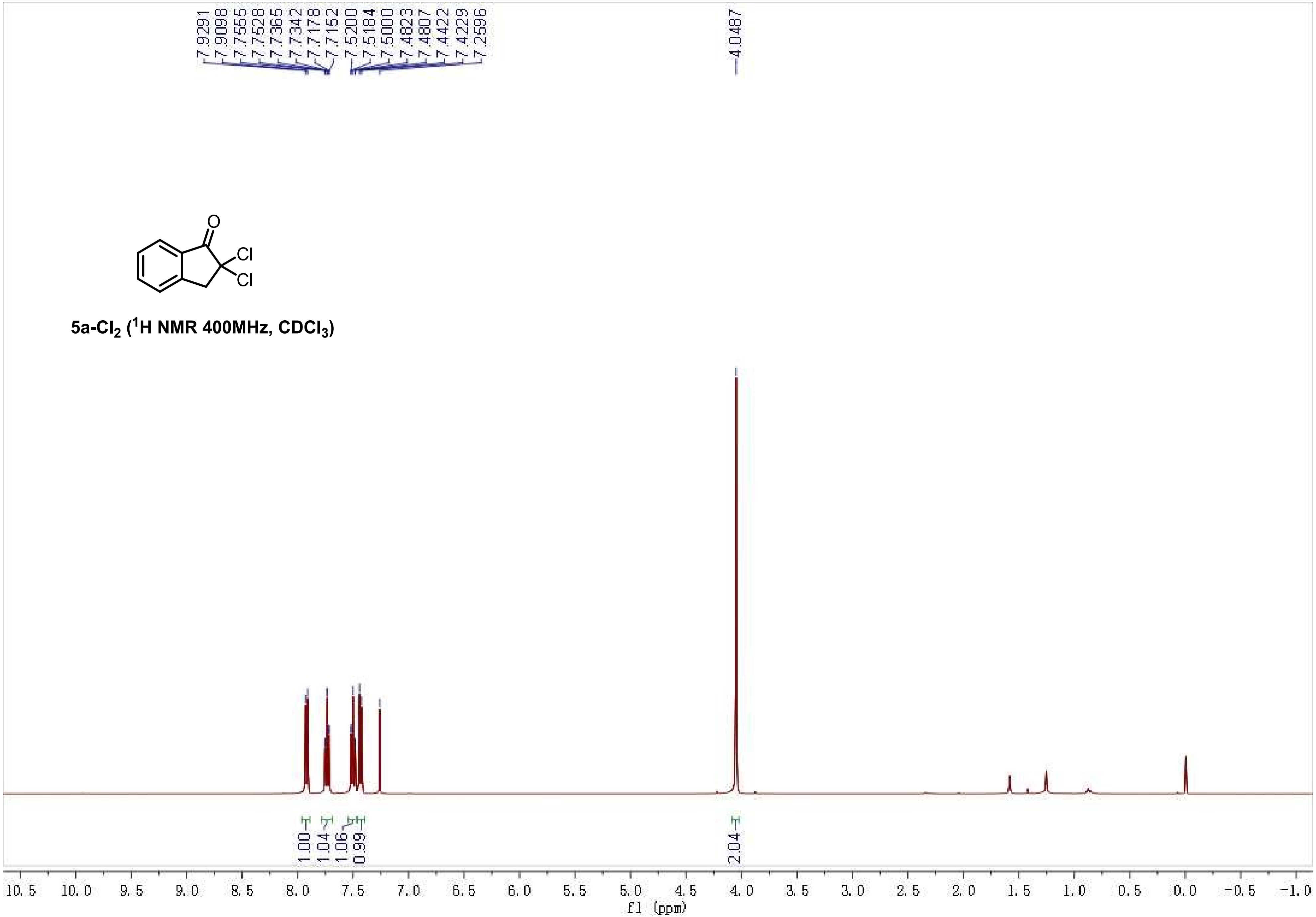
7.9291
7.9098
7.7555
7.7528
7.7365
7.7342
7.7178
7.7152
7.5200
7.5184
7.5000
7.4823
7.4807
7.4422
7.4229
7.2596
4.0487
O
Cl
Cl
5a-Cl2 (1H NMR 400MHz, CDCl3)
1.00
1.04
1.06
0.99
2.04
10.5 10.0 9.5 9.0 8.5 8.0 7.5 7.0 6.5 6.0 5.5 5.0 4.5 4.0 3.5 3.0 2.5 2.0 1.5 1.0 0.5 0.0 -0.5 -1.0
f1 (ppm)

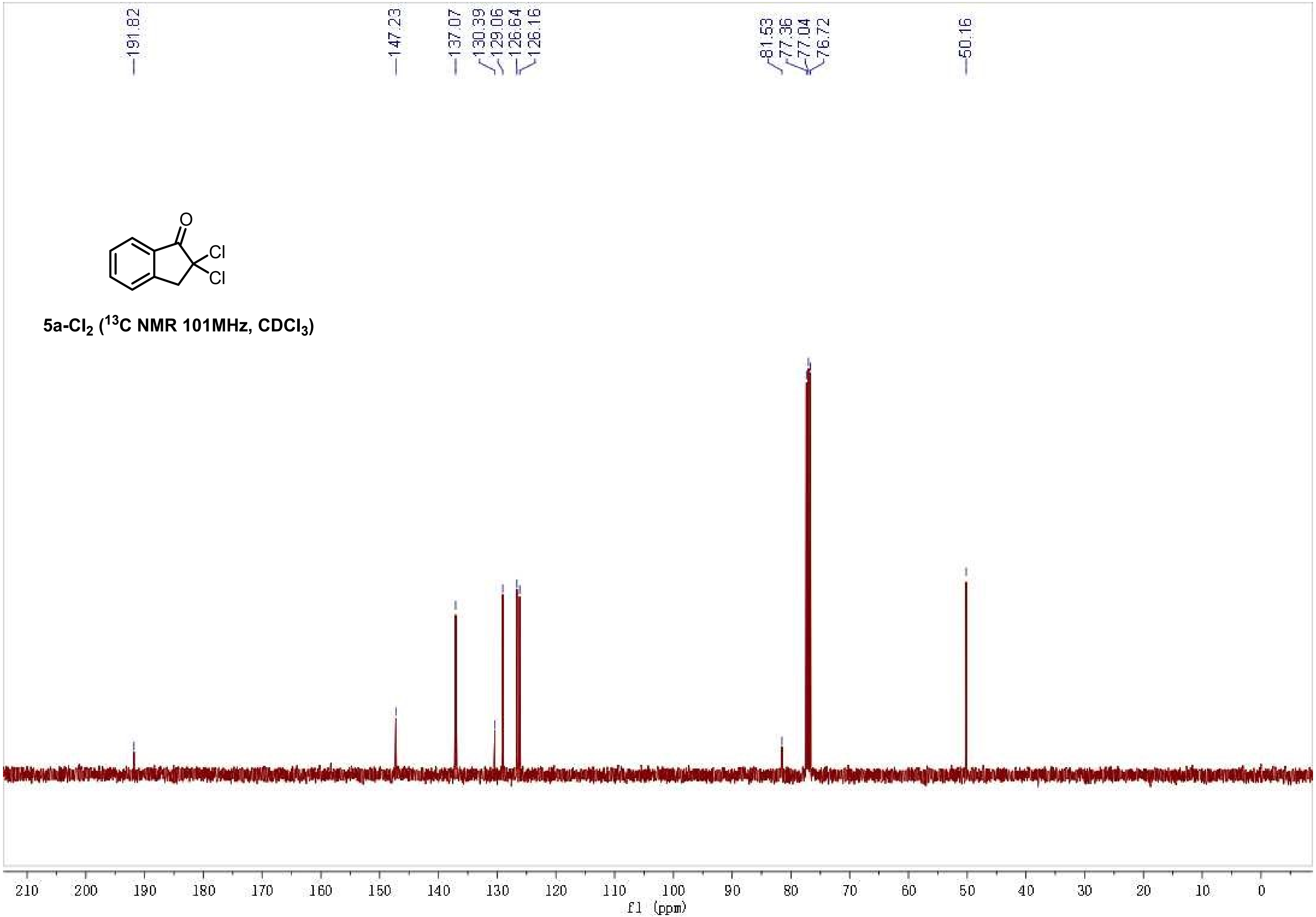

5a-Cl$_2$ (^{13}C NMR 101MHz, CDCl$_3$)

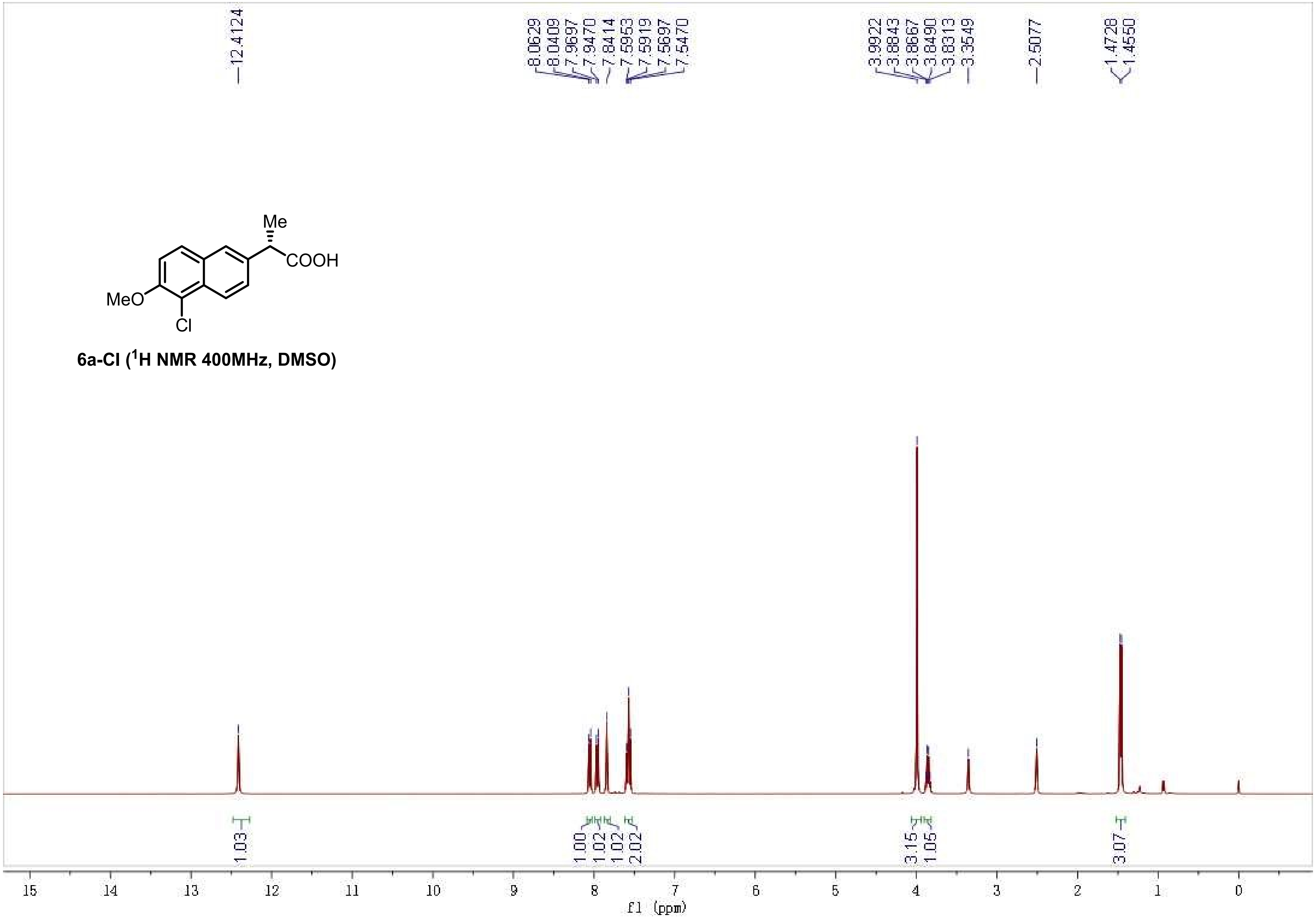

6a-Cl (^{1}H NMR 400MHz, DMSO)

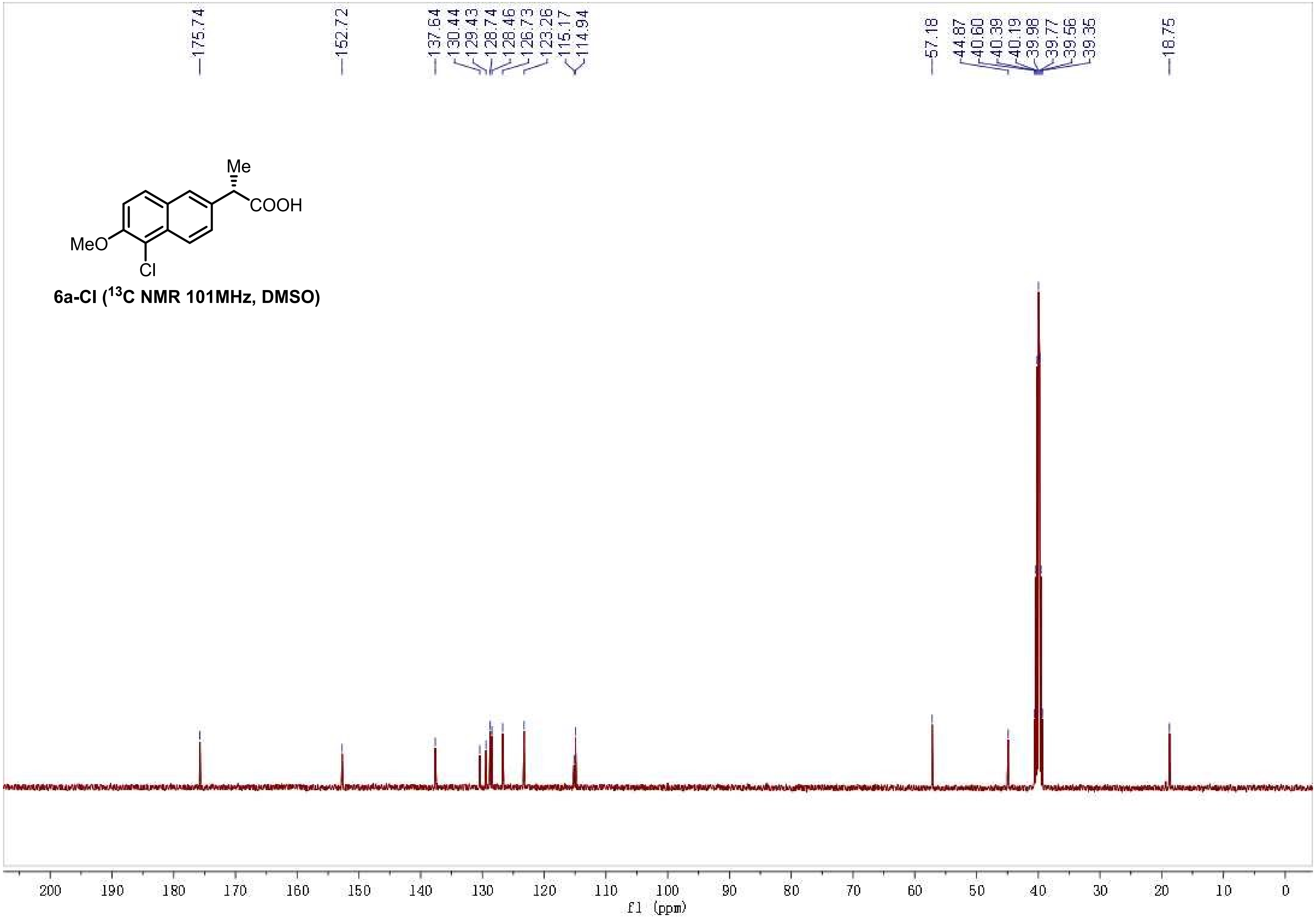

6a-Cl (^{13}C NMR 101MHz, DMSO)

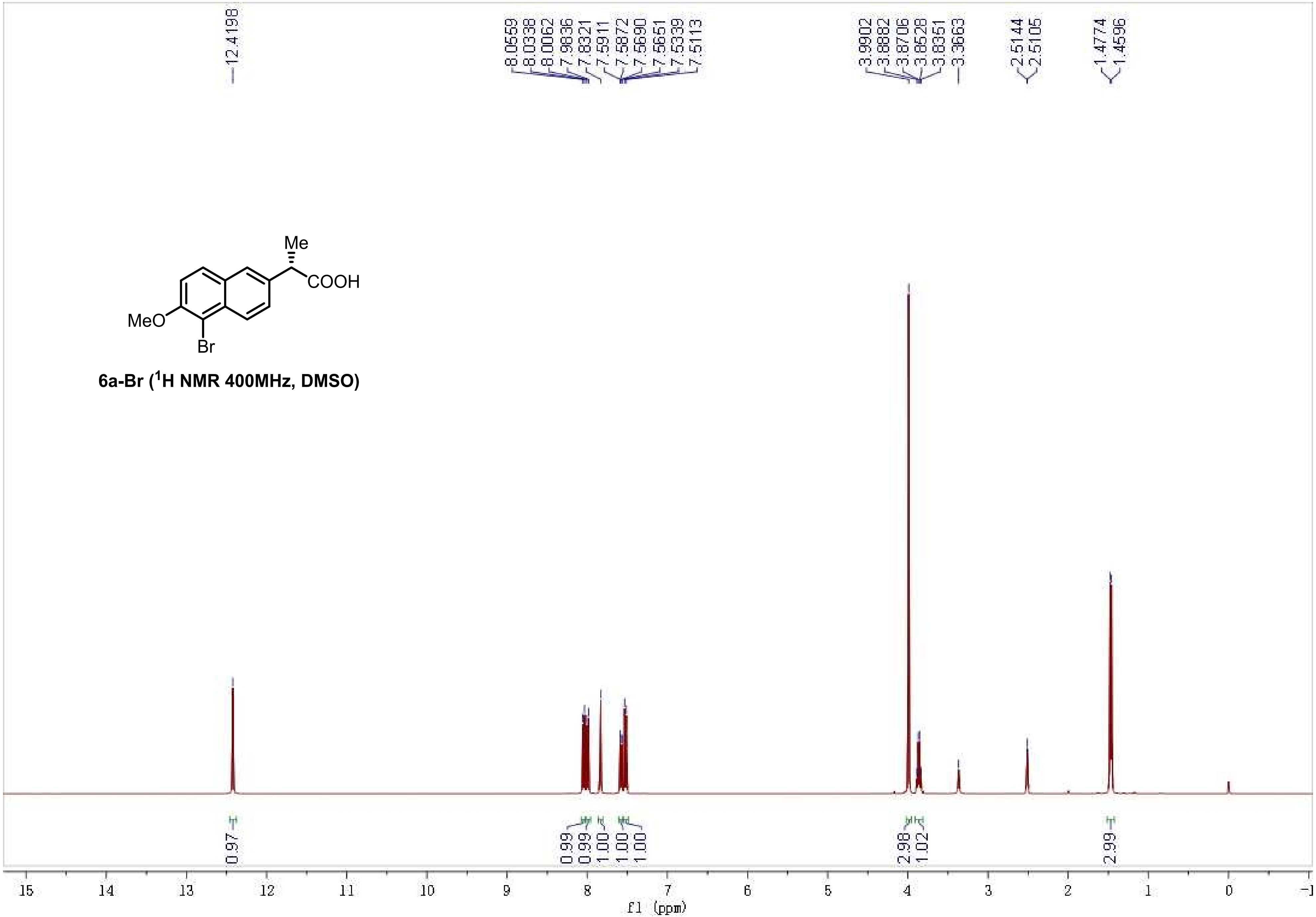

6a-Br (^{1}H NMR 400MHz, DMSO)

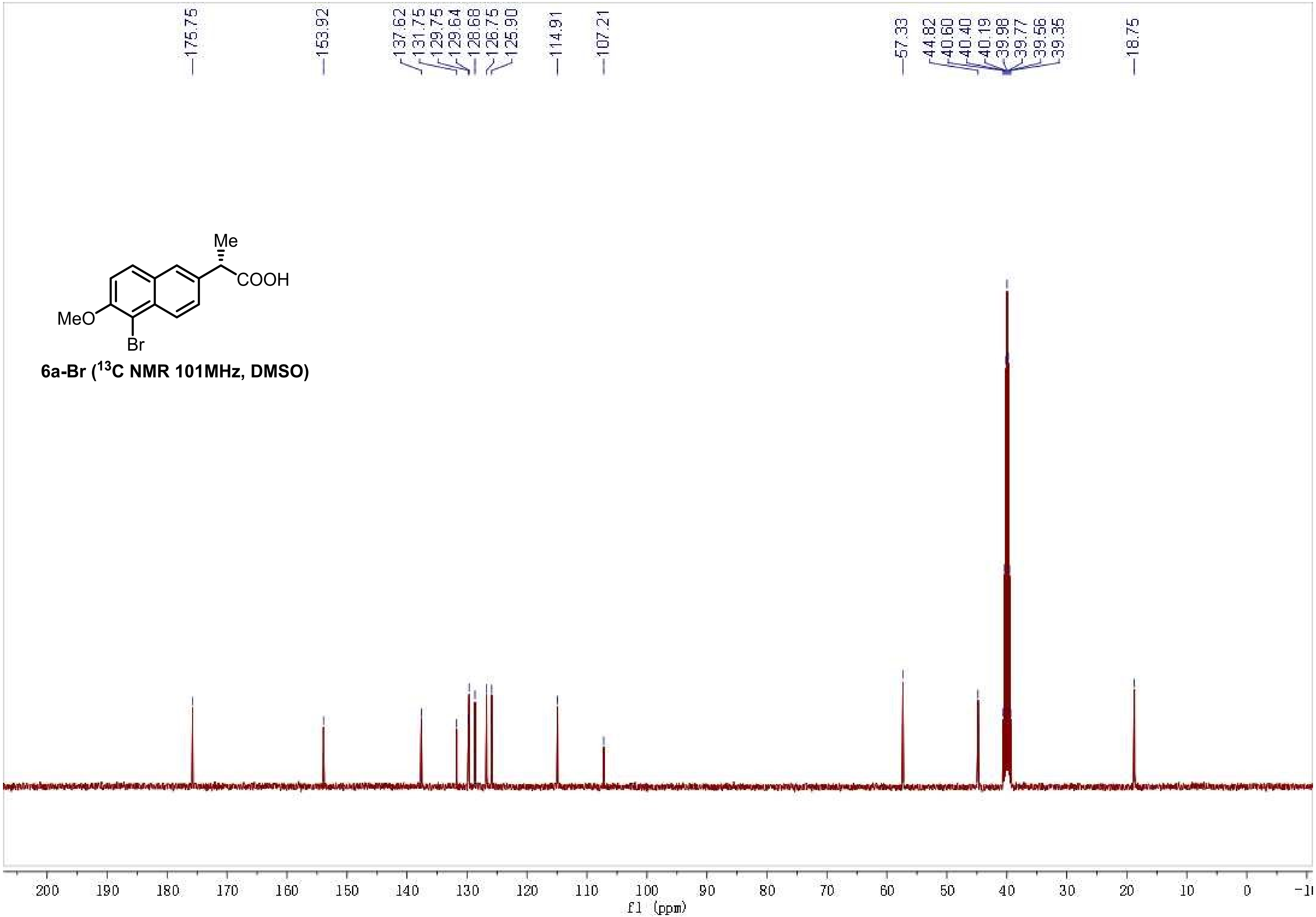

6a-Br (^{13}C NMR 101MHz, DMSO)

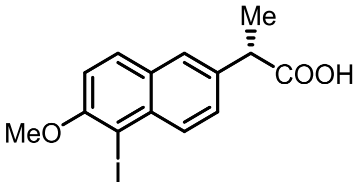

6a-I (^{1}H NMR 400MHz, DMSO)

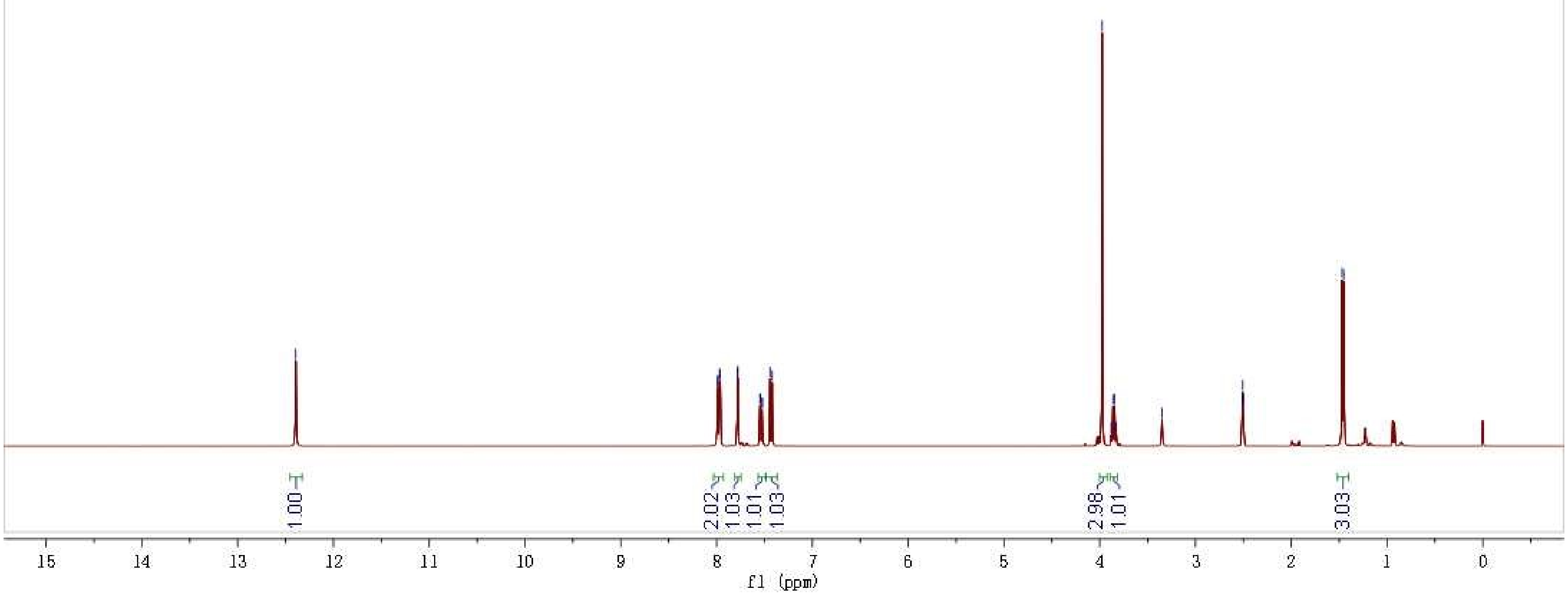

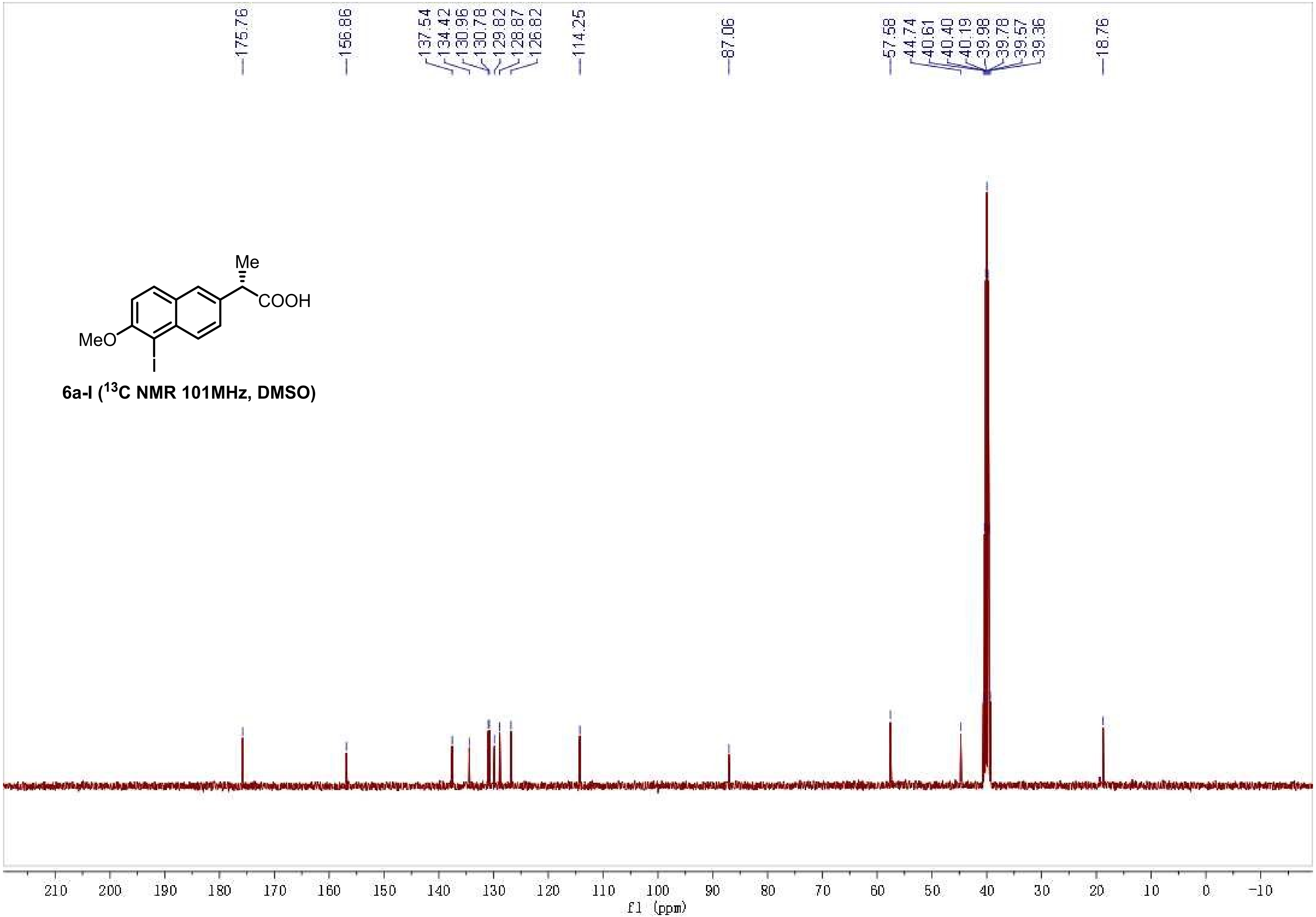

175.76
156.86
137.54
134.42
130.96
130.78
129.82
128.87
126.82
114.25
87.06
57.58
44.74
40.61
40.40
40.19
39.98
39.78
39.57
39.36
18.76
Me
COOH
MeO
I
6a-I (^{13}C NMR 101MHz, DMSO)
210 200 190 180 170 160 150 140 130 120 110 100 90 80 70 60 50 40 30 20 10 0 -10
f1 (ppm)

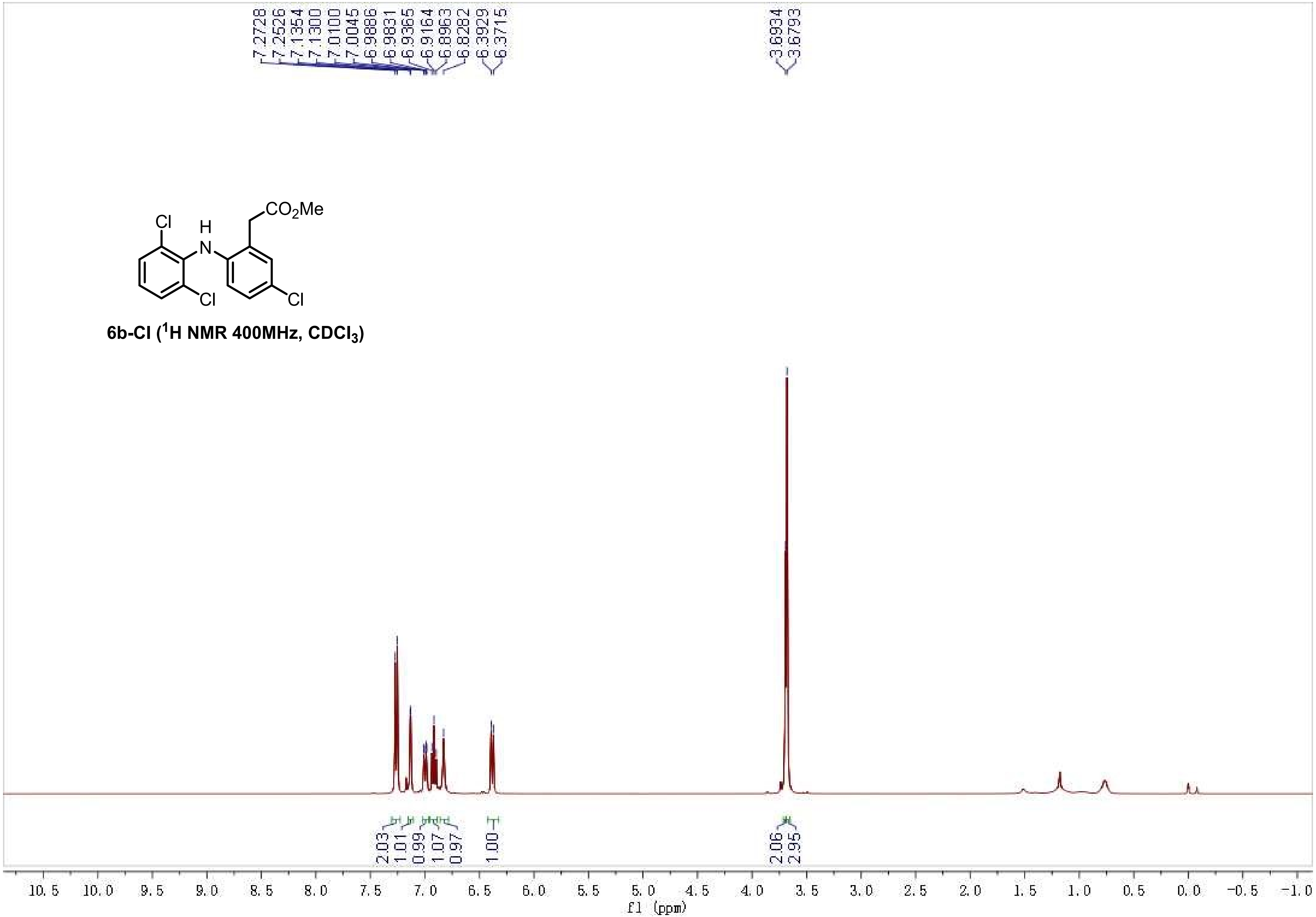

6b-Cl (^{1}H NMR 400MHz, $CDCl_3$)

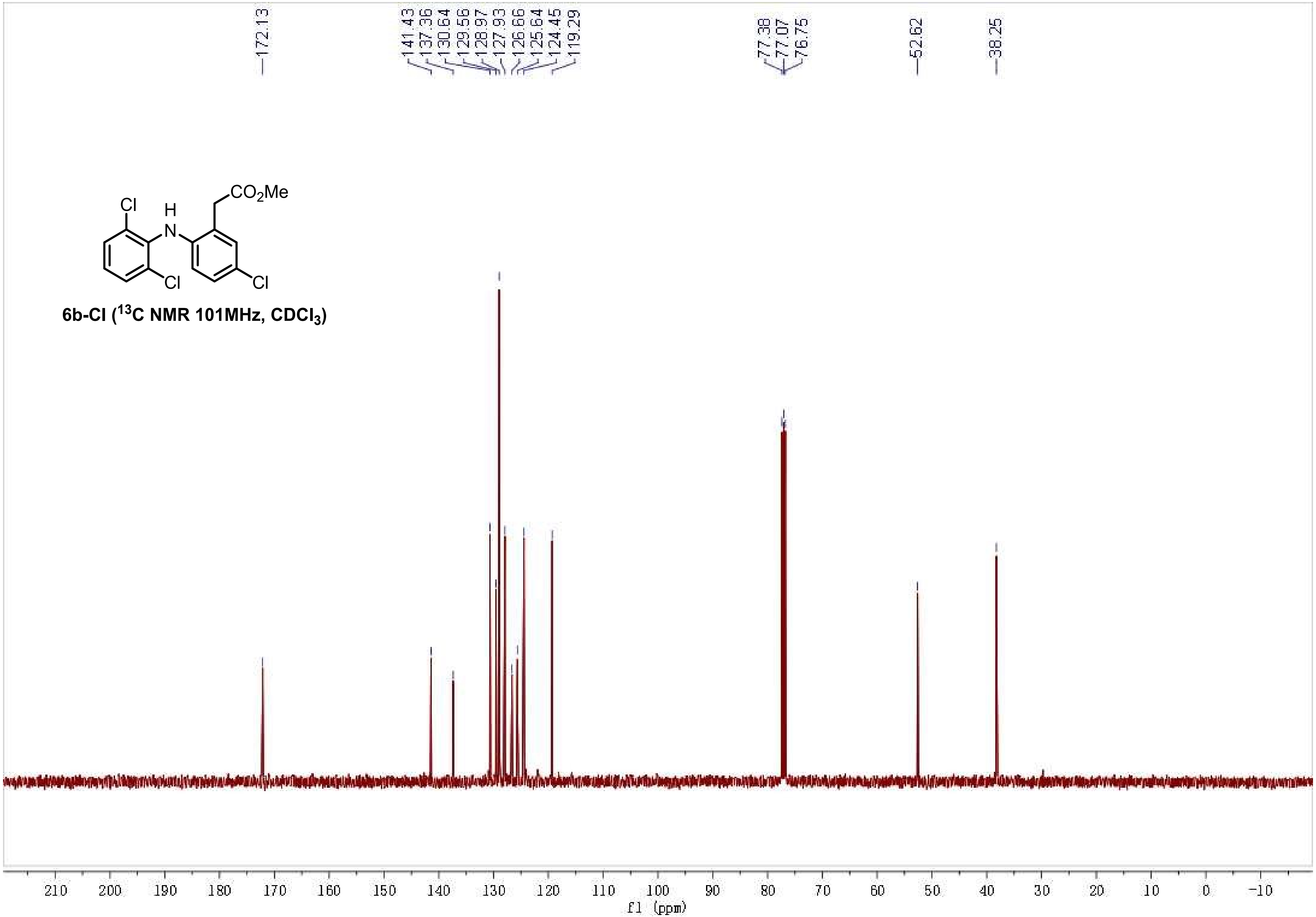

6b-Cl (^{13}C NMR 101MHz, $CDCl_3$)

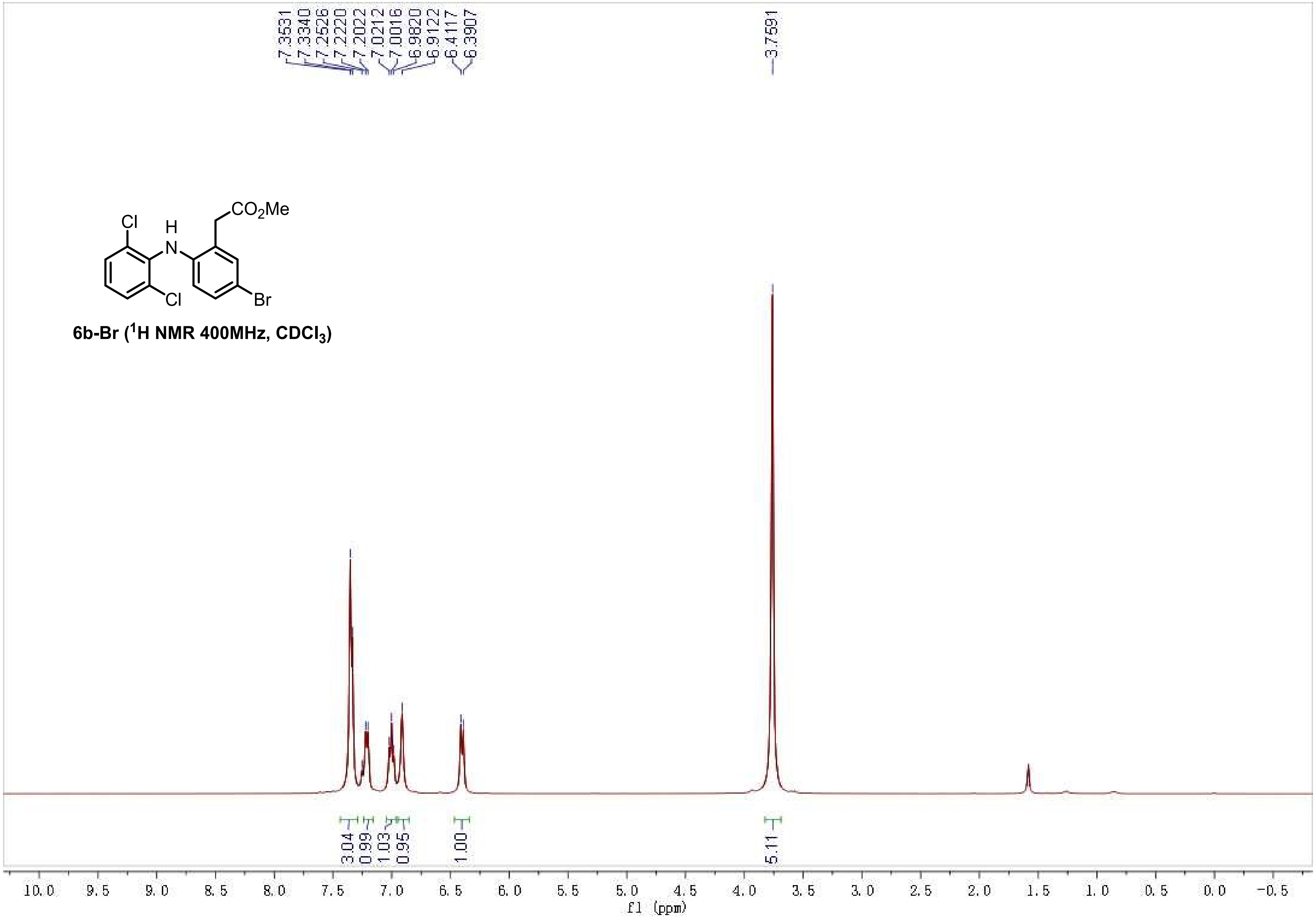

6b-Br (^{1}H NMR 400MHz, $CDCl_3$)

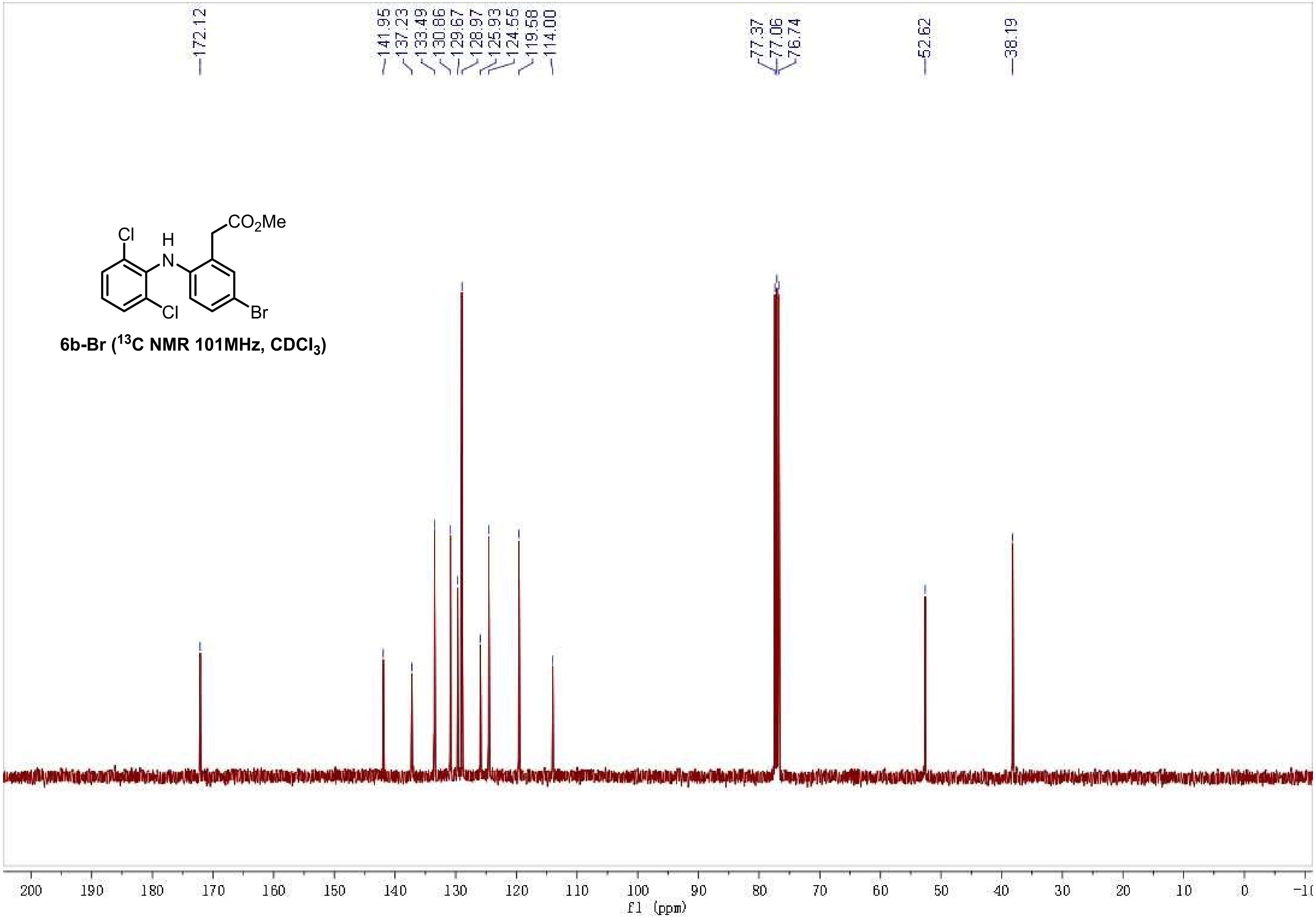

6b-Br (^{13}C NMR 101MHz, $CDCl_3$)

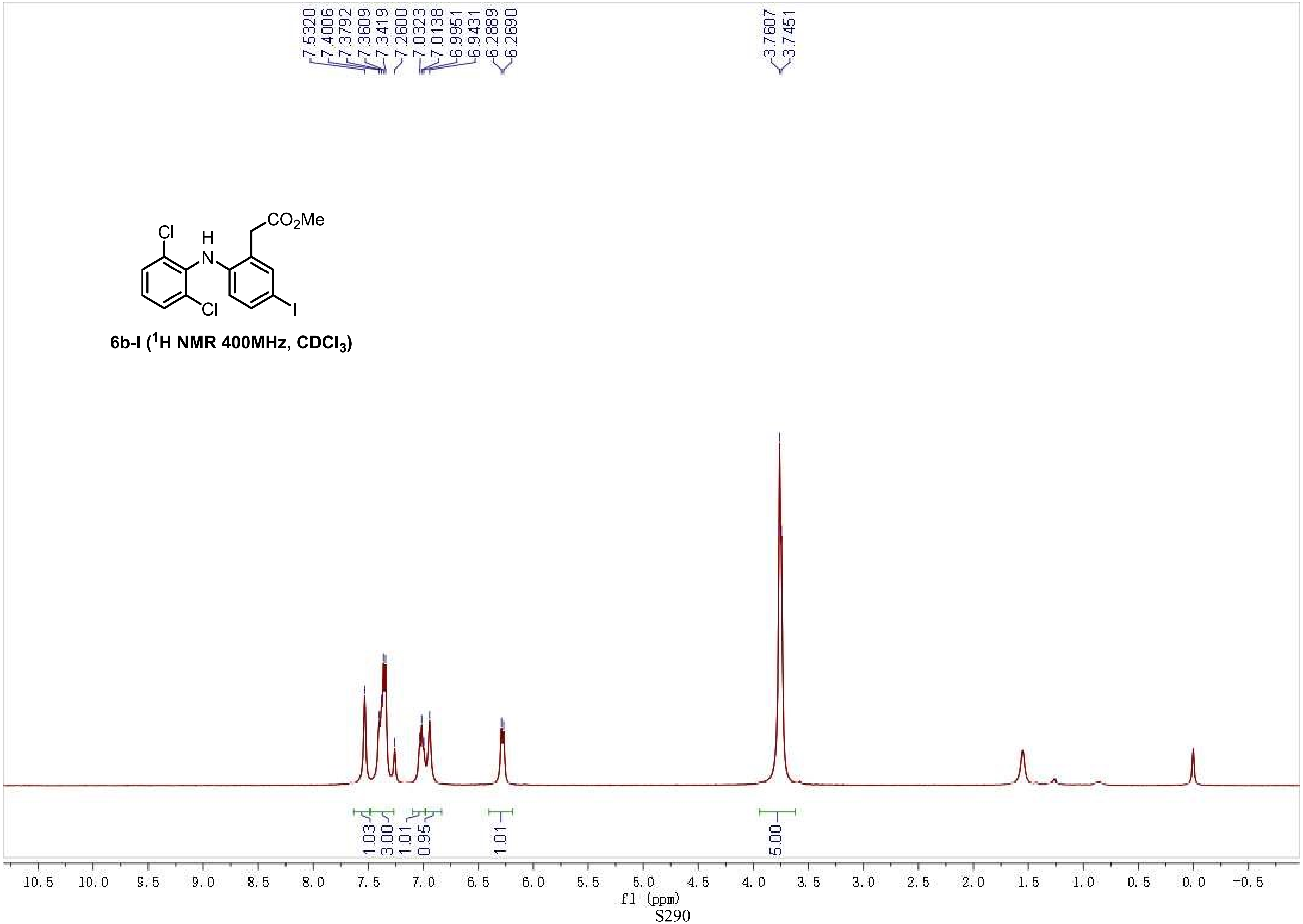

6b-I (^{1}H NMR 400MHz, $CDCl_3$)

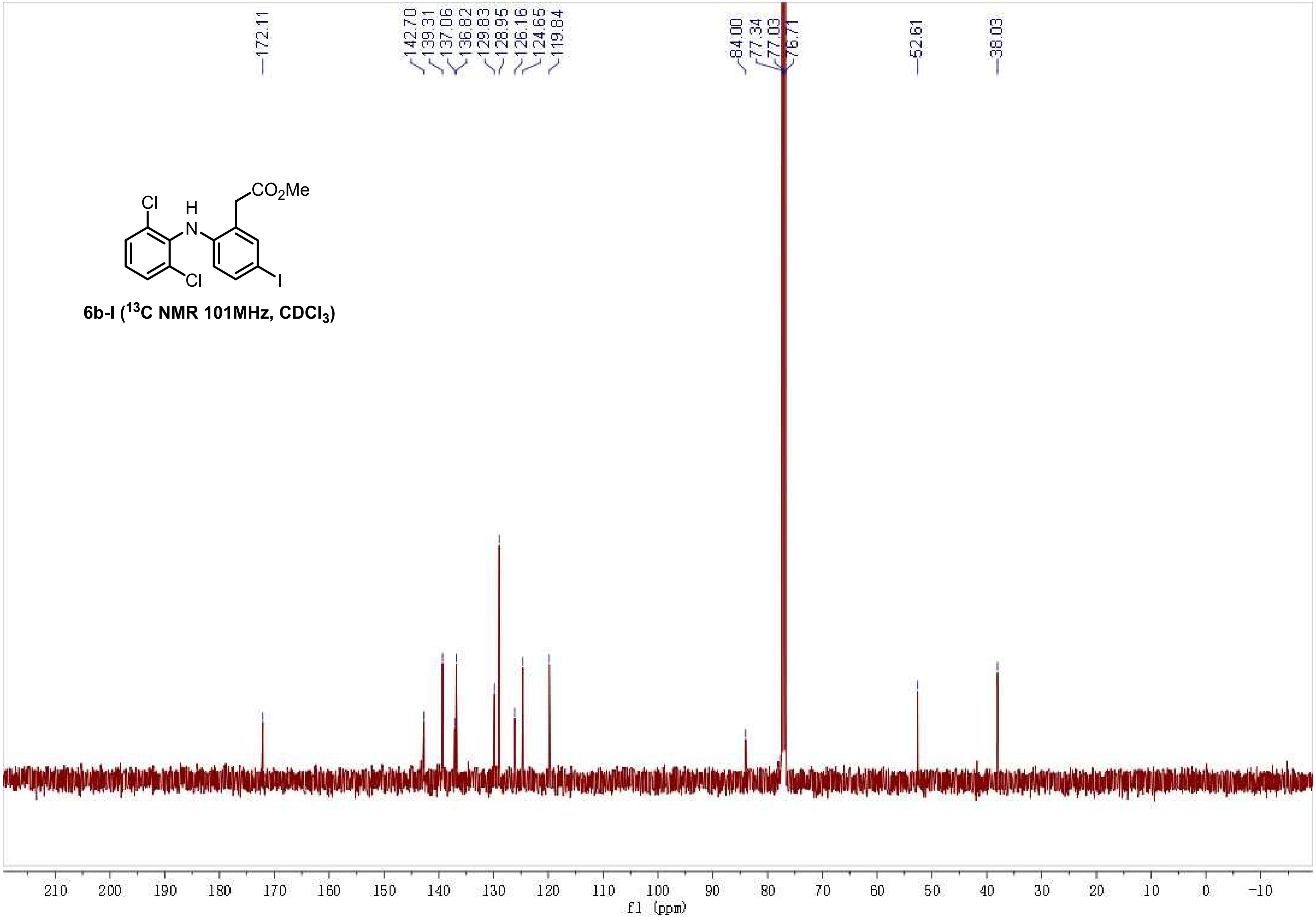

6b-I (^{13}C NMR 101MHz, $CDCl_3$)

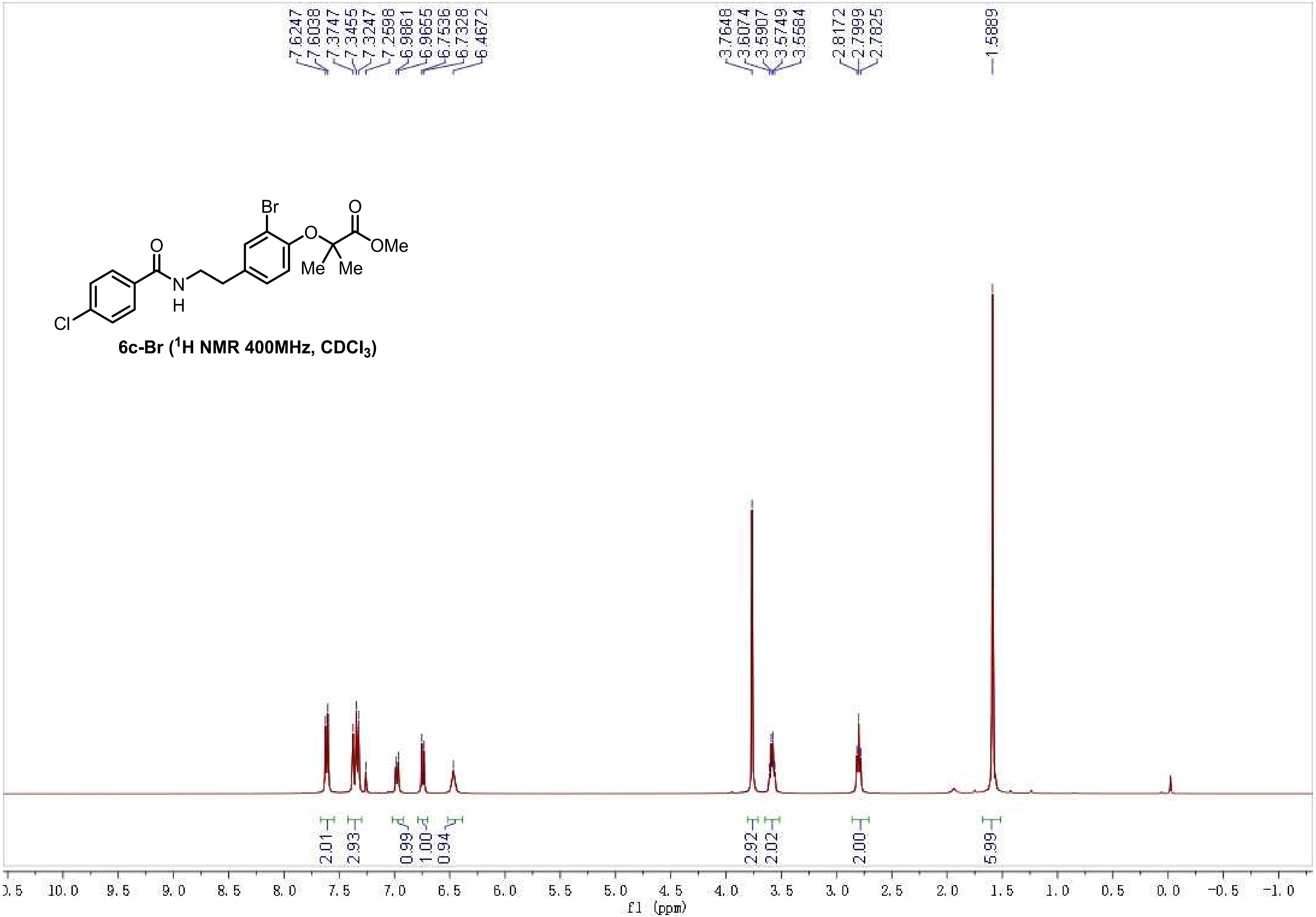

7.6247
7.6038
7.3747
7.3455
7.3247
7.2598
6.9861
6.9655
6.7536
6.7328
6.4672
3.7648
3.6074
3.5907
3.5749
3.5584
2.8172
2.7999
2.7825
1.5889
Br
O
O
OMe
Me
Me
N
H
Cl
6c-Br (1H NMR 400MHz, CDCl3)
2.01
2.93
0.99
1.00
0.94
2.92
2.02
2.00
5.99
f1 (ppm)

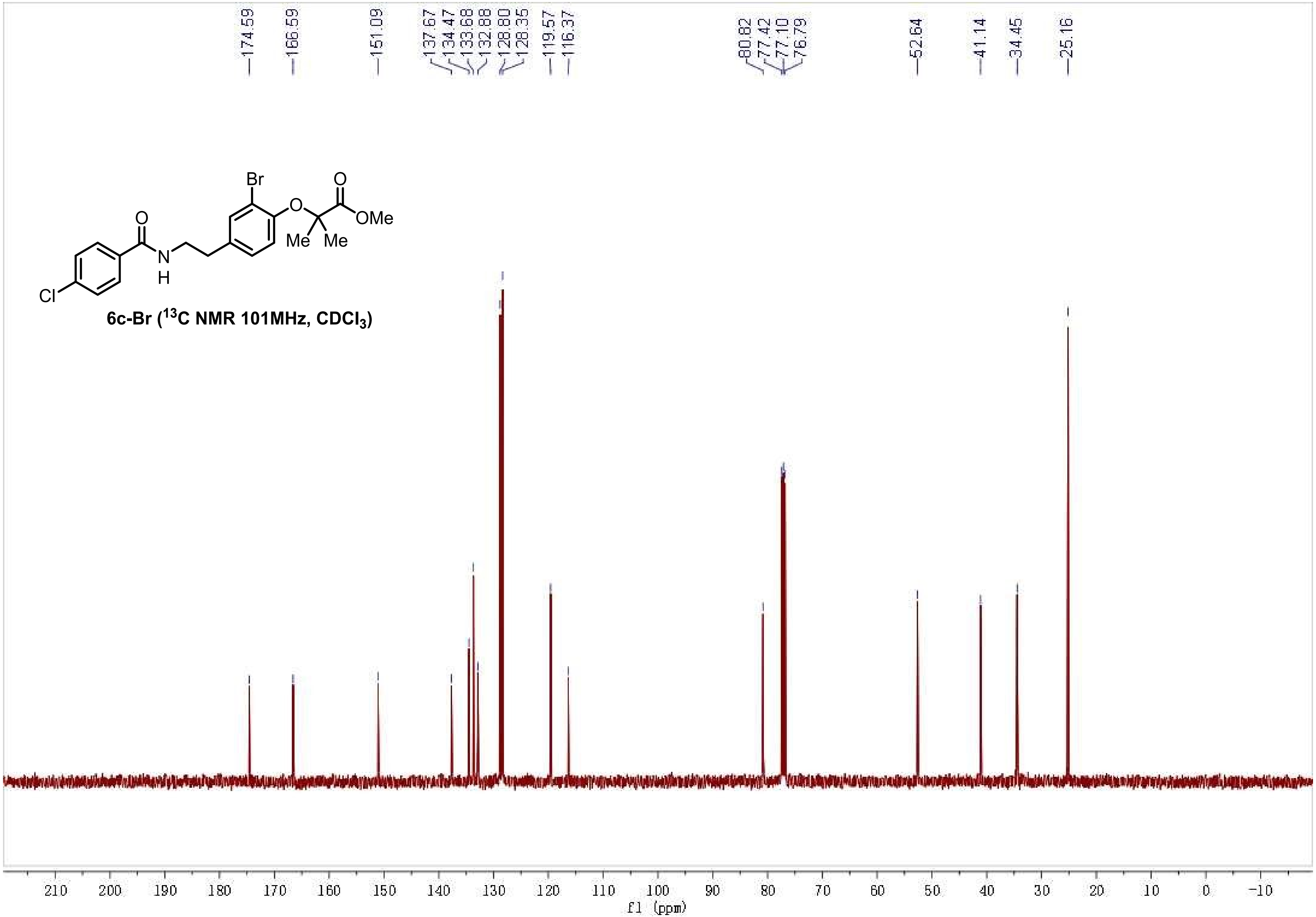

6c-Br (^{13}C NMR 101MHz, $CDCl_3$)

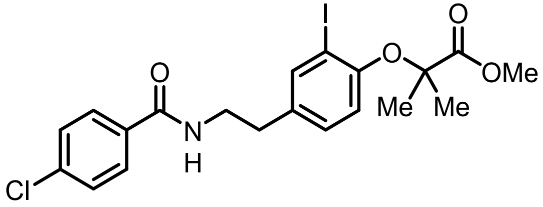

6c-I (^{1}H NMR 400MHz, $CDCl_3$)

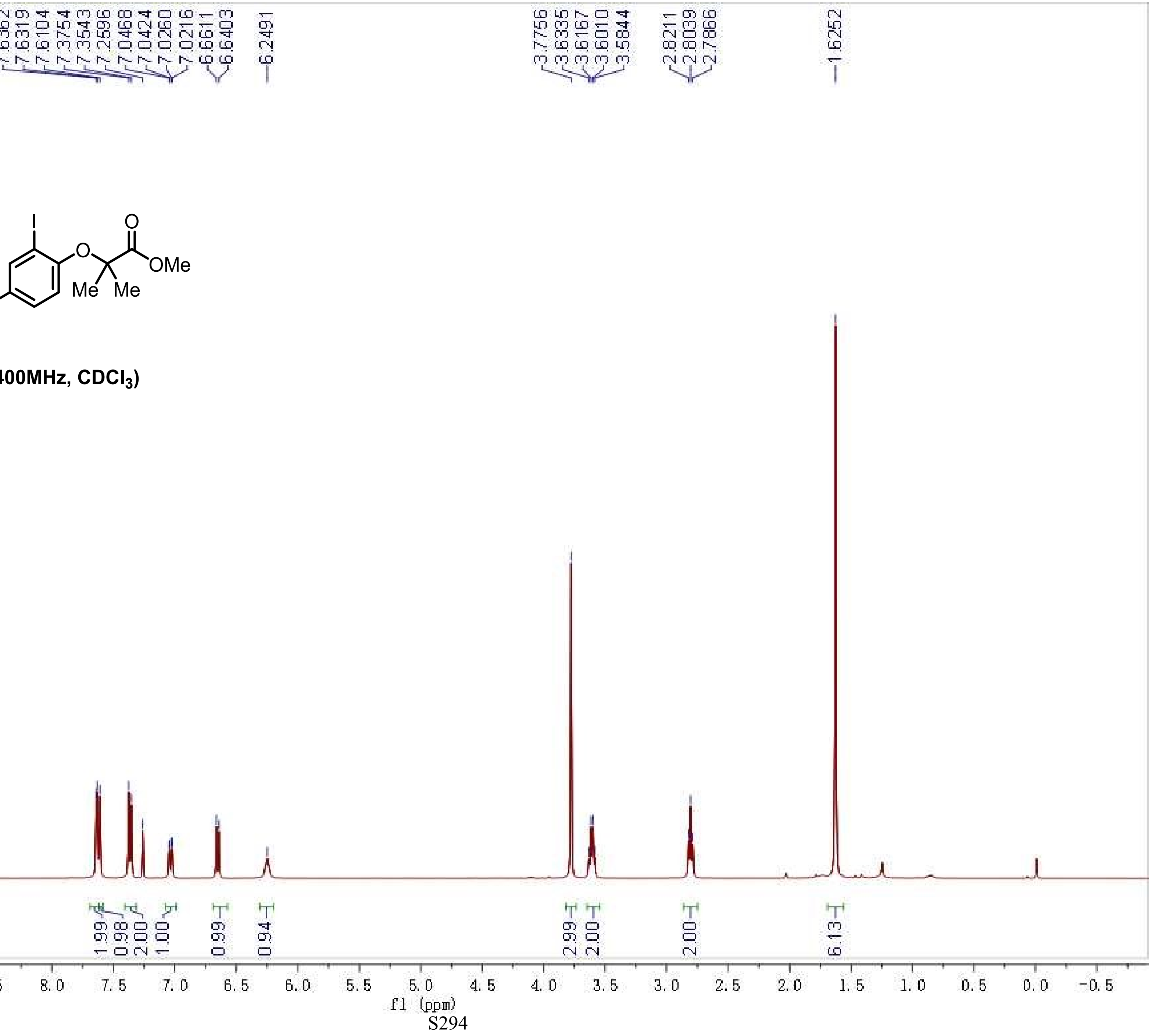

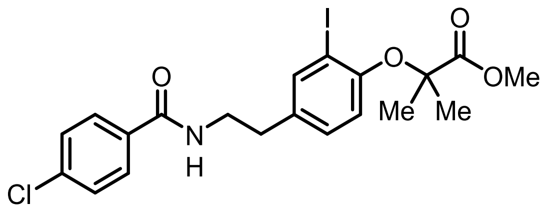

6c-I (^{13}C NMR 101MHz, $CDCl_3$)

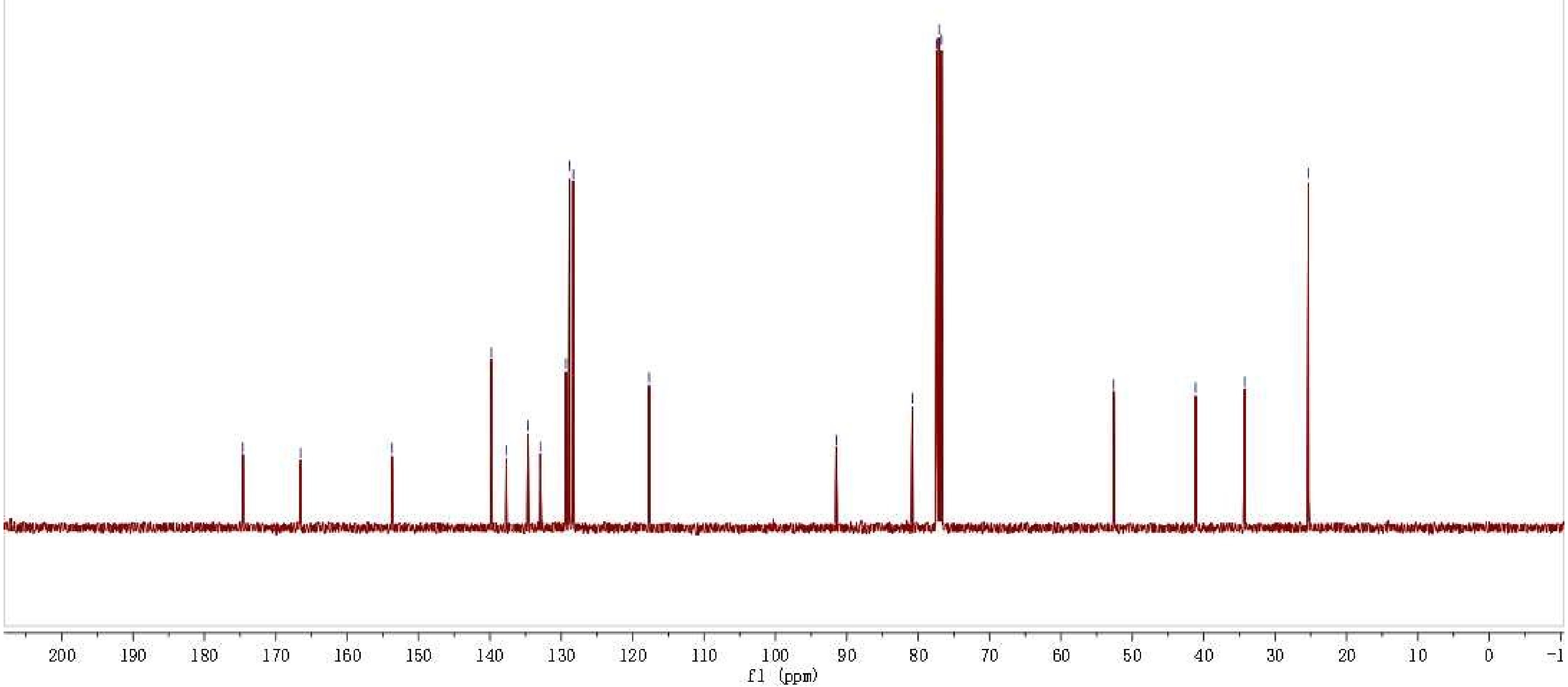

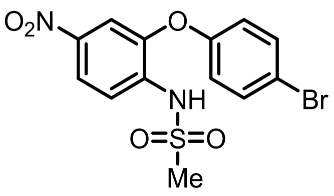

6d-Br (^{1}H NMR 400MHz, $CDCl_3$)

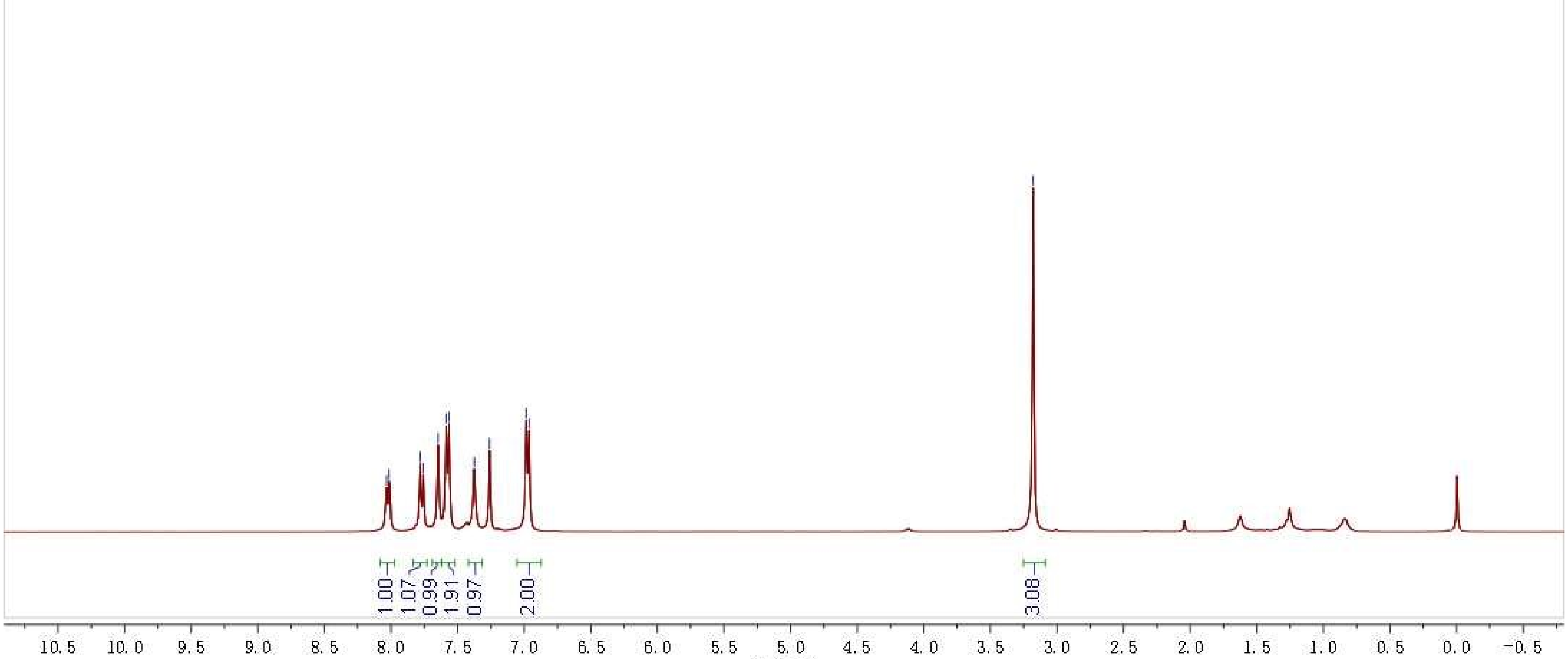

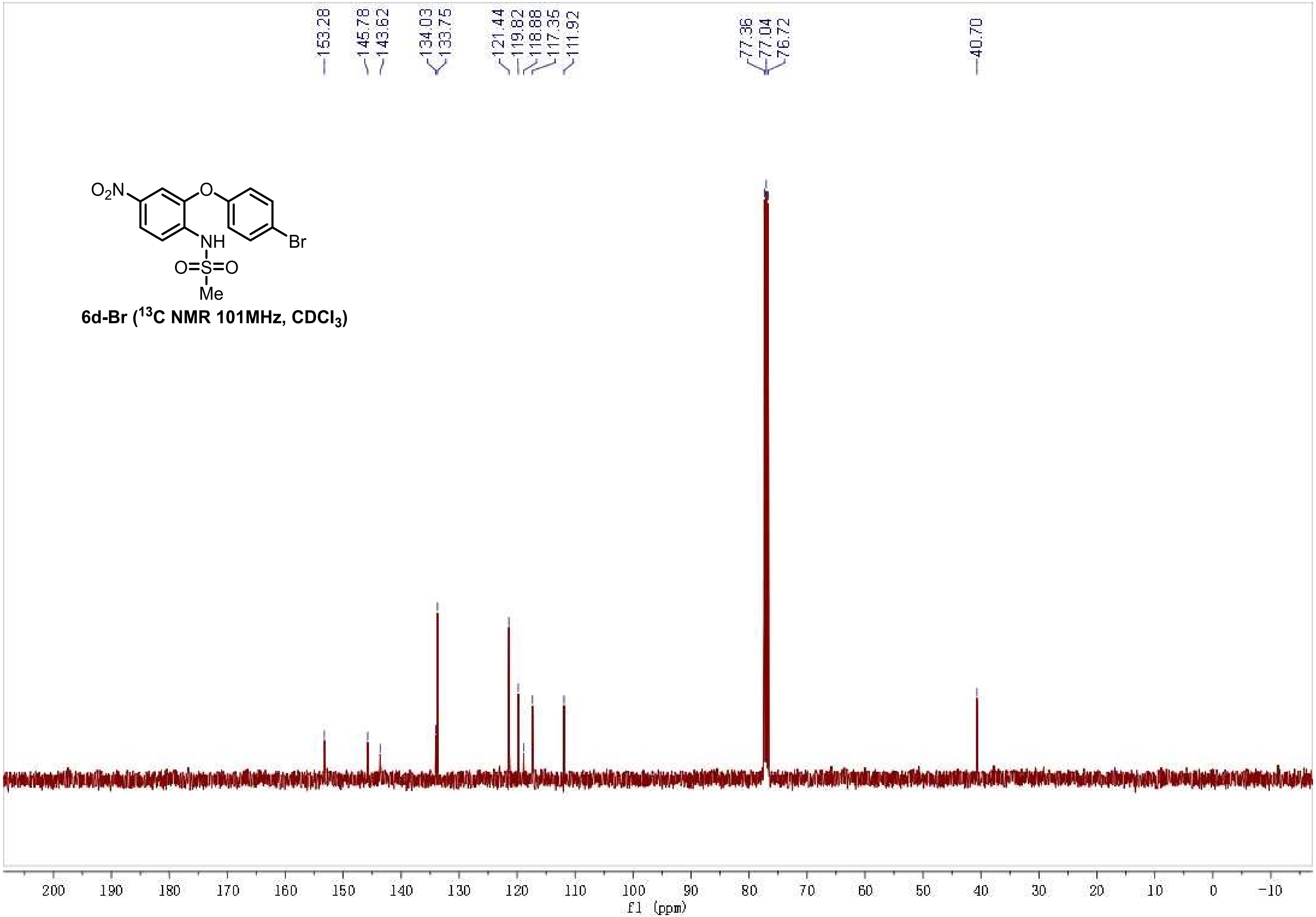

6d-Br (^{13}C NMR 101MHz, $CDCl_3$)

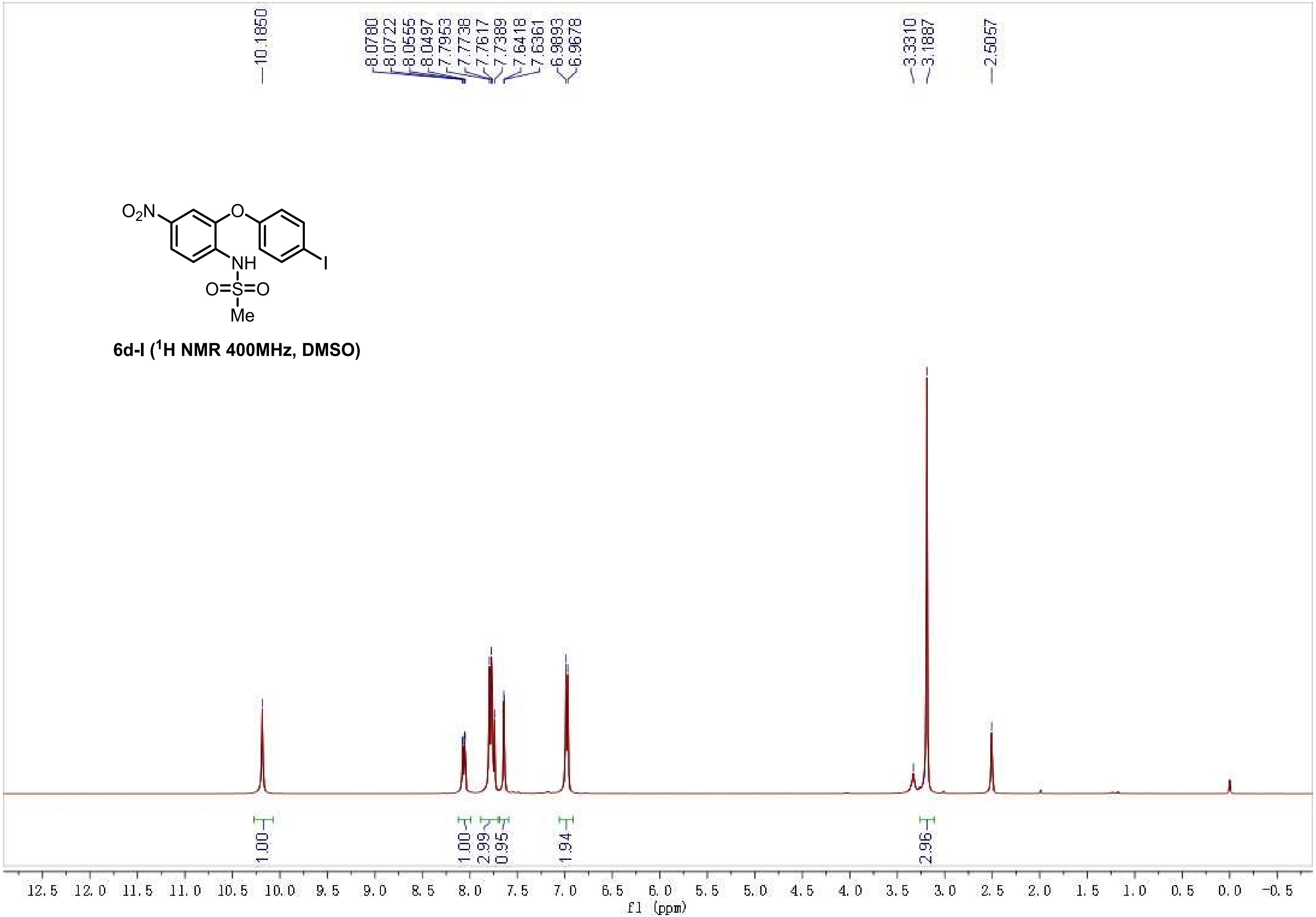
10.1850
8.0780
8.0722
8.0555
8.0497
7.7953
7.7738
7.7617
7.7389
7.6418
7.6361
6.9893
6.9678
3.3310
3.1887
2.5057
O2N
O
NH
I
O=S=O
Me
6d-I (1H NMR 400MHz, DMSO)
1.00
1.00
2.99
0.95
1.94
2.96
12.5 12.0 11.5 11.0 10.5 10.0 9.5 9.0 8.5 8.0 7.5 7.0 6.5 6.0 5.5 5.0 4.5 4.0 3.5 3.0 2.5 2.0 1.5 1.0 0.5 0.0 -0.5
f1 (ppm)

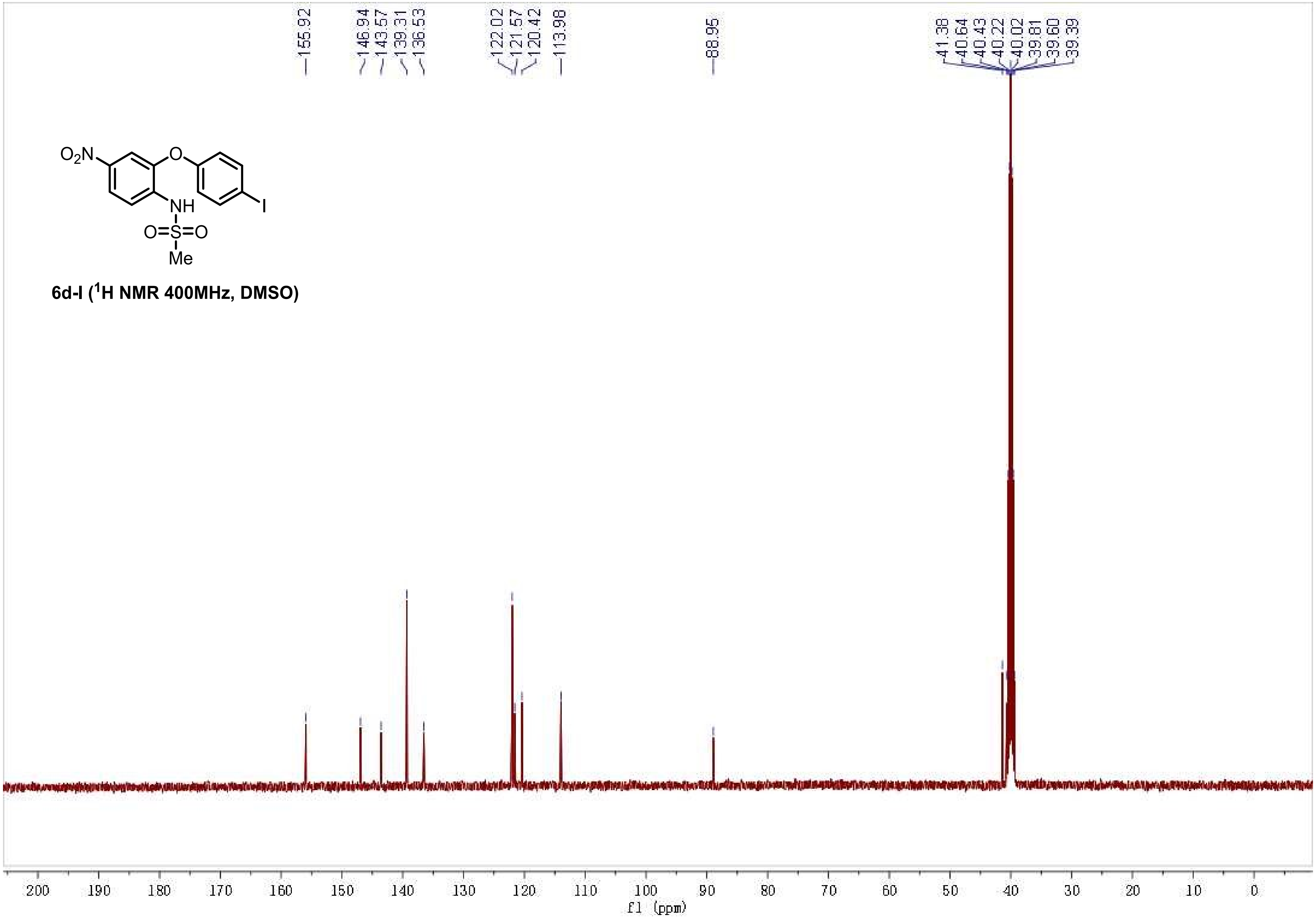

6d-I (^{1}H NMR 400MHz, DMSO)

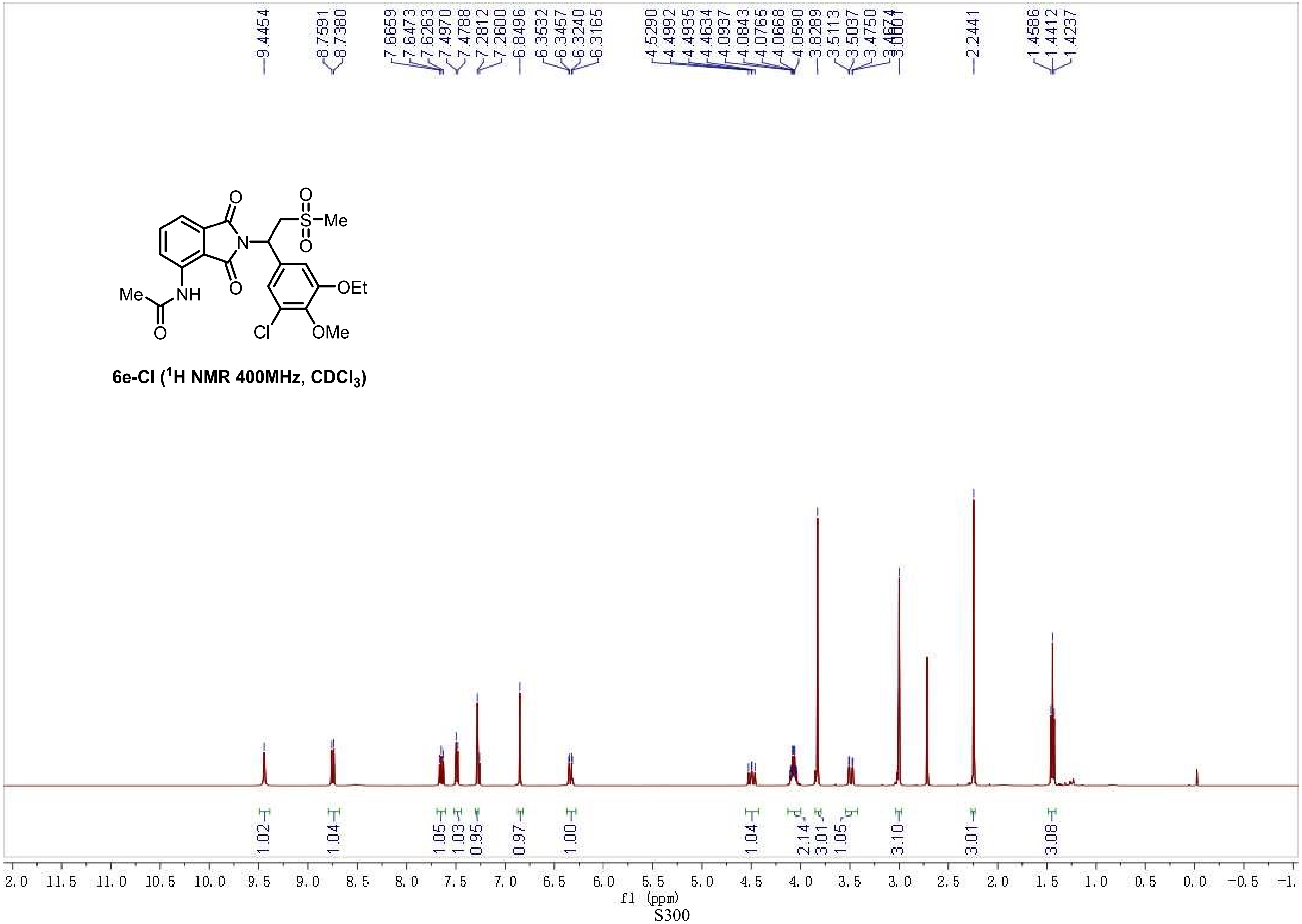

6e-Cl (^{1}H NMR 400MHz, $CDCl_3$)

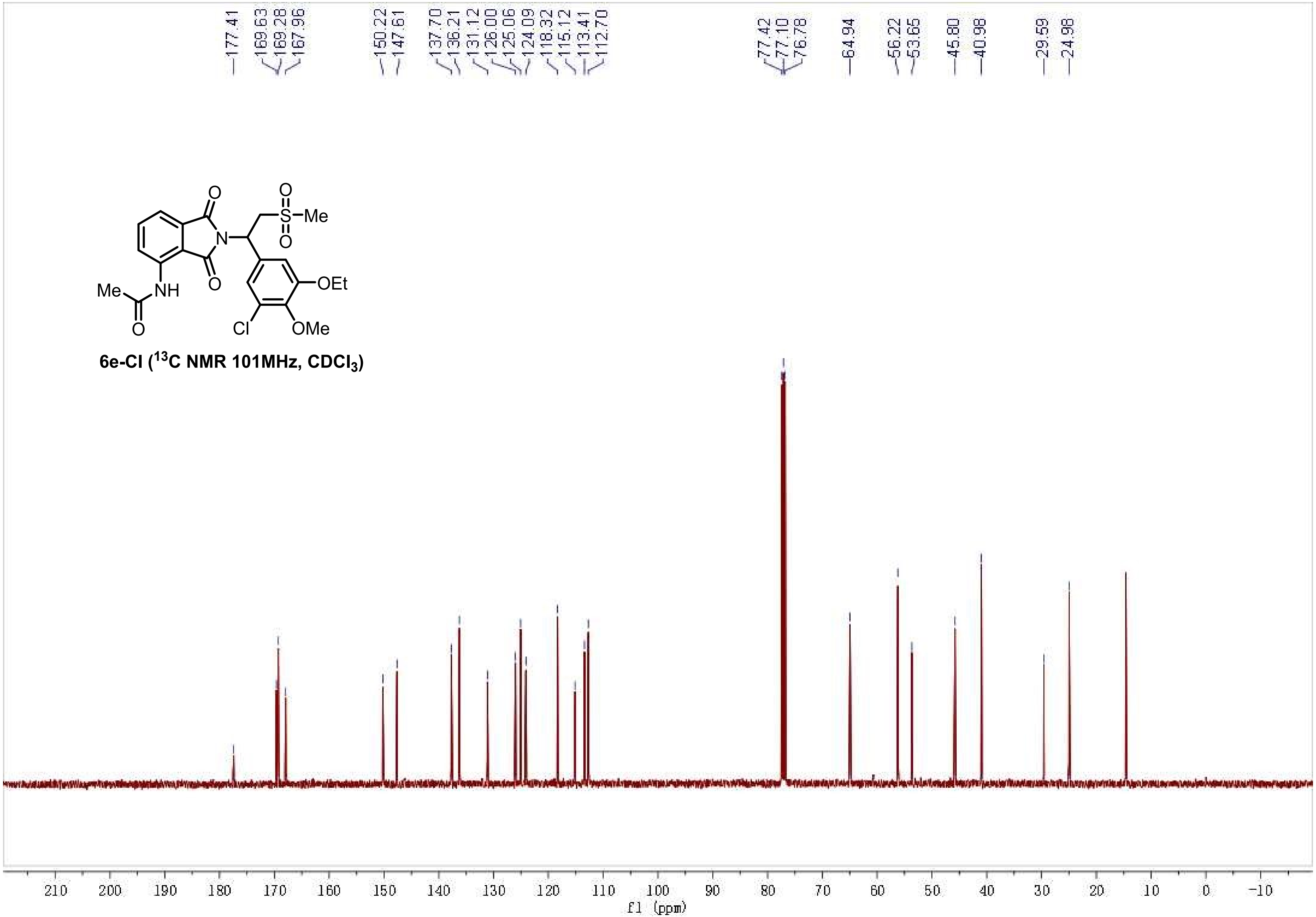

6e-Cl (^{13}C NMR 101MHz, $CDCl_3$)

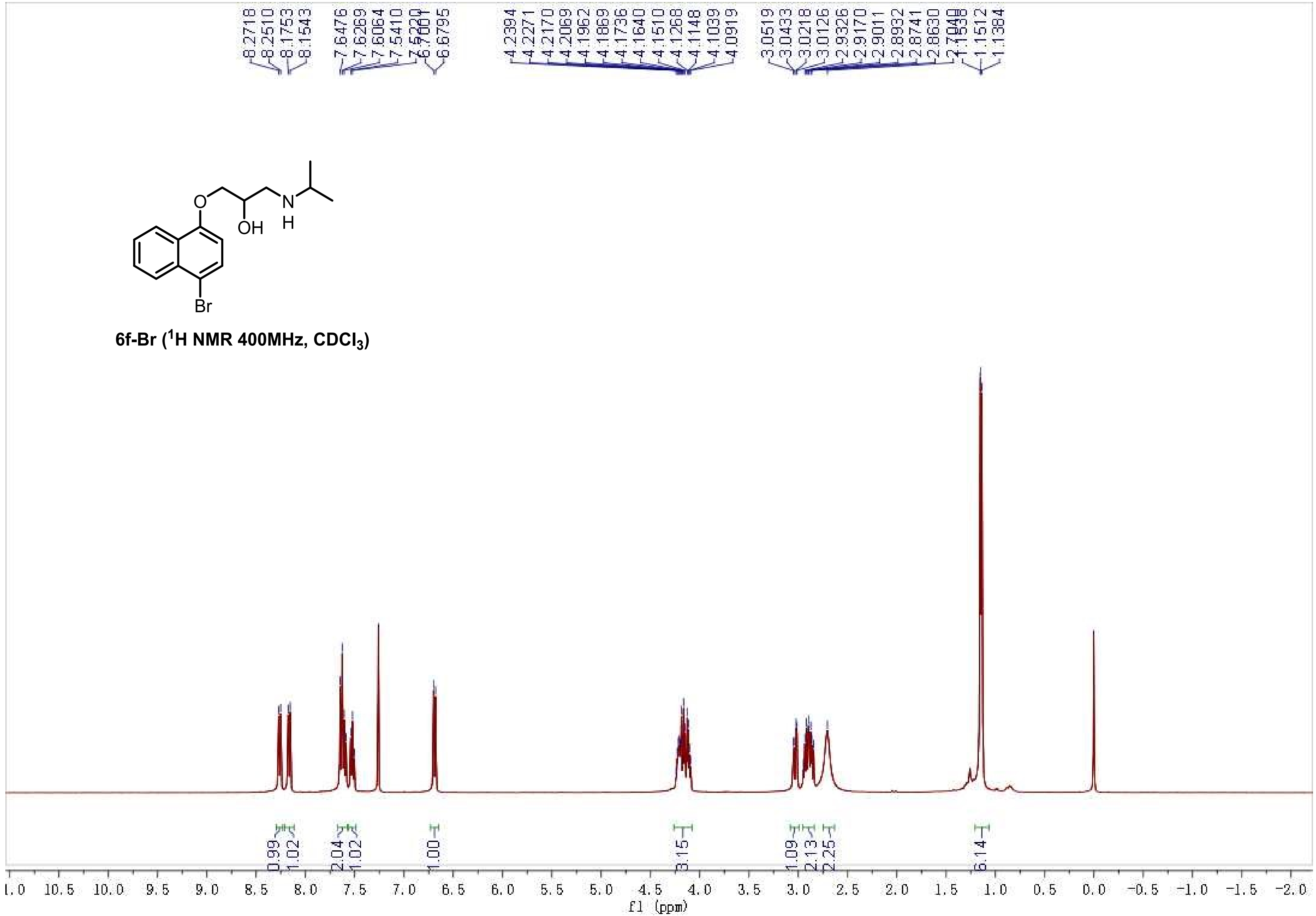

6f-Br (^{1}H NMR 400MHz, $CDCl_3$)

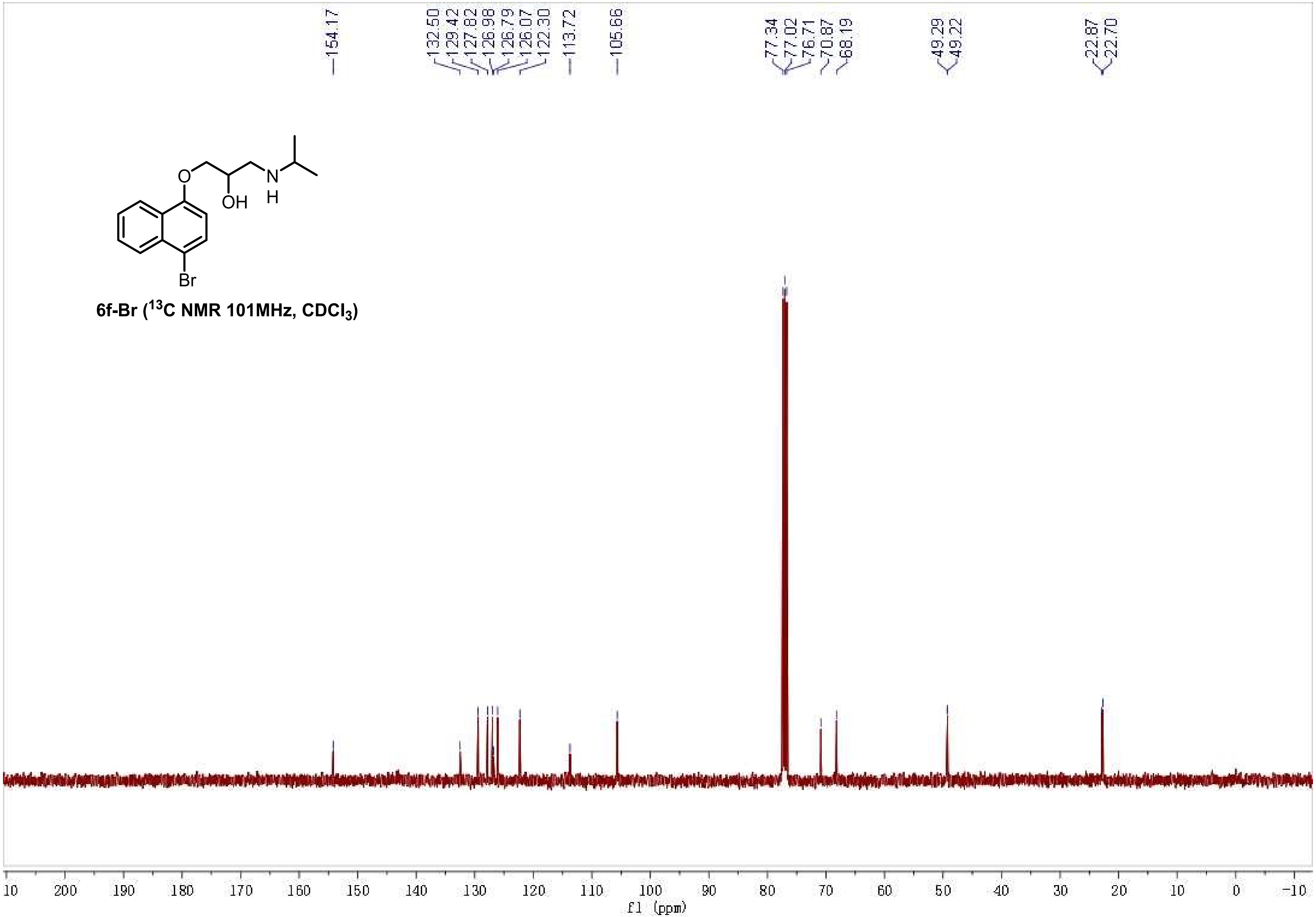

6f-Br (^{13}C NMR 101MHz, $CDCl_3$)

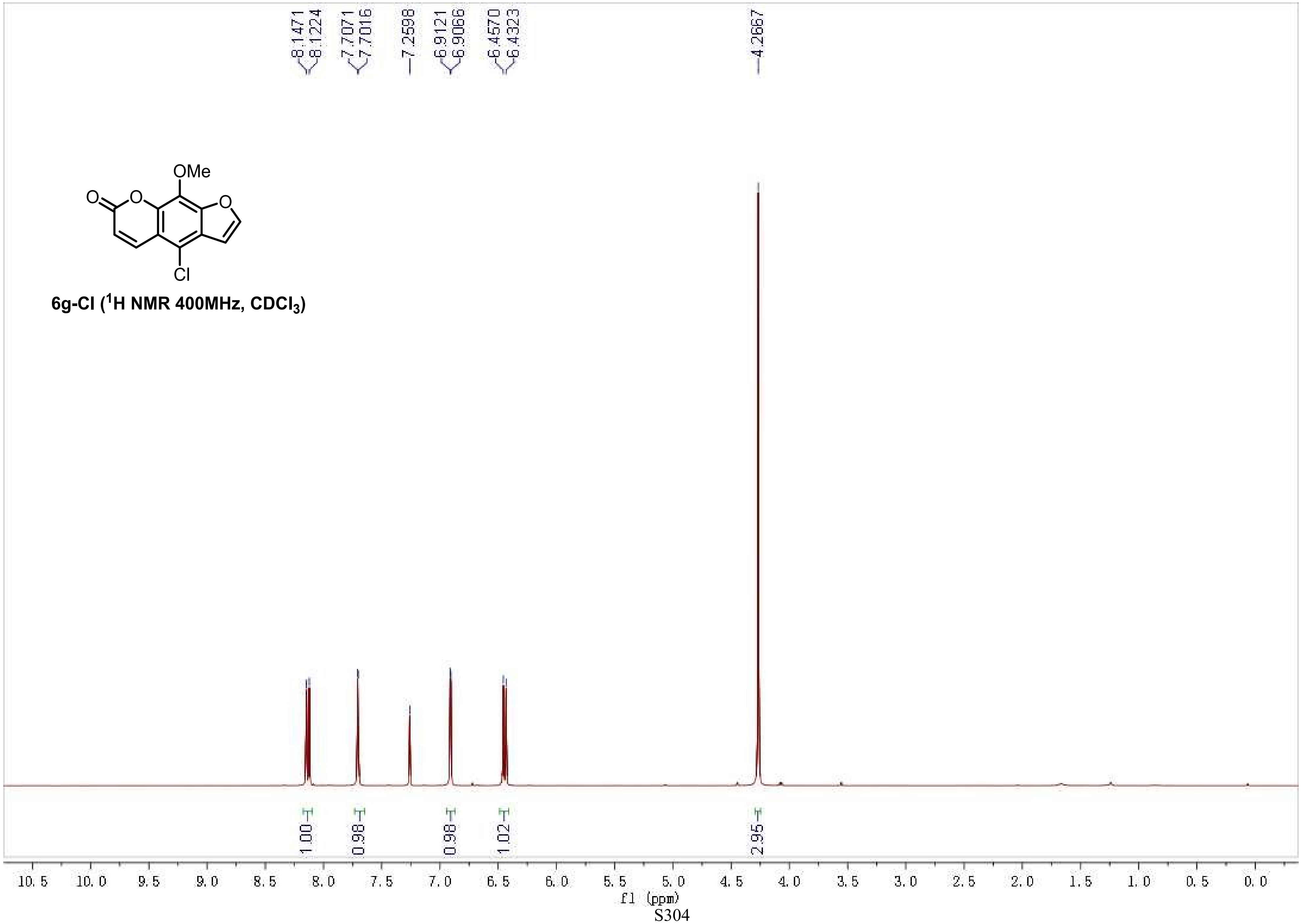

6g-Cl (^{1}H NMR 400MHz, $CDCl_3$)

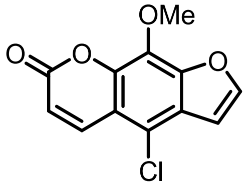

6g-Cl (^{13}C NMR 101MHz, $CDCl_3$)

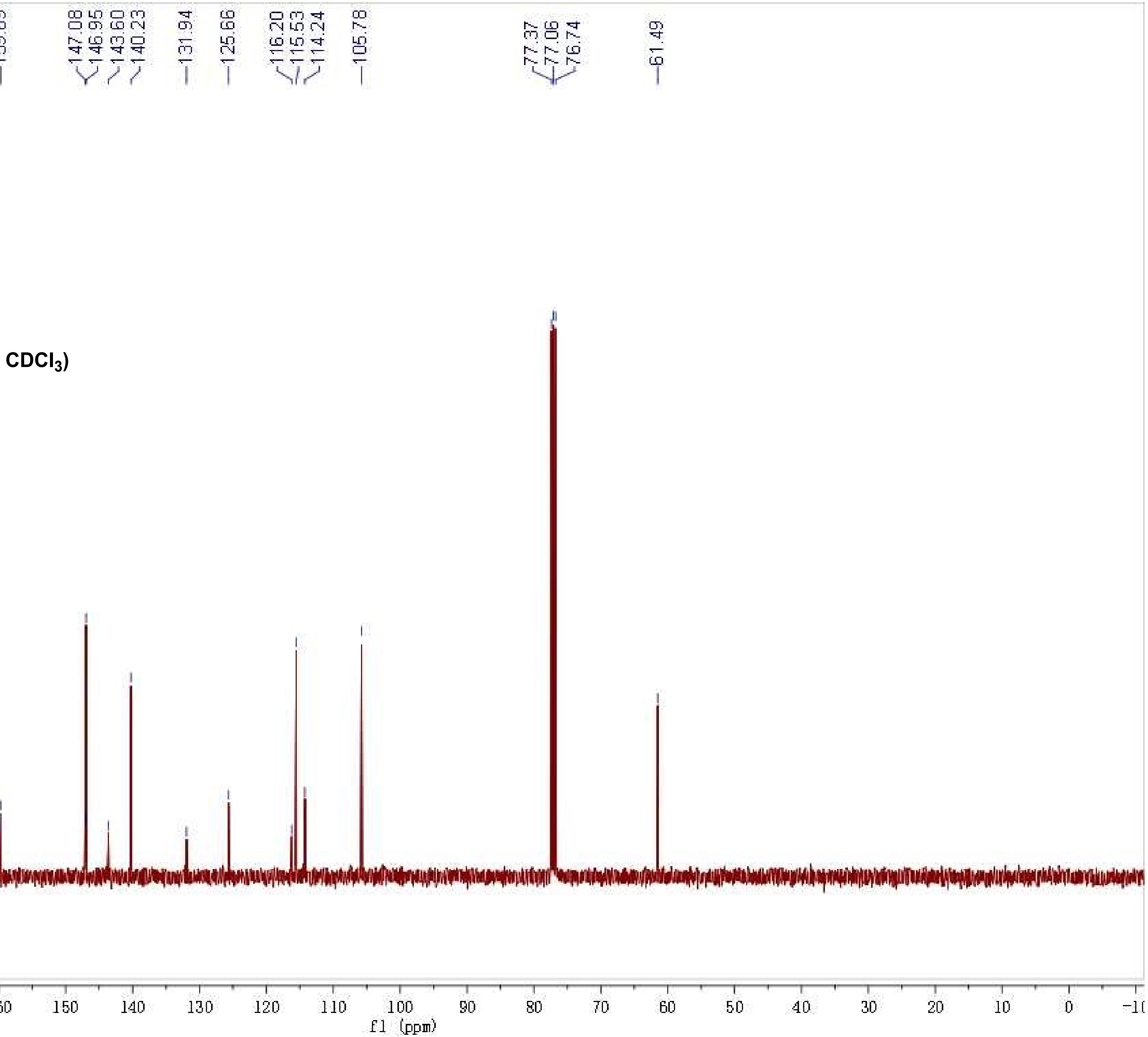

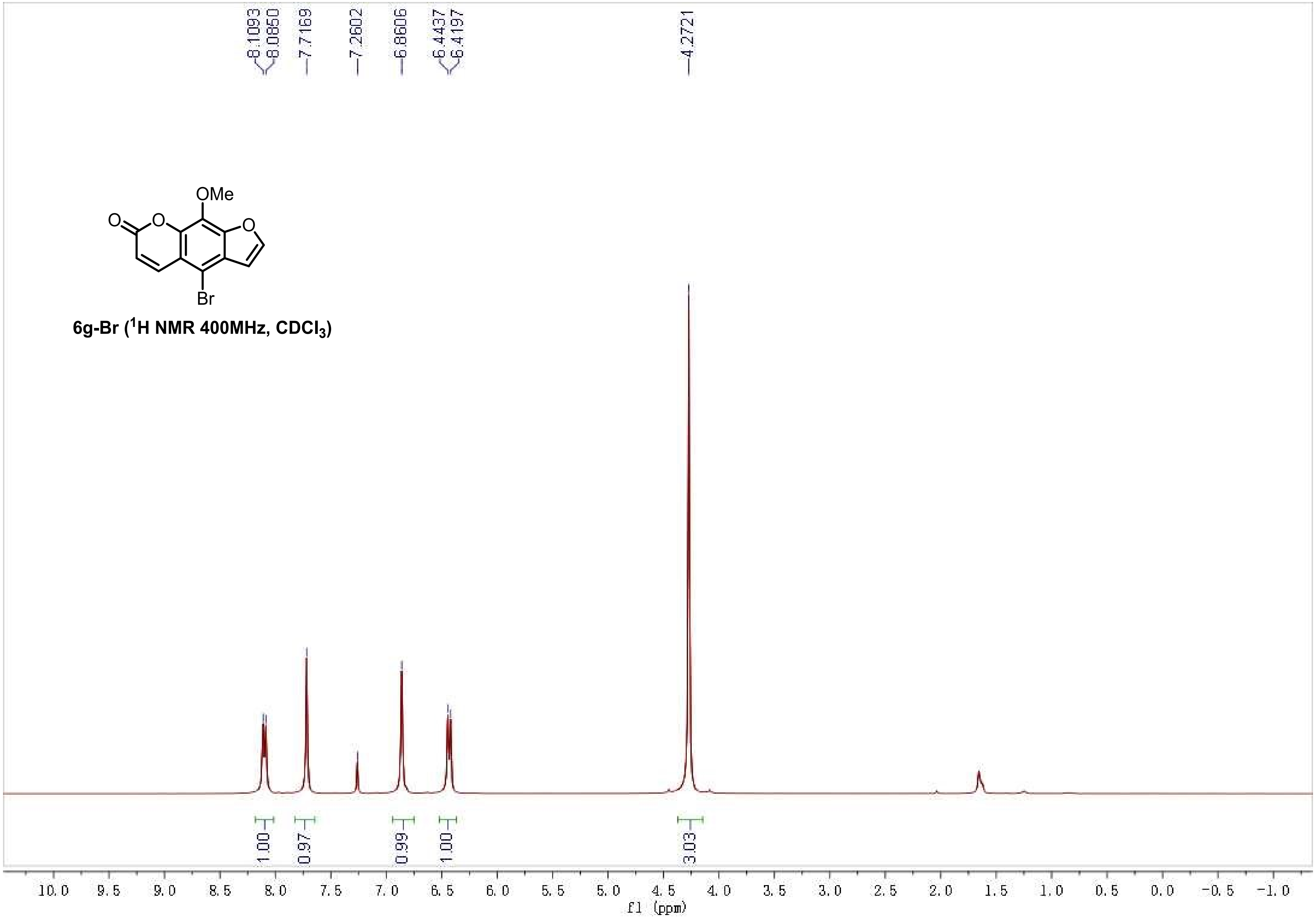

6g-Br (^{1}H NMR 400MHz, $CDCl_3$)

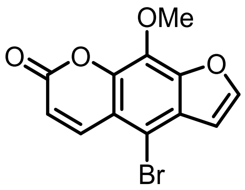

6g-Br (^{13}C NMR 101MHz, $CDCl_3$)

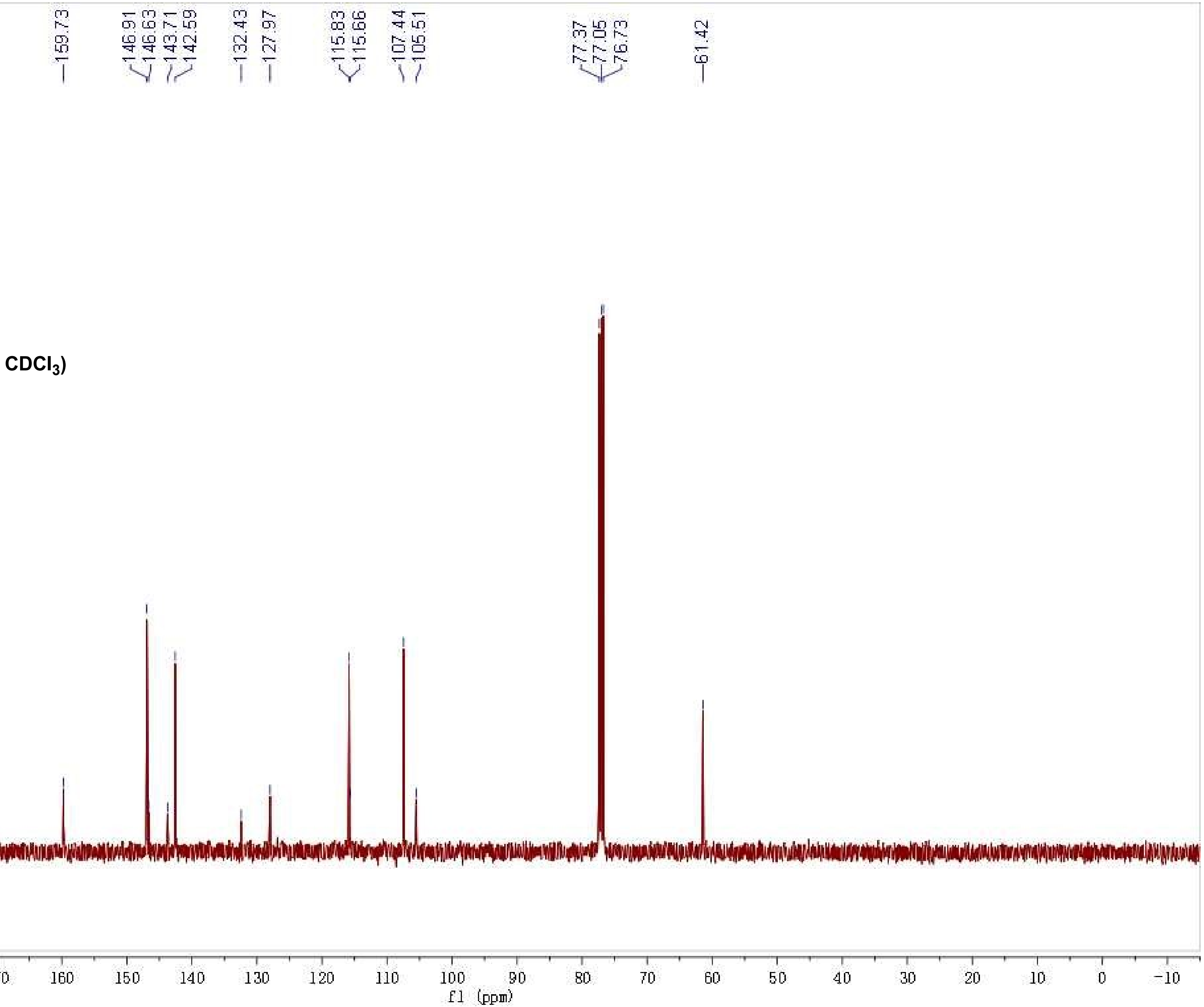

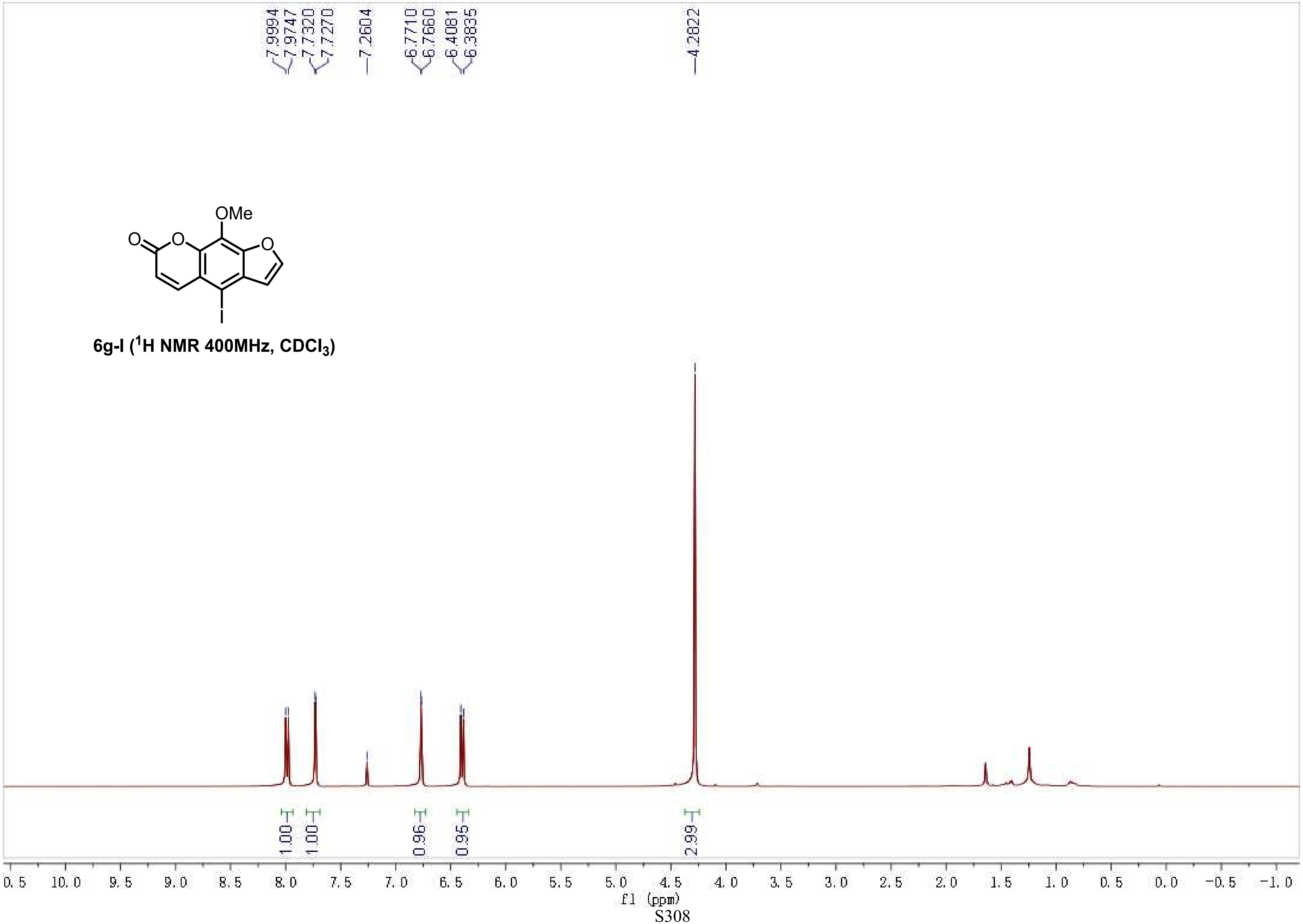
7.9994
7.9747
7.7320
7.7270
7.2604
6.7710
6.7660
6.4081
6.3835
4.2822
OMe
O
O
O
I
6g-I (1H NMR 400MHz, CDCl3)
1.00
1.00
0.96
0.95
2.99
0.5
10.0
9.5
9.0
8.5
8.0
7.5
7.0
6.5
6.0
5.5
5.0
4.5
4.0
3.5
3.0
2.5
2.0
1.5
1.0
0.5
0.0
-0.5
-1.0
f1 (ppm)

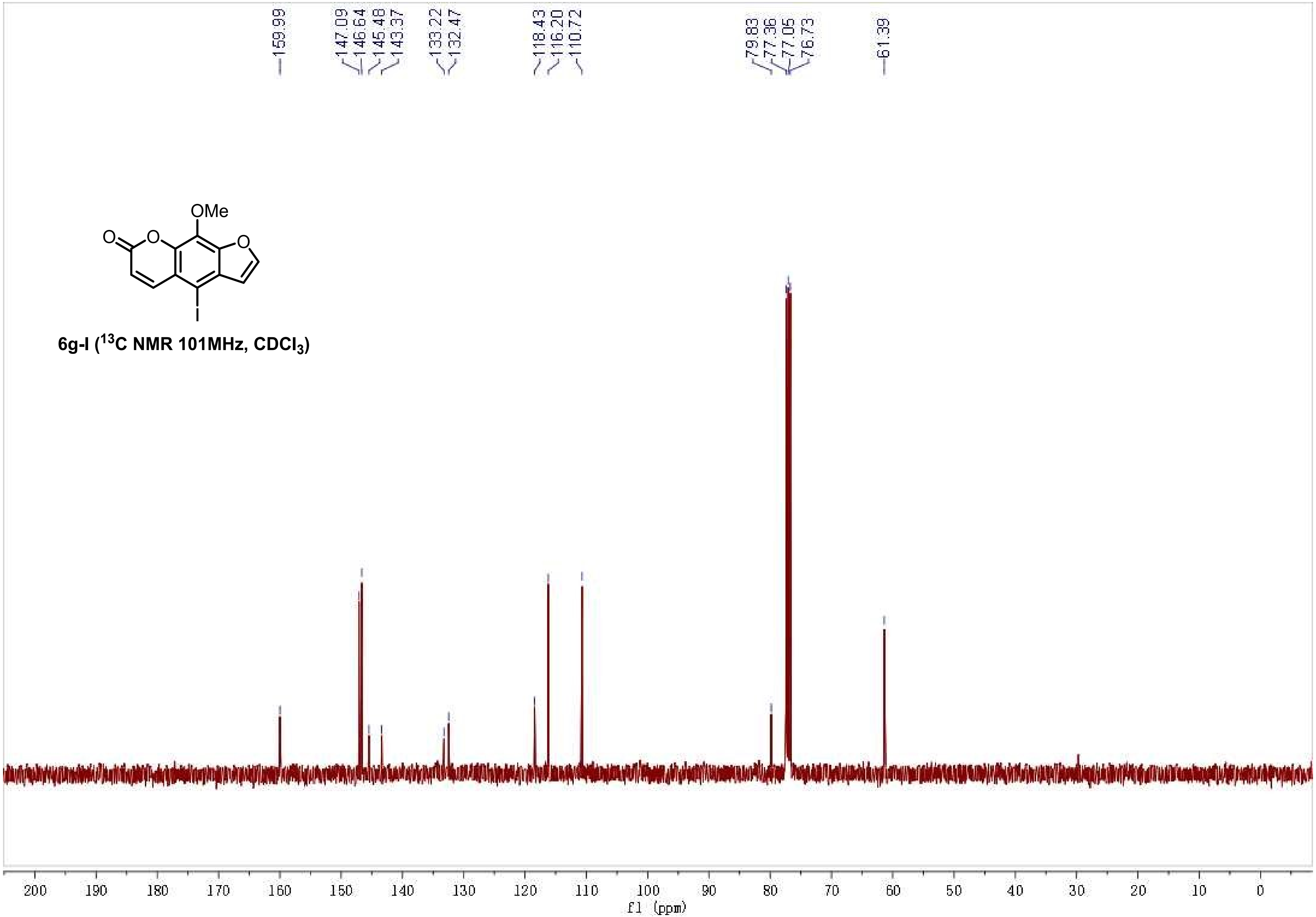
159.99
147.09
146.64
145.48
143.37
133.22
132.47
118.43
116.20
110.72
79.83
77.36
77.05
76.73
61.39
OMe
O
O
O
I
6g-I (^{13}C NMR 101MHz, $CDCl_3$)
200 190 180 170 160 150 140 130 120 110 100 90 80 70 60 50 40 30 20 10 0
f1 (ppm)

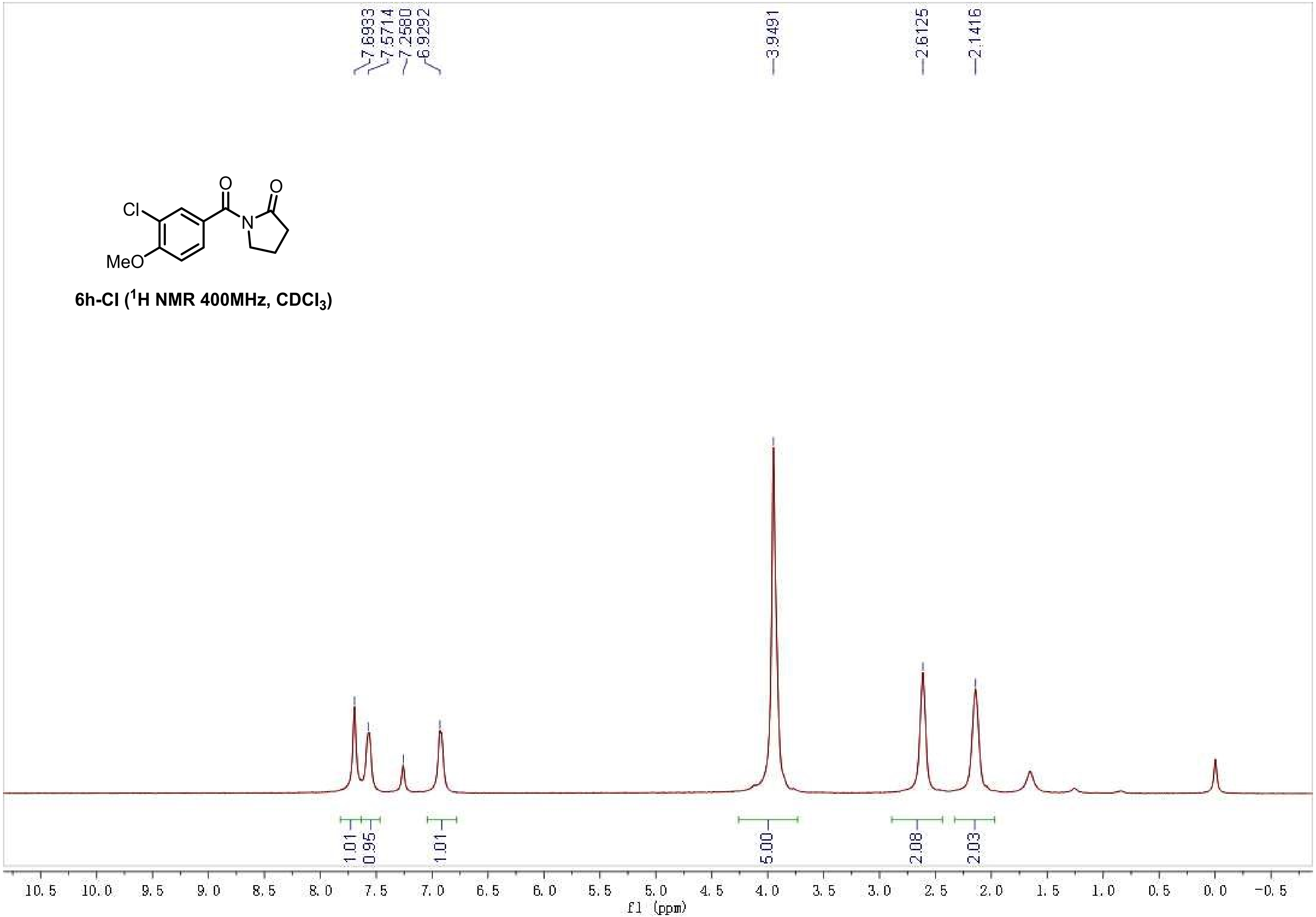

6h-Cl (^{1}H NMR 400MHz, $CDCl_3$)

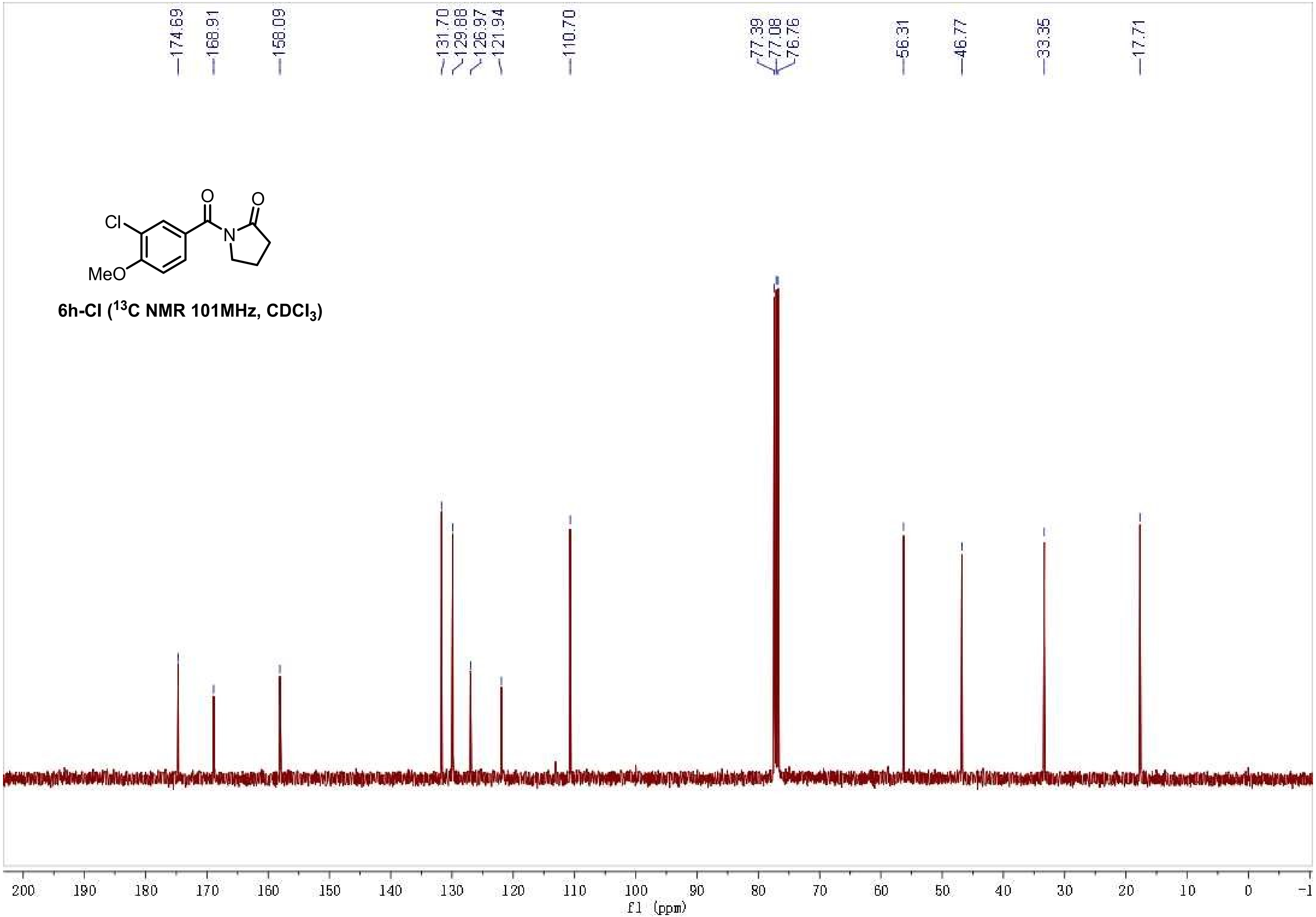

174.69
168.91
158.09
131.70
129.88
126.97
121.94
110.70
77.39
77.08
76.76
56.31
46.77
33.35
17.71
Cl
MeO
O
O
N
6h-Cl (13C NMR 101MHz, CDCl3)
200
190
180
170
160
150
140
130
120
110
100
90
80
70
60
50
40
30
20
10
0
-1
f1 (ppm)

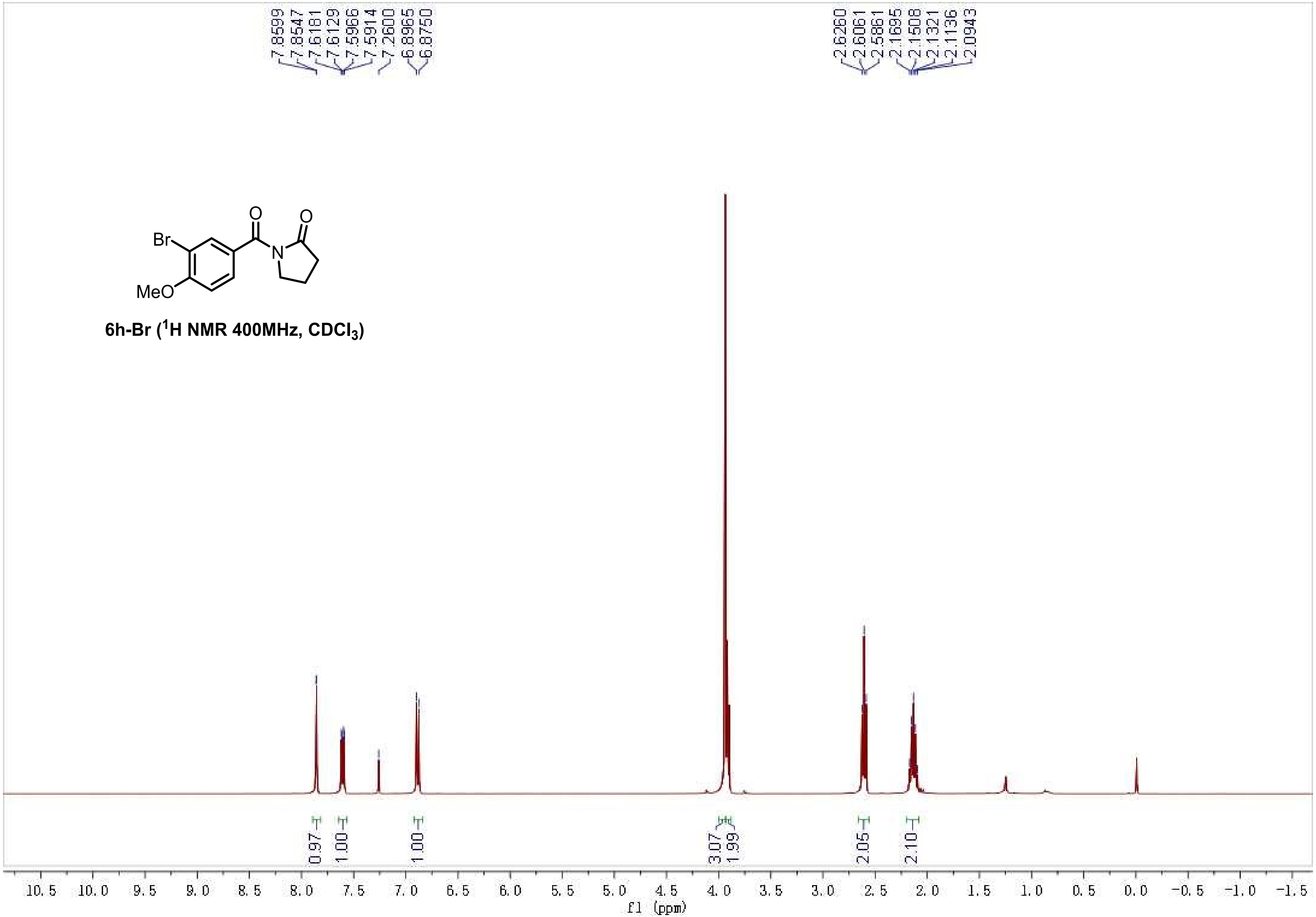

6h-Br (^{1}H NMR 400MHz, $CDCl_3$)

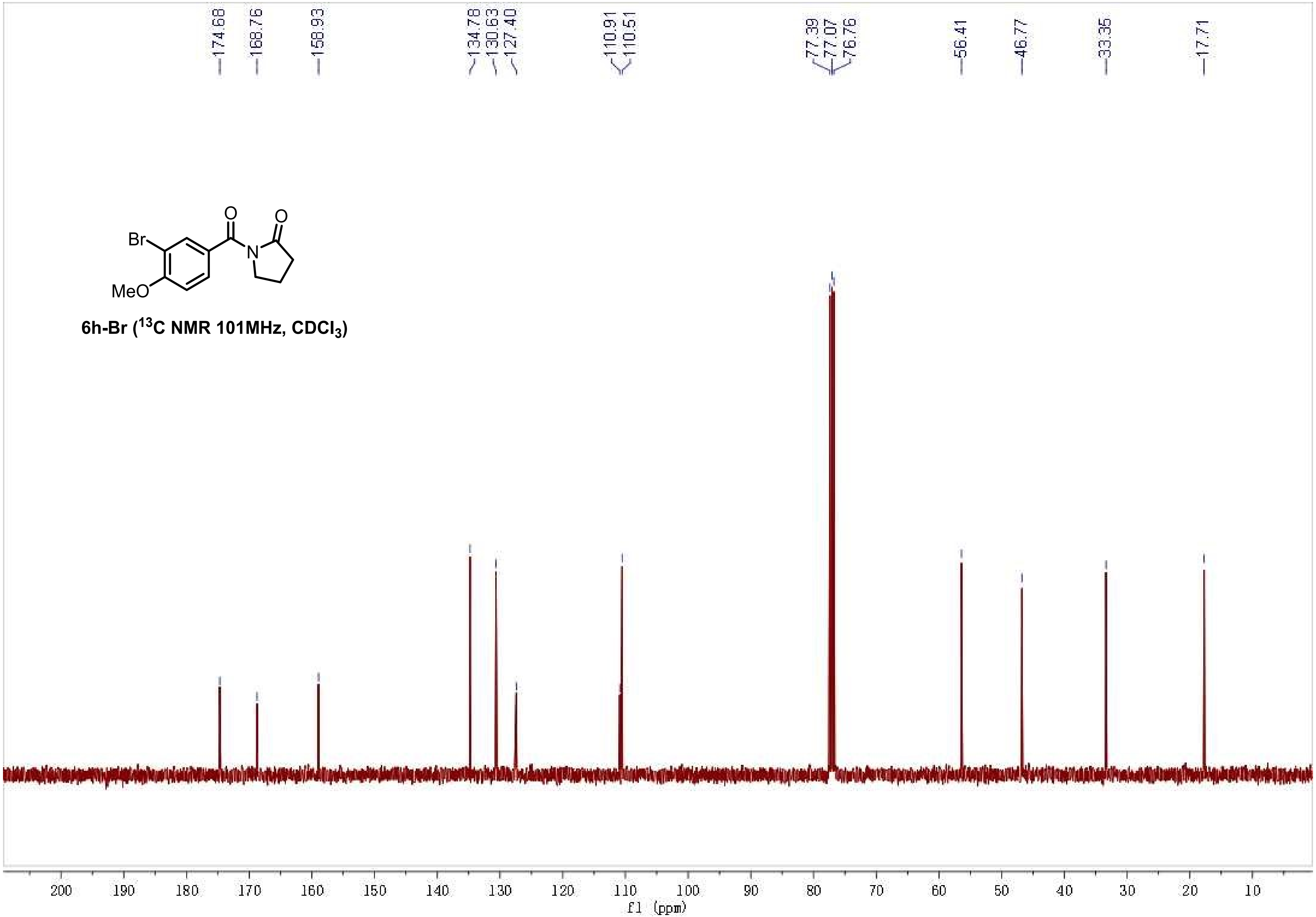

6h-Br (^{13}C NMR 101MHz, $CDCl_3$)

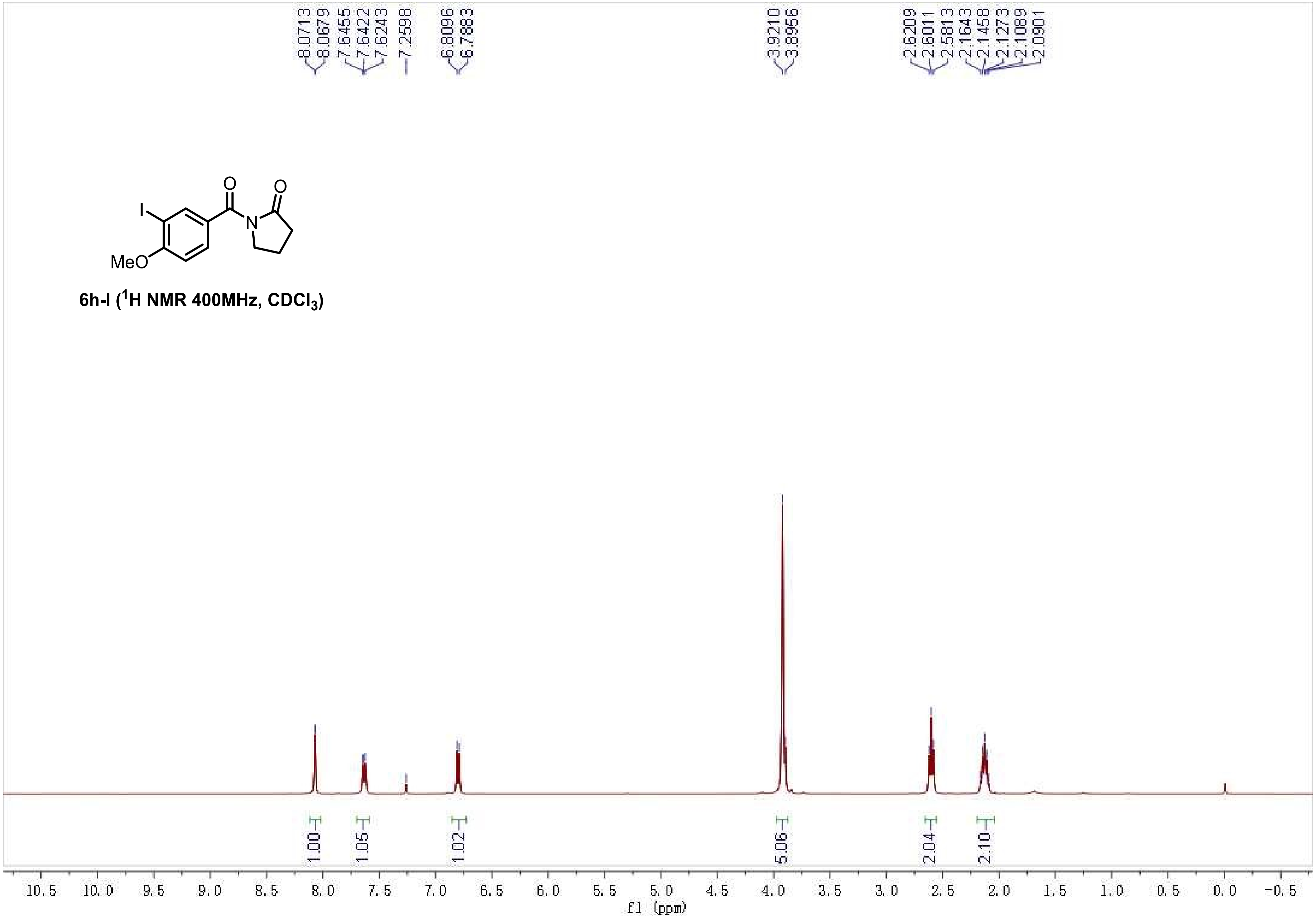

6h-I (^{1}H NMR 400MHz, $CDCl_3$)

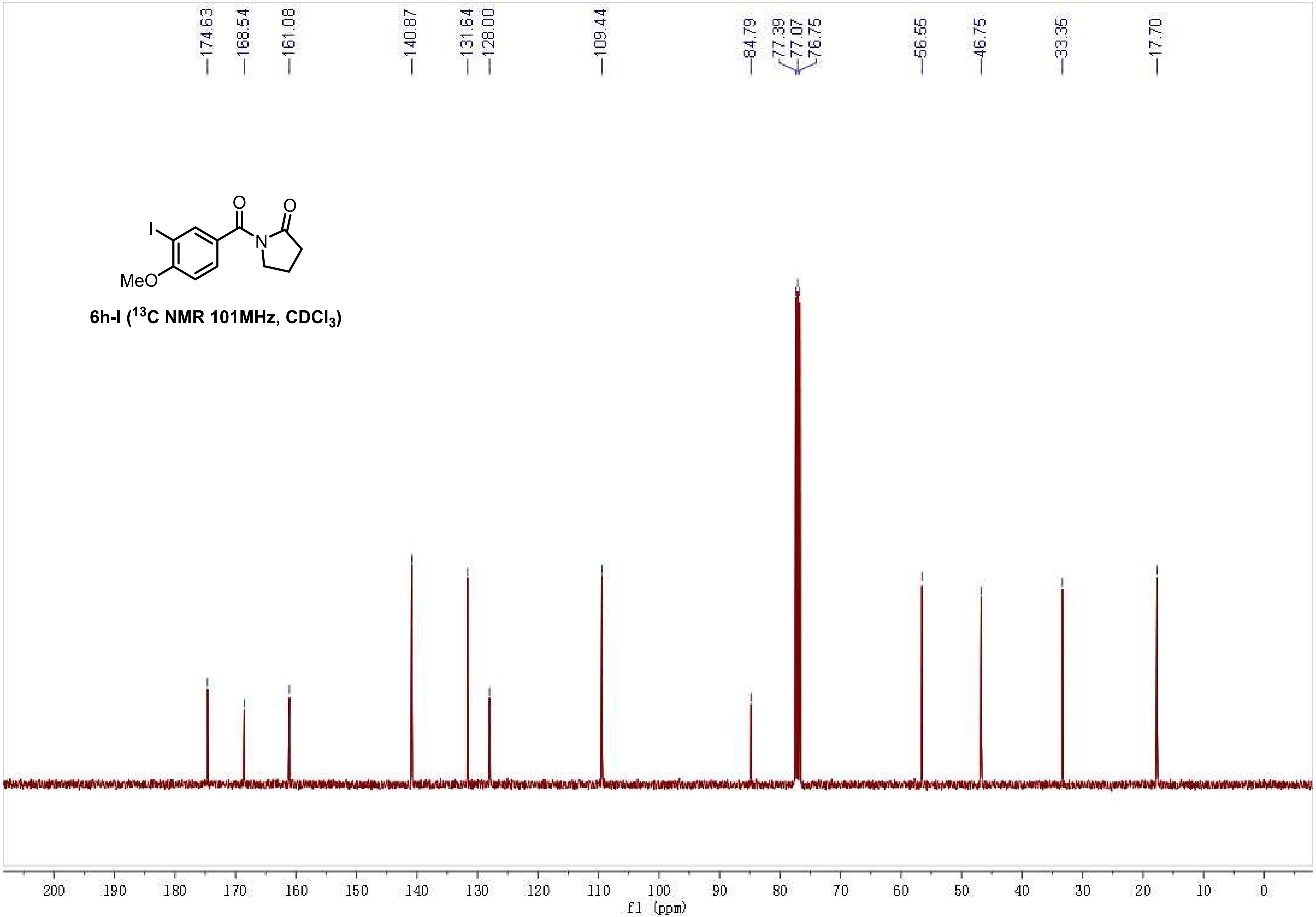

6h-I (^{13}C NMR 101MHz, $CDCl_3$)

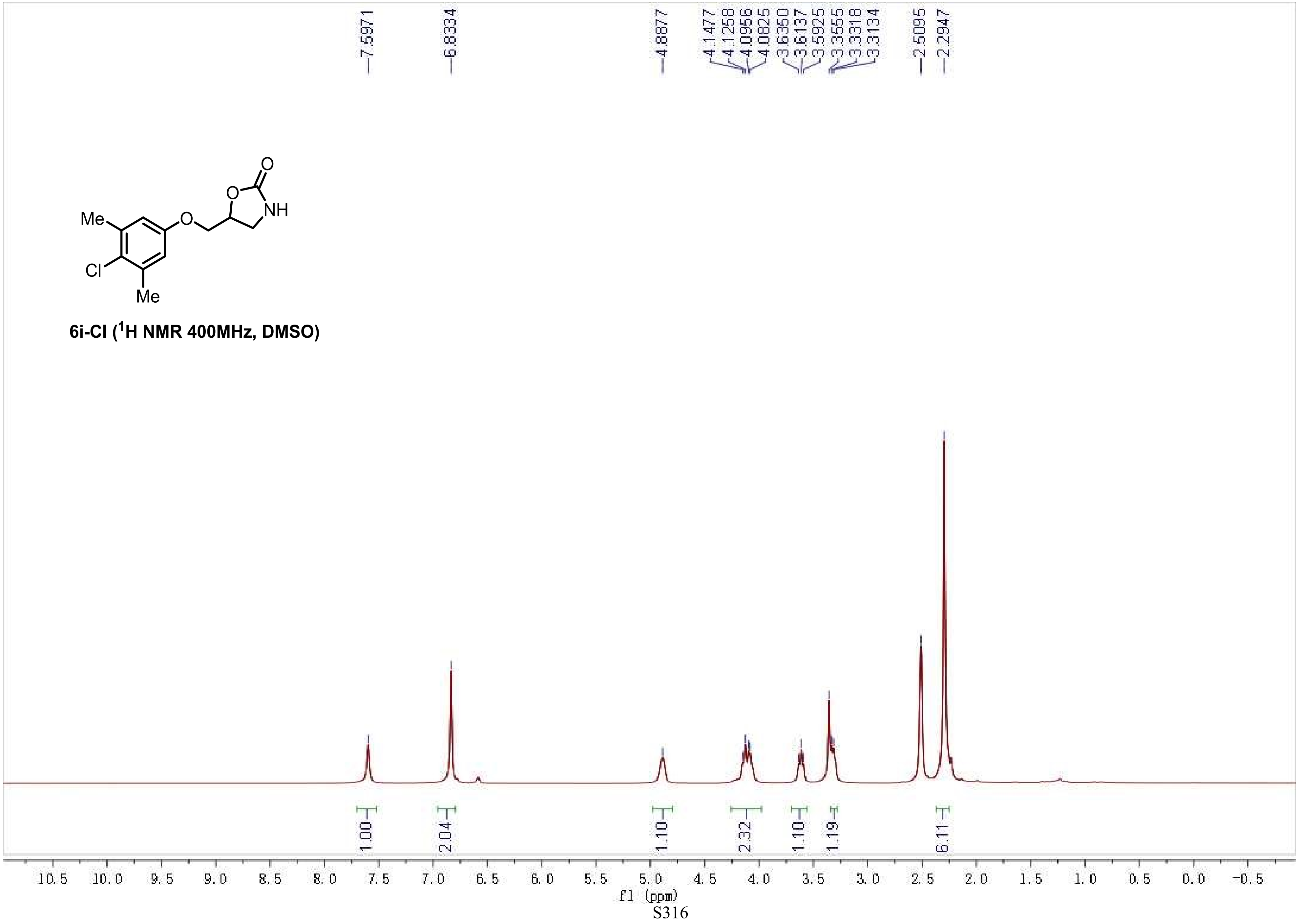

7.5971
6.8334
4.8877
4.1477
4.1258
4.0956
4.0825
3.6350
3.6137
3.5925
3.3555
3.3318
3.3134
2.5095
2.2947
Me
Cl
Me
O
O
NH
O
6i-Cl (1H NMR 400MHz, DMSO)
1.00
2.04
1.10
2.32
1.10
1.19
6.11
10.5 10.0 9.5 9.0 8.5 8.0 7.5 7.0 6.5 6.0 5.5 5.0 4.5 4.0 3.5 3.0 2.5 2.0 1.5 1.0 0.5 0.0 -0.5
f1 (ppm)

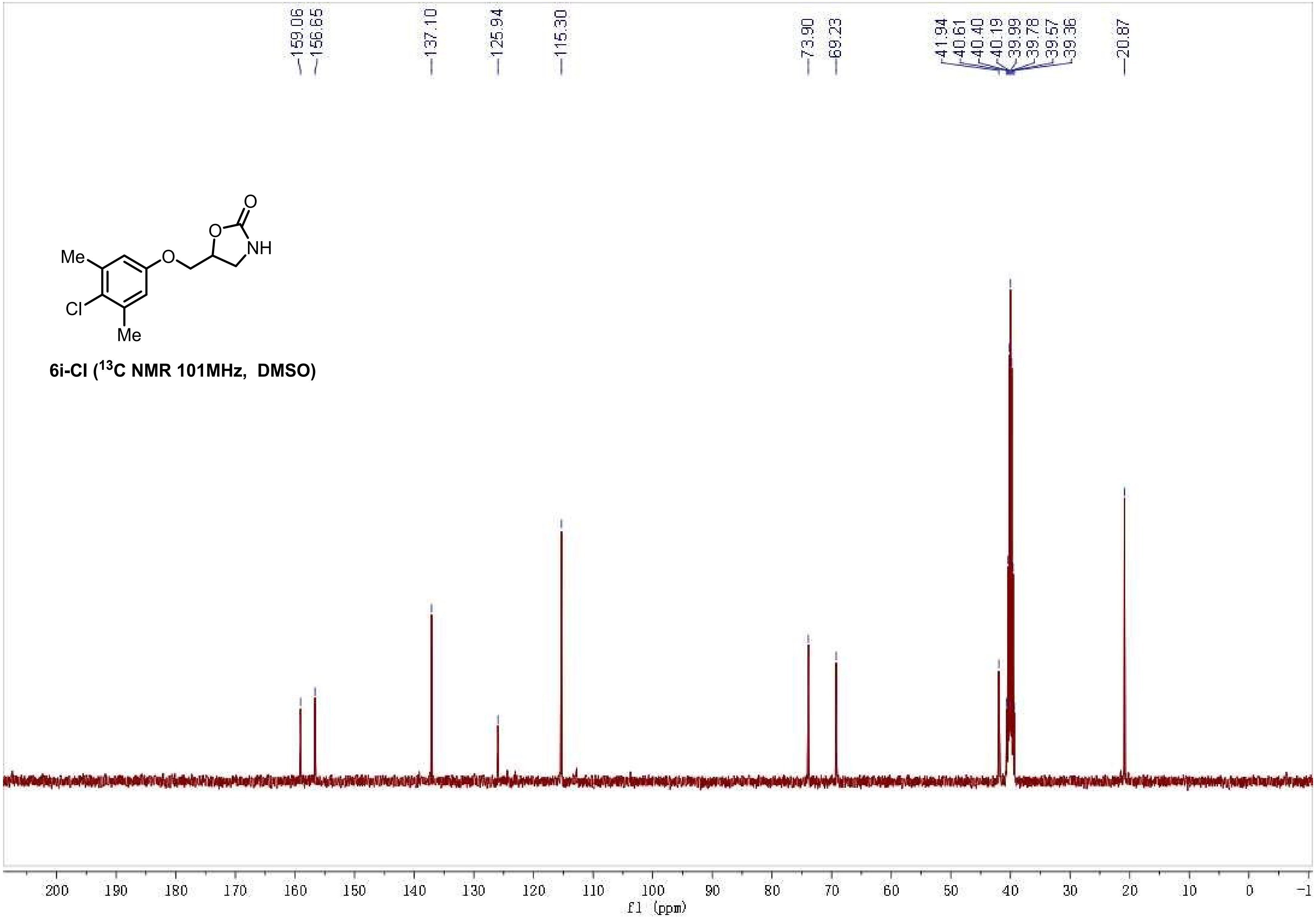

6i-Cl (^{13}C NMR 101MHz, DMSO)

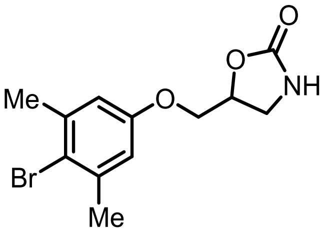

6i-Br (¹H NMR 400MHz, DMSO)

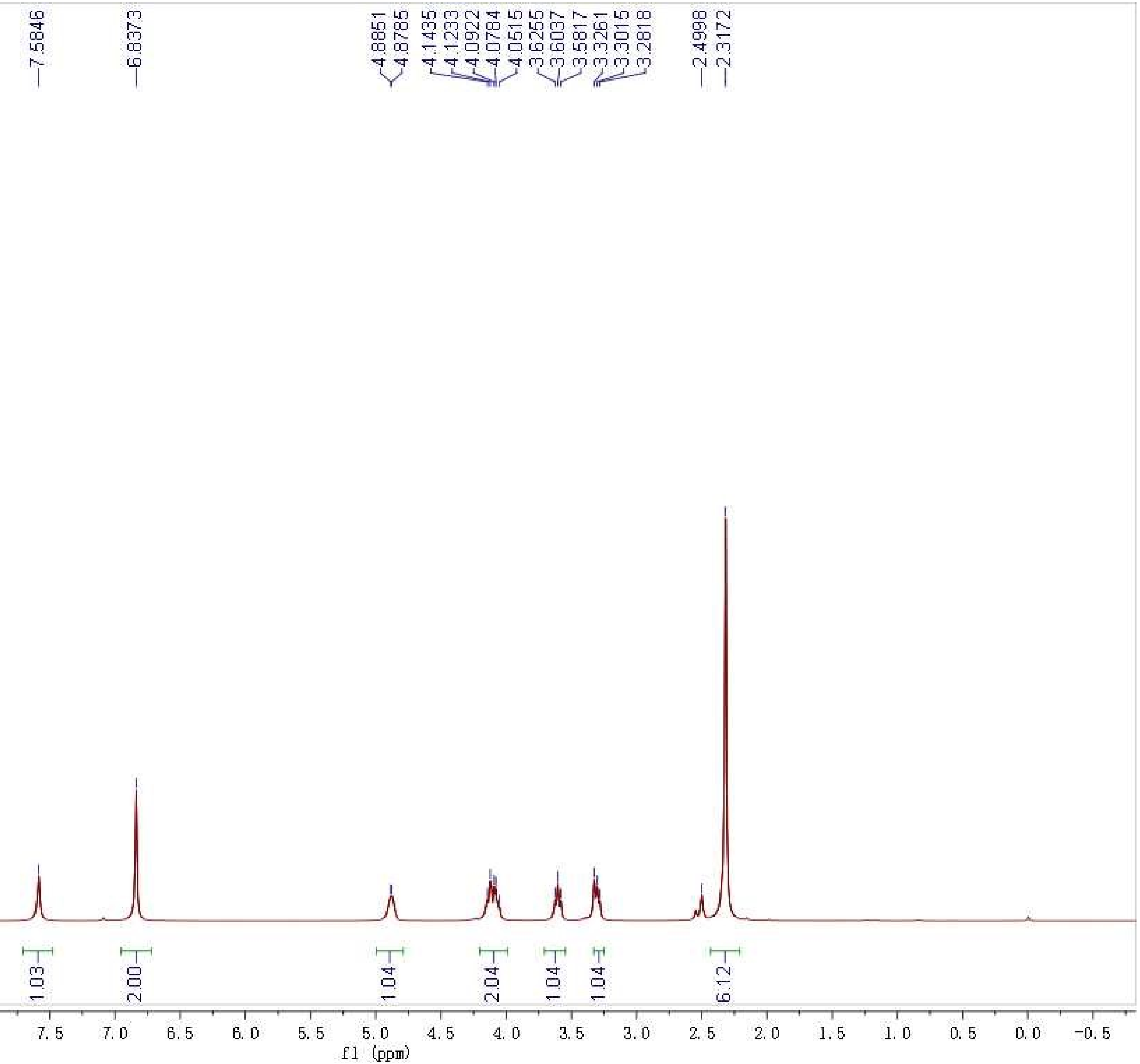

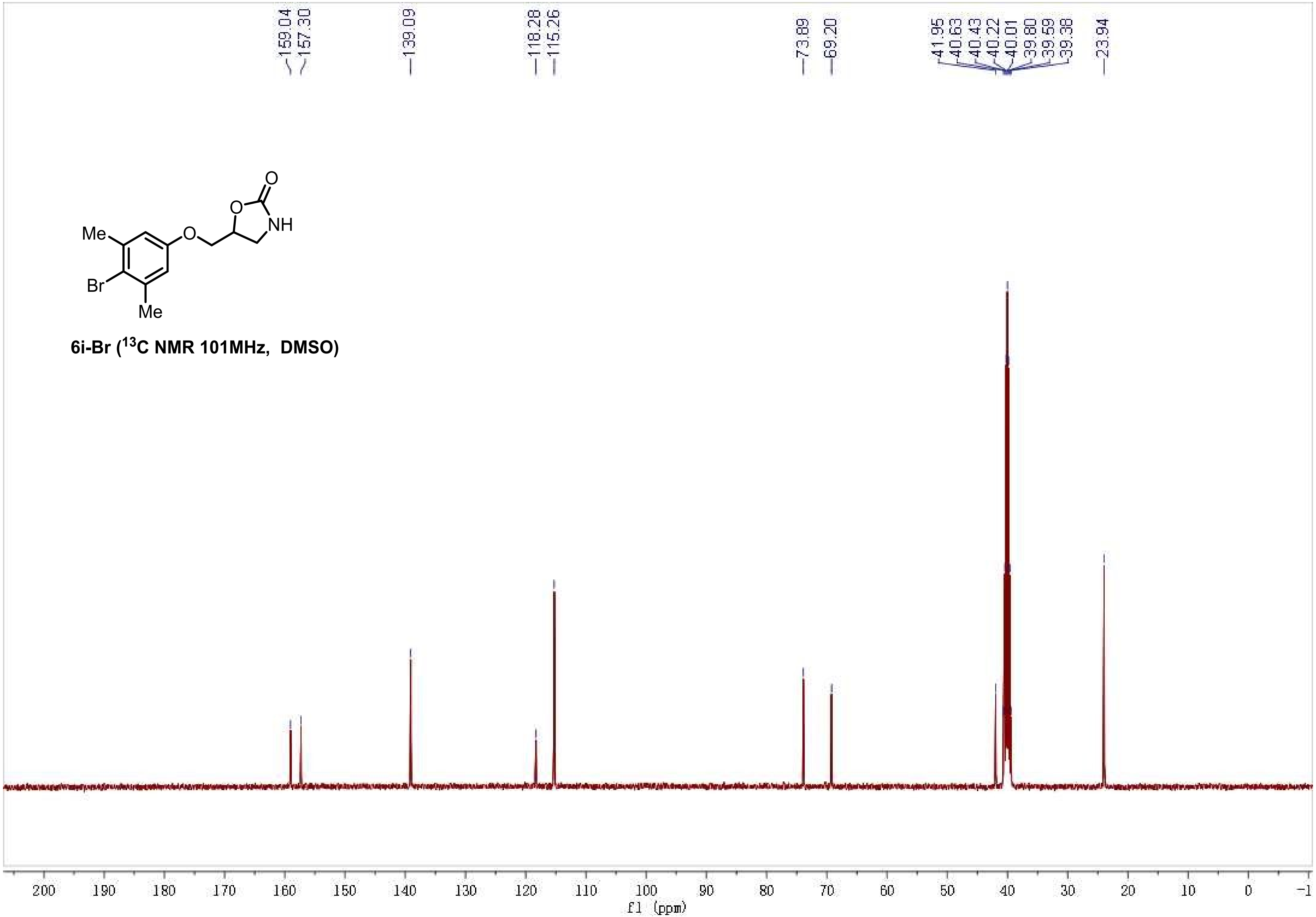

6i-Br (^{13}C NMR 101MHz, DMSO)

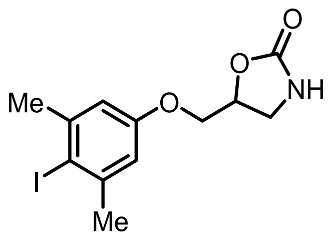

6i-I (^{1}H NMR 400MHz, DMSO)

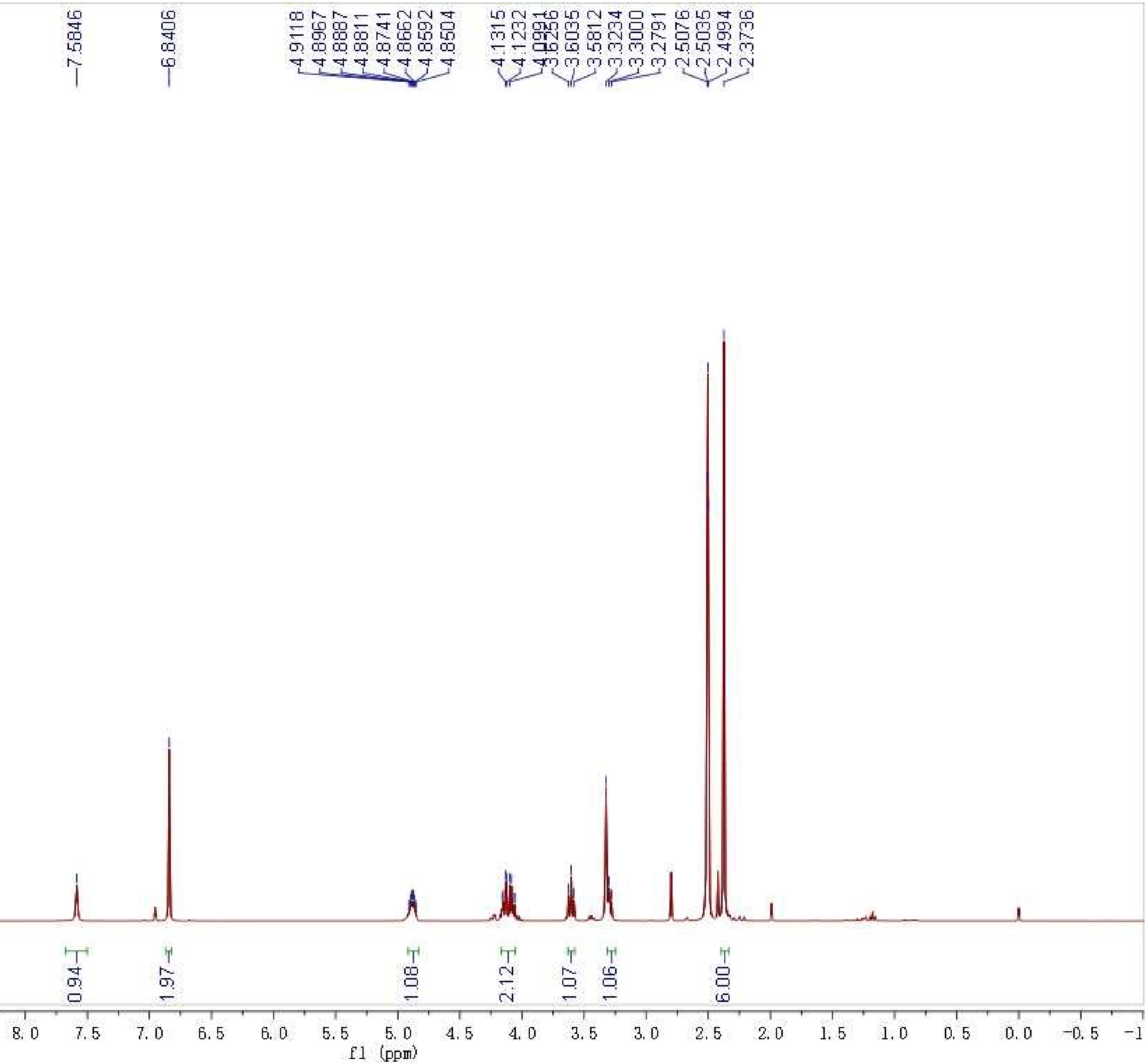

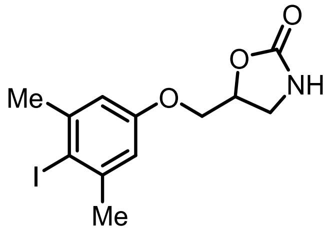

6i-I (^{13}C NMR 101MHz, DMSO)

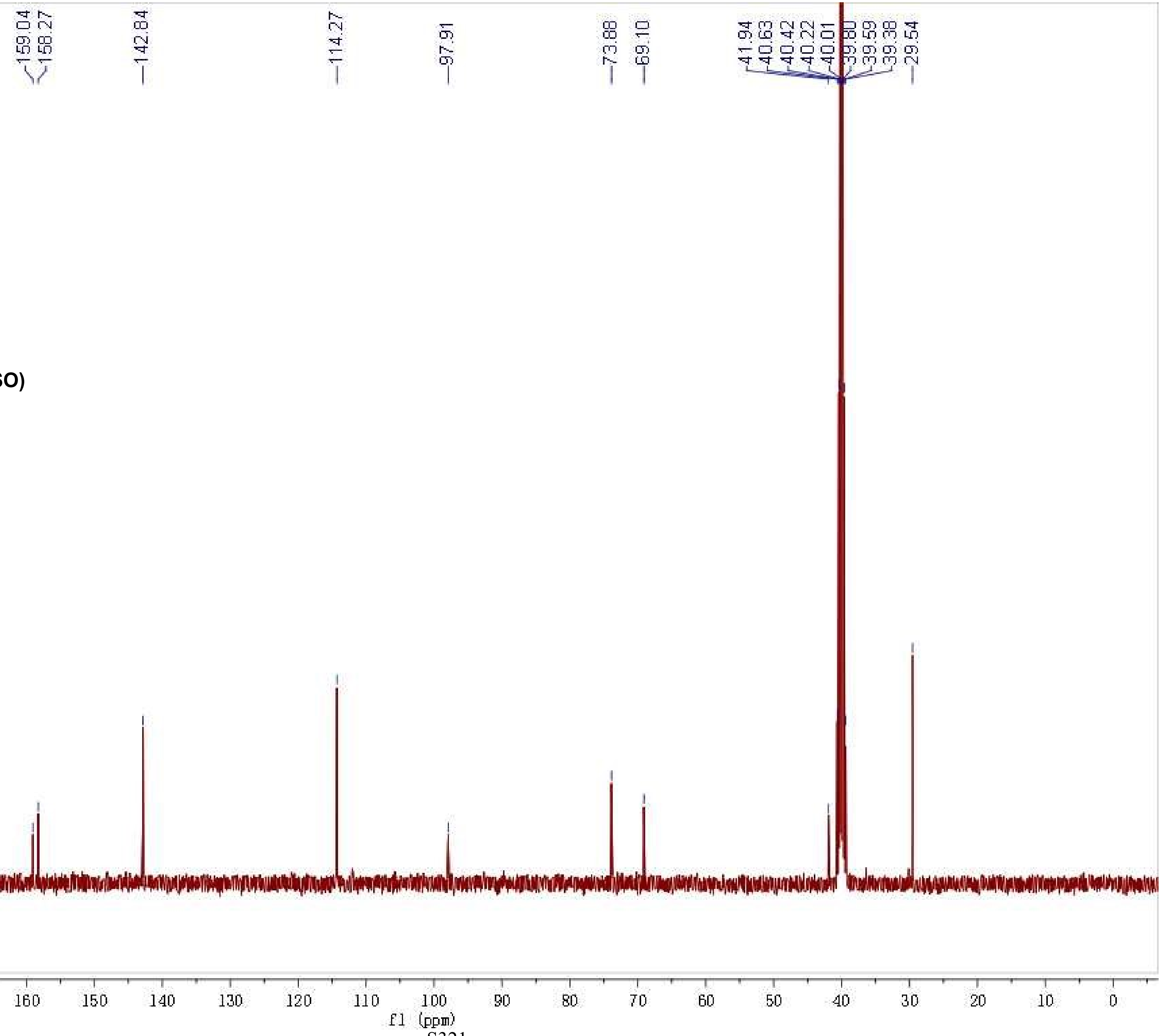

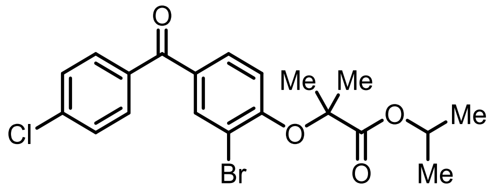

6j-Br (^{1}H NMR 400MHz, $CDCl_3$)

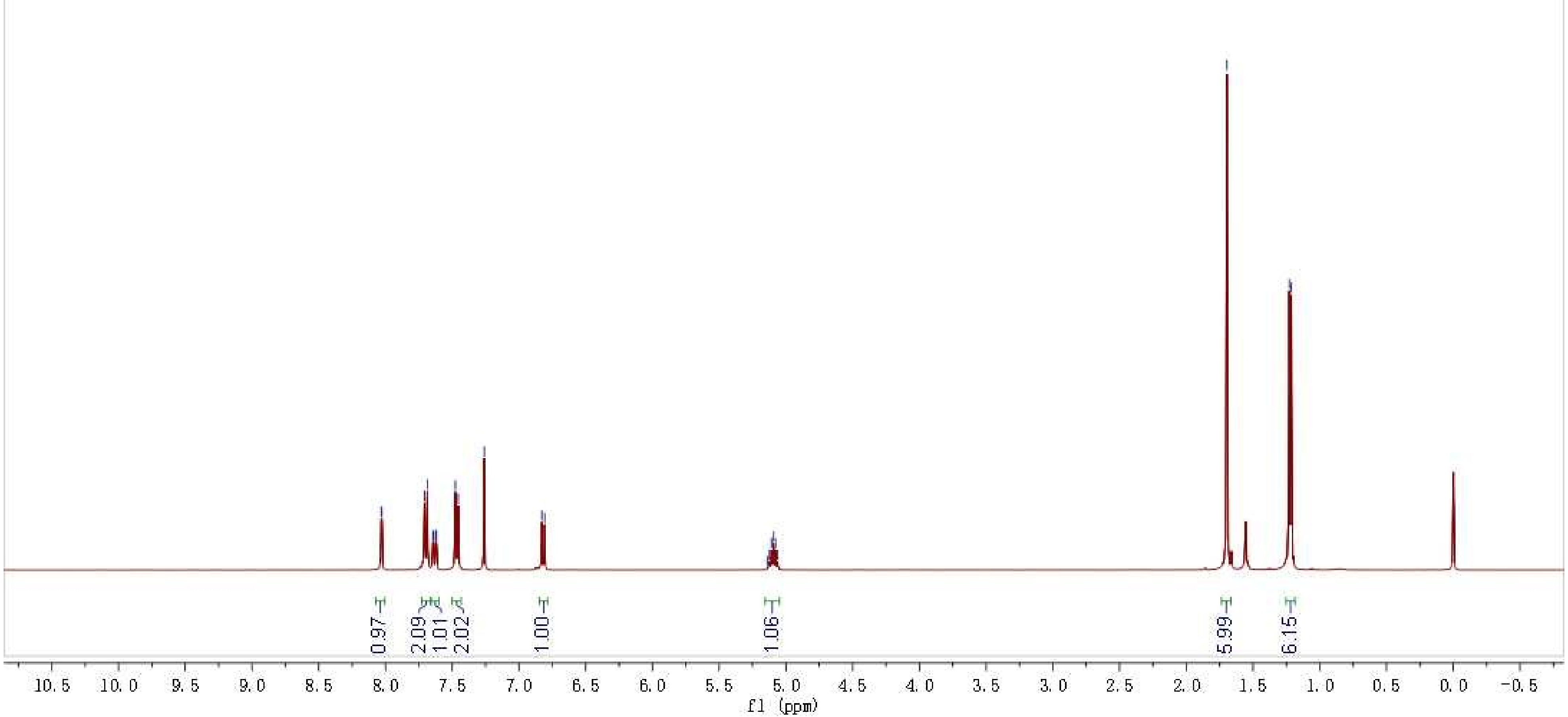

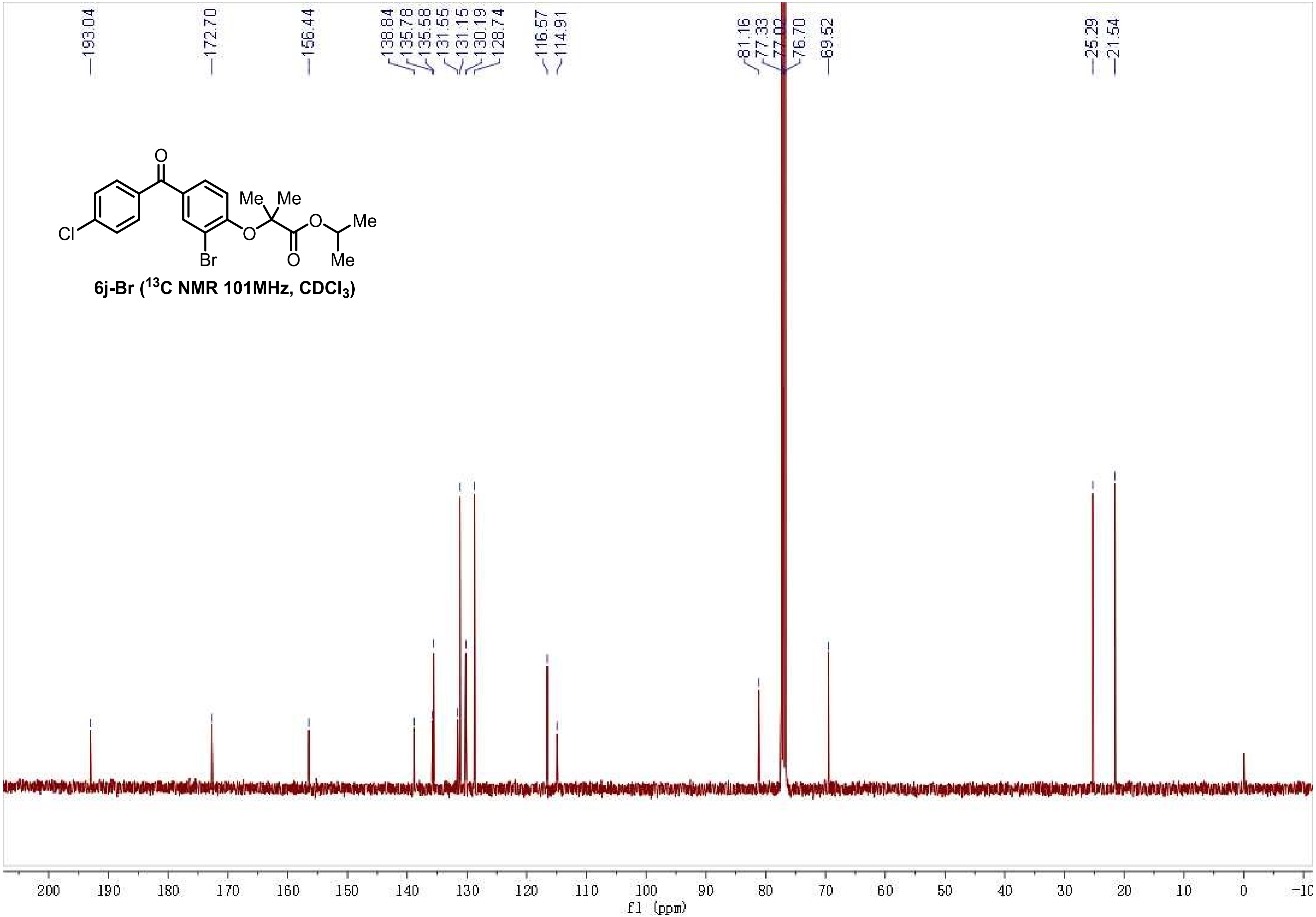

193.04
172.70
156.44
138.84
135.78
135.58
131.55
131.15
130.19
128.74
116.57
114.91
81.16
77.33
77.02
76.70
69.52
25.29
21.54
6j-Br (^{13}C NMR 101MHz, CDCl$_3$)
200 190 180 170 160 150 140 130 120 110 100 90 80 70 60 50 40 30 20 10 0 -10
f1 (ppm)

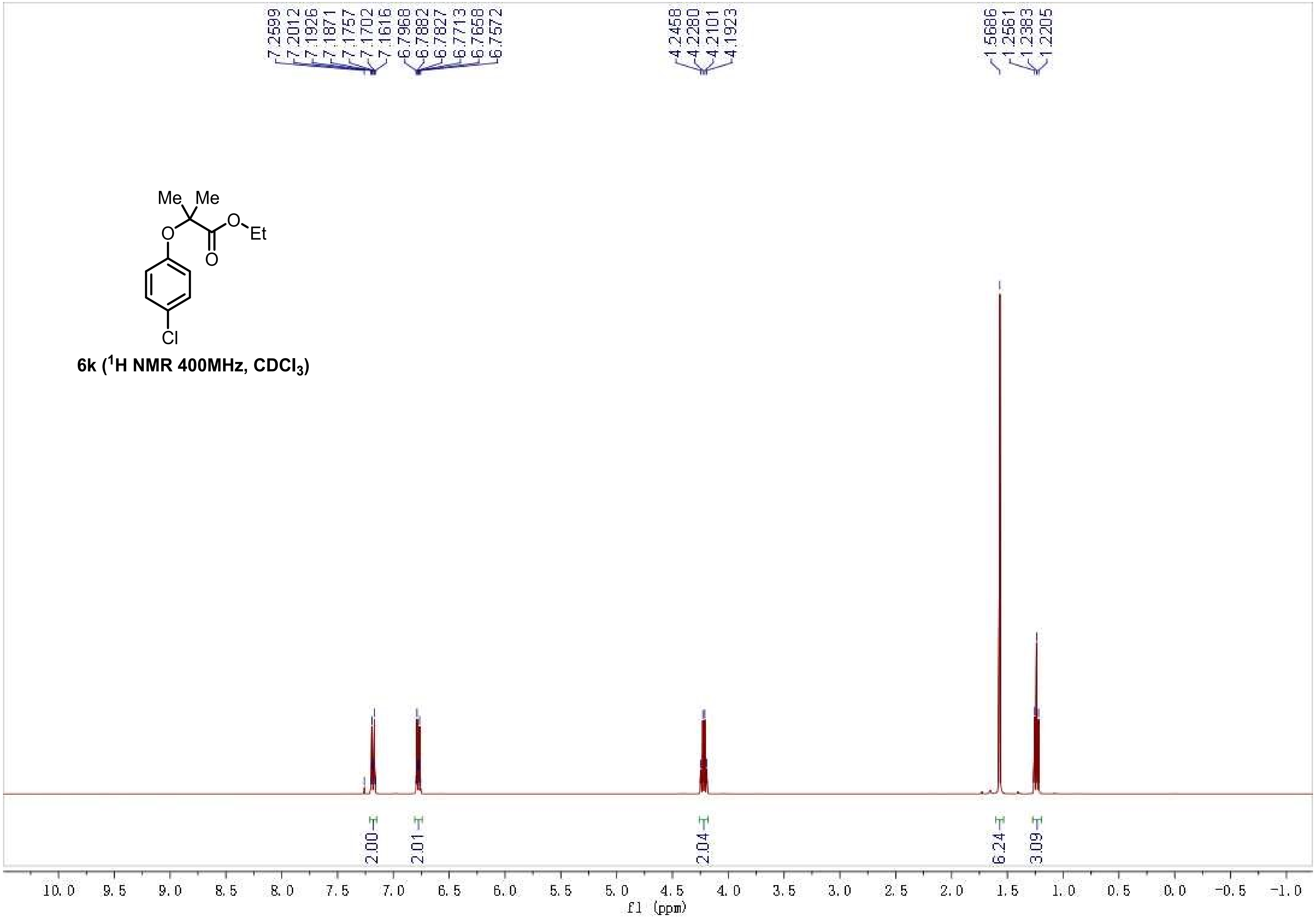

6k (^{1}H NMR 400MHz, $CDCl_3$)

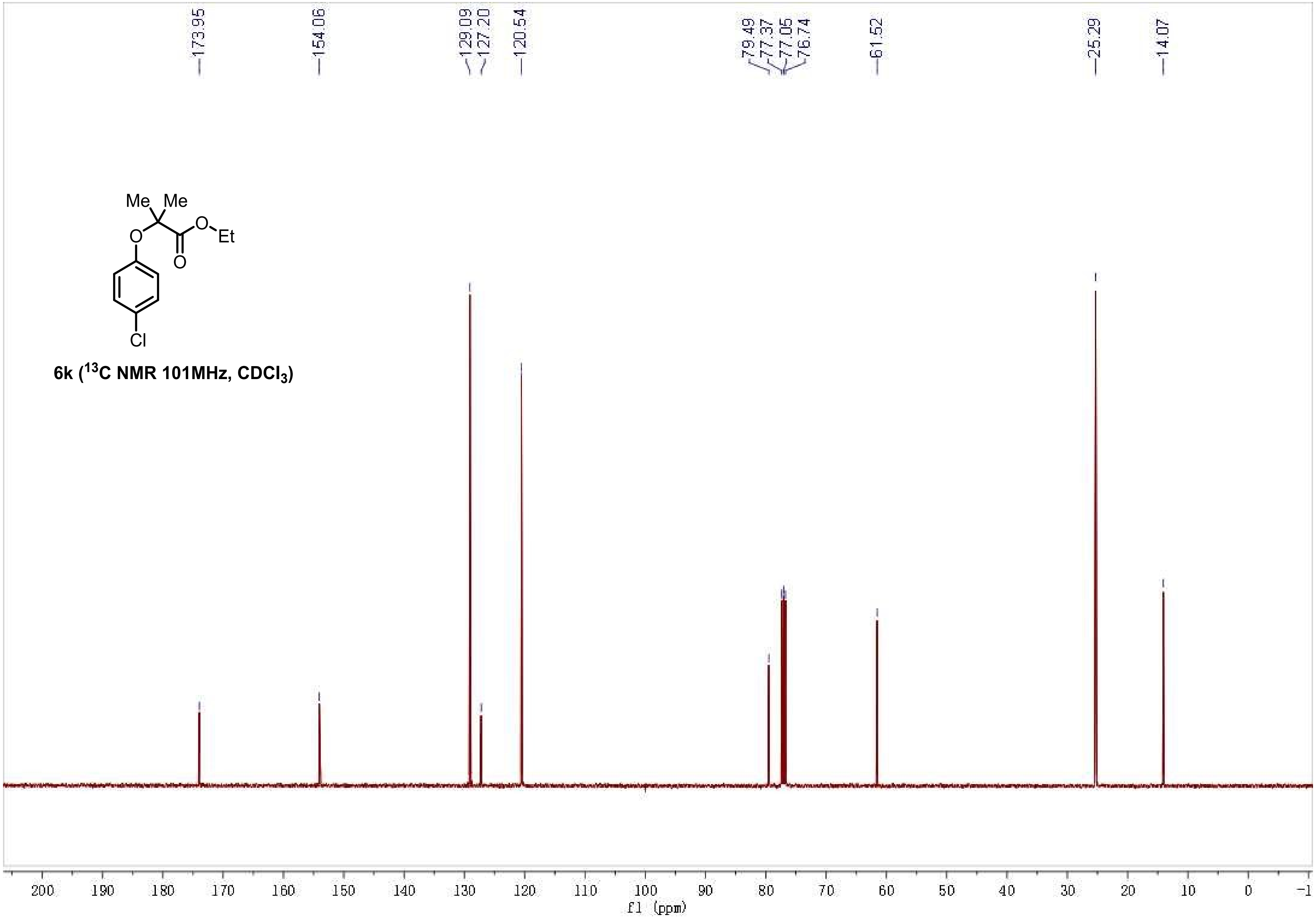

6k (^{13}C NMR 101MHz, $CDCl_3$)

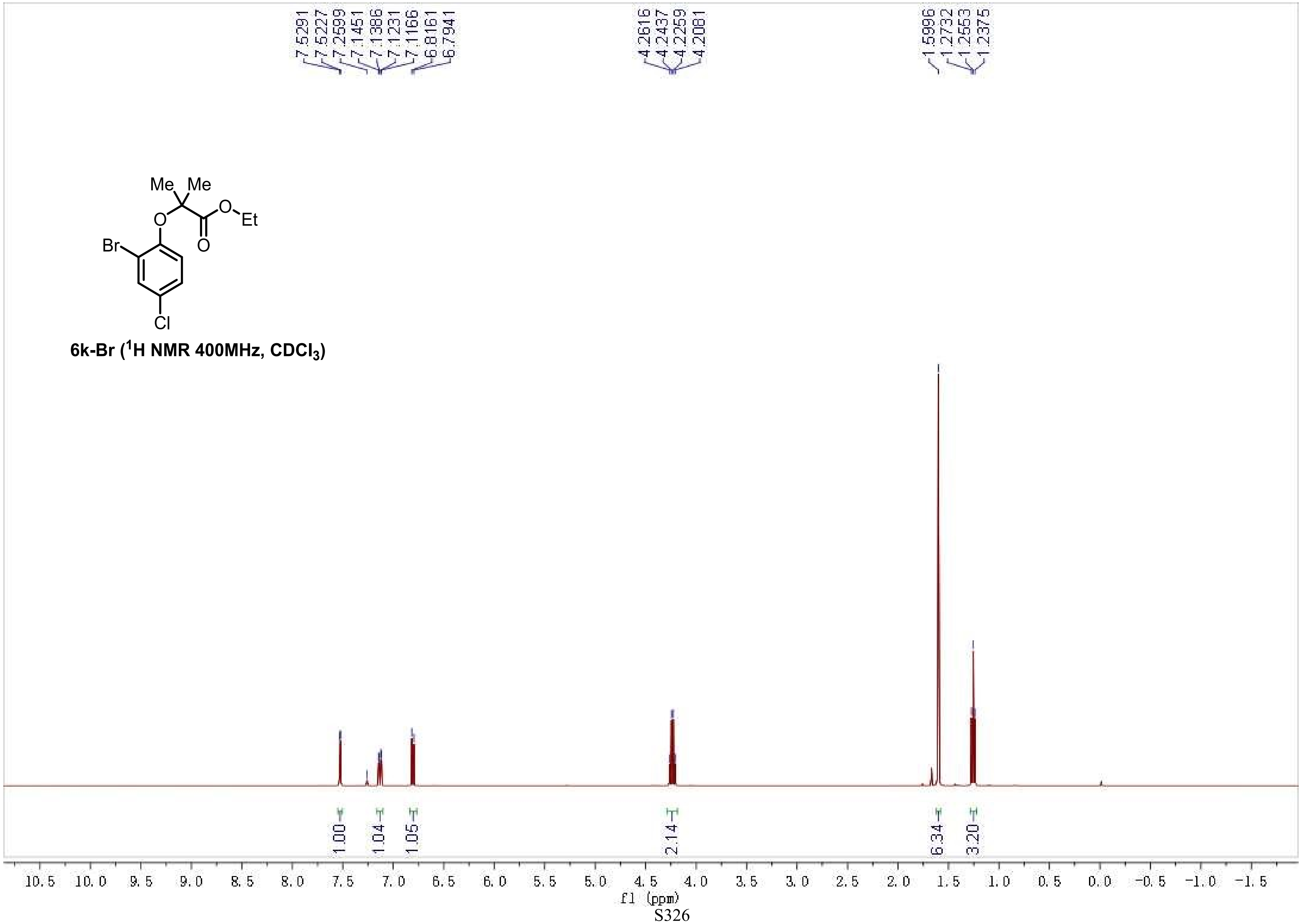

6k-Br (^{1}H NMR 400MHz, $CDCl_3$)

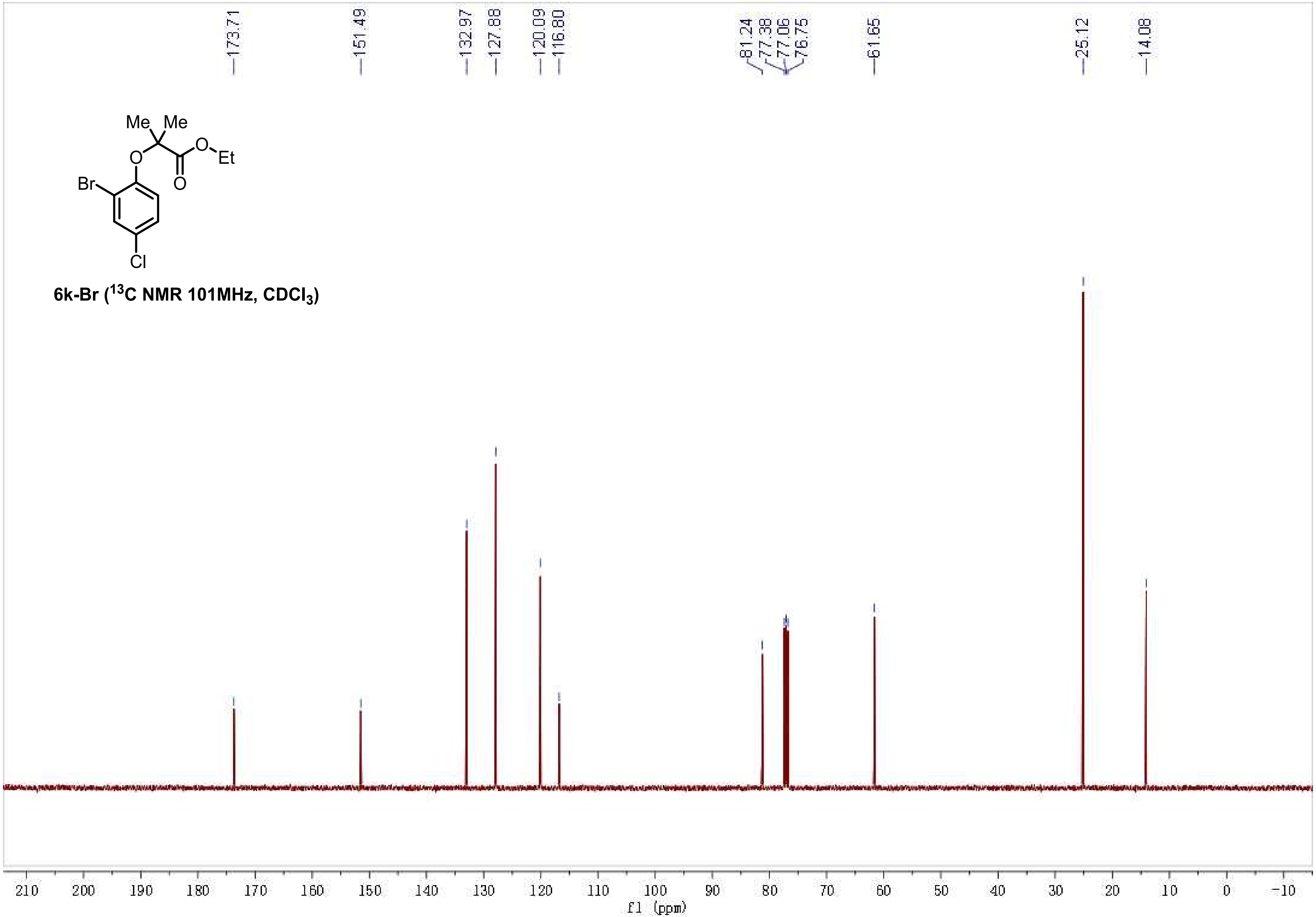

6k-Br (^{13}C NMR 101MHz, $CDCl_3$)

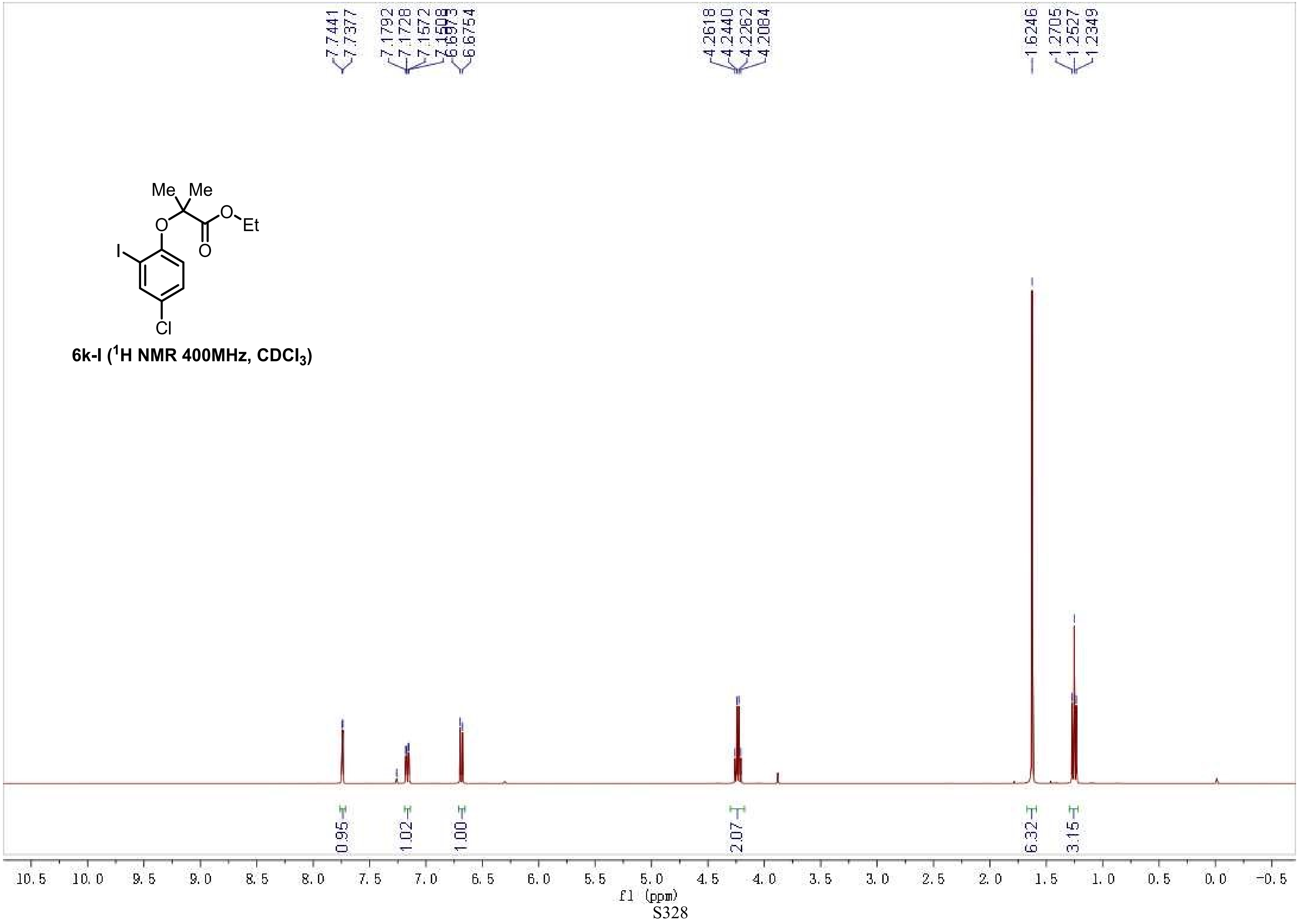
7.7441
7.7377
7.1792
7.1728
7.1572
7.1508
6.6973
6.6754
4.2618
4.2440
4.2262
4.2084
1.6246
1.2705
1.2527
1.2349
Me Me
O
O
Et
I
O
Cl
6k-I (^{1}H NMR 400MHz, $CDCl_3$)
0.95
1.02
1.00
2.07
6.32
3.15
10.5 10.0 9.5 9.0 8.5 8.0 7.5 7.0 6.5 6.0 5.5 5.0 4.5 4.0 3.5 3.0 2.5 2.0 1.5 1.0 0.5 0.0 -0.5
f1 (ppm)

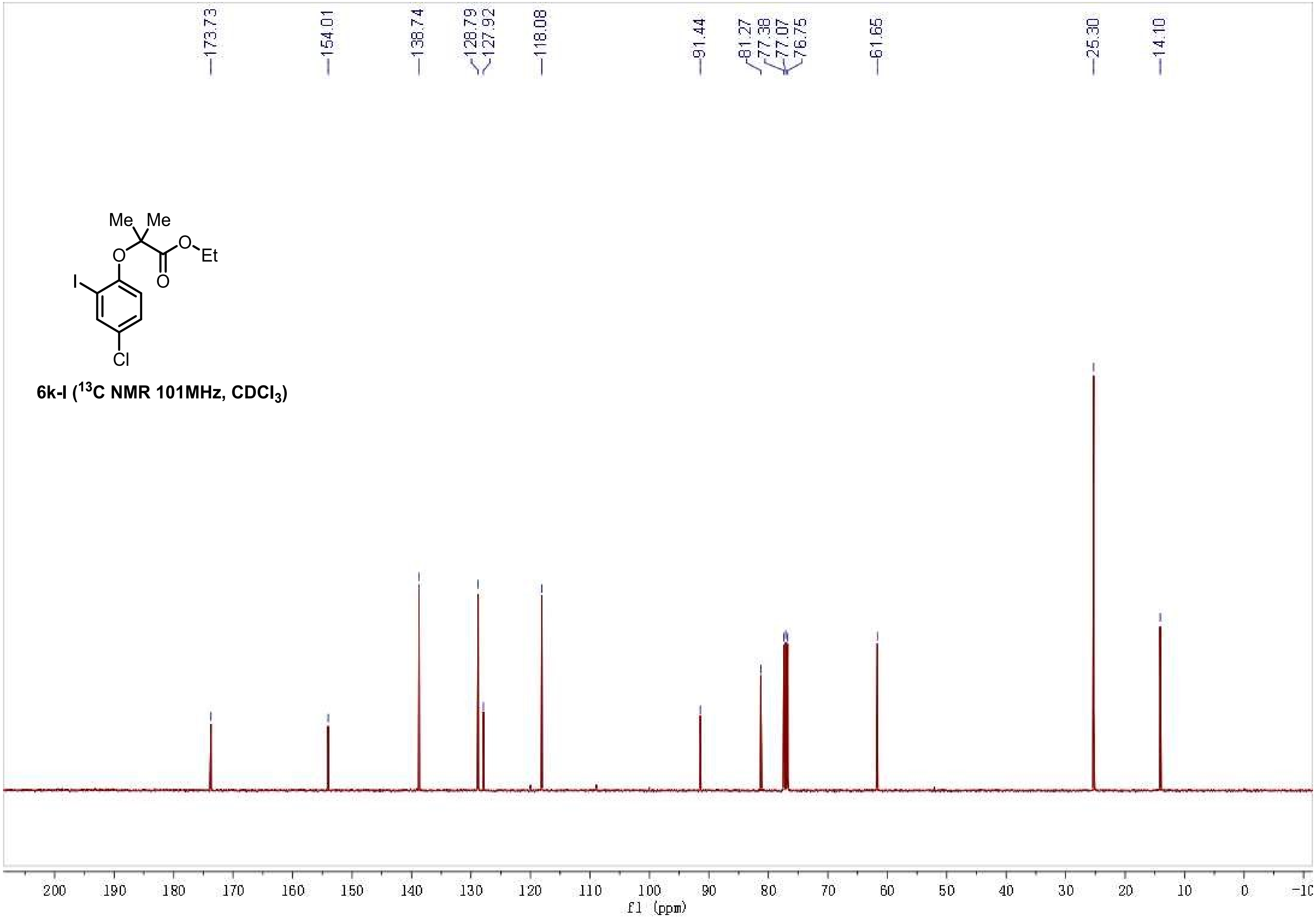

6k-I (^{13}C NMR 101MHz, $CDCl_3$)

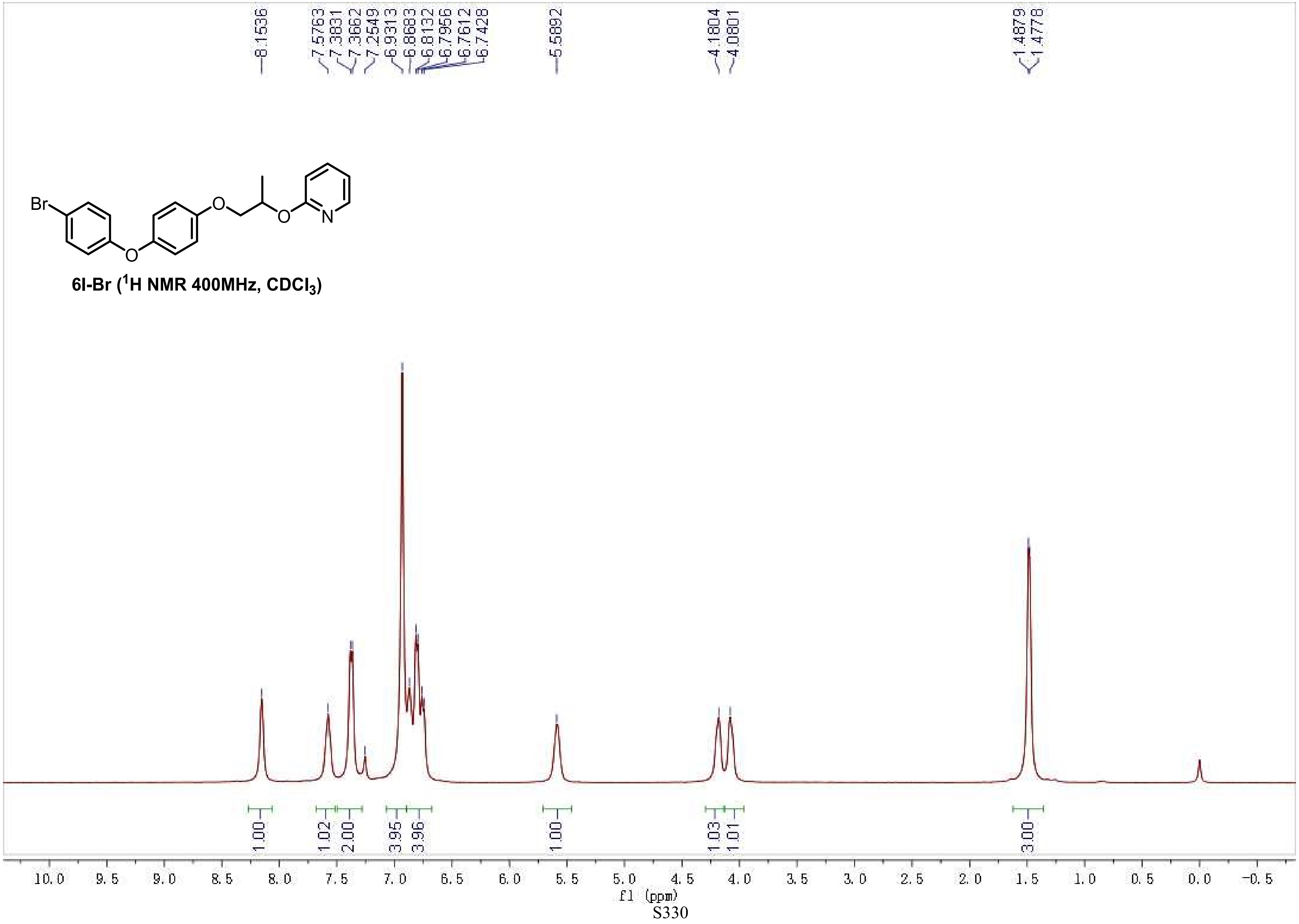

6l-Br (^{1}H NMR 400MHz, $CDCl_3$)

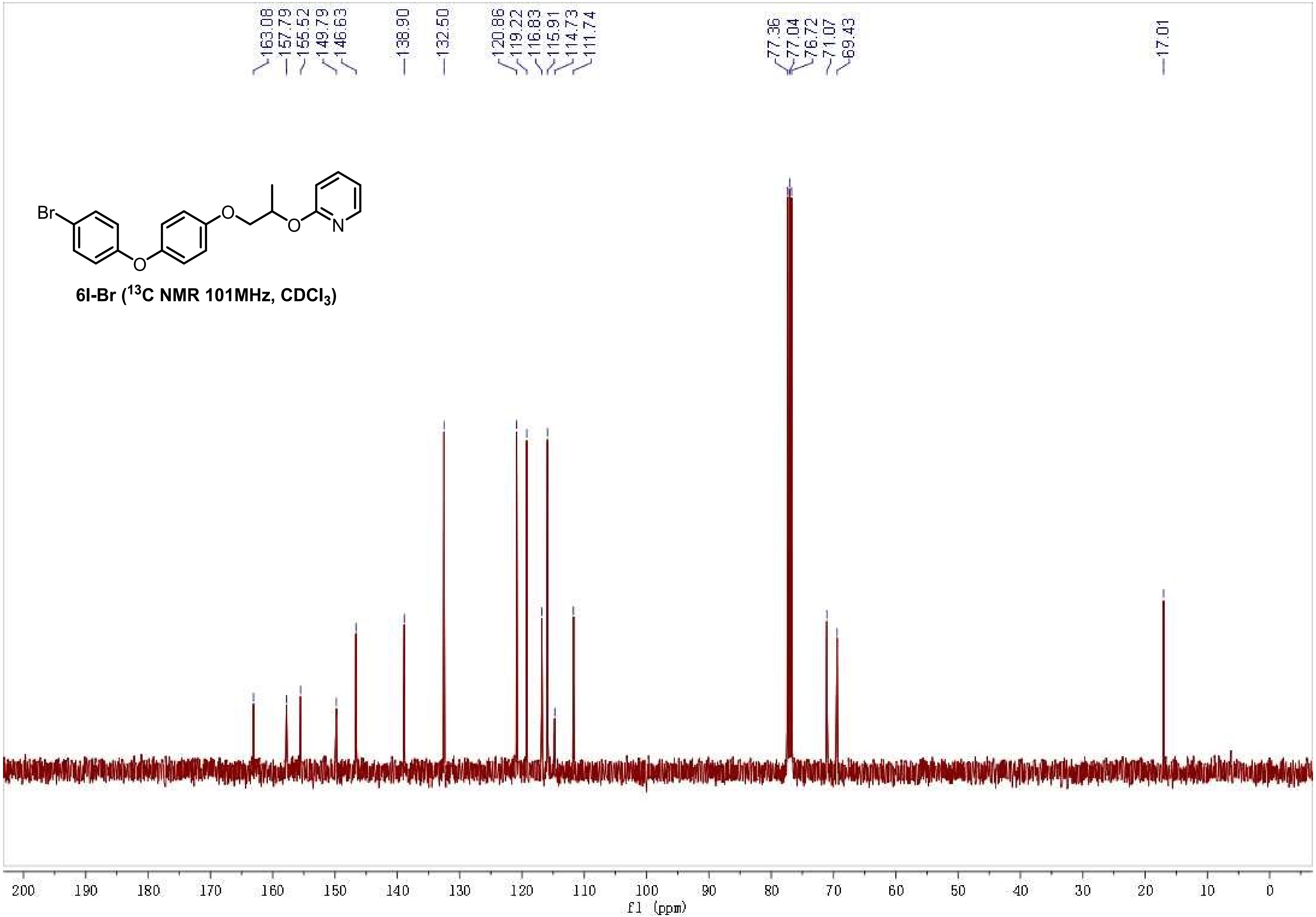

6l-Br (^{13}C NMR 101MHz, $CDCl_3$)

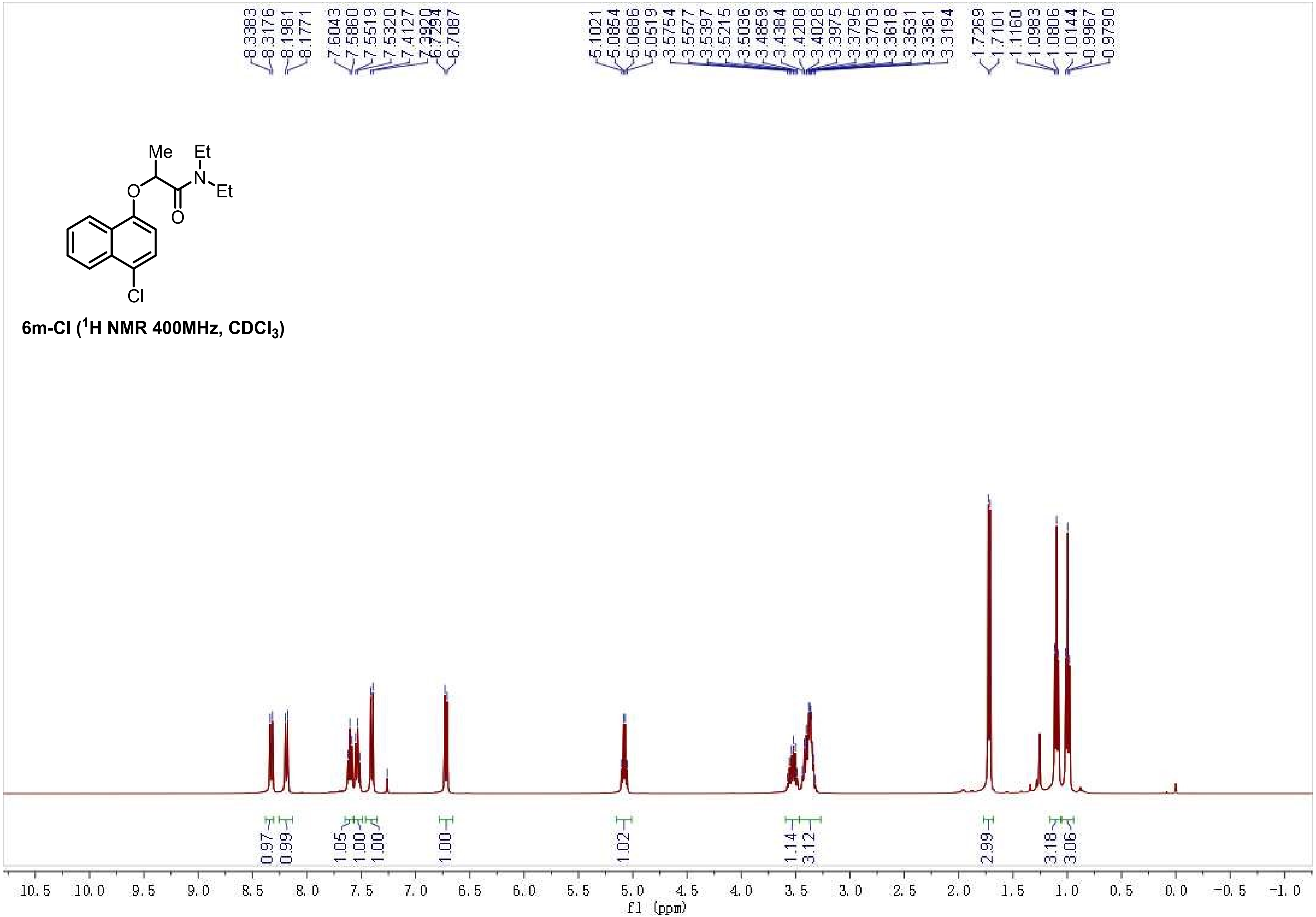

6m-Cl (^{1}H NMR 400MHz, $CDCl_3$)

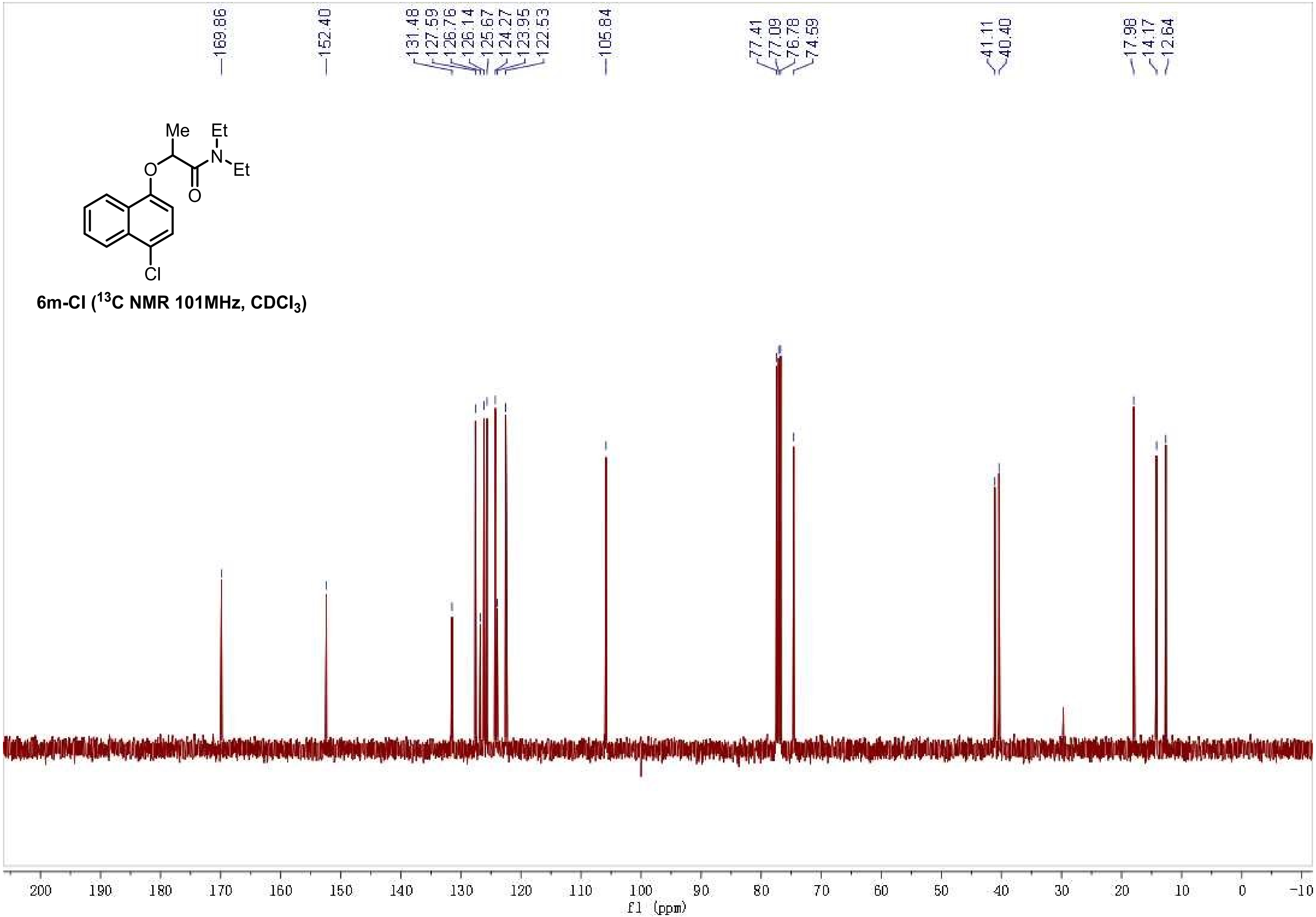

6m-Cl (^{13}C NMR 101MHz, $CDCl_3$)

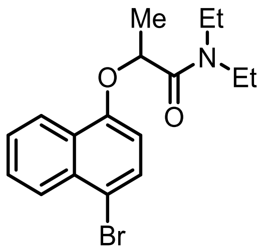

6m-Br (^{1}H NMR 400MHz, $CDCl_3$)

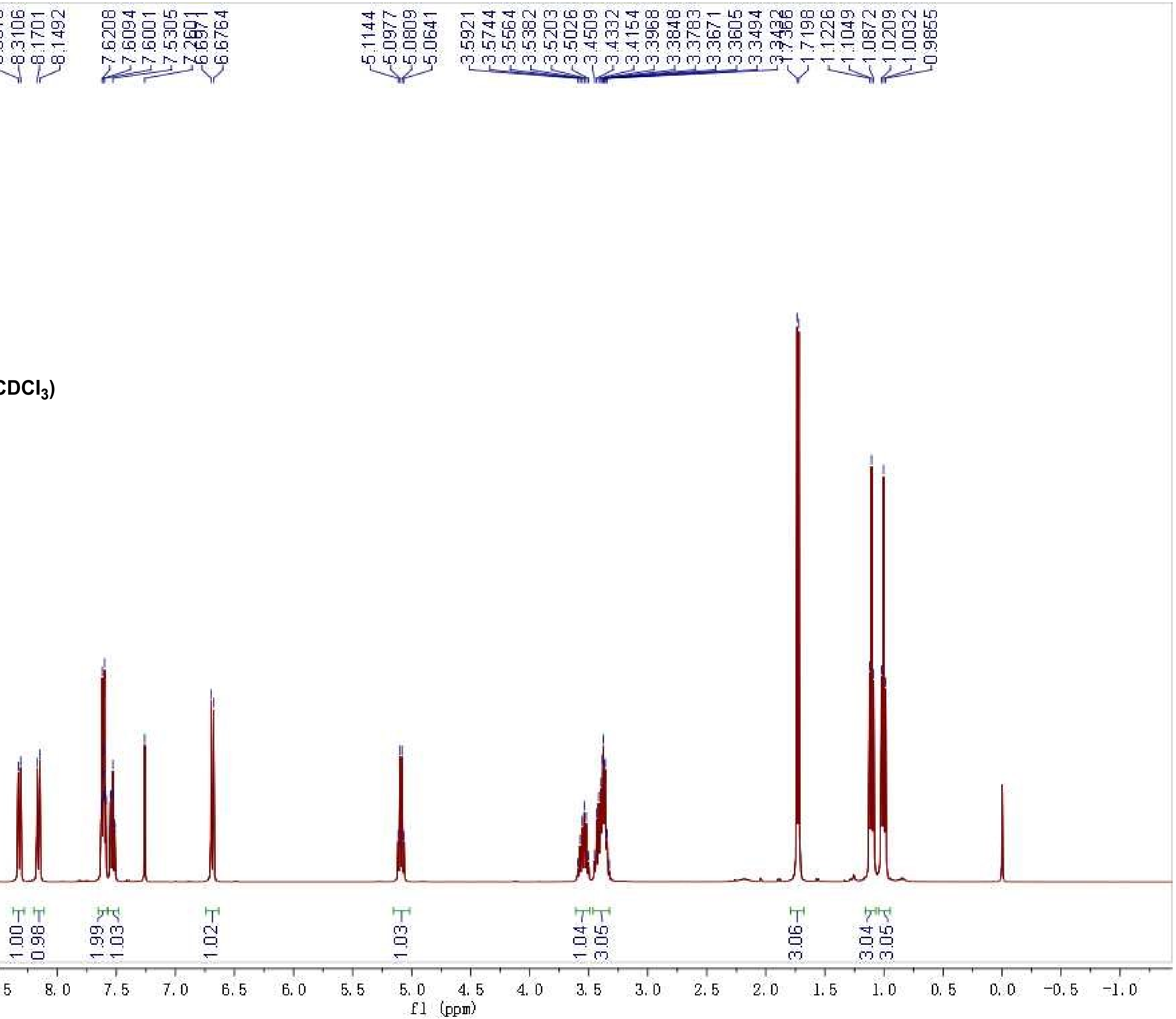

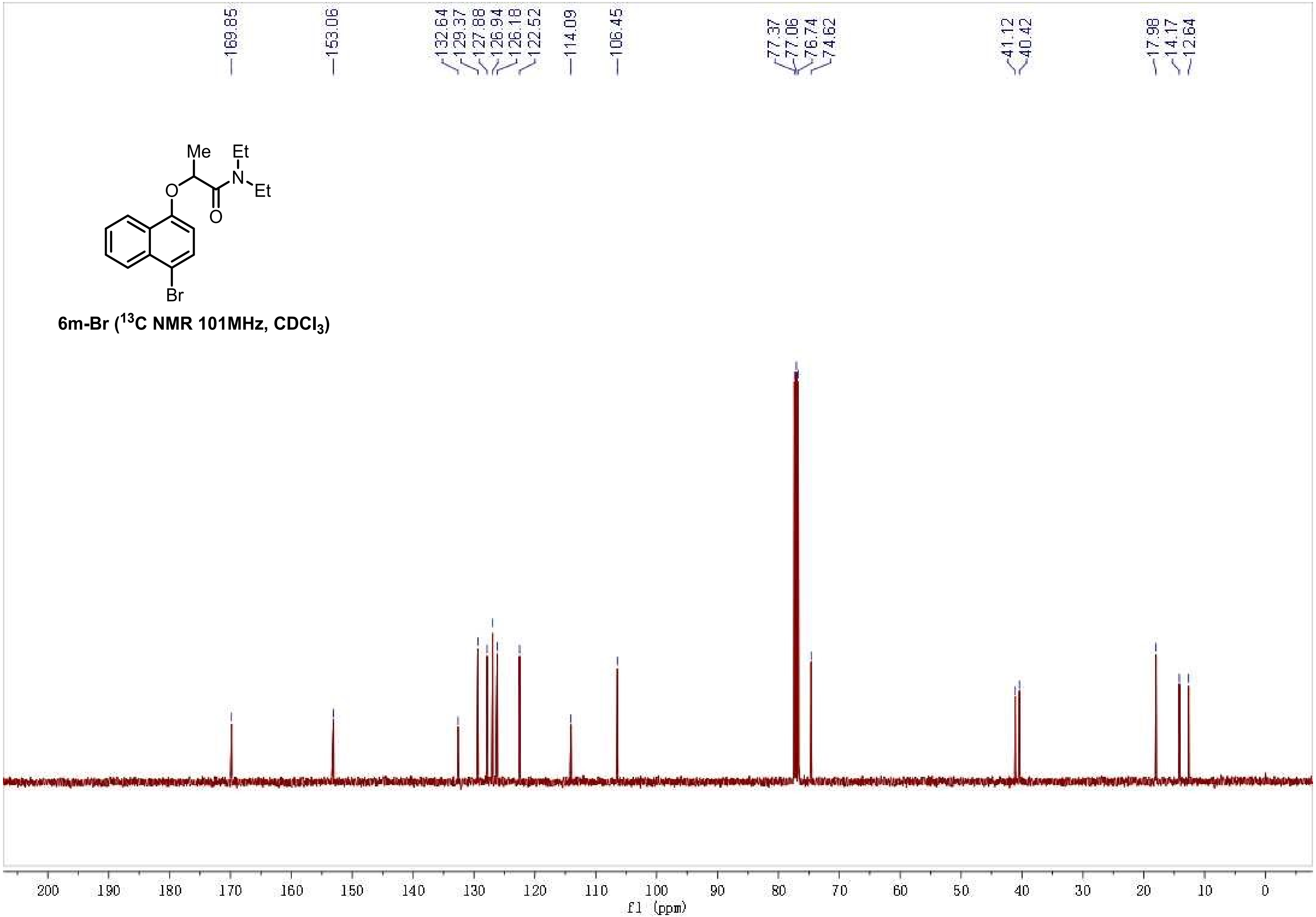

169.85
153.06
132.64
129.37
127.88
126.94
126.18
122.52
114.09
106.45
77.37
77.06
76.74
74.62
41.12
40.42
17.98
14.17
12.64
Me
Et
O
N
Et
O
Br
6m-Br (^{13}C NMR 101MHz, CDCl$_3$)
200
190
180
170
160
150
140
130
120
110
100
90
80
70
60
50
40
30
20
10
0
f1 (ppm)

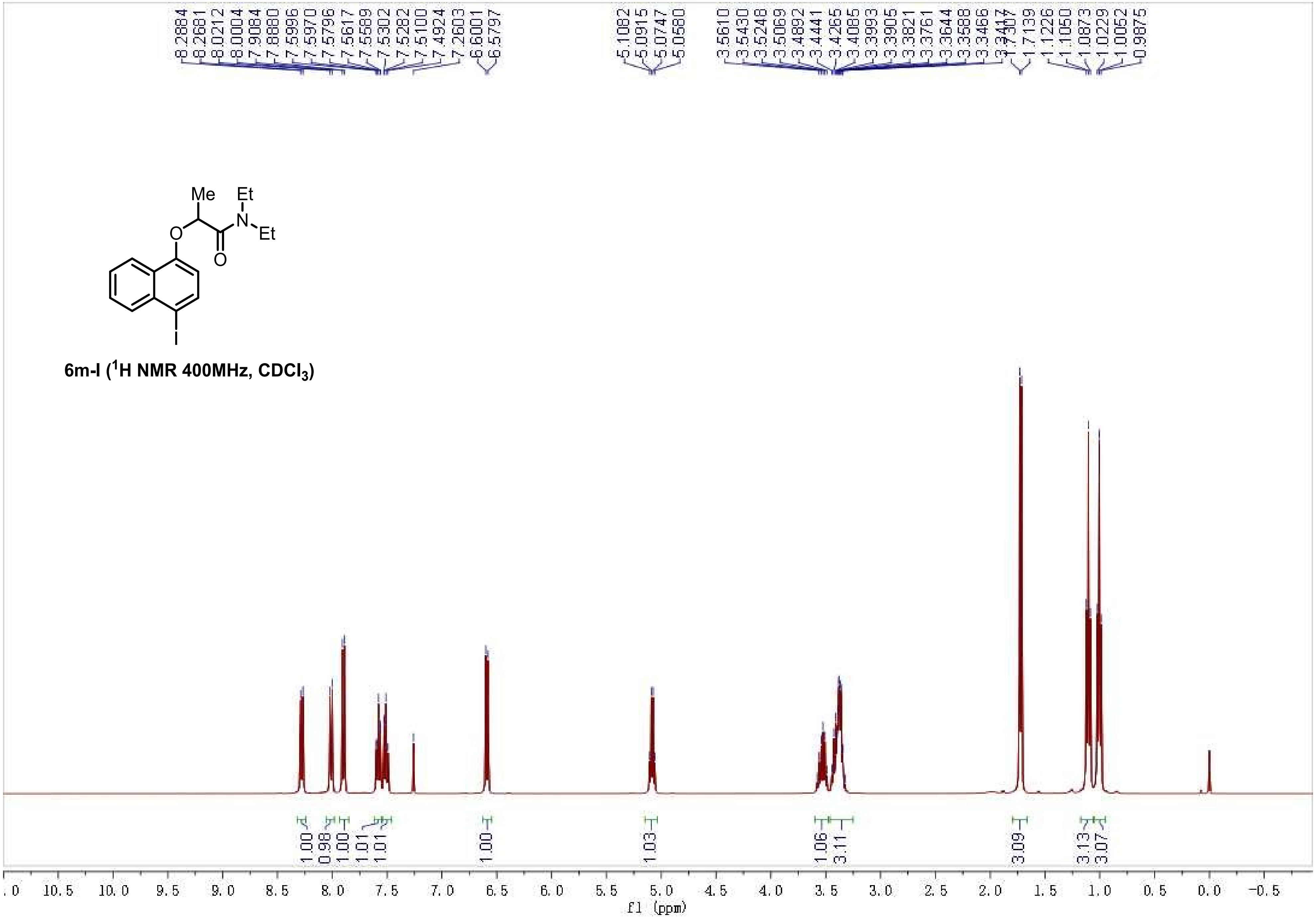

6m-I (^{1}H NMR 400MHz, $CDCl_3$)

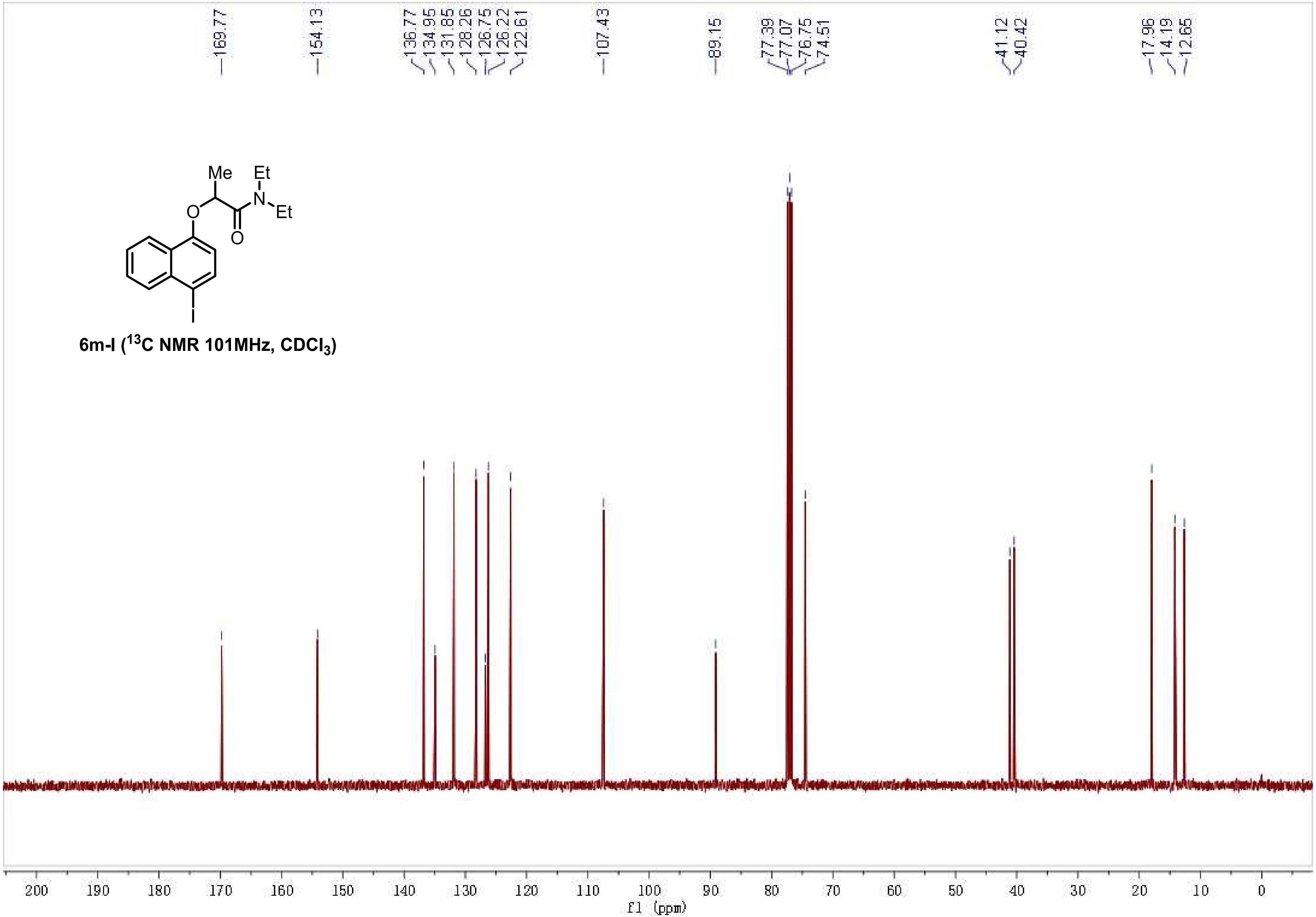
169.77
154.13
136.77
134.95
131.85
128.26
126.75
126.22
122.61
107.43
89.15
77.39
77.07
76.75
74.51
41.12
40.42
17.96
14.19
12.65
Me
Et
N
Et
O
O
I
6m-I (^{13}C NMR 101MHz, $CDCl_3$)
200 190 180 170 160 150 140 130 120 110 100 90 80 70 60 50 40 30 20 10 0
f1 (ppm)

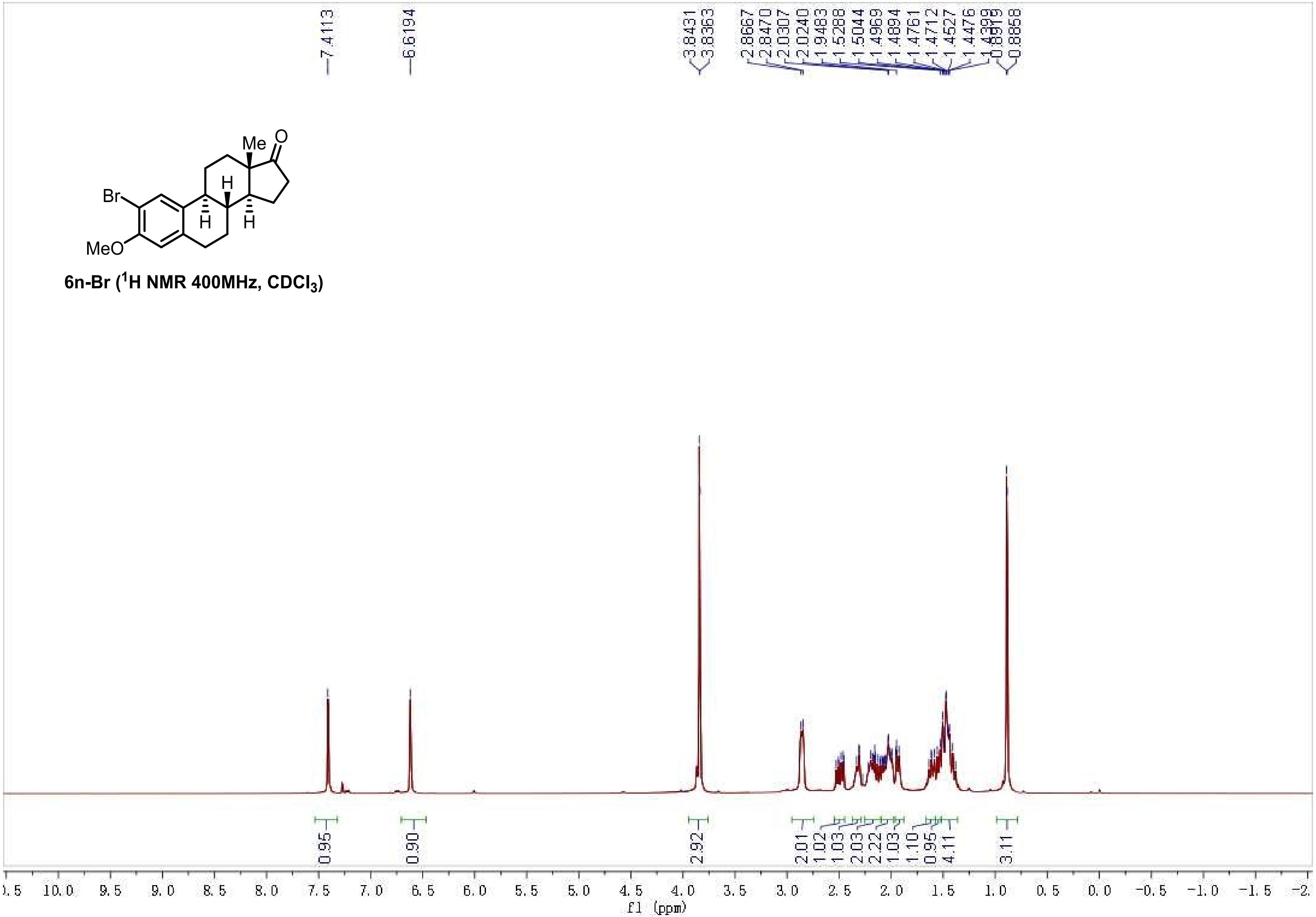

6n-Br (^1H NMR 400MHz, $CDCl_3$)

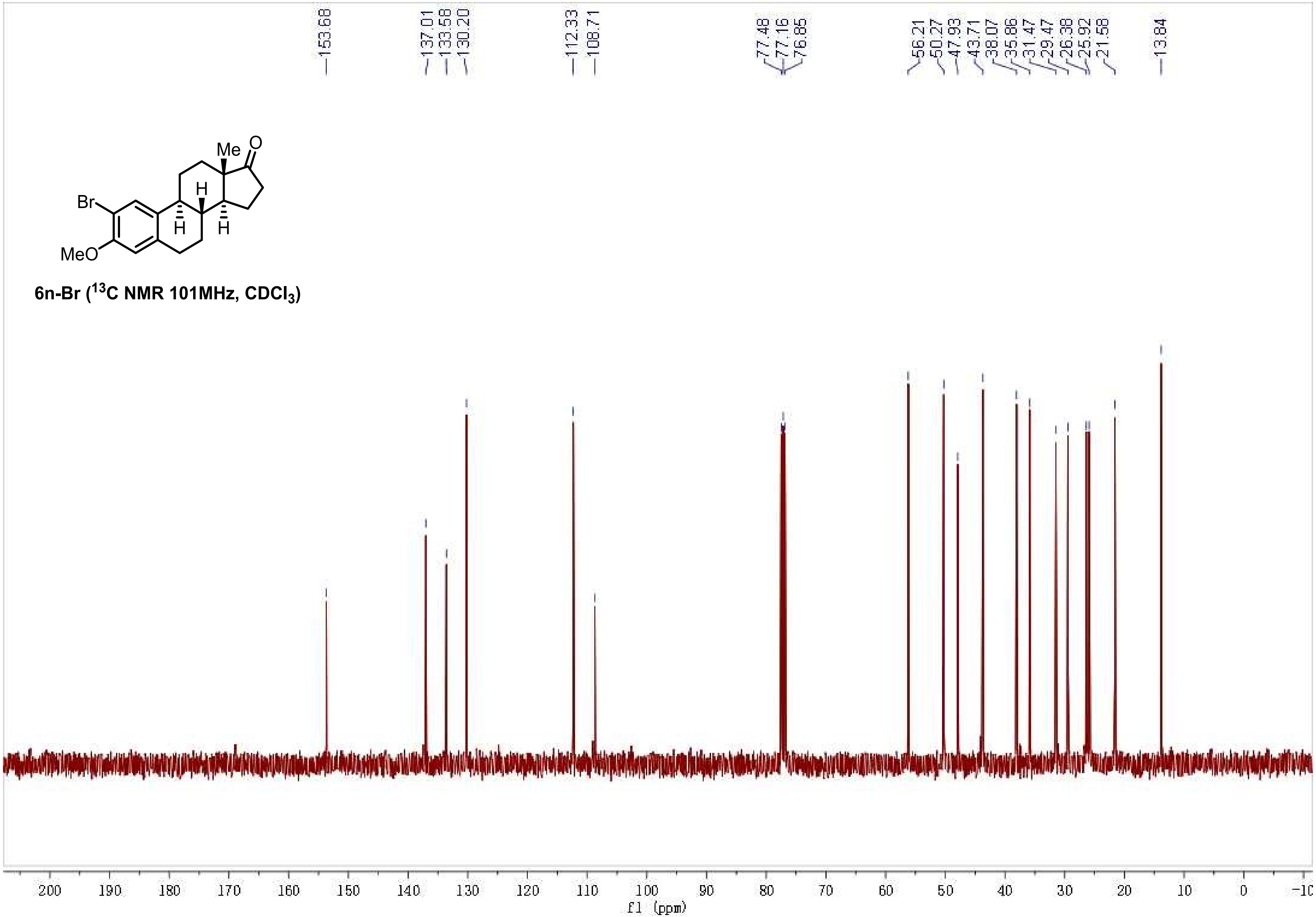

153.68
137.01
133.58
130.20
112.33
108.71
77.48
77.16
76.85
56.21
50.27
47.93
43.71
38.07
35.86
31.47
29.47
26.38
25.92
21.58
13.84
Me
O
Br
H
H
H
MeO
6n-Br (^{13}C NMR 101MHz, $CDCl_3$)
200
190
180
170
160
150
140
130
120
110
100
90
80
70
60
50
40
30
20
10
0
-10
f1 (ppm)

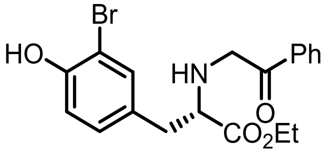

6o-Br (^{1}H NMR 400MHz, $CDCl_3$)

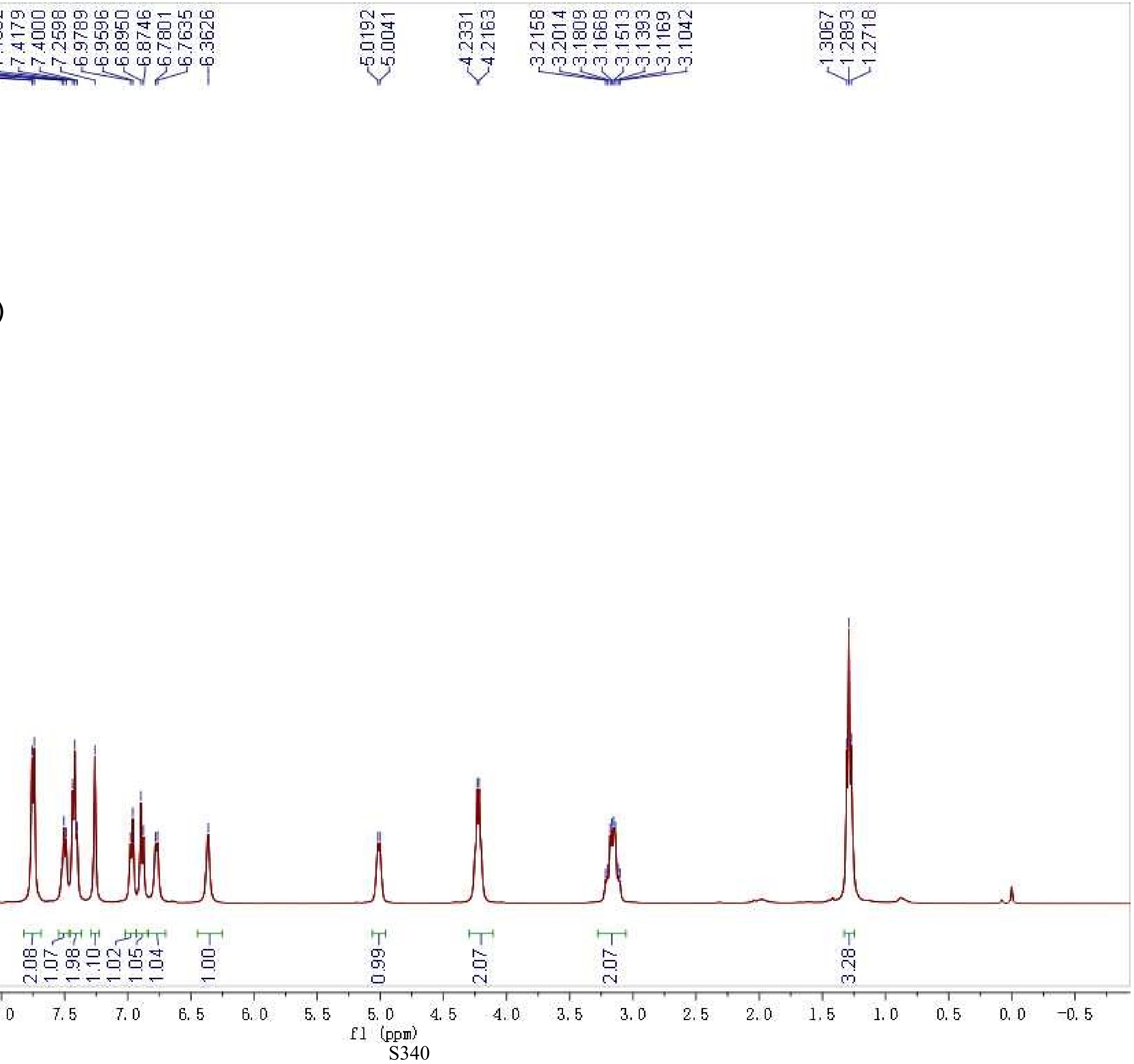

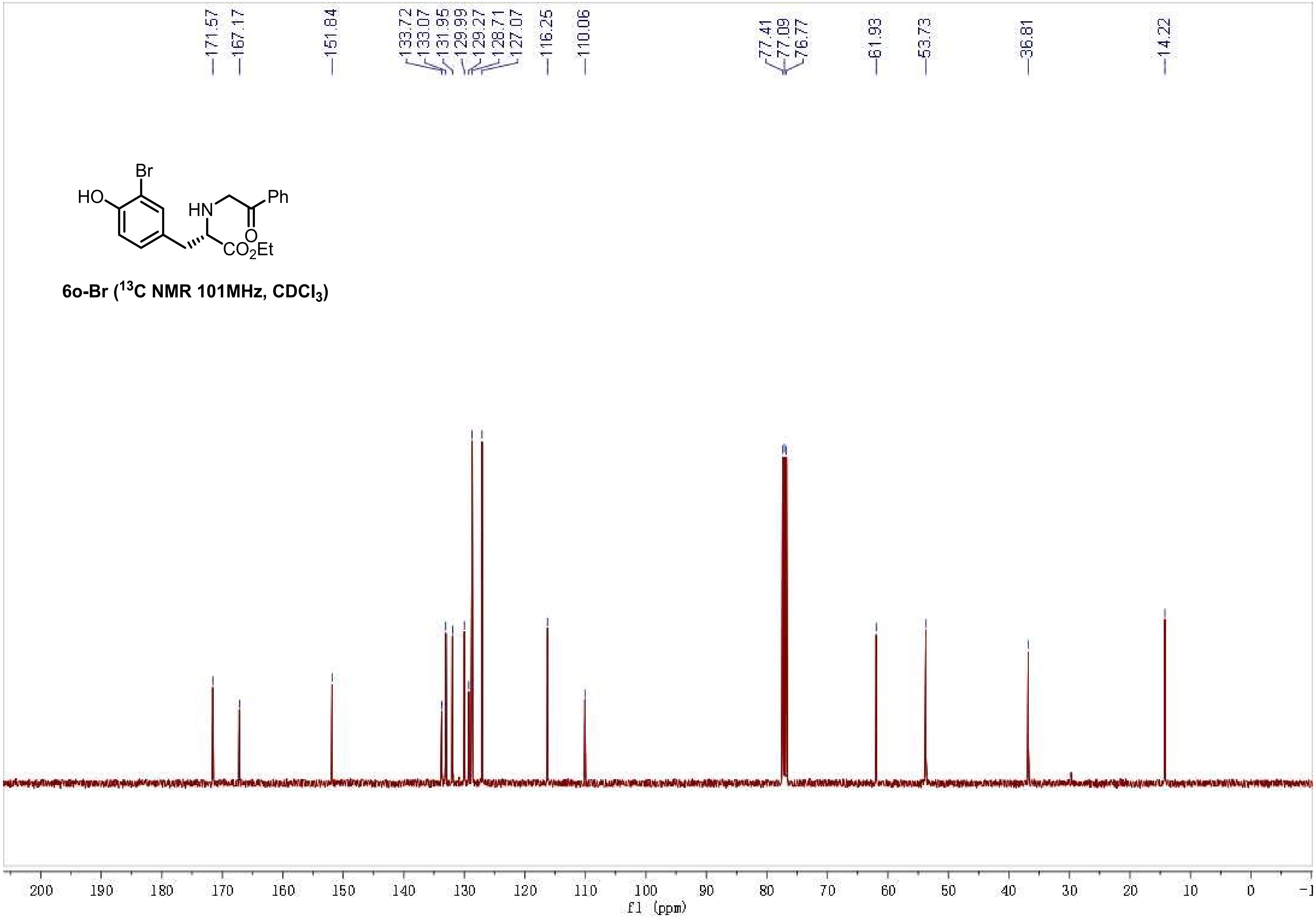

6o-Br (^{13}C NMR 101MHz, $CDCl_3$)

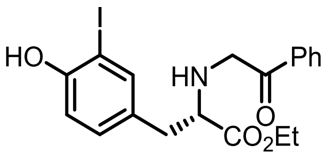

6o-I (^{1}H NMR 400MHz, $CDCl_3$)

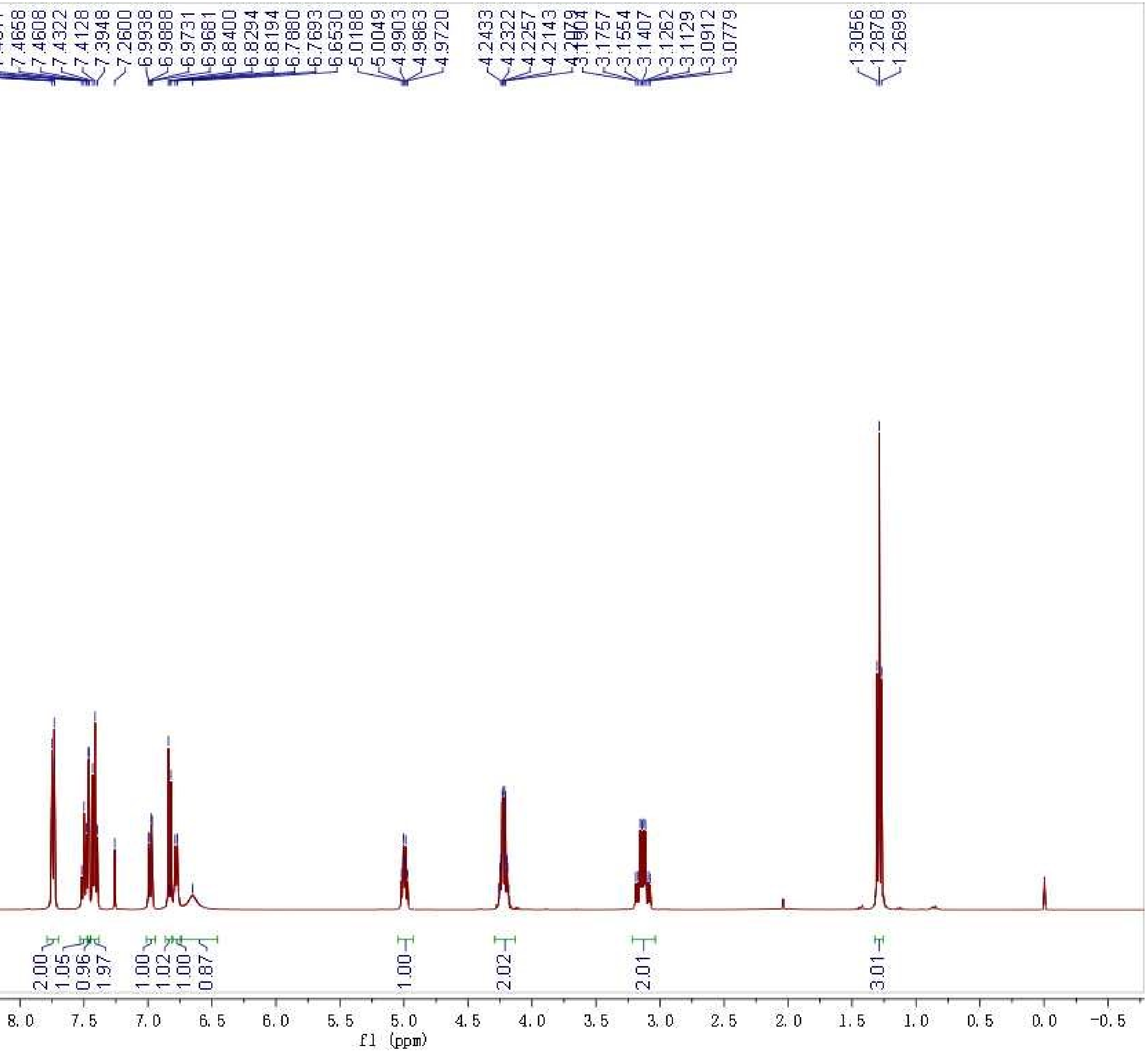

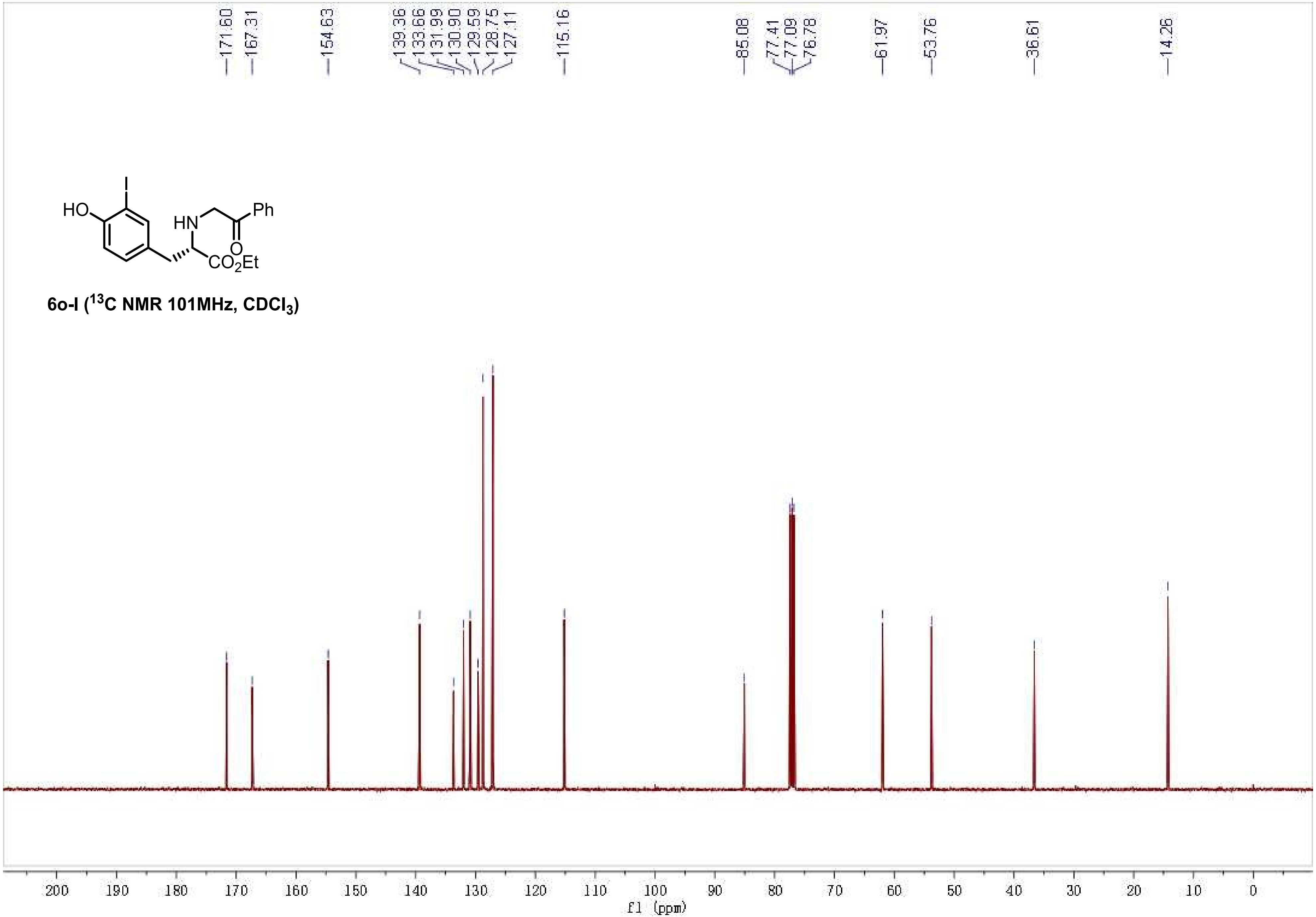

6o-I (^{13}C NMR 101MHz, $CDCl_3$)

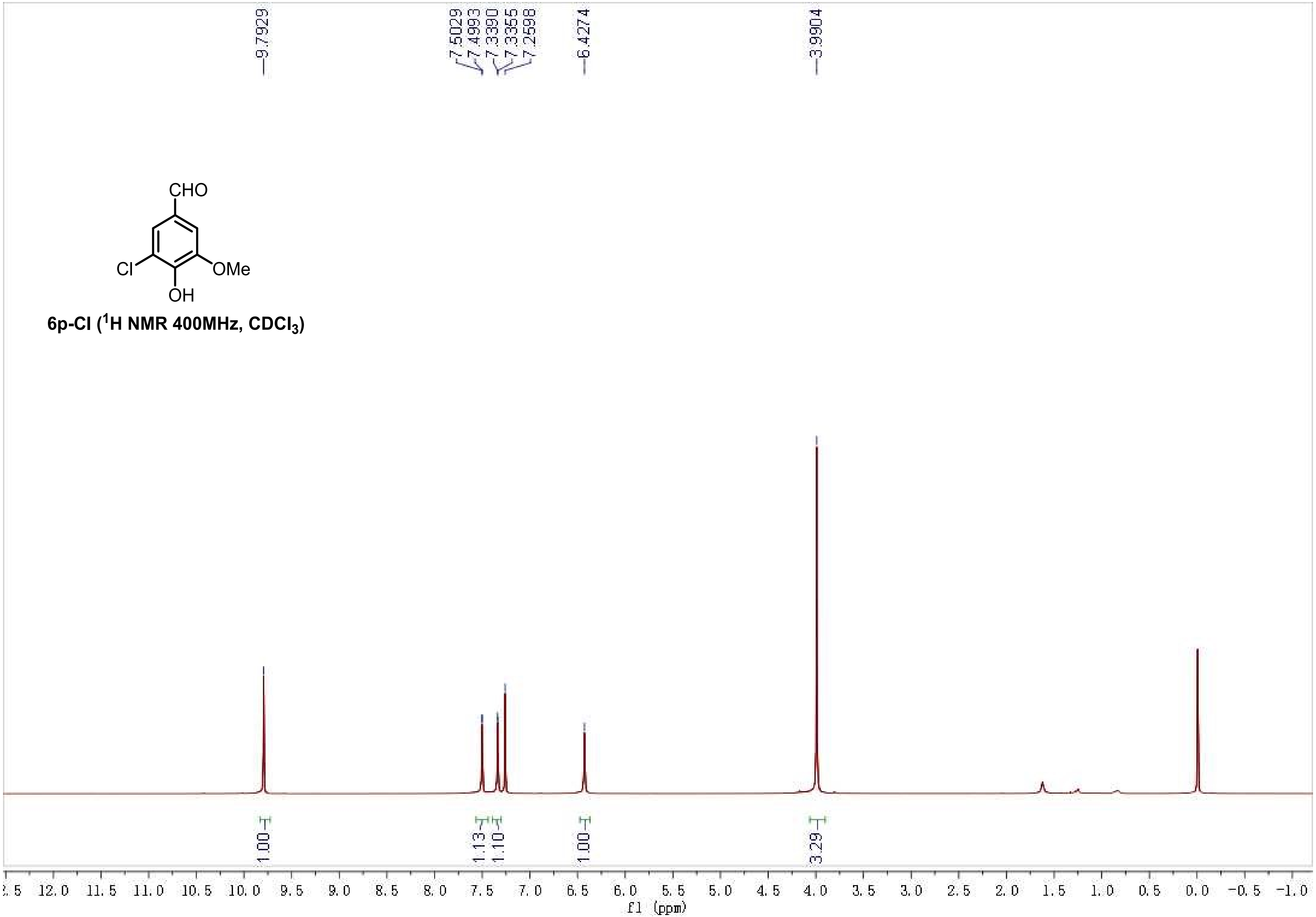

6p-Cl (^{1}H NMR 400MHz, $CDCl_3$)

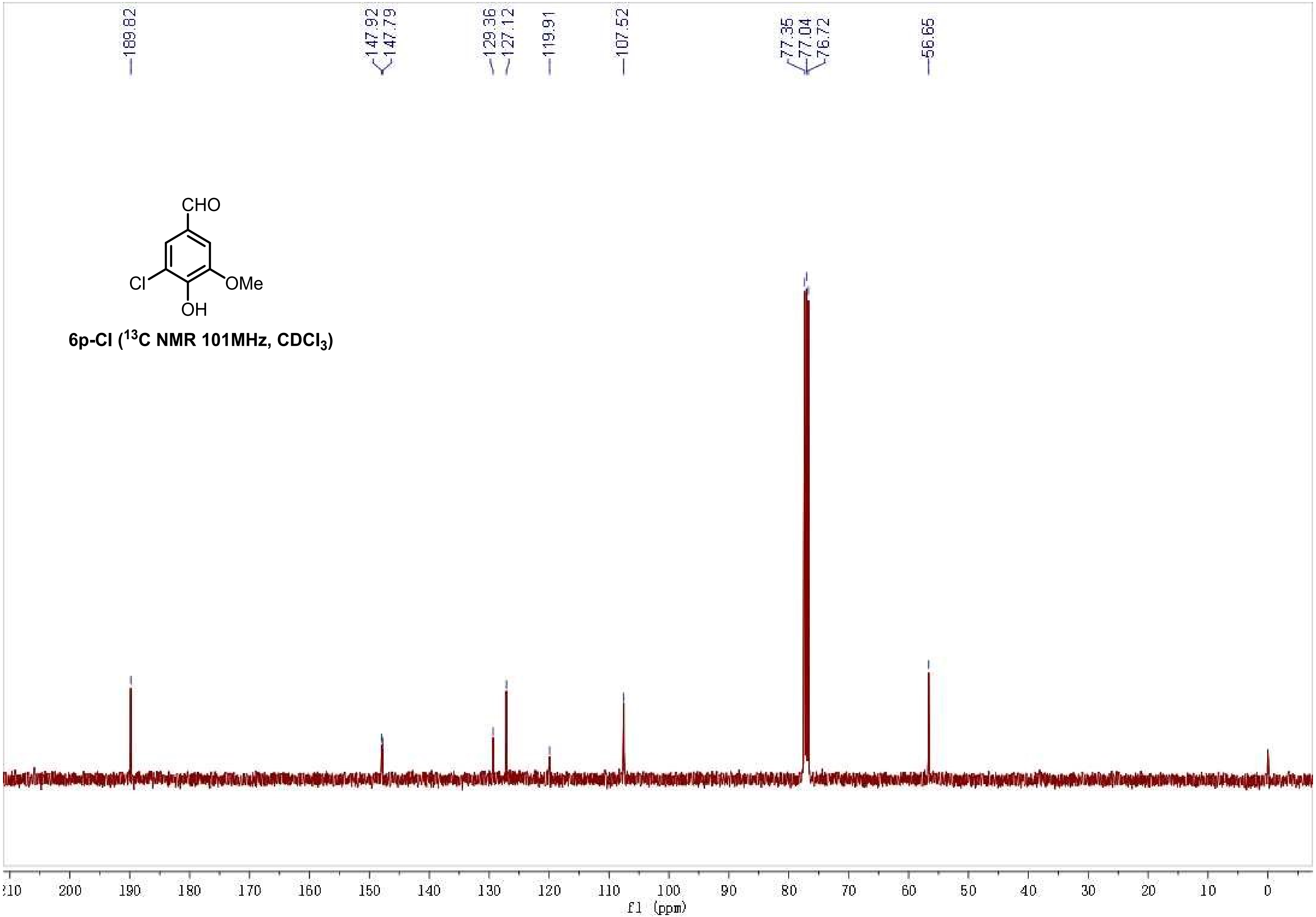

6p-Cl (^{13}C NMR 101MHz, $CDCl_3$)

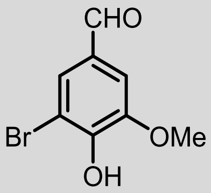

6p-Br (^{1}H NMR 400MHz, CDCl$_3$)

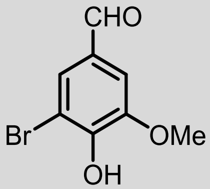

6p-Br (^{13}C NMR 101MHz, $CDCl_3$)

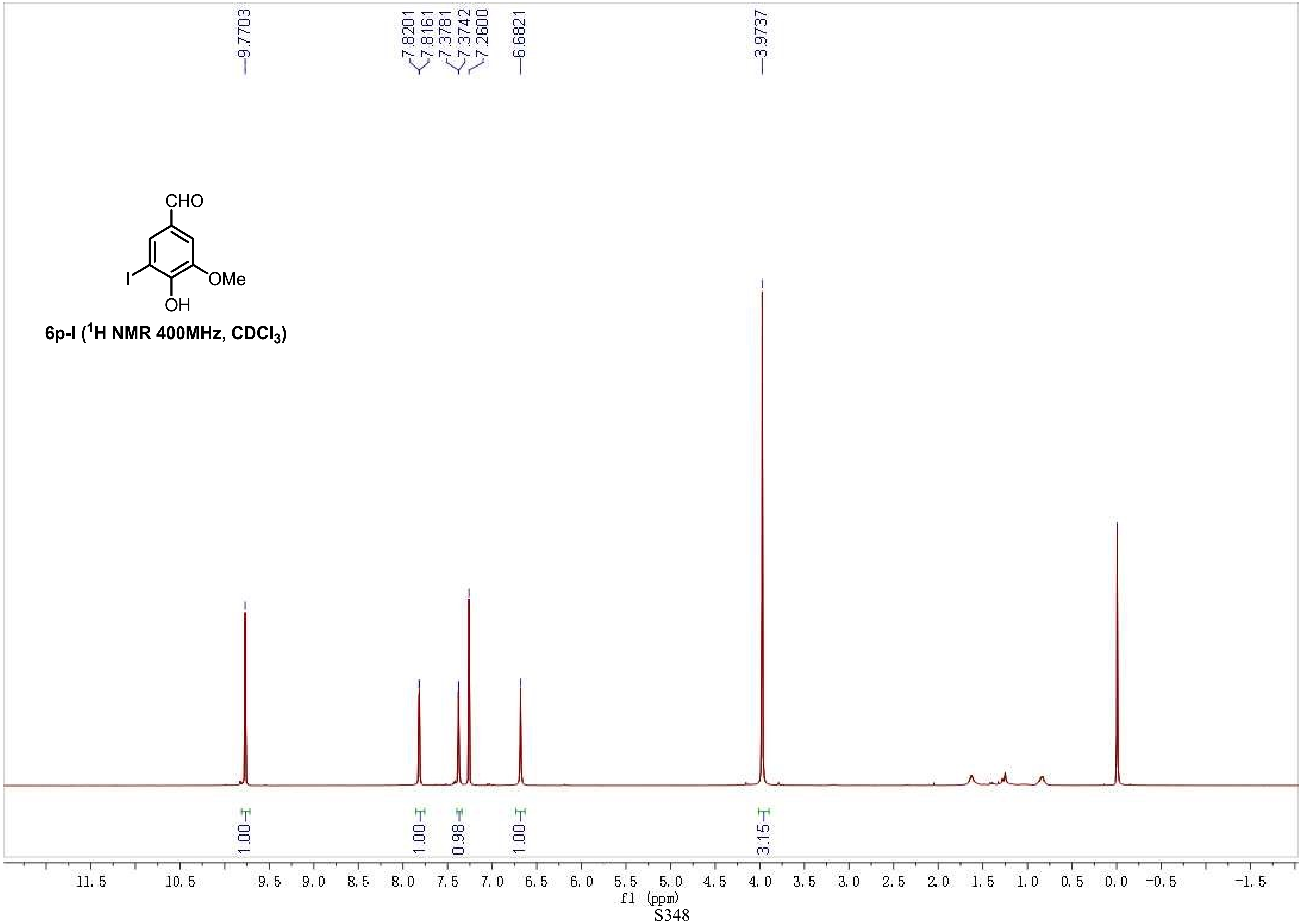

6p-I (^{1}H NMR 400MHz, $CDCl_3$)

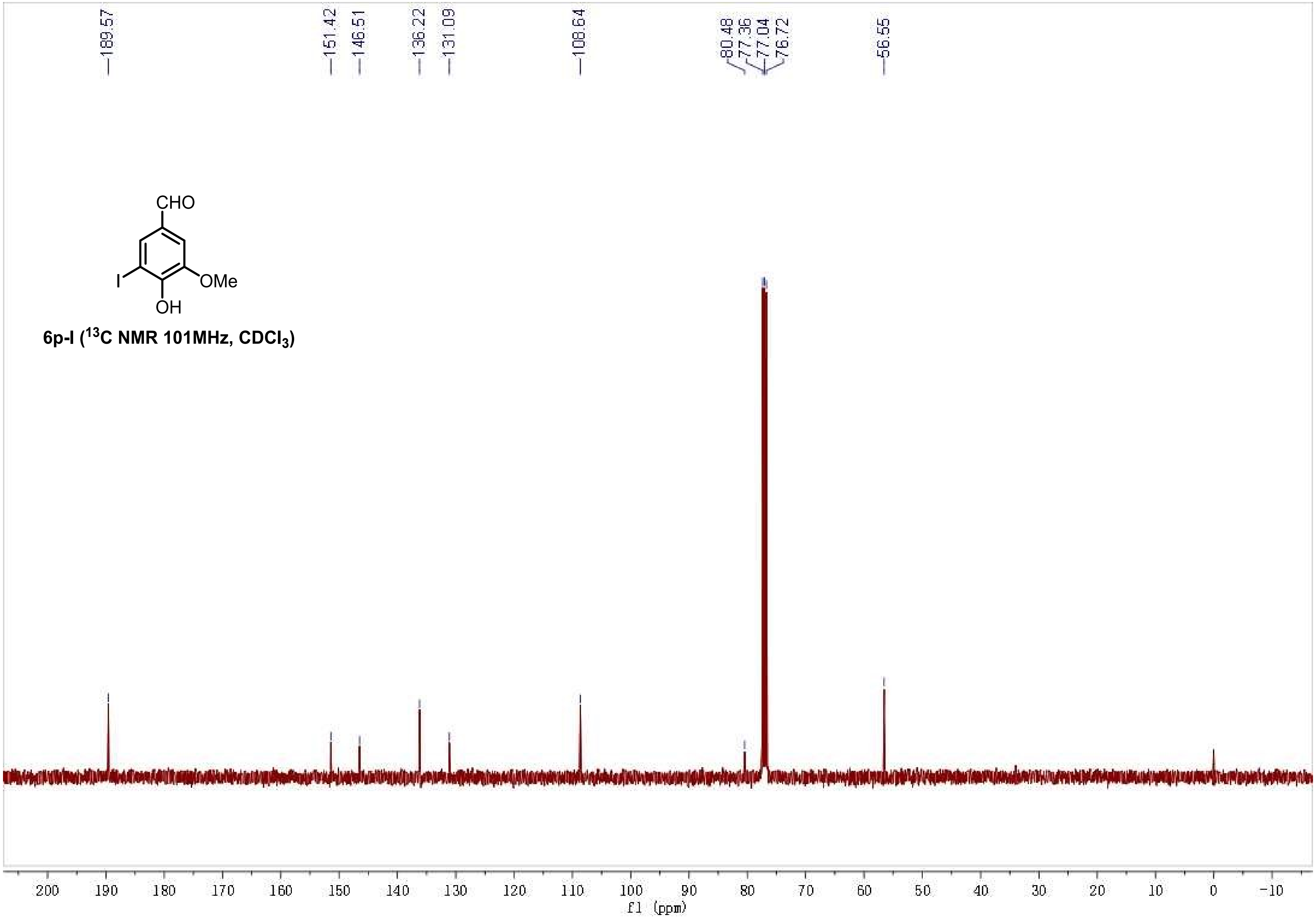

6p-I (^{13}C NMR 101MHz, $CDCl_3$)

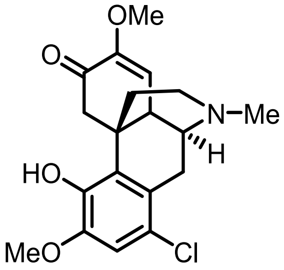

6q-Cl (^1H NMR 400MHz, $CDCl_3$)

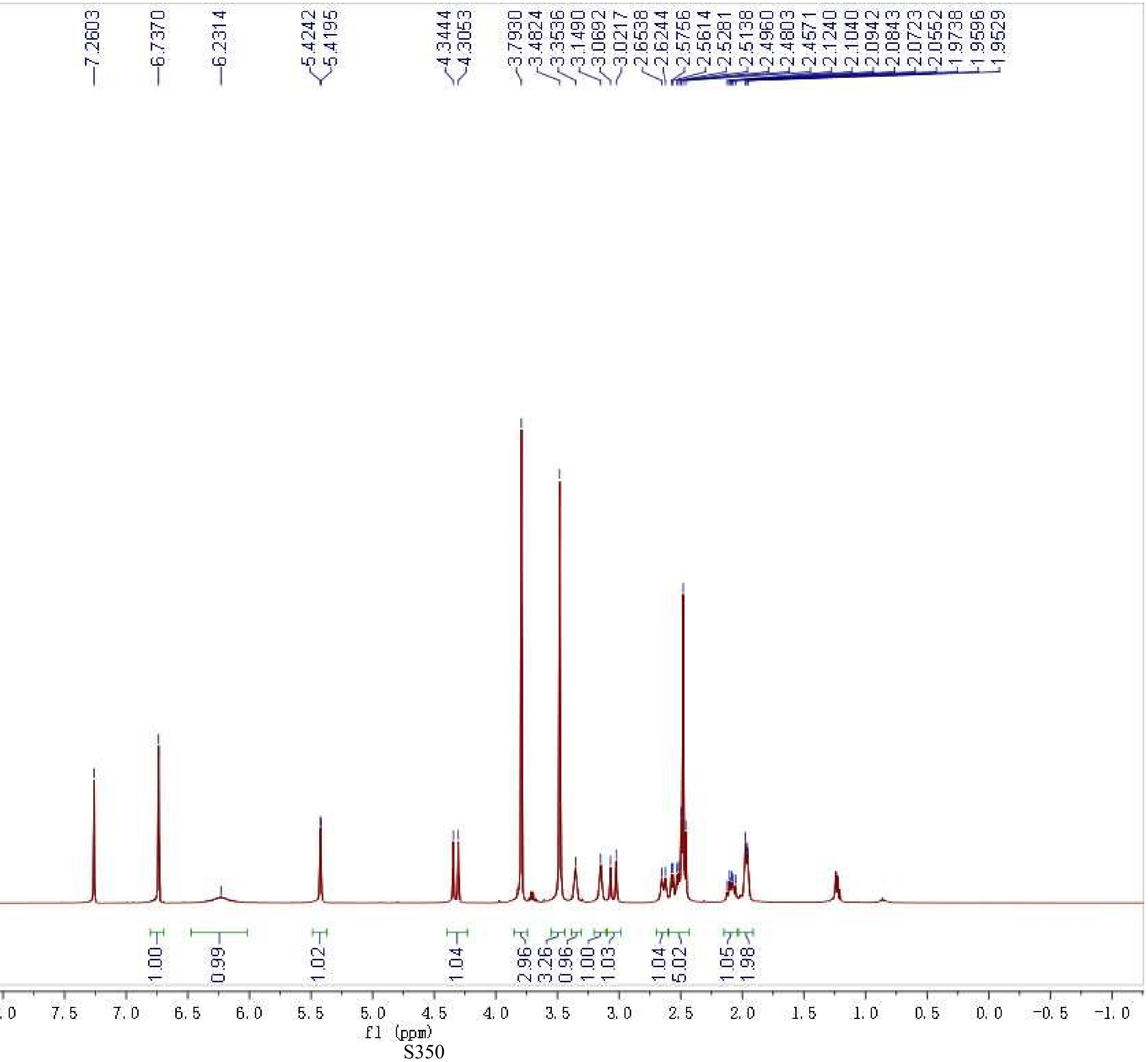

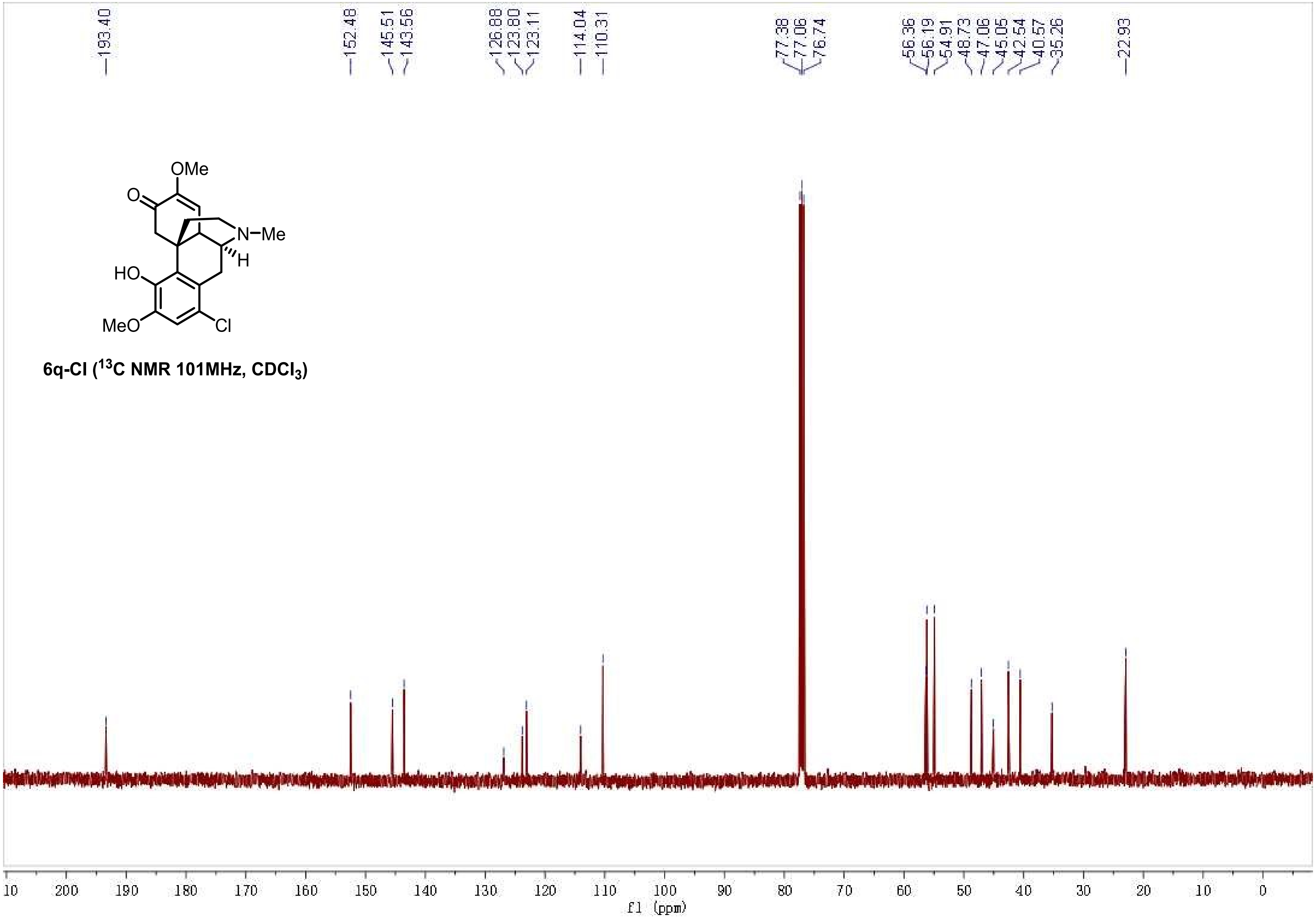

6q-Cl (^{13}C NMR 101MHz, $CDCl_3$)

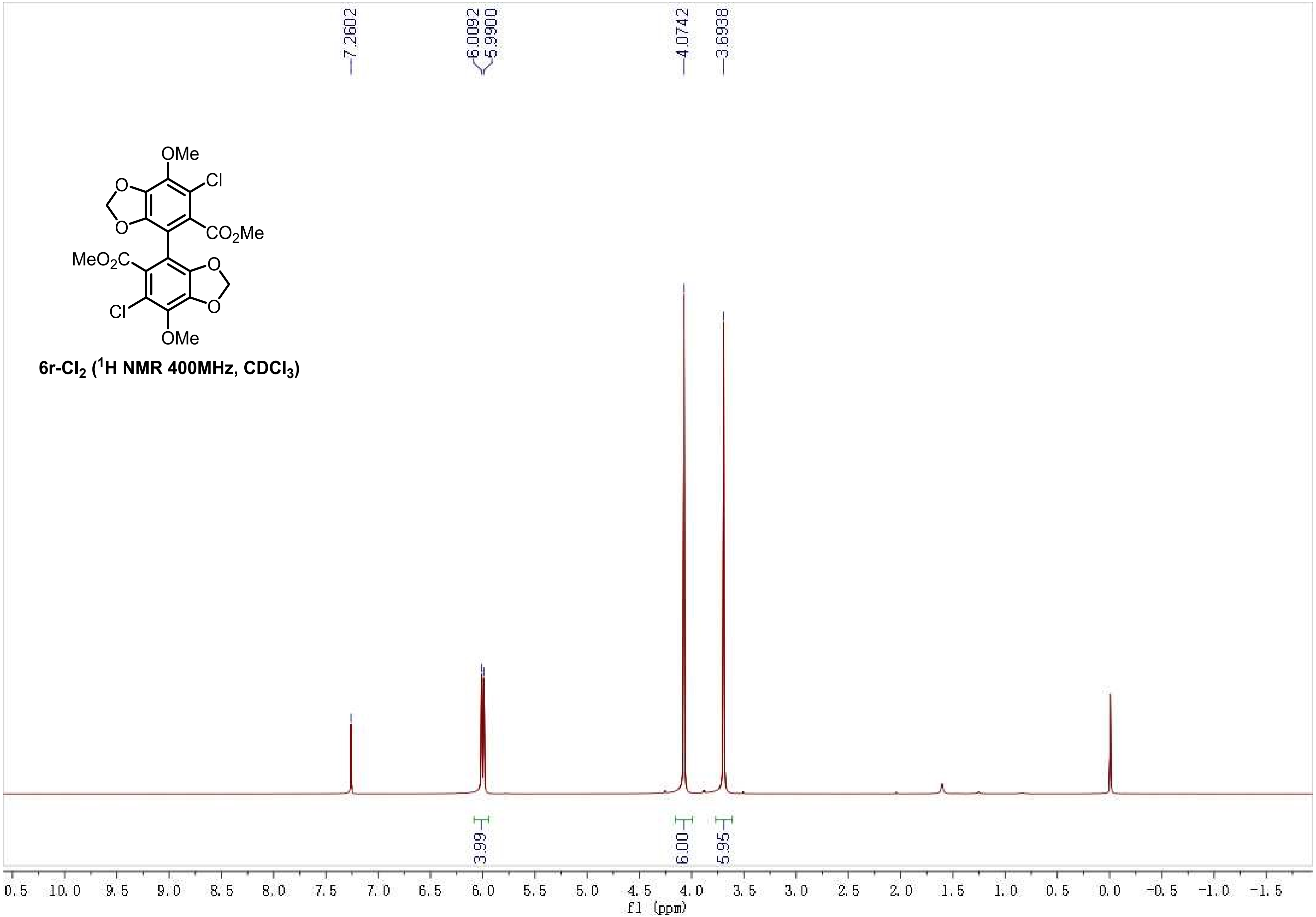

7.2602
6.0092
5.9900
4.0742
3.6938
OMe
Cl
O
O
CO2Me
MeO2C
O
O
Cl
OMe
6r-Cl2 (1H NMR 400MHz, CDCl3)
3.99
6.00
5.95
0.5 10.0 9.5 9.0 8.5 8.0 7.5 7.0 6.5 6.0 5.5 5.0 4.5 4.0 3.5 3.0 2.5 2.0 1.5 1.0 0.5 0.0 -0.5 -1.0 -1.5
f1 (ppm)

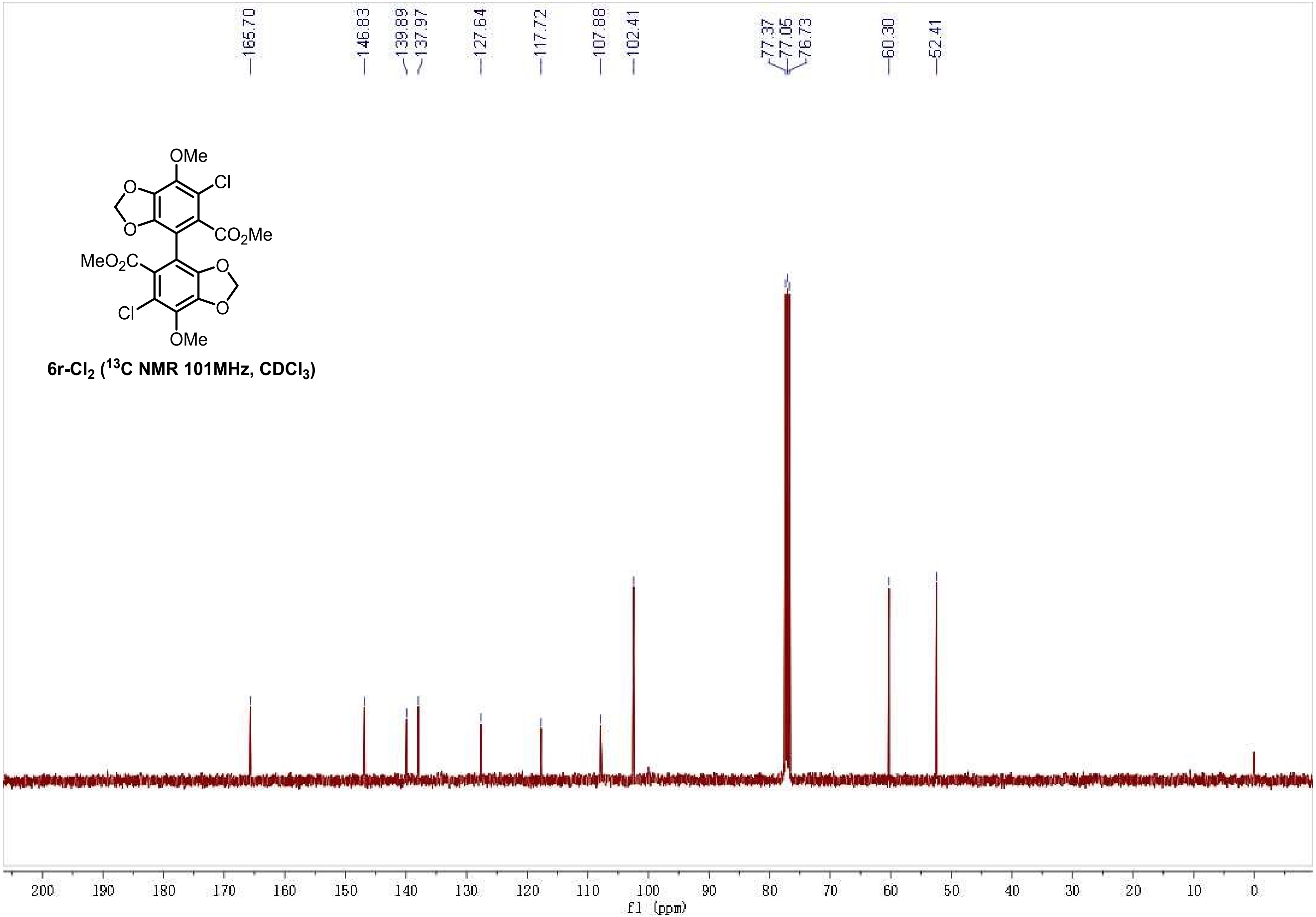

6r-Cl_2 (^{13}C NMR 101MHz, $CDCl_3$)

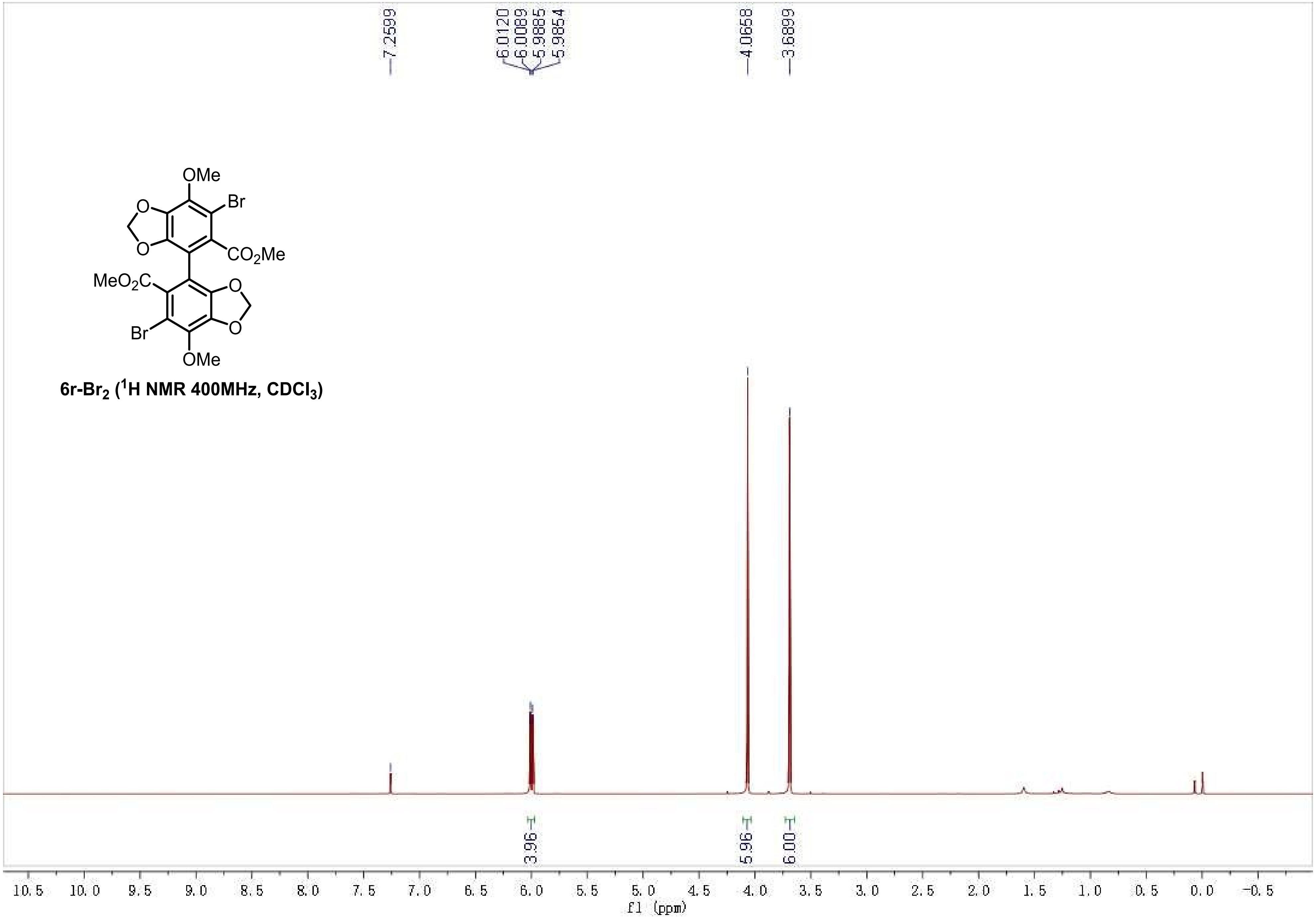

6r-Br$_2$ (^{1}H NMR 400MHz, CDCl$_3$)

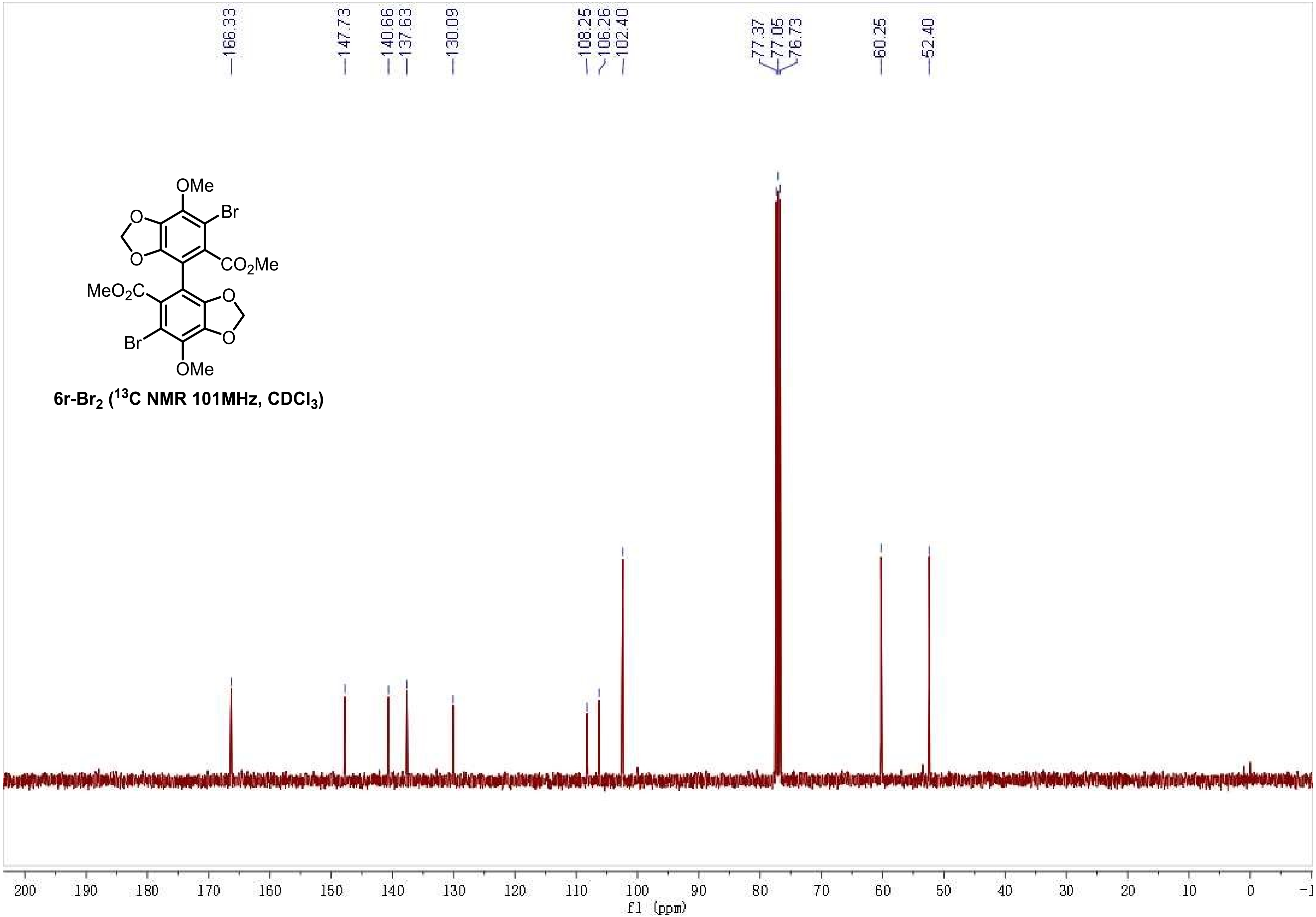

6r-Br$_2$ (^{13}C NMR 101MHz, $CDCl_3$)

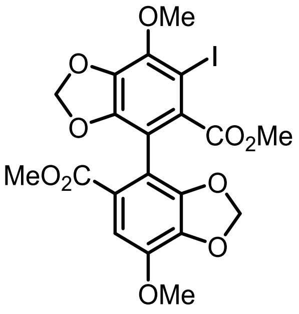

6r-I (^{1}H NMR 400MHz, $CDCl_3$)

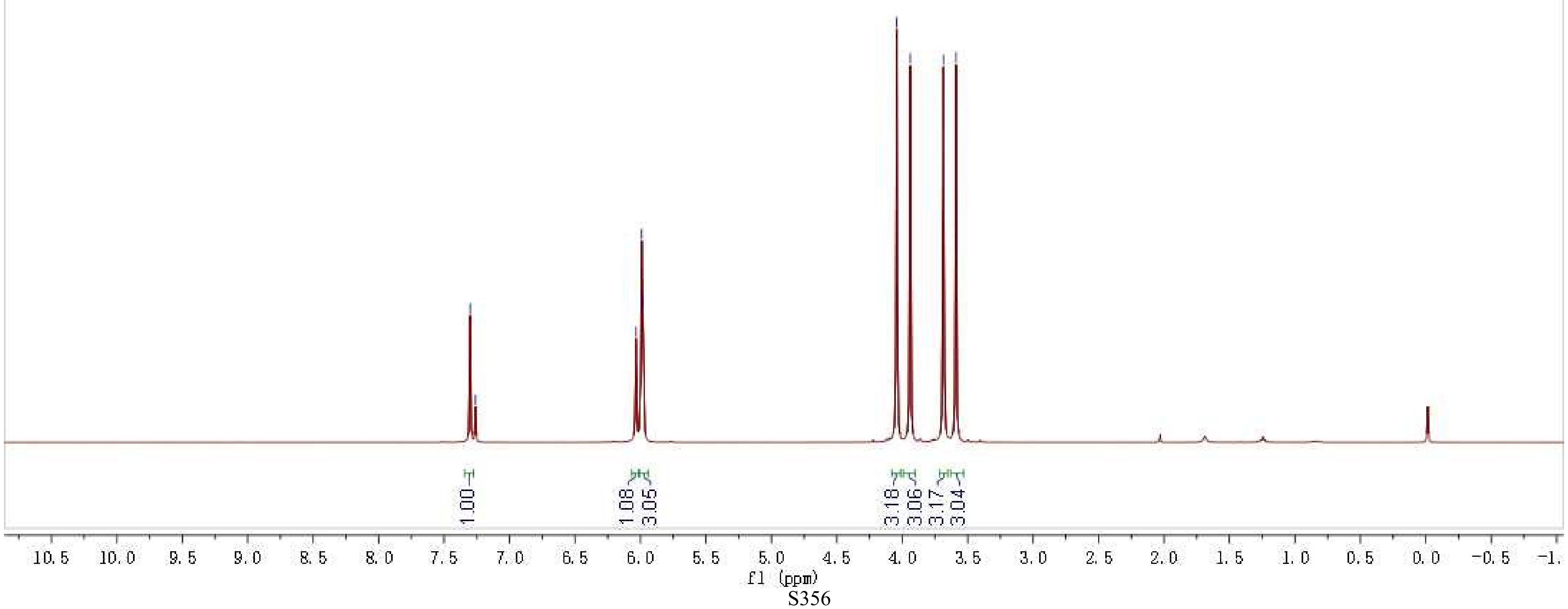

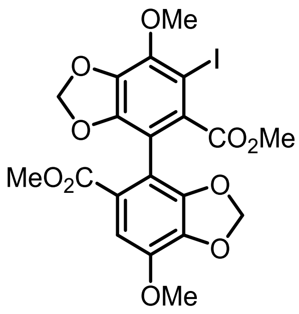

6r-I (^{13}C NMR 101MHz, $CDCl_3$)

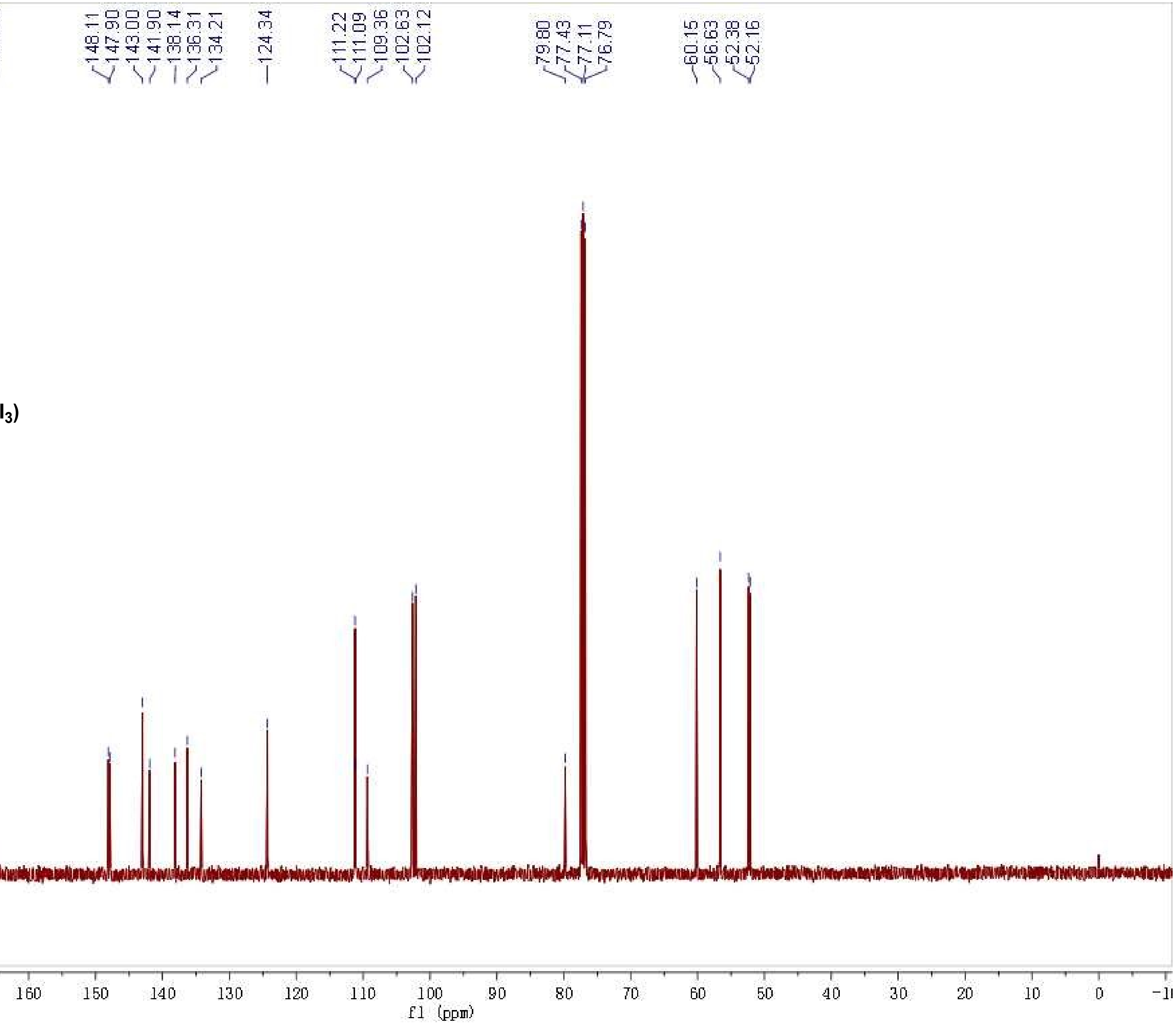

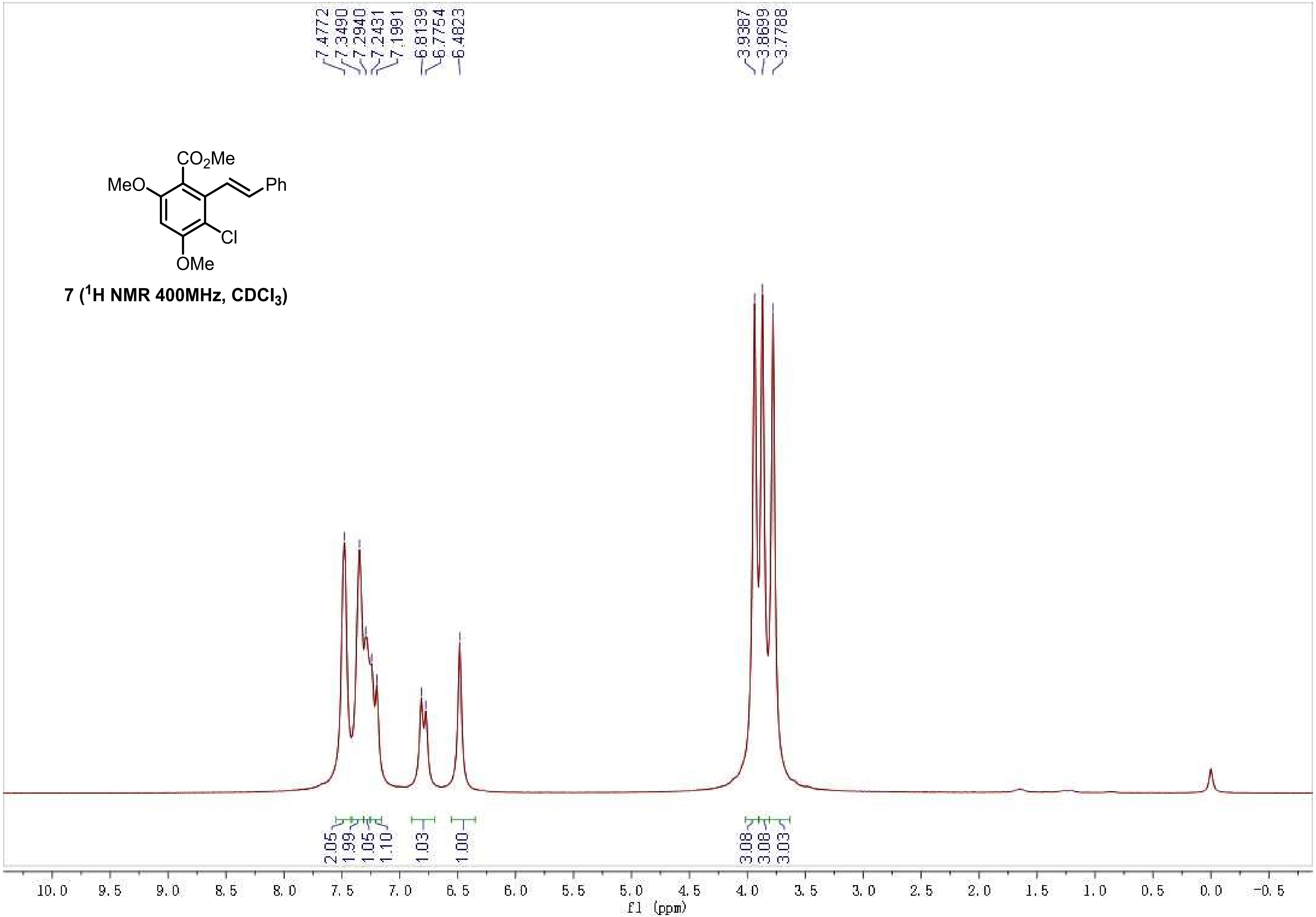

7 (^{1}H NMR 400MHz, $CDCl_3$)

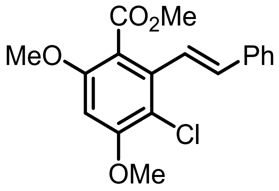

7 (^{13}C NMR 101MHz, $CDCl_3$)

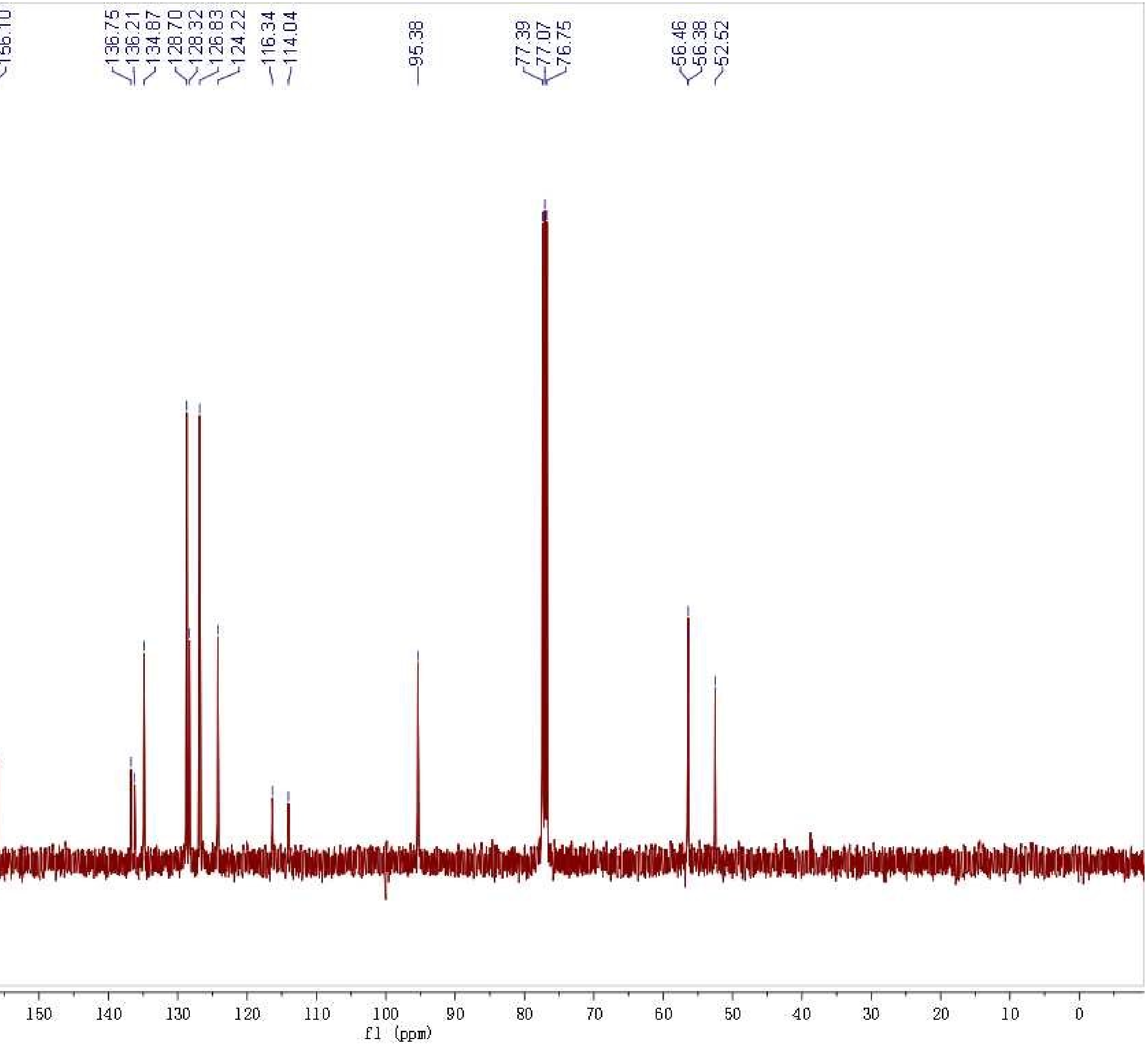

Supplement: SC-015-D4SC04461D-s001 [file SC-015-D4SC04461D-s001.pdf]
